# Clarity Amidst Ambiguity: Towards Precise Definitions in Biological-Informed Disciplines for Enhanced Communication

**DOI:** 10.3390/biomimetics10020076

**Published:** 2025-01-25

**Authors:** Tim Huber, Jörg Müssig

**Affiliations:** 1 Luxembourg Institute of Science and Technology, 4940 Luxembourg, Luxembourg; tim.huber@list.lu; 2 School of Product Design, University of Canterbury, Private Bag 4800, Christchurch 8140, New Zealand; 3 The Biological Materials Group, Department of Biomimetics, HSB—City University of Applied Sciences Bremen, Neustadtswall 30, D-28199 Bremen, Germany

**Keywords:** biological-informed disciplines, meta-analysis, consistency in terminology, biological research frameworks, interdisciplinary communication

## Abstract

This study conducts a meta-analysis of over 1000 abstracts to examine the use and consistency of the terminology in biomimetics, bioinspiration, biomimicry, and bionics, focusing on how these terms impact biological study design. Despite the increasing research in these areas, the ambiguous definitions of key terms complicate study design and interdisciplinary collaboration. The primary aim of this work is to analyse how biological studies in these fields are conceptualised and evaluated, particularly concerning the inconsistent use of terminology. By identifying discrepancies in term usage, we offer refined definitions and practical examples to improve the clarity of study design and research methodologies. Our findings underscore the importance of standardised terminology for ensuring that biological research is accurately designed and executed, leading to more rigorous experimental frameworks and better alignment across disciplines. This meta-analysis reveals how clearer, more consistent terminology can enhance study design in biologically inspired research fields.

## 1. Introduction

The following presents key developments in biologically informed disciplines and the field of biomimetics, highlighting critical steps and addressing the challenges of defining terms within this interdisciplinary field. An important starting point is Nachtigall’s work. Nachtigall [1] analysed terms and meanings and described technical biology and biomimetics as antipodes. They are perspectives that have developed into independent disciplines. In his understanding, technical biology means ’understanding nature with the help of technology’. Biomimetics means ’learning from nature for technology’ [1]. Vincent et al. [2] placed the development of biomimetics within a historical context and summarised that biomimetics, which emerged in the 1950s, bridges biology and technology by transferring biological principles to innovative solutions in engineering, design, and science. They did not propose any conceptual distinctions. Instead, they stated: ‘… biomimetics (which we here mean to be synonymous with ‘biomimesis’, ‘biomimicry’, ‘bionics’, ‘biognosis’, ‘biologically inspired design’, and similar words and phrases implying copying or adaptation or derivation from biology)’ [2]. The following authors went into great detail about interesting concepts from nature that need to be applied to technical issues and described what they meant by biomimetics in this context, without giving precise definitions. According to Bhushan [3], biomimetics uses the evolutionary optimisation of hierarchical structures and processes in nature, from the macro- to the nanoscale, for the development of innovative materials, devices, and technologies, with applications such as self-cleaning, energy efficiency, and high-strength materials. However, the paper goes into less detail on the conceptual categorisations and definitions [3]. In Bar-Cohen’s [4] description of biomimetics, he referred to the varying depth of abstraction of the biological transmission processes. Biomimetics leverages nature’s evolutionary solutions to inspire innovative technologies, ranging from simple designs like swimming fins (making simple copies) to advanced systems like prosthetics and brain-interfaced sensory (greater mimicking complexity) devices, offering the transformative potential for future advancements [4]. With a strong focus on botany and textile-based composites, Milwich et al. [5] used the terms biomimetics and bionics synonymously in their description and described the concepts of biomimetics, utilising the development of a composite material as an example. They used biomimetics to describe nature’s complex, multi-level optimised structures, such as plant stems, to develop innovative, lightweight, and mechanically efficient technical textiles and composites. They also demonstrated the potential of transferring biological principles to technology using modern manufacturing methods [5]. Focusing on materials, Wegst et al. [6] stressed the value of bioinspiration for replicating the complex hierarchical structures of natural materials. These insights guided the development of lighter, stronger, and more resilient manufactured materials, though fully replicating natural properties remains challenging. Staying in the field of biomimetics and materials, the work of Fratzl [7] should be mentioned, who stated that biomimetics in materials science is synonymous with terms such as ‘materials bionics’ and ‘bio-inspired materials research’. He described biomimetics in materials science as a method that translates bioinspiration into innovative, multifunctional materials by systematically analysing structure–function relationships in biological tissues, using strategies such as hierarchical structuring, adaptive growth, and self-healing, and fostering interdisciplinary collaboration with function-oriented knowledge dissemination [7]. For biomimetics in architecture, Gruber and Jeronimidis [8] saw the following approaches: attempts were being made to integrate profound principles of biology—such as material anisotropy, heterogeneity, and adaptive form formation—into design and construction. However, achieving fully biomimetic buildings would require interdisciplinary collaboration and overcoming the challenges of translating biological insights into practical architectural applications [8,9]. For Knippers and Speck [10], biomimetics in architecture extended traditional design approaches by drawing inspiration from natural forms and functions, such as the elastic movements of plants, to develop innovative strategies that balance aesthetics, functionality, and scalability, moving beyond conventional methods and linear abstraction. However, bioinspiration plays an important role in adhesion mechanisms [11]. Gorb [12], for example, focused his attention in biomimetics primarily on the diverse bonding mechanisms in nature and aimed to develop innovative, efficient, and environmentally friendly bonding systems by understanding and imitating the structural, chemical, and mechanical principles of biological bonding devices. However, the biological-inspired disciplines approach is not limited to materials, surfaces, and architecture. As an interdisciplinary bridge between biology and technology, biomimetics offers excellent potential for optimising economic processes due to its high innovation potential and applicability to specialist areas such as economics, especially logistics [13]. In marine biomimetics, Fish [14] saw the great advantage of bioinspiration for product optimisation. Bioinspiration uses the superior swimming abilities of aquatic animals—such as speed, efficiency, manoeuvrability, and camouflage—to develop advanced, bioinspired, autonomous underwater vehicles that can overcome the limitations of conventional marine robotics. Sarikaya [15] explored molecular biomimetics, emphasising surface-specific proteins as templates for controlled material assembly. This approach facilitates the creation of intricate hybrid structures and advances in genetically engineered materials. Nanobiomimetics and nano bioinspiration are considered important areas for the future by Dirks and Brüggemann [16]. Nanobiomimetics harnesses the transformative power of bioinspired innovation at the nanoscale and offers immense potential for technological advances through interdisciplinary collaboration, miniaturisation, and the multidisciplinary education of the next generation of students and researchers [16]. As Wanieck and Beismann [17] described, an essential step for arriving at a definition was the development of guidelines by the Association of German Engineers (VDI), developed by the Biomimetic Group of the VDI Society for Technologies of the Life Sciences [18,19]. Wanieck and Beismann [17] emphasised that standardisation in biomimetics is crucial for interdisciplinary understanding, quality assurance in research, and the successful implementation of biomimetic findings, but was not yet sufficiently established in the scientific literature despite international standards.

However, in science education, the precision of terminology is critical for fostering a deep understanding among students and enabling them to apply these concepts accurately in their academic and professional pursuits. Jacobs [20] conducted a study comparing first-year physics students’ perceived understanding of everyday physics terminology with their actual knowledge and found that, on average, the meaning of more than 15 terms was not correctly understood, which could be a significant obstacle in physics education. The inconsistent use of terms like biomimetics and bioinspiration in educational materials can lead to misconceptions and hinder students’ ability to grasp complex interdisciplinary concepts. Yun and Park [21] emphasised in their work that students’ learning of scientific language leads to the acquisition of the structure of scientific knowledge and thinking. Clear definitions are essential for students transitioning from theoretical learning to practical application in fields that combine biology with technology. When students understand the precise meanings of terms, they can better engage with the material, apply it in practical contexts, and innovate within the field.

This literature review reveals a pervasive issue: the terms biomimetics, bioinspiration, biomimicry, and bionics are often used synonymously and interchangeably, if not randomly. This inconsistency makes it challenging to locate and build upon existing research accurately. For instance, searching for developments in biologically informed disciplines using the term “biomimetics” may yield irrelevant results unless each abstract is individually analysed to ensure it pertains to the intended subject. This issue underscores the need for precise definitions to facilitate accurate literature searches and support interdisciplinary research, both of which are crucial for advancing science education [22,23].

The term “biomimetics” stands out with a more standardised definition within the scientific community. According to ISO 18458 [24], biomimetics refers to the interdisciplinary cooperation of biology and technology to solve technical problems through the abstraction, transfer, and application of knowledge gained from biological models. However, terms such as “bioinspiration,” “biomimicry,” and “bionics” lack universally accepted definitions, often relying on anecdotal descriptions or varying interpretations. For example, bioinspiration typically denotes drawing inspiration from biological systems to fuel creative ideas and innovations, while biomimicry focuses on imitating nature’s designs to solve human problems sustainably. Bionics, on the other hand, involves integrating biological principles into artificial systems.

Fayemi et al. [25] discussed different terms that describe the process of ’learning from nature’, such as bioinspiration, biomimicry, bionics, and biologically inspired design. The authors mentioned that these terms are often treated as synonyms in the scientific literature and gave some examples [2,26,27]. Fayemi et al. [25] considered this appropriate when referring to the end result of these approaches, namely, an invention made possible by knowledge derived from nature. However, they qualified this by saying that differences arise when considering the scope of each term and the development processes. They used the definitions of key terms from the literature but did not explore whether these different areas could be clearly separated. Instead, they focused on biomimetic working models, for which they developed a problem-driven process model. In addition, they proposed a utility tree to guide users in selecting tools based on expertise, making biomimetics more accessible and encouraging its practical implementation [25].

These varying interpretations can lead to significant misunderstandings, particularly in educational settings where the clear communication of concepts is paramount. For students and educators, the lack of standardised definitions can create barriers to learning and teaching, impeding the effective dissemination of knowledge. As science education increasingly incorporates interdisciplinary approaches, the need for precise terminology becomes even more critical.

The combination of two innovative areas—additive manufacturing (often referred to as 3D printing) and biologically informed disciplines—further complicates the terminological landscape. Additive manufacturing involves creating three-dimensional objects by adding a material layer by layer based on a digital model. While all 3D printing is a form of additive manufacturing, the terms are often used interchangeably despite the preference for “additive manufacturing” among engineers [28]. When research combines nature-inspired innovation with additive manufacturing, clear and consistent definitions are essential to ensure effective communication and collaboration across disciplines.

Our research focused on identifying inconsistencies and errors in the usage of terms, such as bionics, biomimetics, biomimicry, and bioinspiration, within the scientific literature. We aimed to determine whether these inconsistencies stem from a lack of clear definitions and to understand the origins of these mistakes. By analysing over 1000 abstracts and conducting detailed studies on terminology, we sought to ascertain if students and researchers possess a clear grasp of the differences between these terms. If gaps in understanding were identified, we aimed to propose solutions to improve clarity and comprehension within the scientific and educational communities. To this end, we developed new definitions for the terms bionics, biomimetics, biomimicry, and bioinspiration, and validated them through quantitative and qualitative analyses.

In this paper, we present the findings of our research efforts, highlighting the inconsistencies in current terminology and providing evidence on how improved and standardised definitions could enhance scientific communication, foster fruitful collaboration, and accelerate progress in nature-inspired innovation and science education. Establishing a common language and understanding is essential for supporting transformative breakthroughs and advancing the interdisciplinary field of biologically informed innovation.

## 2. Materials and Methods

### 2.1. Search Strategy

To test the accuracy of the established first set of definitions and to better understand the use of the terms in the scientific literature, we collected 3877 abstracts using an Octoparse (Octopus Data Inc., www.octoparse.com) web crawler algorithm and the terms “biomimetics”, “bionics”, “biomimicry”, and “bioinspired”, each in combination with the term “3D printing”. The abstracts were collected from Google Scholar between the 5th and 9th of July 2019. A more detailed description of the search queries and references for the collected abstracts can be found in the supplementary information.

An overview of the abstracts found by the search query is given in Table 1. After removing duplicates, between 225 and 469 abstracts were used, depending on each term used. 

The complete list of collected abstracts for each category is in the supplementary materials.

### 2.2. Screening and Categorisation

**Figure 1:**
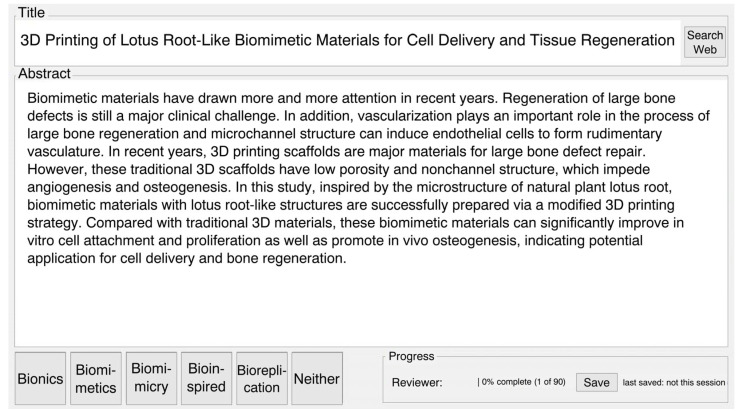
The GUI for assigning abstracts to the categories bioinspired, biomimetics, biomimicry, bionics, or neither.

All information except for the publication and the abstract were removed, and the remaining data were copied into CSV files. The CSV files were imported into R (v 3.5.0), and a code was used that displayed each abstract and title using a graphical interface that also displayed five buttons labelled “Biomimetics”, “Biomimicry”, “Bionics”, “Bioinspired”, and “Neither”. The code generated a new CSV file with the abstract title, the abstract, the name of the reviewer, and the label of the button that was clicked after the abstract had been categorised by the reviewer (Figure 1). The code for the GUI and list of used libraries are available in the supplementary material.

Both authors read the abstracts independently and assigned them to one of the definitions or “neither”, and the results were compared (for results, see Section 3). Discrepancies were identified, and extensions and adjustments were made to the definitions (for results, see Section 3). The expanded and additionally introduced terms were tested by randomly selecting and re-categorising 90 of the previously collected 1259 abstracts. An additional button labelled “Bioreplication” was added to the original user interface and underlying R-code. The definitions were further refined and tested with more participants based on extensive discussions (for results, see Section 3).

Six participants were selected from the staff and six participants from the student body of the Biomimetics Degree Course at the City University of Applied Science Bremen, Germany, and supplied with slightly refined definitions of the terms “Biomimetics”, “Bionics”, “Biomimicry”, “Bioreplication“, and “Bioinspired”. Eighty of the previously collected 1259 abstracts were randomly collected, and the participants were asked to categorise them as either one of the defined terms or as “Neither” (for results, see Section 3). To better understand the difficulties they had with these terms, 2 group interviews were scheduled with 3 participants each.

In all cases, data were processed, and statistical tests were performed with R (v 3.5.0).

### 2.3. Qualitative Analysis

Using the study results and the interviews, we developed more precise definitions and created practical examples for each term to enable better understanding within the scientific community.

## 3. Results and Discussion

### 3.1. Definition Finding

Based on an extensive literature review, we formulated the following definitions for biomimetics, biomimicry, bionics, and bioinspiration as a foundational step.

- **Biomimetics:** interdisciplinary cooperation of biology and technology aimed at solving technical problems through the abstraction, transfer, and application of knowledge gained from biological models [24].
- **Biomimicry:** a transdisciplinary approach for studying and understanding the concepts found in nature and replicating them to solve human problems while adhering to ecological standards for sustainable development [24,29,30,31].
- **Bionics:** a transdisciplinary approach combining biology and engineering, focusing on robotics, medicine, prosthetics, and electronics to study, copy, and apply biological functions or systems to create innovative products or systems functioning in analogy with living organisms [24,31,32].
- **Bioinspiration:** a methodology where biological systems and living organisms are studied to draw general analogies for application to human-made solutions and industrial challenges [24,33,34,35].

**Figure 2:**
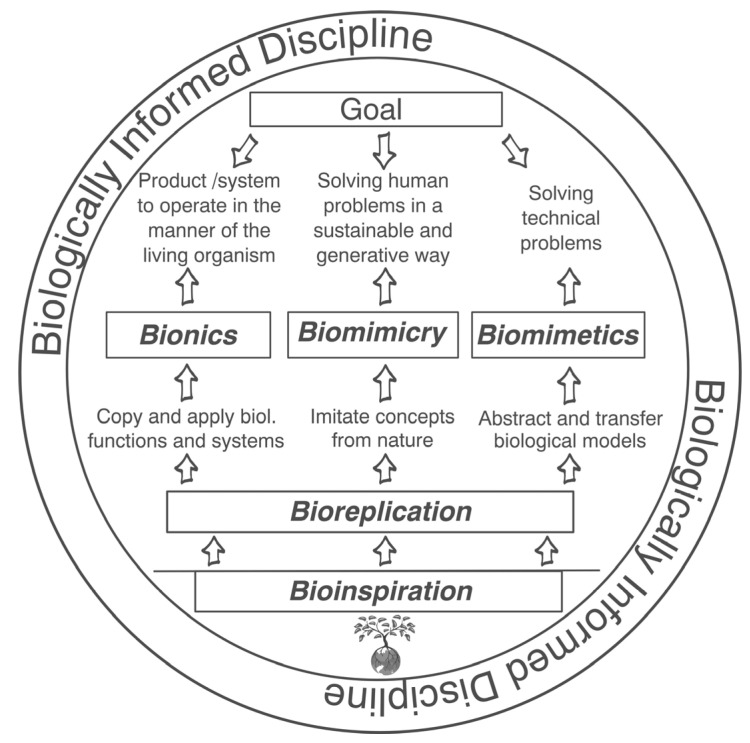
A visual presentation of biologically informed disciplines to distinguish better between the terms bioinspiration, bionics, biomimetics, biomimicry, and bioreplication.

In addition, we created a visual illustration (see Figure 2) showing the intricate interplay of the key terms in biologically informed disciplines. Bioinspiration, bionics, biomimetics, biomimicry, and bioreplication are commonly used terms, yet their precise distinctions remain elusive. Our illustration represents these terms and facilitates a clearer understanding of their individual characteristics and distinctions.

### 3.2. Analysis of Collected Abstracts

**Figure 3:**
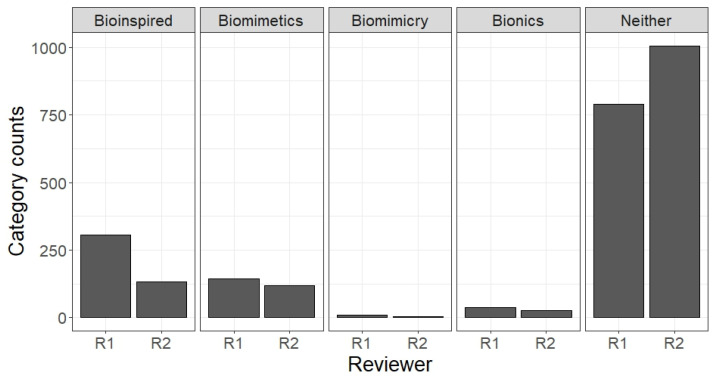
The analysis of the distribution of responses when assigning abstracts to the categories bioinspired, biomimetics, biomimicry, bionics, or neither.

A total of 3877 abstracts were collected using a web crawler algorithm, focusing on the terms “biomimetics,” “bioinspiration,” “biomimicry,” and “bionics” combined with “3D printing.” Of these, 1259 abstracts were selected, read, and categorised according to the above definitions or marked as “Neither” if they did not fit. Significant discrepancies were observed, particularly between the categories “Bioinspiration” and “Neither,” where biological structures were replicated without a clear abstraction of the biological principles (see Figure 3).

Given the challenges to categorising the works accurately, an additional term, “Bioreplication”, was introduced and defined as follows:

- **Bioreplication**: the production of copies of complex biological structures or systems using man-made technology and materials.

**Figure 4:**
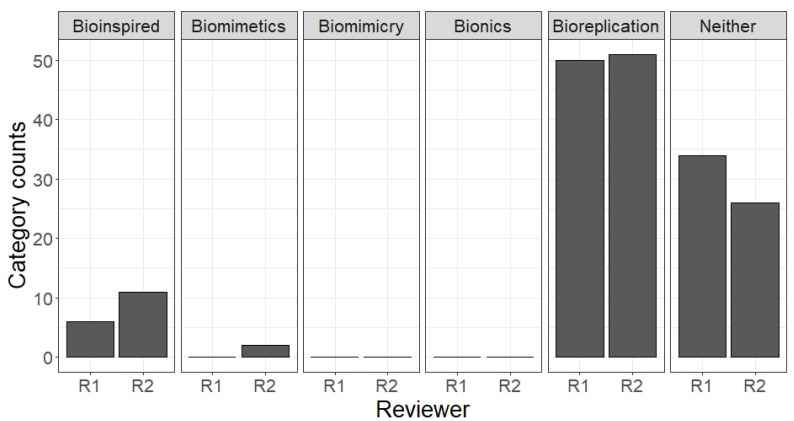
The analysis of the distribution of responses when assigning abstracts to the categories bioinspired, biomimetics, biomimicry, bionics, neither, or the added category bioreplication.

To validate this new term, 90 of the previously collected abstracts were randomly selected and re-categorised, showing improved coherence between the reviewers, with many abstracts fitting the “Bioreplication” category (see Figure 4).

### 3.3. Definition Refinement

The largest discrepancies occurred between “bioinspiration” and “Neither.” To address this, the definition of “bioinspiration” was refined. Additionally, the distinction between “bionics” and “bioreplication” was clarified, with examples to illustrate the differences. Table 2 presents an overview of the refined definitions.

### 3.4. Quantitative and Qualitative Assessments

**Figure 5:**
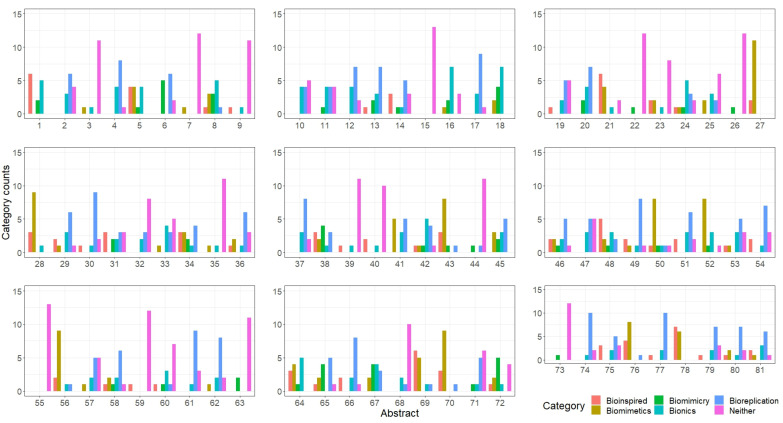
Evaluation of the survey of study participants with a biomimetics background; six participants were selected from the scientific staff and six participants from the student body of the biomimetics programme. The number of evaluated abstracts are shown on the x-axis and how many participants placed the abstracts into one of the 6 available categories are shown on the y-axis.

The refined definitions were tested with study participants from the Biomimetics Degree Course at the City University of Applied Science Bremen, Germany. Six staff members and six students were provided with the refined definitions and asked to categorise 80 abstracts. There was high agreement (>50%) for “Neither,” “Biomimetics,” and “Bioreplication,” but less consensus for “Bionics,” “Bioinspired,” and “Biomimicry” (see Figure 5).

**Figure 6:**
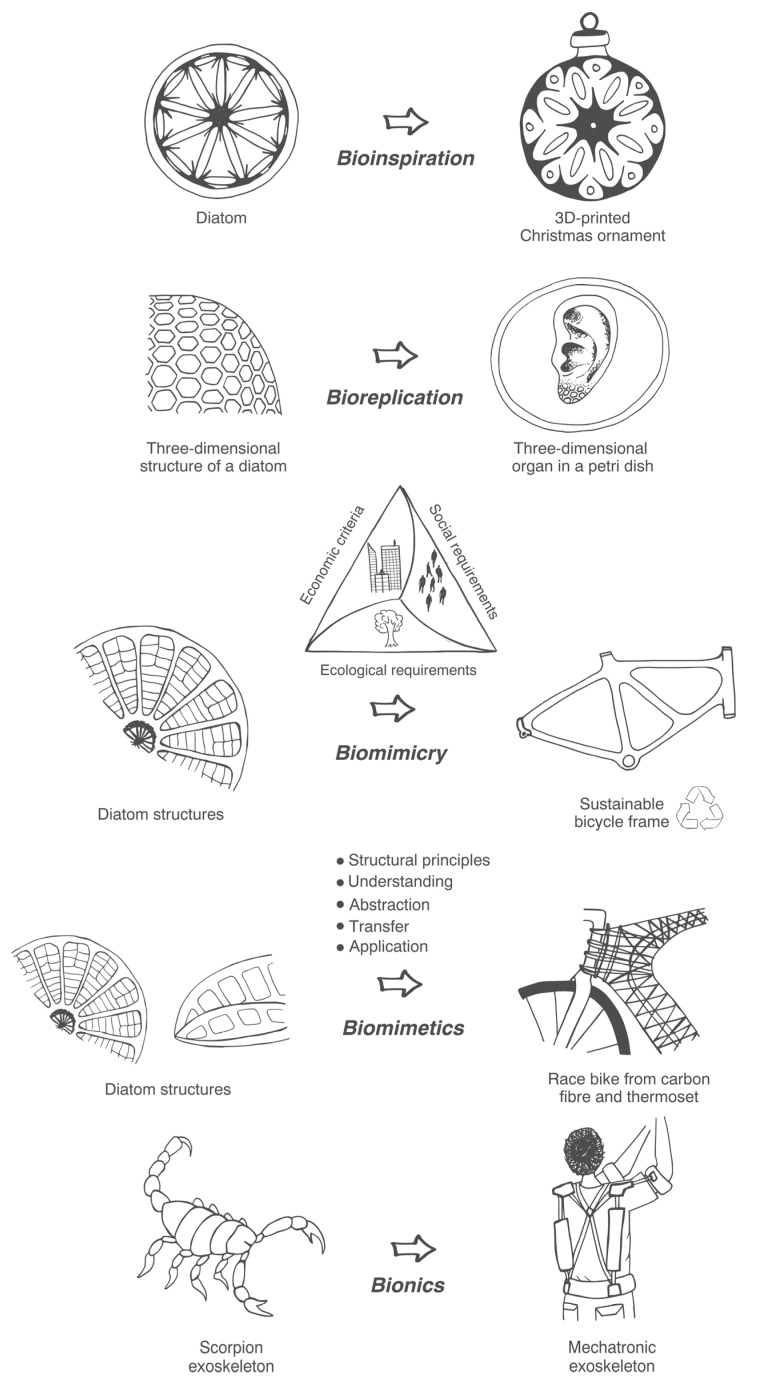
Visualisation of the concepts of bioinspiration, bioreplication, biomimicry, biomimetics, and bionics.

Two group interviews were conducted with three participants each to further understand the difficulties. While the interviewees understood the theoretical differences between the terms, they found practical differentiation challenging without clear examples. Thus, the following examples are proposed to clarify the differences between the provided definitions. The examples provided in Figure 6 are fictional and were developed to represent the core attributes of the proposed definitions visually. They are intended to serve as illustrative aids rather than representations of existing biologically inspired designs.

- **Bioinspiration:** A diatom is a single-celled organism belonging to the group of microalgae known as Bacillariophyta. Diatoms are characterised by their unique cell walls, called frustules, which are made of silica and have intricate patterns and structures. They are found in various aquatic environments, including oceans, lakes, rivers, and even damp soil. Diatoms exhibit a remarkable diversity, with tens of thousands of known species. One can draw inspiration from the intricate, complex, and arguably beautiful patterns to create and 3D print a new Christmas ornament.
- **Bioreplication:** one could study the three-dimensional structure of one species of diatom and replicate it using a three-dimensional printed gel to create a tissue scaffold with the aim of providing a structural framework that supports the growth and organisation of cells, enabling the formation of functional three-dimensional (3D) tissues or organs in tissue engineering and regenerative medicine.
- **Biomimicry:** Diatoms are very lightweight, optimised to withstand high compressive loads, and absorb large vibrations. One could design a bicycle frame for a city bike using diatom structures as the inspiration to reduce the bike’s mass and improve riding comfort, produce the frame locally according to high sustainable standards and from sustainable materials, to convince more people to ride bikes and use their cars less.
- **Biomimetics:** a bike frame could be designed using a diatom structure to reduce mass, improve stiffness, and reduce vibration absorption to create a high-performance race bike from carbon fibre and thermoset resins to gain a competitive advantage in professional bike racing.
- **Bionics:** An exoskeleton is a rigid external structure that surrounds and supports the body of certain organisms. It is primarily found in invertebrates, such as insects, crustaceans, and some arachnids. The exoskeleton provides structural support to an organism’s body, allowing it to maintain its shape and posture. Muscles attach to the exoskeleton, enabling movement and locomotion. One could create a mechatronic exoskeleton according to the same principles to support workers who need to lift heavy loads repeatedly.

## 4. Conclusions

Our study reveals a critical issue within the scientific community: the inconsistent use and understanding of terms, such as bionics, biomimetics, biomimicry, and bioinspiration. We uncovered significant inconsistencies in the use of these terms across the scientific literature, which hampers effective communication and collaborative efforts in nature-inspired innovation.

Our findings underscore the need for adopting standardised definitions and terminology in biologically informed innovation. These terms are often used interchangeably without clear distinctions, leading to significant confusion and misinterpretation. For instance, biomimetics and biomimicry are frequently conflated despite having distinct meanings and implications. This semantic ambiguity creates barriers to understanding and collaboration, highlighting the need for clear and distinct definitions.

Furthermore, our research indicates that this issue extends into education, where students struggle to grasp the nuances between these terms. This lack of clarity hinders their ability to apply these concepts accurately in their studies and poses a significant obstacle to their future careers. Standardising terminology could enhance science education, providing students with a solid foundation in these critical concepts, fostering innovation, and leading to significant advancements in biologically informed disciplines and engineering.

Our study performs the following:

- Introduces and validates the term “bioreplication” alongside refined definitions for biomimetics, biomimicry, bionics, and bioinspiration to address the classification gaps and enhance clarity in biologically inspired disciplines.
- Demonstrates how adopting standardised definitions can streamline literature searches, improve publication clarity, and foster interdisciplinary collaboration across biology, engineering, and design.
- Highlights the role of precise definitions in improving science education, equipping students and educators with the tools for a better understanding and practical application of biologically inspired concepts.
- Provides real-world examples, such as bioinspired design in 3D printing, showcasing how clarified terminologies facilitate innovation in robotics, materials science, and architecture.
- Advocates for the global adoption of these definitions and proposes future research directions, including longitudinal studies and the exploration of emerging fields like nanobiomimetics and AI-driven terminology analysis.

This research lays the foundation for a unified framework that will enhance clarity, precision, and interdisciplinary collaboration, fostering transformative breakthroughs in biologically informed innovation by addressing these inconsistencies.

## Figures and Tables

**Table 1:** Search queries used for the web crawler algorithm, number of abstracts found using those terms, the year range of publication for the abstracts in each category, and the number of abstracts selected for each category.

Query	Number of Collected Abstracts	Publication Years	Number of Selected Abstracts
bionics AND 3D printing	999	1994–2019	225
biomimetics AND 3D printing	878	1995–2019	304
bioinspired 3D printing	1000	1976–2019	469
biomimicry AND 3D printing	1000	1953–2019	261

**Table 2:** The initial definitions of “Biomimetics”, “Biomimicry”, “Bionics”, “Bioinspiration”, and “Bioreplication” developed by the authors and the corresponding refinement after the first round of assessment.

Term	1st Definition	2nd Definition
**BIOMIMETICS**	Interdisciplinary cooperation of biology and technology with the goal of solving technical problems through the abstraction, transfer, and application of knowledge gained from biological models.	Interdisciplinary cooperation of biology and technology with the goal of solving technical problems through the abstraction, transfer, and application of knowledge gained from biological models.
**BIOMIMICRY**	Biomimicry (biomimetism) is a transdisciplinary approach to study and understand concepts found in nature and to imitate (replicate) those concepts to solve human problems adhering to an ecological standard to achieve a solution that fulfils all requirements of sustainable development (social, environmental, and economic).	Biomimicry (biomimetism) is a transdisciplinary approach to study and understand concepts found in nature and to imitate (replicate) those concepts to solve human problems adhering to an ecological standard to achieve a solution that fulfils all requirements of sustainable development (social, environmental, and economic).
**BIONICS**	Bionics is a transdisciplinary approach combing biology and engineering disciplines, focused primarily on robotics, medicine, prosthetic fittings, and electronics, to study, copy, and apply biological functions or systems with the goal of creating innovative functional products or systems which function in analogy with the studied living organisms.	Bionics is a transdisciplinary approach which combines biology and engineering disciplines, focussing primarily on robotics, medicine, prosthetic fittings, and electronics. Bionics aims to study, copy, and apply biological functions or systems to create products or systems which replicate, increase, or replace such functions in analogy with the studied living organisms. However, these products or systems are not one-to-one substitutes for the product/system to be replaced and always aim to improve the functionality of the product/system.
**BIOINSPIRATION**	Bioinspiration is a methodology in which biological systems and living organisms are studied to draw general analogies, which in turn are used in man-made applications and industrial challenges.	Bioinspiration is a method with the objective of drawing general analogies based on the study of biological systems and living organisms, which in turn are used in man-made applications and industrial challenges.
**BIOREPLICATION**	Bioreplication is the production of copies of complex biological structures or biological systems using made-man technology and materials.	

## Data Availability

The data can be made available upon request.

## Supplementary Materials

The following supporting information can be downloaded at: https://www.mdpi.com/article/10.3390/biomimetics10020076/s1, R-code for the GUI: Rcode.txt, Search query results: S1.pdf
