# Supplementary material for: Clarity Amidst Ambiguity: Towards Precise Definitions in Biological-Informed Disciplines for Enhanced Communication
Source: Biomimetics (Basel). 2025 Jan 25;10(2):76. doi: 10.3390/biomimetics10020076 (PMC11853458; doi:10.3390/biomimetics10020076)
Supplement: Supplementary file 1 [file biomimetics-10-00076-s001.zip › S1.pdf]

S1: Search query results

biomimetics AND 3d-printing
Publish or Perish 7.10.2373.7118
Windows (x64) edition, running on Windows 10.0.16299 (x64)
Search terms
Keywords: biomimetics AND 3d-printing
Years: all

Data retrieval
Data source: Google Scholar
Search date: 2019-07-05 13:52:53 +1200
Cache date: 2019-07-05 14:14:16 +1200
Search result: [0] No error

Metrics
Reference date: 2019-07-05 14:14:16 +1200
Publication years: 1995-2019
Citation years: 24 (1995-2019)
Papers: 878
Citations: 66104
Citations/year: 2754.33
Citations/paper: 75.29 (acc1=843, acc2=770, acc5=591, acc10=387, acc20=183)
Authors/paper: 4.13/4.0/4 (mean/median/mode)
Age-weighed citation rate: 12642.62 (sqrt=112.44), 3575.40/author
Hirsch h-index: 134 (a=3.68, m=5.58, 43946 cites=66.5% coverage)
Egghe g-index: 230 (g/h=1.72, 53222 cites=80.5% coverage)
PoP hI,norm: 70
PoP hI,annual: 2.92

Results
AS Gladman, EA Matsumoto, RG Nuzzo, ... (2016) Biomimetic 4D printing. Nature materials, nature.com, cited by 749 (249.67 per year)
TQ Huang, X Qu, J Liu, S Chen (2014) 3D printing of biomimetic microstructures for cancer cell migration. Biomedical microdevices, Springer, cited by 115 (23.00 per year)
F Pati, DH Ha, J Jang, HH Han, JW Rhie, DW Cho (2015) Biomimetic 3D tissue printing for soft tissue regeneration. Biomaterials, Elsevier, cited by 154 (38.50 per year)
Y Yang, Z Chen, X Song, Z Zhang, J Zhang, ... (2017) Biomimetic anisotropic reinforcement architectures by electrically assisted nanocomposite 3D printing. Advanced …, Wiley Online Library, cited by 53 (26.50 per year)
JM Taboas, RD Maddox, PH Krebsbach, SJ Hollister (2003) Indirect solid free form fabrication of local and global porous, biomimetic and composite 3D polymer-ceramic scaffolds. Biomaterials, Elsevier, cited by 721 (45.06 per year)
W Zhu, X Ma, M Gou, D Mei, K Zhang, ... (2016) 3D printing of functional biomaterials for tissue engineering. Current opinion in …, Elsevier, cited by 153 (51.00 per year)

P Zhang, MA Heyne, AC To (2015) Biomimetic staggered composites with highly enhanced energy dissipation: Modeling, 3D printing, and testing. Journal of the Mechanics and Physics of …, Elsevier, cited by 45 (11.25 per year)
DE Ingber, VC Mow, D Butler, L Niklason, ... (2006) Tissue engineering and developmental biology: going biomimetic. Tissue …, liebertpub.com, cited by 243 (18.69 per year)
AV Do, B Khorsand, SM Geary, ... (2015) 3D printing of scaffolds for tissue regeneration applications. Advanced healthcare …, Wiley Online Library, cited by 262 (65.50 per year)
BG Compton, JA Lewis (2014) 3D-printing of lightweight cellular composites. Advanced materials, Wiley Online Library, cited by 557 (111.40 per year)
AL Rutz, KE Hyland, AE Jakus, ... (2015) A multimaterial bioink method for 3D printing tunable, cell-compatible hydrogels. Advanced …, Wiley Online Library, cited by 219 (54.75 per year)
EE de Obaldia, C Jeong, LK Grunenfelder, ... (2015) Analysis of the mechanical response of biomimetic materials with highly oriented microstructures through 3D printing, mechanical testing and modeling. Journal of the …, Elsevier, cited by 43 (10.75 per year)
JY Lee, B Choi, B Wu, M Lee (2013) Customized biomimetic scaffolds created by indirect three-dimensional printing for tissue engineering. Biofabrication, iopscience.iop.org, cited by 104 (17.33 per year)
BN Peele, TJ Wallin, H Zhao, ... (2015) 3D printing antagonistic systems of artificial muscle using projection stereolithography. … & biomimetics, iopscience.iop.org, cited by 77 (19.25 per year)
W Zhang, Q Lian, D Li, K Wang, D Hao, W Bian, ... (2015) The effect of interface microstructure on interfacial shear strength for osteochondral scaffolds based on biomimetic design and 3D printing. Materials Science and …, Elsevier, cited by 31 (7.75 per year)
CB Highley, CB Rodell, JA Burdick (2015) Direct 3D printing of shear-thinning hydrogels into self-healing hydrogels. Advanced Materials, Wiley Online Library, cited by 308 (77.00 per year)
C Kucukgul, SB Ozler, I Inci, E Karakas, ... (2015) 3D bioprinting of biomimetic aortic vascular constructs with self-supporting cells. Biotechnology and …, Wiley Online Library, cited by 76 (19.00 per year)
LS Dimas, GH Bratzel, I Eylon, ... (2013) Tough composites inspired by mineralized natural materials: computation, 3D printing, and testing. Advanced Functional …, Wiley Online Library, cited by 204 (34.00 per year)
NJ Castro, J O'Brien, LG Zhang (2015) Integrating biologically inspired nanomaterials and table-top stereolithography for 3D printed biomimetic osteochondral scaffolds. Nanoscale, pubs.rsc.org, cited by 81 (20.25 per year)
C Feng, W Zhang, C Deng, G Li, J Chang, ... (2017) 3D printing of lotus root-like biomimetic materials for cell delivery and tissue regeneration. Advanced …, Wiley Online Library, cited by 23 (11.50 per year)
W Wu, A DeConinck, JA Lewis (2011) Omnidirectional printing of 3D microvascular networks. Advanced materials, Wiley Online Library, cited by 360 (45.00 per year)
Y Yang, X Li, X Zheng, Z Chen, Q Zhou, ... (2018) 3D-Printed Biomimetic Super-Hydrophobic Structure for Microdroplet Manipulation and Oil/Water Separation. Advanced …, Wiley Online Library, cited by 72 (72.00 per year)
X Ma, X Qu, W Zhu, YS Li, S Yuan, ... (2016) Deterministically patterned biomimetic human iPSC-derived hepatic model via rapid 3D bioprinting. Proceedings of the …, National Acad Sciences, cited by 205 (68.33 per year)

M Costantini, J Idaszek, K Szöke, J Jaroszewicz, ... (2016) 3D bioprinting of BM-MSCs-loaded ECM biomimetic hydrogels for in vitro neocartilage formation. …, iopscience.iop.org, cited by 68 (22.67 per year)
D Chimene, KK Lennox, RR Kaunas, ... (2016) Advanced bioinks for 3D printing: a materials science perspective. Annals of biomedical …, Springer, cited by 126 (42.00 per year)
M Gou, X Qu, W Zhu, M Xiang, J Yang, K Zhang, ... (2014) Bio-inspired detoxification using 3D-printed hydrogel nanocomposites. Nature …, nature.com, cited by 155 (31.00 per year)
K Markstedt, A Escalante, G Toriz, ... (2017) Biomimetic inks based on cellulose nanofibrils and cross-linkable xylans for 3D printing. ACS applied materials …, ACS Publications, cited by 20 (10.00 per year)
A Bowyer (2014) 3D printing and humanity's first imperfect replicator. 3D printing and additive manufacturing, liebertpub.com, cited by 99 (19.80 per year)
AP Zhang, X Qu, P Soman, KC Hribar, ... (2012) Rapid fabrication of complex 3D extracellular microenvironments by dynamic optical projection stereolithography. Advanced …, Wiley Online Library, cited by 181 (25.86 per year)
B Zhang, X Pei, C Zhou, Y Fan, Q Jiang, A Ronca, ... (2018) The biomimetic design and 3D printing of customized mechanical properties porous Ti6Al4V scaffold for load-bearing bone reconstruction. Materials & Design, Elsevier, cited by 21 (21.00 per year)
C Minas, D Carnelli, E Tervoort, ... (2016) 3D printing of emulsions and foams into hierarchical porous ceramics. Advanced Materials, Wiley Online Library, cited by 88 (29.33 per year)
L Wen, JC Weaver, GV Lauder (2014) Biomimetic shark skin: design, fabrication and hydrodynamic function. Journal of Experimental Biology, jeb.biologists.org, cited by 185 (37.00 per year)
NA Sears, DR Seshadri, PS Dhavalikar, ... (2016) A review of three-dimensional printing in tissue engineering. … Engineering Part B …, liebertpub.com, cited by 116 (38.67 per year)
GX Gu, M Takaffoli, AJ Hsieh, MJ Buehler (2016) Biomimetic additive manufactured polymer composites for improved impact resistance. Extreme Mechanics Letters, Elsevier, cited by 68 (22.67 per year)
MI Abid, L Wang, QD Chen, XW Wang, ... (2017) Angle-multiplexed optical printing of biomimetic hierarchical 3D textures. Laser & Photonics …, Wiley Online Library, cited by 18 (9.00 per year)
G Brunello, S Sivolella, R Meneghello, L Ferroni, ... (2016) Powder-based 3D printing for bone tissue engineering. Biotechnology …, Elsevier, cited by 61 (20.33 per year)
N Sears, P Dhavalikar, M Whitely, ... (2017) Fabrication of biomimetic bone grafts with multi-material 3D printing. …, iopscience.iop.org, cited by 13 (6.50 per year)
BS Kim, JS Lee, G Gao, DW Cho (2017) Direct 3D cell-printing of human skin with functional transwell system. Biofabrication, iopscience.iop.org, cited by 47 (23.50 per year)
K Zhu, SR Shin, T van Kempen, YC Li, ... (2017) Gold nanocomposite bioink for printing 3D cardiac constructs. Advanced functional …, Wiley Online Library, cited by 56 (28.00 per year)
J Vanderburgh, JA Sterling, SA Guelcher (2017) 3D Printing of Tissue Engineered Constructs for In Vitro Modeling of Disease Progression and Drug Screening. Annals of biomedical …, Springer, cited by 43 (21.50 per year)
K Zhang, Q Fu, J Yoo, X Chen, P Chandra, X Mo, ... (2017) 3D bioprinting of urethra with PCL/PLCL blend and dual autologous cells in fibrin hydrogel: An in vitro evaluation of biomimetic mechanical property and cell …. Acta biomaterialia, Elsevier, cited by 48 (24.00 per year)

F Libonati, GX Gu, Z Qin, L Vergani, ... (2016) Bone-inspired materials by design: toughness amplification observed using 3D printing and testing. Advanced Engineering …, Wiley Online Library, cited by 57 (19.00 per year)
S Knowlton, B Yenilmez, S Tasoglu (2016) Towards single-step biofabrication of organs on a chip via 3D printing. Trends in biotechnology, Elsevier, cited by 32 (10.67 per year)
J Warner, P Soman, W Zhu, M Tom, ... (2016) Design and 3D printing of hydrogel scaffolds with fractal geometries. … Biomaterials Science & …, ACS Publications, cited by 25 (8.33 per year)
Y Xu, X Wang (2015) Application of 3D biomimetic models in drug delivery and regenerative medicine. Current pharmaceutical design, ingentaconnect.com, cited by 28 (7.00 per year)
D Correa, A Papadopoulou, C Guberan, ... (2015) 3D-printed wood: programming hygroscopic material transformations. 3D Printing and …, liebertpub.com, cited by 40 (10.00 per year)
JG Torres-Rendon, T Femmer, L De Laporte, ... (2015) Bioactive gyroid scaffolds formed by sacrificial templating of nanocellulose and nanochitin hydrogels as instructive platforms for biomimetic tissue engineering. Advanced …, Wiley Online Library, cited by 93 (23.25 per year)
W Sun, B Starly, A Darling, ... (2004) Computer-aided tissue engineering: application to biomimetic modelling and design of tissue scaffolds. … and applied biochemistry, Wiley Online Library, cited by 218 (14.53 per year)
YJ Yoon, SK Moon, J Hwang (2014) 3D printing as an efficient way for comparative study of biomimetic structures—trabecular bone and honeycomb. Journal of Mechanical Science and …, Springer, cited by 11 (2.20 per year)
B Ward-Cherrier, N Pestell, L Cramphorn, B Winstone, ... (2018) The tactip family: Soft optical tactile sensors with 3d-printed biomimetic morphologies. Soft robotics, liebertpub.com, cited by 28 (28.00 per year)
S Panzavolta, P Torricelli, S Amadori, ... (2013) 3D interconnected porous biomimetic scaffolds: In vitro cell response. … Research Part A: An …, Wiley Online Library, cited by 36 (6.00 per year)
JC Culver, JC Hoffmann, RA Poché, ... (2012) Three-dimensional biomimetic patterning in hydrogels to guide cellular organization. Advanced …, Wiley Online Library, cited by 139 (19.86 per year)
KA Heintz, ME Bregenzer, JL Mantle, ... (2016) Fabrication of 3D biomimetic microfluidic networks in hydrogels. Advanced …, Wiley Online Library, cited by 25 (8.33 per year)
BAE Lehner, DT Schmieden, AS Meyer (2017) A straightforward approach for 3D bacterial printing. ACS synthetic biology, ACS Publications, cited by 31 (15.50 per year)
M Nakamura, A Kobayashi, F Takagi, ... (2005) Biocompatible inkjet printing technique for designed seeding of individual living cells. Tissue …, liebertpub.com, cited by 417 (29.79 per year)
FY Hsieh, S Hsu (2015) 3D bioprinting: a new insight into the therapeutic strategy of neural tissue regeneration. Organogenesis, Taylor & Francis, cited by 37 (9.25 per year)
B Mosadegh, G Xiong, S Dunham, JK Min (2015) Current progress in 3D printing for cardiovascular tissue engineering. Biomedical Materials, iopscience.iop.org, cited by 79 (19.75 per year)
CY Liaw, M Guvendiren (2017) Current and emerging applications of 3D printing in medicine. Biofabrication, iopscience.iop.org, cited by 56 (28.00 per year)

W Zhu, C O'Brien, JR O'Brien, LG Zhang (2014) 3D nano/microfabrication techniques and nanobiomaterials for neural tissue regeneration. Nanomedicine, Future Medicine, cited by 78 (15.60 per year)
H Seitz, W Rieder, S Irsen, B Leukers, ... (2005) Three-dimensional printing of porous ceramic scaffolds for bone tissue engineering. Journal of Biomedical …, Wiley Online Library, cited by 653 (46.64 per year)
X Qu, P Xia, J He, D Li (2016) Microscale electrohydrodynamic printing of biomimetic PCL/nHA composite scaffolds for bone tissue engineering. Materials Letters, Elsevier, cited by 15 (5.00 per year)
A Szojka, K Lalh, SHJ Andrews, NM Jomha, M Osswald, ... (2017) Biomimetic 3D printed scaffolds for meniscus tissue engineering. Bioprinting, Elsevier, cited by 14 (7.00 per year)
L Ouyang, CB Highley, CB Rodell, W Sun, ... (2016) 3D printing of shear-thinning hyaluronic acid hydrogels with secondary cross-linking. ACS Biomaterials …, ACS Publications, cited by 134 (44.67 per year)
UA Gurkan, R El Assal, SE Yildiz, Y Sung, ... (2014) Engineering anisotropic biomimetic fibrocartilage microenvironment by bioprinting mesenchymal stem cells in nanoliter gel droplets. Molecular …, ACS Publications, cited by 73 (14.60 per year)
S Limmahakhun, A Oloyede, K Sitthiseripratip, ... (2017) 3D-printed cellular structures for bone biomimetic implants. Additive …, Elsevier, cited by 16 (8.00 per year)
M López, R Rubio, S Martín, B Croxford (2017) How plants inspire façades. From plants to architecture: Biomimetic principles for the development of adaptive architectural envelopes. Renewable and Sustainable …, Elsevier, cited by 33 (16.50 per year)
S Ji, M Guvendiren (2017) Recent advances in bioink design for 3D bioprinting of tissues and organs. Frontiers in bioengineering and biotechnology, frontiersin.org, cited by 82 (41.00 per year)
MA Nowicki, NJ Castro, MW Plesniak, ... (2016) 3D printing of novel osteochondral scaffolds with graded microstructure. Nanotechnology, iopscience.iop.org, cited by 24 (8.00 per year)
CZ Liu, ZD Xia, ZW Han, PA Hulley, ... (2008) Novel 3D collagen scaffolds fabricated by indirect printing technique for tissue engineering. … Research Part B …, Wiley Online Library, cited by 88 (8.00 per year)
W Zhu, J Li, YJ Leong, I Rozen, X Qu, ... (2015) 3D-printed artificial microfish. Advanced …, Wiley Online Library, cited by 123 (30.75 per year)
J Huling, IK Ko, A Atala, JJ Yoo (2016) Fabrication of biomimetic vascular scaffolds for 3D tissue constructs using vascular corrosion casts. Acta biomaterialia, Elsevier, cited by 22 (7.33 per year)
NJ Castro, R Patel, LG Zhang (2015) Design of a novel 3D printed bioactive nanocomposite scaffold for improved osteochondral regeneration. Cellular and molecular bioengineering, Springer, cited by 37 (9.25 per year)
SJ Lee, M Nowicki, B Harris, LG Zhang (2017) Fabrication of a Highly Aligned Neural Scaffold via a Table Top Stereolithography 3D Printing and Electrospinning. Tissue Engineering Part A, liebertpub.com, cited by 30 (15.00 per year)
AA Egorov, AY Fedotov, AV Mironov, ... (2016) 3D printing of mineral–polymer bone substitutes based on sodium alginate and calcium phosphate. Beilstein journal of …, beilstein-journals.org, cited by 12 (4.00 per year)
BN Johnson, KZ Lancaster, IB Hogue, F Meng, ... (2016) 3D printed nervous system on a chip. Lab on a Chip, pubs.rsc.org, cited by 63 (21.00 per year)

P Soman, PH Chung, AP Zhang, ... (2013) Digital microfabrication of user-defined 3D microstructures in cell-laden hydrogels. Biotechnology and …, Wiley Online Library, cited by 102 (17.00 per year)
S Sant, MJ Hancock, JP Donnelly, D Iyer, ... (2010) Biomimetic gradient hydrogels for tissue engineering. … Canadian journal of …, Wiley Online Library, cited by 197 (21.89 per year)
NE Fedorovich, J Alblas, JR de Wijn, ... (2007) Hydrogels as extracellular matrices for skeletal tissue engineering: state-of-the-art and novel application in organ printing. Tissue …, liebertpub.com, cited by 386 (32.17 per year)
J Palmisano, R Ramamurti, KJ Lu, ... (2007) Design of a biomimetic controlled-curvature robotic pectoral fin. … on Robotics and …, ieeexplore.ieee.org, cited by 62 (5.17 per year)
V Schmidt, M Belegratis (2016) Laser technology in biomimetics., Springer, cited by 13 (4.33 per year)
CZ Liu, E Sachlos, DA Wahl, ZW Han, ... (2007) On the manufacturability of scaffold mould using a 3D printing technology. Rapid Prototyping …, emeraldinsight.com, cited by 24 (2.00 per year)
J Rossiter, P Walters, ... (2009) Printing 3D dielectric elastomer actuators for soft robotics. … Polymer Actuators and …, spiedigitallibrary.org, cited by 99 (9.90 per year)
A Arslan-Yildiz, R El Assal, P Chen, S Guven, ... (2016) Towards artificial tissue models: past, present, and future of 3D bioprinting. …, iopscience.iop.org, cited by 92 (30.67 per year)
MM Porter, N Ravikumar, F Barthelat, ... (2017) 3D-printing and mechanics of bio-inspired articulated and multi-material structures. Journal of the mechanical …, Elsevier, cited by 15 (7.50 per year)
B Duan (2017) State-of-the-art review of 3D bioprinting for cardiovascular tissue engineering. Annals of biomedical engineering, Springer, cited by 98 (49.00 per year)
SJ Lee, W Zhu, M Nowicki, G Lee, ... (2018) 3D printing nano conductive multi-walled carbon nanotube scaffolds for nerve regeneration. Journal of neural …, iopscience.iop.org, cited by 21 (21.00 per year)
WJ Kim, M Kim, GH Kim (2018) 3D-printed biomimetic scaffold simulating microfibril muscle structure. Advanced Functional Materials, Wiley Online Library, cited by 13 (13.00 per year)
W Zhu, B Holmes, RI Glazer, LG Zhang (2016) 3D printed nanocomposite matrix for the study of breast cancer bone metastasis. … : Nanotechnology, Biology and …, Elsevier, cited by 60 (20.00 per year)
L Ionov (2013) Biomimetic hydrogel-based actuating systems. Advanced Functional Materials, Wiley Online Library, cited by 242 (40.33 per year)
B Bhushan, M Caspers (2017) An overview of additive manufacturing (3D printing) for microfabrication. Microsystem Technologies, Springer, cited by 59 (29.50 per year)
J Wang, M Yang, Y Zhu, L Wang, AP Tomsia, ... (2014) Phage nanofibers induce vascularized osteogenesis in 3D printed bone scaffolds. Advanced …, Wiley Online Library, cited by 126 (25.20 per year)
X Zhou, NJ Castro, W Zhu, H Cui, M Aliabouzar, ... (2016) Improved human bone marrow mesenchymal stem cell osteogenesis in 3D bioprinted tissue scaffolds with low intensity pulsed ultrasound stimulation. Scientific reports, nature.com, cited by 43 (14.33 per year)
X Li, J He, W Zhang, N Jiang, D Li (2016) Additive manufacturing of biomedical constructs with biomimetic structural organizations. Materials, mdpi.com, cited by 11 (3.67 per year)
E Sachlos, D Gotora, JT Czernuszka (2006) Collagen scaffolds reinforced with biomimetic composite nano-sized carbonate-substituted hydroxyapatite crystals and shaped by rapid

prototyping to contain internal …. Tissue engineering, liebertpub.com, cited by 164 (12.62 per year)
H Yang, WR Leow, X Chen (2018) 3D printing of flexible electronic devices. Small Methods, Wiley Online Library, cited by 17 (17.00 per year)
J Wu, L Xie, WZY Lin, Q Chen (2017) Biomimetic nanofibrous scaffolds for neural tissue engineering and drug development. Drug discovery today, Elsevier, cited by 14 (7.00 per year)
L Wen, JC Weaver, PJM Thornycroft, ... (2015) Hydrodynamic function of biomimetic shark skin: effect of denticle pattern and spacing. … & biomimetics, iopscience.iop.org, cited by 41 (10.25 per year)
S Song, M Sitti (2014) Soft grippers using micro-fibrillar adhesives for transfer printing. Advanced Materials, Wiley Online Library, cited by 58 (11.60 per year)
S Hong, D Sycks, HF Chan, S Lin, GP Lopez, ... (2015) 3D printing of highly stretchable and tough hydrogels into complex, cellularized structures. Advanced …, Wiley Online Library, cited by 324 (81.00 per year)
AA Al-Munajjed, NA Plunkett, ... (2009) Development of a biomimetic collagen-hydroxyapatite scaffold for bone tissue engineering using a SBF immersion technique. … Research Part B …, Wiley Online Library, cited by 145 (14.50 per year)
NJ Castro, C Meinert, P Levett, ... (2017) Current developments in multifunctional smart materials for 3D/4D bioprinting. Current Opinion in …, Elsevier, cited by 14 (7.00 per year)
Y Zhao, Y Li, S Mao, W Sun, R Yao (2015) The influence of printing parameters on cell survival rate and printability in microextrusion-based 3D cell printing technology. Biofabrication, iopscience.iop.org, cited by 79 (19.75 per year)
L Huang, R Jiang, J Wu, J Song, H Bai, B Li, ... (2017) Ultrafast digital printing toward 4D shape changing materials. Advanced …, Wiley Online Library, cited by 133 (66.50 per year)
R Wimmer, B Steyrer, J Woess, T Koddenberg, ... (2015) 3D printing and wood. Pro Ligno, researchgate.net, cited by 11 (2.75 per year)
J Wang, P Ghassemi, A Melchiorri, ... (2015) 3D printed biomimetic vascular phantoms for assessment of hyperspectral imaging systems. … of Phantoms Used …, spiedigitallibrary.org, cited by 10 (2.50 per year)
JT Muth, DM Vogt, RL Truby, Y Mengüç, ... (2014) Embedded 3D printing of strain sensors within highly stretchable elastomers. Advanced …, Wiley Online Library, cited by 677 (135.40 per year)
X Wang, H Schröder, Q Feng, F Draenert, W Müller (2013) The deep-sea natural products, biogenic polyphosphate (Bio-PolyP) and biogenic silica (Bio-Silica), as biomimetic scaffolds for bone tissue engineering: fabrication of …. Marine drugs, mdpi.com, cited by 30 (5.00 per year)
H Ko, H Yi, HE Jeong (2017) Wall and ceiling climbing quadruped robot with superior water repellency manufactured using 3D printing (UNIclimb). International Journal of Precision Engineering and …, Springer, cited by 15 (7.50 per year)
U Ghosh, S Ning, Y Wang, ... (2018) Addressing unmet clinical needs with 3D printing technologies. Advanced healthcare …, Wiley Online Library, cited by 13 (13.00 per year)
GX Gu, I Su, S Sharma, ... (2016) Three-dimensional-printing of bio-inspired composites. Journal of …, … .asmedigitalcollection.asme.org, cited by 67 (22.33 per year)
CG Jeong, A Atala (2015) 3D printing and biofabrication for load bearing tissue engineering. Engineering Mineralized and Load Bearing Tissues, Springer, cited by 21 (5.25 per year)

R Nigam, B Mahanta (2014) An overview of various biomimetic scaffolds: Challenges and applications in tissue engineering. Journal of Tissue Science & …, pdfs.semanticscholar.org, cited by 19 (3.80 per year)
Z Hu, K Thiyagarajan, A Bhusal, T Letcher, ... (2017) Design of ultra-lightweight and high-strength cellular structural composites inspired by biomimetics. Composites Part B …, Elsevier, cited by 14 (7.00 per year)
S Kyle, ZM Jessop, A Al-Sabah, ... (2017) 'Printability'of Candidate Biomaterials for Extrusion Based 3D Printing: State-of-the-Art. Advanced healthcare …, Wiley Online Library, cited by 49 (24.50 per year)
J Rossiter, S Sareh (2014) Kirigami design and fabrication for biomimetic robotics. … , Biomimetics, and Bioreplication 2014, spiedigitallibrary.org, cited by 12 (2.40 per year)
CM O'Brien, B Holmes, S Faucett, ... (2014) Three-dimensional printing of nanomaterial scaffolds for complex tissue regeneration. Tissue Engineering Part …, liebertpub.com, cited by 94 (18.80 per year)
DX Liu, YL Sun, WF Dong, RZ Yang, ... (2014) Dynamic laser prototyping for biomimetic nanofabrication. Laser & Photonics …, Wiley Online Library, cited by 18 (3.60 per year)
P Ghassemi, J Wang, AJ Melchiorri, ... (2015) Rapid prototyping of biomimetic vascular phantoms for hyperspectral reflectance imaging. … of biomedical optics, spiedigitallibrary.org, cited by 21 (5.25 per year)
C Xu, W Chai, Y Huang, ... (2012) Scaffold-free inkjet printing of three-dimensional zigzag cellular tubes. Biotechnology and …, Wiley Online Library, cited by 190 (27.14 per year)
BN Johnson, KZ Lancaster, G Zhen, ... (2015) 3D printed anatomical nerve regeneration pathways. Advanced functional …, Wiley Online Library, cited by 107 (26.75 per year)
J Koffler, W Zhu, X Qu, O Platoshyn, JN Dulin, J Brock, ... (2019) Biomimetic 3D-printed scaffolds for spinal cord injury repair. Nature medicine, nature.com, cited by 9 (9.00 per year)
E Volkmer, I Drosse, S Otto, A Stangelmayer, ... (2008) Hypoxia in static and dynamic 3D culture systems for tissue engineering of bone. … Engineering Part A, liebertpub.com, cited by 268 (24.36 per year)
MM Porter, N Ravikumar (2017) 3D-printing a 'family'of biomimetic models to explain armored grasping in syngnathid fishes. Bioinspiration & biomimetics, iopscience.iop.org, cited by 5 (2.50 per year)
SH Pyo, P Wang, HH Hwang, W Zhu, ... (2016) Continuous optical 3D printing of green aliphatic polyurethanes. … applied materials & …, ACS Publications, cited by 13 (4.33 per year)
CS Chen (2016) 3D biomimetic cultures: the next platform for cell biology. Trends in cell biology, Elsevier, cited by 13 (4.33 per year)
S Knowlton, CH Yu, F Ersoy, S Emadi, ... (2016) 3D-printed microfluidic chips with patterned, cell-laden hydrogel constructs. …, iopscience.iop.org, cited by 46 (15.33 per year)
C Colasante, Z Sanford, E Garfein, O Tepper (2016) Current trends in 3D printing, bioprosthetics, and tissue engineering in plastic and reconstructive surgery. Current Surgery Reports, Springer, cited by 13 (4.33 per year)
M Rumpler, A Woesz, F Varga, ... (2007) Three-dimensional growth behavior of osteoblasts on biomimetic hydroxylapatite scaffolds. … Research Part A, Wiley Online Library, cited by 43 (3.58 per year)
RL Truby, M Wehner, AK Grosskopf, ... (2018) Soft somatosensitive actuators via embedded 3D printing. Advanced …, Wiley Online Library, cited by 53 (53.00 per year)

KJ Tsai, S Dixon, LR Hale, A Darbyshire, ... (2017) Biomimetic heterogenous elastic tissue development. NPJ Regenerative …, nature.com, cited by 10 (5.00 per year)
M Lee, JCY Dunn, BM Wu (2005) Scaffold fabrication by indirect three-dimensional printing. Biomaterials, Elsevier, cited by 253 (18.07 per year)
M Röhrig, M Thiel, M Worgull, H Hölscher (2012) 3D direct laser writing of nano-and microstructured hierarchical gecko-mimicking surfaces. Small, Wiley Online Library, cited by 101 (14.43 per year)
HD Kim, S Amirthalingam, SL Kim, ... (2017) Biomimetic materials and fabrication approaches for bone tissue engineering. Advanced …, Wiley Online Library, cited by 26 (13.00 per year)
J Zhang, Y Yao, L Sheng, J Liu (2015) Self-fueled biomimetic liquid metal mollusk. Advanced Materials, Wiley Online Library, cited by 176 (44.00 per year)
H Cui, W Zhu, B Holmes, LG Zhang (2016) Biologically inspired smart release system based on 3D bioprinted perfused scaffold for vascularized tissue regeneration. Advanced science, Wiley Online Library, cited by 42 (14.00 per year)
A Fallahi, A Khademhosseini, A Tamayol (2016) Textile processes for engineering tissues with biomimetic architectures and properties. Trends in biotechnology, Elsevier, cited by 10 (3.33 per year)
G Siqueira, D Kokkinis, R Libanori, ... (2017) Cellulose nanocrystal inks for 3D printing of textured cellular architectures. Advanced Functional …, Wiley Online Library, cited by 128 (64.00 per year)
YS Zhang, K Yue, J Aleman, ... (2017) 3D bioprinting for tissue and organ fabrication. Annals of biomedical …, Springer, cited by 155 (77.50 per year)
W Tan, TA Desai (2005) Microscale multilayer cocultures for biomimetic blood vessels. … of Biomedical Materials Research Part A: An …, Wiley Online Library, cited by 114 (8.14 per year)
V Mironov, V Kasyanov, C Drake, RR Markwald (2008) Organ printing: promises and challenges., Future Medicine, cited by 249 (22.64 per year)
W Zhu, NJ Castro, H Cui, X Zhou, B Boualam, ... (2016) A 3D printed nano bone matrix for characterization of breast cancer cell and osteoblast interactions. …, iopscience.iop.org, cited by 19 (6.33 per year)
W Zhang, Q Lian, D Li, K Wang, D Hao, ... (2014) Cartilage repair and subchondral bone migration using 3D printing osteochondral composites: a one-year-period study in rabbit trochlea. BioMed research …, hindawi.com, cited by 61 (12.20 per year)
ME Staymates, WA MacCrehan, JL Staymates, ... (2016) Biomimetic sniffing improves the detection performance of a 3D printed nose of a dog and a commercial trace vapor detector. Scientific reports, nature.com, cited by 19 (6.33 per year)
X Bao, L Zhu, X Huang, D Tang, D He, J Shi, G Xu (2017) 3D biomimetic artificial bone scaffolds with dual-cytokines spatiotemporal delivery for large weight-bearing bone defect repair. Scientific reports, nature.com, cited by 11 (5.50 per year)
NE Fedorovich, JR De Wijn, AJ Verbout, ... (2008) Three-dimensional fiber deposition of cell-laden, viable, patterned constructs for bone tissue printing. … Engineering Part A, liebertpub.com, cited by 294 (26.73 per year)
YW Moon, IJ Choi, YH Koh, HE Kim (2015) Porous alumina ceramic scaffolds with biomimetic macro/micro-porous structure using three-dimensional (3-D) ceramic/camphene-based extrusion. Ceramics International, Elsevier, cited by 18 (4.50 per year)

J Delamare, R Sanders, G Krijnen (2016) 3D printed biomimetic whisker-based sensor with co-planar capacitive sensing. 2016 IEEE SENSORS, ieeexplore.ieee.org, cited by 7 (2.33 per year)
S Frølich, JC Weaver, MN Dean, ... (2017) Uncovering Nature's Design Strategies through Parametric Modeling, Multi-Material 3D Printing, and Mechanical Testing. Advanced Engineering …, Wiley Online Library, cited by 8 (4.00 per year)
Y He, J Qiu, J Fu, J Zhang, Y Ren, A Liu (2015) Printing 3D microfluidic chips with a 3D sugar printer. Microfluidics and Nanofluidics, Springer, cited by 49 (12.25 per year)
JEM Teoh, J An, CK Chua, M Lv, ... (2017) Hierarchically self-morphing structure through 4D printing. Virtual and Physical …, Taylor & Francis, cited by 26 (13.00 per year)
E Pei (2014) 4D printing–revolution or fad?. Assembly Automation, emeraldinsight.com, cited by 41 (8.20 per year)
R Ghosh, H Ebrahimi, A Vaziri (2014) Contact kinematics of biomimetic scales. Applied Physics Letters, aip.scitation.org, cited by 23 (4.60 per year)
K Christensen, C Xu, W Chai, Z Zhang, ... (2015) Freeform inkjet printing of cellular structures with bifurcations. Biotechnology and …, Wiley Online Library, cited by 122 (30.50 per year)
TG Kim, H Shin, DW Lim (2012) Biomimetic scaffolds for tissue engineering. Advanced Functional Materials, Wiley Online Library, cited by 264 (37.71 per year)
NA Meisel, AM Elliott, ... (2015) A procedure for creating actuated joints via embedding shape memory alloys in PolyJet 3D printing. Journal of intelligent …, journals.sagepub.com, cited by 45 (11.25 per year)
X Zhai, Y Ma, C Hou, F Gao, Y Zhang, ... (2017) 3D-printed high strength bioactive supramolecular polymer/clay nanocomposite hydrogel scaffold for bone regeneration. ACS Biomaterials …, ACS Publications, cited by 39 (19.50 per year)
R Aversa, RV Petrescu, FI Petrescu, ... (2016) Biomimetic and evolutionary design driven innovation in sustainable products development. American Journal of …, papers.ssrn.com, cited by 59 (19.67 per year)
MA Bohl, MA Mooney, GJ Repp, P Nakaji, SW Chang, ... (2018) The Barrow Biomimetic Spine: fluoroscopic analysis of a synthetic spine model made of variable 3D-printed materials and print parameters. Spine, journals.lww.com, cited by 7 (7.00 per year)
MO Wang, CE Vorwald, ML Dreher, EJ Mott, ... (2015) Evaluating 3D-Printed biomaterials as scaffolds for vascularized bone tissue engineering. Advanced …, Wiley Online Library, cited by 127 (31.75 per year)
KC Hung, CS Tseng, S Hsu (2014) Synthesis and 3D printing of biodegradable polyurethane elastomer by a water-based process for cartilage tissue engineering applications. Advanced healthcare materials, Wiley Online Library, cited by 78 (15.60 per year)
RC Thomas, P Vu, SP Modi, PE Chung, ... (2017) Sacrificial crystal templated hyaluronic acid hydrogels as biomimetic 3D tissue scaffolds for nerve tissue regeneration. ACS Biomaterials …, ACS Publications, cited by 10 (5.00 per year)
MS Chen, Y Zhang, L Zhang (2017) Fabrication and characterization of a 3D bioprinted nanoparticle-hydrogel hybrid device for biomimetic detoxification. Nanoscale, pubs.rsc.org, cited by 7 (3.50 per year)
JS Mulford, S Babazadeh, N Mackay (2016) Three-dimensional printing in orthopaedic surgery: review of current and future applications. ANZ journal of surgery, Wiley Online Library, cited by 42 (14.00 per year)
SS Liao, FZ Cui, W Zhang, ... (2004) Hierarchically biomimetic bone scaffold materials: nano-HA/collagen/PLA composite. Journal of Biomedical …, Wiley Online Library, cited by 487 (32.47 per year)

Y Luo, Y Li, X Qin, Q Wa (2018) 3D printing of concentrated alginate/gelatin scaffolds with homogeneous nano apatite coating for bone tissue engineering. Materials & Design, Elsevier, cited by 15 (15.00 per year)
B Duan, LA Hockaday, KH Kang, ... (2013) 3D bioprinting of heterogeneous aortic valve conduits with alginate/gelatin hydrogels. Journal of biomedical …, Wiley Online Library, cited by 484 (80.67 per year)
X Kuang, DJ Roach, J Wu, CM Hamel, ... (2019) Advances in 4D printing: Materials and applications. Advanced Functional …, Wiley Online Library, cited by 23 (23.00 per year)
SL Dong, L Han, CX Du, XY Wang, ... (2017) 3D printing of aniline tetramer-grafted-polyethylenimine and pluronic F127 composites for electroactive scaffolds. Macromolecular rapid …, Wiley Online Library, cited by 12 (6.00 per year)
LJ Stiltner, AM Elliott, ... (2011) A method for creating actuated joints via fiber embedding in a polyjet 3D printing process. International Solid …, sffsymposium.engr.utexas.edu, cited by 20 (2.50 per year)
Y Xu, Y Hu, C Liu, H Yao, B Liu, S Mi (2018) A Novel Strategy for Creating Tissue-Engineered Biomimetic Blood Vessels Using 3D Bioprinting Technology. Materials, mdpi.com, cited by 7 (7.00 per year)
T Link, X Wang, U Schloßmacher, Q Feng, ... (2013) An approach to a biomimetic bone scaffold: increased expression of BMP-2 and of osteoprotegerin in SaOS-2 cells grown onto silica-biologized 3D printed …. RSC Advances, pubs.rsc.org, cited by 8 (1.33 per year)
F Pati, J Gantelius, HA Svahn (2016) 3D bioprinting of tissue/organ models. … Chemie International Edition, Wiley Online Library, cited by 87 (29.00 per year)
R Bogue (2008) Biomimetic adhesives: a review of recent developments. Assembly Automation, emeraldinsight.com, cited by 8 (0.73 per year)
AD Valentine, TA Busbee, JW Boley, ... (2017) Hybrid 3D printing of soft electronics. advanced …, Wiley Online Library, cited by 96 (48.00 per year)
A Paul (2015) Nanocomposite hydrogels: an emerging biomimetic platform for myocardial therapy and tissue engineering. Nanomedicine, Future Medicine, cited by 30 (7.50 per year)
GH Wu, S Hsu (2015) polymeric-based 3D printing for tissue engineering. Journal of medical and biological engineering, Springer, cited by 74 (18.50 per year)
D Wu, JN Wang, SZ Wu, QD Chen, ... (2011) Three-level biomimetic rice-leaf surfaces with controllable anisotropic sliding. Advanced Functional …, Wiley Online Library, cited by 166 (20.75 per year)
JE Kim, SH Kim, Y Jung (2016) Current status of three-dimensional printing inks for soft tissue regeneration. Tissue engineering and regenerative medicine, Springer, cited by 22 (7.33 per year)
R Martini, Y Balit, F Barthelat (2017) A comparative study of bio-inspired protective scales using 3D printing and mechanical testing. Acta biomaterialia, Elsevier, cited by 19 (9.50 per year)
A Accardo, MC Blatché, R Courson, I Loubinoux, ... (2017) Multiphoton direct laser writing and 3D imaging of polymeric freestanding architectures for cell colonization. Small, Wiley Online Library, cited by 28 (14.00 per year)
V Kapsali, A Toomey, R Oliver, L Tandler (2013) Biomimetic spatial and temporal (4D) design and fabrication. Conference on Biomimetic and …, Springer, cited by 5 (0.83 per year)
SZ Guo, K Qiu, F Meng, SH Park, ... (2017) 3D printed stretchable tactile sensors. Advanced …, Wiley Online Library, cited by 80 (40.00 per year)

D He, C Zhuang, S Xu, X Ke, X Yang, L Zhang, ... (2016) 3D printing of Mg-substituted wollastonite reinforcing diopside porous bioceramics with enhanced mechanical and biological performances. Bioactive materials, Elsevier, cited by 13 (4.33 per year)
L Koch, A Deiwick, S Schlie, S Michael, ... (2012) Skin tissue generation by laser cell printing. Biotechnology and …, Wiley Online Library, cited by 287 (41.00 per year)
J Duro-Royo, K Zolotovsky, L Mogas-Soldevila, ... (2015) MetaMesh: A hierarchical computational model for design and fabrication of biomimetic armored surfaces. Computer-Aided …, Elsevier, cited by 31 (7.75 per year)
JL Simon, S Michna, JA Lewis, ... (2007) In vivo bone response to 3D periodic hydroxyapatite scaffolds assembled by direct ink writing. … Research Part A: An …, Wiley Online Library, cited by 142 (11.83 per year)
WL Ng, JTZ Qi, WY Yeong, MW Naing (2018) Proof-of-concept: 3D bioprinting of pigmented human skin constructs. Biofabrication, iopscience.iop.org, cited by 31 (31.00 per year)
M Lee, BM Wu (2012) Recent advances in 3D printing of tissue engineering scaffolds. Computer-Aided Tissue Engineering, Springer, cited by 60 (8.57 per year)
A Gupta, AM Seifalian, Z Ahmad, ... (2007) Novel electrohydrodynamic printing of nanocomposite biopolymer scaffolds. Journal of bioactive …, journals.sagepub.com, cited by 75 (6.25 per year)
CC Spackman, CR Frank, KC Picha, ... (2016) 3D printing of fiber-reinforced soft composites: Process study and material characterization. Journal of Manufacturing …, Elsevier, cited by 53 (17.67 per year)
JS Naftulin, EY Kimchi, SS Cash (2015) Streamlined, inexpensive 3D printing of the brain and skull. PloS one, journals.plos.org, cited by 71 (17.75 per year)
FA Maulvi, MJ Shah, BS Solanki, ... (2017) Application of 3D printing technology in the development of novel drug delivery systems. Int J Drug Dev …, pdfs.semanticscholar.org, cited by 15 (7.50 per year)
TJ Hinton, Q Jallerat, RN Palchesko, ... (2015) Three-dimensional printing of complex biological structures by freeform reversible embedding of suspended hydrogels. Science …, advances.sciencemag.org, cited by 370 (92.50 per year)
VK Lee, G Dai (2017) Printing of three-dimensional tissue analogs for regenerative medicine. Annals of biomedical engineering, Springer, cited by 40 (20.00 per year)
AK Capulli, MY Emmert, FS Pasqualini, D Kehl, ... (2017) JetValve: rapid manufacturing of biohybrid scaffolds for biomimetic heart valve replacement. Biomaterials, Elsevier, cited by 25 (12.50 per year)
DJ Richards, Y Tan, J Jia, H Yao, ... (2013) 3D printing for tissue engineering. Israel journal of …, Wiley Online Library, cited by 50 (8.33 per year)
T Bartolini, V Mwaffo, A Showler, S Macrì, ... (2016) Zebrafish response to 3D printed shoals of conspecifics: the effect of body size. … & biomimetics, iopscience.iop.org, cited by 29 (9.67 per year)
C Richter, H Lipson (2011) Untethered hovering flapping flight of a 3D-printed mechanical insect. Artificial life, MIT Press, cited by 85 (10.63 per year)
K Schacht, T Jüngst, M Schweinlin, ... (2015) Biofabrication of cell-loaded 3D spider silk constructs. Angewandte Chemie …, Wiley Online Library, cited by 104 (26.00 per year)
L Koch, S Kuhn, H Sorg, M Gruene, ... (2009) Laser printing of skin cells and human stem cells. … Engineering Part C …, liebertpub.com, cited by 246 (24.60 per year)
X Liu, H Gu, M Wang, X Du, B Gao, A Elbaz, ... (2018) 3D printing of bioinspired liquid superrepellent structures. Advanced …, Wiley Online Library, cited by 19 (19.00 per year)

S Bradshaw, A Bowyer, P Haufe (2010) The intellectual property implications of low-cost 3D printing. ScriptEd, HeinOnline, cited by 154 (17.11 per year)
SV Murphy, A Skardal, A Atala (2013) Evaluation of hydrogels for bio-printing applications. Journal of Biomedical …, Wiley Online Library, cited by 284 (47.33 per year)
C Yu, C Li, C Gao, Z Dong, L Wu, L Jiang (2018) Time-dependent liquid transport on a biomimetic topological surface. ACS nano, ACS Publications, cited by 12 (12.00 per year)
LE Murr, LE Murr (2015) Biomimetics and biologically inspired materials. … of materials structures, properties, processing and …, Springer, cited by 11 (2.75 per year)
Y Li, KA Kilian (2015) Bridging the gap: from 2D cell culture to 3D microengineered extracellular matrices. Advanced healthcare materials, Wiley Online Library, cited by 36 (9.00 per year)
NJ Castro, CM O'brien, LG Zhang (2016) Biomimetic biphasic 3d nanocomposite scaffold for osteochondral regeneration. US Patent App. 14/854,504, Google Patents, cited by 7 (2.33 per year)
H Yang, WR Leow, T Wang, J Wang, J Yu, ... (2017) 3D printed photoresponsive devices based on shape memory composites. Advanced …, Wiley Online Library, cited by 72 (36.00 per year)
A Roy, V Saxena, LM Pandey (2018) 3D printing for cardiovascular tissue engineering: a review. Materials technology, Taylor & Francis, cited by 5 (5.00 per year)
JS Lee, YJ Seol, M Sung, W Moon, SW Kim, ... (2016) Development and analysis of three-dimensional (3D) printed biomimetic ceramic. International Journal of …, Springer, cited by 5 (1.67 per year)
E Vorndran, M Klarner, U Klammert, ... (2008) 3D powder printing of β-tricalcium phosphate ceramics using different strategies. Advanced …, Wiley Online Library, cited by 113 (10.27 per year)
DB Kolesky, RL Truby, AS Gladman, ... (2014) 3D bioprinting of vascularized, heterogeneous cell-laden tissue constructs. Advanced …, Wiley Online Library, cited by 948 (189.60 per year)
Y Yang, X Song, X Li, Z Chen, C Zhou, ... (2018) Recent progress in biomimetic additive manufacturing technology: from materials to functional structures. Advanced …, Wiley Online Library, cited by 38 (38.00 per year)
R Kankala, XM Xu, CG Liu, AZ Chen, SB Wang (2018) 3D-printing of microfibrous porous scaffolds based on hybrid approaches for bone tissue engineering. Polymers, mdpi.com, cited by 14 (14.00 per year)
Z Tan, C Parisi, L Di Silvio, D Dini, AE Forte (2017) Cryogenic 3D printing of super soft hydrogels. Scientific reports, nature.com, cited by 19 (9.50 per year)
C Colosi, SR Shin, V Manoharan, S Massa, ... (2016) Microfluidic bioprinting of heterogeneous 3D tissue constructs using low-viscosity bioink. Advanced …, Wiley Online Library, cited by 241 (80.33 per year)
N Oxman, M Kayser, J Laucks, ... (2013) Robotically controlled fiber-based manufacturing as case study for biomimetic digital fabrication. Green Design, Materials …, books.google.com, cited by 8 (1.33 per year)
Y Zuo, X Liu, D Wei, J Sun, W Xiao, ... (2015) Photo-cross-linkable methacrylated gelatin and hydroxyapatite hybrid hydrogel for modularly engineering biomimetic osteon. … applied materials & …, ACS Publications, cited by 46 (11.50 per year)
B Feigl, D Hutmacher (2013) Eyes on 3D-Current 3D Biomimetic Disease Concept Models and Potential Applications in Age-Related Macular Degeneration. Advanced healthcare materials, Wiley Online Library, cited by 8 (1.33 per year)

W Jia, PS Gungor-Ozkerim, YS Zhang, K Yue, K Zhu, ... (2016) Direct 3D bioprinting of perfusable vascular constructs using a blend bioink. Biomaterials, Elsevier, cited by 230 (76.67 per year)
F Zhu, L Cheng, ZJ Wang, W Hong, ZL Wu, ... (2017) 3D-printed ultratough hydrogel structures with titin-like domains. … applied materials & …, ACS Publications, cited by 18 (9.00 per year)
HJ Jeong, MH Jee, SY Kim, SJ Lee (2014) Measurement of the compressive force on the knee joint model fabricated by 3D printing. Journal of the Korean …, koreascience.or.kr, cited by 7 (1.40 per year)
Q Gu, J Hao, Y Lu, L Wang, GG Wallace, ... (2015) Three-dimensional bio-printing. Science China Life …, Springer, cited by 41 (10.25 per year)
R Kankala, FJ Lu, CG Liu, SS Zhang, AZ Chen, ... (2018) Effect of icariin on engineered 3d-printed porous scaffolds for cartilage repair. Materials, mdpi.com, cited by 9 (9.00 per year)
Y Lu, S Yi, Y Liu, Y Ji (2016) A novel path planning method for biomimetic robot based on deep learning. Assembly automation, emeraldinsight.com, cited by 9 (3.00 per year)
DHT Nguyen, SC Stapleton, MT Yang, ... (2013) Biomimetic model to reconstitute angiogenic sprouting morphogenesis in vitro. Proceedings of the …, National Acad Sciences, cited by 217 (36.17 per year)
T Boland, V Mironov, A Gutowska, ... (2003) Cell and organ printing 2: Fusion of cell aggregates in three-dimensional gels. … Record Part A …, Wiley Online Library, cited by 338 (21.13 per year)
MA Darabi, A Khosrozadeh, R Mbeleck, ... (2017) Skin-inspired multifunctional autonomic-intrinsic conductive self-healing hydrogels with pressure sensitivity, stretchability, and 3D printability. Advanced …, Wiley Online Library, cited by 116 (58.00 per year)
S Knaack, A Lode, B Hoyer, ... (2014) Heparin modification of a biomimetic bone matrix for controlled release of VEGF. … Research Part A, Wiley Online Library, cited by 34 (6.80 per year)
T Umedachi, V Vikas, BA Trimmer (2016) Softworms: the design and control of non-pneumatic, 3D-printed, deformable robots. Bioinspiration & biomimetics, iopscience.iop.org, cited by 76 (25.33 per year)
DN Heo, NJ Castro, SJ Lee, H Noh, W Zhu, LG Zhang (2017) Enhanced bone tissue regeneration using a 3D printed microstructure incorporated with a hybrid nano hydrogel. Nanoscale, pubs.rsc.org, cited by 29 (14.50 per year)
M Gruene, M Pflaum, C Hess, ... (2011) Laser printing of three-dimensional multicellular arrays for studies of cell–cell and cell–environment interactions. … Engineering Part C …, liebertpub.com, cited by 126 (15.75 per year)
C Li, C Yu, D Hao, L Wu, Z Dong, ... (2018) Smart Liquid Transport on Dual Biomimetic Surface via Temperature Fluctuation Control. Advanced Functional …, Wiley Online Library, cited by 6 (6.00 per year)
S Liang, W Rao, K Song, J Liu (2018) Fluorescent Liquid Metal As a Transformable Biomimetic Chameleon. ACS applied materials & …, ACS Publications, cited by 12 (12.00 per year)
RA Rezende, F Pereira, V Kasyanov, DT Kemmoku, ... (2013) Scalable biofabrication of tissue spheroids for organ printing. Procedia CIRP, Elsevier, cited by 32 (5.33 per year)
B Holmes, K Bulusu, M Plesniak, LG Zhang (2016) A synergistic approach to the design, fabrication and evaluation of 3D printed micro and nano featured scaffolds for vascularized bone tissue repair. Nanotechnology, iopscience.iop.org, cited by 41 (13.67 per year)

S Miao, W Zhu, NJ Castro, M Nowicki, X Zhou, H Cui, ... (2016) 4D printing smart biomedical scaffolds with novel soybean oil epoxidized acrylate. Scientific reports, nature.com, cited by 69 (23.00 per year)
P Li (2003) Biomimetic nano-apatite coating capable of promoting bone ingrowth. Journal of Biomedical Materials Research Part A: An …, Wiley Online Library, cited by 170 (10.63 per year)
C Chua, W Yeong, J An (2017) 3D printing for biomedical engineering., mdpi.com, cited by 6 (3.00 per year)
C Vyas, R Pereira, B Huang, F Liu, W Wang, ... (2017) Engineering the vasculature with additive manufacturing. Current Opinion in …, Elsevier, cited by 39 (19.50 per year)
L Liu, Y Li (2018) Failure mechanism transition of 3D-printed biomimetic sutures. Engineering Fracture Mechanics, Elsevier, cited by 4 (4.00 per year)
R Chang, J Nam, W Sun (2008) Effects of dispensing pressure and nozzle diameter on cell survival from solid freeform fabrication–based direct cell writing. Tissue Engineering Part A, liebertpub.com, cited by 296 (26.91 per year)
A Derossi, R Caporizzi, D Azzollini, ... (2018) Application of 3D printing for customized food. A case on the development of a fruit-based snack for children. Journal of Food …, Elsevier, cited by 38 (38.00 per year)
FPW Melchels, MM Blokzijl, R Levato, QC Peiffer, ... (2016) Hydrogel-based reinforcement of 3D bioprinted constructs. …, iopscience.iop.org, cited by 35 (11.67 per year)
HMCM Anver, R Mutlu, G Alici (2017) 3D printing of a thin-wall soft and monolithic gripper using fused filament fabrication. 2017 IEEE International …, ieeexplore.ieee.org, cited by 7 (3.50 per year)
SM Barinov, IV Vakhrushev, VS Komlev, ... (2015) 3D printing of ceramic scaffolds for engineering of bone tissue. Inorganic Materials …, Springer, cited by 6 (1.50 per year)
Y Liu, P Ghassemi, A Depkon, MI Iacono, ... (2018) Biomimetic 3D-printed neurovascular phantoms for near-infrared fluorescence imaging. Biomedical Optics …, osapublishing.org, cited by 4 (4.00 per year)
J Kim, A Alspach, K Yamane (2015) 3D printed soft skin for safe human-robot interaction. 2015 IEEE/RSJ International …, ieeexplore.ieee.org, cited by 36 (9.00 per year)
SH Kim, YK Yeon, JM Lee, JR Chao, YJ Lee, ... (2018) Precisely printable and biocompatible silk fibroin bioink for digital light processing 3D printing. Nature …, nature.com, cited by 25 (25.00 per year)
C Mandon, L Blum, C Marquette (2017) 3D–4D printed objects: New bioactive material opportunities. Micromachines, mdpi.com, cited by 7 (3.50 per year)
MA Bohl, MA Mooney, GJ Repp, C Cavallo, P Nakaji, ... (2018) The Barrow Biomimetic Spine: comparative testing of a 3D-printed L4-L5 Schwab grade 2 osteotomy model to a cadaveric model. Cureus, ncbi.nlm.nih.gov, cited by 5 (5.00 per year)
RD Farahani, M Dubé, D Therriault (2016) Three-dimensional printing of multifunctional nanocomposites: manufacturing techniques and applications. Advanced Materials, Wiley Online Library, cited by 174 (58.00 per year)
J Schweiger, F Beuer, M Stimmelmayr, D Edelhoff, ... (2016) Histo-anatomic 3D printing of dental structures. British dental …, nature.com, cited by 9 (3.00 per year)
YHA Wu, YC Chiu, YH Lin, CC Ho, MY Shie, ... (2019) 3D-printed bioactive calcium silicate/poly- ε -caprolactone bioscaffolds modified with biomimetic extracellular matrices for bone regeneration. International journal of …, mdpi.com, cited by 5 (5.00 per year)

X Zhou, W Zhu, M Nowicki, S Miao, H Cui, ... (2016) 3D bioprinting a cell-laden bone matrix for breast cancer metastasis study. … applied materials & …, ACS Publications, cited by 56 (18.67 per year)
AV Do, R Smith, TM Acri, SM Geary, ... (2018) 3D printing technologies for 3D scaffold engineering. Functional 3D Tissue …, Elsevier, cited by 5 (5.00 per year)
Z Shen, J Na, Z Wang (2017) A biomimetic underwater soft robot inspired by cephalopod mollusc. IEEE Robotics and Automation Letters, ieeexplore.ieee.org, cited by 6 (3.00 per year)
R Guo, S Lu, JM Page, AR Merkel, ... (2015) Fabrication of 3D scaffolds with precisely controlled substrate modulus and pore size by templated-fused deposition modeling to direct osteogenic differentiation. Advanced …, Wiley Online Library, cited by 22 (5.50 per year)
H Cui, W Zhu, M Nowicki, X Zhou, ... (2016) Hierarchical fabrication of engineered vascularized bone biphasic constructs via dual 3D bioprinting: integrating regional bioactive factors into architectural design. Advanced …, Wiley Online Library, cited by 41 (13.67 per year)
SH Lee, JJ Moon, JL West (2008) Three-dimensional micropatterning of bioactive hydrogels via two-photon laser scanning photolithography for guided 3D cell migration. Biomaterials, Elsevier, cited by 341 (31.00 per year)
E De Jaeghere, E De Vlieghere, J Van Hoorick, ... (2018) Heterocellular 3D scaffolds as biomimetic to recapitulate the tumor microenvironment of peritoneal metastases in vitro and in vivo. Biomaterials, Elsevier, cited by 5 (5.00 per year)
DJ Lorang, D Tanaka, CM Spadaccini, ... (2011) Photocurable liquid core–fugitive shell printing of optical waveguides. Advanced …, Wiley Online Library, cited by 46 (5.75 per year)
R Gauvin, YC Chen, JW Lee, P Soman, P Zorlutuna, ... (2012) Microfabrication of complex porous tissue engineering scaffolds using 3D projection stereolithography. Biomaterials, Elsevier, cited by 343 (49.00 per year)
S Kumar, BL Wardle, MF Arif (2016) Strength and performance enhancement of bonded joints by spatial tailoring of adhesive compliance via 3D printing. ACS applied materials & interfaces, ACS Publications, cited by 22 (7.33 per year)
Y Yang, Y Chen, Y Li, MZ Chen (2016) 3D printing of variable stiffness hyper-redundant robotic arm. 2016 IEEE International …, ieeexplore.ieee.org, cited by 12 (4.00 per year)
O Tricinci, T Terencio, B Mazzolai, ... (2015) 3D Micropatterned Surface Inspired by Salvinia molesta via Direct Laser Lithography. … applied materials & …, ACS Publications, cited by 42 (10.50 per year)
CS Tiwary, S Kishore, S Sarkar, ... (2015) Morphogenesis and mechanostabilization of complex natural and 3D printed shapes. Science …, advances.sciencemag.org, cited by 17 (4.25 per year)
D Rosenzweig, E Carelli, T Steffen, P Jarzem, ... (2015) 3D-printed ABS and PLA scaffolds for cartilage and nucleus pulposus tissue regeneration. International journal of …, mdpi.com, cited by 125 (31.25 per year)
K Markstedt, A Mantas, I Tournier, ... (2015) 3D bioprinting human chondrocytes with nanocellulose–alginate bioink for cartilage tissue engineering applications. …, ACS Publications, cited by 412 (103.00 per year)
CG Helguero, VM Mustahsan, ... (2017) Biomechanical properties of 3D-printed bone scaffolds are improved by treatment with CRFP. Journal of …, josr-online.biomedcentral.com, cited by 5 (2.50 per year)

F Hahn, S Jensen, S Tanev (2014) Disruptive innovation vs disruptive technology: The disruptive potential of the value propositions of 3D printing technology startups. Technology innovation management …, timreview.ca, cited by 22 (4.40 per year)
SA Park, JH Lee, WD Kim (2009) Development of biomimetic scaffold for tissue engineering. Elastomers and Composites, koreascience.or.kr, cited by 14 (1.40 per year)
KC Wong (2016) 3D-printed patient-specific applications in orthopedics. Orthopedic research and reviews, ncbi.nlm.nih.gov, cited by 20 (6.67 per year)
L Cramphorn, B Ward-Cherrier, ... (2017) Addition of a biomimetic fingerprint on an artificial fingertip enhances tactile spatial acuity. IEEE Robotics and …, ieeexplore.ieee.org, cited by 14 (7.00 per year)
JH Park, J Jang, JS Lee, DW Cho (2017) Three-dimensional printing of tissue/organ analogues containing living cells. Annals of biomedical engineering, Springer, cited by 28 (14.00 per year)
M Singh, Y Tong, K Webster, E Cesewski, AP Haring, ... (2017) 3D printed conformal microfluidics for isolation and profiling of biomarkers from whole organs. Lab on a Chip, pubs.rsc.org, cited by 23 (11.50 per year)
M Deng, SG Kumbar, LS Nair, AL Weikel, ... (2011) Biomimetic Structures: Biological Implications of Dipeptide-Substituted Polyphosphazene–Polyester Blend Nanofiber Matrices for Load-Bearing Bone Regeneration. Advanced Functional …, Wiley Online Library, cited by 104 (13.00 per year)
X Li, R Cui, L Sun, KE Aifantis, Y Fan, Q Feng, ... (2014) 3D-printed biopolymers for tissue engineering application. International Journal of …, hindawi.com, cited by 95 (19.00 per year)
S Stevanovic, P Chavanne, O Braissant, ... (2013) Improvement of mechanical properties of 3d printed hydroxyapatite scaffolds by polymeric infiltration. … Dev Appl S, pdfs.semanticscholar.org, cited by 9 (1.50 per year)
L Moroni, JR De Wijn, CA Van Blitterswijk (2006) 3D fiber-deposited scaffolds for tissue engineering: influence of pores geometry and architecture on dynamic mechanical properties. Biomaterials, Elsevier, cited by 390 (30.00 per year)
TL Khuong, Z Gang, M Farid, R Yu, ZZ Sun, ... (2014) Tensile strength and flexural strength testing of acrylonitrile butadiene styrene (ABS) materials for biomimetic robotic applications. … of Biomimetics …, Trans Tech Publ, cited by 4 (0.80 per year)
T Zhang, H Zhang, L Zhang, S Jia, J Liu, Z Xiong, ... (2017) Biomimetic design and fabrication of multilayered osteochondral scaffolds by low-temperature deposition manufacturing and thermal-induced phase-separation …. …, iopscience.iop.org, cited by 15 (7.50 per year)
G Ahn, KH Min, C Kim, JS Lee, D Kang, JY Won, ... (2017) Precise stacking of decellularized extracellular matrix based 3D cell-laden constructs by a 3D cell printing system equipped with heating modules. Scientific reports, nature.com, cited by 21 (10.50 per year)
Moderator:, B Trimmer, Participants:, JA Lewis, ... (2015) 3d printing soft materials: what is possible?. Soft …, liebertpub.com, cited by 22 (5.50 per year)
S Khalil, W Sun (2009) Bioprinting endothelial cells with alginate for 3D tissue constructs. Journal of biomechanical …, … .asmedigitalcollection.asme.org, cited by 216 (21.60 per year)
YJ Choi, HG Yi, SW Kim, DW Cho (2017) 3D cell printed tissue analogues: a new platform for theranostics. Theranostics, ncbi.nlm.nih.gov, cited by 26 (13.00 per year)
HA Almeida, ESG Oliveira (2016) Sustainability based on biomimetic design models. Handbook of Sustainability in Additive …, Springer, cited by 5 (1.67 per year)

JH Shim, SE Kim, JY Park, J Kundu, SW Kim, ... (2014) Three-dimensional printing of rhBMP-2-loaded scaffolds with long-term delivery for enhanced bone regeneration in a rabbit diaphyseal defect. … Engineering Part A, liebertpub.com, cited by 58 (11.60 per year)
D Sundaramurthi, S Rauf, C Hauser (2016) 3D bioprinting technology for regenerative medicine applications. International Journal of …, ijb.whioce.com, cited by 18 (6.00 per year)
S Miao, W Zhu, NJ Castro, J Leng, ... (2016) Four-dimensional printing hierarchy scaffolds with highly biocompatible smart polymers for tissue engineering applications. Tissue Engineering Part …, liebertpub.com, cited by 28 (9.33 per year)
HH Lin, FY Hsieh, CS Tseng, S Hsu (2016) Preparation and characterization of a biodegradable polyurethane hydrogel and the hybrid gel with soy protein for 3D cell-laden bioprinting. Journal of Materials Chemistry B, pubs.rsc.org, cited by 21 (7.00 per year)
ZY Jonelle, E Korkmaz, MI Berg, PR LeDuc, ... (2017) Biomimetic scaffolds with three-dimensional undulated microtopographies. Biomaterials, Elsevier, cited by 9 (4.50 per year)
H Shahsavan, L Yu, A Jákli, B Zhao (2017) Smart biomimetic micro/nanostructures based on liquid crystal elastomers and networks. Soft Matter, pubs.rsc.org, cited by 13 (6.50 per year)
D Richards, J Jia, M Yost, R Markwald, ... (2017) 3D bioprinting for vascularized tissue fabrication. Annals of biomedical …, Springer, cited by 63 (31.50 per year)
CE Diesendruck, NR Sottos, JS Moore, ... (2015) Biomimetic self-healing. Angewandte Chemie …, Wiley Online Library, cited by 153 (38.25 per year)
K Kim, W Zhu, X Qu, C Aaronson, WR McCall, S Chen, ... (2014) 3D optical printing of piezoelectric nanoparticle–polymer composite materials. ACS …, ACS Publications, cited by 124 (24.80 per year)
MS Mannoor, Z Jiang, T James, YL Kong, ... (2013) 3D printed bionic ears. Nano …, ACS Publications, cited by 509 (84.83 per year)
W Zhu, X Qu, J Zhu, X Ma, S Patel, J Liu, P Wang, ... (2017) Direct 3D bioprinting of prevascularized tissue constructs with complex microarchitecture. Biomaterials, Elsevier, cited by 111 (55.50 per year)
A de Mel (2016) Three-dimensional printing and the surgeon. Br J Surg, researchgate.net, cited by 9 (3.00 per year)
R Chang, J Nam, W Sun (2008) Direct Cell Writing of 3D Microorgan for In Vitro Pharmacokinetic Model. Tissue Engineering Part C: Methods, liebertpub.com, cited by 159 (14.45 per year)
M Vaezi, H Seitz, S Yang (2013) A review on 3D micro-additive manufacturing technologies. The International Journal of Advanced …, Springer, cited by 645 (107.50 per year)
G Hunt, F Mitzalis, T Alhinai, ... (2014) 3D printing with flying robots. … on Robotics and …, ieeexplore.ieee.org, cited by 37 (7.40 per year)
E Palleau, S Reece, SC Desai, ME Smith, ... (2013) Self-healing stretchable wires for reconfigurable circuit wiring and 3D microfluidics. Advanced …, Wiley Online Library, cited by 230 (38.33 per year)
M Yang, N Zhao, Y Cui, W Gao, Q Zhao, C Gao, H Bai, ... (2017) Biomimetic architectured graphene aerogel with exceptional strength and resilience. ACS …, ACS Publications, cited by 58 (29.00 per year)
H Cui, M Nowicki, JP Fisher, ... (2017) 3D bioprinting for organ regeneration. Advanced healthcare …, Wiley Online Library, cited by 77 (38.50 per year)
SR Shin, R Farzad, A Tamayol, ... (2016) A bioactive carbon nanotube-based ink for printing 2D and 3D flexible electronics. Advanced …, Wiley Online Library, cited by 70 (23.33 per year)

DB Berry, S You, J Warner, LR Frank, ... (2017) A 3D Tissue-Printing Approach for Validation of Diffusion Tensor Imaging in Skeletal Muscle. … Engineering Part A, liebertpub.com, cited by 7 (3.50 per year)
YT Matsunaga, Y Morimoto, S Takeuchi (2011) Molding cell beads for rapid construction of macroscopic 3D tissue architecture. Advanced materials, Wiley Online Library, cited by 244 (30.50 per year)
H Gong, J Agustin, D Wootton, JG Zhou (2014) Biomimetic design and fabrication of porous chitosan–gelatin liver scaffolds with hierarchical channel network. Journal of Materials Science …, Springer, cited by 25 (5.00 per year)
K Jiang, C Dong, Y Xu, L Wang (2016) Microfluidic-based biomimetic models for life science research. RSC Advances, pubs.rsc.org, cited by 6 (2.00 per year)
KD Park, X Wang, JY Lee, ... (2016) Research trends in biomimetic medical materials for tissue engineering: commentary. Biomaterials …, biomaterialsres.biomedcentral.com, cited by 8 (2.67 per year)
S Zhang, Y Qian, P Liao, F Qin, ... (2016) Design and control of an agile robotic fish with integrative biomimetic mechanisms. IEEE/ASME Transactions …, ieeexplore.ieee.org, cited by 24 (8.00 per year)
T Serra, M Ortiz-Hernandez, E Engel, JA Planell, ... (2014) Relevance of PEG in PLA-based blends for tissue engineering 3D-printed scaffolds. Materials Science and …, Elsevier, cited by 98 (19.60 per year)
Y Luo, S Chen, Y Shi, J Ma (2018) 3D printing of strontium-doped hydroxyapatite based composite scaffolds for repairing critical-sized rabbit calvarial defects. Biomedical Materials, iopscience.iop.org, cited by 4 (4.00 per year)
D Mandt, P Gruber, M Markovic, ... (2018) Fabrication of biomimetic placental barrier structures within a microfluidic device utilizing two-photon polymerization. … Journal of Bioprinting, biblio.ugent.be, cited by 7 (7.00 per year)
D Xue, Y Wang, J Zhang, D Mei, Y Wang, ... (2018) Projection-Based 3D Printing of Cell Patterning Scaffolds with Multiscale Channels. ACS applied materials …, ACS Publications, cited by 4 (4.00 per year)
HG Hosseinabadi, R Bagheri, LA Gray, V Altstädt, ... (2017) Plasticity in polymeric honeycombs made by photo-polymerization and nozzle based 3D-printing. Polymer Testing, Elsevier, cited by 4 (2.00 per year)
J Zhou, L Lu, K Byrapogu, DM Wootton, ... (2007) Electrowetting-based multi-microfluidics array printing of high resolution tissue construct with embedded cells and growth factors. Virtual and Physical …, Taylor & Francis, cited by 29 (2.42 per year)
PX Lan, JW Lee, YJ Seol, DW Cho (2009) Development of 3D PPF/DEF scaffolds using micro-stereolithography and surface modification. Journal of Materials Science …, Springer, cited by 111 (11.10 per year)
P Hangge, Y Pershad, AA Witting, ... (2018) Three-dimensional (3D) printing and its applications for aortic diseases. Cardiovascular …, ncbi.nlm.nih.gov, cited by 4 (4.00 per year)
SY Jung, SJ Lee, HY Kim, HS Park, Z Wang, ... (2016) 3D printed polyurethane prosthesis for partial tracheal reconstruction: a pilot animal study. …, iopscience.iop.org, cited by 14 (4.67 per year)
K Picha, C Spackman, J Samuel (2016) Droplet spreading characteristics observed during 3D printing of aligned fiber-reinforced soft composites. Additive Manufacturing, Elsevier, cited by 34 (11.33 per year)

F Hanßke, O Bas, C Vaquette, G Hochleitner, ... (2017) Via precise interface engineering towards bioinspired composites with improved 3D printing processability and mechanical properties. Journal of Materials …, pubs.rsc.org, cited by 11 (5.50 per year)
BB Holmes, LG Zhang (2016) 3d biomimetic, bi-phasic key featured scaffold for osteochondral repair. US Patent App. 14/854,584, Google Patents, cited by 4 (1.33 per year)
D Hua, X Zhang, Z Ji, C Yan, B Yu, Y Li, ... (2018) 3D printing of shape changing composites for constructing flexible paper-based photothermal bilayer actuators. Journal of Materials …, pubs.rsc.org, cited by 14 (14.00 per year)
KM Farooqi, PP Sengupta (2015) Echocardiography and three-dimensional printing: sound ideas to touch a heart. Journal of the …, internationaljournalofcardiology.com, cited by 51 (12.75 per year)
B O'Brien, T Gisby, SQ Xie, E Calius, ... (2010) Biomimetic control for DEA arrays. … and Devices (EAPAD …, spiedigitallibrary.org, cited by 12 (1.33 per year)
Y Xu, X Wang (2015) Fluid and cell behaviors along a 3D printed alginate/gelatin/fibrin channel. Biotechnology and bioengineering, Wiley Online Library, cited by 34 (8.50 per year)
CG Helguero, JL Amaya, DE Komatsu, S Pentyala, ... (2017) Trabecular scaffolds' mechanical properties of bone reconstruction using biomimetic implants. Procedia Cirp, Elsevier, cited by 3 (1.50 per year)
A Lambert, S Valiulis, Q Cheng (2018) Advances in Optical Sensing and Bioanalysis Enabled by 3D Printing. ACS sensors, ACS Publications, cited by 3 (3.00 per year)
H Korhonen, LH Sinh, ND Luong, ... (2016) Fabrication of graphene-based 3D structures by stereolithography. … status solidi (a), Wiley Online Library, cited by 12 (4.00 per year)
H Xu, N Lu, D Qi, J Hao, L Gao, B Zhang, L Chi (2008) Biomimetic antireflective Si nanopillar arrays. Small, Wiley Online Library, cited by 96 (8.73 per year)
SA Park, JB Lee, YE Kim, JE Kim, JH Lee, ... (2014) Fabrication of biomimetic PCL scaffold using rapid prototyping for bone tissue engineering. Macromolecular …, Springer, cited by 15 (3.00 per year)
PS Gungor-Ozkerim, I Inci, YS Zhang, ... (2018) Bioinks for 3D bioprinting: an overview. Biomaterials …, pubs.rsc.org, cited by 42 (42.00 per year)
L Roseti, V Parisi, M Petretta, C Cavallo, ... (2017) Scaffolds for bone tissue engineering: state of the art and new perspectives. Materials Science and …, Elsevier, cited by 139 (69.50 per year)
ACS Thiele, LR de Menezes, EO da Silva, ... (2016) 3D printed scaffolds as a new perspective for bone tissue regeneration: literature review. Materials Sciences and …, m.scirp.org, cited by 10 (3.33 per year)
C Yang, B Wang, D Li, X Tian (2017) Modelling and characterisation for the responsive performance of CF/PLA and CF/PEEK smart materials fabricated by 4D printing. Virtual and Physical Prototyping, Taylor & Francis, cited by 19 (9.50 per year)
F Asa'ad, G Pagni, SP Pilipchuk, AB Giannì, ... (2016) 3D-printed scaffolds and biomaterials: review of alveolar bone augmentation and periodontal regeneration applications. International journal of …, hindawi.com, cited by 35 (11.67 per year)
D Wu, SZ Wu, J Xu, LG Niu, ... (2014) Hybrid femtosecond laser microfabrication to achieve true 3D glass/polymer composite biochips with multiscale features and high performance: the concept of ship-in …. Laser & Photonics …, Wiley Online Library, cited by 71 (14.20 per year)

N Xu, X Ye, D Wei, J Zhong, Y Chen, ... (2014) 3D artificial bones for bone repair prepared by computed tomography-guided fused deposition modeling for bone repair. ACS applied materials …, ACS Publications, cited by 68 (13.60 per year)
Y Liew, E Beveridge, AK Demetriades, ... (2015) 3D printing of patient-specific anatomy: a tool to improve patient consent and enhance imaging interpretation by trainees. British journal of …, Taylor & Francis, cited by 41 (10.25 per year)
M Mirkhalaf, F Barthelat (2017) Design, 3D printing and testing of architectured materials with bistable interlocks. Extreme Mechanics Letters, Elsevier, cited by 20 (10.00 per year)
F Yang, V Tadepalli, BJ Wiley (2017) 3D printing of a double network hydrogel with a compression strength and elastic modulus greater than those of cartilage. ACS Biomaterials Science & …, ACS Publications, cited by 35 (17.50 per year)
KY Su, JZ Gul, KH Choi (2017) A biomimetic jumping locomotion of functionally graded frog soft robot. 2017 14th International Conference …, ieeexplore.ieee.org, cited by 3 (1.50 per year)
S Huang, B Yao, J Xie, X Fu (2016) 3D bioprinted extracellular matrix mimics facilitate directed differentiation of epithelial progenitors for sweat gland regeneration. Acta biomaterialia, Elsevier, cited by 49 (16.33 per year)
TK Schuessler, XY Chan, HJ Chen, K Ji, KM Park, ... (2014) Biomimetic tissue–engineered systems for advancing cancer research: NCI strategic workshop report., AACR, cited by 21 (4.20 per year)
MM Khoshhesab, Y Li (2018) Mechanical behavior of 3D printed biomimetic Koch fractal contact and interlocking. Extreme Mechanics Letters, Elsevier, cited by 3 (3.00 per year)
J Zeltinger, JK Sherwood, DA Graham, ... (2001) Effect of pore size and void fraction on cellular adhesion, proliferation, and matrix deposition. Tissue …, liebertpub.com, cited by 758 (42.11 per year)
F Pati, DW Cho (2017) Bioprinting of 3D tissue models using decellularized extracellular matrix bioink. 3D Cell Culture, Springer, cited by 11 (5.50 per year)
R Mutlu, G Alici, M in het Panhuis, ... (2015) Effect of flexure hinge type on a 3D printed fully compliant prosthetic finger. 2015 IEEE International …, ieeexplore.ieee.org, cited by 18 (4.50 per year)
Y Zhou, W Sun, Q Ma, L Zhang (2018) Method of producing personalized biomimetic drug-eluting coronary stents by 3D-printing. US Patent 9,943,627, Google Patents, cited by 2 (2.00 per year)
B Winstone, T Pipe, C Melhuish, S Dogramadzi, ... (2015) Biomimetic tactile sensing capsule. … on Biomimetic and …, Springer, cited by 4 (1.00 per year)
BR Ringeisen, H Kim, JA Barron, DB Krizman, ... (2004) Laser printing of pluripotent embryonal carcinoma cells. Tissue …, liebertpub.com, cited by 218 (14.53 per year)
U Ripamonti, PW Richter, RWN Nilen, ... (2008) The induction of bone formation by smart biphasic hydroxyapatite tricalcium phosphate biomimetic matrices in the non-human primate Papio ursinus. Journal of cellular and …, Wiley Online Library, cited by 69 (6.27 per year)
GH Kim, JG Son, SA Park, ... (2008) Hybrid process for fabricating 3D hierarchical scaffolds combining rapid prototyping and electrospinning. Macromolecular rapid …, Wiley Online Library, cited by 112 (10.18 per year)
S Pedron, E Becka, BA Harley (2015) Spatially gradated hydrogel platform as a 3D engineered tumor microenvironment. Advanced Materials, Wiley Online Library, cited by 61 (15.25 per year)

S Malek, JR Raney, JA Lewis, ... (2017) Lightweight 3D cellular composites inspired by balsa. … & biomimetics, iopscience.iop.org, cited by 16 (8.00 per year)
D Han, C Farino, C Yang, T Scott, D Browe, ... (2018) Soft robotic manipulation and locomotion with a 3d printed electroactive hydrogel. … applied materials & …, ACS Publications, cited by 22 (22.00 per year)
Y He, FF Yang, HM Zhao, Q Gao, B Xia, JZ Fu (2016) Research on the printability of hydrogels in 3D bioprinting. Scientific reports, nature.com, cited by 158 (52.67 per year)
PJ Su, QA Tran, JJ Fong, KW Eliceiri, BM Ogle, ... (2012) Mesenchymal stem cell interactions with 3D ECM modules fabricated via multiphoton excited photochemistry. …, ACS Publications, cited by 29 (4.14 per year)
T Ramakrishnan, M Schlafly, ... (2017) Evaluation of 3D printed anatomically scalable transfemoral prosthetic knee. 2017 International …, ieeexplore.ieee.org, cited by 3 (1.50 per year)
M Girolami, S Boriani, S Bandiera, ... (2018) Biomimetic 3D-printed custom-made prosthesis for anterior column reconstruction in the thoracolumbar spine: a tailored option following en bloc resection for …. European Spine …, Springer, cited by 3 (3.00 per year)
Y Luo, A Lode, C Wu, J Chang, ... (2015) Alginate/nanohydroxyapatite scaffolds with designed core/shell structures fabricated by 3D plotting and in situ mineralization for bone tissue engineering. ACS applied materials & …, ACS Publications, cited by 66 (16.50 per year)
Z Yang, C Li, H Sun (2016) Research advances of three-dimension printing technology in vertebrae and intervertebral disc tissue engineering. Zhejiang da xue xue bao. Yi xue ban= Journal …, europepmc.org, cited by 3 (1.00 per year)
LT Kuhn, DJ Fink, AH Heuer (1996) Biomimetic strategies and materials processing., books.google.com, cited by 18 (0.78 per year)
X Song, H Tetik, T Jirakittsonthon, ... (2019) Biomimetic 3D Printing of Hierarchical and Interconnected Porous Hydroxyapatite Structures with High Mechanical Strength for Bone Cell Culture. Advanced …, Wiley Online Library, cited by 2 (2.00 per year)
NE Fedorovich, W Schuurman, ... (2011) Biofabrication of osteochondral tissue equivalents by printing topologically defined, cell-laden hydrogel scaffolds. … Engineering Part C …, liebertpub.com, cited by 263 (32.88 per year)
R MacCurdy, R Katzschmann, Y Kim, ... (2016) Printable hydraulics: A method for fabricating robots by 3D co-printing solids and liquids. 2016 IEEE International …, ieeexplore.ieee.org, cited by 85 (28.33 per year)
R Parwani, M Curto, AP Kao, PJ Rowley, ... (2017) Morphological and mechanical biomimetic bone structures. ACS Biomaterials …, ACS Publications, cited by 3 (1.50 per year)
BP Hung, BA Naved, EL Nyberg, M Dias, ... (2016) Three-dimensional printing of bone extracellular matrix for craniofacial regeneration. ACS biomaterials …, ACS Publications, cited by 55 (18.33 per year)
TBF Woodfield, CAV Blitterswijk, JD Wijn, ... (2005) Polymer scaffolds fabricated with pore-size gradients as a model for studying the zonal organization within tissue-engineered cartilage constructs. Tissue …, liebertpub.com, cited by 233 (16.64 per year)
I Teh, FL Zhou, PL Hubbard Cristinacce, ... (2016) Biomimetic phantom for cardiac diffusion MRI. Journal of Magnetic …, Wiley Online Library, cited by 17 (5.67 per year)
PX Ma, JW Choi (2001) Biodegradable polymer scaffolds with well-defined interconnected spherical pore network. Tissue engineering, liebertpub.com, cited by 623 (34.61 per year)

LM Bellan, M Pearsall, DM Cropek, ... (2012) A 3D interconnected microchannel network formed in gelatin by sacrificial shellac microfibers. Advanced …, Wiley Online Library, cited by 76 (10.86 per year)
T Bückmann, N Stenger, M Kadic, J Kaschke, ... (2012) Tailored 3D mechanical metamaterials made by dip-in direct-laser-writing optical lithography. Advanced …, Wiley Online Library, cited by 371 (53.00 per year)
A Ghanizadeh Tabriz, CG Mills, JJ Mullins, ... (2017) Rapid fabrication of cell-laden alginate hydrogel 3D structures by micro dip-coating. … in Bioengineering and …, frontiersin.org, cited by 7 (3.50 per year)
F Taraballi, G Bauza, P McCulloch, ... (2017) Concise review: biomimetic functionalization of biomaterials to stimulate the endogenous healing process of cartilage and bone tissue. Stem cells …, Wiley Online Library, cited by 7 (3.50 per year)
S Kale, N Khani, A Nadernezhad, B Koc (2017) Modeling and additive manufacturing of biomimetic heterogeneous Scaffold. Procedia CIRP, Elsevier, cited by 3 (1.50 per year)
P Xie, Z Hu, X Zhang, X Li, Z Gao, D Yuan, Q Liu (2014) Application of 3-dimensional printing technology to construct an eye model for fundus viewing study. PLoS One, journals.plos.org, cited by 26 (5.20 per year)
R Suntornnond, J An, CK Chua (2017) Roles of support materials in 3D bioprinting., dr.ntu.edu.sg, cited by 13 (6.50 per year)
S Wang, JM Lee, WY Yeong (2015) Smart hydrogels for 3D bioprinting. International Journal of Bioprinting, ijb.whioce.com, cited by 95 (23.75 per year)
EJ Mott, M Busso, X Luo, C Dolder, MO Wang, ... (2016) Digital micromirror device (DMD)-based 3D printing of poly (propylene fumarate) scaffolds. Materials Science and …, Elsevier, cited by 21 (7.00 per year)
H Ma, J Luo, Z Sun, L Xia, M Shi, M Liu, J Chang, C Wu (2016) 3D printing of biomaterials with mussel-inspired nanostructures for tumor therapy and tissue regeneration. Biomaterials, Elsevier, cited by 39 (13.00 per year)
B Wang, P Ghassemi, J Wang, Q Wang, ... (2016) Performance evaluation of CCD-and mobile-phone-based near-infrared fluorescence imaging systems with molded and 3D-printed phantoms. Design and Quality …, spiedigitallibrary.org, cited by 3 (1.00 per year)
N Celikkin, J Simó Padial, M Costantini, H Hendrikse, ... (2018) 3D printing of thermoresponsive polyisocyanide (PIC) hydrogels as bioink and fugitive material for tissue engineering. Polymers, mdpi.com, cited by 8 (8.00 per year)
LG Zhang, DL Kaplan (2016) Neural engineering: From advanced biomaterials to 3D fabrication techniques., books.google.com, cited by 5 (1.67 per year)
B Holmes, LG Zhang (2013) Enhanced human bone marrow mesenchymal stem cell functions in 3D bioprinted biologically inspired osteochondral construct. ASME 2013 …, … .asmedigitalcollection.asme.org, cited by 3 (0.50 per year)
AA Pawar, G Saada, I Cooperstein, ... (2016) High-performance 3D printing of hydrogels by water-dispersible photoinitiator nanoparticles. Science …, advances.sciencemag.org, cited by 50 (16.67 per year)
JW Lee (2015) 3D nanoprinting technologies for tissue engineering applications. Journal of Nanomaterials, dl.acm.org, cited by 15 (3.75 per year)
A Paul, V Manoharan, D Krafft, A Assmann, ... (2016) Nanoengineered biomimetic hydrogels for guiding human stem cell osteogenesis in three dimensional microenvironments. Journal of Materials …, pubs.rsc.org, cited by 55 (18.33 per year)

Z Cheng, B Landish, Z Chi, C Nannan, D Jingyu, ... (2018) 3D printing hydrogel with graphene oxide is functional in cartilage protection by influencing the signal pathway of Rank/Rankl/OPG. Materials Science and …, Elsevier, cited by 11 (11.00 per year)
CH Chang, CY Lin, FH Liu, MHC Chen, CP Lin, HN Ho, ... (2015) 3D printing bioceramic porous scaffolds with good mechanical property and cell affinity. PloS one, journals.plos.org, cited by 22 (5.50 per year)
T Yang, Y Hu, C Wang, BP Binks (2017) Fabrication of hierarchical macroporous biocompatible scaffolds by combining pickering high internal phase emulsion templates with three-dimensional printing. ACS applied materials & …, ACS Publications, cited by 30 (15.00 per year)
A Zolfagharian, AZ Kouzani, SY Khoo, ... (2016) 3D printed hydrogel soft actuators. 2016 IEEE Region …, ieeexplore.ieee.org, cited by 11 (3.67 per year)
Y Li, W Yang, X Li, X Zhang, C Wang, ... (2015) Improving osteointegration and osteogenesis of three-dimensional porous Ti6Al4V scaffolds by polydopamine-assisted biomimetic hydroxyapatite coating. … applied materials & …, ACS Publications, cited by 63 (15.75 per year)
X Du, S Fu, Y Zhu (2018) 3D printing of ceramic-based scaffolds for bone tissue engineering: an overview. Journal of Materials Chemistry B, pubs.rsc.org, cited by 13 (13.00 per year)
C Qi, J Liu, Y Jin, L Xu, G Wang, Z Wang, L Wang (2018) Photo-crosslinkable, injectable sericin hydrogel as 3D biomimetic extracellular matrix for minimally invasive repairing cartilage. Biomaterials, Elsevier, cited by 21 (21.00 per year)
AK Gaharwar, A Arpanaei, TL Andresen, ... (2016) 3D biomaterial microarrays for regenerative medicine: current state-of-the-art, emerging directions and future trends. Advanced …, Wiley Online Library, cited by 47 (15.67 per year)
JZ Gul, KY Su, KH Choi (2018) Fully 3D printed multi-material soft bio-inspired whisker sensor for underwater-induced vortex detection. Soft robotics, liebertpub.com, cited by 7 (7.00 per year)
G Montalbano, S Fiorilli, A Caneschi, ... (2018) Type I Collagen and Strontium-Containing Mesoporous Glass Particles as Hybrid Material for 3D Printing of Bone-Like Materials. Materials, mdpi.com, cited by 4 (4.00 per year)
S Gómez, MD Vlad, J López, E Fernández (2016) Design and properties of 3D scaffolds for bone tissue engineering. Acta biomaterialia, Elsevier, cited by 61 (20.33 per year)
LG Zhang, JP Fisher, K Leong (2015) 3D bioprinting and nanotechnology in tissue engineering and regenerative medicine., books.google.com, cited by 41 (10.25 per year)
JG Hardy, SA Geissler, D Aguilar Jr, ... (2015) Instructive Conductive 3D Silk Foam-Based Bone Tissue Scaffolds Enable Electrical Stimulation of Stem Cells for Enhanced Osteogenic Differentiation. Macromolecular …, Wiley Online Library, cited by 25 (6.25 per year)
CK Chua, CH Wong, WY Yeong (2017) Standards, quality control, and measurement sciences in 3D printing and additive manufacturing., books.google.com, cited by 38 (19.00 per year)
JH Park, JW Jung, HW Kang, DW Cho (2014) Indirect three-dimensional printing of synthetic polymer scaffold based on thermal molding process. Biofabrication, iopscience.iop.org, cited by 36 (7.20 per year)
JL Ifkovits, JA Burdick (2007) Photopolymerizable and degradable biomaterials for tissue engineering applications. Tissue engineering, liebertpub.com, cited by 508 (42.33 per year)
L Ciocca, IG Lesci, O Mezini, A Parrilli, ... (2017) Customized hybrid biomimetic hydroxyapatite scaffold for bone tissue regeneration. … Research Part B …, Wiley Online Library, cited by 10 (5.00 per year)

ME Kolewe, H Park, C Gray, X Ye, R Langer, ... (2013) 3D structural patterns in scalable, elastomeric scaffolds guide engineered tissue architecture. Advanced …, Wiley Online Library, cited by 77 (12.83 per year)
P Danilevicius, RA Rezende, FDAS Pereira, ... (2015) Burr-like, laser-made 3D microscaffolds for tissue spheroid encagement. …, avs.scitation.org, cited by 22 (5.50 per year)
NS Hmeidat, JW Kemp, BG Compton (2018) High-strength epoxy nanocomposites for 3D printing. Composites Science and …, Elsevier, cited by 9 (9.00 per year)
X Meng, DA Stout, L Sun, ... (2013) Novel injectable biomimetic hydrogels with carbon nanofibers and self assembled rosette nanotubes for myocardial applications. … research Part A, Wiley Online Library, cited by 39 (6.50 per year)
P Shi, A Laude, WY Yeong (2017) Investigation of cell viability and morphology in 3D bio-printed alginate constructs with tunable stiffness. Journal of Biomedical Materials …, Wiley Online Library, cited by 12 (6.00 per year)
B Nan, X Yin, L Zhang, L Cheng (2011) Three-Dimensional Printing of Ti3SiC2-Based Ceramics. Journal of the American …, Wiley Online Library, cited by 55 (6.88 per year)
J Odent, TJ Wallin, W Pan, ... (2017) Highly elastic, transparent, and conductive 3D-printed ionic composite hydrogels. Advanced Functional …, Wiley Online Library, cited by 29 (14.50 per year)
AD Lantada, D Curras, J Mousa, ... (2016) Tissue engineering scaffolds for 3D cell culture. Microsystems for Enhanced …, Springer, cited by 4 (1.33 per year)
SA Morin, Y Shevchenko, J Lessing, ... (2014) Using "Click-e-Bricks" to Make 3D Elastomeric Structures. Advanced …, Wiley Online Library, cited by 51 (10.20 per year)
K Haberstroh, K Ritter, J Kuschnierz, ... (2010) Bone repair by cell-seeded 3D-bioplotted composite scaffolds made of collagen treated tricalciumphosphate or tricalciumphosphate-chitosan-collagen hydrogel or …. … Research Part B …, Wiley Online Library, cited by 54 (6.00 per year)
KA Kwakwa, JP Vanderburgh, SA Guelcher, ... (2017) Engineering 3D models of tumors and bone to understand tumor-induced bone disease and improve treatments. Current osteoporosis …, Springer, cited by 5 (2.50 per year)
J Hardy, R Cornelison, R Sukhavasi, R Saballos, P Vu, ... (2015) Electroactive tissue scaffolds with aligned pores as instructive platforms for biomimetic tissue engineering. Bioengineering, mdpi.com, cited by 26 (6.50 per year)
T Jacobs (2016) 3D printing in the oil field kicks into production mode. Journal of Petroleum Technology, onepetro.org, cited by 3 (1.00 per year)
Q Jiang, X Feng, L Song, Y Gong, H Zheng, J Cui (2016) Modeling rock specimens through 3D printing: Tentative experiments and prospects. Acta Mechanica Sinica, Springer, cited by 31 (10.33 per year)
X Feng, Z Yang, S Chmely, Q Wang, S Wang, ... (2017) Lignin-coated cellulose nanocrystal filled methacrylate composites prepared via 3D stereolithography printing: Mechanical reinforcement and thermal …. Carbohydrate polymers, Elsevier, cited by 27 (13.50 per year)
DL Cohen, E Malone, HOD Lipson, ... (2006) Direct freeform fabrication of seeded hydrogels in arbitrary geometries. Tissue engineering, liebertpub.com, cited by 319 (24.54 per year)
M Shin, H Yoshimoto, JP Vacanti (2004) In Vivo Bone Tissue Engineering Using Mesenchymal Stem Cells on a Novel Electrospun Nanofibrous Scaffold. Tissue engineering, liebertpub.com, cited by 495 (33.00 per year)
M Azami, MJ Moosavifar, N Baheiraei, ... (2012) Preparation of a biomimetic nanocomposite scaffold for bone tissue engineering via mineralization of gelatin hydrogel and study of

mineral transformation in simulated …. … Research Part A, Wiley Online Library, cited by 41 (5.86 per year)
JJ Moon, SH Lee, JL West (2007) Synthetic biomimetic hydrogels incorporated with ephrin-A1 for therapeutic angiogenesis. Biomacromolecules, ACS Publications, cited by 110 (9.17 per year)
WG Whitford, JB Hoying (2016) A bioink by any other name: terms, concepts and constructions related to 3D bioprinting., Future Science, cited by 7 (2.33 per year)
Z Wu, X Su, Y Xu, B Kong, W Sun, S Mi (2016) Bioprinting three-dimensional cell-laden tissue constructs with controllable degradation. Scientific reports, nature.com, cited by 134 (44.67 per year)
W Zhang, C Feng, G Yang, G Li, X Ding, S Wang, ... (2017) 3D-printed scaffolds with synergistic effect of hollow-pipe structure and bioactive ions for vascularized bone regeneration. Biomaterials, Elsevier, cited by 44 (22.00 per year)
FC Fierz, F Beckmann, M Huser, SH Irsen, B Leukers, ... (2008) The morphology of anisotropic 3D-printed hydroxyapatite scaffolds. Biomaterials, Elsevier, cited by 151 (13.73 per year)
S Lapidot, S Meirovitch, S Sharon, A Heyman, ... (2012) Clues for biomimetics from natural composite materials. …, Future Medicine, cited by 31 (4.43 per year)
RE Abouzeid, R Khiari, D Beneventi, ... (2018) Biomimetic mineralization of three-dimensional printed alginate/TEMPO-oxidized cellulose nanofibril scaffolds for bone tissue engineering. …, ACS Publications, cited by 4 (4.00 per year)
JH Park, J Jang, JS Lee, DW Cho (2016) Current advances in three-dimensional tissue/organ printing. Tissue engineering and regenerative …, Springer, cited by 16 (5.33 per year)
WS Chu, KT Lee, SH Song, MW Han, JY Lee, ... (2012) Review of biomimetic underwater robots using smart actuators. International journal of …, Springer, cited by 210 (30.00 per year)
S Kuttappan, D Mathew, MB Nair (2016) Biomimetic composite scaffolds containing bioceramics and collagen/gelatin for bone tissue engineering-A mini review. International journal of biological …, Elsevier, cited by 49 (16.33 per year)
CY Kuo, A Eranki, JK Placone, KR Rhodes, ... (2016) Development of a 3D printed, bioengineered placenta model to evaluate the role of trophoblast migration in preeclampsia. ACS Biomaterials …, ACS Publications, cited by 28 (9.33 per year)
N Scoutaris, S Ross, D Douroumis (2016) Current trends on medical and pharmaceutical applications of inkjet printing technology. Pharmaceutical research, Springer, cited by 33 (11.00 per year)
HE Abaci, A Coffman, Y Doucet, J Chen, ... (2018) Tissue engineering of human hair follicles using a biomimetic developmental approach. Nature …, nature.com, cited by 5 (5.00 per year)
NA Sears, PS Dhavalikar, ... (2016) Emulsion inks for 3D printing of high porosity materials. Macromolecular rapid …, Wiley Online Library, cited by 20 (6.67 per year)
MC Melican, MC Zimmerman, ... (2001) Three-dimensional printing and porous metallic surfaces: A new orthopedic application. … Research: An Official …, Wiley Online Library, cited by 69 (3.83 per year)
TA Ward, M Rezadad, CJ Fearday, ... (2015) A review of biomimetic air vehicle research: 1984-2014. … Journal of Micro Air …, journals.sagepub.com, cited by 41 (10.25 per year)
HW Han, S Hsu (2017) Using 3D bioprinting to produce mini-brain. Neural regeneration research, ncbi.nlm.nih.gov, cited by 7 (3.50 per year)
Y Luo, X Lin, P Huang (2018) 3D bioprinting of artificial tissues: Construction of biomimetic microstructures. Macromolecular bioscience, Wiley Online Library, cited by 2 (2.00 per year)

N Mehrban, GZ Teoh, MA Birchall (2016) 3D bioprinting for tissue engineering: Stem cells in hydrogels. International journal of …, discovery.ucl.ac.uk, cited by 31 (10.33 per year)
YJ Seol, DY Park, JY Park, SW Kim, ... (2013) A new method of fabricating robust freeform 3D ceramic scaffolds for bone tissue regeneration. Biotechnology and …, Wiley Online Library, cited by 69 (11.50 per year)
Y Tong, JM Murbach, V Subramanian, S Chhatre, ... (2018) A hybrid 3D printing and robotic-assisted embedding approach for design and fabrication of nerve cuffs with integrated locking mechanisms. MRS …, cambridge.org, cited by 3 (3.00 per year)
P Phamduy, MA Vazquez, C Kim, V Mwaffo, ... (2017) Design and characterization of a miniature free-swimming robotic fish based on multi-material 3D printing. International Journal of …, Springer, cited by 7 (3.50 per year)
D Drotman, S Jadhav, M Karimi, ... (2017) 3D printed soft actuators for a legged robot capable of navigating unstructured terrain. … on Robotics and …, ieeexplore.ieee.org, cited by 35 (17.50 per year)
S Wüst, R Müller, S Hofmann (2015) 3D B ioprinting of complex channels—Effects of material, orientation, geometry, and cell embedding. Journal of Biomedical Materials …, Wiley Online Library, cited by 39 (9.75 per year)
T Cordonnier, J Sohier, P Rosset, ... (2011) Biomimetic materials for bone tissue engineering–state of the art and future trends. Advanced Engineering …, Wiley Online Library, cited by 48 (6.00 per year)
HB LAN, DC LI, BH LU (2015) Micro-and nanoscale 3D printing. Scientia Sinica Technologica, engine.scichina.com, cited by 7 (1.75 per year)
RR Ma, JT Belter, AM Dollar (2015) Hybrid deposition manufacturing: design strategies for multimaterial mechanisms via three-dimensional printing and material deposition. Journal of …, … .asmedigitalcollection.asme.org, cited by 46 (11.50 per year)
M Costantini, S Testa, P Mozetic, A Barbetta, C Fuoco, ... (2017) Microfluidic-enhanced 3D bioprinting of aligned myoblast-laden hydrogels leads to functionally organized myofibers in vitro and in vivo. Biomaterials, Elsevier, cited by 37 (18.50 per year)
TK Merceron, SV Murphy (2015) Hydrogels for 3D bioprinting applications. Essentials of 3D biofabrication and translation, Elsevier, cited by 7 (1.75 per year)
L Wu, Z Dong, H Du, C Li, NX Fang, Y Song (2018) Bioinspired Ultra-Low Adhesive Energy Interface for Continuous 3D Printing: Reducing Curing Induced Adhesion. Research, spj.sciencemag.org, cited by 2 (2.00 per year)
GX Gu, F Libonati, SD Wettermark, ... (2017) Printing nature: Unraveling the role of nacre's mineral bridges. Journal of the mechanical …, Elsevier, cited by 44 (22.00 per year)
X Dai, L Liu, J Ouyang, X Li, X Zhang, Q Lan, T Xu (2017) Coaxial 3D bioprinting of self-assembled multicellular heterogeneous tumor fibers. Scientific reports, nature.com, cited by 23 (11.50 per year)
PJS Bártolo, H Almeida, T Laoui (2009) Rapid prototyping and manufacturing for tissue engineering scaffolds. International Journal of Computer …, dl.acm.org, cited by 74 (7.40 per year)
A Barba, Y Maazouz, A Diez-Escudero, K Rappe, ... (2018) Osteogenesis by foamed and 3D-printed nanostructured calcium phosphate scaffolds: Effect of pore architecture. Acta biomaterialia, Elsevier, cited by 9 (9.00 per year)
E Salami, PB Ganesan, TA Ward, ... (2016) Design and mechanical analysis of a 3D-printed biodegradable biomimetic micro air vehicle wing. IOP Conference …, iopscience.iop.org, cited by 2 (0.67 per year)

I Casanellas, A García-Lizarribar, ... (2018) Producing 3D biomimetic nanomaterials for musculoskeletal system regeneration. … in bioengineering and …, ncbi.nlm.nih.gov, cited by 2 (2.00 per year)
X Tu, L Wang, J Wei, B Wang, Y Tang, J Shi, ... (2016) 3D printed PEGDA microstructures for gelatin scaffold integration and neuron differentiation. Microelectronic …, Elsevier, cited by 8 (2.67 per year)
W Gao, L Wang, X Wang, H Liu (2016) Magnetic driving flowerlike soft platform: biomimetic fabrication and external regulation. ACS applied materials & …, ACS Publications, cited by 31 (10.33 per year)
B Antebi, X Cheng, JN Harris, LB Gower, ... (2013) Biomimetic collagen–hydroxyapatite composite fabricated via a novel perfusion-flow mineralization technique. … Engineering Part C …, liebertpub.com, cited by 55 (9.17 per year)
APC Almeida, JP Canejo, SN Fernandes, ... (2018) Cellulose-based biomimetics and their applications. Advanced …, Wiley Online Library, cited by 25 (25.00 per year)
TS Jang, HD Jung, MH Pan, WT Han, S Chen, J Song (2018) 3D printing of hydrogel composite systems: Recent advances in technology for tissue engineering., dr.ntu.edu.sg, cited by 10 (10.00 per year)
F Khan, SR Ahmad (2012) Fabrication of 3D Scaffolds and Organ Printing for Tissue Regeneration. Biomaterials and Stem Cells in …, content.taylorfrancis.com, cited by 3 (0.43 per year)
J Taboas, RD Maddox, PH Krebsbach, ... (2006) Controlled local/global and micro/macro-porous 3D plastic, polymer and ceramic/cement composite scaffold fabrication and applications thereof. US Patent …, Google Patents, cited by 116 (8.92 per year)
Y Yang, X Li, X Zheng, Z Chen, Q Zhou, ... (2018) Superhydrophobicity: 3D-Printed Biomimetic Super-Hydrophobic Structure for Microdroplet Manipulation and Oil/Water Separation (Adv. Mater. 9/2018). Advanced …, Wiley Online Library, cited by 2 (2.00 per year)
J Wang, Z Nor Hidayah, SIA Razak, MRA Kadir, ... (2019) Surface entrapment of chitosan on 3D printed polylactic acid scaffold and its biomimetic growth of hydroxyapatite. Composite …, Taylor & Francis, cited by 2 (2.00 per year)
P Walters, D McGoran (2011) Digital fabrication of "smart" structures and mechanisms-creative applications in art and design. NIP & Digital Fabrication Conference, ingentaconnect.com, cited by 22 (2.75 per year)
C Severini, A Derossi, I Ricci, R Caporizzi, ... (2018) Printing a blend of fruit and vegetables. New advances on critical variables and shelf life of 3D edible objects. Journal of Food …, Elsevier, cited by 17 (17.00 per year)
M Prakasam, M Popescu, R Piticescu, ... (2017) Fabrication methodologies of biomimetic and bioactive scaffolds for tissue engineering applications. Scaffolds in Tissue …, books.google.com, cited by 3 (1.50 per year)
ET Baran, K Tuzlakoglu, AJ Salgado, ... (2004) Multichannel mould processing of 3D structures from microporous coralline hydroxyapatite granules and chitosan support materials for guided tissue regeneration …. Journal of Materials …, Springer, cited by 41 (2.73 per year)
MB Chan-Park, JY Shen, Y Cao, ... (2009) Biomimetic control of vascular smooth muscle cell morphology and phenotype for functional tissue-engineered small-diameter blood vessels. … Research Part A: An …, Wiley Online Library, cited by 123 (12.30 per year)

J Xiongfa, Z Hao, Z Liming, X Jun (2018) Recent advances in 3D bioprinting for the regeneration of functional cartilage. Regenerative medicine, Future Medicine, cited by 3 (3.00 per year)

M Milwich, T Speck, O Speck, ... (2006) Biomimetics and technical textiles: solving engineering problems with the help of nature's wisdom. American Journal of …, Wiley Online Library, cited by 132 (10.15 per year)

KW Lee, S Wang, L Lu, E Jabbari, BL Currier, ... (2006) Fabrication and characterization of poly (propylene fumarate) scaffolds with controlled pore structures using 3-dimensional printing and injection molding. Tissue …, liebertpub.com, cited by 117 (9.00 per year)

C Majidi (2014) Soft robotics: a perspective—current trends and prospects for the future. Soft Robotics, liebertpub.com, cited by 506 (101.20 per year)

C Ma, X Le, X Tang, J He, P Xiao, ... (2016) A multiresponsive anisotropic hydrogel with macroscopic 3D complex deformations. Advanced Functional …, Wiley Online Library, cited by 61 (20.33 per year)

A Tamayol, AH Najafabadi, ... (2015) Hydrogel templates for rapid manufacturing of bioactive fibers and 3D constructs. Advanced …, Wiley Online Library, cited by 63 (15.75 per year)

GZ Yu, DT Chou, D Hong, A Roy, ... (2017) Biomimetic rotated lamellar plywood motifs by additive manufacturing of metal alloy scaffolds for bone tissue engineering. … biomaterials science & …, ACS Publications, cited by 4 (2.00 per year)

JE Trachtenberg, JK Placone, BT Smith, ... (2017) Extrusion-based 3D printing of poly (propylene fumarate) scaffolds with hydroxyapatite gradients. Journal of …, Taylor & Francis, cited by 29 (14.50 per year)

TAE Ahmed, EV Dare, M Hincke (2008) Fibrin: a versatile scaffold for tissue engineering applications. Tissue Engineering Part B …, liebertpub.com, cited by 731 (66.45 per year)

JY Lim, HJ Donahue (2007) Cell sensing and response to micro-and nanostructured surfaces produced by chemical and topographic patterning. Tissue engineering, liebertpub.com, cited by 484 (40.33 per year)

Y Zeng, Y Yan, H Yan, C Liu, P Li, P Dong, ... (2018) 3D printing of hydroxyapatite scaffolds with good mechanical and biocompatible properties by digital light processing. Journal of materials …, Springer, cited by 16 (16.00 per year)

J Steckel, H Peremans (2013) BatSLAM: Simultaneous localization and mapping using biomimetic sonar. PloS one, journals.plos.org, cited by 45 (7.50 per year)

J Du, H Chen, L Qing, X Yang, X Jia (2018) Biomimetic neural scaffolds: a crucial step towards optimal peripheral nerve regeneration. Biomaterials science, pubs.rsc.org, cited by 5 (5.00 per year)

W Shi, M Sun, X Hu, B Ren, J Cheng, C Li, ... (2017) Structurally and functionally optimized silk-fibroin–gelatin scaffold using 3D printing to repair cartilage injury in vitro and in vivo. Advanced …, Wiley Online Library, cited by 55 (27.50 per year)

AM Compaan, K Christensen, ... (2016) Inkjet bioprinting of 3D silk fibroin cellular constructs using sacrificial alginate. ACS Biomaterials Science …, ACS Publications, cited by 24 (8.00 per year)

JP Aguilar, M Lipka, GA Primo, ... (2018) 3D Electrophoresis-Assisted Lithography (3DEAL): 3D Molecular Printing to Create Functional Patterns and Anisotropic Hydrogels. Advanced Functional …, Wiley Online Library, cited by 6 (6.00 per year)

RR Naik, S Singamaneni (2017) Introduction: Bioinspired and biomimetic materials., ACS Publications, cited by 11 (5.50 per year)

JM Kemppainen, SJ Hollister (2010) Tailoring the mechanical properties of 3D-designed poly (glycerol sebacate) scaffolds for cartilage applications. … Part A: An Official Journal of …, Wiley Online Library, cited by 117 (13.00 per year)
J Yin, M Yan, Y Wang, J Fu, H Suo (2018) 3D bioprinting of low-concentration cell-laden gelatin methacrylate (GelMA) bioinks with a two-step cross-linking strategy. ACS applied materials & …, ACS Publications, cited by 36 (36.00 per year)
CT Nguyen, H Phung, TD Nguyen, ... (2014) Biomimetic printable hexapod robot driven by soft actuator. … Robots and Ambient …, ieeexplore.ieee.org, cited by 2 (0.40 per year)
BJ Brooks, KM Arif, S Dirven, J Potgieter (2017) Robot-assisted 3D printing of biopolymer thin shells. The International Journal of …, Springer, cited by 10 (5.00 per year)
SH Ahn, KT Lee, HJ Kim, R Wu, JS Kim, ... (2012) Smart soft composite: An integrated 3D soft morphing structure using bend-twist coupling of anisotropic materials. International Journal of …, Springer, cited by 110 (15.71 per year)
X Zhang, Y Zhang (2015) Tissue engineering applications of three-dimensional bioprinting. Cell biochemistry and biophysics, Springer, cited by 74 (18.50 per year)
S Alonso-Sierra, R Velázquez-Castillo, ... (2017) Interconnected porosity analysis by 3D X-ray microtomography and mechanical behavior of biomimetic organic-inorganic composite materials. Materials Science and …, Elsevier, cited by 8 (4.00 per year)
B Aldemìr, S Dıkıcı, O Karaman, ... (2015) Development, 3D printing and characterization of calcium sulfate based scaffolds for bone tissue engineering. 2015 19th National …, ieeexplore.ieee.org, cited by 2 (0.50 per year)
Y Zuo, X He, Y Yang, D Wei, J Sun, M Zhong, R Xie, ... (2016) Microfluidic-based generation of functional microfibers for biomimetic complex tissue construction. Acta biomaterialia, Elsevier, cited by 23 (7.67 per year)
V Guneta, JK Wang, S Maleksaeedi, ... (2014) Three dimensional printing of titanium for bone tissue engineering applications: a preliminary study. … of Biomimetics …, Trans Tech Publ, cited by 10 (2.00 per year)
DC Zuluaga, A Menges (2015) 3D printed hygroscopic programmable material systems. MRS Online Proceedings Library Archive, cambridge.org, cited by 4 (1.00 per year)
X Li, Y Li, Y Zuo, D Qu, Y Liu, T Chen, ... (2015) Osteogenesis and chondrogenesis of biomimetic integrated porous PVA/gel/V-n-HA/pa6 scaffolds and BMSCs construct in repair of articular osteochondral defect. … Research Part A, Wiley Online Library, cited by 17 (4.25 per year)
JT Schantz, DW Hutmacher, CXF Lam, ... (2003) Repair of Calvarial Defects with Customised Tissue-Engineered Bone Grafts II. Evaluation of Cellular Efficiency and Efficacy in Vivo. Tissue …, liebertpub.com, cited by 237 (14.81 per year)
N Meisel, A Gaynor, C Williams, ... (2013) Multiple-material topology optimization of compliant mechanisms created via polyjet 3d printing. … symposium an additive …, pdfs.semanticscholar.org, cited by 24 (4.00 per year)
R Narayan (2014) Rapid prototyping of biomaterials: principles and applications., books.google.com, cited by 25 (5.00 per year)
PG Campbell, LE Weiss (2007) Tissue engineering with the aid of inkjet printers. Expert opinion on biological therapy, Taylor & Francis, cited by 164 (13.67 per year)
W Zhu, KR Tringale, SA Woller, S You, S Johnson, ... (2018) Rapid continuous 3D printing of customizable peripheral nerve guidance conduits. Materials Today, Elsevier, cited by 6 (6.00 per year)

R Murugan, S Ramakrishna (2007) Design strategies of tissue engineering scaffolds with controlled fiber orientation. Tissue engineering, liebertpub.com, cited by 398 (33.17 per year)
S Vijayavenkataraman, L Zhang, S Zhang, ... (2018) Triply Periodic Minimal Surfaces Sheet Scaffolds for Tissue Engineering Applications: An Optimization Approach toward Biomimetic Scaffold Design. ACS Applied Bio …, ACS Publications, cited by 6 (6.00 per year)
Y Jiang, Y Li (2017) 3D printed chiral cellular solids with amplified auxetic effects due to elevated internal rotation. Advanced Engineering Materials, Wiley Online Library, cited by 28 (14.00 per year)
EY Tsai (2012) 4D printing: towards biomimetic additive manufacturing., dspace.mit.edu, cited by 2 (0.29 per year)
YM Kook, Y Jeong, K Lee, ... (2017) Design of biomimetic cellular scaffolds for co-culture system and their application. Journal of tissue …, journals.sagepub.com, cited by 12 (6.00 per year)
S Das, SJ Hollister, C Flanagan, ... (2003) Freeform fabrication of Nylon-6 tissue engineering scaffolds. Rapid Prototyping …, emeraldinsight.com, cited by 106 (6.63 per year)
C Rocchini, P Cignoni, C Montani, ... (2001) A low cost 3D scanner based on structured light. Computer Graphics …, Wiley Online Library, cited by 353 (19.61 per year)
CM Teven, S Fisher, GA Ameer, TC He, ... (2015) Biomimetic approaches to complex craniofacial defects. Annals of maxillofacial …, ncbi.nlm.nih.gov, cited by 21 (5.25 per year)
D McCoul, S Rosset, S Schlatter, ... (2017) Inkjet 3D printing of UV and thermal cure silicone elastomers for dielectric elastomer actuators. Smart Materials and …, iopscience.iop.org, cited by 19 (9.50 per year)
JA Szivek, DA Gonzales, ... (2019) Mesenchymal stem cell seeded, biomimetic 3D printed scaffolds induce complete bridging of femoral critical sized defects. … Research Part B …, Wiley Online Library, cited by 3 (3.00 per year)
A Abbadessa, MM Blokzijl, VHM Mouser, P Marica, ... (2016) A thermo-responsive and photo-polymerizable chondroitin sulfate-based hydrogel for 3D printing applications. Carbohydrate …, Elsevier, cited by 39 (13.00 per year)
TS Shim, SH Kim, CJ Heo, HC Jeon, ... (2012) Controlled origami folding of hydrogel bilayers with sustained reversibility for robust microcarriers. Angewandte Chemie …, Wiley Online Library, cited by 142 (20.29 per year)
WC Wilson Jr, T Boland (2003) Cell and organ printing 1: protein and cell printers. The Anatomical Record Part A …, Wiley Online Library, cited by 407 (25.44 per year)
AD Lantada, A de Blas Romero, ... (2017) Monolithic 3D labs-and organs-on-chips obtained by lithography-based ceramic manufacture. … International Journal of …, Springer, cited by 5 (2.50 per year)
PN Sivasankaran, TA Ward, E Salami, ... (2017) An experimental study of elastic properties of dragonfly-like flapping wings for use in biomimetic micro air vehicles (BMAVs). Chinese Journal of …, Elsevier, cited by 5 (2.50 per year)
S Saghati, A Akbarzadeh, AR Del Bakhshayesh, ... (2018) Electrospinning and 3D Printing: Prospects for Market Opportunity. Electrospinning, pubs.rsc.org, cited by 2 (2.00 per year)
Y Jin, X Li, RI Campbell, S Ji (2018) Visualizing the hotspots and emerging trends of 3D printing through scientometrics. Rapid Prototyping Journal, emeraldinsight.com, cited by 4 (4.00 per year)
R Domingo-Roca, JC Jackson, ... (2017) Bioinspired 3D-printed piezoelectric device for acoustic frequency separation. 2017 IEEE …, ieeexplore.ieee.org, cited by 2 (1.00 per year)

Z Chen, X Zhang, P Chen, W Li, K Zhou, L Shi, ... (2017) 3D multi-nozzle system with dual drives highly potential for 3D complex scaffolds with multi-biomaterials. International Journal of …, Springer, cited by 3 (1.50 per year)
Y He, S Guo, L Shi, S Pan, ... (2014) 3D printing technology-based an amphibious spherical robot. 2014 IEEE International …, ieeexplore.ieee.org, cited by 23 (4.60 per year)
O Rios, W Carter, B Post, P Lloyd, D Fenn, ... (2018) 3D printing via ambient reactive extrusion. Materials Today …, Elsevier, cited by 4 (4.00 per year)
Y Tsukamoto, T Akagi, F Shima, ... (2017) Fabrication of orientation-controlled 3D tissues using a layer-by-layer technique and 3D printed a thermoresponsive gel frame. Tissue Engineering Part C …, liebertpub.com, cited by 9 (4.50 per year)
A Marino, J Barsotti, G de Vito, ... (2015) Two-photon lithography of 3D nanocomposite piezoelectric scaffolds for cell stimulation. … applied materials & …, ACS Publications, cited by 37 (9.25 per year)
X Wang, Y Yan, Y Pan, Z Xiong, H Liu, J Cheng, ... (2006) Generation of three-dimensional hepatocyte/gelatin structures with rapid prototyping system. Tissue …, liebertpub.com, cited by 246 (18.92 per year)
A Silvestri, M Boffito, S Sartori, ... (2013) Biomimetic materials and scaffolds for myocardial tissue regeneration. Macromolecular …, Wiley Online Library, cited by 54 (9.00 per year)
M Vatani, Y Lu, ED Engeberg, JW Choi (2015) Combined 3D printing technologies and material for fabrication of tactile sensors. International Journal of Precision …, Springer, cited by 41 (10.25 per year)
PF Costa, DW Hutmacher, ... (2015) Additively manufactured device for dynamic culture of large arrays of 3D tissue engineered constructs. Advanced …, Wiley Online Library, cited by 11 (2.75 per year)
A Ovsianikov, J Yoo, V Mironov (2018) 3D Printing and Biofabrication., Springer, cited by 2 (2.00 per year)
MJ Mirzaali, ME Edens, AH de la Nava, S Janbaz, ... (2018) Length-scale dependency of biomimetic hard-soft composites. Scientific reports, nature.com, cited by 2 (2.00 per year)
YB Kim, H Lee, GH Kim (2016) Strategy to achieve highly porous/biocompatible macroscale cell blocks, using a collagen/genipin-bioink and an optimal 3D printing process. ACS applied materials & interfaces, ACS Publications, cited by 29 (9.67 per year)
RP Rimington, AJ Capel, SDR Christie, MP Lewis (2017) Biocompatible 3D printed polymers via fused deposition modelling direct C 2 C 12 cellular phenotype in vitro. Lab on a Chip, pubs.rsc.org, cited by 14 (7.00 per year)
BN Johnson, MC McAlpine (2016) From print to patient: 3D-printed personalized nerve regeneration. Biochemist, biochemist.org, cited by 2 (0.67 per year)
A Accardo, R Courson, R Riesco, V Raimbault, ... (2018) Direct laser fabrication of meso-scale 2D and 3D architectures with micrometric feature resolution. Additive …, Elsevier, cited by 2 (2.00 per year)
R Mutlu, C Tawk, G Alici, ... (2017) A 3D printed monolithic soft gripper with adjustable stiffness. IECON 2017-43rd Annual …, ieeexplore.ieee.org, cited by 4 (2.00 per year)
SJ Lee, W Zhu, N Castro, LG Zhang (2016) Biomaterials and 3D printing techniques for neural tissue regeneration. Neural Engineering, Springer, cited by 2 (0.67 per year)
L Saharan, Y Tadesse (2016) Robotic hand with locking mechanism using TCP muscles for applications in prosthetic hand and humanoids. … , Biomimetics, and Bioreplication 2016, spiedigitallibrary.org, cited by 30 (10.00 per year)

Y Wu, J Fuh, YS Wong, J Sun (2017) A hybrid electrospinning and electrospraying 3D printing for tissue engineered scaffolds. Rapid Prototyping Journal, emeraldinsight.com, cited by 3 (1.50 per year)
M Yamato, M Utsumi, AI Kushida, C Konno, ... (2001) Thermo-responsive culture dishes allow the intact harvest of multilayered keratinocyte sheets without dispase by reducing temperature. Tissue …, liebertpub.com, cited by 408 (22.67 per year)
LC Mozdzen, R Rodgers, JM Banks, RC Bailey, ... (2016) Increasing the strength and bioactivity of collagen scaffolds using customizable arrays of 3D-printed polymer fibers. Acta biomaterialia, Elsevier, cited by 33 (11.00 per year)
YH Hsieh, BY Shen, YH Wang, B Lin, HM Lee, ... (2018) Healing of Osteochondral Defects Implanted with Biomimetic Scaffolds of Poly (ε-Caprolactone)/Hydroxyapatite and Glycidyl-Methacrylate-Modified Hyaluronic Acid in …. International journal of …, mdpi.com, cited by 4 (4.00 per year)
S Krishna, K Small, T Maetani, L Chepelev, ... (2017) Musculoskeletal 3D Printing. 3D Printing in …, Springer, cited by 2 (1.00 per year)
RJ Jackson, PS Patrick, K Page, MJ Powell, ... (2018) Chemically Treated 3D Printed Polymer Scaffolds for Biomineral Formation. ACS …, ACS Publications, cited by 6 (6.00 per year)
CC Wang, KC Yang, KH Lin, CC Wu, ... (2014) A biomimetic honeycomb-like scaffold prepared by flow-focusing technology for cartilage regeneration. Biotechnology and …, Wiley Online Library, cited by 18 (3.60 per year)
MK Lee, A DeConde, T Aghaloo, ... (2013) Biomimetic scaffolds loaded with adipose-derived stem cells and BMP-2 induce healing of mandibular defects. … —Head and Neck …, journals.sagepub.com, cited by 2 (0.33 per year)
ME Hoque, G Daei, J Mahmoud, ... (2018) Next generation biomimetic bone tissue engineering matrix from poly (L-lactic acid) PLA/calcium carbonate composites doped with silver nanoparticles. Current Analytical …, ingentaconnect.com, cited by 3 (3.00 per year)
P Xiu, Z Jia, J Lv, C Yin, Y Cheng, K Zhang, ... (2016) Tailored surface treatment of 3D printed porous Ti6Al4V by microarc oxidation for enhanced osseointegration via optimized bone in-growth patterns and interlocked …. … applied materials & …, ACS Publications, cited by 43 (14.33 per year)
L Ren, X Zhou, Q Liu, Y Liang, Z Song, ... (2018) 3D magnetic printing of bio-inspired composites with tunable mechanical properties. Journal of materials …, Springer, cited by 2 (2.00 per year)
X Le, W Lu, J Zhang, T Chen (2019) Recent Progress in Biomimetic Anisotropic Hydrogel Actuators. Advanced Science, Wiley Online Library, cited by 2 (2.00 per year)
G Zhang (2016) 3D bioprinting nanocomposite scaffolds for complex tissue regeneration. The FASEB Journal, fasebj.org, cited by 2 (0.67 per year)
JL Simon, ED Rekow, VP Thompson, ... (2008) MicroCT analysis of hydroxyapatite bone repair scaffolds created via three-dimensional printing for evaluating the effects of scaffold architecture on bone ingrowth. … Research Part A: An …, Wiley Online Library, cited by 28 (2.55 per year)
E Pei, J Shen, J Watling (2015) Direct 3D printing of polymers onto textiles: experimental studies and applications. Rapid Prototyping Journal, emeraldinsight.com, cited by 64 (16.00 per year)
KM Ferlin, ME Prendergast, ML Miller, DS Kaplan, ... (2016) Influence of 3D printed porous architecture on mesenchymal stem cell enrichment and differentiation. Acta biomaterialia, Elsevier, cited by 37 (12.33 per year)

PS Maher, RP Keatch, K Donnelly, ... (2009) Construction of 3D biological matrices using rapid prototyping technology. Rapid Prototyping …, emeraldinsight.com, cited by 71 (7.10 per year)
P Turner, M Dickinson (2009) Development of a biomimetic robotic bear: Or is a bare bear bearable?. … on Robotics and Biomimetics, ieeexplore.ieee.org, cited by 2 (0.20 per year)
R Mutlu, SK Yildiz, G Alici, ... (2016) Mechanical stiffness augmentation of a 3D printed soft prosthetic finger. 2016 IEEE …, ieeexplore.ieee.org, cited by 7 (2.33 per year)
LJ Pourchet, A Thepot, M Albouy, ... (2017) Human skin 3D bioprinting using scaffold-free approach. Advanced …, Wiley Online Library, cited by 66 (33.00 per year)
Y Lu, G Mapili, G Suhali, S Chen, ... (2006) A digital micro-mirror device-based system for the microfabrication of complex, spatially patterned tissue engineering scaffolds. Journal of Biomedical …, Wiley Online Library, cited by 247 (19.00 per year)
JM Lee, SL Sing, M Zhou, WY Yeong (2018) 3D bioprinting processes: A perspective on classification and terminology., dr.ntu.edu.sg, cited by 10 (10.00 per year)
NRFA Silva, L Witek, PG Coelho, ... (2011) Additive CAD/CAM process for dental prostheses. … Implant, Esthetic and …, Wiley Online Library, cited by 73 (9.13 per year)
B Shih, J Mayeda, Z Huo, ... (2018) 3D printed resistive soft sensors. … Conference on Soft …, ieeexplore.ieee.org, cited by 3 (3.00 per year)
A El-Hajje, EC Kolos, JK Wang, S Maleksaeedi, ... (2014) Physical and mechanical characterisation of 3D-printed porous titanium for biomedical applications. Journal of Materials …, Springer, cited by 55 (11.00 per year)
S Chawla, S Midha, A Sharma, ... (2018) Silk-based bioinks for 3D bioprinting. Advanced healthcare …, Wiley Online Library, cited by 19 (19.00 per year)
F Ruedinger, A Lavrentieva, C Blume, ... (2015) Hydrogels for 3D mammalian cell culture: a starting guide for laboratory practice. Applied microbiology …, Springer, cited by 46 (11.50 per year)
X Chen, J Liu, T Shi, H Qian, K Chen, Q Wei (2012) 3D printing robot: model optimization and image compensation. Journal of Control Theory …, Springer, cited by 6 (0.86 per year)
Z Xia, X Yu, M Wei* (2012) Biomimetic collagen/apatite coating formation on Ti6Al4V substrates. … of Biomedical Materials Research Part B …, Wiley Online Library, cited by 40 (5.71 per year)
JP Vacanti (2018) Systems for and methods for using biomimetic structures providing communication in living tissue. US Patent App. 15/752,508, Google Patents, cited by 2 (2.00 per year)
W Schuurman, PA Levett, MW Pot, ... (2013) Gelatin-methacrylamide hydrogels as potential biomaterials for fabrication of tissue-engineered cartilage constructs. Macromolecular …, Wiley Online Library, cited by 324 (54.00 per year)
GX Gu, M Takaffoli, MJ Buehler (2017) Hierarchically enhanced impact resistance of bioinspired composites. Advanced Materials, Wiley Online Library, cited by 53 (26.50 per year)
E Hachet, H Van den Berghe, E Bayma, ... (2012) Design of biomimetic cell-interactive substrates using hyaluronic acid hydrogels with tunable mechanical properties. …, ACS Publications, cited by 83 (11.86 per year)
LE Murr (2015) Handbook of materials structures, properties, processing and performance., Springer, cited by 32 (8.00 per year)
F Fu, Z Qin, C Xu, X Chen, R Li, L Wang, ... (2017) Magnetic resonance imaging-three-dimensional printing technology fabricates customized scaffolds for brain tissue engineering. Neural regeneration …, ncbi.nlm.nih.gov, cited by 11 (5.50 per year)

J Lin, M Wang, H Hu, X Yang, B Wen, ... (2016) Multimodal-imaging-guided Cancer Phototherapy by Versatile Biomimetic Theranostics with UV and γ-irradiation Protection. Advanced …, Wiley Online Library, cited by 78 (26.00 per year)
CJ Newcomb, R Bitton, YS Velichko, ML Snead, ... (2012) The role of nanoscale architecture in supramolecular templating of biomimetic hydroxyapatite mineralization. Small, Wiley Online Library, cited by 49 (7.00 per year)
P Rastogi, B Kandasubramanian (2019) Breakthrough in the printing tactics for stimuli-responsive materials: 4D printing. Chemical Engineering Journal, Elsevier, cited by 3 (3.00 per year)
R Yadav, R Goud, A Dutta, X Wang, ... (2018) Biomimicking of Hierarchal Molluscan Shell Structure Via Layer by Layer 3D Printing. Industrial & …, ACS Publications, cited by 4 (4.00 per year)
BR Ringeisen, RK Pirlo, PK Wu, T Boland, Y Huang, ... (2013) Cell and organ printing turns 15: diverse research to commercial transitions. MRS bulletin, cambridge.org, cited by 71 (11.83 per year)
D Wu, JN Wang, LG Niu, XL Zhang, ... (2014) Bioinspired Fabrication of High-Quality 3D Artificial Compound Eyes by Voxel-Modulation Femtosecond Laser Writing for Distortion-Free Wide-Field-of-View …. Advanced Optical …, Wiley Online Library, cited by 56 (11.20 per year)
R Mutlu, G Alici, M in het Panhuis, GM Spinks (2016) 3D printed flexure hinges for soft monolithic prosthetic fingers. Soft Robotics, liebertpub.com, cited by 34 (11.33 per year)
MT Tolley, RF Shepherd, B Mosadegh, KC Galloway, ... (2014) A resilient, untethered soft robot. Soft robotics, liebertpub.com, cited by 384 (76.80 per year)
J Najem, B Akle, SA Sarles, DJ Leo (2011) Design and development of a biomimetic jellyfish robot that features ionic polymer metal composites actuators. ASME 2011 Conference on …, researchgate.net, cited by 7 (0.88 per year)
AG Mikos, SW Herring, P Ochareon, J Elisseeff, ... (2006) Engineering complex tissues. Tissue …, liebertpub.com, cited by 517 (39.77 per year)
H Liu, H Zhou, H Lan, T Liu, X Liu, H Yu (2017) 3D printing of artificial blood vessel: study on multi-parameter optimization design for vascular molding effect in alginate and gelatin. Micromachines, mdpi.com, cited by 5 (2.50 per year)
Y Qian, X Zhou, H Sun, J Yang, Y Chen, ... (2018) Biomimetic domain-active electrospun scaffolds facilitating bone regeneration synergistically with antibacterial efficacy for bone defects. … applied materials & …, ACS Publications, cited by 7 (7.00 per year)
Z Zhou, CA Mitchell, FJ Buchanan, ... (2012) Effects of heat treatment on the mechanical and degradation properties of 3D-printed calcium-sulphate-based scaffolds. ISRN …, downloads.hindawi.com, cited by 22 (3.14 per year)
HH Hwang, W Zhu, G Victorine, N Lawrence, ... (2018) 3D-Printing of Functional Biomedical Microdevices via Light-and Extrusion-Based Approaches. Small …, Wiley Online Library, cited by 9 (9.00 per year)
V Dikshit, NA Prasanth, JD Kumar, YL Yap, WY Yeong (2016) Investigation of quasi static indentation on 3D printed honeycomb based truncated-pyramid square structure., dr.ntu.edu.sg, cited by 5 (1.67 per year)
E Jabbari (2011) Bioconjugation of hydrogels for tissue engineering. Current opinion in biotechnology, Elsevier, cited by 67 (8.38 per year)
H Zhao, Y Chen, L Shao, M Xie, J Nie, J Qiu, P Zhao, ... (2018) Airflow-assisted 3D bioprinting of human heterogeneous microspheroidal organoids with microfluidic nozzle. Small, Wiley Online Library, cited by 13 (13.00 per year)

E Sachlos, JT Czernuszka (2003) Making tissue engineering scaffolds work. Review: the application of solid freeform fabrication technology to the production of tissue engineering scaffolds. Eur Cell Mater, pdfs.semanticscholar.org, cited by 1328 (83.00 per year)
K Pataky, T Braschler, A Negro, P Renaud, ... (2012) Microdrop printing of hydrogel bioinks into 3D tissue-like geometries. Advanced …, Wiley Online Library, cited by 158 (22.57 per year)
S Ravindran, Y Song, A George (2009) Development of three-dimensional biomimetic scaffold to study epithelial–mesenchymal interactions. Tissue Engineering Part A, liebertpub.com, cited by 38 (3.80 per year)
S Noh, N Myung, M Park, S Kim, SU Zhang, ... (2018) 3D bioprinting for tissue engineering. … Medicine in Urology, Springer, cited by 2 (2.00 per year)
A Le Duigou, M Castro, R Bevan, N Martin (2016) 3D printing of wood fibre biocomposites: From mechanical to actuation functionality. Materials & Design, Elsevier, cited by 107 (35.67 per year)
Z Jamalpoor, H Mirzadeh, MT Joghataei, ... (2015) Fabrication of cancellous biomimetic chitosan-based nanocomposite scaffolds applying a combinational method for bone tissue engineering. … research Part A, Wiley Online Library, cited by 23 (5.75 per year)
TH Kwok, Y Chen (2017) GDFE: Geometry-Driven Finite Element for Four-Dimensional Printing. Journal of Manufacturing …, … .asmedigitalcollection.asme.org, cited by 3 (1.50 per year)
N Vargas-Alfredo, A Dorronsoro, ... (2017) Antimicrobial 3D porous scaffolds prepared by additive manufacturing and breath figures. … applied materials & …, ACS Publications, cited by 11 (5.50 per year)
GN Bancroft, VI Sikavitsas, AG Mikos (2003) Design of a flow perfusion bioreactor system for bone tissue-engineering applications. Tissue engineering, liebertpub.com, cited by 389 (24.31 per year)
A Wu (2013) Single-action three-dimensional model printing methods. US Patent 8,579,620, Google Patents, cited by 63 (10.50 per year)
AE Loiselle, L Wei, M Faryad, EM Paul, ... (2013) Specific biomimetic hydroxyapatite nanotopographies enhance osteoblastic differentiation and bone graft osteointegration. … Engineering Part A, liebertpub.com, cited by 21 (3.50 per year)
SJ Lee, T Esworthy, S Stake, S Miao, ... (2018) Advances in 3D bioprinting for neural tissue engineering. Advanced …, Wiley Online Library, cited by 6 (6.00 per year)
L Ciocca, F De Crescenzio, M Fantini, ... (2009) CAD/CAM and rapid prototyped scaffold construction for bone regenerative medicine and surgical transfer of virtual planning: a pilot study. … Medical Imaging and …, Elsevier, cited by 139 (13.90 per year)
JI Sasaki, M Hashimoto, S Yamaguchi, Y Itoh, ... (2015) Fabrication of biomimetic bone tissue using mesenchymal stem cell-derived three-dimensional constructs incorporating endothelial cells. PLoS …, journals.plos.org, cited by 16 (4.00 per year)
A Barba, A Diez-Escudero, Y Maazouz, ... (2017) Osteoinduction by foamed and 3D-printed calcium phosphate scaffolds: effect of nanostructure and pore architecture. … applied materials & …, ACS Publications, cited by 31 (15.50 per year)
Z Wang, X Shi, H Huang, C Yao, W Xie, C Huang, ... (2017) Magnetically actuated functional gradient nanocomposites for strong and ultra-durable biomimetic interfaces/surfaces. Materials …, pubs.rsc.org, cited by 6 (3.00 per year)
S Arabnejad, B Johnston, M Tanzer, ... (2017) Fully porous 3D printed titanium femoral stem to reduce stress-shielding following total hip arthroplasty. Journal of Orthopaedic …, Wiley Online Library, cited by 65 (32.50 per year)

JM Lee, WY Yeong (2016) Design and printing strategies in 3D bioprinting of cell-hydrogels: A review. Advanced healthcare materials, Wiley Online Library, cited by 69 (23.00 per year)
X Zhang, CL Pint, MH Lee, BE Schubert, A Jamshidi, ... (2011) Optically-and thermally-responsive programmable materials based on carbon nanotube-hydrogel polymer composites. Nano …, ACS Publications, cited by 319 (39.88 per year)
JC Hu, KA Athanasiou (2006) A self-assembling process in articular cartilage tissue engineering. Tissue engineering, liebertpub.com, cited by 235 (18.08 per year)
E Kennedy, D Fecheyr-Lippens, BK Hsiung, ... (2015) Biomimicry: A path to sustainable innovation. Design Issues, MIT Press, cited by 29 (7.25 per year)
C Fidkowski, MR Kaazempur-Mofrad, ... (2005) Endothelialized microvasculature based on a biodegradable elastomer. Tissue …, liebertpub.com, cited by 343 (24.50 per year)
S Juodkazis (2016) Manufacturing: 3D printed micro-optics. Nature Photonics, nature.com, cited by 18 (6.00 per year)
J Guo, R Zhang, L Zhang, X Cao (2018) 4D printing of robust hydrogels consisted of agarose nanofibers and polyacrylamide. ACS Macro Letters, ACS Publications, cited by 10 (10.00 per year)
A Fischer, S Rommel, A Verl (2015) 3D Printed Objects and Components Enabling Next Generation of True Soft Robotics. Soft Robotics, Springer, cited by 5 (1.25 per year)
AI Neto, PA Levkin, JF Mano (2018) Patterned superhydrophobic surfaces to process and characterize biomaterials and 3D cell culture. Materials Horizons, pubs.rsc.org, cited by 13 (13.00 per year)
JM Sirrine, V Meenakshisundaram, NG Moon, PJ Scott, ... (2018) Functional siloxanes with photo-activated, simultaneous chain extension and crosslinking for lithography-based 3D printing. Polymer, Elsevier, cited by 8 (8.00 per year)
G Stoychev, S Zakharchenko, S Turcaud, ... (2012) Shape-programmed folding of stimuli-responsive polymer bilayers. ACS …, ACS Publications, cited by 179 (25.57 per year)
J Seo, WY Byun, A Frank, ... (2016) Human blinking 'eye-on-a-chip'. Investig …, pdfs.semanticscholar.org, cited by 10 (3.33 per year)
LE Murr, LE Murr (2015) 3D Printing: Printed Electronics. … of Materials Structures, Properties, Processing and …, Springer, cited by 5 (1.25 per year)
SW Bae, KW Lee, JH Park, JH Lee, CR Jung, ... (2018) 3D bioprinted artificial trachea with epithelial cells and chondrogenic-differentiated bone marrow-derived mesenchymal stem cells. International journal of …, mdpi.com, cited by 12 (12.00 per year)
V Rodriguez-Rivera, JW Weidner, MJ Yost (2016) Three-dimensional biomimetic technology: novel biorubber creates defined micro-and macro-scale architectures in collagen hydrogels. JoVE (Journal of Visualized …, jove.com, cited by 4 (1.33 per year)
AA Narkhede, LA Shevde, ... (2017) Biomimetic strategies to recapitulate organ specific microenvironments for studying breast cancer metastasis. International journal of …, Wiley Online Library, cited by 9 (4.50 per year)
R El-Ayoubi, N Eliopoulos, R Diraddo, ... (2008) Design and fabrication of 3D porous scaffolds to facilitate cell-based gene therapy. … Engineering Part A, liebertpub.com, cited by 42 (3.82 per year)
RM Nerem, A Sambanis (1995) Tissue engineering: from biology to biological substitutes. Tissue engineering, liebertpub.com, cited by 421 (17.54 per year)
RP Wilkerson, B Gludovatz, J Watts, ... (2016) A Novel Approach to Developing Biomimetic ("Nacre-Like") Metal-Compliant-Phase (Nickel–Alumina) Ceramics through Coextrusion. Advanced …, Wiley Online Library, cited by 27 (9.00 per year)

V Mironov, N Reis, B Derby (2006) Bioprinting: A beginning. Tissue engineering, liebertpub.com, cited by 244 (18.77 per year)
A Nadernezhad, N Khani, GA Skvortsov, ... (2016) Multifunctional 3D printing of heterogeneous hydrogel structures. Scientific reports, nature.com, cited by 18 (6.00 per year)
L Fan, JL Li, Z Cai, X Wang (2018) Creating biomimetic anisotropic architectures with co-aligned nanofibers and macrochannels by manipulating ice crystallization. ACS nano, ACS Publications, cited by 5 (5.00 per year)
RV Badhe, D Bijukumar, DR Chejara, M Mabrouk, ... (2017) A composite chitosan-gelatin bi-layered, biomimetic macroporous scaffold for blood vessel tissue engineering. Carbohydrate …, Elsevier, cited by 24 (12.00 per year)
A Čučaković, B Jovic, M Komnenov (2016) Biomimetic geometry approach to generative design. Periodica Polytechnica Architecture, pp.bme.hu, cited by 3 (1.00 per year)
B Ward-Cherrier, L Cramphorn, ... (2017) Exploiting sensor symmetry for generalized tactile perception in biomimetic touch. IEEE Robotics and …, ieeexplore.ieee.org, cited by 9 (4.50 per year)
X Chen, J Liu, S Lin, T Shi, K Chen, ... (2011) Model optimization and image compensation in 3D printing. Proceedings of the 30th …, ieeexplore.ieee.org, cited by 3 (0.38 per year)
Y Kim, Y Kim, TI Lee, TS Kim, S Ryu (2018) An extended analytic model for the elastic properties of platelet-staggered composites and its application to 3D printed structures. Composite Structures, Elsevier, cited by 7 (7.00 per year)
A Doraiswamy, TM Dunaway, JJ Wilker, ... (2009) Inkjet printing of bioadhesives. Journal of Biomedical …, Wiley Online Library, cited by 43 (4.30 per year)
SM Chen, HL Gao, YB Zhu, HB Yao, ... (2018) Biomimetic twisted plywood structural materials. National Science …, academic.oup.com, cited by 5 (5.00 per year)
S Raymond, Y Maazouz, EB Montufar, RA Perez, ... (2018) Accelerated hardening of nanotextured 3D-plotted self-setting calcium phosphate inks. Acta biomaterialia, Elsevier, cited by 3 (3.00 per year)
C Vyas, G Poologasundarampillai, J Hoyland, ... (2017) 3D printing of biocomposites for osteochondral tissue engineering. Biomedical …, Elsevier, cited by 3 (1.50 per year)
L Wang, J Lau, EL Thomas, MC Boyce (2011) Co-continuous composite materials for stiffness, strength, and energy dissipation. Advanced Materials, Wiley Online Library, cited by 138 (17.25 per year)
Z Feng, Y Li, L Hao, Y Yang, T Tang, D Tang, ... (2019) Graphene-Reinforced Biodegradable Resin Composites for Stereolithographic 3D Printing of Bone Structure Scaffolds. Journal of …, hindawi.com, cited by 2 (2.00 per year)
A Shapira, DH Kim, T Dvir (2014) Advanced micro-and nanofabrication technologies for tissue engineering. Biofabrication, iopscience.iop.org, cited by 19 (3.80 per year)
KH Cho, MG Song, H Jung, J Park, ... (2016) A robotic finger driven by twisted and coiled polymer actuator. Electroactive …, spiedigitallibrary.org, cited by 48 (16.00 per year)
Z Shen, AGP Kottapalli, V Subramaniam, ... (2016) Biomimetic flow sensors for biomedical flow sensing in intravenous tubes. 2016 IEEE …, ieeexplore.ieee.org, cited by 2 (0.67 per year)
I Paun, R Popescu, B Calin, C Mustaciosu, ... (2018) 3D biomimetic magnetic structures for static magnetic field stimulation of osteogenesis. International journal of …, mdpi.com, cited by 7 (7.00 per year)

WJ Vas, M Shah, R Al Hosni, ... (2017) Biomimetic strategies for fracture repair: Engineering the cell microenvironment for directed tissue formation. Journal of tissue …, journals.sagepub.com, cited by 3 (1.50 per year)
B Holmes, W Zhu, LG Zhang (2014) Development of a novel 3D bioprinted in vitro nano bone model for breast cancer bone metastasis study. MRS Online Proceedings Library …, cambridge.org, cited by 3 (0.60 per year)
X Zhao, S Du, L Chai, X Zhou, L Liu, ... (2015) Anti-cancer drug screening based on a adipose-derived stem cell/hepatocyte 3D printing technique. Journal of Stem …, pdfs.semanticscholar.org, cited by 11 (2.75 per year)
RK Kankala, K Zhu, J Li, CS Wang, SB Wang, ... (2017) Fabrication of arbitrary 3D components in cardiac surgery: from macro-, micro-to nanoscale. …, iopscience.iop.org, cited by 31 (15.50 per year)
NS Moghaddam, R Skoracki, M Miller, M Elahinia, ... (2016) Three dimensional printing of stiffness-tuned, nitinol skeletal fixation hardware with an example of mandibular segmental defect repair. Procedia CIRP, Elsevier, cited by 37 (12.33 per year)
K Tonsomboon, AL Butcher, ML Oyen (2017) Strong and tough nanofibrous hydrogel composites based on biomimetic principles. Materials Science and Engineering …, Elsevier, cited by 27 (13.50 per year)
SE Kim, YP Yun, KS Shim, HJ Kim, K Park, ... (2016) 3D printed alendronate-releasing poly (caprolactone) porous scaffolds enhance osteogenic differentiation and bone formation in rat tibial defects. Biomedical …, iopscience.iop.org, cited by 13 (4.33 per year)
VT Widyaya, EK Riga, C Müller, K Lienkamp (2018) Submicrometer-Sized, 3D Surface-Attached Polymer Networks by Microcontact Printing: Using UV-Cross-Linking Efficiency To Tune Structure Height. Macromolecules, ACS Publications, cited by 2 (2.00 per year)
S Zhang, S Vijayavenkataraman, ... (2019) A review on the use of computational methods to characterize, design, and optimize tissue engineering scaffolds, with a potential in 3D printing fabrication. Journal of Biomedical …, Wiley Online Library, cited by 4 (4.00 per year)
S Naficy, R Gately, R Gorkin III, H Xin, ... (2017) 4D printing of reversible shape morphing hydrogel structures. Macromolecular …, Wiley Online Library, cited by 57 (28.50 per year)
Y Li, X Jiang, L Li, ZN Chen, G Gao, R Yao, ... (2018) 3D printing human induced pluripotent stem cells with novel hydroxypropyl chitin bioink: scalable expansion and uniform aggregation. Biofabrication, iopscience.iop.org, cited by 6 (6.00 per year)
X Li, H Cai, X Cui, P Cao, J Zhang, G Li, ... (2014) Prevention of late postpneumonectomy complications using a 3D printed lung in dog models. European Journal of …, academic.oup.com, cited by 9 (1.80 per year)
E Zizer, D Roppenecker, F Helmes, S Hafner, ... (2016) A new 3D-printed overtube system for endoscopic submucosal dissection: first results of a randomized study in a porcine model. …, thieme-connect.com, cited by 15 (5.00 per year)
S Singare, Y Liu, D Li, B Lu, S He (2008) Individually prefabricated prosthesis for maxilla reconstruction. Journal of Prosthodontics, Wiley Online Library, cited by 56 (5.09 per year)
C Shen, Q Meng, G Zhang (2014) Design of 3D printed insert for hanging culture of Caco-2 cells. Biofabrication, iopscience.iop.org, cited by 7 (1.40 per year)
JFV Vincent (2016) Biomimetics in architectural design. Intelligent Buildings International, Taylor & Francis, cited by 11 (3.67 per year)
P Dadhich, B Das, P Pal, PK Srivas, ... (2016) A simple approach for an eggshell-based 3D-printed osteoinductive multiphasic calcium phosphate scaffold. … applied materials & …, ACS Publications, cited by 21 (7.00 per year)

D Tumey, S Berriman (2017) Skin printing and auto-grafting. US Patent 9,545,302, Google Patents, cited by 5 (2.50 per year)
H Horng, Y Liu, N Suresh, P Ghassemi, J Pfefer, ... (2018) Biomimetic Microvascular Tissue Phantoms Fabricated with Two-Photon 3D Printing. Frontiers in …, osapublishing.org, cited by 1 (1.00 per year)
WJ Li, YJ Jiang, RS Tuan (2006) Chondrocyte phenotype in engineered fibrous matrix is regulated by fiber size. Tissue engineering, liebertpub.com, cited by 240 (18.46 per year)
MJ Kim, MJ Yeo, M Kim, GH Kim (2018) Biomimetic cellulose/calcium-deficient-hydroxyapatite composite scaffolds fabricated using an electric field for bone tissue engineering. RSC advances, pubs.rsc.org, cited by 2 (2.00 per year)
S Vijayavenkataraman (2017) 3D bioprinted skin: the first 'to-be'successful printed organ?., Future Medicine, cited by 2 (1.00 per year)
JH Yang, Z Zhao, SH Park (2015) Evaluation of directional mechanical properties of 3D printed polymer parts. 2015 15th International …, ieeexplore.ieee.org, cited by 3 (0.75 per year)
S Vijayavenkataraman, S Thaharah, S Zhang, ... (2019) 3D-Printed PCL/rGO Conductive Scaffolds for Peripheral Nerve Injury Repair. Artificial …, Wiley Online Library, cited by 3 (3.00 per year)
PDE Baniqued, JR Dungao, ... (2018) Biomimetics in the design of a robotic exoskeleton for upper limb therapy. AIP Conference …, aip.scitation.org, cited by 2 (2.00 per year)
X Pu, G Li, Y Liu (2016) Progress and perspective of studies on biomimetic shark skin drag reduction. ChemBioEng Reviews, Wiley Online Library, cited by 7 (2.33 per year)
BI Oladapo, SA Zahedi, AOM Adeoye (2019) 3D printing of bone scaffolds with hybrid biomaterials. Composites Part B: Engineering, Elsevier, cited by 6 (6.00 per year)
F Simone, A York, S Seelecke (2015) Design and fabrication of a three-finger prosthetic hand using SMA muscle wires. Bioinspiration, Biomimetics …, spiedigitallibrary.org, cited by 17 (4.25 per year)
H Shao, Y He, J Fu, D He, X Yang, J Xie, C Yao, ... (2016) 3D printing magnesium-doped wollastonite/ β -TCP bioceramics scaffolds with high strength and adjustable degradation. Journal of the European …, Elsevier, cited by 45 (15.00 per year)
L Chen, X Tang, P Xie, J Xu, Z Chen, Z Cai, ... (2018) 3D Printing of Artificial Leaf with Tunable Hierarchical Porosity for CO2 Photoreduction. Chemistry of …, ACS Publications, cited by 15 (15.00 per year)
Z Chang, W Liu, J Tong, L Guo, H Xie, X Yang, ... (2016) Design and experiments of biomimetic stubble cutter. Journal of Bionic …, Elsevier, cited by 5 (1.67 per year)
B Mosadegh, P Polygerinos, ... (2014) Pneumatic networks for soft robotics that actuate rapidly. Advanced functional …, Wiley Online Library, cited by 468 (93.60 per year)
WN Zeng, FY Wang, C Chen, Y Zhang, ... (2016) A Novel Dual-Chamber Culture System with Biomimetic Scaffold for Osteochondral Tissue Engineering. … of Biomaterials and …, ingentaconnect.com, cited by 2 (0.67 per year)
CJ Liao, CF Chen, JH Chen, SF Chiang, ... (2002) Fabrication of porous biodegradable polymer scaffolds using a solvent merging/particulate leaching method. … Research: An Official …, Wiley Online Library, cited by 269 (15.82 per year)
B Lei, KH Shin, YH Koh, HE Kim (2014) Porous gelatin–siloxane hybrid scaffolds with biomimetic structure and properties for bone tissue regeneration. Journal of Biomedical …, Wiley Online Library, cited by 24 (4.80 per year)
C Murphy (2016) 3D Printing infill using Biomimetics., cited by 3 (1.00 per year)

F Clavica, X Zhao, M ElMahdy, MJ Drake, X Zhang, ... (2014) Investigating the flow dynamics in the obstructed and stented ureter by means of a biomimetic artificial model. PloS one, journals.plos.org, cited by 20 (4.00 per year)
S Kilper, SJ Facey, Z Burghard, B Hauer, ... (2018) Macroscopic Properties of Biomimetic Ceramics Are Governed by the Molecular Recognition at the Bioorganic–Inorganic Interface. Advanced Functional …, Wiley Online Library, cited by 7 (7.00 per year)
D Chimene, CW Peak, JL Gentry, ... (2018) Nanoengineered ionic–covalent entanglement (NICE) bioinks for 3D bioprinting. … applied materials & …, ACS Publications, cited by 18 (18.00 per year)
D Tumey, S Berriman (2015) Skin printing and auto-grafting. US Patent App. 14/801,893, Google Patents, cited by 6 (1.50 per year)
J Prša, F Irlinger, TC Lueth (2014) Algorithm for detecting and solving the problem of under-Filled pointed ends based on 3D printing plastic droplet generation. ASME 2014 …, … .asmedigitalcollection.asme.org, cited by 4 (0.80 per year)
T Oner, IF Cengiz, M Pitikakis, L Cesario, ... (2017) 3D segmentation of intervertebral discs: from concept to the fabrication of patient-specific scaffolds. Journal of 3D printing …, Future Medicine, cited by 7 (3.50 per year)
L Zhang, S Chizhik, Y Wen, ... (2016) Directed motility of hygroresponsive biomimetic actuators. Advanced Functional …, Wiley Online Library, cited by 36 (12.00 per year)
P Sander (2015) Additive Layer Manufacturing. Airbus technical magazine-FAST, sfa-am.ch, cited by 7 (1.75 per year)
JS Kim, S Hong, C Hwang (2016) Bio-ink Materials for 3D Bio-printing. Journal of International Society for …, researchgate.net, cited by 4 (1.33 per year)
Z Jia, L Wang (2018) 3D Printing of Biomimetic Composites with Improved Fracture Toughness. Available at SSRN 3300049, papers.ssrn.com, cited by 1 (1.00 per year)
A Nishiguchi, A Mourran, H Zhang, ... (2018) In-Gel direct laser writing for 3D-Designed hydrogel composites that undergo complex self-shaping. Advanced …, Wiley Online Library, cited by 8 (8.00 per year)
E Blasco, J Müller, P Müller, V Trouillet, ... (2016) Fabrication of Conductive 3D Gold-Containing Microstructures via Direct Laser Writing. Advanced …, Wiley Online Library, cited by 45 (15.00 per year)
HJ Lee, YB Kim, SH Ahn, JS Lee, ... (2015) A new approach for fabricating collagen/ECM-based bioinks using preosteoblasts and human adipose stem cells. Advanced …, Wiley Online Library, cited by 39 (9.75 per year)
X Zhou, T Li, J Wang, F Chen, D Zhou, ... (2018) Mechanochemical regulated origami with tough hydrogels by ion transfer printing. … applied materials & …, ACS Publications, cited by 16 (16.00 per year)
E Jabbari, DN Rocheleau, W Xu, ... (2007) Fabrication of biomimetic scaffolds with well-defined pore geometry by fused deposition modeling. ASME 2007 …, … .asmedigitalcollection.asme.org, cited by 3 (0.25 per year)
C Yue, S Guo, L Shi (2016) Design and performance evaluation of a biomimetic microrobot for the father–son underwater intervention robotic system. Microsystem Technologies, Springer, cited by 20 (6.67 per year)
K Arai, T Yoshida, M Okabe, M Goto, ... (2017) Fabrication of 3D-culture platform with sandwich architecture for preserving liver-specific functions of hepatocytes using 3D bioprinter. … Research Part A, Wiley Online Library, cited by 7 (3.50 per year)

M Lee, BM Wu, JCY Dunn (2008) Effect of scaffold architecture and pore size on smooth muscle cell growth. … Research Part A: An Official Journal …, Wiley Online Library, cited by 109 (9.91 per year)
Y Luo, MS Shoichet (2004) A photolabile hydrogel for guided three-dimensional cell growth and migration. Nature materials, nature.com, cited by 705 (47.00 per year)
T Serra, C Capelli, R Toumpaniari, IR Orriss, ... (2016) Design and fabrication of 3D-printed anatomically shaped lumbar cage for intervertebral disc (IVD) degeneration treatment. …, iopscience.iop.org, cited by 22 (7.33 per year)
R Domingo-Roca, B Tiller, JC Jackson, ... (2018) Bio-inspired 3D-printed piezoelectric device for acoustic frequency selection. Sensors and Actuators A …, Elsevier, cited by 4 (4.00 per year)
F Momeni, J Ni (2018) Nature-inspired smart solar concentrators by 4D printing. Renewable energy, Elsevier, cited by 7 (7.00 per year)
TL Nguyen, SJ Allen, SJ Phee (2013) Exploiting 3D printing technology to develop robotic running foot for footwear testing: This paper describes a framework for a prosthesis that has four controlled …. Virtual and Physical Prototyping, Taylor & Francis, cited by 5 (0.83 per year)
JP Temple, DL Hutton, BP Hung, ... (2014) Engineering anatomically shaped vascularized bone grafts with hASCs and 3D-printed PCL scaffolds. … research Part A, Wiley Online Library, cited by 141 (28.20 per year)
W Xu, X Zhang, P Yang, O Långvik, ... (2019) Surface engineered biomimetic inks based on uv cross-linkable wood biopolymers for 3D printing. … applied materials & …, ACS Publications, cited by 1 (1.00 per year)
J He, W Zhang, Y Liu, X Li, D Li, Z Jin (2015) Design and fabrication of biomimetic multiphased scaffolds for ligament-to-bone fixation. Materials science and engineering …, Elsevier, cited by 14 (3.50 per year)
M Lopez, R Rubio, S Martín, ... (2015) Active materials for adaptive architectural envelopes based on plant adaptation principles. Journal of Facade …, content.iospress.com, cited by 28 (7.00 per year)
P Gupta, M Adhikary, M Kumar, ... (2016) Biomimetic, osteoconductive non-mulberry silk fiber reinforced tricomposite scaffolds for bone tissue engineering. … applied materials & …, ACS Publications, cited by 32 (10.67 per year)
AP Napolitano, DM Dean, AJ Man, J Youssef, ... (2007) Scaffold-free three-dimensional cell culture utilizing micromolded nonadhesive hydrogels. …, Future Science, cited by 163 (13.58 per year)
JD Carrico, JM Erickson, ... (2016) Characterization of 3D-printed IPMC actuators. … Polymer Actuators and …, spiedigitallibrary.org, cited by 3 (1.00 per year)
J Lee, MJ Cuddihy, NA Kotov (2008) Three-dimensional cell culture matrices: state of the art. Tissue Engineering Part B: Reviews, liebertpub.com, cited by 832 (75.64 per year)
Y Hao, Z Gong, Z Xie, S Guan, X Yang, ... (2016) Universal soft pneumatic robotic gripper with variable effective length. 2016 35th Chinese …, ieeexplore.ieee.org, cited by 35 (11.67 per year)
Z Izadifar, T Chang, W Kulyk, X Chen, ... (2015) Analyzing biological performance of 3D-printed, cell-impregnated hybrid constructs for cartilage tissue engineering. … Engineering Part C …, liebertpub.com, cited by 53 (13.25 per year)
JR Jones, LL Hench (2003) Regeneration of trabecular bone using porous ceramics. Current Opinion in Solid State and Materials Science, Elsevier, cited by 314 (19.63 per year)

R Oftadeh, B Haghpanah, D Vella, A Boudaoud, ... (2014) Optimal fractal-like hierarchical honeycombs. Physical review …, APS, cited by 67 (13.40 per year)
A Khademhosseini, R Langer (2016) A decade of progress in tissue engineering. Nature protocols, nature.com, cited by 156 (52.00 per year)
JEM Teoh, CK Chua, Y Liu, J An (2017) 4D printing of customised smart sunshade. Challenges for Technology …, ingentaconnect.com, cited by 4 (2.00 per year)
Y Qian, H Chen, Y Xu, J Yang, X Zhou, ... (2016) The preosteoblast response of electrospinning PLGA/PCL nanofibers: Effects of biomimetic architecture and collagen I. International journal …, ncbi.nlm.nih.gov, cited by 15 (5.00 per year)
SH Jariwala, GS Lewis, ZJ Bushman, ... (2015) 3D printing of personalized artificial bone scaffolds. 3D printing and …, liebertpub.com, cited by 32 (8.00 per year)
Y Yu, S Hua, M Yang, Z Fu, S Teng, K Niu, Q Zhao, ... (2016) Fabrication and characterization of electrospinning/3D printing bone tissue engineering scaffold. RSC Advances, pubs.rsc.org, cited by 22 (7.33 per year)
D Rana, TS Kumar, ... (2017) Impact of nanotechnology on 3D bioprinting. Journal of Bionanoscience, ingentaconnect.com, cited by 3 (1.50 per year)
J Baek, X Chen, S Sovani, S Jin, ... (2015) Meniscus tissue engineering using a novel combination of electrospun scaffolds and human meniscus cells embedded within an extracellular matrix hydrogel. Journal of …, Wiley Online Library, cited by 48 (12.00 per year)
C Yue, S Guo, M Li, Y Li (2015) Characteristics evaluation of a biomimetic microrobot for a father-son underwater intervention robotic system. 2015 IEEE/RSJ International …, ieeexplore.ieee.org, cited by 5 (1.25 per year)
JH Shim, JY Kim, M Park, J Park, DW Cho (2011) Development of a hybrid scaffold with synthetic biomaterials and hydrogel using solid freeform fabrication technology. Biofabrication, iopscience.iop.org, cited by 162 (20.25 per year)
S Barui, S Chatterjee, S Mandal, A Kumar, ... (2017) Microstructure and compression properties of 3D powder printed Ti-6Al-4V scaffolds with designed porosity: Experimental and computational analysis. Materials Science and …, Elsevier, cited by 32 (16.00 per year)
AD Marchese, CD Onal, D Rus (2014) Autonomous soft robotic fish capable of escape maneuvers using fluidic elastomer actuators. Soft Robotics, liebertpub.com, cited by 335 (67.00 per year)
YS Cho, S Choi, SH Lee, KK Kim, YS Cho (2019) … /hydroxyapatite composite scaffold with enhanced biomimetic mineralization by exposure to hydroxyapatite via a 3D-printing system and alkaline erosion. European Polymer Journal, Elsevier, cited by 1 (1.00 per year)
LE Freed, F Guilak, XE Guo, ML Gray, ... (2006) Advanced tools for tissue engineering: scaffolds, bioreactors, and signaling. Tissue …, liebertpub.com, cited by 290 (22.31 per year)
H Stratesteffen, M Köpf, F Kreimendahl, A Blaeser, ... (2017) GelMA-collagen blends enable drop-on-demand 3D printablility and promote angiogenesis. …, iopscience.iop.org, cited by 24 (12.00 per year)
J Aizenberg, P Fratzl (2013) New materials through bioinspiration and nanoscience. Advanced Functional …, aizenberglab.seas.harvard.edu, cited by 19 (3.17 per year)
JK Min, B Mosadegh, S Dunham, SJ Al'Aref (2018) 3D Printing Applications in Cardiovascular Medicine., books.google.com, cited by 2 (2.00 per year)
N Mohan, J Wilson, D Joseph, ... (2015) Biomimetic fiber assembled gradient hydrogel to engineer glycosaminoglycan enriched and mineralized cartilage: An in vitro study. Journal of Biomedical …, Wiley Online Library, cited by 10 (2.50 per year)

CJ Hansen, R Saksena, DB Kolesky, ... (2013) High-Throughput Printing via Microvascular Multinozzle Arrays. Advanced …, Wiley Online Library, cited by 83 (13.83 per year)
A Dzian, J Živčák, R Penciak, ... (2018) Implantation of a 3D-printed titanium sternum in a patient with a sternal tumor. World journal of …, wjso.biomedcentral.com, cited by 3 (3.00 per year)
GD Nicodemus, SJ Bryant (2008) Cell encapsulation in biodegradable hydrogels for tissue engineering applications. Tissue Engineering Part B: Reviews, liebertpub.com, cited by 906 (82.36 per year)
G Zurlo, L Truskinovsky (2017) Printing non-euclidean solids. Physical review letters, APS, cited by 8 (4.00 per year)
A Stroud, M Morris, K Carey, JC Williams, ... (2013) MU-L8: The design architecture and 3D printing of a Teen-Sized humanoid soccer robot. 8th Workshop on …, ais.uni-bonn.de, cited by 10 (1.67 per year)
M Neufurth, X Wang, S Wang, R Steffen, M Ackermann, ... (2017) 3D printing of hybrid biomaterials for bone tissue engineering: Calcium-polyphosphate microparticles encapsulated by polycaprolactone. Acta biomaterialia, Elsevier, cited by 19 (9.50 per year)
Z Wang, JK Min, G Xiong (2015) Robotics-driven printing of curved 3D structures for manufacturing cardiac therapeutic devices. … on Robotics and Biomimetics …, ieeexplore.ieee.org, cited by 3 (0.75 per year)
Y Jiang, Y Li (2018) Novel 3D-Printed Hybrid Auxetic Mechanical Metamaterial with Chirality-Induced Sequential Cell Opening Mechanisms. Advanced Engineering Materials, Wiley Online Library, cited by 13 (13.00 per year)
S Reitelshöfer, M Göttler, P Schmidt, ... (2016) Aerosol-jet-printing silicone layers and electrodes for stacked dielectric elastomer actuators in one processing device. Electroactive …, spiedigitallibrary.org, cited by 15 (5.00 per year)
Y Martens, A Ehrmann (2017) Composites of 3D-printed polymers and textile fabrics. IOP Conference Series: Materials …, iopscience.iop.org, cited by 9 (4.50 per year)
ET Roche, R Wohlfarth, JTB Overvelde, ... (2014) A bioinspired soft actuated material. Advanced …, Wiley Online Library, cited by 124 (24.80 per year)
F Libonati, C Colombo, L Vergani (2014) Design and characterization of a biomimetic composite inspired to human bone. Fatigue & Fracture of …, Wiley Online Library, cited by 19 (3.80 per year)
S Bose, D Banerjee, A Shivaram, S Tarafder, ... (2018) Calcium phosphate coated 3D printed porous titanium with nanoscale surface modification for orthopedic and dental applications. Materials & Design, Elsevier, cited by 11 (11.00 per year)
Z Jia, Y Yu, S Hou, L Wang (2019) Biomimetic architected materials with improved dynamic performance. Journal of the Mechanics and Physics of Solids, Elsevier, cited by 2 (2.00 per year)
AJ Salgado, OP Coutinho, ... (2004) Bone tissue engineering: state of the art and future trends. Macromolecular bioscience, Wiley Online Library, cited by 1558 (103.87 per year)
G Haghiashtiani, E Habtour, SH Park, F Gardea, ... (2018) 3D printed electrically-driven soft actuators. Extreme Mechanics …, Elsevier, cited by 9 (9.00 per year)
L Lao, H Tan, Y Wang, C Gao (2008) Chitosan modified poly (L-lactide) microspheres as cell microcarriers for cartilage tissue engineering. Colloids and Surfaces B: Biointerfaces, Elsevier, cited by 111 (10.09 per year)
S Wei, G Qu, G Luo, Y Huang, H Zhang, ... (2018) Scalable and Automated Fabrication of Conductive Tough-Hydrogel Microfibers with Ultrastretchability, 3D Printability, and Stress Sensitivity. … applied materials & …, ACS Publications, cited by 7 (7.00 per year)

S Knowlton, A Joshi, B Yenilmez, IT Ozbolat, ... (2016) Advancing cancer research using bioprinting for tumor-on-a-chip platforms. Int J …, tasoglulab.net, cited by 19 (6.33 per year)
A Wang, Q Wang, D Li, Y Tao, X Li (2009) Relationship between stereo depth and parallax image captured in stereoscopic display. Optics and precision …, en.cnki.com.cn, cited by 24 (2.40 per year)
G Gao, T Yonezawa, K Hubbell, G Dai, ... (2015) Inkjet-bioprinted acrylated peptides and PEG hydrogel with human mesenchymal stem cells promote robust bone and cartilage formation with minimal printhead …. Biotechnology …, Wiley Online Library, cited by 122 (30.50 per year)
A Liu, M Sun, X Yang, C Ma, Y Liu, ... (2016) Three-dimensional printing akermanite porous scaffolds for load-bearing bone defect repair: An investigation of osteogenic capability and mechanical evolution. Journal of …, journals.sagepub.com, cited by 10 (3.33 per year)
XY Yin, Y Zhang, X Cai, Q Guo, J Yang, ZL Wang (2019) 3D printing of ionic conductors for high-sensitivity wearable sensors. Materials Horizons, pubs.rsc.org, cited by 3 (3.00 per year)
P Song, C Zhou, H Fan, B Zhang, X Pei, Y Fan, ... (2018) Novel 3D porous biocomposite scaffolds fabricated by fused deposition modeling and gas foaming combined technology. Composites Part B …, Elsevier, cited by 7 (7.00 per year)
J Prša, J Schwaiger, F Irlinger, ... (2013) Dense 3D-packing algorithm for filling the offset contours of a new printing process based on 3D plastic droplet generation. … and Biomimetics (ROBIO …, ieeexplore.ieee.org, cited by 5 (0.83 per year)
J Rouwkema, JD Boer, CAV Blitterswijk (2006) Endothelial cells assemble into a 3-dimensional prevascular network in a bone tissue engineering construct. Tissue engineering, liebertpub.com, cited by 302 (23.23 per year)
F Marga, A Neagu, I Kosztin, ... (2007) Developmental biology and tissue engineering. Birth Defects Research …, Wiley Online Library, cited by 98 (8.17 per year)
G Forgacs, W Sun (2013) Biofabrication: micro-and nano-fabrication, printing, patterning and assemblies., books.google.com, cited by 12 (2.00 per year)
B Müller, H Deyhle, FC Fierz, SH Irsen, ... (2009) Bio-mimetic hollow scaffolds for long bone replacement. Biomimetics and …, spiedigitallibrary.org, cited by 14 (1.40 per year)
P Lichte, HC Pape, T Pufe, P Kobbe, H Fischer (2011) Scaffolds for bone healing: concepts, materials and evidence. Injury, Elsevier, cited by 220 (27.50 per year)
AD Lantada, S Hengsbach, K Bade (2017) Lotus-on-chip: computer-aided design and 3D direct laser writing of bioinspired surfaces for controlling the wettability of materials and devices. Bioinspiration & biomimetics, iopscience.iop.org, cited by 5 (2.50 per year)
JA Phillippi, E Miller, L Weiss, J Huard, ... (2008) Microenvironments engineered by inkjet bioprinting spatially direct adult stem cells toward muscle-and bone-like subpopulations. Stem …, Wiley Online Library, cited by 251 (22.82 per year)
C Dawson, JFV Vincent, AM Rocca (1997) How pine cones open. Nature, nature.com, cited by 297 (13.50 per year)
CM Smith, AL Stone, RL Parkhill, RL Stewart, ... (2004) Three-dimensional bioassembly tool for generating viable tissue-engineered constructs. Tissue …, liebertpub.com, cited by 267 (17.80 per year)
G Zhao, ZZ Sun, LL Li, Y Ge (2016) Fabrication of bionic linear actuator and application study based on 3d printing. Journal of Biomimetics, Biomaterials …, Trans Tech Publ, cited by 1 (0.33 per year)

JM Walker, E Bodamer, O Krebs, Y Luo, ... (2017) Effect of chemical and physical properties on the in vitro degradation of 3D printed high resolution poly (propylene fumarate) scaffolds. …, ACS Publications, cited by 19 (9.50 per year)
X Pu, G Li, H Huang (2016) Preparation, anti-biofouling and drag-reduction properties of a biomimetic shark skin surface. Biology open, bio.biologists.org, cited by 21 (7.00 per year)
X Jing, Z Guo (2018) Biomimetic super durable and stable surfaces with superhydrophobicity. Journal of Materials Chemistry A, pubs.rsc.org, cited by 19 (19.00 per year)
S Kaihara, J Borenstein, R Koka, S Lalan, ... (2000) Silicon micromachining to tissue engineer branched vascular channels for liver fabrication. Tissue …, liebertpub.com, cited by 365 (19.21 per year)
VM Čolić-Damjanovic, ... (2016) Potentials of fablabs for biomimetic architectural research. 2016 International …, ieeexplore.ieee.org, cited by 1 (0.33 per year)
S Trabia, Z Olsen, KJ Kim (2017) Searching for a new ionomer for 3D printable ionic polymer–metal composites: Aquivion as a candidate. Smart Materials and Structures, iopscience.iop.org, cited by 7 (3.50 per year)
CB Ahn, Y Kim, SJ Park, Y Hwang, ... (2018) Development of arginine-glycine-aspartate-immobilized 3D printed poly (propylene fumarate) scaffolds for cartilage tissue engineering. Journal of Biomaterials …, Taylor & Francis, cited by 6 (6.00 per year)
C Credi, G Griffini, M Levi, S Turri (2018) Biotinylated Photopolymers for 3D-Printed Unibody Lab-on-a-Chip Optical Platforms. Small, Wiley Online Library, cited by 4 (4.00 per year)
PA Wieringa, AR Gonçalves de Pinho, ... (2018) Biomimetic architectures for peripheral nerve repair: a review of biofabrication strategies. Advanced …, Wiley Online Library, cited by 9 (9.00 per year)
H Zheng, S SCHLEICHER (2018) Bio-Inspired 3D Printing Experiments. CAADRIA 2018, researchgate.net, cited by 1 (1.00 per year)
D Gendreau, A Mohand-Ousaid, ... (2016) 3D-Printing: A promising technology to design three-dimensional microsystems. … and Robotics at …, ieeexplore.ieee.org, cited by 4 (1.33 per year)
B Guillotin, S Catros, F Guillemot (2013) Laser assisted bio-printing (LAB) of cells and bio-materials based on laser induced forward transfer (LIFT). Laser Technology in Biomimetics, Springer, cited by 3 (0.50 per year)
X Huang, S Lin, Y Hu, Y Liao, W Wang, ... (2018) Preparation and characterization of digital coral hydroxyapatite artificial bone scaffolds based on 3D printing. … of Biomaterials and …, ingentaconnect.com, cited by 4 (4.00 per year)
JY Park, G Gao, J Jang, DW Cho (2016) 3D printed structures for delivery of biomolecules and cells: tissue repair and regeneration. Journal of Materials Chemistry B, pubs.rsc.org, cited by 20 (6.67 per year)
W Yuan, Y Yao, L Keer, Y Jiao, J Yu, Q Li, ... (2019) 3D-printed biomimetic surface structures with abnormal friction properties. Extreme Mechanics …, Elsevier, cited by 1 (1.00 per year)
L Badarnah (2017) Form follows environment: biomimetic approaches to building envelope design for environmental adaptation. Buildings, mdpi.com, cited by 8 (4.00 per year)
F Barrere, CM Van Der Valk, ... (2003) In vitro and in vivo degradation of biomimetic octacalcium phosphate and carbonate apatite coatings on titanium implants. … Research Part A: An …, Wiley Online Library, cited by 177 (11.06 per year)
WG Bae, HN Kim, D Kim, SH Park, ... (2014) 25th anniversary article: scalable multiscale patterned structures inspired by nature: the role of hierarchy. Advanced …, Wiley Online Library, cited by 119 (23.80 per year)

Y Wang, S Wu, MA Kuss, PN Streubel, ... (2017) Effects of hydroxyapatite and hypoxia on chondrogenesis and hypertrophy in 3D bioprinted ADMSC laden constructs. … Biomaterials Science & …, ACS Publications, cited by 9 (4.50 per year)
G Huang, Y Mei (2018) Assembly and Self-Assembly of Nanomembrane Materials—From 2D to 3D. Small, Wiley Online Library, cited by 13 (13.00 per year)
S Pan, S Guo, L Shi, Y He, Z Wang, ... (2014) A spherical robot based on all programmable SoC and 3-D printing. 2014 IEEE International …, ieeexplore.ieee.org, cited by 20 (4.00 per year)
J Li, Y Hsu, E Luo, A Khadka, J Hu (2011) Computer-aided design and manufacturing and rapid prototyped nanoscale hydroxyapatite/polyamide (n-HA/PA) construction for condylar defect caused by …. Aesthetic plastic surgery, Springer, cited by 56 (7.00 per year)
K Turksen (2015) Bioprinting in regenerative medicine., Springer, cited by 15 (3.75 per year)
J Li, M Chen, X Wei, Y Hao, J Wang (2017) Evaluation of 3D-printed polycaprolactone scaffolds coated with freeze-dried platelet-rich plasma for bone regeneration. Materials, mdpi.com, cited by 17 (8.50 per year)
OD Yirmibesoglu, J Morrow, S Walker, ... (2018) Direct 3D printing of silicone elastomer soft robots and their performance comparison with molded counterparts. … Conference on Soft …, ieeexplore.ieee.org, cited by 5 (5.00 per year)
B Zhang, J He, X Li, F Xu, D Li (2016) Micro/nanoscale electrohydrodynamic printing: From 2D to 3D. Nanoscale, pubs.rsc.org, cited by 40 (13.33 per year)
AR Studart (2016) Additive manufacturing of biologically-inspired materials. Chemical Society Reviews, pubs.rsc.org, cited by 141 (47.00 per year)
J Chang, PI Tsai, M Kuo, JS Sun, SY Chen, HH Shen (2019) Augmentation of DMLS Biomimetic Dental Implants with Weight-Bearing Strut to Balance of Biologic and Mechanical Demands: From Bench to Animal. Materials, mdpi.com, cited by 2 (2.00 per year)
X Cui, K Breitenkamp, MG Finn, M Lotz, ... (2012) Direct human cartilage repair using three-dimensional bioprinting technology. … Engineering Part A, liebertpub.com, cited by 361 (51.57 per year)
NA Yaraghi, N Guarín-Zapata, ... (2016) A sinusoidally architected helicoidal biocomposite. Advanced …, Wiley Online Library, cited by 40 (13.33 per year)
A Kosik-Kozioł, M Costantini, T Bolek, K Szöke, ... (2017) PLA short sub-micron fiber reinforcement of 3D bioprinted alginate constructs for cartilage regeneration. …, iopscience.iop.org, cited by 11 (5.50 per year)
Y Ma, L Xie, B Yang, W Tian (2019) Three-dimensional printing biotechnology for the regeneration of the tooth and tooth-supporting tissues. Biotechnology and …, Wiley Online Library, cited by 2 (2.00 per year)
C Li, A Faulkner-Jones, AR Dun, J Jin, ... (2015) Rapid Formation of a Supramolecular Polypeptide–DNA Hydrogel for In Situ Three-Dimensional Multilayer Bioprinting. Angewandte Chemie …, Wiley Online Library, cited by 153 (38.25 per year)
C Deng, Q Yao, C Feng, J Li, L Wang, ... (2017) 3D printing of bilineage constructive biomaterials for bone and cartilage regeneration. Advanced Functional …, Wiley Online Library, cited by 28 (14.00 per year)
M Zhou, Y Tao, L Cheng, W Liu, X Fu (2013) A biomimetic earthworm-like micro robot using nut-type piezoelectric motor. International Conference on …, Springer, cited by 5 (0.83 per year)

AF Girão, Â Semitela, G Ramalho, A Completo, ... (2018) Mimicking nature: Fabrication of 3D anisotropic electrospun polycaprolactone scaffolds for cartilage tissue engineering applications. Composites Part B …, Elsevier, cited by 4 (4.00 per year)
X Chen, JK Possel, C Wacongne, AF Van Ham, ... (2017) 3D printing and modelling of customized implants and surgical guides for non-human primates. Journal of neuroscience …, Elsevier, cited by 19 (9.50 per year)
J Cai, A Vanhorn, C Mullikin, ... (2015) 4D printing of soft robotic facial muscles. 26th international …, sffsymposium.engr.utexas.edu, cited by 4 (1.00 per year)
IT Ozbolat (2016) 3D Bioprinting: fundamentals, principles and applications., books.google.com, cited by 24 (8.00 per year)
S Midha, S Ghosh (2017) Silk-based bioinks for 3D bioprinting. Regenerative Medicine: Laboratory to Clinic, Springer, cited by 4 (2.00 per year)
Y Holovenko, M Antonov, L Kollo, ... (2018) Friction studies of metal surfaces with various 3D printed patterns tested in dry sliding conditions. Proceedings of the …, journals.sagepub.com, cited by 7 (7.00 per year)
BK Hsiung, RH Siddique, L Jiang, Y Liu, ... (2017) Tarantula-Inspired Noniridescent Photonics with Long-Range Order. Advanced Optical …, Wiley Online Library, cited by 19 (9.50 per year)
B Kaehr, JB Shear (2007) Mask-directed multiphoton lithography. Journal of the American Chemical Society, ACS Publications, cited by 72 (6.00 per year)
B Holmes, W Zhu, J Li, JD Lee, ... (2014) Development of novel three-dimensional printed scaffolds for osteochondral regeneration. Tissue Engineering Part …, liebertpub.com, cited by 55 (11.00 per year)
Y Takeoka, K Matsumoto, D Taniguchi, T Tsuchiya, ... (2019) Regeneration of esophagus using a scaffold-free biomimetic structure created with bio-three-dimensional printing. PloS one, journals.plos.org, cited by 1 (1.00 per year)
JY Yoon, GW Kim (2019) Harnessing the bilinear nonlinearity of a 3D printed biomimetic diaphragm for acoustic sensor applications. Mechanical Systems and Signal Processing, Elsevier, cited by 1 (1.00 per year)
H Kakisawa, T Sumitomo, R Inoue, Y Kagawa (2010) Fabrication of nature-inspired bulk laminar composites by a powder processing. Composites Science and …, Elsevier, cited by 24 (2.67 per year)
F Cheng, X Cao, H Li, T Liu, X Xie, D Huang, ... (2019) Generation of Cost-Effective Paper-Based Tissue Models through Matrix-Assisted Sacrificial 3D Printing. Nano …, ACS Publications, cited by 1 (1.00 per year)
LG Griffith (2002) Emerging design principles in biomaterials and scaffolds for tissue engineering. Annals of the New York Academy of Sciences, Wiley Online Library, cited by 481 (28.29 per year)
I Buj-Corral, A Bagheri, O Petit-Rojo (2018) 3D printing of porous scaffolds with controlled porosity and pore size values. Materials, mdpi.com, cited by 3 (3.00 per year)
I Kuru, H Maier, M Müller, T Lenarz, TC Lueth (2016) A 3D-printed functioning anatomical human middle ear model. Hearing research, Elsevier, cited by 16 (5.33 per year)
JD Carrico, NW Traeden, ... (2015) Fused filament additive manufacturing of ionic polymer-metal composite soft active 3D structures. ASME 2015 …, … .asmedigitalcollection.asme.org, cited by 5 (1.25 per year)
Q Fu, E Saiz, AP Tomsia (2011) Bioinspired strong and highly porous glass scaffolds. Advanced functional materials, Wiley Online Library, cited by 184 (23.00 per year)

A Basu, A Saha, C Goodman, ... (2017) Catalytically Initiated Gel-in-Gel Printing of Composite Hydrogels. … applied materials & …, ACS Publications, cited by 10 (5.00 per year)
O Elliott, S Gray, M McClay, B Nassief, ... (2017) Design and Manufacturing of High Surface Area 3D-Printed Media for Moving Bed Bioreactors for Wastewater Treatment. … Water Research & …, Wiley Online Library, cited by 8 (4.00 per year)
MS Alam, A Akhtar, I Ahsan, ... (2018) Pharmaceutical product development exploiting 3D printing technology: Conventional to novel drug delivery system. Current pharmaceutical …, ingentaconnect.com, cited by 1 (1.00 per year)
T Li, G Li, Y Liang, T Cheng, J Dai, ... (2017) Fast-moving soft electronic fish. Science …, advances.sciencemag.org, cited by 115 (57.50 per year)
C Lee, M Kim, YJ Kim, N Hong, S Ryu, HJ Kim, ... (2017) Soft robot review. International Journal of …, Springer, cited by 45 (22.50 per year)
K Pusch, TJ Hinton, AW Feinberg (2018) Large volume syringe pump extruder for desktop 3D printers. HardwareX, Elsevier, cited by 7 (7.00 per year)
JB Costa, J Silva-Correia, JM Oliveira, ... (2017) Fast Setting Silk Fibroin Bioink for Bioprinting of Patient-Specific Memory-Shape Implants. Advanced healthcare …, Wiley Online Library, cited by 10 (5.00 per year)
MA Bohl, R Mauria, JJ Zhou, MA Mooney, ... (2019) The Barrow Biomimetic Spine: face, content, and construct validity of a 3D-printed spine model for freehand and minimally invasive pedicle screw insertion. Global Spine …, journals.sagepub.com, cited by 1 (1.00 per year)
RC Nordberg, EG Loboa (2015) Our fat future: translating adipose stem cell therapy. Stem cells translational medicine, Wiley Online Library, cited by 36 (9.00 per year)
Z Xu, S Kolev, E Todorov (2014) Design, optimization, calibration, and a case study of a 3D-printed, low-cost fingertip sensor for robotic manipulation. 2014 IEEE International Conference …, ieeexplore.ieee.org, cited by 12 (2.40 per year)
IC Liao, FT Moutos, BT Estes, X Zhao, ... (2013) Composite three-dimensional woven scaffolds with interpenetrating network hydrogels to create functional synthetic articular cartilage. Advanced functional …, Wiley Online Library, cited by 131 (21.83 per year)
C Yu, X Ma, W Zhu, P Wang, KL Miller, J Stupin, ... (2019) Scanningless and continuous 3D bioprinting of human tissues with decellularized extracellular matrix. Biomaterials, Elsevier, cited by 5 (5.00 per year)
J Nam, Y Huang, S Agarwal, J Lannutti (2007) Improved cellular infiltration in electrospun fiber via engineered porosity. Tissue engineering, liebertpub.com, cited by 353 (29.42 per year)
T Dutta Roy, JL Simon, JL Ricci, ... (2003) Performance of hydroxyapatite bone repair scaffolds created via three-dimensional fabrication techniques. … Research Part A: An …, Wiley Online Library, cited by 140 (8.75 per year)
Z Wang, H Kumar, Z Tian, X Jin, ... (2018) Visible light photoinitiation of cell-adhesive gelatin methacryloyl hydrogels for stereolithography 3D bioprinting. … applied materials & …, ACS Publications, cited by 9 (9.00 per year)
WT Navaraj, H Nassar, R Dahiya (2019) Prosthetic Hand with Biomimetic Tactile Sensing and Force Feedback. 2019 IEEE International …, ieeexplore.ieee.org, cited by 1 (1.00 per year)
PJ Bártolo, HA Almeida, RA Rezende, T Laoui, ... (2008) Advanced processes to fabricate scaffolds for tissue engineering. Virtual prototyping & bio …, Springer, cited by 58 (5.27 per year)

I Bankole, S Oladapo, AOM Adeoye, SA Zahedi (2018) 3D printing of bone scaffolds with hybrid biomaterials., dora.dmu.ac.uk, cited by 1 (1.00 per year)
D Choudhury, S Anand, MW Naing (2018) The arrival of commercial bioprinters–Towards 3D bioprinting revolution. Int J Bioprint, researchgate.net, cited by 6 (6.00 per year)
G Gao, AF Schilling, T Yonezawa, J Wang, ... (2014) Bioactive nanoparticles stimulate bone tissue formation in bioprinted three-dimensional scaffold and human mesenchymal stem cells. Biotechnology …, Wiley Online Library, cited by 110 (22.00 per year)
Y Pan, A Patil, P Guo, C Zhou (2017) A novel projection based electro-stereolithography (PES) process for production of 3D polymer-particle composite objects. Rapid Prototyping Journal, emeraldinsight.com, cited by 6 (3.00 per year)
MK Joshi, AP Tiwari, HR Pant, ... (2015) In situ generation of cellulose nanocrystals in polycaprolactone nanofibers: effects on crystallinity, mechanical strength, biocompatibility, and biomimetic mineralization. … applied materials & …, ACS Publications, cited by 54 (13.50 per year)
NF Lepora, A Duff, A Mura, TJ Prescott, ... (2014) Biomimetic and biohybrid systems., Springer International Publishing, cited by 6 (1.20 per year)
AG Domel, G Domel, JC Weaver, ... (2018) Hydrodynamic properties of biomimetic shark skin: Effect of denticle size and swimming speed. … & biomimetics, iopscience.iop.org, cited by 3 (3.00 per year)
MM Savalani, RA Harris (2006) Layer Manufacturing for in Vivo Devices. … , Part H: Journal of Engineering in …, journals.sagepub.com, cited by 19 (1.46 per year)
CH Park, HF Rios, AD Taut, ... (2013) Image-based, fiber guiding scaffolds: a platform for regenerating tissue interfaces. … Engineering Part C …, liebertpub.com, cited by 57 (9.50 per year)
S Mehrotra, JC Moses, A Bandyopadhyay, ... (2019) 3D Printing/Bioprinting Based Tailoring of in Vitro Tissue Models: Recent Advances and Challenges. ACS Applied Bio …, ACS Publications, cited by 1 (1.00 per year)
N Bhardwaj, D Chouhan, BB Mandal (2018) 3D functional scaffolds for skin tissue engineering. Functional 3D Tissue Engineering …, Elsevier, cited by 8 (8.00 per year)
TJ Klein, SC Rizzi, JC Reichert, ... (2009) Strategies for zonal cartilage repair using hydrogels. Macromolecular …, Wiley Online Library, cited by 134 (13.40 per year)
P Gruber, B Imhof (2017) Patterns of Growth—Biomimetics and Architectural Design. Buildings, mdpi.com, cited by 1 (0.50 per year)
H He, Y Pan, A Feinerman, J Xu (2018) Air-Diffusion-Channel Constrained Surface Based Stereolithography for Three-Dimensional Printing of Objects With Wide Solid Cross Sections. Journal of …, … .asmedigitalcollection.asme.org, cited by 6 (6.00 per year)
P Calvert (2016) 3D printing of gels with living photosynthetic algae. Mrs Advances, cambridge.org, cited by 1 (0.33 per year)
GL Koons, AG Mikos (2019) Progress in three-dimensional printing with growth factors. Journal of controlled release, Elsevier, cited by 2 (2.00 per year)
V Lee, G Singh, JP Trasatti, C Bjornsson, ... (2013) Design and fabrication of human skin by three-dimensional bioprinting. … Engineering Part C …, liebertpub.com, cited by 303 (50.50 per year)
S Vijayavenkataraman, J Fuh, W Lu (2017) 3D printing and 3D bioprinting in pediatrics. Bioengineering, mdpi.com, cited by 13 (6.50 per year)
E Gargus, P Lewis, R Shah (2018) Bioinks for 3D printing. 3D Bioprinting in …, content.taylorfrancis.com, cited by 1 (1.00 per year)

X Li, D Li, B Lu, Y Tang, L Wang, ... (2005) Design and fabrication of CAP scaffolds by indirect solid free form fabrication. Rapid Prototyping …, emeraldinsight.com, cited by 49 (3.50 per year)
E Prina, P Mistry, LE Sidney, J Yang, ... (2017) 3D microfabricated scaffolds and microfluidic devices for ocular surface replacement: a review. Stem Cell Reviews and …, Springer, cited by 5 (2.50 per year)
SE Bakarich, R Gorkin III, R Gately, S Naficy, ... (2017) 3D printing of tough hydrogel composites with spatially varying materials properties. Additive …, Elsevier, cited by 17 (8.50 per year)
KM Park, S Gerecht (2015) Polymeric hydrogels as artificial extracellular microenvironments for cancer research. European Polymer Journal, Elsevier, cited by 11 (2.75 per year)
SC Cox (2013) Synthesis and 3D printing of hydroxyapatite scaffolds for applications in bone tissue engineering., wrap.warwick.ac.uk, cited by 3 (0.50 per year)
CM Schumacher, M Loepfe, R Fuhrer, RN Grass, ... (2014) 3D printed lost-wax casted soft silicone monoblocks enable heart-inspired pumping by internal combustion. Rsc Advances, pubs.rsc.org, cited by 23 (4.60 per year)
C Comotti, D Regazzoni, ... (2017) Additive manufacturing to advance functional design: an application in the medical field. Journal of …, ebooks.asmedigitalcollection.asme …, cited by 13 (6.50 per year)
JT Borenstein, EJ Weinberg, BK Orrick, ... (2007) Microfabrication of three-dimensional engineered scaffolds. Tissue …, liebertpub.com, cited by 180 (15.00 per year)
AC Daly, SE Critchley, EM Rencsok, DJ Kelly (2016) A comparison of different bioinks for 3D bioprinting of fibrocartilage and hyaline cartilage. Biofabrication, iopscience.iop.org, cited by 83 (27.67 per year)
T Matsumoto, JI Sasaki, E Alsberg, H Egusa, H Yatani, ... (2007) Three-dimensional cell and tissue patterning in a strained fibrin gel system. PloS one, journals.plos.org, cited by 89 (7.42 per year)
R Nielson, B Kaehr, JB Shear (2009) Microreplication and design of biological architectures using dynamic-mask multiphoton lithography. Small, Wiley Online Library, cited by 131 (13.10 per year)
SH Jin, JK Lee, S Lee, KC Lee (2014) Output characteristic of a flexible tactile sensor manufactured by 3D printing technique. Journal of the …, Korean Society of Precision …, cited by 8 (1.60 per year)
JH Park, J Jang, DW Cho (2014) Three-dimensional printed 3D structure for tissue engineering. Transactions of the Korean Society of …, koreascience.or.kr, cited by 13 (2.60 per year)
连芩, 庄佩, 边卫国, 李涤尘, 靳忠民 (2015) 大尺寸关节支架的 3D 打印及应用. 中国科学: 信息科学, engine.scichina.com, cited by 5 (1.25 per year)
艾尔肯, 刘广鹏 (2012) Micro-CT 及三维打印机制备仿生化数字化组织工程指骨支架材料的研究. 中华手外科杂志, airitilibrary.com, cited by 5 (0.71 per year)

bio-inspired 3D-printing
Publish or Perish 7.10.2373.7118
Windows (x64) edition, running on Windows 10.0.16299 (x64)
Search terms
Keywords: bio-inspired 3D-printing
Years: all

Data retrieval
Data source: Google Scholar
Search date: 2019-07-08 08:56:46 +1200
Cache date: 2019-07-08 09:25:13 +1200
Search result: [0] No error

Metrics
Reference date: 2019-07-08 09:25:13 +1200
Publication years: 1976-2019
Citation years: 43 (1976-2019)
Papers: 1000
Citations: 94649
Citations/year: 2201.14
Citations/paper: 94.65 (acc1=927, acc2=825, acc5=628, acc10=444, acc20=267)
Authors/paper: 4.01/4.0/4 (mean/median/mode)
Age-weighed citation rate: 17732.20 (sqrt=133.16), 5200.03/author
Hirsch h-index: 166 (a=3.43, m=3.86, 67977 cites=71.8% coverage)
Egghe g-index: 286 (g/h=1.72, 82024 cites=86.7% coverage)
PoP hI,norm: 85
PoP hI,annual: 1.98

Results
LS Dimas, GH Bratzel, I Eylon, ... (2013) Tough composites inspired by mineralized natural materials: computation, 3D printing, and testing. Advanced Functional …, Wiley Online Library, cited by 205 (34.17 per year)
M Gou, X Qu, W Zhu, M Xiang, J Yang, K Zhang, ... (2014) Bio-inspired detoxification using 3D-printed hydrogel nanocomposites. Nature …, nature.com, cited by 155 (31.00 per year)
P Tran, TD Ngo, A Ghazlan, D Hui (2017) Bimaterial 3D printing and numerical analysis of bio-inspired composite structures under in-plane and transverse loadings. Composites Part B: Engineering, Elsevier, cited by 58 (29.00 per year)
GX Gu, I Su, S Sharma, ... (2016) Three-dimensional-printing of bio-inspired composites. Journal of …, … .asmedigitalcollection.asme.org, cited by 67 (22.33 per year)
W Zhu, X Ma, M Gou, D Mei, K Zhang, ... (2016) 3D printing of functional biomaterials for tissue engineering. Current opinion in …, Elsevier, cited by 153 (51.00 per year)
F Libonati, GX Gu, Z Qin, L Vergani, ... (2016) Bone-inspired materials by design: toughness amplification observed using 3D printing and testing. Advanced Engineering …, Wiley Online Library, cited by 58 (19.33 per year)
IA Malik, M Mirkhalaf, F Barthelat (2017) Bio-inspired "jigsaw"-like interlocking sutures: Modeling, optimization, 3D printing and testing. Journal of the Mechanics and Physics of …, Elsevier, cited by 22 (11.00 per year)

CM Magin, DL Alge, KS Anseth (2016) Bio-inspired 3D microenvironments: a new dimension in tissue engineering. Biomedical Materials, iopscience.iop.org, cited by 37 (12.33 per year)
JJ Martin, BE Fiore, RM Erb (2015) Designing bioinspired composite reinforcement architectures via 3D magnetic printing. Nature communications, nature.com, cited by 142 (35.50 per year)
R Martini, Y Balit, F Barthelat (2017) A comparative study of bio-inspired protective scales using 3D printing and mechanical testing. Acta biomaterialia, Elsevier, cited by 20 (10.00 per year)
J Hou, H Zhang, Q Yang, M Li, Y Song, ... (2014) Bio-inspired photonic-crystal microchip for fluorescent ultratrace detection. Angewandte Chemie …, Wiley Online Library, cited by 168 (33.60 per year)
E Lin, Y Li, C Ortiz, MC Boyce (2014) 3D printed, bio-inspired prototypes and analytical models for structured suture interfaces with geometrically-tuned deformation and failure behavior. Journal of the Mechanics and Physics of …, Elsevier, cited by 67 (13.40 per year)
MM Porter, N Ravikumar, F Barthelat, ... (2017) 3D-printing and mechanics of bio-inspired articulated and multi-material structures. Journal of the mechanical …, Elsevier, cited by 15 (7.50 per year)
BG Compton, JA Lewis (2014) 3D-printing of lightweight cellular composites. Advanced materials, Wiley Online Library, cited by 560 (112.00 per year)
NW Bartlett, MT Tolley, JTB Overvelde, ... (2015) A 3D-printed, functionally graded soft robot powered by combustion. …, science.sciencemag.org, cited by 367 (91.75 per year)
W Wu, A DeConinck, JA Lewis (2011) Omnidirectional printing of 3D microvascular networks. Advanced materials, Wiley Online Library, cited by 361 (45.13 per year)
LS Dimas, MJ Buehler (2014) Modeling and additive manufacturing of bio-inspired composites with tunable fracture mechanical properties. Soft Matter, pubs.rsc.org, cited by 47 (9.40 per year)
P Cai, M Layani, WR Leow, S Amini, Z Liu, ... (2016) Bio-Inspired Mechanotactic Hybrids for Orchestrating Traction-Mediated Epithelial Migration. Advanced …, Wiley Online Library, cited by 42 (14.00 per year)
AL Rutz, KE Hyland, AE Jakus, ... (2015) A multimaterial bioink method for 3D printing tunable, cell-compatible hydrogels. Advanced …, Wiley Online Library, cited by 219 (54.75 per year)
D Han, H Gu, J Kim, S Yokota (2017) A bio-inspired 3D-printed hybrid finger with integrated ECF (electro-conjugate fluid) micropumps. Sensors and Actuators A: Physical, Elsevier, cited by 15 (7.50 per year)
V Slesarenko, N Kazarinov, ... (2017) Distinct failure modes in bio-inspired 3D-printed staggered composites under non-aligned loadings. Smart Materials and …, iopscience.iop.org, cited by 16 (8.00 per year)
AR Studart (2016) Additive manufacturing of biologically-inspired materials. Chemical Society Reviews, pubs.rsc.org, cited by 142 (47.33 per year)
S Hong, D Sycks, HF Chan, S Lin, GP Lopez, ... (2015) 3D printing of highly stretchable and tough hydrogels into complex, cellularized structures. Advanced …, Wiley Online Library, cited by 325 (81.25 per year)
L Djumas, A Molotnikov, GP Simon, Y Estrin (2016) Enhanced mechanical performance of bio-inspired hybrid structures utilising topological interlocking geometry. Scientific reports, nature.com, cited by 35 (11.67 per year)

S Song, M Sitti (2014) Soft grippers using micro-fibrillar adhesives for transfer printing. Advanced Materials, Wiley Online Library, cited by 58 (11.60 per year)
RL Truby, JA Lewis (2016) Printing soft matter in three dimensions. Nature, nature.com, cited by 277 (92.33 per year)
M Lapeyre, P Rouanet, J Grizou, S Nguyen, ... (2014) Poppy project: open-source fabrication of 3D printed humanoid robot for science, education and art., hal.inria.fr, cited by 39 (7.80 per year)
JD Carrico, NW Traeden, M Aureli, ... (2015) Fused filament 3D printing of ionic polymer-metal composites (IPMCs). Smart Materials and …, iopscience.iop.org, cited by 41 (10.25 per year)
H Yu, CY Tay, M Pal, WS Leong, H Li, ... (2013) A Bio-inspired Platform to Modulate Myogenic Differentiation of Human Mesenchymal Stem Cells Through Focal Adhesion Regulation. Advanced …, Wiley Online Library, cited by 35 (5.83 per year)
E Lin, Y Li, JC Weaver, C Ortiz, ... (2014) Tunability and enhancement of mechanical behavior with additively manufactured bio-inspired hierarchical suture interfaces. Journal of Materials …, cambridge.org, cited by 46 (9.20 per year)
Y He, S Guo, L Shi, S Pan, ... (2014) 3D printing technology-based an amphibious spherical robot. 2014 IEEE International …, ieeexplore.ieee.org, cited by 23 (4.60 per year)
D Kokkinis, M Schaffner, AR Studart (2015) Multimaterial magnetically assisted 3D printing of composite materials. Nature communications, nature.com, cited by 233 (58.25 per year)
CT Kao, CC Lin, YW Chen, CH Yeh, HY Fang, ... (2015) Poly (dopamine) coating of 3D printed poly (lactic acid) scaffolds for bone tissue engineering. Materials Science and …, Elsevier, cited by 129 (32.25 per year)
CB Highley, CB Rodell, JA Burdick (2015) Direct 3D printing of shear-thinning hydrogels into self-healing hydrogels. Advanced Materials, Wiley Online Library, cited by 309 (77.25 per year)
AV Do, B Khorsand, SM Geary, ... (2015) 3D printing of scaffolds for tissue regeneration applications. Advanced healthcare …, Wiley Online Library, cited by 263 (65.75 per year)
M Mirkhalaf, F Barthelat (2017) Design, 3D printing and testing of architectured materials with bistable interlocks. Extreme Mechanics Letters, Elsevier, cited by 20 (10.00 per year)
B Compton (2015) 3D printing of composites with controlled architecture., dc.engconfintl.org, cited by 15 (3.75 per year)
J Wang, L Lin, Q Cheng, L Jiang (2012) A Strong Bio-Inspired Layered PNIPAM–Clay Nanocomposite Hydrogel. … Chemie International Edition, Wiley Online Library, cited by 150 (21.43 per year)
E Feilden, C Ferraro, Q Zhang, E García-Tuñón, ... (2017) 3D printing bioinspired ceramic composites. Scientific reports, nature.com, cited by 20 (10.00 per year)
GX Gu, F Libonati, SD Wettermark, ... (2017) Printing nature: Unraveling the role of nacre's mineral bridges. Journal of the mechanical …, Elsevier, cited by 45 (22.50 per year)
SE Bakarich, R Gorkin III, R Gately, S Naficy, ... (2017) 3D printing of tough hydrogel composites with spatially varying materials properties. Additive …, Elsevier, cited by 17 (8.50 per year)
X Wang, M Jiang, Z Zhou, J Gou, D Hui (2017) 3D printing of polymer matrix composites: A review and prospective. Composites Part B: Engineering, Elsevier, cited by 421 (210.50 per year)
T Patino, R Mestre, S Sanchez (2016) Miniaturized soft bio-hybrid robotics: a step forward into healthcare applications. Lab on a Chip, pubs.rsc.org, cited by 27 (9.00 per year)

S Shin, J Seo, H Han, S Kang, H Kim, T Lee (2016) Bio-inspired extreme wetting surfaces for biomedical applications. Materials, mdpi.com, cited by 61 (20.33 per year)
S Malek, JR Raney, JA Lewis, ... (2017) Lightweight 3D cellular composites inspired by balsa. Bioinspiration & …, iopscience.iop.org, cited by 16 (8.00 per year)
JZ Gul, M Sajid, MM Rehman, GU Siddiqui, ... (2018) 3D printing for soft robotics–a review. … and technology of …, Taylor & Francis, cited by 23 (23.00 per year)
P Zhang, MA Heyne, AC To (2015) Biomimetic staggered composites with highly enhanced energy dissipation: Modeling, 3D printing, and testing. Journal of the Mechanics and Physics of …, Elsevier, cited by 46 (11.50 per year)
H Devaraj, J Travas-Sejdic, R Sharma, ... (2015) Bio-inspired flow sensor from printed PEDOT: PSS micro-hairs. Bioinspiration & …, iopscience.iop.org, cited by 13 (3.25 per year)
JZ Gul, BS Yang, YJ Yang, DE Chang, ... (2016) In situ UV curable 3D printing of multi-material tri-legged soft bot with spider mimicked multi-step forward dynamic gait. Smart Materials and …, iopscience.iop.org, cited by 21 (7.00 per year)
AK Goel, DA McAdams, RB Stone (2015) Biologically inspired design., Springer, cited by 78 (19.50 per year)
K Chung, S Yu, CJ Heo, JW Shim, SM Yang, ... (2012) Flexible, angle-independent, structural color reflectors inspired by morpho butterfly wings. Advanced …, Wiley Online Library, cited by 168 (24.00 per year)
AP Zhang, X Qu, P Soman, KC Hribar, ... (2012) Rapid fabrication of complex 3D extracellular microenvironments by dynamic optical projection stereolithography. Advanced …, Wiley Online Library, cited by 181 (25.86 per year)
L Henderson, T Glaser, F Kuester (2017) Towards bio-inspired structural design of a 3D printable, ballistically deployable, multi-rotor UAV. 2017 IEEE Aerospace …, ieeexplore.ieee.org, cited by 7 (3.50 per year)
R Wimmer, B Steyrer, J Woess, T Koddenberg, ... (2015) 3D printing and wood. Pro Ligno, researchgate.net, cited by 11 (2.75 per year)
A Bakhshinejad, RM D'souza (2015) A brief comparison between available bio-printing methods. 2015 IEEE Great Lakes …, ieeexplore.ieee.org, cited by 11 (2.75 per year)
W Zhang, FK Yang, Z Pan, J Zhang, ... (2014) Bio-Inspired Dopamine Functionalization of Polypyrrole for Improved Adhesion and Conductivity. Macromolecular rapid …, Wiley Online Library, cited by 48 (9.60 per year)
V Slesarenko, S Rudykh (2018) Towards mechanical characterization of soft digital materials for multimaterial 3D-printing. International Journal of Engineering Science, Elsevier, cited by 14 (14.00 per year)
A Arslan-Yildiz, R El Assal, P Chen, S Guven, ... (2016) Towards artificial tissue models: past, present, and future of 3D bioprinting. …, iopscience.iop.org, cited by 92 (30.67 per year)
JZ Gul, KY Su, KH Choi (2018) Fully 3D printed multi-material soft bio-inspired whisker sensor for underwater-induced vortex detection. Soft robotics, liebertpub.com, cited by 7 (7.00 per year)
M Röhrig, M Thiel, M Worgull, H Hölscher (2012) 3D direct laser writing of nano-and microstructured hierarchical gecko-mimicking surfaces. Small, Wiley Online Library, cited by 101 (14.43 per year)
E MacDonald, R Wicker (2016) Multiprocess 3D printing for increasing component functionality. Science, science.sciencemag.org, cited by 187 (62.33 per year)
HD Kim, SR Peyton (2011) Bio-inspired materials for parsing matrix physicochemical control of cell migration: a review. Integrative biology, academic.oup.com, cited by 43 (5.38 per year)

Z Liu, W Chen, J Carstensen, J Ketkaew, RMO Mota, ... (2016) 3D metallic glass cellular structures. Acta Materialia, Elsevier, cited by 28 (9.33 per year)
E Siéfert, E Reyssat, J Bico, B Roman (2019) Bio-inspired pneumatic shape-morphing elastomers. Nature materials, nature.com, cited by 10 (10.00 per year)
J Li, J Zhu, X Gao (2014) Bio-Inspired High-Performance Antireflection and Antifogging Polymer Films. Small, Wiley Online Library, cited by 49 (9.80 per year)
D Richards, M Amos (2015) Designing with gradients: bio-inspired computation for digital fabrication., e-space.mmu.ac.uk, cited by 8 (2.00 per year)
D Gu (2016) Materials creation adds new dimensions to 3D printing. Science Bulletin, Springer, cited by 12 (4.00 per year)
JG Fernandez, DE Ingber (2012) Unexpected strength and toughness in chitosan-fibroin laminates inspired by insect cuticle. Advanced materials, Wiley Online Library, cited by 57 (8.14 per year)
W Liu, Y Li, J Liu, X Niu, Y Wang, D Li (2013) Application and performance of 3D printing in nanobiomaterials. Journal of Nanomaterials, dl.acm.org, cited by 31 (5.17 per year)
D Zappetti, S Mintchev, J Shintake, ... (2017) Bio-inspired tensegrity soft modular robots. Conference on Biomimetic …, Springer, cited by 7 (3.50 per year)
D Singh, D Singh, S Han (2016) 3D printing of scaffold for cells delivery: Advances in skin tissue engineering. Polymers, mdpi.com, cited by 43 (14.33 per year)
U Jammalamadaka, K Tappa (2018) Recent advances in biomaterials for 3D printing and tissue engineering. Journal of functional biomaterials, mdpi.com, cited by 46 (46.00 per year)
Y Yang, Y Chen, Y Wei, Y Li (2016) Novel design and three-dimensional printing of variable stiffness robotic grippers. Journal of …, … .asmedigitalcollection.asme.org, cited by 23 (7.67 per year)
R Chang, J Nam, W Sun (2008) Direct Cell Writing of 3D Microorgan for In Vitro Pharmacokinetic Model. Tissue Engineering Part C: Methods, liebertpub.com, cited by 159 (14.45 per year)
JT Muth, DM Vogt, RL Truby, Y Mengüç, ... (2014) Embedded 3D printing of strain sensors within highly stretchable elastomers. Advanced …, Wiley Online Library, cited by 679 (135.80 per year)
W Bian, D Li, Q Lian, X Li, W Zhang, ... (2012) Fabrication of a bio-inspired beta-Tricalcium phosphate/collagen scaffold based on ceramic stereolithography and gel casting for osteochondral tissue engineering. Rapid Prototyping …, emeraldinsight.com, cited by 87 (12.43 per year)
S Babaee, J Shim, JC Weaver, ER Chen, ... (2013) 3D soft metamaterials with negative Poisson's ratio. Advanced …, Wiley Online Library, cited by 311 (51.83 per year)
O Tricinci, T Terencio, B Mazzolai, ... (2015) 3D Micropatterned Surface Inspired by Salvinia molesta via Direct Laser Lithography. … applied materials & …, ACS Publications, cited by 42 (10.50 per year)
R Domingo-Roca, B Tiller, JC Jackson, ... (2018) Bio-inspired 3D-printed piezoelectric device for acoustic frequency selection. Sensors and Actuators A …, Elsevier, cited by 4 (4.00 per year)
SJ Furst, G Bunget, S Seelecke (2012) Design and fabrication of a bat-inspired flapping-flight platform using shape memory alloy muscles and joints. Smart Materials and Structures, iopscience.iop.org, cited by 39 (5.57 per year)
C Mandrycky, Z Wang, K Kim, DH Kim (2016) 3D bioprinting for engineering complex tissues. Biotechnology advances, Elsevier, cited by 378 (126.00 per year)

AR Studart (2015) Biologically inspired dynamic material systems. Angewandte Chemie International Edition, Wiley Online Library, cited by 77 (19.25 per year)
YJ Choi, TG Kim, J Jeong, HG Yi, ... (2016) 3D cell printing of functional skeletal muscle constructs using skeletal muscle-derived bioink. Advanced …, Wiley Online Library, cited by 68 (22.67 per year)
SZ Guo, F Gosselin, N Guerin, AM Lanouette, ... (2013) Solvent-cast three-dimensional printing of multifunctional microsystems. Small, Wiley Online Library, cited by 91 (15.17 per year)
CS Tiwary, S Kishore, S Sarkar, ... (2015) Morphogenesis and mechanostabilization of complex natural and 3D printed shapes. Science …, advances.sciencemag.org, cited by 17 (4.25 per year)
WW Godfrey, SB Nair (2011) A bio-inspired technique for servicing networked robots. International Journal of Rapid …, inderscienceonline.com, cited by 7 (0.88 per year)
W Xie, V Kothari, BS Terry (2015) A bio-inspired attachment mechanism for long-term adhesion to the small intestine. Biomedical microdevices, Springer, cited by 11 (2.75 per year)
A Davoudinejad, MM Ribo, DB Pedersen, ... (2018) Direct fabrication of bio-inspired gecko-like geometries with vat polymerization additive manufacturing method. Journal of …, iopscience.iop.org, cited by 7 (7.00 per year)
J Ou, G Dublon, CY Cheng, F Heibeck, ... (2016) Cilllia: 3D printed micro-pillar structures for surface texture, actuation and sensing. Proceedings of the …, dl.acm.org, cited by 36 (12.00 per year)
FL Bargardi, H Le Ferrand, R Libanori, ... (2016) Bio-inspired self-shaping ceramics. Nature …, nature.com, cited by 26 (8.67 per year)
SM Kang, I You, WK Cho, HK Shon, ... (2010) One-step modification of superhydrophobic surfaces by a mussel-inspired polymer coating. Angewandte Chemie …, Wiley Online Library, cited by 322 (35.78 per year)
V Guarino, A Gloria, MG Raucci, ... (2012) Bio-inspired composite and cell instructive platforms for bone regeneration. International …, Taylor & Francis, cited by 43 (6.14 per year)
YE Choonara, LC du Toit, P Kumar, ... (2016) 3D-printing and the effect on medical costs: a new era?. Expert review of …, Taylor & Francis, cited by 43 (14.33 per year)
P Sitthi-Amorn, JE Ramos, Y Wangy, J Kwan, ... (2015) MultiFab: a machine vision assisted platform for multi-material 3D printing. ACM Transactions on …, dl.acm.org, cited by 101 (25.25 per year)
Y Kobayashi, T Saito, A Isogai (2014) Aerogels with 3D ordered nanofiber skeletons of liquid-crystalline nanocellulose derivatives as tough and transparent insulators. … Chemie International Edition, Wiley Online Library, cited by 232 (46.40 per year)
Y Zhang, M Lu, CH Wang, G Sun, G Li (2016) Out-of-plane crashworthiness of bio-inspired self-similar regular hierarchical honeycombs. Composite Structures, Elsevier, cited by 38 (12.67 per year)
Z He, Y Chen, J Yang, C Tang, J Lv, Y Liu, J Mei, ... (2017) Fabrication of Polydimethylsiloxane films with special surface wettability by 3D printing. Composites Part B …, Elsevier, cited by 13 (6.50 per year)
B Eijking, R Sanders, G Krijnen (2017) Development of whisker inspired 3D multi-material printed flexible tactile sensors. 2017 IEEE SENSORS, ieeexplore.ieee.org, cited by 4 (2.00 per year)

C Richter, H Lipson (2011) Untethered hovering flapping flight of a 3D-printed mechanical insect. Artificial life, MIT Press, cited by 85 (10.63 per year)
J Peng, Q Cheng (2017) High-performance nanocomposites inspired by nature. Advanced Materials, Wiley Online Library, cited by 35 (17.50 per year)
F Zhu, L Cheng, ZJ Wang, W Hong, ZL Wu, ... (2017) 3D-printed ultratough hydrogel structures with titin-like domains. … applied materials & …, ACS Publications, cited by 18 (9.00 per year)
H Ragones, D Schreiber, A Inberg, O Berkh, ... (2015) Disposable electrochemical sensor prepared using 3D printing for cell and tissue diagnostics. Sensors and Actuators B …, Elsevier, cited by 22 (5.50 per year)
M Schaffner, PA Rühs, F Coulter, ... (2017) 3D printing of bacteria into functional complex materials. Science …, advances.sciencemag.org, cited by 44 (22.00 per year)
K Chen, B Shi, Y Yue, J Qi, L Guo (2015) Binary synergy strengthening and toughening of bio-inspired nacre-like graphene oxide/sodium alginate composite paper. Acs Nano, ACS Publications, cited by 84 (21.00 per year)
A Zadpoor (2017) Design for additive bio-manufacturing: From patient-specific medical devices to rationally designed meta-biomaterials. International journal of molecular sciences, mdpi.com, cited by 21 (10.50 per year)
M Singh, Y Tong, K Webster, E Cesewski, AP Haring, ... (2017) 3D printed conformal microfluidics for isolation and profiling of biomarkers from whole organs. Lab on a Chip, pubs.rsc.org, cited by 23 (11.50 per year)
CH Yeh, YW Chen, MY Shie, HY Fang (2015) Poly (dopamine)-assisted immobilization of Xu Duan on 3D printed poly (lactic acid) scaffolds to up-regulate osteogenic and angiogenic markers of bone marrow stem …. Materials, mdpi.com, cited by 28 (7.00 per year)
S Kumar, BL Wardle, MF Arif (2016) Strength and performance enhancement of bonded joints by spatial tailoring of adhesive compliance via 3D printing. ACS applied materials & interfaces, ACS Publications, cited by 22 (7.33 per year)
N Mehrban, GZ Teoh, MA Birchall (2016) 3D bioprinting for tissue engineering: Stem cells in hydrogels. International journal of …, discovery.ucl.ac.uk, cited by 31 (10.33 per year)
M Schaffner, JA Faber, L Pianegonda, PA Rühs, ... (2018) 3D printing of robotic soft actuators with programmable bioinspired architectures. Nature …, nature.com, cited by 32 (32.00 per year)
PS Mehta, JS Ocampo, A Tovar, P Chaudhari (2016) Bio-inspired design of lightweight and protective structures., sae.org, cited by 4 (1.33 per year)
H Ko, H Yi, HE Jeong (2017) Wall and ceiling climbing quadruped robot with superior water repellency manufactured using 3D printing (UNIclimb). International Journal of Precision Engineering and …, Springer, cited by 15 (7.50 per year)
DB Brommer, T Giesa, DI Spivak, MJ Buehler (2015) Categorical prototyping: incorporating molecular mechanisms into 3D printing. Nanotechnology, iopscience.iop.org, cited by 4 (1.00 per year)
C Zhang, C Rossi (2017) A review of compliant transmission mechanisms for bio-inspired flapping-wing micro air vehicles. Bioinspiration & biomimetics, iopscience.iop.org, cited by 23 (11.50 per year)
M Lapeyre, P Rouanet, PY Oudeyer (2013) Poppy: A new bio-inspired humanoid robot platform for biped locomotion and physical human-robot interaction., hal.inria.fr, cited by 4 (0.67 per year)

B Farahi (2016) Caress of the gaze: A gaze actuated 3D printed body architecture. 36th Annual Conference of the Association for …, papers.cumincad.org, cited by 6 (2.00 per year)
J Delamare, R Sanders, G Krijnen (2016) 3D printed biomimetic whisker-based sensor with co-planar capacitive sensing. 2016 IEEE SENSORS, ieeexplore.ieee.org, cited by 7 (2.33 per year)
JH Shin, JH Heo, S Jeon, JH Park, S Kim, ... (2019) Bio-inspired hollow PDMS sponge for enhanced oil–water separation. Journal of hazardous …, Elsevier, cited by 4 (4.00 per year)
MM Porter, N Ravikumar (2017) 3D-printing a 'family'of biomimetic models to explain armored grasping in syngnathid fishes. Bioinspiration & biomimetics, iopscience.iop.org, cited by 5 (2.50 per year)
S Derakhshanfar, R Mbeleck, K Xu, X Zhang, W Zhong, ... (2018) 3D bioprinting for biomedical devices and tissue engineering: A review of recent trends and advances. Bioactive materials, Elsevier, cited by 60 (60.00 per year)
C Xue, X Shi, X Fang, H Tao, H Zhu, F Yu, ... (2016) The "pure marriage" between 3D printing and well-ordered nanoarrays by using peald assisted hydrothermal surface engineering. … applied materials & …, ACS Publications, cited by 10 (3.33 per year)
T Zhang, C Zhou, S Su (2015) Design and development of bio-inspired flapping wing aerial vehicles. 2015 International Conference on …, ieeexplore.ieee.org, cited by 5 (1.25 per year)
HB Evans, AM Hamed, S Gorumlu, ... (2018) Engineered bio-inspired coating for passive flow control. Proceedings of the …, National Acad Sciences, cited by 5 (5.00 per year)
R Yan, M Chen, H Zhou, T Liu, X Tang, K Zhang, ... (2016) Bio-inspired plasmonic nanoarchitectured hybrid system towards enhanced far red-to-near infrared solar photocatalysis. Scientific reports, nature.com, cited by 27 (9.00 per year)
DE Ingber, VC Mow, D Butler, L Niklason, ... (2006) Tissue engineering and developmental biology: going biomimetic. Tissue …, liebertpub.com, cited by 243 (18.69 per year)
Z Hu, K Thiyagarajan, A Bhusal, T Letcher, ... (2017) Design of ultra-lightweight and high-strength cellular structural composites inspired by biomimetics. Composites Part B …, Elsevier, cited by 14 (7.00 per year)
Q Li, Q Zeng, L Shi, X Zhang, KQ Zhang (2016) Bio-inspired sensors based on photonic structures of Morpho butterfly wings: a review. Journal of Materials …, pubs.rsc.org, cited by 31 (10.33 per year)
WG Bae, HN Kim, D Kim, SH Park, ... (2014) 25th anniversary article: scalable multiscale patterned structures inspired by nature: the role of hierarchy. Advanced …, Wiley Online Library, cited by 120 (24.00 per year)
F Libonati (2016) Bio-inspired composites: using nature to tackle composite limitations. Advanced Engineering Materials and Modeling, Wiley Online Library, cited by 4 (1.33 per year)
J Groll, T Boland, T Blunk, JA Burdick, DW Cho, ... (2016) Biofabrication: reappraising the definition of an evolving field. …, iopscience.iop.org, cited by 205 (68.33 per year)
B Lu, H Lan, H Liu (2018) Additive manufacturing frontier: 3D printing electronics. Opto-Electronic Advances, oejournal.org, cited by 11 (11.00 per year)
H Zhou, J Xu, X Liu, H Zhang, D Wang, ... (2018) Bio-Inspired Photonic Materials: Prototypes and Structural Effect Designs for Applications in Solar Energy Manipulation. Advanced Functional …, Wiley Online Library, cited by 18 (18.00 per year)

B Winstone, C Melhuish, T Pipe, ... (2016) Toward bio-inspired tactile sensing capsule endoscopy for detection of submucosal tumors. IEEE Sensors …, ieeexplore.ieee.org, cited by 8 (2.67 per year)
LF Boesel, C Greiner, E Arzt, ... (2010) Gecko-inspired surfaces: a path to strong and reversible dry adhesives. Advanced Materials, Wiley Online Library, cited by 338 (37.56 per year)
H Seitz, W Rieder, S Irsen, B Leukers, ... (2005) Three-dimensional printing of porous ceramic scaffolds for bone tissue engineering. Journal of Biomedical …, Wiley Online Library, cited by 654 (46.71 per year)
N Oxman (2011) Variable property rapid prototyping: inspired by nature, where form is characterized by heterogeneous compositions, the paper presents a novel approach to layered …. Virtual and physical prototyping, Taylor & Francis, cited by 133 (16.63 per year)
LM Bellan, M Pearsall, DM Cropek, ... (2012) A 3D interconnected microchannel network formed in gelatin by sacrificial shellac microfibers. Advanced …, Wiley Online Library, cited by 76 (10.86 per year)
JD Carrico, KJ Kim, KK Leang (2017) 3D-printed ionic polymer-metal composite soft crawling robot. 2017 IEEE International …, ieeexplore.ieee.org, cited by 11 (5.50 per year)
T Boland, V Mironov, A Gutowska, ... (2003) Cell and organ printing 2: Fusion of cell aggregates in three-dimensional gels. … Record Part A …, Wiley Online Library, cited by 338 (21.13 per year)
R Aversa, F Tamburrino, RV Petrescu, ... (2016) Biomechanically inspired shape memory effect machines driven by muscle like acting NiTi alloys. American Journal of …, papers.ssrn.com, cited by 100 (33.33 per year)
DC Zuluaga, A Menges (2015) 3D printed hygroscopic programmable material systems. MRS Online Proceedings Library Archive, cambridge.org, cited by 4 (1.00 per year)
Y Wu, Q Liu, J Fu, Q Li, D Hui (2017) Dynamic crash responses of bio-inspired aluminum honeycomb sandwich structures with CFRP panels. Composites Part B: Engineering, Elsevier, cited by 48 (24.00 per year)
Y Chen, T Li, Z Jia, F Scarpa, CW Yao, L Wang (2018) 3D printed hierarchical honeycombs with shape integrity under large compressive deformations. Materials & Design, Elsevier, cited by 39 (39.00 per year)
U Kalsoom, PN Nesterenko, B Paull (2016) Recent developments in 3D printable composite materials. RSC Advances, pubs.rsc.org, cited by 77 (25.67 per year)
AK Au, W Huynh, LF Horowitz, ... (2016) 3D-printed microfluidics. Angewandte Chemie …, Wiley Online Library, cited by 264 (88.00 per year)
L Huang, R Jiang, J Wu, J Song, H Bai, B Li, ... (2017) Ultrafast digital printing toward 4D shape changing materials. Advanced …, Wiley Online Library, cited by 134 (67.00 per year)
M Kuang, J Wang, B Bao, F Li, L Wang, ... (2014) Inkjet printing patterned photonic crystal domes for wide viewing-angle displays by controlling the sliding three phase contact line. Advanced Optical …, Wiley Online Library, cited by 121 (24.20 per year)
K Kumar, J Liu, C Christianson, M Ali, MT Tolley, ... (2017) A biologically inspired, functionally graded end effector for soft robotics applications. Soft robotics, liebertpub.com, cited by 11 (5.50 per year)
S Li, DM Vogt, D Rus, RJ Wood (2017) Fluid-driven origami-inspired artificial muscles. Proceedings of the …, National Acad Sciences, cited by 83 (41.50 per year)
B Ward-Cherrier, N Pestell, L Cramphorn, B Winstone, ... (2018) The tactip family: Soft optical tactile sensors with 3d-printed biomimetic morphologies. Soft robotics, liebertpub.com, cited by 28 (28.00 per year)

JD Carrico, KK Leang (2017) Fused filament 3D printing of ionic polymer-metal composites for soft robotics. Electroactive Polymer Actuators and …, spiedigitallibrary.org, cited by 6 (3.00 per year)
M Sajid, S Aziz, GB Kim, SW Kim, J Jo, KH Choi (2016) Bio-compatible organic humidity sensor transferred to arbitrary surfaces fabricated using single-cell-thick onion membrane as both the substrate and sensing …. Scientific reports, nature.com, cited by 13 (4.33 per year)
V Serpooshan, M Mahmoudi, DA Hu, ... (2017) Bioengineering cardiac constructs using 3D printing. Journal of 3D printing …, Future Medicine, cited by 11 (5.50 per year)
S Yin, Z Niu, X Chen (2012) Assembly of graphene sheets into 3D macroscopic structures. Small, Wiley Online Library, cited by 146 (20.86 per year)
L Lin, M Liu, L Chen, P Chen, J Ma, D Han, ... (2010) Bio-inspired hierarchical macromolecule–nanoclay hydrogels for robust underwater superoleophobicity. Advanced …, Wiley Online Library, cited by 193 (21.44 per year)
R Yadav, M Naebe, X Wang, ... (2017) Review on 3D prototyping of damage tolerant interdigitating brick arrays of nacre. Industrial & …, ACS Publications, cited by 9 (4.50 per year)
XG Zhao, KJ Hwang, D Lee, T Kim, N Kim (2018) Enhanced mechanical properties of self-polymerized polydopamine-coated recycled PLA filament used in 3D printing. Applied Surface Science, Elsevier, cited by 8 (8.00 per year)
A Dubois, C Farhat, AH Abukhwejah (2016) Parameterization Framework for Aeroelastic Design Optimization of Bio-Inspired Wing Structural Layout. 57th AIAA/ASCE/AHS/ASC …, arc.aiaa.org, cited by 5 (1.67 per year)
Y Kim, Y Kim, TI Lee, TS Kim, S Ryu (2018) An extended analytic model for the elastic properties of platelet-staggered composites and its application to 3D printed structures. Composite Structures, Elsevier, cited by 7 (7.00 per year)
H Lipson, M Kurman (2013) Fabricated: The new world of 3D printing., books.google.com, cited by 1058 (176.33 per year)
A Tabatabai, A Fassler, C Usiak, C Majidi (2013) Liquid-phase gallium–indium alloy electronics with microcontact printing. Langmuir, ACS Publications, cited by 175 (29.17 per year)
UK Roopavath, DM Kalaskar (2017) Introduction to 3D printing in medicine. 3D Printing in Medicine, Elsevier, cited by 6 (3.00 per year)
N Hu, P Feng, G Dai (2013) The gift from nature: bio-inspired strategy for developing innovative bridges. Journal of Bionic Engineering, Elsevier, cited by 19 (3.17 per year)
D Chen, Y Liu, H Chen, D Zhang (2018) Bio-inspired drag reduction surface from sharkskin. Biosurface and Biotribology, ieeexplore.ieee.org, cited by 3 (3.00 per year)
K Jakab, C Norotte, B Damon, F Marga, ... (2008) Tissue engineering by self-assembly of cells printed into topologically defined structures. … Engineering Part A, liebertpub.com, cited by 283 (25.73 per year)
S Yang, KF Leong, Z Du, CK Chua (2002) The design of scaffolds for use in tissue engineering. Part II. Rapid prototyping techniques. Tissue engineering, liebertpub.com, cited by 795 (46.76 per year)
H Kakisawa, T Sumitomo, R Inoue, Y Kagawa (2010) Fabrication of nature-inspired bulk laminar composites by a powder processing. Composites Science and …, Elsevier, cited by 24 (2.67 per year)
TAE Ahmed, EV Dare, M Hincke (2008) Fibrin: a versatile scaffold for tissue engineering applications. Tissue Engineering Part B …, liebertpub.com, cited by 731 (66.45 per year)

L Chen, X Tang, P Xie, J Xu, Z Chen, Z Cai, ... (2018) 3D Printing of Artificial Leaf with Tunable Hierarchical Porosity for CO2 Photoreduction. Chemistry of …, ACS Publications, cited by 16 (16.00 per year)
BP Cumming, GE Schröder-Turk, ... (2017) Bragg-mirror-like circular dichroism in bio-inspired quadruple-gyroid 4srs nanostructures. Light: Science & …, nature.com, cited by 15 (7.50 per year)
N Tan, RE Mohan, K Elangovan (2016) A bio-inspired reconfigurable robot. Advances in Reconfigurable …, Springer, cited by 10 (3.33 per year)
X Zhou, B Guo, L Zhang, GH Hu (2017) Progress in bio-inspired sacrificial bonds in artificial polymeric materials. Chemical Society Reviews, pubs.rsc.org, cited by 23 (11.50 per year)
R Liska, M Schuster, R InfŘhr, C Turecek, ... (2007) Photopolymers for rapid prototyping. Journal of Coatings …, Springer, cited by 146 (12.17 per year)
Q Gu, E Tomaskovic-Crook, R Lozano, ... (2016) Functional 3D neural mini-tissues from printed gel-based bioink and human neural stem cells. Advanced …, Wiley Online Library, cited by 81 (27.00 per year)
B Duan, LA Hockaday, KH Kang, ... (2013) 3D bioprinting of heterogeneous aortic valve conduits with alginate/gelatin hydrogels. Journal of biomedical …, Wiley Online Library, cited by 487 (81.17 per year)
L Ren, X Zhou, Q Liu, Y Liang, Z Song, ... (2018) 3D magnetic printing of bio-inspired composites with tunable mechanical properties. Journal of materials …, Springer, cited by 2 (2.00 per year)
J Choi, OC Kwon, W Jo, HJ Lee, ... (2015) 4D printing technology: A review. 3D Printing and Additive …, liebertpub.com, cited by 60 (15.00 per year)
R Matsuzaki, M Ueda, M Namiki, TK Jeong, ... (2016) Three-dimensional printing of continuous-fiber composites by in-nozzle impregnation. Scientific reports, nature.com, cited by 217 (72.33 per year)
MS Mannoor, Z Jiang, T James, YL Kong, ... (2013) 3D printed bionic ears. Nano …, ACS Publications, cited by 509 (84.83 per year)
S Xia, Z Wang, H Chen, W Fu, J Wang, Z Li, L Jiang (2015) Nanoasperity: structure origin of nacre-inspired nanocomposites. ACS nano, ACS Publications, cited by 38 (9.50 per year)
MN Cooke, JP Fisher, D Dean, ... (2003) Use of stereolithography to manufacture critical-sized 3D biodegradable scaffolds for bone ingrowth. Journal of Biomedical …, Wiley Online Library, cited by 518 (32.38 per year)
ME Staymates, WA MacCrehan, JL Staymates, ... (2016) Biomimetic sniffing improves the detection performance of a 3D printed nose of a dog and a commercial trace vapor detector. Scientific reports, nature.com, cited by 19 (6.33 per year)
C Majidi (2014) Soft robotics: a perspective—current trends and prospects for the future. Soft Robotics, liebertpub.com, cited by 510 (102.00 per year)
J Vanderburgh, JA Sterling, SA Guelcher (2017) 3D Printing of Tissue Engineered Constructs for In Vitro Modeling of Disease Progression and Drug Screening. Annals of biomedical …, Springer, cited by 44 (22.00 per year)
LE Freed, F Guilak, XE Guo, ML Gray, ... (2006) Advanced tools for tissue engineering: scaffolds, bioreactors, and signaling. Tissue …, liebertpub.com, cited by 291 (22.38 per year)
LE Murr, LE Murr (2015) Biomimetics and biologically inspired materials. … of materials structures, properties, processing and …, Springer, cited by 11 (2.75 per year)

J Maurath, N Willenbacher (2017) 3D printing of open-porous cellular ceramics with high specific strength. Journal of the European Ceramic Society, Elsevier, cited by 24 (12.00 per year)
M Lapeyre, P Rouanet, ... (2013) Poppy humanoid platform: Experimental evaluation of the role of a bio-inspired thigh shape. 2013 13th IEEE-RAS …, ieeexplore.ieee.org, cited by 10 (1.67 per year)
X Zhou, T Li, J Wang, F Chen, D Zhou, ... (2018) Mechanochemical regulated origami with tough hydrogels by ion transfer printing. … applied materials & …, ACS Publications, cited by 16 (16.00 per year)
JY Lim, HJ Donahue (2007) Cell sensing and response to micro-and nanostructured surfaces produced by chemical and topographic patterning. Tissue engineering, liebertpub.com, cited by 484 (40.33 per year)
K Markstedt, A Mantas, I Tournier, ... (2015) 3D bioprinting human chondrocytes with nanocellulose–alginate bioink for cartilage tissue engineering applications. …, ACS Publications, cited by 415 (103.75 per year)
J He, M Mao, Y Liu, J Shao, Z Jin, ... (2013) Fabrication of nature-inspired microfluidic network for perfusable tissue constructs. Advanced healthcare …, Wiley Online Library, cited by 45 (7.50 per year)
Z Song, C Mazzola, E Schwartz, R Chen, ... (2016) A compact autonomous underwater vehicle with cephalopod-inspired propulsion. Marine Technology …, ingentaconnect.com, cited by 14 (4.67 per year)
AI Mainong, AF Ayob, MR Arshad (2017) Investigating pectoral shapes and locomotive strategies for conceptual designing bio-inspired robotic fish. Journal of Engineering Science …, eprints.usm.my, cited by 3 (1.50 per year)
S Pedron, E Becka, BA Harley (2015) Spatially gradated hydrogel platform as a 3D engineered tumor microenvironment. Advanced Materials, Wiley Online Library, cited by 61 (15.25 per year)
Y He, J Qiu, J Fu, J Zhang, Y Ren, A Liu (2015) Printing 3D microfluidic chips with a 3D sugar printer. Microfluidics and Nanofluidics, Springer, cited by 50 (12.50 per year)
A Chiappone, E Fantino, I Roppolo, ... (2016) 3D printed PEG-based hybrid nanocomposites obtained by sol–gel technique. … applied materials & …, ACS Publications, cited by 30 (10.00 per year)
B Duan (2017) State-of-the-art review of 3D bioprinting for cardiovascular tissue engineering. Annals of biomedical engineering, Springer, cited by 99 (49.50 per year)
MA Khan, S Kumar (2018) Performance enhancement of tubular multilayers via compliance-tailoring: 3D printing, testing and modeling. International Journal of Mechanical Sciences, Elsevier, cited by 7 (7.00 per year)
G Liu, Y Zhao, G Wu, J Lu (2018) Origami and 4D printing of elastomer-derived ceramic structures. Science advances, advances.sciencemag.org, cited by 9 (9.00 per year)
GX Gu, M Takaffoli, AJ Hsieh, MJ Buehler (2016) Biomimetic additive manufactured polymer composites for improved impact resistance. Extreme Mechanics Letters, Elsevier, cited by 69 (23.00 per year)
SE Hudson (2014) Printing teddy bears: a technique for 3D printing of soft interactive objects. Proceedings of the SIGCHI Conference on Human …, dl.acm.org, cited by 91 (18.20 per year)
MF Stoelen, F Bonsignorio, A Cangelosi (2016) Co-exploring actuator antagonism and bio-inspired control in a printable robot arm. International Conference on …, Springer, cited by 6 (2.00 per year)

W Hao, Y Liu, H Zhou, H Chen, D Fang (2018) Preparation and characterization of 3D printed continuous carbon fiber reinforced thermosetting composites. Polymer Testing, Elsevier, cited by 34 (34.00 per year)
JJ Adams, EB Duoss, TF Malkowski, ... (2011) Conformal printing of electrically small antennas on three-dimensional surfaces. Advanced …, Wiley Online Library, cited by 370 (46.25 per year)
Y Cai, L Lin, Z Xue, M Liu, S Wang, ... (2014) Filefish-inspired surface design for anisotropic underwater oleophobicity. Advanced Functional …, Wiley Online Library, cited by 120 (24.00 per year)
R Bauer, Y Zhang, JC Jackson, ... (2016) Housing influence on multi-band directional MEMS microphones inspired by Ormia ochracea. 2016 IEEE …, ieeexplore.ieee.org, cited by 4 (1.33 per year)
L Li, B Yan, J Yang, L Chen, H Zeng (2015) Novel mussel-inspired injectable self-healing hydrogel with anti-biofouling property. Advanced Materials, Wiley Online Library, cited by 203 (50.75 per year)
AE Vellwock, L Vergani, F Libonati (2018) A multiscale XFEM approach to investigate the fracture behavior of bio-inspired composite materials. Composites Part B: Engineering, Elsevier, cited by 3 (3.00 per year)
L Wang, J Lau, EL Thomas, MC Boyce (2011) Co-continuous composite materials for stiffness, strength, and energy dissipation. Advanced Materials, Wiley Online Library, cited by 139 (17.38 per year)
A Miriyev, K Stack, H Lipson (2017) Soft material for soft actuators. Nature communications, nature.com, cited by 102 (51.00 per year)
Z Wang, P Huang, O Jacobson, Z Wang, Y Liu, L Lin, ... (2016) Biomineralization-inspired synthesis of copper sulfide–ferritin nanocages as cancer theranostics. ACS …, ACS Publications, cited by 137 (45.67 per year)
AA Pawar, G Saada, I Cooperstein, ... (2016) High-performance 3D printing of hydrogels by water-dispersible photoinitiator nanoparticles. Science …, advances.sciencemag.org, cited by 51 (17.00 per year)
A Qureshi, B Li, KT Tan (2016) Numerical investigation of band gaps in 3D printed cantilever-in-mass metamaterials. Scientific reports, nature.com, cited by 26 (8.67 per year)
R Yadav, R Goud, A Dutta, X Wang, ... (2018) Biomimicking of Hierarchal Molluscan Shell Structure Via Layer by Layer 3D Printing. Industrial & …, ACS Publications, cited by 4 (4.00 per year)
SN Jayasinghe, J Auguste, CJ Scotton (2015) Platform technologies for directly reconstructing 3D living biomaterials. Advanced Materials, Wiley Online Library, cited by 12 (3.00 per year)
SAN Gowers, VF Curto, CA Seneci, C Wang, ... (2015) 3D printed microfluidic device with integrated biosensors for online analysis of subcutaneous human microdialysate. Analytical …, ACS Publications, cited by 81 (20.25 per year)
MW Mosesson, KR Siebenlist, ... (2001) The structure and biological features of fibrinogen and fibrin. Annals of the New York …, Wiley Online Library, cited by 632 (35.11 per year)
F Narducci, ST Pinho (2017) Exploiting nacre-inspired crack deflection mechanisms in CFRP via micro-structural design. Composites Science and Technology, Elsevier, cited by 10 (5.00 per year)
G Haghiashtiani, E Habtour, SH Park, F Gardea, ... (2018) 3D printed electrically-driven soft actuators. Extreme Mechanics …, Elsevier, cited by 9 (9.00 per year)

AV Do, R Smith, TM Acri, SM Geary, ... (2018) 3D printing technologies for 3D scaffold engineering. Functional 3D Tissue …, Elsevier, cited by 5 (5.00 per year)
C Pouya, JTB Overvelde, M Kolle, ... (2016) Characterization of a Mechanically Tunable Gyroid Photonic Crystal Inspired by the Butterfly Parides Sesostris. Advanced Optical …, Wiley Online Library, cited by 14 (4.67 per year)
A Le Duigou, S Requile, J Beaugrand, ... (2017) Natural fibres actuators for smart bio-inspired hygromorph biocomposites. Smart Materials and …, iopscience.iop.org, cited by 14 (7.00 per year)
Y Huang, F Li, M Qin, L Jiang, ... (2013) A Multi-stopband Photonic-Crystal Microchip for High-Performance Metal-Ion Recognition Based on Fluorescent Detection. Angewandte Chemie …, Wiley Online Library, cited by 92 (15.33 per year)
C Xu, R Inai, M Kotaki, S Ramakrishna (2004) Electrospun nanofiber fabrication as synthetic extracellular matrix and its potential for vascular tissue engineering. Tissue engineering, liebertpub.com, cited by 375 (25.00 per year)
SH Ku, JS Lee, CB Park (2010) Spatial control of cell adhesion and patterning through mussel-inspired surface modification by polydopamine. Langmuir, ACS Publications, cited by 171 (19.00 per year)
JP Carrel, A Wiskott, M Moussa, ... (2016) A 3D printed TCP/HA structure as a new osteoconductive scaffold for vertical bone augmentation. Clinical oral implants …, Wiley Online Library, cited by 29 (9.67 per year)
J Li, F Ye, S Vaziri, M Muhammed, ... (2013) Efficient inkjet printing of graphene. Advanced …, Wiley Online Library, cited by 262 (43.67 per year)
T Umedachi, V Vikas, BA Trimmer (2016) Softworms: the design and control of non-pneumatic, 3D-printed, deformable robots. Bioinspiration & biomimetics, iopscience.iop.org, cited by 77 (25.67 per year)
SM Chin, CV Synatschke, S Liu, RJ Nap, ... (2018) Covalent-supramolecular hybrid polymers as muscle-inspired anisotropic actuators. Nature …, nature.com, cited by 14 (14.00 per year)
GD Nicodemus, SJ Bryant (2008) Cell encapsulation in biodegradable hydrogels for tissue engineering applications. Tissue Engineering Part B: Reviews, liebertpub.com, cited by 906 (82.36 per year)
X Yang, G Li, T Cheng, Q Zhao, ... (2016) Bio-Inspired fast actuation by mechanical instability of thermoresponding hydrogel structures. Journal of …, … .asmedigitalcollection.asme.org, cited by 7 (2.33 per year)
AG EVans, EA Charles (1976) Fracture toughness determinations by indentation. Journal of the American Ceramic …, Wiley Online Library, cited by 2109 (49.05 per year)
BM Boyle, TA French, RM Pearson, BG McCarthy, ... (2017) Structural color for additive manufacturing: 3D-printed photonic crystals from block copolymers. ACS …, ACS Publications, cited by 53 (26.50 per year)
A Cheng, DJ Cohen, BD Boyan, Z Schwartz (2016) Laser-sintered constructs with bio-inspired porosity and surface micro/nano-roughness enhance mesenchymal stem cell differentiation and matrix …. Calcified tissue international, Springer, cited by 9 (3.00 per year)
N Guo, MC Leu (2013) Additive manufacturing: technology, applications and research needs. Frontiers of Mechanical Engineering, Springer, cited by 766 (127.67 per year)
K Zolotovsky (2012) BioConstructs: methods for bio-inspired and bio-fabricated design., dspace.mit.edu, cited by 7 (1.00 per year)

JM Taboas, RD Maddox, PH Krebsbach, SJ Hollister (2003) Indirect solid free form fabrication of local and global porous, biomimetic and composite 3D polymer-ceramic scaffolds. Biomaterials, Elsevier, cited by 723 (45.19 per year)
AD Marchese, CD Onal, D Rus (2014) Autonomous soft robotic fish capable of escape maneuvers using fluidic elastomer actuators. Soft Robotics, liebertpub.com, cited by 336 (67.20 per year)
DB Burckel, JR Wendt, GA Ten Eyck, ... (2010) Fabrication of 3D Metamaterial Resonators Using Self-Aligned Membrane Projection Lithography. Advanced …, Wiley Online Library, cited by 62 (6.89 per year)
HW Chien, WH Kuo, MJ Wang, SW Tsai, WB Tsai (2012) Tunable micropatterned substrates based on poly (dopamine) deposition via microcontact printing. Langmuir, ACS Publications, cited by 106 (15.14 per year)
UGK Wegst, H Bai, E Saiz, AP Tomsia, RO Ritchie (2015) Bioinspired structural materials. Nature materials, nature.com, cited by 1329 (332.25 per year)
Q Fu, E Saiz, AP Tomsia (2011) Bioinspired strong and highly porous glass scaffolds. Advanced functional materials, Wiley Online Library, cited by 184 (23.00 per year)
Y Jiang, Q Wang (2016) Highly-stretchable 3D-architected mechanical metamaterials. Scientific reports, nature.com, cited by 44 (14.67 per year)
X Hou, Y Hu, A Grinthal, M Khan, J Aizenberg (2015) Liquid-based gating mechanism with tunable multiphase selectivity and antifouling behaviour. Nature, nature.com, cited by 175 (43.75 per year)
L Zhao, G Zeng, Y Gu, Z Tang, G Wang, T Tang, ... (2019) Nature inspired fractal tree-like photobioreactor via 3D printing for CO2 capture by microaglae. Chemical Engineering …, Elsevier, cited by 2 (2.00 per year)
W Li, F Li, H Li, M Su, M Gao, Y Li, D Su, ... (2016) Flexible circuits and soft actuators by printing assembly of graphene. … applied materials & …, ACS Publications, cited by 50 (16.67 per year)
X Ma, X Qu, W Zhu, YS Li, S Yuan, ... (2016) Deterministically patterned biomimetic human iPSC-derived hepatic model via rapid 3D bioprinting. Proceedings of the …, National Acad Sciences, cited by 206 (68.67 per year)
W Zhang, Q Lian, D Li, K Wang, D Hao, ... (2014) Cartilage repair and subchondral bone migration using 3D printing osteochondral composites: a one-year-period study in rabbit trochlea. BioMed research …, hindawi.com, cited by 61 (12.20 per year)
X Wang, H Hu, Y Shen, X Zhou, Z Zheng (2011) Stretchable conductors with ultrahigh tensile strain and stable metallic conductance enabled by prestrained polyelectrolyte nanoplatforms. Advanced Materials, Wiley Online Library, cited by 159 (19.88 per year)
Y Li, T Gao, Z Yang, C Chen, W Luo, J Song, ... (2017) 3D-printed, all-in-one evaporator for high-efficiency solar steam generation under 1 sun illumination. Advanced …, Wiley Online Library, cited by 147 (73.50 per year)
L Liu, Y Li (2018) Failure mechanism transition of 3D-printed biomimetic sutures. Engineering Fracture Mechanics, Elsevier, cited by 4 (4.00 per year)
YJ Zhao, XW Zhao, J Hu, J Li, WY Xu, ... (2009) Multiplex label-free detection of biomolecules with an imprinted suspension array. Angewandte Chemie …, Wiley Online Library, cited by 161 (16.10 per year)
Y Xu, X Wang (2015) Application of 3D biomimetic models in drug delivery and regenerative medicine. Current pharmaceutical design, ingentaconnect.com, cited by 28 (7.00 per year)

R Bauer, Y Zhang, JC Jackson, ... (2017) Influence of Microphone Housing on the Directional Response of Piezoelectric MEMS Microphones Inspired by Ormia Ochracea. IEEE Sensors …, ieeexplore.ieee.org, cited by 6 (3.00 per year)
J Lee, MJ Cuddihy, NA Kotov (2008) Three-dimensional cell culture matrices: state of the art. Tissue Engineering Part B: Reviews, liebertpub.com, cited by 832 (75.64 per year)
C Li, N Li, X Zhang, Z Dong, H Chen, ... (2016) Uni-Directional Transportation on Peristome-Mimetic Surfaces for Completely Wetting Liquids. Angewandte Chemie …, Wiley Online Library, cited by 44 (14.67 per year)
NE Fedorovich, W Schuurman, ... (2011) Biofabrication of osteochondral tissue equivalents by printing topologically defined, cell-laden hydrogel scaffolds. … Engineering Part C …, liebertpub.com, cited by 264 (33.00 per year)
KS Toohey, NR Sottos, JA Lewis, JS Moore, ... (2007) Self-healing materials with microvascular networks. Nature materials, nature.com, cited by 1085 (90.42 per year)
X Cui, K Breitenkamp, MG Finn, M Lotz, ... (2012) Direct human cartilage repair using three-dimensional bioprinting technology. … Engineering Part A, liebertpub.com, cited by 362 (51.71 per year)
Q Zhu, Q Pan (2014) Mussel-inspired direct immobilization of nanoparticles and application for oil–water separation. ACS nano, ACS Publications, cited by 219 (43.80 per year)
S Ishutov, FJ Hasiuk, C Harding, JN Gray (2015) 3D printing sandstone porosity models. Interpretation, library.seg.org, cited by 35 (8.75 per year)
M Li, F He, Q Liao, J Liu, L Xu, L Jiang, ... (2008) Ultrasensitive DNA detection using photonic crystals. Angewandte Chemie …, Wiley Online Library, cited by 144 (13.09 per year)
E Fantino, A Chiappone, F Calignano, M Fontana, ... (2016) In situ thermal generation of silver nanoparticles in 3D printed polymeric structures. Materials, mdpi.com, cited by 22 (7.33 per year)
X Li, ZH Xu, R Wang (2006) In situ observation of nanograin rotation and deformation in nacre. Nano letters, ACS Publications, cited by 228 (17.54 per year)
M Tieu, DM Michael, JB Pflueger, ... (2016) Demonstrations of bio-inspired perching landing gear for UAVs. Bioinspiration …, spiedigitallibrary.org, cited by 2 (0.67 per year)
B Li, T Du, B Yu, J van der Gucht, F Zhou (2015) Caterpillar-inspired design and fabrication of a self-walking actuator with anisotropy, gradient, and instant response. Small, Wiley Online Library, cited by 31 (7.75 per year)
F Libonati, V Cipriano, L Vergani, ... (2017) Computational framework to predict failure and performance of bone-inspired materials. … Biomaterials Science & …, ACS Publications, cited by 4 (2.00 per year)
C Ma, T Li, Q Zhao, X Yang, J Wu, Y Luo, ... (2014) Supramolecular Lego Assembly Towards Three-Dimensional Multi-Responsive Hydrogels. Advanced …, Wiley Online Library, cited by 108 (21.60 per year)
M Malinauskas, A Žukauskas, S Hasegawa, ... (2016) Ultrafast laser processing of materials: from science to industry. Light: Science & …, nature.com, cited by 330 (110.00 per year)
L Shang, W Zhang, K Xu, Y Zhao (2019) Bio-inspired intelligent structural color materials. Materials Horizons, pubs.rsc.org, cited by 2 (2.00 per year)
J Wang, R Rai (2016) Classification of Bio-Inspired Periodic Cubic Cellular Materials Based on Compressive Deformation Behaviors of 3D Printed Parts and FE Simulations. ASME 2016 International …, … .asmedigitalcollection.asme.org, cited by 2 (0.67 per year)
B Yang, J Yin, Y Chen, S Pan, H Yao, ... (2018) 2D-Black-Phosphorus-Reinforced 3D-Printed Scaffolds: A Stepwise Countermeasure for Osteosarcoma. Advanced …, Wiley Online Library, cited by 41 (41.00 per year)

J Aizenberg, P Fratzl (2013) New materials through bioinspiration and nanoscience. Advanced Functional …, aizenberglab.seas.harvard.edu, cited by 19 (3.17 per year)
HA Bruck, AL Gershon, ... (2004) Enhancement of mechanical engineering curriculum to introduce manufacturing techniques and principles for bio-inspired product development. ASME 2004 …, … .asmedigitalcollection.asme.org, cited by 11 (0.73 per year)
B Gao, Q Yang, X Zhao, G Jin, Y Ma, F Xu (2016) 4D bioprinting for biomedical applications. Trends in biotechnology, Elsevier, cited by 139 (46.33 per year)
J Ryu, SH Ku, H Lee, CB Park (2010) Mussel-inspired polydopamine coating as a universal route to hydroxyapatite crystallization. Advanced Functional Materials, Wiley Online Library, cited by 477 (53.00 per year)
Z Pan, F Cheng, B Zhao (2017) Bio-inspired polymeric structures with special wettability and their applications: an overview. Polymers, mdpi.com, cited by 6 (3.00 per year)
J Nam, Y Huang, S Agarwal, J Lannutti (2007) Improved cellular infiltration in electrospun fiber via engineered porosity. Tissue engineering, liebertpub.com, cited by 354 (29.50 per year)
AP Esser-Kahn, PR Thakre, H Dong, ... (2011) Three-dimensional microvascular fiber-reinforced composites. Advanced …, Wiley Online Library, cited by 199 (24.88 per year)
Y Liu, K He, G Chen, WR Leow, X Chen (2017) Nature-inspired structural materials for flexible electronic devices. Chemical reviews, ACS Publications, cited by 114 (57.00 per year)
J Auerbach, D Aydin, A Maesani, ... (2014) Robogen: Robot generation through artificial evolution. Artificial Life …, MIT Press, cited by 37 (7.40 per year)
C Chen, S Bang, Y Cho, S Lee, ... (2016) Research trends in biomimetic medical materials for tissue engineering: 3D bioprinting, surface modification, nano/micro-technology and clinical aspects in …. Biomaterials …, biomaterialsres.biomedcentral.com, cited by 31 (10.33 per year)
L Yao, R Niiyama, J Ou, S Follmer, ... (2013) PneUI: pneumatically actuated soft composite materials for shape changing interfaces. Proceedings of the 26th …, dl.acm.org, cited by 98 (16.33 per year)
E Kanhere, N Wang, M Asadnia, ... (2015) Crocodile inspired Dome Pressure sensor for hydrodynamic sensing. … Conference on Solid …, ieeexplore.ieee.org, cited by 4 (1.00 per year)
F Marga, A Neagu, I Kosztin, ... (2007) Developmental biology and tissue engineering. Birth Defects Research …, Wiley Online Library, cited by 98 (8.17 per year)
H Wang, Y Yang, L Guo (2017) Nature-Inspired Electrochemical Energy-Storage Materials and Devices. Advanced Energy Materials, Wiley Online Library, cited by 51 (25.50 per year)
Y Zhang (2017) Post-3D printing modification for improved biomedical applications., dr.ntu.edu.sg, cited by 2 (1.00 per year)
AS Mathews, S Abraham, SK Kumaran, J Fan, ... (2017) Bio nano ink for 4D printing membrane proteins. RSC Advances, pubs.rsc.org, cited by 2 (1.00 per year)
Z Han, L Liu, K Wang, H Song, D Chen, Z Wang, ... (2018) Artificial hair-like sensors inspired from nature: A review. Journal of Bionic …, Springer, cited by 5 (5.00 per year)
J Xu, Y Gao, H Huang, Q Yang, L Guo, L Jiang (2016) Diamond-structured hollow-tube lattice Ni materials via 3D printing. Science China Chemistry, Springer, cited by 2 (0.67 per year)
N Vargas-Alfredo, A Dorronsoro, ... (2017) Antimicrobial 3D porous scaffolds prepared by additive manufacturing and breath figures. … applied materials & …, ACS Publications, cited by 11 (5.50 per year)
F Ilievski, AD Mazzeo, RF Shepherd, ... (2011) Soft robotics for chemists. Angewandte Chemie …, Wiley Online Library, cited by 930 (116.25 per year)

S Shan, SH Kang, JR Raney, P Wang, ... (2015) Multistable architected materials for trapping elastic strain energy. Advanced …, Wiley Online Library, cited by 198 (49.50 per year)
A Sadeghi, A Mondini, B Mazzolai (2017) Toward self-growing soft robots inspired by plant roots and based on additive manufacturing technologies. Soft robotics, liebertpub.com, cited by 25 (12.50 per year)
S Rudykh, MC Boyce (2014) Analysis of elasmoid fish imbricated layered scale-tissue systems and their bio-inspired analogues at finite strains and bending. IMA Journal of Applied Mathematics, ieeexplore.ieee.org, cited by 24 (4.80 per year)
T Qu, J Chen, S Shen, Z Xiao, Z Yue, ... (2016) Motion control of a bio-inspired wire-driven multi-backbone continuum minimally invasive surgical manipulator. … on Robotics and …, ieeexplore.ieee.org, cited by 4 (1.33 per year)
J Hildebrandt, A Bezama, ... (2017) Cascade use indicators for selected biopolymers: are we aiming for the right solutions in the design for recycling of bio-based polymers?. Waste Management & …, journals.sagepub.com, cited by 12 (6.00 per year)
D Rus, MT Tolley (2015) Design, fabrication and control of soft robots. Nature, nature.com, cited by 1289 (322.25 per year)
B Tiller, A Reid, B Zhu, J Guerreiro, R Domingo-Roca, ... (2019) Piezoelectric microphone via a digital light processing 3D printing process. Materials & Design, Elsevier, cited by 2 (2.00 per year)
F Momeni, J Ni (2018) Nature-inspired smart solar concentrators by 4D printing. Renewable energy, Elsevier, cited by 7 (7.00 per year)
W Jamróz, J Szafraniec, M Kurek, R Jachowicz (2018) 3D printing in pharmaceutical and medical applications–recent achievements and challenges. Pharmaceutical research, Springer, cited by 22 (22.00 per year)
GX Gu, M Takaffoli, MJ Buehler (2017) Hierarchically enhanced impact resistance of bioinspired composites. Advanced Materials, Wiley Online Library, cited by 55 (27.50 per year)
AG Mikos, SW Herring, P Ochareon, J Elisseeff, ... (2006) Engineering complex tissues. Tissue …, liebertpub.com, cited by 517 (39.77 per year)
LM Bellan, T Kniazeva, ES Kim, ... (2012) Fabrication of a Hybrid Microfluidic System Incorporating both Lithographically Patterned Microchannels and a 3D Fiber-Formed Microfluidic Network. Advanced …, Wiley Online Library, cited by 22 (3.14 per year)
J Park, Y Lee, J Hong, Y Lee, M Ha, Y Jung, H Lim, ... (2014) Tactile-direction-sensitive and stretchable electronic skins based on human-skin-inspired interlocked microstructures. ACS …, ACS Publications, cited by 205 (41.00 per year)
W Crooks, G Vukasin, M O'Sullivan, ... (2016) Fin ray® effect inspired soft robotic gripper: From the robosoft grand challenge toward optimization. Frontiers in Robotics …, frontiersin.org, cited by 20 (6.67 per year)
H Le Ferrand, F Bouville (2018) Processing of dense bio-inspired ceramics with deliberate microtexture. Journal of the American Ceramic …, Wiley Online Library, cited by 2 (2.00 per year)
L Shi, H Carstensen, K Hölzl, M Lunzer, ... (2017) Dynamic coordination chemistry enables free directional printing of biopolymer hydrogel. Chemistry of …, ACS Publications, cited by 23 (11.50 per year)
MK Hausmann, PA Ruhs, G Siqueira, J Läuger, ... (2018) Dynamics of cellulose nanocrystal alignment during 3D printing. ACS …, ACS Publications, cited by 20 (20.00 per year)
R Domingo-Roca, JC Jackson, ... (2017) Bioinspired 3D-printed piezoelectric device for acoustic frequency separation. 2017 IEEE …, ieeexplore.ieee.org, cited by 2 (1.00 per year)

A Radke, T Gissibl, T Klotzbücher, PV Braun, ... (2011) Three-dimensional bichiral plasmonic crystals fabricated by direct laser writing and electroless silver plating. Advanced …, Wiley Online Library, cited by 166 (20.75 per year)
C Liu, N Huang, F Xu, J Tong, Z Chen, X Gui, Y Fu, ... (2018) 3D printing technologies for flexible tactile sensors toward wearable electronics and electronic skin. Polymers, mdpi.com, cited by 14 (14.00 per year)
C Zhang, W Wang, YC Li, YG Yang, Y Wu, ... (2018) 3D printing of Fe-based bulk metallic glasses and composites with large dimensions and enhanced toughness by thermal spraying. Journal of Materials …, pubs.rsc.org, cited by 14 (14.00 per year)
D Rosen (2014) Design for additive manufacturing: Past, present, and future directions. Journal of Mechanical Design, … .asmedigitalcollection.asme.org, cited by 39 (7.80 per year)
AA Zadpoor, J Malda (2017) Additive manufacturing of biomaterials, tissues, and organs., Springer, cited by 84 (42.00 per year)
AR Studart (2013) Biological and bioinspired composites with spatially tunable heterogeneous architectures. Advanced Functional Materials, Wiley Online Library, cited by 83 (13.83 per year)
H Zheng, S SCHLEICHER (2018) Bio-Inspired 3D Printing Experiments. CAADRIA 2018, researchgate.net, cited by 1 (1.00 per year)
E Salami, PB Ganesan, TA Ward, ... (2016) Design and mechanical analysis of a 3D-printed biodegradable biomimetic micro air vehicle wing. IOP Conference …, iopscience.iop.org, cited by 2 (0.67 per year)
JC André (2017) From Additive Manufacturing to 3D/4D Printing: Breakthrough Innovations: Programmable Material, 4D Printing and Bio-printing., John Wiley & Sons, cited by 4 (2.00 per year)
N Lu, DH Kim (2014) Flexible and stretchable electronics paving the way for soft robotics. Soft Robotics, liebertpub.com, cited by 204 (40.80 per year)
G Cai, J Wang, K Qian, J Chen, S Li, ... (2017) Extremely stretchable strain sensors based on conductive self-healing dynamic cross-links hydrogels for human-motion detection. Advanced …, Wiley Online Library, cited by 150 (75.00 per year)
Y Wu, Y Huang, H Ma (2007) A facile method for permanent and functional surface modification of poly (dimethylsiloxane). Journal of the American Chemical Society, ACS Publications, cited by 88 (7.33 per year)
GK Lau, M Shrestha (2017) Ink-jet printing of micro-electro-mechanical systems (MEMS). Micromachines, mdpi.com, cited by 14 (7.00 per year)
Y Liu, W Xiong, LJ Jiang, YS Zhou, ... (2016) Precise 3D printing of micro/nanostructures using highly conductive carbon nanotube-thiol-acrylate composites. … 3D Manufacturing III, spiedigitallibrary.org, cited by 2 (0.67 per year)
EESC Lin (2015) Bio-inspired design of geometrically-structured suture interfaces and composites., dspace.mit.edu, cited by 3 (0.75 per year)
H Zeng, OM Wani, P Wasylczyk, ... (2018) Light-driven, caterpillar-inspired miniature inching robot. Macromolecular rapid …, Wiley Online Library, cited by 43 (43.00 per year)
M Tavakoli, A Sayuk, J Lourenço, P Neto (2017) Anthropomorphic finger for grasping applications: 3D printed endoskeleton in a soft skin. The International Journal of …, Springer, cited by 8 (4.00 per year)
P Kodati, X Deng (2009) Bio-inspired Robotic Fish with Multiple Fins. Underwater Vehicles, books.google.com, cited by 3 (0.30 per year)

N Hanauer, PL Latreille, S Alsharif, ... (2015) 2D, 3D and 4D active compound delivery in tissue engineering and regenerative medicine. Current pharmaceutical …, ingentaconnect.com, cited by 10 (2.50 per year)
W Wu, L Geng, Y Niu, D Qi, X Cui, D Fang (2018) Compression twist deformation of novel tetrachiral architected cylindrical tube inspired by towel gourd tendrils. Extreme Mechanics Letters, Elsevier, cited by 11 (11.00 per year)
B Mosadegh, P Polygerinos, ... (2014) Pneumatic networks for soft robotics that actuate rapidly. Advanced functional …, Wiley Online Library, cited by 468 (93.60 per year)
J Heo, T Kang, SG Jang, DS Hwang, ... (2012) Improved performance of protected catecholic polysiloxanes for bioinspired wet adhesion to surface oxides. Journal of the …, ACS Publications, cited by 78 (11.14 per year)
Z Xia, S Jin, K Ye (2018) Tissue and organ 3D bioprinting. SLAS TECHNOLOGY: Translating Life …, journals.sagepub.com, cited by 11 (11.00 per year)
AMS Ibrahim, RR Jose, AN Rabie, ... (2015) Three-dimensional printing in developing countries. … surgery Global open, ncbi.nlm.nih.gov, cited by 32 (8.00 per year)
MM Khoshhesab, Y Li (2018) Mechanical behavior of 3D printed biomimetic Koch fractal contact and interlocking. Extreme Mechanics Letters, Elsevier, cited by 3 (3.00 per year)
JK Carrow, AK Gaharwar (2015) Bioinspired polymeric nanocomposites for regenerative medicine. Macromolecular Chemistry and …, Wiley Online Library, cited by 73 (18.25 per year)
V Magdanz, G Stoychev, L Ionov, ... (2014) Stimuli-Responsive Microjets with Reconfigurable Shape. Angewandte Chemie …, Wiley Online Library, cited by 109 (21.80 per year)
D Gur, B Leshem, M Pierantoni, V Farstey, ... (2015) Structural basis for the brilliant colors of the sapphirinid copepods. Journal of the …, ACS Publications, cited by 46 (11.50 per year)
C Li, L Wu, C Yu, Z Dong, L Jiang (2017) Peristome-Mimetic Curved Surface for Spontaneous and Directional Separation of Micro Water-in-Oil Drops. Angewandte Chemie, Wiley Online Library, cited by 22 (11.00 per year)
A Martinez, S Palumbo (2018) Anisotropic shear behavior of soil-structure interfaces: bio-inspiration from snake skin. IFCEE 2018, ascelibrary.org, cited by 2 (2.00 per year)
CT Nguyen, H Phung, H Jung, U Kim, ... (2015) Printable monolithic hexapod robot driven by soft actuator. … on Robotics and …, ieeexplore.ieee.org, cited by 22 (5.50 per year)
CM Donatelli, ZT Serlin, P Echols-Jones, ... (2017) Soft foam robot with caterpillar-inspired gait regimes for terrestrial locomotion. 2017 IEEE/RSJ …, ieeexplore.ieee.org, cited by 3 (1.50 per year)
J Rossiter, H Hauser (2016) Soft robotics—the next industrial revolution. IEEE Robot. Autom. Mag, research-information.bristol.ac.uk, cited by 28 (9.33 per year)
N Li, T Yang, P Yu, J Chang, L Zhao, ... (2018) Bio-inspired upper limb soft exoskeleton to reduce stroke-induced complications. Bioinspiration & …, iopscience.iop.org, cited by 2 (2.00 per year)
YC Li, YS Zhang, A Akpek, SR Shin, ... (2016) 4D bioprinting: the next-generation technology for biofabrication enabled by stimuli-responsive materials. …, iopscience.iop.org, cited by 92 (30.67 per year)
AI Neto, PA Levkin, JF Mano (2018) Patterned superhydrophobic surfaces to process and characterize biomaterials and 3D cell culture. Materials Horizons, pubs.rsc.org, cited by 14 (14.00 per year)
NJ Castro, R Patel, LG Zhang (2015) Design of a novel 3D printed bioactive nanocomposite scaffold for improved osteochondral regeneration. Cellular and molecular bioengineering, Springer, cited by 37 (9.25 per year)

MC Mulakkal, RS Trask, VP Ting, AM Seddon (2018) Responsive cellulose-hydrogel composite ink for 4D printing. Materials & Design, Elsevier, cited by 4 (4.00 per year)
C Lee, M Kim, YJ Kim, N Hong, S Ryu, HJ Kim, ... (2017) Soft robot review. International Journal of …, Springer, cited by 45 (22.50 per year)
S Shriyam, A Mishra, D Nayak, A Thakur (2014) Design, fabrication and gait planning of alligator-inspired robot. Int. J. Curr. Eng. Technol, academia.edu, cited by 3 (0.60 per year)
D Correa, A Papadopoulou, C Guberan, ... (2015) 3D-printed wood: programming hygroscopic material transformations. 3D Printing and …, liebertpub.com, cited by 40 (10.00 per year)
DD Allison, KJ Grande-Allen (2006) Hyaluronan: a powerful tissue engineering tool. Tissue engineering, liebertpub.com, cited by 332 (25.54 per year)
RD Sochol, E Sweet, CC Glick, SY Wu, C Yang, ... (2018) 3D printed microfluidics and microelectronics. Microelectronic …, Elsevier, cited by 31 (31.00 per year)
S Tsunenari, T Oya (2016) Method for evaluating mechanical characteristics of biological material for bio-inspired lightweight design. Computer-Aided Design and Applications, Taylor & Francis, cited by 3 (1.00 per year)
SI Rich, RJ Wood, C Majidi (2018) Untethered soft robotics. Nature Electronics, nature.com, cited by 71 (71.00 per year)
B Dhariwala, E Hunt, T Boland (2004) Rapid prototyping of tissue-engineering constructs, using photopolymerizable hydrogels and stereolithography. Tissue engineering, liebertpub.com, cited by 292 (19.47 per year)
Z Gong, Z Xie, X Yang, T Wang, ... (2016) Design, fabrication and kinematic modeling of a 3D-motion soft robotic arm. 2016 IEEE International …, ieeexplore.ieee.org, cited by 10 (3.33 per year)
A Basu, A Saha, C Goodman, ... (2017) Catalytically Initiated Gel-in-Gel Printing of Composite Hydrogels. … applied materials & …, ACS Publications, cited by 10 (5.00 per year)
JYH Fuh, J Sun, EQ Li, J Li, L Chang, GS Hong, ... (2015) Micro-and Bio-Rapid Prototyping Using Drop-On-Demand 3D Printing. Handbook of …, Springer, cited by 2 (0.50 per year)
J Hughes, U Culha, F Giardina, F Guenther, ... (2016) Soft manipulators and grippers: a review. Frontiers in Robotics …, frontiersin.org, cited by 71 (23.67 per year)
D Zhu, CF Ortega, R Motamedi, ... (2012) Structure and mechanical performance of a "modern" fish scale. Advanced …, Wiley Online Library, cited by 132 (18.86 per year)
JJ Kim, J Hong, SJ Lee (2017) Bio-inspired cab-roof fairing of heavy vehicles for enhancing drag reduction and driving stability. International Journal of Mechanical Sciences, Elsevier, cited by 5 (2.50 per year)
S Jung, JH Kim, J Kim, S Choi, J Lee, I Park, ... (2014) Reverse-micelle-induced porous pressure-sensitive rubber for wearable human–machine interfaces. Advanced …, Wiley Online Library, cited by 250 (50.00 per year)
D Costa, G Palmieri, MC Palpacelli, ... (2016) Design of a bio-inspired underwater vehicle. 2016 12th IEEE …, ieeexplore.ieee.org, cited by 3 (1.00 per year)
J Monahan, AA Gewirth, RG Nuzzo (2001) A method for filling complex polymeric microfluidic devices and arrays. Analytical chemistry, ACS Publications, cited by 166 (9.22 per year)
W Jiang, L Yan, H Ma, Y Fan, J Wang, M Feng, S Qu (2018) Electromagnetic wave absorption and compressive behavior of a three-dimensional metamaterial absorber based on 3D printed honeycomb. Scientific reports, nature.com, cited by 16 (16.00 per year)

SH Reichert (2010) Reverse engineering nature: design principles for flexible protection inspired by ancient fish armor of Polypteridae., dspace.mit.edu, cited by 14 (1.56 per year)
D Wu, JN Wang, LG Niu, XL Zhang, ... (2014) Bioinspired Fabrication of High‐Quality 3D Artificial Compound Eyes by Voxel‐Modulation Femtosecond Laser Writing for Distortion‐Free Wide‐Field‐of‐View …. Advanced Optical …, Wiley Online Library, cited by 56 (11.20 per year)
S Zhao, Y Chen, BP Partlow, AS Golding, P Tseng, ... (2016) Bio-functionalized silk hydrogel microfluidic systems. Biomaterials, Elsevier, cited by 29 (9.67 per year)
D Marbach, AJ Ijspeert (2004) Co-evolution of configuration and control for homogenous modular robots. … of the eighth conference on intelligent …, infoscience.epfl.ch, cited by 62 (4.13 per year)
MM Ribó, A Islam (2017) 3D Printing of Bio-inspired surfaces. Technical University of Denmark, core.ac.uk, cited by 1 (0.50 per year)
G Borghesan, G Palli, ... (2010) Design of tendon-driven robotic fingers: Modeling and control issues. 2010 IEEE International …, ieeexplore.ieee.org, cited by 30 (3.33 per year)
R Pfeifer, HG Marques, F Iida (2013) Soft robotics: the next generation of intelligent machines. Twenty-Third International Joint Conference …, aaai.org, cited by 31 (5.17 per year)
T Swetly, J Stampfl, G Kempf, RM Hucke, ... (2016) Bioinspired engineering polymers by voxel-based 3D-printing. …, degruyter.com, cited by 3 (1.00 per year)
P Phamduy, MA Vazquez, C Kim, V Mwaffo, ... (2017) Design and characterization of a miniature free-swimming robotic fish based on multi-material 3D printing. International Journal of …, Springer, cited by 7 (3.50 per year)
B Richter, V Hahn, S Bertels, TK Claus, ... (2017) Guiding cell attachment in 3D microscaffolds selectively functionalized with two distinct adhesion proteins. Advanced …, Wiley Online Library, cited by 54 (27.00 per year)
MT Tolley, RF Shepherd, B Mosadegh, KC Galloway, ... (2014) A resilient, untethered soft robot. Soft robotics, liebertpub.com, cited by 384 (76.80 per year)
Z Zhakypov, M Falahi, M Shah, ... (2015) The design and control of the multi-modal locomotion origami robot, Tribot. 2015 IEEE/RSJ …, ieeexplore.ieee.org, cited by 36 (9.00 per year)
JY Sun, C Keplinger, GM Whitesides, ... (2014) Ionic skin. Advanced …, Wiley Online Library, cited by 320 (64.00 per year)
H Maleki, S Montes, N Hayati-Roodbari, ... (2018) … , Thermally Insulating, and Fire Retardant Aerogels through Self-Assembling Silk Fibroin Biopolymers Inside a Silica Structure—An Approach towards 3D Printing of …. … applied materials & …, ACS Publications, cited by 10 (10.00 per year)
D Wu, JN Wang, SZ Wu, QD Chen, ... (2011) Three‐level biomimetic rice‐leaf surfaces with controllable anisotropic sliding. Advanced Functional …, Wiley Online Library, cited by 166 (20.75 per year)
HW Kim, BD McCloskey, TH Choi, C Lee, ... (2013) Oxygen concentration control of dopamine-induced high uniformity surface coating chemistry. … applied materials & …, ACS Publications, cited by 132 (22.00 per year)
Z Wang, Q Zhang, S Long, Y Luo, P Yu, ... (2018) Three-dimensional printing of polyaniline/reduced graphene oxide composite for high-performance planar supercapacitor. … applied materials & …, ACS Publications, cited by 25 (25.00 per year)
YL Kong, MK Gupta, BN Johnson, MC McAlpine (2016) 3D printed bionic nanodevices. Nano Today, Elsevier, cited by 45 (15.00 per year)

Y Hao, Z Gong, Z Xie, S Guan, X Yang, ... (2016) Universal soft pneumatic robotic gripper with variable effective length. 2016 35th Chinese …, ieeexplore.ieee.org, cited by 36 (12.00 per year)
S Chung, SE Song, YT Cho (2017) Effective software solutions for 4D printing: A review and proposal. International Journal of Precision …, Springer, cited by 4 (2.00 per year)
T Li, G Li, Y Liang, T Cheng, J Dai, ... (2017) Fast-moving soft electronic fish. Science …, advances.sciencemag.org, cited by 115 (57.50 per year)
F Libonati, C Colombo, L Vergani (2014) Design and characterization of a biomimetic composite inspired to human bone. Fatigue & Fracture of …, Wiley Online Library, cited by 19 (3.80 per year)
F Xu, CM Wu, V Rengarajan, TD Finley, ... (2011) Three-dimensional magnetic assembly of microscale hydrogels. Advanced …, Wiley Online Library, cited by 180 (22.50 per year)
JY Hong, S Yun, JJ Wie, X Zhang, MS Dresselhaus, ... (2016) Cartilage-inspired superelastic ultradurable graphene aerogels prepared by the selective gluing of intersheet joints. Nanoscale, pubs.rsc.org, cited by 12 (4.00 per year)
S Trabia, Z Olsen, KJ Kim (2017) Searching for a new ionomer for 3D printable ionic polymer–metal composites: Aquivion as a candidate. Smart Materials and Structures, iopscience.iop.org, cited by 7 (3.50 per year)
CH Yang, MX Wang, H Haider, JH Yang, ... (2013) Strengthening alginate/polyacrylamide hydrogels using various multivalent cations. … applied materials & …, ACS Publications, cited by 247 (41.17 per year)
GX Gu, L Dimas, Z Qin, ... (2016) Optimization of composite fracture properties: method, validation, and applications. Journal of …, … .asmedigitalcollection.asme.org, cited by 23 (7.67 per year)
B Sharma, CG Williams, TK Kim, D Sun, A Malik, ... (2007) Designing zonal organization into tissue-engineered cartilage. Tissue …, liebertpub.com, cited by 151 (12.58 per year)
K Yang, C Zhou, H Fan, Y Fan, Q Jiang, P Song, ... (2017) Bio-functional design, application and trends in metallic biomaterials. International journal of …, mdpi.com, cited by 7 (3.50 per year)
S Tottori, L Zhang, F Qiu, KK Krawczyk, ... (2012) Magnetic helical micromachines: fabrication, controlled swimming, and cargo transport. Advanced …, Wiley Online Library, cited by 598 (85.43 per year)
R Mutlu, C Tawk, G Alici, ... (2017) A 3D printed monolithic soft gripper with adjustable stiffness. IECON 2017-43rd Annual …, ieeexplore.ieee.org, cited by 4 (2.00 per year)
Z Luo, DE Weiss, Q Liu, B Tian (2018) Biomimetic approaches toward smart bio-hybrid systems. Nano research, Springer, cited by 3 (3.00 per year)
CK Hsu, J Evans, S Vytla, PG Huang (2010) Development of flapping wing micro air vehicles-design, CFD, experiment and actual flight. 48th AIAA aerospace sciences …, arc.aiaa.org, cited by 41 (4.56 per year)
S Sprio, M Sandri, M Iafisco, S Panseri, ... (2016) Bio-inspired assembling/mineralization process as a flexible approach to develop new smart scaffolds for the regeneration of complex anatomical regions. Journal of the European …, Elsevier, cited by 6 (2.00 per year)
Y Yang, Y Chen, Y Li, MZ Chen (2016) 3D printing of variable stiffness hyper-redundant robotic arm. 2016 IEEE International …, ieeexplore.ieee.org, cited by 12 (4.00 per year)
SJ Keating, MI Gariboldi, WG Patrick, S Sharma, ... (2016) 3D printed multimaterial microfluidic valve. PloS one, journals.plos.org, cited by 25 (8.33 per year)

Y Ma, X Feng, JA Rogers, Y Huang, Y Zhang (2017) Design and application of 'J-shaped'stress–strain behavior in stretchable electronics: a review. Lab on a Chip, pubs.rsc.org, cited by 34 (17.00 per year)
S Lin, Y Zhong, X Zhao, T Sawada, X Li, ... (2018) Synthetic Multifunctional Graphene Composites with Reshaping and Self-Healing Features via a Facile Biomineralization-Inspired Process. Advanced …, Wiley Online Library, cited by 12 (12.00 per year)
Z Wang, J Zhang, J Xie, C Li, Y Li, ... (2010) Bioinspired water-vapor-responsive organic/inorganic hybrid one-dimensional photonic crystals with tunable full-color stop band. Advanced Functional …, Wiley Online Library, cited by 139 (15.44 per year)
MG Yeo, GH Kim (2011) Preparation and characterization of 3D composite scaffolds based on rapid-prototyped PCL/ β -TCP struts and electrospun PCL coated with collagen and HA for bone …. Chemistry of Materials, ACS Publications, cited by 84 (10.50 per year)
P Calvert (2001) Inkjet printing for materials and devices. Chemistry of materials, ACS Publications, cited by 1795 (99.72 per year)
L Wu, Z Dong, N Li, F Li, L Jiang, Y Song (2015) Manipulating oil droplets by superamphiphobic nozzle. Small, Wiley Online Library, cited by 21 (5.25 per year)
X Zhao, Q Lang, L Yildirimer, ZY Lin, ... (2016) Photocrosslinkable gelatin hydrogel for epidermal tissue engineering. Advanced …, Wiley Online Library, cited by 184 (61.33 per year)
K Zhang, P Chermprayong, TM Alhinai, ... (2017) Spidermav: Perching and stabilizing micro aerial vehicles with bio-inspired tensile anchoring systems. 2017 IEEE/RSJ …, ieeexplore.ieee.org, cited by 5 (2.50 per year)
W Wang, L Yao, T Zhang, CY Cheng, ... (2017) Transformative appetite: shape-changing food transforms from 2D to 3D by water interaction through cooking. Proceedings of the …, dl.acm.org, cited by 19 (9.50 per year)
V Slesarenko, S Engelkemier, P Galich, D Vladimirsky, ... (2018) Strategies to control performance of 3d-printed, cable-driven soft polymer actuators: from simple architectures to gripper prototype. Polymers, mdpi.com, cited by 3 (3.00 per year)
T Li, Y Chen, L Wang (2018) Enhanced fracture toughness in architected interpenetrating phase composites by 3D printing. Composites Science and Technology, Elsevier, cited by 9 (9.00 per year)
GM Policastro, ML Becker (2016) Osteogenic growth peptide and its use as a bio-conjugate in regenerative medicine applications. Wiley Interdisciplinary Reviews …, Wiley Online Library, cited by 19 (6.33 per year)
S Wei, G Qu, G Luo, Y Huang, H Zhang, ... (2018) Scalable and Automated Fabrication of Conductive Tough-Hydrogel Microfibers with Ultrastretchability, 3D Printability, and Stress Sensitivity. … applied materials & …, ACS Publications, cited by 7 (7.00 per year)
M Saari, B Xia, B Cox, PS Krueger, ... (2016) Fabrication and analysis of a composite 3D printed capacitive force sensor. 3D Printing and …, liebertpub.com, cited by 11 (3.67 per year)
R Passieux, L Guthrie, SH Rad, M Lévesque, ... (2015) Instability-assisted direct writing of microstructured fibers featuring sacrificial bonds. Advanced …, Wiley Online Library, cited by 29 (7.25 per year)
H Peng, J Mankoff, SE Hudson, J McCann (2015) A layered fabric 3D printer for soft interactive objects. Proceedings of the 33rd …, dl.acm.org, cited by 56 (14.00 per year)
R Guo, Y Yu, Z Xie, X Liu, X Zhou, Y Gao, ... (2013) Matrix-assisted catalytic printing for the fabrication of multiscale, flexible, foldable, and stretchable metal conductors. Advanced …, Wiley Online Library, cited by 119 (19.83 per year)

T Uyar, E Kny (2017) Electrospun materials for tissue engineering and biomedical applications: research, design and commercialization., books.google.com, cited by 33 (16.50 per year)
KJ Cui, CZ Zhu, H Zhang, Q Xuan, WZ Zou, ... (2017) Blue laser diode-initiated photosensitive resins for 3D printing. Journal of Materials …, pubs.rsc.org, cited by 2 (1.00 per year)
KC Galloway, KP Becker, B Phillips, J Kirby, S Licht, ... (2016) Soft robotic grippers for biological sampling on deep reefs. Soft robotics, liebertpub.com, cited by 189 (63.00 per year)
DJ Cohen, D Mitra, K Peterson, MM Maharbiz (2012) A highly elastic, capacitive strain gauge based on percolating nanotube networks. Nano letters, ACS Publications, cited by 274 (39.14 per year)
Y Wang, YM Kim, R Langer (2003) In vivo degradation characteristics of poly(glycerol sebacate). … Part A: An Official Journal of The …, Wiley Online Library, cited by 324 (20.25 per year)
TJ Klein, SC Rizzi, JC Reichert, ... (2009) Strategies for zonal cartilage repair using hydrogels. Macromolecular …, Wiley Online Library, cited by 134 (13.40 per year)
J Kim, A Alspach, K Yamane (2015) 3D printed soft skin for safe human-robot interaction. 2015 IEEE/RSJ International …, ieeexplore.ieee.org, cited by 36 (9.00 per year)
SM Giannitelli, P Mozetic, M Trombetta, A Rainer (2015) Combined additive manufacturing approaches in tissue engineering. Acta biomaterialia, Elsevier, cited by 76 (19.00 per year)
BN Johnson, KZ Lancaster, G Zhen, ... (2015) 3D printed anatomical nerve regeneration pathways. Advanced functional …, Wiley Online Library, cited by 107 (26.75 per year)
RV Martinez, JL Branch, CR Fish, L Jin, ... (2013) Robotic tentacles with three-dimensional mobility based on flexible elastomers. Advanced …, Wiley Online Library, cited by 395 (65.83 per year)
J Duro-Royo, K Zolotovsky, L Mogas-Soldevila, ... (2015) MetaMesh: A hierarchical computational model for design and fabrication of biomimetic armored surfaces. Computer-Aided …, Elsevier, cited by 31 (7.75 per year)
K Haraguchi, HJ Li (2005) Control of the Coil-to-Globule Transition and Ultrahigh Mechanical Properties of PNIPA in Nanocomposite Hydrogels. Angewandte Chemie International Edition, Wiley Online Library, cited by 243 (17.36 per year)
JZ Wang, NY Xiong, LZ Zhao, JT Hu, DC Kong, ... (2018) Review fantastic medical implications of 3D-printing in liver surgeries, liver regeneration, liver transplantation and drug hepatotoxicity testing: A review. International Journal of …, Elsevier, cited by 2 (2.00 per year)
J Mačiulaitis, M Deveikytė, S Rekštytė, ... (2015) Preclinical study of SZ2080 material 3D microstructured scaffolds for cartilage tissue engineering made by femtosecond direct laser writing lithography. …, iopscience.iop.org, cited by 72 (18.00 per year)
MD Bartlett, AB Croll, DR King, BM Paret, ... (2012) Looking beyond fibrillar features to scale gecko-like adhesion. Advanced …, Wiley Online Library, cited by 166 (23.71 per year)
F Simone, A York, S Seelecke (2015) Design and fabrication of a three-finger prosthetic hand using SMA muscle wires. … , and Bioreplication 2015, spiedigitallibrary.org, cited by 17 (4.25 per year)
MD Guillemette, H Park, JC Hsiao, ... (2010) Combined technologies for microfabricating elastomeric cardiac tissue engineering scaffolds. Macromolecular …, Wiley Online Library, cited by 61 (6.78 per year)

J Mueller, D Courty, M Spielhofer, ... (2017) Mechanical properties of interfaces in inkjet 3D printed single-and multi-material parts. 3D Printing and …, liebertpub.com, cited by 9 (4.50 per year)
J Guerreiro, JC Jackson, JFC Windmill (2016) Bio-inspired frequency agile acoustic system. 2016 IEEE SENSORS, ieeexplore.ieee.org, cited by 2 (0.67 per year)
Y Xu, X Wu, X Guo, B Kong, M Zhang, X Qian, S Mi, ... (2017) The boom in 3D-printed sensor technology. Sensors, mdpi.com, cited by 57 (28.50 per year)
H Cho, U Jammalamadaka, K Tappa (2018) Nanogels for pharmaceutical and biomedical applications and their fabrication using 3D printing technologies. Materials, mdpi.com, cited by 5 (5.00 per year)
Y Chen, T Li, F Scarpa, L Wang (2017) Lattice metamaterials with mechanically tunable Poisson's ratio for vibration control. Physical Review Applied, APS, cited by 71 (35.50 per year)
Y Yang, X Li, X Zheng, Z Chen, Q Zhou, ... (2018) Superhydrophobicity: 3D-Printed Biomimetic Super-Hydrophobic Structure for Microdroplet Manipulation and Oil/Water Separation (Adv. Mater. 9/2018). Advanced …, Wiley Online Library, cited by 2 (2.00 per year)
A Ji, Z Zhao, P Manoonpong, W Wang, G Chen, ... (2018) A bio-inspired climbing robot with flexible pads and claws. Journal of Bionic …, Springer, cited by 3 (3.00 per year)
A Gregor, E Filová, M Novák, ... (2017) Designing of PLA scaffolds for bone tissue replacement fabricated by ordinary commercial 3D printer. Journal of …, jbioleng.biomedcentral.com, cited by 35 (17.50 per year)
M Lapeyre, P Rouanet, ... (2013) The poppy humanoid robot: Leg design for biped locomotion. 2013 IEEE/RSJ …, ieeexplore.ieee.org, cited by 34 (5.67 per year)
R Wang, J Shang, X Li, Z Wang, ... (2018) Novel topological design of 3D Kagome structure for additive manufacturing. Rapid Prototyping …, emeraldinsight.com, cited by 2 (2.00 per year)
TM Valentin, SE Leggett, PY Chen, JK Sodhi, ... (2017) Stereolithographic printing of ionically-crosslinked alginate hydrogels for degradable biomaterials and microfluidics. Lab on a Chip, pubs.rsc.org, cited by 15 (7.50 per year)
C Hintz, P Khanbolouki, AM Perez, M Tehrani, ... (2018) Experimental study of the effects of bio-inspired blades and 3D printing on the performance of a small propeller. 2018 Applied …, arc.aiaa.org, cited by 1 (1.00 per year)
C Credi, A Fiorese, M Tironi, R Bernasconi, ... (2016) 3D printing of cantilever-type microstructures by stereolithography of ferromagnetic photopolymers. … applied materials & …, ACS Publications, cited by 27 (9.00 per year)
L Moroni, T Boland, JA Burdick, C De Maria, ... (2018) Biofabrication: a guide to technology and terminology. Trends in …, Elsevier, cited by 58 (58.00 per year)
SJ Leigh, J Bowen, CP Purssell, ... (2012) Rapid manufacture of monolithic micro-actuated forceps inspired by echinoderm pedicellariae. Bioinspiration & …, iopscience.iop.org, cited by 5 (0.71 per year)
MAT Arango, NJ Morris, KA Sierros (2018) Direct Writing and Controlling of Hierarchical Functional Metal-Oxides: Bio-inspired Multiphase Processing, 3D Printing and Hierarchical Cellular Structuring. JOM, Springer, cited by 1 (1.00 per year)
A Bruyas, F Geiskopf, P Renaud (2014) Towards statically balanced compliant joints using multimaterial 3D printing. ASME 2014 …, … .asmedigitalcollection.asme.org, cited by 10 (2.00 per year)

J Wang, Y Wen, H Ge, Z Sun, Y Zheng, ... (2006) Simple fabrication of full color colloidal crystal films with tough mechanical strength. Macromolecular …, Wiley Online Library, cited by 200 (15.38 per year)
SMM Valashani, F Barthelat (2015) A laser-engraved glass duplicating the structure, mechanics and performance of natural nacre. Bioinspiration & biomimetics, iopscience.iop.org, cited by 57 (14.25 per year)
ZZ Gu, H Uetsuka, K Takahashi, ... (2003) Structural color and the lotus effect. Angewandte Chemie …, Wiley Online Library, cited by 456 (28.50 per year)
F Libonati, MJ Buehler (2017) Advanced structural materials by bioinspiration. Advanced Engineering Materials, Wiley Online Library, cited by 28 (14.00 per year)
V Schmidt, M Belegratis (2016) Laser technology in biomimetics., Springer, cited by 13 (4.33 per year)
JB Berger, HNG Wadley, RM McMeeking (2017) Mechanical metamaterials at the theoretical limit of isotropic elastic stiffness. Nature, nature.com, cited by 94 (47.00 per year)
AR Abdel Fattah, S Ghosh, IK Puri (2016) Printing three-dimensional heterogeneities in the elastic modulus of an elastomeric matrix. ACS applied materials & …, ACS Publications, cited by 10 (3.33 per year)
L Ambrosio (2017) Biomedical composites., books.google.com, cited by 17 (8.50 per year)
MS Alsoufi, AE Elsayed (2017) How surface roughness performance of printed parts manufactured by desktop FDM 3D printer with PLA+ is influenced by measuring direction. Am. J. Mech. Eng, researchgate.net, cited by 13 (6.50 per year)
YF Zhou, CZ Yao, QL Yang, L Guo, ... (2016) Mechanical Properties of Diamond-Structured Polymer Microlattices Coated with the Silicon Nitride Film. Advanced Engineering …, Wiley Online Library, cited by 8 (2.67 per year)
H Gu, D Ren (2014) Materials and surface engineering to control bacterial adhesion and biofilm formation: A review of recent advances. Frontiers of Chemical Science and Engineering, Springer, cited by 42 (8.40 per year)
Z Lei, Q Wang, S Sun, W Zhu, P Wu (2017) A bioinspired mineral hydrogel as a self-healable, mechanically adaptable ionic skin for highly sensitive pressure sensing. Advanced Materials, Wiley Online Library, cited by 132 (66.00 per year)
A Ovsianikov, B Chichkov, P Mente, ... (2007) Two photon polymerization of polymer–ceramic hybrid materials for transdermal drug delivery. … journal of applied …, Wiley Online Library, cited by 140 (11.67 per year)
Y Kim, H Yuk, R Zhao, SA Chester, X Zhao (2018) Printing ferromagnetic domains for untethered fast-transforming soft materials. Nature, nature.com, cited by 85 (85.00 per year)
H Zhang, X Mao, Z Du, W Jiang, X Han, ... (2016) Three dimensional printed macroporous polylactic acid/hydroxyapatite composite scaffolds for promoting bone formation in a critical-size rat calvarial defect model. … and Technology of …, Taylor & Francis, cited by 45 (15.00 per year)
V Palermo, IA Kinloch, S Ligi, NM Pugno (2016) Nanoscale mechanics of graphene and graphene oxide in composites: a scientific and technological perspective. Advanced Materials, Wiley Online Library, cited by 54 (18.00 per year)
P He, J Zhao, J Zhang, B Li, Z Gou, ... (2018) Bioprinting of skin constructs for wound healing. Burns & …, burnstrauma.biomedcentral.com, cited by 27 (27.00 per year)
X Yao, Q Chen, L Xu, Q Li, Y Song, ... (2010) Bioinspired ribbed nanoneedles with robust superhydrophobicity. Advanced functional …, Wiley Online Library, cited by 158 (17.56 per year)

S Jo, SM Kang, SA Park, WD Kim, ... (2013) Enhanced Adhesion of Preosteoblasts inside 3 D PCL Scaffolds by Polydopamine Coating and Mineralization. Macromolecular …, Wiley Online Library, cited by 37 (6.17 per year)
R Mirzaeifar, LS Dimas, Z Qin, ... (2015) Defect-tolerant bioinspired hierarchical composites: simulation and experiment. … Biomaterials Science & …, ACS Publications, cited by 33 (8.25 per year)
T Gong, J Xie, J Liao, T Zhang, S Lin, Y Lin (2015) Nanomaterials and bone regeneration. Bone research, nature.com, cited by 201 (50.25 per year)
M Kucewicz, P Baranowski, J Małachowski, ... (2018) Modelling, and characterization of 3D printed cellular structures. Materials & Design, Elsevier, cited by 25 (25.00 per year)
N Kaushik, N Kaushik, S Pardeshi, J Sharma, S Lee, ... (2015) Biomedical and clinical importance of mussel-inspired polymers and materials. Marine drugs, mdpi.com, cited by 34 (8.50 per year)
GX Gu, CT Chen, MJ Buehler (2018) De novo composite design based on machine learning algorithm. Extreme Mechanics Letters, Elsevier, cited by 30 (30.00 per year)
JO Dabiri, JR Greer, JR Koseff, P Moin, ... (2015) A new approach to wind energy: opportunities and challenges. AIP Conference …, aip.scitation.org, cited by 32 (8.00 per year)
M Kuang, L Wang, Y Song (2014) Controllable printing droplets for high-resolution patterns. Advanced materials, Wiley Online Library, cited by 175 (35.00 per year)
KJ Ang, KS Riley, J Faber, ... (2018) Switchable Bistability in 3D Printed Shells With Bio-Inspired Architectures and Spatially Distributed Pre-Stress. ASME 2018 …, … .asmedigitalcollection.asme.org, cited by 1 (1.00 per year)
MD Sarker, S Naghieh, NK Sharma, X Chen (2018) 3D biofabrication of vascular networks for tissue regeneration: A report on recent advances. Journal of pharmaceutical …, Elsevier, cited by 10 (10.00 per year)
N Gaissert, R Mugrauer, G Mugrauer, A Jebens, ... (2013) Inventing a micro aerial vehicle inspired by the mechanics of dragonfly flight. Conference Towards …, Springer, cited by 20 (3.33 per year)
L Wu, M Larkin, A Potnuru, Y Tadesse (2016) HBS-1: a modular child-size 3D printed humanoid. Robotics, mdpi.com, cited by 14 (4.67 per year)
GX Gu, S Wettermark, MJ Buehler (2017) Algorithm-driven design of fracture resistant composite materials realized through additive manufacturing. Additive Manufacturing, Elsevier, cited by 18 (9.00 per year)
G Vozzi, A Previti, D De Rossi, A Ahluwalia (2002) Microsyringe-based deposition of two-dimensional and three-dimensional polymer scaffolds with a well-defined geometry for application to tissue engineering. Tissue engineering, liebertpub.com, cited by 212 (12.47 per year)
S Kim, Y Su, A Mihi, S Lee, Z Liu, TK Bhandakkar, J Wu, ... (2012) Imbricate scales as a design construct for microsystem technologies. small, Wiley Online Library, cited by 29 (4.14 per year)
Y He, L Shi, S Guo, S Pan, Z Wang (2016) Preliminary mechanical analysis of an improved amphibious spherical father robot. Microsystem Technologies, Springer, cited by 31 (10.33 per year)
MA Meyers, YS Lin, EA Olevsky, ... (2012) Battle in the Amazon: arapaima versus piranha. Advanced Engineering …, Wiley Online Library, cited by 67 (9.57 per year)

Q Zhao, W Zou, Y Luo, T Xie (2016) Shape memory polymer network with thermally distinct elasticity and plasticity. Science advances, advances.sciencemag.org, cited by 165 (55.00 per year)
P Taynton, H Ni, C Zhu, K Yu, S Loob, Y Jin, ... (2016) Repairable woven carbon fiber composites with full recyclability enabled by malleable polyimine networks. Advanced …, Wiley Online Library, cited by 111 (37.00 per year)
R Li, W Wu, H Qiao (2015) The compliance of robotic hands–from functionality to mechanism. Assembly Automation, emeraldinsight.com, cited by 12 (3.00 per year)
CJ Liao, CF Chen, JH Chen, SF Chiang, ... (2002) Fabrication of porous biodegradable polymer scaffolds using a solvent merging/particulate leaching method. … Research: An Official …, Wiley Online Library, cited by 269 (15.82 per year)
YL Cheng, YW Chen, K Wang, MY Shie (2016) Enhanced adhesion and differentiation of human mesenchymal stem cell inside apatite-mineralized/poly (dopamine)-coated poly (ε -caprolactone) scaffolds by …. Journal of Materials …, pubs.rsc.org, cited by 36 (12.00 per year)
A Rao, MR Elara, K Elangovan (2016) Constrained VPH+: a local path planning algorithm for a bio-inspired crawling robot with customized ultrasonic scanning sensor. Robotics and biomimetics, biomedcentral.com, cited by 2 (0.67 per year)
SE Naleway, MM Porter, J McKittrick, ... (2015) Structural design elements in biological materials: application to bioinspiration. Advanced …, Wiley Online Library, cited by 161 (40.25 per year)
E Gultepe, JS Randhawa, S Kadam, ... (2013) Biopsy with thermally-responsive untethered microtools. Advanced …, Wiley Online Library, cited by 127 (21.17 per year)
X Xu, H Li, Q Zhang, H Hu, Z Zhao, J Li, J Li, Y Qiao, ... (2015) Self-sensing, ultralight, and conductive 3D graphene/iron oxide aerogel elastomer deformable in a magnetic field. ACS …, ACS Publications, cited by 159 (39.75 per year)
WH Yeo, YS Kim, J Lee, A Ameen, L Shi, ... (2013) Multifunctional epidermal electronics printed directly onto the skin. Advanced …, Wiley Online Library, cited by 429 (71.50 per year)
B Li, H Tan, S Anastasova, M Power, ... (2019) A bio-inspired 3D micro-structure for graphene-based bacteria sensing. Biosensors and …, Elsevier, cited by 1 (1.00 per year)
MS Alsoufi, AE Elsayed (2017) Warping deformation of desktop 3D printed parts manufactured by open source fused deposition modeling (FDM) system. International Journal of Mechanical and …, researchgate.net, cited by 10 (5.00 per year)
Y Wu, J Fuh, YS Wong, J Sun (2017) A hybrid electrospinning and electrospraying 3D printing for tissue engineered scaffolds. Rapid Prototyping Journal, emeraldinsight.com, cited by 3 (1.50 per year)
AP Haring, AU Khan, G Liu, ... (2017) 3D Printed Functionally Graded Plasmonic Constructs. Advanced Optical …, Wiley Online Library, cited by 13 (6.50 per year)
YL Sun, WF Dong, RZ Yang, X Meng, ... (2012) Dynamically tunable protein microlenses. Angewandte Chemie …, Wiley Online Library, cited by 87 (12.43 per year)
R Ma, Y Lai, L Li, H Tan, J Wang, Y Li, T Tang, L Qin (2015) Bacterial inhibition potential of 3D rapid-prototyped magnesium-based porous composite scaffolds–an in vitro efficacy study. Scientific reports, nature.com, cited by 25 (6.25 per year)
A Horn, S Hiltl, A Fery, A Böker (2010) Ordering and printing virus arrays: a straightforward way to functionalize surfaces. Small, Wiley Online Library, cited by 45 (5.00 per year)

S Kim, F Qiu, S Kim, A Ghanbari, C Moon, ... (2013) Fabrication and characterization of magnetic microrobots for three-dimensional cell culture and targeted transportation. Advanced …, Wiley Online Library, cited by 189 (31.50 per year)
SM Giannitelli, D Accoto, M Trombetta, A Rainer (2014) Current trends in the design of scaffolds for computer-aided tissue engineering. Acta biomaterialia, Elsevier, cited by 226 (45.20 per year)
A Fassler, C Majidi (2015) Liquid-phase metal inclusions for a conductive polymer composite. Advanced Materials, Wiley Online Library, cited by 116 (29.00 per year)
SH Pyo, P Wang, HH Hwang, W Zhu, ... (2016) Continuous optical 3D printing of green aliphatic polyurethanes. … applied materials & …, ACS Publications, cited by 13 (4.33 per year)
S Jiang, F Liu, A Lerch, L Ionov, ... (2015) Unusual and superfast temperature-triggered actuators. Advanced materials, Wiley Online Library, cited by 112 (28.00 per year)
SM Mihaila, AK Gaharwar, RL Reis, ... (2013) Photocrosslinkable Kappa-Carrageenan Hydrogels for Tissue Engineering Applications. Advanced …, Wiley Online Library, cited by 82 (13.67 per year)
L Guiducci, K Razghandi, L Bertinetti, S Turcaud, ... (2016) Honeycomb actuators inspired by the unfolding of ice plant seed capsules. PloS one, journals.plos.org, cited by 4 (1.33 per year)
SCN Chang, JA Rowley, G Tobias, ... (2001) Injection molding of chondrocyte/alginate constructs in the shape of facial implants. … Research: An Official …, Wiley Online Library, cited by 322 (17.89 per year)
YY Diao, XY Liu, GW Toh, L Shi, ... (2013) Multiple structural coloring of silk-fibroin photonic crystals and humidity-responsive color sensing. Advanced Functional …, Wiley Online Library, cited by 116 (19.33 per year)
RF Shepherd, AA Stokes, RMD Nunes, ... (2013) Soft machines that are resistant to puncture and that self seal. Advanced …, Wiley Online Library, cited by 109 (18.17 per year)
JA Neal, D Mozhdehi, Z Guan (2015) Enhancing mechanical performance of a covalent self-healing material by sacrificial noncovalent bonds. Journal of the American Chemical …, ACS Publications, cited by 138 (34.50 per year)
M Strantza, D De Baere, M Rombouts, ... (2015) Feasibility study on integrated structural health monitoring system produced by metal three-dimensional printing. Structural Health …, journals.sagepub.com, cited by 10 (2.50 per year)
H Marvi, S Song, M Sitti (2015) Experimental investigation of optimal adhesion of mushroomlike elastomer microfibrillar adhesives. Langmuir, ACS Publications, cited by 16 (4.00 per year)
L Xu, TC Shyu, NA Kotov (2017) Origami and kirigami nanocomposites. Acs Nano, ACS Publications, cited by 57 (28.50 per year)
A Cheng, A Humayun, BD Boyan, ... (2016) Enhanced osteoblast response to porosity and resolution of additively manufactured Ti-6Al-4V constructs with trabeculae-inspired porosity. 3D printing and additive …, liebertpub.com, cited by 7 (2.33 per year)
L Wen, JC Weaver, GV Lauder (2014) Biomimetic shark skin: design, fabrication and hydrodynamic function. Journal of Experimental Biology, jeb.biologists.org, cited by 185 (37.00 per year)
A Papadopoulou, J Laucks, S Tibbits (2017) Auxetic materials in design and architecture. Nat. Rev. Mater, nature.com, cited by 10 (5.00 per year)

M Mirkhalaf, F Barthelat (2016) Nacre-like materials using a simple doctor blading technique: Fabrication, testing and modeling. Journal of the mechanical behavior of biomedical …, Elsevier, cited by 20 (6.67 per year)
S Yang, KF Leong, Z Du, CK Chua (2001) The design of scaffolds for use in tissue engineering. Part I. Traditional factors. Tissue engineering, liebertpub.com, cited by 2272 (126.22 per year)
S Rudykh, MC Boyce (2014) Transforming small localized loading into large rotational motion in soft anisotropically structured materials. Advanced Engineering Materials, Wiley Online Library, cited by 17 (3.40 per year)
Y Zhang, Z Dong, P Phillips, S Wang, G Ji, ... (2015) Detection of subjects and brain regions related to Alzheimer's disease using 3D MRI scans based on eigenbrain and machine learning. Frontiers in …, frontiersin.org, cited by 118 (29.50 per year)
JI Sasaki, TA Asoh, T Matsumoto, H Egusa, ... (2010) Fabrication of three-dimensional cell constructs using temperature-responsive hydrogel. … Engineering Part A, liebertpub.com, cited by 27 (3.00 per year)
C Santulli, C Langella (2016) Study and development of concepts of auxetic structures in bio-inspired design. International Journal of Sustainable …, researchgate.net, cited by 4 (1.33 per year)
SKV Aditya, J Ignasov, K Filonenko, ... (2017) Bio-Inspired Design and Kinematic Analysis of Dung Beetle-Like Legs. … and Bio-Inspired …, portal.findresearcher.sdu.dk, cited by 1 (0.50 per year)
Z Qin, L Dimas, D Adler, G Bratzel, ... (2014) Biological materials by design. Journal of Physics …, iopscience.iop.org, cited by 18 (3.60 per year)
PF Egan, SJ Ferguson, K Shea (2017) Design of hierarchical three-dimensional printed scaffolds considering mechanical and biological factors for bone tissue engineering. Journal of …, … .asmedigitalcollection.asme.org, cited by 19 (9.50 per year)
T Nathan-Walleser, IM Lazar, ... (2014) 3D Micro-Extrusion of Graphene-based Active Electrodes: Towards High-Rate AC Line Filtering Performance Electrochemical Capacitors. Advanced Functional …, Wiley Online Library, cited by 43 (8.60 per year)
L Zhang, H Liu, Y Zhao, X Sun, Y Wen, ... (2012) Inkjet printing high-resolution, large-area graphene patterns by coffee-ring lithography. Advanced …, Wiley Online Library, cited by 108 (15.43 per year)
M Alfano, C Morano, L Bruno, M Muzzupappa, ... (2018) Analysis of debonding in bio-inspired interfaces obtained by additive manufacturing. Procedia Structural …, Elsevier, cited by 2 (2.00 per year)
A Carlson, AM Bowen, Y Huang, RG Nuzzo, ... (2012) Transfer printing techniques for materials assembly and micro/nanodevice fabrication. Advanced …, Wiley Online Library, cited by 460 (65.71 per year)
GJM Krijnen, RGP Sanders (2017) Recent developments in bio-inspired sensors fabricated by additive manufacturing technologies. Advances in science and technology, Trans Tech Publ, cited by 1 (0.50 per year)
N Picollet-D'hahan, ME Dolega, L Liguori, ... (2016) A 3D toolbox to enhance physiological relevance of human tissue models. Trends in …, Elsevier, cited by 34 (11.33 per year)
P Allgeuer, H Farazi, M Schreiber, ... (2015) Child-sized 3D printed igus humanoid open platform. 2015 IEEE-RAS 15th …, ieeexplore.ieee.org, cited by 29 (7.25 per year)
D Chandra, S Yang (2010) Stability of high-aspect-ratio micropillar arrays against adhesive and capillary forces. Accounts of chemical research, ACS Publications, cited by 178 (19.78 per year)

M Biondi, F Ungaro, F Quaglia, PA Netti (2008) Controlled drug delivery in tissue engineering. Advanced drug delivery reviews, Elsevier, cited by 348 (31.64 per year)
S Li, KW Wang (2015) Fluidic origami: a plant-inspired adaptive structure with shape morphing and stiffness tuning. Smart Materials and Structures, iopscience.iop.org, cited by 43 (10.75 per year)
Q Chen, L Zhu, C Zhao, Q Wang, ... (2013) A robust, one-pot synthesis of highly mechanical and recoverable double network hydrogels using thermoreversible sol-gel polysaccharide. Advanced materials, Wiley Online Library, cited by 297 (49.50 per year)
JH Jeong, V Chan, C Cha, P Zorlutuna, ... (2012) "Living" microvascular stamp for patterning of functional neovessels; orchestrated control of matrix property and geometry. Advanced …, Wiley Online Library, cited by 56 (8.00 per year)
P Li (2003) Biomimetic nano-apatite coating capable of promoting bone ingrowth. Journal of Biomedical Materials Research Part A: An …, Wiley Online Library, cited by 170 (10.63 per year)
J Hodde (2002) Naturally occurring scaffolds for soft tissue repair and regeneration. Tissue engineering, liebertpub.com, cited by 331 (19.47 per year)
R Matthew, H Lund, K Yoshida (2010) A bio-inspired compliant claw for arboreal locomotion in microgravity environments. 2010 IEEE/SICE International …, ieeexplore.ieee.org, cited by 4 (0.44 per year)
S Ling, DL Kaplan, MJ Buehler (2018) Nanofibrils in nature and materials engineering. Nature Reviews Materials, nature.com, cited by 51 (51.00 per year)
D Krupke, N Hendrich, J Zhang, ... (2016) Gait optimization based on physics simulation of 3d robot models with a modular robotic simulation system. … : Proceedings of the 18th …, World Scientific, cited by 4 (1.33 per year)
A Skardal, D Mack, E Kapetanovic, ... (2012) Bioprinted amniotic fluid-derived stem cells accelerate healing of large skin wounds. Stem cells …, Wiley Online Library, cited by 280 (40.00 per year)
C Tawk, M in het Panhuis, GM Spinks, G Alici (2018) Bioinspired 3D Printable Soft Vacuum Actuators for Locomotion Robots, Grippers and Artificial Muscles. Soft robotics, liebertpub.com, cited by 3 (3.00 per year)
NF Lepora, A Duff, A Mura, TJ Prescott, ... (2014) Biomimetic and biohybrid systems., Springer International Publishing, cited by 6 (1.20 per year)
RTL Ferreira, IC Amatte, TA Dutra, D Bürger (2017) Experimental characterization and micrography of 3D printed PLA and PLA reinforced with short carbon fibers. Composites Part B …, Elsevier, cited by 75 (37.50 per year)
J Wei, L Wang, L Liao, J Wang, Y Han, ... (2017) Construction of bio-inspired composites for bone tissue repair. Advances in Bioinspired …, ACS Publications, cited by 1 (0.50 per year)
GH Loh, E Pei, D Harrison, MD Monzon (2018) An overview of functionally graded additive manufacturing. Additive Manufacturing, Elsevier, cited by 24 (24.00 per year)
JC Breger, CK Yoon, R Xiao, HR Kwag, ... (2015) Self-folding thermo-magnetically responsive soft microgrippers. … applied materials & …, ACS Publications, cited by 169 (42.25 per year)
Y Ding, F Qiu, X Casadevall i Solvas, F Chiu, B Nelson, ... (2016) Microfluidic-based droplet and cell manipulations using artificial bacterial flagella. Micromachines, mdpi.com, cited by 22 (7.33 per year)
P Taynton, K Yu, RK Shoemaker, Y Jin, ... (2014) Heat-or Water-Driven Malleability in a Highly Recyclable Covalent Network Polymer. Advanced …, Wiley Online Library, cited by 185 (37.00 per year)

ATT Nguyen, M Brandt, AC Orifici, S Feih (2016) Hierarchical surface features for improved bonding and fracture toughness of metal–metal and metal–composite bonded joints. International Journal of Adhesion …, Elsevier, cited by 19 (6.33 per year)
X Du, S Fu, Y Zhu (2018) 3D printing of ceramic-based scaffolds for bone tissue engineering: an overview. Journal of Materials Chemistry B, pubs.rsc.org, cited by 13 (13.00 per year)
O Guillame-Gentil, O Semenov, AS Roca, ... (2010) Engineering the extracellular environment: strategies for building 2D and 3D cellular structures. Advanced …, Wiley Online Library, cited by 136 (15.11 per year)
HL Gao, SM Chen, LB Mao, ZQ Song, HB Yao, ... (2017) Mass production of bulk artificial nacre with excellent mechanical properties. Nature …, nature.com, cited by 65 (32.50 per year)
S Sareh, R Siddall, T Alhinai, M Kovac (2017) Bio-inspired soft aerial robots: adaptive morphology for high-performance flight. Soft Robotics: Trends …, Springer, cited by 1 (0.50 per year)
H Ko, A Javey (2017) Smart actuators and adhesives for reconfigurable matter. Accounts of chemical research, ACS Publications, cited by 38 (19.00 per year)
P Xie (2014) Development of a bio-inspired UAV perching system., search.proquest.com, cited by 3 (0.60 per year)
N Li, P Yu, T Yang, L Zhao, Z Liu, ... (2017) Bio-inspired wearable soft upper-limb exoskeleton robot for stroke survivors. 2017 IEEE International …, ieeexplore.ieee.org, cited by 1 (0.50 per year)
PI Galich, V Slesarenko, S Rudykh (2017) Shear wave propagation in finitely deformed 3D fiber-reinforced composites. International Journal of Solids and …, Elsevier, cited by 14 (7.00 per year)
CF Revelo, HA Colorado (2018) 3D printing of kaolinite clay ceramics using the Direct Ink Writing (DIW) technique. Ceramics International, Elsevier, cited by 8 (8.00 per year)
ME Gomes, MT Rodrigues, ... (2017) Tissue engineering and regenerative medicine: new trends and directions—a year in review. … Engineering Part B …, liebertpub.com, cited by 37 (18.50 per year)
Y Tang, S Gong, Y Chen, LW Yap, W Cheng (2014) Manufacturable conducting rubber ambers and stretchable conductors from copper nanowire aerogel monoliths. ACS nano, ACS Publications, cited by 141 (28.20 per year)
A Tan, R Chawla, G Natasha, ... (2016) Nanotechnology and regenerative therapeutics in plastic surgery: the next frontier. Journal of Plastic …, Elsevier, cited by 9 (3.00 per year)
E Cabane, T Keplinger, T Künniger, V Merk, I Burgert (2016) Functional lignocellulosic materials prepared by ATRP from a wood scaffold. Scientific reports, nature.com, cited by 20 (6.67 per year)
E Munch, E Saiz, AP Tomsia, ... (2009) Architectural control of freeze-cast ceramics through additives and templating. Journal of the American …, Wiley Online Library, cited by 190 (19.00 per year)
J Clune, A Chen, H Lipson (2013) Upload any object and evolve it: Injecting complex geometric patterns into CPPNs for further evolution. 2013 IEEE Congress on …, ieeexplore.ieee.org, cited by 15 (2.50 per year)
F Barthelat (2015) Architectured materials in engineering and biology: fabrication, structure, mechanics and performance. International Materials Reviews, Taylor & Francis, cited by 45 (11.25 per year)

L Jiang, Y Zhao, J Zhai (2004) A lotus-leaf-like superhydrophobic surface: a porous microsphere/nanofiber composite film prepared by electrohydrodynamics. Angewandte Chemie International …, Wiley Online Library, cited by 1053 (70.20 per year)
O Bas, D D'Angella, JG Baldwin, ... (2017) An integrated design, material, and fabrication platform for engineering biomechanically and biologically functional soft tissues. … applied materials & …, ACS Publications, cited by 19 (9.50 per year)
H Ardiny, S Witwicki, F Mondada (2015) Are autonomous mobile robots able to take over construction? A review. International Journal of Robotics …, ijr.kntu.ac.ir, cited by 15 (3.75 per year)
G Di Canio, S Stoyanov, JC Larsen, J Hallam, ... (2016) A robot leg with compliant tarsus and its neural control for efficient and adaptive locomotion on complex terrains. Artificial Life and …, Springer, cited by 8 (2.67 per year)
Y Chen, F Wan, T Wu, C Song (2017) Soft-rigid interaction mechanism towards a lobster-inspired hybrid actuator. Journal of Micromechanics and …, iopscience.iop.org, cited by 2 (1.00 per year)
AM Mehta, D Rus, K Mohta, Y Mulgaonkar, M Piccoli, ... (2016) A scripted printable quadrotor: Rapid design and fabrication of a folded MAV. Robotics Research, Springer, cited by 21 (7.00 per year)
Z Chen, L Ren, J Li, L Yao, Y Chen, B Liu, L Jiang (2018) Rapid fabrication of microneedles using magnetorheological drawing lithography. Acta biomaterialia, Elsevier, cited by 10 (10.00 per year)
J Wang, Y Wen, X Feng, Y Song, ... (2006) Control over the wettability of colloidal crystal films by assembly temperature. Macromolecular rapid …, Wiley Online Library, cited by 79 (6.08 per year)
H Bai, F Walsh, B Gludovatz, B Delattre, ... (2016) Bioinspired hydroxyapatite/poly (methyl methacrylate) composite with a nacre-mimetic architecture by a bidirectional freezing method. Advanced …, Wiley Online Library, cited by 144 (48.00 per year)
Y Jin, Y Shen, J Yin, J Qian, ... (2018) Nanoclay-based self-supporting responsive nanocomposite hydrogels for printing applications. ACS applied materials & …, ACS Publications, cited by 11 (11.00 per year)
TC Hung, RH Piedrahita (2014) Experimental validation of a novel bio-inspired particle separator. Aquacultural engineering, Elsevier, cited by 1 (0.20 per year)
R Bao, J Li, L Li, TJ Cutright, L Chen, ... (2017) Bio-Inspired Bridge Scour Countermeasures: Streamlining and Biocementation. … on Materials Science …, dpi-proceedings.com, cited by 1 (0.50 per year)
SS Xie, O Vasylkiv, V Silberschmidt, ... (2013) Bio-inspired structured boron carbide-boron nitride composite by reactive spark plasma sintering: This paper describes the prototyping of an approach to improve …. Virtual and Physical …, Taylor & Francis, cited by 2 (0.33 per year)
Y Engel, R Elnathan, A Pevzner, ... (2010) Supersensitive detection of explosives by silicon nanowire arrays. Angewandte Chemie …, Wiley Online Library, cited by 241 (26.78 per year)
JM McCracken, A Badea, ME Kandel, ... (2016) Programming mechanical and physicochemical properties of 3d hydrogel cellular microcultures via direct ink writing. Advanced …, Wiley Online Library, cited by 18 (6.00 per year)
AA Bauhofer, S Krödel, J Rys, OR Bilal, ... (2017) Harnessing photochemical shrinkage in direct laser writing for shape morphing of polymer sheets. Advanced …, Wiley Online Library, cited by 19 (9.50 per year)

T Borangiu (2014) Advances in robot design and intelligent control. Switzerland: Springer International Publishing, Springer, cited by 13 (2.60 per year)
A Kapilavai, J Ignasov, K Filonenko, ... (2017) Bio-inspired design and kinematic analysis of dung beetle-like legs. … and Bio-Inspired …, researchgate.net, cited by 1 (0.50 per year)
F Xu, Y Zhu (2012) Highly conductive and stretchable silver nanowire conductors. Advanced materials, Wiley Online Library, cited by 782 (111.71 per year)
Y Liu, E Gill, YY Shery Huang (2017) Microfluidic on-chip biomimicry for 3D cell culture: a fit-for-purpose investigation from the end user standpoint. Future science OA, Future Science, cited by 15 (7.50 per year)
RG Jadhav, AK Das (2017) Four dimensional printing in healthcare. 3D Printing in Medicine, Elsevier, cited by 1 (0.50 per year)
J Duan, X Liang, J Guo, K Zhu, L Zhang (2016) Ultra-Stretchable and Force-Sensitive Hydrogels Reinforced with Chitosan Microspheres Embedded in Polymer Networks. Advanced Materials, Wiley Online Library, cited by 72 (24.00 per year)
H Gu, Y Zhao, Y Cheng, Z Xie, F Rong, J Li, B Wang, ... (2013) Tailoring colloidal photonic crystals with wide viewing angles. Small, Wiley Online Library, cited by 55 (9.17 per year)
C Vendrely, T Scheibel (2007) Biotechnological production of spider-silk proteins enables new applications. Macromolecular bioscience, Wiley Online Library, cited by 219 (18.25 per year)
Y Shang, J Wang, T Ikeda, L Jiang (2019) Bio-inspired liquid crystal actuator materials. Journal of Materials Chemistry C, pubs.rsc.org, cited by 1 (1.00 per year)
P Liu, Y Zhang, S Liu, Y Zhang, Z Du, L Qu (2019) Bio-inspired fabrication of fire-retarding, magnetic-responsive, superhydrophobic sponges for oil and organics collection. Applied Clay Science, Elsevier, cited by 2 (2.00 per year)
RJ Wood (2014) The challenge of manufacturing between Macro and Micro: classic ways of folding paper into dynamic shapes--origami, pop-up books--inspire methods to engineer …. American Scientist, go.galegroup.com, cited by 18 (3.60 per year)
J Hughes, F Iida (2017) 3D Printed Sensorized Soft Robotic Manipulator Design. Annual Conference Towards Autonomous Robotic …, Springer, cited by 1 (0.50 per year)
K Glette, M Hovin (2010) Evolution of artificial muscle-based robotic locomotion in PhysX. 2010 IEEE/RSJ International Conference on …, ieeexplore.ieee.org, cited by 15 (1.67 per year)
J Cai, S Chen, L Cui, C Chen, B Su, ... (2015) Tailored Porphyrin Assembly at the Oil–Aqueous Interface Based on the Receding of Three-Phase Contact Line of Droplet Template. Advanced Materials …, Wiley Online Library, cited by 13 (3.25 per year)
A Žemaitis, M Gaidys, P Gečys, G Račiukaitis, ... (2019) Rapid high-quality 3D micro-machining by optimised efficient ultrashort laser ablation. Optics and Lasers in …, Elsevier, cited by 5 (5.00 per year)
W Yuan, Y Yao, L Keer, Y Jiao, J Yu, Q Li, ... (2019) 3D-printed biomimetic surface structures with abnormal friction properties. Extreme Mechanics …, Elsevier, cited by 1 (1.00 per year)
R Kumar, M Kiristi, F Soto, J Li, VV Singh, J Wang (2015) Self-propelled screen-printable catalytic swimmers. RSC Advances, pubs.rsc.org, cited by 11 (2.75 per year)
M Shishehbor, PD Zavattieri (2019) Effects of interface properties on the mechanical properties of bio-inspired cellulose nanocrystal (CNC)-based materials. Journal of the Mechanics and Physics of …, Elsevier, cited by 8 (8.00 per year)
SFA Acquah, BE Leonhardt, ... (2016) Carbon nanotubes and graphene as additives in 3D printing. … -current progress of …, books.google.com, cited by 8 (2.67 per year)

YL Park, RJ Wood (2013) Smart pneumatic artificial muscle actuator with embedded microfluidic sensing. SENSORS, 2013 IEEE, ieeexplore.ieee.org, cited by 44 (7.33 per year)
B Li, B Yu, Q Ye, F Zhou (2014) Tapping the potential of polymer brushes through synthesis. Accounts of chemical research, ACS Publications, cited by 58 (11.60 per year)
MM Porter, J McKittrick (2014) It's tough to be strong: Advances. Am. Ceram. Soc. Bull, researchgate.net, cited by 17 (3.40 per year)
C Li, C Yu, D Hao, L Wu, Z Dong, ... (2018) Smart Liquid Transport on Dual Biomimetic Surface via Temperature Fluctuation Control. Advanced Functional …, Wiley Online Library, cited by 6 (6.00 per year)
M Castilho, J Rodrigues, I Pires, B Gouveia, ... (2015) Fabrication of individual alginate-TCP scaffolds for bone tissue engineering by means of powder printing. …, iopscience.iop.org, cited by 36 (9.00 per year)
A Bulgarelli, G Toscana, LO Russo, ... (2016) A low-cost open source 3D-printable dexterous anthropomorphic robotic hand with a parallel spherical joint wrist for sign languages reproduction. International …, journals.sagepub.com, cited by 3 (1.00 per year)
RC Luo, YW Perng, PK Tseng (2017) 3D printing process for multi-heterogeneous objects fabrication. 2017 IEEE/SICE International …, ieeexplore.ieee.org, cited by 1 (0.50 per year)
Y Zhang, J Zhu, Z Wang, Y Zhou, X Zhang (2015) Constructing a 3D-printable, bioceramic sheathed articular spacer assembly for infected hip arthroplasty. Journal of Medical Hypotheses …, Elsevier, cited by 7 (1.75 per year)
RG Wells (2008) The role of matrix stiffness in regulating cell behavior. Hepatology, Wiley Online Library, cited by 655 (59.55 per year)
L Wang, Y Jian, X Le, W Lu, C Ma, J Zhang, ... (2018) Actuating and memorizing bilayer hydrogels for a self-deformed shape memory function. Chemical …, pubs.rsc.org, cited by 16 (16.00 per year)
CM Niemeyer, B Ceyhan (2001) DNA-directed functionalization of colloidal gold with proteins. Angewandte Chemie International …, Wiley Online Library, cited by 214 (11.89 per year)
Y Si, Z Dong, L Jiang (2018) Bioinspired designs of superhydrophobic and superhydrophilic materials. ACS central science, ACS Publications, cited by 11 (11.00 per year)
HK Yap, HY Ng, CH Yeow (2016) High-force soft printable pneumatics for soft robotic applications. Soft Robotics, liebertpub.com, cited by 103 (34.33 per year)
SR Shin, SM Jung, M Zalabany, K Kim, P Zorlutuna, ... (2013) Carbon-nanotube-embedded hydrogel sheets for engineering cardiac constructs and bioactuators. ACS …, ACS Publications, cited by 401 (66.83 per year)
G Stoychev, S Zakharchenko, S Turcaud, ... (2012) Shape-programmed folding of stimuli-responsive polymer bilayers. ACS …, ACS Publications, cited by 181 (25.86 per year)
R Niiyama (2016) Micro CT study of soft/elastic structures of beetle toward insect-inspired robotics. 2016 IEEE/SICE International Symposium on …, ieeexplore.ieee.org, cited by 1 (0.33 per year)
K Pillearachchige, T Pereira, ... (2016) Bio-inspired multitasking robotic gripper design and development. 2016 12th IEEE/ASME …, ieeexplore.ieee.org, cited by 1 (0.33 per year)
D Rogozhnikov, PJ O'Brien, S Elahipanah, ... (2016) Scaffold free bio-orthogonal assembly of 3-dimensional cardiac tissue via cell surface engineering. Scientific reports, nature.com, cited by 28 (9.33 per year)
J Isermann, S Ulrich, R Bruns Design of highly elastic bending actuators for use in bio-inspired picking gripper. Conference Proceedings ACTUATOR, researchgate.net, cited by 1 (0.00 per year)

Y Luo, D Zhang, Y Liu, Y Li, EYK Ng (2015) Chemical, mechanical and hydrodynamic properties research on composite drag reduction surface based on biological sharkskin morphology and mucus nanolong …. Journal of Mechanics in …, World Scientific, cited by 17 (4.25 per year)
J Wang, Q Cheng, Z Tang (2012) Layered nanocomposites inspired by the structure and mechanical properties of nacre. Chemical Society Reviews, pubs.rsc.org, cited by 312 (44.57 per year)
F Bonnet, Y Kato, J Halloy, F Mondada (2016) Infiltrating the zebrafish swarm: Design, implementation and experimental tests of a miniature robotic fish lure for fish–robot interaction studies. Artificial Life and Robotics, Springer, cited by 17 (5.67 per year)
TH Park, ML Shuler (2003) Integration of cell culture and microfabrication technology. Biotechnology progress, Wiley Online Library, cited by 576 (36.00 per year)
Q Zhao, Y Wang, H Cui, X Du (2019) Bio-inspired sensing and actuating materials. Journal of Materials Chemistry C, pubs.rsc.org, cited by 1 (1.00 per year)
MR Mansouri, H Montazerian, S Schmauder, ... (2018) 3D-printed multimaterial composites tailored for compliancy and strain recovery. Composite …, Elsevier, cited by 10 (10.00 per year)
JP Wissman, K Sampath, SE Freeman, CA Rohde (2019) Capacitive Bio-Inspired Flow Sensing Cupula. Sensors, mdpi.com, cited by 1 (1.00 per year)
JJ Wu, YG Tan, GF Ma (2015) 3D printing monitoring platform based on the Internet of Things., IET, cited by 1 (0.25 per year)
L Ionov (2013) Biomimetic hydrogel-based actuating systems. Advanced Functional Materials, Wiley Online Library, cited by 243 (40.50 per year)
J Xiong, R Mines, R Ghosh, A Vaziri, ... (2015) Advanced micro-lattice materials. Advanced …, Wiley Online Library, cited by 66 (16.50 per year)
H Banerjee, ZTH Tse, H Ren (2018) Soft robotics with compliance and adaptation for biomedical applications and forthcoming challenges. International Journal of Robotics …, researchgate.net, cited by 11 (11.00 per year)
F Luo, TL Sun, T Nakajima, T Kurokawa, ... (2015) Oppositely charged polyelectrolytes form tough, self-healing, and rebuildable hydrogels. Advanced …, Wiley Online Library, cited by 232 (58.00 per year)
H Tao, DL Kaplan, FG Omenetto (2012) Silk materials–a road to sustainable high technology. Advanced materials, Wiley Online Library, cited by 294 (42.00 per year)
M Bukka, PJ Rednam, M Sinha (2018) Drug-eluting balloon: design, technology and clinical aspects. Biomedical Materials, iopscience.iop.org, cited by 3 (3.00 per year)
M Saari, B Cox, M Galla, PS Krueger, ... (2015) Multi-material additive manufacturing of robot components with integrated sensor arrays. … and Bio-inspired …, spiedigitallibrary.org, cited by 11 (2.75 per year)
M Jaffar-Bandjee, J Casas, G Krijnen (2018) Additive manufacturing: state of the art and potential for insect science. Current opinion in insect science, Elsevier, cited by 5 (5.00 per year)
B Zhu, H Wang, WR Leow, Y Cai, XJ Loh, ... (2016) Silk fibroin for flexible electronic devices. Advanced …, Wiley Online Library, cited by 145 (48.33 per year)
SI Roohani-Esfahani, P Newman, H Zreiqat (2016) Design and fabrication of 3D printed scaffolds with a mechanical strength comparable to cortical bone to repair large bone defects. Scientific reports, nature.com, cited by 109 (36.33 per year)
CS Ong, P Yesantharao, CY Huang, G Mattson, ... (2018) 3D bioprinting using stem cells. Pediatric …, nature.com, cited by 20 (20.00 per year)

T Li, X Hu, Y Chen, L Wang (2017) Harnessing out-of-plane deformation to design 3D architected lattice metamaterials with tunable Poisson's ratio. Scientific reports, nature.com, cited by 19 (9.50 per year)
YL Zhang, H Xia, E Kim, HB Sun (2012) Recent developments in superhydrophobic surfaces with unique structural and functional properties. Soft Matter, pubs.rsc.org, cited by 255 (36.43 per year)
W Yang, IH Chen, B Gludovatz, ... (2013) Natural flexible dermal armor. Advanced …, Wiley Online Library, cited by 196 (32.67 per year)
SA Suresh, DL Christensen, ... (2015) Surface and shape deposition manufacturing for the fabrication of a curved surface gripper. Journal of …, … .asmedigitalcollection.asme.org, cited by 21 (5.25 per year)
X Hou (2016) Smart Gating Multi-Scale Pore/Channel-Based Membranes. Advanced Materials, Wiley Online Library, cited by 89 (29.67 per year)
LM Weber, CG Lopez, KS Anseth (2009) Effects of PEG hydrogel crosslinking density on protein diffusion and encapsulated islet survival and function. … Part A: An Official Journal of …, Wiley Online Library, cited by 188 (18.80 per year)
L Zhai, MC Berg, FC Cebeci, Y Kim, JM Milwid, ... (2006) Patterned superhydrophobic surfaces: toward a synthetic mimic of the Namib Desert beetle. Nano Letters, ACS Publications, cited by 676 (52.00 per year)
H Schoof, J Apel, I Heschel, ... (2001) Control of pore structure and size in freeze-dried collagen sponges. Journal of Biomedical …, Wiley Online Library, cited by 374 (20.78 per year)
H Zhao, K O'Brien, S Li, RF Shepherd (2016) Optoelectronically innervated soft prosthetic hand via stretchable optical waveguides. Science Robotics, pdfs.semanticscholar.org, cited by 151 (50.33 per year)
AE Eiben, N Bredeche, M Hoogendoorn, ... (2013) The triangle of life: Evolving robots in real-time and real-space. Artificial Life …, MIT Press, cited by 30 (5.00 per year)
J Fuh (2013) Micro-and Bio-rapid prototyping using drop-on-demand 3D printing. Handbook of Manufacturing Engineering and …, Springer, cited by 1 (0.17 per year)
CC Huang, X Wu, H Liu, B Aldalali, JA Rogers, H Jiang (2014) Large-field-of-view wide-spectrum artificial reflecting superposition compound eyes. Small, Wiley Online Library, cited by 43 (8.60 per year)
JC Case, EL White, RK Kramer (2015) Soft material characterization for robotic applications. Soft Robotics, liebertpub.com, cited by 65 (16.25 per year)
D Yang, Z Zhao, F Bai, S Wang, ... (2017) Promoting cell migration in tissue engineering scaffolds with graded channels. Advanced healthcare …, Wiley Online Library, cited by 8 (4.00 per year)
E Thompson-Bean, R Das, ... (2016) Methodology for designing and manufacturing complex biologically inspired soft robotic fluidic actuators: prosthetic hand case study. Bioinspiration & …, iopscience.iop.org, cited by 5 (1.67 per year)
J Xue, ZK Zhou, Z Wei, R Su, J Lai, J Li, C Li, ... (2015) Scalable, full-colour and controllable chromotropic plasmonic printing. Nature …, nature.com, cited by 87 (21.75 per year)
J O'Donnell, M Kim, HS Yoon (2017) A review on electromechanical devices fabricated by additive manufacturing. Journal of …, … .asmedigitalcollection.asme.org, cited by 24 (12.00 per year)
WS Chu, KT Lee, SH Song, MW Han, JY Lee, ... (2012) Review of biomimetic underwater robots using smart actuators. International journal of …, Springer, cited by 210 (30.00 per year)

X Li, J He, W Zhang, N Jiang, D Li (2016) Additive manufacturing of biomedical constructs with biomimetic structural organizations. Materials, mdpi.com, cited by 12 (4.00 per year)
JB Fan, Y Song, S Wang, J Meng, ... (2015) Directly coating hydrogel on filter paper for effective oil–water separation in highly acidic, alkaline, and salty environment. Advanced Functional …, Wiley Online Library, cited by 141 (35.25 per year)
S Hengsbach, AD Lantada (2014) Rapid prototyping of multi-scale biomedical microdevices by combining additive manufacturing technologies. Biomedical microdevices, Springer, cited by 38 (7.60 per year)
T Yang, W Wang, H Zhang, X Li, J Shi, Y He, Q Zheng, ... (2015) Tactile sensing system based on arrays of graphene woven microfabrics: electromechanical behavior and electronic skin application. ACS …, ACS Publications, cited by 125 (31.25 per year)
GH Lee, TM Choi, B Kim, SH Han, JM Lee, SH Kim (2017) Chameleon-inspired mechanochromic photonic films composed of non-close-packed colloidal arrays. ACS nano, ACS Publications, cited by 24 (12.00 per year)
F Ghorbani, A Zamanian, A Behnamghader, ... (2019) Bone-like hydroxyapatite mineralization on the bio-inspired PDA nanoparticles using microwave irradiation. Surfaces and …, Elsevier, cited by 3 (3.00 per year)
QP Pham, U Sharma, AG Mikos (2006) Electrospinning of polymeric nanofibers for tissue engineering applications: a review. Tissue engineering, liebertpub.com, cited by 2068 (159.08 per year)
F Guo, SS Gan (2006) Guo du., mae.rutgers.edu, cited by 6 (0.46 per year)
H Cho, S Mayer, E Pöselt, M Susoff, PJ in't Veld, ... (2017) Deformation mechanisms of thermoplastic elastomers: Stress-strain behavior and constitutive modeling. Polymer, Elsevier, cited by 11 (5.50 per year)
DB Berry, S You, J Warner, LR Frank, ... (2017) A 3D Tissue-Printing Approach for Validation of Diffusion Tensor Imaging in Skeletal Muscle. … Engineering Part A, liebertpub.com, cited by 7 (3.50 per year)
J Liu, Y Wang, D Zhao, C Zhang, ... (2014) Design and fabrication of an IPMC-embedded tube for minimally invasive surgery applications. … Polymer Actuators and …, spiedigitallibrary.org, cited by 8 (1.60 per year)
S Araya, E Zolotovsky, M Gidekel (2012) Living Architecture: Micro performances of bio fabrication., papers.cumincad.org, cited by 7 (1.00 per year)
E García-Tuñón, E Feilden, H Zheng, ... (2017) Graphene oxide: an all-in-one processing additive for 3D printing. … applied materials & …, ACS Publications, cited by 9 (4.50 per year)
T Ramanathan, H Liu, ... (2005) Functionalized SWNT/polymer nanocomposites for dramatic property improvement. Journal of Polymer Science …, Wiley Online Library, cited by 331 (23.64 per year)
R Akbarzadeh, AM Yousefi (2014) Effects of processing parameters in thermally induced phase separation technique on porous architecture of scaffolds for bone tissue engineering. Journal of biomedical materials …, Wiley Online Library, cited by 74 (14.80 per year)
SJ Jeon, AW Hauser, RC Hayward (2017) Shape-morphing materials from stimuli-responsive hydrogel hybrids. Accounts of chemical …, ACS Publications, cited by 100 (50.00 per year)
R Dittrich, G Tomandl, F Despang, ... (2007) Scaffolds for hard tissue engineering by ionotropic gelation of alginate–influence of selected preparation parameters. Journal of the …, Wiley Online Library, cited by 50 (4.17 per year)

M Frey, D Widner, JS Segmehl, K Casdorff, ... (2018) Delignified and densified cellulose bulk materials with excellent tensile properties for sustainable engineering. … applied materials & …, ACS Publications, cited by 21 (21.00 per year)
S Park, K Park (2016) Engineered polymeric hydrogels for 3D tissue models. Polymers, mdpi.com, cited by 14 (4.67 per year)
Y Lu, S Yi, Y Liu, Y Ji (2016) A novel path planning method for biomimetic robot based on deep learning. Assembly automation, emeraldinsight.com, cited by 9 (3.00 per year)
H Leon-Rodriguez, VH Le, SY Ko, ... (2015) Ferrofluid soft-robot bio-inspired by Amoeba locomotion. … and Systems (ICCAS …, ieeexplore.ieee.org, cited by 1 (0.25 per year)
C Pang, C Lee, KY Suh (2013) Recent advances in flexible sensors for wearable and implantable devices. Journal of Applied Polymer Science, Wiley Online Library, cited by 256 (42.67 per year)
R Fuhrer, EK Athanassiou, NA Luechinger, WJ Stark (2009) Crosslinking metal nanoparticles into the polymer backbone of hydrogels enables preparation of soft, magnetic field-driven actuators with muscle-like flexibility. Small, Wiley Online Library, cited by 207 (20.70 per year)
Q Li, AP Alloncle, D Grojo, P Delaporte (2017) Generating liquid nanojets from copper by dual laser irradiation for ultra-high resolution printing. Optics express, osapublishing.org, cited by 11 (5.50 per year)
A Moosavian, MH Malakooti, J Lin, ... (2017) Design and Manufacturing of a Morphing Trailing Edge Using a 3D-Printed Piezoelectric Polymer. 8th Conference on …, congress.cimne.com, cited by 1 (0.50 per year)
DJ Lipomi, BCK Tee, M Vosgueritchian, ... (2011) Stretchable organic solar cells. Advanced …, Wiley Online Library, cited by 626 (78.25 per year)
Z Chen (2017) A review on robotic fish enabled by ionic polymer–metal composite artificial muscles. Robotics and biomimetics, Springer, cited by 8 (4.00 per year)
C Yu, C Li, C Gao, Z Dong, L Wu, L Jiang (2018) Time-dependent liquid transport on a biomimetic topological surface. ACS nano, ACS Publications, cited by 14 (14.00 per year)
J Go, AJ Hart (2016) A framework for teaching the fundamentals of additive manufacturing and enabling rapid innovation. Additive Manufacturing, Elsevier, cited by 72 (24.00 per year)
L Margheri, B Trimmer (2014) Soft robotics community events: Meeting different backgrounds for common challenges. Soft Robotics, liebertpub.com, cited by 9 (1.80 per year)
F Roger, P Krawczak (2015) 3D-printing of thermoplastic structures by FDM using heterogeneous infill and multi-materials: An integrated design-advanced manufacturing approach for factories of …. Congrès français de mécanique, documents.irevues.inist.fr, cited by 2 (0.50 per year)
A Andrearczyk, G Żywica (2016) A concept of a test stand for the investigation of a 3D printed turbochargers and selected fluid-flow machinery. Transactions of the Institute of Fluid-Flow …, infona.pl, cited by 3 (1.00 per year)
T Xie, X Xiao, YT Cheng (2009) Revealing triple-shape memory effect by polymer bilayers. Macromolecular Rapid …, Wiley Online Library, cited by 189 (18.90 per year)
AE Gayduk, VY Prinz, VA Seleznev, ... (2016) Large-area multilayer infrared nano-wire grid polarizers. Infrared Physics & …, Elsevier, cited by 5 (1.67 per year)
M Rogóż, H Zeng, C Xuan, DS Wiersma, ... (2016) Light-driven soft robot mimics caterpillar locomotion in natural scale. Advanced Optical …, Wiley Online Library, cited by 67 (22.33 per year)

Y Luo, X Xu, D Li, W Song (2016) Recent developments in fabricating drag reduction surfaces covering biological sharkskin morphology. Reviews in Chemical Engineering, degruyter.com, cited by 10 (3.33 per year)
SS Liao, FZ Cui, W Zhang, ... (2004) Hierarchically biomimetic bone scaffold materials: nano-HA/collagen/PLA composite. Journal of Biomedical …, Wiley Online Library, cited by 488 (32.53 per year)
H Zhang, JK Nagel, A Al-Qas, E Gibbons, ... (2018) Additive Manufacturing with Bioinspired Sustainable Product Design: A Conceptual Model. Procedia Manufacturing, Elsevier, cited by 6 (6.00 per year)
A Davoudinejad, MM Ribo, DB Pedersen, ... (2017) Biological features produced by additive manufacturing processes using vat photopolymerization method. Joint Special Interest …, researchgate.net, cited by 3 (1.50 per year)
Z Zhao, S Zhuo, R Fang, L Zhang, X Zhou, ... (2018) Dual-Programmable Shape-Morphing and Self-Healing Organohydrogels Through Orthogonal Supramolecular Heteronetworks. Advanced …, Wiley Online Library, cited by 7 (7.00 per year)
M Milwich, T Speck, O Speck, ... (2006) Biomimetics and technical textiles: solving engineering problems with the help of nature's wisdom. American Journal of …, Wiley Online Library, cited by 132 (10.15 per year)
CJ Bettinger, R Langer, ... (2009) Engineering substrate topography at the micro-and nanoscale to control cell function. Angewandte Chemie …, Wiley Online Library, cited by 1035 (103.50 per year)
Y Zhao, M Qin, A Wang, D Kim (2013) Bioinspired superhydrophobic carbonaceous hairy microstructures with strong water adhesion and high gas retaining capability. Advanced Materials, Wiley Online Library, cited by 20 (3.33 per year)
C Howell, TL Vu, JJ Lin, S Kolle, N Juthani, ... (2014) Self-replenishing vascularized fouling-release surfaces. … applied materials & …, ACS Publications, cited by 89 (17.80 per year)
L Ren, B Li, Z Song, Q Liu, L Ren, X Zhou (2019) Bioinspired fiber-regulated composite with tunable permanent shape and shape memory properties via 3d magnetic printing. Composites Part B: Engineering, Elsevier, cited by 1 (1.00 per year)
Y Yang, Y Chen (2017) Innovative design of embedded pressure and position sensors for soft actuators. IEEE Robotics and Automation Letters, ieeexplore.ieee.org, cited by 9 (4.50 per year)
S Harada, W Honda, T Arie, S Akita, K Takei (2014) Fully printed, highly sensitive multifunctional artificial electronic whisker arrays integrated with strain and temperature sensors. ACS nano, ACS Publications, cited by 151 (30.20 per year)
T Pinto, L Cai, C Wang, X Tan (2017) CNT-based sensor arrays for local strain measurements in soft pneumatic actuators. International Journal of Intelligent Robotics …, Springer, cited by 7 (3.50 per year)
K Sanderson (2015) Artificial armour. Nature, nature.com, cited by 9 (2.25 per year)
S Sant, MJ Hancock, JP Donnelly, D Iyer, ... (2010) Biomimetic gradient hydrogels for tissue engineering. … Canadian journal of …, Wiley Online Library, cited by 197 (21.89 per year)
Z Li, H Yu, H Ren, PWY Chiu, ... (2015) A novel constrained tendon-driven serpentine manipulator. 2015 IEEE/RSJ …, ieeexplore.ieee.org, cited by 11 (2.75 per year)
H Zhao, Z Yang, L Guo (2018) Nacre-inspired composites with different macroscopic dimensions: strategies for improved mechanical performance and applications. NPG Asia Materials, nature.com, cited by 9 (9.00 per year)

L Wu, Z Dong, H Du, C Li, NX Fang, Y Song (2018) Bioinspired Ultra-Low Adhesive Energy Interface for Continuous 3D Printing: Reducing Curing Induced Adhesion. Research, spj.sciencemag.org, cited by 3 (3.00 per year)
X Pan, Q Wang, D Ning, L Dai, K Liu, Y Ni, ... (2018) Ultraflexible self-healing guar gum-glycerol hydrogel with injectable, antifreeze, and strain-sensitive properties. ACS Biomaterials …, ACS Publications, cited by 10 (10.00 per year)
L Roseti, V Parisi, M Petretta, C Cavallo, ... (2017) Scaffolds for bone tissue engineering: state of the art and new perspectives. Materials Science and …, Elsevier, cited by 140 (70.00 per year)
S Daynes, S Feih, WF Lu, J Wei (2017) Optimisation of functionally graded lattice structures using isostatic lines. Materials & Design, Elsevier, cited by 30 (15.00 per year)
R Gautam, S Idapalapati, S Feih (2018) Printing and characterisation of Kagome lattice structures by fused deposition modelling. Materials & Design, Elsevier, cited by 10 (10.00 per year)
S Bukner, F Dialami, L Ding, ... (2015) Bio-inspired design to support reduced energy consumption via the 'light weighting'of machine system elements. International Journal of …, eprints.uwe.ac.uk, cited by 2 (0.50 per year)
A Ovsianikov, A Khademhosseini, V Mironov (2018) The synergy of scaffold-based and scaffold-free tissue engineering strategies. Trends in biotechnology, Elsevier, cited by 24 (24.00 per year)
VT Widyaya, EK Riga, C Müller, K Lienkamp (2018) Submicrometer-Sized, 3D Surface-Attached Polymer Networks by Microcontact Printing: Using UV-Cross-Linking Efficiency To Tune Structure Height. Macromolecules, ACS Publications, cited by 2 (2.00 per year)
Y Deng, J Kuiper (2017) Functional 3D tissue engineering scaffolds: materials, technologies, and applications., books.google.com, cited by 9 (4.50 per year)
L Mogas-Soldevila, J Duro-Royo, ... (2015) Designing the ocean pavilion: Biomaterial templating of structural, manufacturing, and environmental performance. Proceedings of IASS …, ingentaconnect.com, cited by 8 (2.00 per year)
D Rebolj, M Fischer, D Endy, T Moore, ... (2011) Can we grow buildings? Concepts and requirements for automated nano-to meter-scale building. Advanced engineering …, Elsevier, cited by 33 (4.13 per year)
H Philamore, J Rossiter, A Stinchcombe, ... (2015) Row-bot: An energetically autonomous artificial water boatman. 2015 IEEE/RSJ …, ieeexplore.ieee.org, cited by 9 (2.25 per year)
AK Rajasekharan, R Bordes, C Sandström, M Ekh, ... (2017) Hierarchical and Heterogeneous Bioinspired Composites—Merging Molecular Self-Assembly with Additive Manufacturing. Small, Wiley Online Library, cited by 12 (6.00 per year)
E Kanhere, N Wang, AGP Kottapalli, ... (2016) Crocodile-inspired dome-shaped pressure receptors for passive hydrodynamic sensing. Bioinspiration & …, iopscience.iop.org, cited by 10 (3.33 per year)
ML Burroughs, KB Freckleton, ... (2016) A sarrus-based passive mechanism for rotorcraft perching. Journal of …, … .asmedigitalcollection.asme.org, cited by 10 (3.33 per year)
A Pacifici, L Laino, M Gargari, F Guzzo, ... (2018) Decellularized hydrogels in bone tissue engineering: a topical review. … journal of medical …, ncbi.nlm.nih.gov, cited by 5 (5.00 per year)
A du Plessis, C Broeckhoven (2018) Looking deep into nature: A review of micro-computed tomography in biomimicry. Acta biomaterialia, Elsevier, cited by 9 (9.00 per year)

F Simone, G Rizzello, S Seelecke (2017) Metal muscles and nerves—a self-sensing SMA-actuated hand concept. Smart Materials and …, iopscience.iop.org, cited by 17 (8.50 per year)
P Egan, SJ Ferguson, K Shea (2016) Design and 3D printing of hierarchical tissue engineering scaffolds based on mechanics and biology perspectives. ASME 2016 …, … .asmedigitalcollection.asme.org, cited by 5 (1.67 per year)
HJ Lee, YB Kim, SH Ahn, JS Lee, ... (2015) A new approach for fabricating collagen/ECM-based bioinks using preosteoblasts and human adipose stem cells. Advanced …, Wiley Online Library, cited by 39 (9.75 per year)
Z Deng, T Hu, Q Lei, J He, PX Ma, ... (2019) Stimuli-Responsive Conductive Nanocomposite Hydrogels with High Stretchability, Self-Healing, Adhesiveness, and 3D Printability for Human Motion Sensing. ACS applied materials & …, ACS Publications, cited by 5 (5.00 per year)
M Kim, H Yun, GH Kim (2017) Electric-field assisted 3D-fibrous bioceramic-based scaffolds for bone tissue regeneration: Fabrication, characterization, and in vitro cellular activities. Scientific reports, nature.com, cited by 9 (4.50 per year)
M Mirkhalaf, T Zhou, F Barthelat (2018) Simultaneous improvements of strength and toughness in topologically interlocked ceramics. Proceedings of the …, National Acad Sciences, cited by 12 (12.00 per year)
F Luo, TL Sun, T Nakajima, T Kurokawa, ... (2015) Free reprocessability of tough and self-healing hydrogels based on polyion complex. ACS Macro …, ACS Publications, cited by 48 (12.00 per year)
Y Zhang, L Tao, S Li, Y Wei (2011) Synthesis of multiresponsive and dynamic chitosan-based hydrogels for controlled release of bioactive molecules. Biomacromolecules, ACS Publications, cited by 323 (40.38 per year)
FJ Martin-Martinez, K Jin, D López Barreiro, ... (2018) The rise of hierarchical nanostructured materials from renewable sources: Learning from nature. ACS …, ACS Publications, cited by 9 (9.00 per year)
L Liu, Y Li (2018) Predicting the mixed-mode I/II spatial damage propagation along 3D-printed soft interfacial layer via a hyperelastic softening model. Journal of the Mechanics and Physics of Solids, Elsevier, cited by 7 (7.00 per year)
D Correa, A Menges (2017) Fused Filament Fabrication for Multi-Kinematic-State Climate-Responsive Aperture. Fabricate 2017, JSTOR, cited by 3 (1.50 per year)
W Wang, L Yao, CY Cheng, T Zhang, ... (2017) Harnessing the hygroscopic and biofluorescent behaviors of genetically tractable microbial cells to design biohybrid wearables. Science …, advances.sciencemag.org, cited by 22 (11.00 per year)
N Deoray, B Kandasubramanian (2018) Review on three-dimensionally emulated fiber-embedded lactic acid polymer composites: opportunities in engineering sector. Polymer-Plastics Technology …, Taylor & Francis, cited by 9 (9.00 per year)
H Wang, B Zhu, H Wang, X Ma, Y Hao, X Chen (2016) Ultra-Lightweight Resistive Switching Memory Devices Based on Silk Fibroin. Small, Wiley Online Library, cited by 35 (11.67 per year)
C Park, KH Kim, YM Lee, W Giannobile, ... (2017) 3D printed, microgroove pattern-driven generation of oriented ligamentous architectures. International journal of …, mdpi.com, cited by 4 (2.00 per year)
J Lalevée, JP Fouassier (2018) Photopolymerisation Initiating Systems., books.google.com, cited by 11 (11.00 per year)

D Joung, V Truong, CC Neitzke, SZ Guo, ... (2018) 3D Printed Stem-Cell Derived Neural Progenitors Generate Spinal Cord Scaffolds. Advanced Functional …, Wiley Online Library, cited by 11 (11.00 per year)
J Najem, B Akle, SA Sarles, DJ Leo (2011) Design and development of a biomimetic jellyfish robot that features ionic polymer metal composites actuators. ASME 2011 Conference on …, researchgate.net, cited by 7 (0.88 per year)
J Silva-Correia, JM Oliveira, ... (2011) Gellan gum-based hydrogels for intervertebral disc tissue-engineering applications. Journal of tissue …, Wiley Online Library, cited by 162 (20.25 per year)
S Fu, W Liu, S Liu, S Zhao, Y Zhu (2018) 3D printed porous β-Ca2SiO4 scaffolds derived from preceramic resin and their physicochemical and biological properties. Science and technology of …, Taylor & Francis, cited by 5 (5.00 per year)
D Lee (2018) Investigation of Laser Ablation on Acrylonitrile Butadiene Styrene Plastic Used for 3D Printing. Journal of Welding and Joining, e-sciencecentral.org, cited by 2 (2.00 per year)
PYV Leung (2017) Sugar 3D Printing: Additive Manufacturing with Molten Sugar for Investigating Molten Material Fed Printing. 3D Printing and Additive Manufacturing, liebertpub.com, cited by 2 (1.00 per year)
J Thiele, Y Ma, SMC Bruekers, S Ma, ... (2014) 25th anniversary article: designer hydrogels for cell cultures: a materials selection guide. Advanced …, Wiley Online Library, cited by 217 (43.40 per year)
A Bahmani, G Li, TL Willett, J Montesano (2019) Three-dimensional micromechanical assessment of bio-inspired composites with non-uniformly dispersed inclusions. Composite Structures, Elsevier, cited by 1 (1.00 per year)
N Biloria, JR Chang (2012) Hypercell: A bio-inspired information design framework for real-time adaptive spatial components., papers.cumincad.org, cited by 1 (0.14 per year)
RV Martinez, CR Fish, X Chen, ... (2012) Elastomeric origami: programmable paper-elastomer composites as pneumatic actuators. Advanced functional …, Wiley Online Library, cited by 334 (47.71 per year)
M Lei, CM Hamel, C Yuan, H Lu, HJ Qi (2018) 3D printed two-dimensional periodic structures with tailored in-plane dynamic responses and fracture behaviors. Composites Science and …, Elsevier, cited by 1 (1.00 per year)
TL Khuong, Z Gang, M Farid, R Yu, ZZ Sun, ... (2014) Tensile strength and flexural strength testing of acrylonitrile butadiene styrene (ABS) materials for biomimetic robotic applications. Journal of …, Trans Tech Publ, cited by 4 (0.80 per year)
Z Feng, M Yamato, T Akutsu, T Nakamura, ... (2003) Investigation on the mechanical properties of contracted collagen gels as a scaffold for tissue engineering. Artificial …, Wiley Online Library, cited by 117 (7.31 per year)
AM Pankonien, GW Reich, N Lindsley, ... (2017) 3D-Printed Wind Tunnel Flutter Model. 58th AIAA/ASCE/AHS …, arc.aiaa.org, cited by 9 (4.50 per year)
H Droogendijk, MJ de Boer, RGP Sanders, ... (2015) Bio-inspired hair-based inertial sensors. Biomimetic …, Elsevier, cited by 2 (0.50 per year)
H Yi, M Kang, MK Kwak, HE Jeong (2016) Simple and reliable fabrication of bioinspired mushroom-shaped micropillars with precisely controlled tip geometries. ACS applied materials & …, ACS Publications, cited by 15 (5.00 per year)
S Bauer, S Bauer-Gogonea, I Graz, ... (2014) 25th anniversary article: A soft future: From robots and sensor skin to energy harvesters. Advanced …, Wiley Online Library, cited by 417 (83.40 per year)

AR Studart (2012) Towards high-performance bioinspired composites. Advanced Materials, Wiley Online Library, cited by 214 (30.57 per year)
AP Haring, Y Tong, J Halper, ... (2018) Programming of multicomponent temporal release profiles in 3D printed polypills via core–shell, multilayer, and gradient concentration profiles. Advanced healthcare …, Wiley Online Library, cited by 9 (9.00 per year)
A Potnuru, Y Tadesse (2019) Investigation of polylactide and carbon nanocomposite filament for 3D printing. Progress in Additive Manufacturing, Springer, cited by 3 (3.00 per year)
RA Le Feuvre, NS Scrutton (2018) A living foundry for synthetic biological materials: a synthetic biology roadmap to new advanced materials. Synthetic and systems biotechnology, Elsevier, cited by 13 (13.00 per year)
H Yang, D Qi, Z Liu, BK Chandran, T Wang, ... (2016) Soft thermal sensor with mechanical adaptability. Advanced …, Wiley Online Library, cited by 63 (21.00 per year)
H Yi, FU Rehman, C Zhao, B Liu, N He (2016) Recent advances in nano scaffolds for bone repair. Bone research, nature.com, cited by 119 (39.67 per year)
Y Li, D Li, B Lu, D Gao, J Zhou (2015) Current status of additive manufacturing for tissue engineering scaffold. Rapid Prototyping Journal, emeraldinsight.com, cited by 13 (3.25 per year)
Q Cheng, M Wu, M Li, L Jiang, ... (2013) Ultratough artificial nacre based on conjugated cross-linked graphene oxide. Angewandte Chemie …, Wiley Online Library, cited by 260 (43.33 per year)
M Luo, EH Skorina, W Tao, F Chen, S Ozel, Y Sun, ... (2017) Toward modular soft robotics: Proprioceptive curvature sensing and sliding-mode control of soft bidirectional bending modules. Soft robotics, liebertpub.com, cited by 28 (14.00 per year)
IO Smith, XH Liu, LA Smith, ... (2009) Nanostructured polymer scaffolds for tissue engineering and regenerative medicine. Wiley Interdisciplinary …, Wiley Online Library, cited by 242 (24.20 per year)
J Zhu, H Cheng (2018) Recent development of flexible and stretchable antennas for bio-integrated electronics. Sensors, mdpi.com, cited by 4 (4.00 per year)
X Wang, Y Li, C Zhong (2015) Amyloid-directed assembly of nanostructures and functional devices for bionanoelectronics. Journal of Materials Chemistry B, pubs.rsc.org, cited by 19 (4.75 per year)
E Cohen, V Vikas, B Trimmer, ... (2015) Design methodologies for soft-material robots through additive manufacturing, from prototyping to locomotion. ASME 2015 …, … .asmedigitalcollection.asme.org, cited by 6 (1.50 per year)
N Pateromichelakis, A Mazel, MA Hache, ... (2014) Head-eyes system and gaze analysis of the humanoid robot Romeo. 2014 IEEE/RSJ …, ieeexplore.ieee.org, cited by 23 (4.60 per year)
R Scaffaro, F Lopresti, L Botta, A Maio (2016) Mechanical behavior of polylactic acid/polycaprolactone porous layered functional composites. Composites Part B: Engineering, Elsevier, cited by 33 (11.00 per year)
Y Hu, L He, Y Yin (2011) Magnetically responsive photonic nanochains. Angewandte Chemie International Edition, Wiley Online Library, cited by 97 (12.13 per year)
K Lin, L Yuan, D Gu (2019) Influence of laser parameters and complex structural features on the bio-inspired complex thin-wall structures fabricated by selective laser melting. Journal of Materials Processing Technology, Elsevier, cited by 2 (2.00 per year)
C Vyas, G Poologasundarampillai, J Hoyland, ... (2017) 3D printing of biocomposites for osteochondral tissue engineering. Biomedical …, Elsevier, cited by 3 (1.50 per year)

CT Kao, YJ Chen, HY Ng, A Lee, TH Huang, TF Lin, ... (2018) Surface modification of calcium silicate via mussel-inspired polydopamine and effective adsorption of extracellular matrix to promote osteogenesis differentiation for …. Materials, mdpi.com, cited by 3 (3.00 per year)

Y Cai, Q Lu, X Guo, S Wang, J Qiao, ... (2015) Salt-Tolerant Superoleophobicity on Alginate Gel Surfaces Inspired by Seaweed (Saccharina japonica). Advanced …, Wiley Online Library, cited by 69 (17.25 per year)

WT Brinkman, K Nagapudi, BS Thomas, ... (2003) Photo-cross-linking of type I collagen gels in the presence of smooth muscle cells: mechanical properties, cell viability, and function. …, ACS Publications, cited by 152 (9.50 per year)

S Duan, K Yang, Z Wang, M Chen, ... (2016) Fabrication of highly stretchable conductors based on 3D printed porous poly (dimethylsiloxane) and conductive carbon nanotubes/graphene network. … applied materials & …, ACS Publications, cited by 37 (12.33 per year)

P Chattopadhyay, SK Ghoshal (2018) Adhesion technologies of bio-inspired climbing robots: A survey. International Journal of Robotics and …, actapress.com, cited by 1 (1.00 per year)

M Weber, I Gonzalez de Torre, R Moreira, ... (2015) Multiple-step injection molding for fibrin-based tissue-engineered heart valves. … Engineering Part C …, liebertpub.com, cited by 24 (6.00 per year)

L Badarnah (2017) Form follows environment: biomimetic approaches to building envelope design for environmental adaptation. Buildings, mdpi.com, cited by 8 (4.00 per year)

JM Holzwarth, PX Ma (2011) Biomimetic nanofibrous scaffolds for bone tissue engineering. Biomaterials, Elsevier, cited by 482 (60.25 per year)

DJ Horst, SM Tebcherani, ET Kubaski, ... (2017) Bioactive Potential of 3D-Printed Oleo-Gum-Resin Disks: B. papyrifera, C. myrrha, and S. benzoin Loading Nanooxides—TiO2, P25, Cu2O, and MoO3. Bioinorganic chemistry …, hindawi.com, cited by 5 (2.50 per year)

GC Pidcock, M in het Panhuis (2012) Extrusion printing of flexible electrically conducting carbon nanotube networks. Advanced Functional Materials, Wiley Online Library, cited by 46 (6.57 per year)

M Mirkhalaf, B Ashrafi (2017) A numerical study on improving the specific properties of staggered composites by incorporating voids. Materials Today Communications, Elsevier, cited by 5 (2.50 per year)

L Cui, Y Zhang, J Wang, Y Ren, ... (2009) Ultra-Fast Fabrication of Colloidal Photonic Crystals by Spray Coating. Macromolecular rapid …, Wiley Online Library, cited by 82 (8.20 per year)

Y Zhao, X Zhao, B Tang, W Xu, J Li, ... (2010) Quantum-dot-tagged bioresponsive hydrogel suspension array for multiplex label-free DNA detection. Advanced Functional …, Wiley Online Library, cited by 132 (14.67 per year)

V Sharma, S Kumar, KL Reddy, ... (2016) Bioinspired functional surfaces for technological applications. Journal of Molecular …, World Scientific, cited by 8 (2.67 per year)

S Chowdhury, V Maldonado (2017) Bio-inspired Active and Passive Surface Flow Control for Aerodynamic Eciency. 47th AIAA Fluid Dynamics Conference, arc.aiaa.org, cited by 1 (0.50 per year)

K Wu, Z Zheng, S Zhang, L He, H Yao, X Gong, Y Ni (2019) Interfacial strength-controlled energy dissipation mechanism and optimization in impact-resistant nacreous structure. Materials & Design, Elsevier, cited by 4 (4.00 per year)

AD Marchese, RK Katzschmann, D Rus (2015) A recipe for soft fluidic elastomer robots. Soft Robotics, liebertpub.com, cited by 200 (50.00 per year)

JD Kiang, JH Wen, JC del Álamo, ... (2013) Dynamic and reversible surface topography influences cell morphology. Journal of Biomedical …, Wiley Online Library, cited by 32 (5.33 per year)
L Ren, Q Jiang, Z Chen, K Chen, S Xu, J Gao, ... (2017) Flexible microneedle array electrode using magnetorheological drawing lithography for bio-signal monitoring. Sensors and Actuators A …, Elsevier, cited by 6 (3.00 per year)
CS Sharma, A Sharma, M Madou (2010) Multiscale carbon structures fabricated by direct micropatterning of electrospun mats of SU-8 photoresist nanofibers. Langmuir, ACS Publications, cited by 75 (8.33 per year)
N Jorapur, R West, CB Williams, ... (2016) Design of fiber-reinforced cellular structures with tensegrity behavior manufactured using 3D printed sand molds. International Solid …, sffsymposium.engr.utexas.edu, cited by 1 (0.33 per year)
Y Si, T Wang, C Li, C Yu, N Li, C Gao, Z Dong, ... (2018) Liquids Unidirectional Transport on Dual-Scale Arrays. ACS nano, ACS Publications, cited by 7 (7.00 per year)
G Ficht, P Allgeuer, H Farazi, ... (2017) NimbRo-OP2: Grown-up 3D printed open humanoid platform for research. 2017 IEEE-RAS 17th …, ieeexplore.ieee.org, cited by 6 (3.00 per year)
MB Trimmer, PRH Ewoldt, M Kovac, H Lipson, N Lu, ... (2014) At the crossroads: interdisciplinary paths to soft robots. Soft …, liebertpub.com, cited by 13 (2.60 per year)
F Moreira, A Abundis, M Aguirre, J Castillo, ... (2018) An inchworm-inspired robot based on modular body, electronics and passive friction pads performing the two-anchor crawl gait. Journal of Bionic …, Springer, cited by 1 (1.00 per year)
B Winstone, T Pipe, C Melhuish, S Dogramadzi, ... (2015) Biomimetic tactile sensing capsule. … on Biomimetic and …, Springer, cited by 4 (1.00 per year)
B Natarajan, JW Gilman (2017) Bioinspired Bouligand cellulose nanocrystal composites: a review of mechanical properties. Philosophical Transactions of …, royalsocietypublishing.org, cited by 13 (6.50 per year)
M Bryant, J Fitzgerald, S Miller, ... (2014) Climbing robot actuated by meso-hydraulic artificial muscles. Active and Passive …, spiedigitallibrary.org, cited by 6 (1.20 per year)
EA Flores-Johnson, JG Carrillo, C Zhai, RA Gamboa, ... (2018) Microstructure and mechanical properties of hard Acrocomia mexicana fruit shell. Scientific reports, nature.com, cited by 7 (7.00 per year)
S Lapidot, S Meirovitch, S Sharon, A Heyman, ... (2012) Clues for biomimetics from natural composite materials. …, Future Medicine, cited by 32 (4.57 per year)
SB Kordmahale, J Kameoka (2015) Smart Soft Actuation System. Ann Materials Sci Eng, researchgate.net, cited by 4 (1.00 per year)
Y Si, C Yu, Z Dong, L Jiang (2018) Wetting and spreading: Fundamental theories to cutting-edge applications. Current opinion in colloid & interface science, Elsevier, cited by 9 (9.00 per year)
X Wang, Z Liu, T Zhang (2017) Flexible sensing electronics for wearable/attachable health monitoring. Small, Wiley Online Library, cited by 166 (83.00 per year)
M Islam, S Beverung, R Steward Jr (2017) Bio-inspired microdevices that mimic the human vasculature. Micromachines, mdpi.com, cited by 1 (0.50 per year)
S Ryu, P Lee, JB Chou, R Xu, R Zhao, AJ Hart, ... (2015) Extremely elastic wearable carbon nanotube fiber strain sensor for monitoring of human motion. ACS nano, ACS Publications, cited by 274 (68.50 per year)
A Elbaz, Z He, B Gao, J Chi, E Su, D Zhang, ... (2018) Recent biomedical applications of bio-sourced materials. Bio-design and …, Springer, cited by 4 (4.00 per year)

Y Zhang, CW Lo, JA Taylor, S Yang (2006) Replica molding of high-aspect-ratio polymeric nanopillar arrays with high fidelity. Langmuir, ACS Publications, cited by 196 (15.08 per year)
L García-Guzmán, L Távara, J Reinoso, J Justo, ... (2018) Fracture resistance of 3D printed adhesively bonded DCB composite specimens using structured interfaces: Experimental and theoretical study. Composite …, Elsevier, cited by 4 (4.00 per year)
J Sun, C Yun, B Cui, P Li, G Liu, X Wang, F Chu (2018) A facile approach for fabricating microstructured surface based on etched template by inkjet printing technology. Polymers, mdpi.com, cited by 3 (3.00 per year)
X Qu, M Gou, J Zaidan, K Zhang, S Chen (2014) Challenges and opportunities in developing nanoparticles for detoxification. Nanomedicine, Future Medicine, cited by 4 (0.80 per year)
R Belisle, C Yu, R Nagpal (2010) Mechanical design and locomotion of modular-expanding robots. Proceedings of the IEEE, academia.edu, cited by 10 (1.11 per year)
P Cai, WR Leow, X Wang, YL Wu, ... (2017) Programmable nano–bio interfaces for functional biointegrated devices. Advanced Materials, Wiley Online Library, cited by 60 (30.00 per year)
X Yu, SG Nurzaman, U Culha, F Iida (2014) Soft robotics education. Soft Robotics, liebertpub.com, cited by 6 (1.20 per year)
A Hengstenberg, A Blöchl, ID Dietzel, ... (2001) Spatially resolved detection of neurotransmitter secretion from individual cells by means of scanning electrochemical microscopy. Angewandte Chemie …, Wiley Online Library, cited by 122 (6.78 per year)
A Amendola, E Hernández-Nava, R Goodall, I Todd, ... (2015) On the additive manufacturing, post-tensioning and testing of bi-material tensegrity structures. Composite …, Elsevier, cited by 73 (18.25 per year)
P Allgeuer, H Farazi, G Ficht, M Schreiber, ... (2016) The igus humanoid open platform. KI-Künstliche …, Springer, cited by 7 (2.33 per year)
Q Yang, H Li, M Li, Y Li, S Chen, B Bao, ... (2017) Rayleigh instability-assisted satellite droplets elimination in inkjet printing. ACS applied materials …, ACS Publications, cited by 4 (2.00 per year)
P Ohta, L Valle, J King, K Low, J Yi, CG Atkeson, ... (2018) Design of a lightweight soft robotic arm using pneumatic artificial muscles and inflatable sleeves. Soft …, liebertpub.com, cited by 21 (21.00 per year)
YS Zhang, M Duchamp, R Oklu, ... (2016) Bioprinting the cancer microenvironment. ACS biomaterials …, ACS Publications, cited by 75 (25.00 per year)
F Libonati, AE Vellwock, F Ielmini, D Abliz, ... (2019) Bone-inspired enhanced fracture toughness of de novo fiber reinforced composites. Scientific reports, nature.com, cited by 1 (1.00 per year)
SK Gupta, G Fowler (2004) A step towards integrated product/process development of molded multi-material structures. Tools And Methods Of Competitive …, terpconnect.umd.edu, cited by 5 (0.33 per year)
H Banerjee, H Ren (2018) Electromagnetically responsive soft-flexible robots and sensors for biomedical applications and impending challenges. Electromagnetic Actuation and Sensing in Medical …, Springer, cited by 8 (8.00 per year)
F Liu, Q Chen, C Liu, Q Ao, X Tian, J Fan, H Tong, ... (2018) Natural polymers for organ 3D bioprinting. Polymers, mdpi.com, cited by 3 (3.00 per year)
L Feng, Z Zhang, Z Mai, Y Ma, B Liu, ... (2004) A super-hydrophobic and super-oleophilic coating mesh film for the separation of oil and water. Angewandte Chemie …, Wiley Online Library, cited by 1191 (79.40 per year)

F Baino, M Ferraris (2017) Learning from Nature: Using bioinspired approaches and natural materials to make porous bioceramics. International Journal of Applied Ceramic …, Wiley Online Library, cited by 11 (5.50 per year)
N Bhardwaj, D Chouhan, BB Mandal (2018) 3D functional scaffolds for skin tissue engineering. Functional 3D Tissue Engineering …, Elsevier, cited by 8 (8.00 per year)
M Guo, DV Gealy, J Liang, J Mahler, ... (2017) Design of parallel-jaw gripper tip surfaces for robust grasping. … on Robotics and …, ieeexplore.ieee.org, cited by 12 (6.00 per year)
C García-Saura, F de Borja Rodríguez, ... (2014) Design principles for cooperative robots with uncertainty-aware and resource-wise adaptive behavior. Conference on Biomimetic …, Springer, cited by 4 (0.80 per year)
C Zhang, W Wang, N Xi, Y Wang, L Liu (2018) Development and future challenges of bio-syncretic robots. Engineering, Elsevier, cited by 4 (4.00 per year)
JD Carrico, NW Traeden, ... (2015) Fused filament additive manufacturing of ionic polymer-metal composite soft active 3D structures. ASME 2015 …, … .asmedigitalcollection.asme.org, cited by 5 (1.25 per year)
UA Fiaz, N Toumi, JS Shamma (2017) Passive aerial grasping of ferrous objects. IFAC-PapersOnLine, Elsevier, cited by 6 (3.00 per year)
Z Dong, J Ma, L Jiang (2013) Manipulating and dispensing micro/nanoliter droplets by superhydrophobic needle nozzles. ACS nano, ACS Publications, cited by 61 (10.17 per year)
S Haque, S Md, M Whittaker, ... (2018) The applications of 3D printing in pulmonary drug delivery and treatment of respiratory disorders. Current pharmaceutical …, ingentaconnect.com, cited by 1 (1.00 per year)
A Iglesias, A Gálvez (2016) Nature-inspired swarm intelligence for data fitting in reverse engineering: recent advances and future trends. Nature-Inspired Computation in Engineering, Springer, cited by 5 (1.67 per year)
M Mao, J He, Y Lu, X Li, T Li, W Zhou, D Li (2018) Leaf-templated, microwell-integrated microfluidic chips for high-throughput cell experiments. Biofabrication, iopscience.iop.org, cited by 5 (5.00 per year)
Y Han, H Marvi, M Sitti (2015) Fiberbot: A miniature crawling robot using a directional fibrillar pad. 2015 IEEE International Conference on …, ieeexplore.ieee.org, cited by 6 (1.50 per year)
M Amjadi, A Pichitpajongkit, S Lee, S Ryu, I Park (2014) Highly stretchable and sensitive strain sensor based on silver nanowire–elastomer nanocomposite. ACS nano, ACS Publications, cited by 878 (175.60 per year)
L Zhang, G Yang, BN Johnson, X Jia (2018) Three-dimensional (3D) printed scaffold and material selection for bone repair. Acta biomaterialia, Elsevier, cited by 6 (6.00 per year)
GC Yeo (2019) A new vascular engineering strategy using 3D printed ice. Trends in biotechnology, Elsevier, cited by 1 (1.00 per year)
M Schneider, C Günter, A Taubert (2018) Co-Deposition of a hydrogel/calcium phosphate hybrid layer on 3D printed poly (lactic acid) scaffolds via dip coating: Towards automated biomaterials fabrication. Polymers, mdpi.com, cited by 3 (3.00 per year)
M Lapeyre, P Rouanet, PY Oudeyer (2013) Physical Human-like Interaction with the Acroban and Poppy Humanoid Platforms.., hal.inria.fr, cited by 4 (0.67 per year)
J Fu (2019) Hydrogel properties and applications. Journal of Materials Chemistry B, pubs.rsc.org, cited by 2 (2.00 per year)
B Wang, B Pan, G Lubineau (2018) Morphological evolution and internal strain mapping of pomelo peel using X-ray computed tomography and digital volume correlation. Materials & Design, Elsevier, cited by 8 (8.00 per year)

CM Chew, QY Lim, KS Yeo (2015) Development of propulsion mechanism for Robot Manta Ray. 2015 IEEE International …, ieeexplore.ieee.org, cited by 7 (1.75 per year)
X Lu, H Zhang, G Fei, B Yu, X Tong, H Xia, ... (2018) Liquid-Crystalline Dynamic Networks Doped with Gold Nanorods Showing Enhanced Photocontrol of Actuation. Advanced …, Wiley Online Library, cited by 24 (24.00 per year)
MR Cutkosky, S Kim (2009) Design and fabrication of multi-material structures for bioinspired robots. Philosophical Transactions of the …, royalsocietypublishing.org, cited by 91 (9.10 per year)
X Li, WC Chang, YJ Chao, R Wang, M Chang (2004) Nanoscale structural and mechanical characterization of a natural nanocomposite material: the shell of red abalone. Nano letters, ACS Publications, cited by 487 (32.47 per year)
AFT Winfield, J Timmis (2015) Evolvable robot hardware. Evolvable Hardware, Springer, cited by 5 (1.25 per year)
SW Hwang, G Park, H Cheng, JK Song, ... (2014) 25th anniversary article: materials for high-performance biodegradable semiconductor devices. Advanced …, Wiley Online Library, cited by 99 (19.80 per year)
Z Chen, Y Lin, W Lee, L Ren, B Liu, ... (2018) Additive manufacturing of honeybee-inspired microneedle for easy skin insertion and difficult removal. … applied materials & …, ACS Publications, cited by 3 (3.00 per year)
FW Baumann, M Schuermann, ... (2017) From gcode to stl: Reconstruct models from 3d printing as a service. IOP Conference Series …, iopscience.iop.org, cited by 1 (0.50 per year)
D Yang, W Luo, Y Huang, S Huang (2019) Facile Synthesis of Monodispersed SiO2@Fe3O4 Core–Shell Colloids for Printing and Three-Dimensional Coating with Noniridescent Structural Colors. ACS Omega, ACS Publications, cited by 1 (1.00 per year)
M Mirkhalaf, A Sunesara, B Ashrafi, ... (2019) Toughness by segmentation: Fabrication, testing and micromechanics of architectured ceramic panels for impact applications. International Journal of …, Elsevier, cited by 5 (5.00 per year)
NA Yaraghi, N Guarín-Zapata, ... (2016) Biocomposites: A Sinusoidally Architected Helicoidal Biocomposite (Adv. Mater. 32/2016). Advanced …, Wiley Online Library, cited by 5 (1.67 per year)
E Garcia-Tunon, S Barg, R Bell, ... (2013) Designing smart particles for the assembly of complex macroscopic structures. Angewandte Chemie …, Wiley Online Library, cited by 24 (4.00 per year)
MC Bélanger, Y Marois (2001) Hemocompatibility, biocompatibility, inflammatory and in vivo studies of primary reference materials low-density polyethylene and polydimethylsiloxane: A review. … Research: An Official Journal of The …, Wiley Online Library, cited by 365 (20.28 per year)
J Cui, JGM Adams, Y Zhu (2018) Controlled bending and folding of a bilayer structure consisting of a thin stiff film and a heat shrinkable polymer sheet. Smart Materials and Structures, iopscience.iop.org, cited by 3 (3.00 per year)
B Wicklein, G Salazar-Alvarez (2013) Functional hybrids based on biogenic nanofibrils and inorganic nanomaterials. Journal of Materials Chemistry A, pubs.rsc.org, cited by 44 (7.33 per year)
S Mao, E Dong, H Jin, M Xu, ... (2016) Locomotion and gait analysis of multi-limb soft robots driven by smart actuators. 2016 IEEE/RSJ …, ieeexplore.ieee.org, cited by 4 (1.33 per year)
Z Chen, J Xu, B Liu, YL Zhang, J Wu (2019) Structural integrity analysis of transmission structure in flapping-wing micro aerial vehicle via 3D printing. Engineering Failure Analysis, Elsevier, cited by 1 (1.00 per year)

J Roleček, L Pejchalová, FJ Martínez-Vázquez, ... (2019) Bioceramic scaffolds fabrication: Indirect 3D printing combined with ice-templating vs. robocasting. Journal of the European …, Elsevier, cited by 1 (1.00 per year)
C Beyer (2014) Strategic implications of current trends in additive manufacturing. Journal of Manufacturing Science and …, … .asmedigitalcollection.asme.org, cited by 96 (19.20 per year)
EB Joyee, Y Pan (2019) A Fully Three-Dimensional Printed Inchworm-Inspired Soft Robot with Magnetic Actuation. Soft robotics, liebertpub.com, cited by 1 (1.00 per year)
WC Eberhardt, BF Wakefield, CT Murphy, ... (2016) Development of an artificial sensor for hydrodynamic detection inspired by a seal's whisker array. Bioinspiration & …, iopscience.iop.org, cited by 6 (2.00 per year)
M Bacher, A Schwen, J Koestel (2015) Three-dimensional printing of macropore networks of an undisturbed soil sample. Vadose Zone Journal, dl.sciencesocieties.org, cited by 11 (2.75 per year)
V Slesarenko, KY Volokh, J Aboudi, ... (2017) Understanding the strength of bioinspired soft composites. International Journal of …, Elsevier, cited by 5 (2.50 per year)
Z Gong, J Cheng, X Chen, W Sun, X Fang, K Hu, ... (2018) A Bio-inspired Soft Robotic Arm: Kinematic Modeling and Hydrodynamic Experiments. Journal of Bionic …, Springer, cited by 1 (1.00 per year)
V Laghi, M Palermo, M Pragliola, ... (2018) Towards 3D-printed steel grid-shells: the main idea and first studies. Proceedings of IASS …, ingentaconnect.com, cited by 1 (1.00 per year)
K Jin, Z Qin, MJ Buehler (2015) Molecular deformation mechanisms of the wood cell wall material. Journal of the mechanical behavior of biomedical …, Elsevier, cited by 33 (8.25 per year)
R Harbird, G Barbareschi, E Makrygianni, ... (2017) Fleas, Caterpillars and Cockroaches: A Summer School in Bio-inspired Robotics. IEEE Global …, discovery.ucl.ac.uk, cited by 1 (0.50 per year)
A Vikram Singh, M Sitti (2016) Targeted drug delivery and imaging using mobile milli/microrobots: A promising future towards theranostic pharmaceutical design. Current pharmaceutical design, ingentaconnect.com, cited by 41 (13.67 per year)
Z Jia, L Wang (2018) 3D Printing of Biomimetic Composites with Improved Fracture Toughness. Available at SSRN 3300049, papers.ssrn.com, cited by 1 (1.00 per year)
S Wang, Y Zhang, G Ji, J Yang, J Wu, L Wei (2015) Fruit classification by wavelet-entropy and feedforward neural network trained by fitness-scaled chaotic ABC and biogeography-based optimization. Entropy, mdpi.com, cited by 110 (27.50 per year)
M Tavakoli, C Viegas (2015) Bio-inspired climbing robots. Biomimetic Technologies, Elsevier, cited by 1 (0.25 per year)
A Davoudinejad, Y Cai, DB Pedersen, X Luo, ... (2019) Fabrication of micro-structured surfaces by additive manufacturing, with simulation of dynamic contact angle. Materials & Design, Elsevier, cited by 4 (4.00 per year)
X Zhang, J Xie, J Chen, Y Okabe, L Pan, M Xu (2017) The beetle elytron plate: a lightweight, high-strength and buffering functional-structural bionic material. Scientific reports, nature.com, cited by 18 (9.00 per year)
J Zhu, M Dexheimer, H Cheng (2017) Reconfigurable systems for multifunctional electronics. npj Flexible Electronics, nature.com, cited by 12 (6.00 per year)
S Araya, E Zolotovsky, F Veliz, J Song, S Reichert, ... (2013) Bioinformed Performative Composite Structures., papers.cumincad.org, cited by 2 (0.33 per year)

D Popov, A Klimchik, I Afanasyev (2017) Design and Stiffness Analysis of 12 DoF Poppy-inspired Humanoid.. ICINCO (2), scitepress.org, cited by 2 (1.00 per year)
T Kaully, K Kaufman-Francis, A Lesman, ... (2009) Vascularization–the conduit to viable engineered tissues. … Engineering Part B …, liebertpub.com, cited by 266 (26.60 per year)
KY Fok, CT Cheng, N Ganganath, ... (2018) An ACO-Based Tool-Path Optimizer for 3-D Printing Applications. IEEE Transactions on …, ieeexplore.ieee.org, cited by 3 (3.00 per year)
M Amirpour, S Bickerton, E Calius, R Das, B Mace (2019) Numerical and experimental study on deformation of 3D-printed polymeric functionally graded plates: 3D-Digital Image Correlation approach. Composite Structures, Elsevier, cited by 1 (1.00 per year)
AR Jensenius, KH Glette, RI Godøy, ME Høvin, ... (2010) fourMs, University of Oslo Lab Report., duo.uio.no, cited by 3 (0.33 per year)
J Yang, EC Baker, HOT Ware, F Zhou, ... (2018) 3d printing of biomedical implants. US Patent App. 15 …, Google Patents, cited by 2 (2.00 per year)
L Moroni, G Poort, F Van Keulen, ... (2006) Dynamic mechanical properties of 3D fiber-deposited PEOT/PBT scaffolds: An experimental and numerical analysis. … Research Part A: An …, Wiley Online Library, cited by 52 (4.00 per year)
S James, R Contractor (2018) Study on Nature-inspired Fractal Design-based Flexible Counter Electrodes for Dye-Sensitized Solar Cells Fabricated using Additive Manufacturing. Scientific reports, nature.com, cited by 1 (1.00 per year)
A Marrella, A Lagazzo, E Dellacasa, C Pasquini, ... (2018) 3D Porous Gelatin/PVA Hydrogel as Meniscus Substitute Using Alginate Micro-Particles as Porogens. Polymers, mdpi.com, cited by 4 (4.00 per year)
AA Sequeira, A Usman, OP Tharakan, ... (2016) Biologically Inspired Robots into a New Dimension-A Review. International Journal of …, researchgate.net, cited by 3 (1.00 per year)
MJ Mirzaali, ME Edens, AH de la Nava, S Janbaz, ... (2018) Length-scale dependency of biomimetic hard-soft composites. Scientific reports, nature.com, cited by 2 (2.00 per year)
A Figliola, M Rossi (2014) Parametric design for technological and" smart" system. Adaptive and optimized skin. 30th international PLEA conference, researchgate.net, cited by 2 (0.40 per year)
D Huerta-Murillo, AI Aguilar-Morales, S Alamri, ... (2017) Fabrication of multi-scale periodic surface structures on Ti-6Al-4V by direct laser writing and direct laser interference patterning for modified wettability …. Optics and Lasers in …, Elsevier, cited by 14 (7.00 per year)
M Jain, K Matsumura (2016) Polyampholyte-and nanosilicate-based soft bionanocomposites with tailorable mechanical and cell adhesion properties. … of Biomedical Materials Research Part A, Wiley Online Library, cited by 8 (2.67 per year)
H Zhang, X Guo, J Wu, D Fang, ... (2018) Soft mechanical metamaterials with unusual swelling behavior and tunable stress-strain curves. Science …, advances.sciencemag.org, cited by 11 (11.00 per year)
U Chandrasekhar, LJ Yang, B Esakki, ... (2017) Rapid prototyping of flapping mechanisms for monoplane and biplane ornithopter configurations. Int. J. Mod. Manuf …, researchgate.net, cited by 3 (1.50 per year)
LSS Dimas (2013) Bio-inspired composites: a de novo approach to the conceptualization, design and synthesis of tough mesoscale structures with simple building blocks., dspace.mit.edu, cited by 1 (0.17 per year)
AK Gaharwar, NA Peppas, ... (2014) Nanocomposite hydrogels for biomedical applications. Biotechnology and …, Wiley Online Library, cited by 467 (93.40 per year)

YC Chen, RZ Lin, H Qi, Y Yang, H Bae, ... (2012) Functional human vascular network generated in photocrosslinkable gelatin methacrylate hydrogels. Advanced functional …, Wiley Online Library, cited by 321 (45.86 per year)
KG Karwa, S Mondal, A Kumar, ... (2016) An open source low-cost alligator-inspired robotic research platform. 2016 Sixth International …, ieeexplore.ieee.org, cited by 1 (0.33 per year)
H Kim, J Jang, J Park, KP Lee, S Lee, DM Lee, ... (2019) Shear-induced alignment of collagen fibrils using 3D cell printing for corneal stroma tissue engineering. …, iopscience.iop.org, cited by 1 (1.00 per year)
S Taccola, F Greco, E Sinibaldi, A Mondini, ... (2015) Toward a new generation of electrically controllable hygromorphic soft actuators. Advanced …, Wiley Online Library, cited by 125 (31.25 per year)
P Kodati, J Hinkle, X Deng (2007) Micro autonomous robotic ostraciiform (MARCO): design and fabrication. Proceedings 2007 IEEE …, ieeexplore.ieee.org, cited by 24 (2.00 per year)
T Kymäläinen (2016) Science fiction prototypes as a method for discussing socio-technical issues within emerging technology research and foresight. Athens Journal of Technology & Engineering …, athensjournals.gr, cited by 4 (1.33 per year)
I Hwang, H Yi, J Choi, HE Jeong (2017) Fabrication of bioinspired dry adhesives by CNC machining and replica molding. International Journal of Precision …, Springer, cited by 4 (2.00 per year)
P Moazzam, H Tavassoli, A Razmjou, ME Warkiani, ... (2018) Mist harvesting using bioinspired polydopamine coating and microfabrication technology. Desalination, Elsevier, cited by 13 (13.00 per year)
F Liravi, E Toyserkani (2018) Additive manufacturing of silicone structures: A review and prospective. Additive Manufacturing, Elsevier, cited by 7 (7.00 per year)
H Shen, J Guo, H Wang, N Zhao, ... (2015) Bioinspired modification of h-BN for high thermal conductive composite films with aligned structure. ACS applied materials & …, ACS Publications, cited by 162 (40.50 per year)
DM Aukes, RJ Wood (2015) PopupCAD: a tool for automated design, fabrication, and analysis of laminate devices. Micro-and Nanotechnology Sensors …, spiedigitallibrary.org, cited by 7 (1.75 per year)
K Wei (2015) Bio-inspired reconfigurable elastomer-liquid lens: design, actuation and optimization., rave.ohiolink.edu, cited by 2 (0.50 per year)
M Strantza, M Hinderdael, D De Baere, ... (2016) Additive manufacturing for novel structural health monitoring systems. Proceedings of the 8th …, ndt.net, cited by 3 (1.00 per year)
CJ Thrasher, JJ Schwartz, ... (2017) Modular elastomer photoresins for digital light processing additive manufacturing. ACS applied materials & …, ACS Publications, cited by 19 (9.50 per year)
TT Oliveira, AC Reis (2019) Fabrication of dental implants by the additive manufacturing method: A systematic review. The Journal of prosthetic dentistry, Elsevier, cited by 4 (4.00 per year)
Z Zhou, S Zhang, Y Cao, B Marelli, X Xia, ... (2018) Engineering the future of silk materials through advanced manufacturing. Advanced …, Wiley Online Library, cited by 9 (9.00 per year)
M Cheng, Q Liu, G Ju, Y Zhang, L Jiang, ... (2014) Bell-Shaped Superhydrophilic–Superhydrophobic–Superhydrophilic Double Transformation on a pH-Responsive Smart Surface. Advanced …, Wiley Online Library, cited by 71 (14.20 per year)
MS Pham, C Liu, I Todd, J Lertthanasarn (2019) Damage-tolerant architected materials inspired by crystal microstructure. Nature, nature.com, cited by 6 (6.00 per year)

L Neely, J Gaiennie, N Noble, ... (2016) Stingray-inspired robot with simply actuated intermediate motion. … and Bioreplication 2016, spiedigitallibrary.org, cited by 1 (0.33 per year)
X Yu, L Pan, J Chen, X Zhang, P Wei (2019) Experimental and numerical study on the energy absorption abilities of trabecular–honeycomb biomimetic structures inspired by beetle elytra. Journal of materials science, Springer, cited by 2 (2.00 per year)
T Naito, M Nakamura, N Kaji, T Kubo, Y Baba, K Otsuka (2016) Three-dimensional fabrication for microfluidics by conventional techniques and equipment used in mass production. Micromachines, mdpi.com, cited by 9 (3.00 per year)
Q Zhang, W Xu, X Wang (2018) Carbon nanocomposites with high photothermal conversion efficiency. Science China Materials, Springer, cited by 4 (4.00 per year)
N West, K Sammut, Y Tang (2018) Material selection and manufacturing of riblets for drag reduction: An updated review. Proceedings of the Institution of …, journals.sagepub.com, cited by 3 (3.00 per year)
R Xie, M Su, Y Zhang, M Li, H Zhu, ... (2018) PISRob: A pneumatic soft robot for locomoting like an inchworm. 2018 IEEE International …, ieeexplore.ieee.org, cited by 4 (4.00 per year)
C Seidel, S Jayaram, L Kunkel, ... (2017) Structural Analysis of Biologically Inspired Small Wind Turbine Blades. … Journal of Mechanical …, ijmme.springeropen.com, cited by 1 (0.50 per year)
G Yang, Y Zhang, J Yang, G Ji, Z Dong, S Wang, ... (2016) Automated classification of brain images using wavelet-energy and biogeography-based optimization. Multimedia Tools and …, Springer, cited by 124 (41.33 per year)
SW Chang, TK Lin, SY Kuo, TH Huang (2017) Integration of high-resolution laser displacement sensors and 3d printing for structural health monitoring. Sensors, mdpi.com, cited by 1 (0.50 per year)
J Ubaid, BL Wardle, S Kumar (2018) Strength and performance enhancement of multilayers by spatial tailoring of adherend compliance and morphology via multimaterial jetting additive …. Scientific reports, nature.com, cited by 2 (2.00 per year)
C Zhang, DA Mcadams, JC Grunlan (2016) Nano/micro-manufacturing of bioinspired materials: A review of methods to mimic natural structures. Advanced Materials, Wiley Online Library, cited by 113 (37.67 per year)
M Kuang, Y Song (2017) Inkjet Printing of Photonic Crystals. Nanomaterials for 2D and 3D Printing, John Wiley & Sons, cited by 1 (0.50 per year)
JFM Molenbroek, M Fleuren, ... (2013) From SML-XL to Mass Customization Case Study: External Ankle Sprain Protection with Exo-L. Proceedings of the 4th …, academia.edu, cited by 2 (0.33 per year)
C Prakash, S Singh, R Singh, S Ramakrishna, ... (2019) Biomanufacturing., Springer, cited by 2 (2.00 per year)
JD Frost, MM Roozbahani, AF Peralta, ... (2017) The evolving role of materials in geotechnical infrastructure systems. Journal of Structural …, Taylor & Francis, cited by 2 (1.00 per year)
J Meng, P Zhang, S Wang (2016) Recent progress of abrasion-resistant materials: learning from nature. Chemical Society Reviews, pubs.rsc.org, cited by 27 (9.00 per year)
Y Yang, Z Pei, Z Li, Y Wei, Y Ji (2016) Making and remaking dynamic 3D structures by shining light on flat liquid crystalline vitrimer films without a mold. Journal of the American Chemical …, ACS Publications, cited by 108 (36.00 per year)

L Lu, J Liu, Y Hu, Y Zhang, ... (2012) Highly stable air working bimorph actuator based on a graphene nanosheet/carbon nanotube hybrid electrode. Advanced …, Wiley Online Library, cited by 79 (11.29 per year)
M Saari, B Xia, PS Krueger, AL Cohen, ... (2016) Additive Manufacturing of Soft Parts from Thermoplastic Elastomers. 12th TPE Topical …, researchgate.net, cited by 2 (0.67 per year)
S Burattini, BW Greenland, W Hayes, ... (2010) A supramolecular polymer based on tweezer-type π − π stacking interactions: molecular design for healability and enhanced toughness. Chemistry of …, ACS Publications, cited by 142 (15.78 per year)
G Deng, F Li, H Yu, F Liu, C Liu, W Sun, ... (2012) Dynamic hydrogels with an environmental adaptive self-healing ability and dual responsive sol–gel transitions. ACS Macro …, ACS Publications, cited by 296 (42.29 per year)
I Duarte, N Peixinho, A Andrade-Campos, ... (2018) Special issue on cellular materials. Science and Technology …, Elsevier, cited by 3 (3.00 per year)
ER Miranda, E Braund (2017) A Method for Growing Bio-memristors from Slime Mold. JoVE (Journal of Visualized Experiments), jove.com, cited by 1 (0.50 per year)
A Davoudinejad, LCD Perez, D Quagliotti, ... (2018) Geometrical and feature of size design effect on direct stereolithography micro additively manufactured components. Procedia Structural …, Elsevier, cited by 3 (3.00 per year)
K Albert, XC Huang, HY Hsu (2017) Bio-templated silica composites for next-generation biomedical applications. Advances in colloid and interface science, Elsevier, cited by 12 (6.00 per year)
HA Bruck, AL Gershon, I Golden, ... (2007) Training mechanical engineering students to utilize biological inspiration during product development. Bioinspiration & …, iopscience.iop.org, cited by 40 (3.33 per year)
G Skeldon, B Lucendo-Villarin, ... (2018) Three-dimensional bioprinting of stem-cell derived tissues for human regenerative medicine. … Transactions of the …, royalsocietypublishing.org, cited by 3 (3.00 per year)
SH Kang, S Shan, WL Noorduin, M Khan, ... (2013) Buckling-induced reversible symmetry breaking and amplification of chirality using supported cellular structures. Advanced …, Wiley Online Library, cited by 59 (9.83 per year)
L Wu, F Karami, A Hamidi, ... (2018) Biorobotic systems design and development using TCP muscles. … Polymer Actuators and …, spiedigitallibrary.org, cited by 2 (2.00 per year)
JA Roll (2012) Bio-inspired flapper with electromagnetic actuation., search.proquest.com, cited by 2 (0.29 per year)
MO Coppens (2005) Scaling-up and-down in a nature-inspired way. Industrial & engineering chemistry research, ACS Publications, cited by 56 (4.00 per year)
M Mastrangeli, A Martinoli, J Brugger (2014) Three-dimensional polymeric microtiles for optically-tracked fluidic self-assembly. Microelectronic engineering, Elsevier, cited by 3 (0.60 per year)
M Silva, A Mateus, D Oliveira, ... (2017) An alternative method to produce metal/plastic hybrid components for orthopedics applications. Proceedings of the …, journals.sagepub.com, cited by 2 (1.00 per year)
R Bogue (2011) Recent developments in adhesive technology: a review. Assembly Automation, emeraldinsight.com, cited by 8 (1.00 per year)
KT Kang, YG Koh, J Son, JS Yeom, JH Park, ... (2017) Biomechanical evaluation of pedicle screw fixation system in spinal adjacent levels using polyetheretherketone, carbon-fiber-reinforced polyetheretherketone …. Composites Part B …, Elsevier, cited by 12 (6.00 per year)

S Ma, B Yu, X Pei, F Zhou (2016) Structural hydrogels. Polymer, Elsevier, cited by 35 (11.67 per year)
G Li, R Urbina, H Zhang, ... (2017) Concept design and simulation of a water proofing modular robot for amphibious locomotion. … Conference on Advanced …, ieeexplore.ieee.org, cited by 2 (1.00 per year)
JM Zhan, YJ Gong, TZ Li (2017) Gliding locomotion of manta rays, killer whales and swordfish near the water surface. Scientific reports, nature.com, cited by 3 (1.50 per year)
TL Mohren, TL Daniel, SL Brunton, ... (2018) Neural-inspired sensors enable sparse, efficient classification of spatiotemporal data. Proceedings of the …, National Acad Sciences, cited by 3 (3.00 per year)
F Connolly, P Polygerinos, CJ Walsh, K Bertoldi (2015) Mechanical programming of soft actuators by varying fiber angle. Soft Robotics, liebertpub.com, cited by 141 (35.25 per year)
TJG Waduge, M Joordens (2017) Fish robotic research platform for swarms. 2017 25th International Conference …, ieeexplore.ieee.org, cited by 3 (1.50 per year)
N Abid, M Mirkhalaf, F Barthelat (2018) Discrete-element modeling of nacre-like materials: Effects of random microstructures on strain localization and mechanical performance. Journal of the Mechanics and Physics of …, Elsevier, cited by 18 (18.00 per year)
P Van Arnum (2014) The future of dosage forms. Pharmaceutical Technology, pharmtech.com, cited by 2 (0.40 per year)
TM Valentin, AK Landauer, LC Morales, EM DuBois, ... (2019) Alginate-graphene oxide hydrogels with enhanced ionic tunability and chemomechanical stability for light-directed 3D printing. Carbon, Elsevier, cited by 2 (2.00 per year)
M Quan, B Yang, J Wang, H Yu, ... (2018) Simultaneous Microscopic Structure Characteristics of Shape-Memory Effects of Thermo-Responsive Poly(vinylidene fluoride-co-hexafluoropropylene) Inverse Opals. ACS applied materials & …, ACS Publications, cited by 9 (9.00 per year)
PDE Baniqued, JR Dungao, ... (2018) Biomimetics in the design of a robotic exoskeleton for upper limb therapy. AIP Conference …, aip.scitation.org, cited by 2 (2.00 per year)
M Lušić, A Barabanov, D Morina, F Feuerstein, ... (2015) Towards zero waste in additive manufacturing: a case study investigating one pressurised rapid tooling mould to ensure resource efficiency. Procedia CIRP, Elsevier, cited by 7 (1.75 per year)
DE Hebel, F Heisel (2017) Cultivated Building Materials. Industrialized Natural Resources for …, researchgate.net, cited by 3 (1.50 per year)
R Rocha, P Lopes, AT de Almeida, ... (2017) Soft-matter sensor for proximity, tactile and pressure detection. 2017 IEEE/RSJ …, ieeexplore.ieee.org, cited by 3 (1.50 per year)
J Li, S Yang, D Li, V Chalivendra (2018) Numerical and experimental studies of additively manufactured polymers for enhanced fracture properties. Engineering Fracture Mechanics, Elsevier, cited by 11 (11.00 per year)
Y Du, H Liu, Q Yang, S Wang, J Wang, J Ma, I Noh, ... (2017) Selective laser sintering scaffold with hierarchical architecture and gradient composition for osteochondral repair in rabbits. Biomaterials, Elsevier, cited by 42 (21.00 per year)
D Cheneler, E Buselli, D Camboni, C Anthony, ... (2014) A bio-hybrid tactile sensor incorporating living artificial skin and an impedance sensing array. Sensors, mdpi.com, cited by 3 (0.60 per year)
Z Jiang, Y Luo, Y Jin (2017) New cable-driven continuun robot with only one actuator. … and Intelligent Systems (CIS) and IEEE …, ieeexplore.ieee.org, cited by 2 (1.00 per year)

S Ni, J Chang, L Chou, W Zhai (2007) Comparison of osteoblast-like cell responses to calcium silicate and tricalcium phosphate ceramics in vitro. Journal of Biomedical …, Wiley Online Library, cited by 166 (13.83 per year)
L Zhang, K Xia, Z Lu, G Li, J Chen, Y Deng, ... (2014) Efficient and facile synthesis of gold nanorods with finely tunable plasmonic peaks from visible to near-IR range. Chemistry of …, ACS Publications, cited by 170 (34.00 per year)
W Hu, W Li, G Alici (2018) 3d printed helical soft pneumatic actuators. 2018 IEEE/ASME International Conference …, ieeexplore.ieee.org, cited by 1 (1.00 per year)
G Kwon, AK Kota, Y Li, A Sohani, JM Mabry, ... (2012) On-demand separation of oil-water mixtures. Advanced …, Wiley Online Library, cited by 315 (45.00 per year)
S Pina, JM Oliveira, RL Reis (2015) Natural-based nanocomposites for bone tissue engineering and regenerative medicine: A review. Advanced Materials, Wiley Online Library, cited by 298 (74.50 per year)
I Rianasari, F Benyettou, SK Sharma, T Blanton, ... (2016) A chemical template for synthesis of molecular sheets of calcium carbonate. Scientific reports, nature.com, cited by 5 (1.67 per year)
ML Shofner, K Lozano, ... (2003) Nanofiber-reinforced polymers prepared by fused deposition modeling. Journal of applied …, Wiley Online Library, cited by 256 (16.00 per year)
X Wang, S Xu, S Zhou, W Xu, M Leary, P Choong, ... (2016) Topological design and additive manufacturing of porous metals for bone scaffolds and orthopaedic implants: A review. Biomaterials, Elsevier, cited by 446 (148.67 per year)
A Tijore, SA Irvine, U Sarig, P Mhaisalkar, ... (2018) Contact guidance for cardiac tissue engineering using 3D bioprinted gelatin patterned hydrogel. …, iopscience.iop.org, cited by 24 (24.00 per year)
R Pfeifer, F Iida, M Lungarella (2014) Cognition from the bottom up: on biological inspiration, body morphology, and soft materials. Trends in cognitive sciences, Elsevier, cited by 63 (12.60 per year)
M Saari, M Galla, B Cox, ... (2015) Additive Manufacturing of Soft and Composite Parts from Thermoplastic Elastomers. … , TX: University of …, sffsymposium.engr.utexas.edu, cited by 7 (1.75 per year)
T Yanagida, R Elara Mohan, T Pathmakumar, ... (2017) Design and implementation of a shape shifting rolling–crawling–wall-climbing robot. Applied Sciences, mdpi.com, cited by 9 (4.50 per year)
D Bell, T Siegmund (2018) 3D-printed polymers exhibit a strength size effect. Additive Manufacturing, Elsevier, cited by 2 (2.00 per year)
Z Qichao, C Yingjie, D Rui, R Ziyu, ... (2014) Bio-inspired flexible robotic caudal fin with three-dimensional locomotion. Proceeding of the …, ieeexplore.ieee.org, cited by 1 (0.20 per year)
张小翠, 陈鹏, 汪健 (2017) 3D 生物打印技术及其在组织工程中的应用. 临床与病理杂志, lcbl.amegroups.com, cited by 1 (0.50 per year)
K Walas (2013) Foot design for a hexapod walking robot. Pomiary Automatyka Robotyka, yadda.icm.edu.pl, cited by 8 (1.33 per year)
连岑, 庄佩, 边卫国, 李涤尘, 靳忠民 (2015) 大尺寸关节支架的 3D 打印及应用. 中国科学: 信息科学, engine.scichina.com, cited by 5 (1.25 per year)

biomimicy AND 3D-printing
Publish or Perish 7.10.2373.7118
Windows (x64) edition, running on Windows 10.0.16299 (x64)
Search terms
Keywords: biomimicy AND 3D-printing
Years: all

Data retrieval
Data source: Google Scholar
Search date: 2019-07-09 08:34:53 +1200
Cache date: 2019-07-09 08:57:42 +1200
Search result: [0] No error

Metrics
Reference date: 2019-07-09 08:57:42 +1200
Publication years: 1953-2019
Citation years: 66 (1953-2019)
Papers: 1000
Citations: 22142
Citations/year: 335.48
Citations/paper: 22.14 (acc1=453, acc2=367, acc5=260, acc10=144, acc20=75)
Authors/paper: 2.90/3.0/1 (mean/median/mode)
Age-weighed citation rate: 5564.68 (sqrt=74.60), 1924.22/author
Hirsch h-index: 72 (a=4.27, m=1.09, 15755 cites=71.2% coverage)
Egghe g-index: 137 (g/h=1.90, 18982 cites=85.7% coverage)
PoP hI,norm: 37
PoP hI,annual: 0.56

Results

E Kennedy, D Fecheyr-Lippens, BK Hsiung, ... (2015) Biomimicry: A path to sustainable innovation. Design Issues, MIT Press, cited by 29 (7.25 per year)
SV Murphy, A Atala (2014) 3D bioprinting of tissues and organs. Nature biotechnology, nature.com, cited by 2505 (501.00 per year)
DM Kalaskar (2017) 3D printing in medicine., books.google.com, cited by 12 (6.00 per year)
Y Liu, E Gill, YY Shery Huang (2017) Microfluidic on-chip biomimicry for 3D cell culture: a fit-for-purpose investigation from the end user standpoint. Future science OA, Future Science, cited by 15 (7.50 per year)
F Pati, J Gantelius, HA Svahn (2016) 3D bioprinting of tissue/organ models. … Chemie International Edition, Wiley Online Library, cited by 87 (29.00 per year)
LS Osborn (2013) Of PhDs, Pirates, and the Public: Three-Dimensional Printing Technology and the Arts. Tex. A&M L. Rev., HeinOnline, cited by 51 (8.50 per year)
GX Gu, I Su, S Sharma, ... (2016) Three-dimensional-printing of bio-inspired composites. Journal of …, … .asmedigitalcollection.asme.org, cited by 67 (22.33 per year)
P Soman, PH Chung, AP Zhang, ... (2013) Digital microfabrication of user-defined 3D microstructures in cell-laden hydrogels. Biotechnology and …, Wiley Online Library, cited by 102 (17.00 per year)
M Guvendiren, J Molde, RMD Soares, ... (2016) Designing biomaterials for 3D printing. … biomaterials science & …, ACS Publications, cited by 170 (56.67 per year)

R Raman, R Bashir (2017) Biomimicry, biofabrication, and biohybrid systems: The emergence and evolution of biological design. Advanced healthcare materials, Wiley Online Library, cited by 15 (7.50 per year)
H Lee, DW Cho (2016) One-step fabrication of an organ-on-a-chip with spatial heterogeneity using a 3D bioprinting technology. Lab on a Chip, pubs.rsc.org, cited by 84 (28.00 per year)
U Jammalamadaka, K Tappa (2018) Recent advances in biomaterials for 3D printing and tissue engineering. Journal of functional biomaterials, mdpi.com, cited by 47 (47.00 per year)
A Taylor, E Unver (2015) 3D Printing our future: Now., eprints.hud.ac.uk, cited by 4 (1.00 per year)
A du Plessis, C Broeckhoven (2018) Looking deep into nature: A review of micro-computed tomography in biomimicry. Acta biomaterialia, Elsevier, cited by 9 (9.00 per year)
J Drager, JL Ramirez-GarciaLuna, A Kumar, ... (2017) Hypoxia Biomimicry to Enhance Monetite Bone Defect Repair. … Engineering Part A, liebertpub.com, cited by 7 (3.50 per year)
ML Terfansky, M Thangavelu (2013) 3D printing of food for space missions. AIAA SPACE 2013 Conference and …, arc.aiaa.org, cited by 7 (1.17 per year)
N Hong, GH Yang, JH Lee, ... (2018) 3D bioprinting and its in vivo applications. Journal of Biomedical …, Wiley Online Library, cited by 37 (37.00 per year)
A Szojka, K Lalh, SHJ Andrews, NM Jomha, M Osswald, ... (2017) Biomimetic 3D printed scaffolds for meniscus tissue engineering. Bioprinting, Elsevier, cited by 14 (7.00 per year)
AL Rutz, KE Hyland, AE Jakus, ... (2015) A multimaterial bioink method for 3D printing tunable, cell-compatible hydrogels. Advanced …, Wiley Online Library, cited by 219 (54.75 per year)
UK Roopavath, DM Kalaskar (2017) Introduction to 3D printing in medicine. 3D Printing in Medicine, Elsevier, cited by 6 (3.00 per year)
D Gu (2016) Materials creation adds new dimensions to 3D printing. Science Bulletin, Springer, cited by 13 (4.33 per year)
M Costantini, J Idaszek, K Szöke, J Jaroszewicz, ... (2016) 3D bioprinting of BM-MSCs-loaded ECM biomimetic hydrogels for in vitro neocartilage formation. …, iopscience.iop.org, cited by 68 (22.67 per year)
HMCM Anver, R Mutlu, G Alici (2017) 3D printing of a thin-wall soft and monolithic gripper using fused filament fabrication. 2017 IEEE International …, ieeexplore.ieee.org, cited by 7 (3.50 per year)
D Sundaramurthi, S Rauf, C Hauser (2016) 3D bioprinting technology for regenerative medicine applications. International Journal of …, ijb.whioce.com, cited by 18 (6.00 per year)
GX Gu, F Libonati, SD Wettermark, ... (2017) Printing nature: Unraveling the role of nacre's mineral bridges. Journal of the mechanical …, Elsevier, cited by 45 (22.50 per year)
AS Munoz-Abraham, MI Rodriguez-Davalos, ... (2016) 3D printing of organs for transplantation: where are we and where are we heading?. Current Transplantation …, Springer, cited by 13 (4.33 per year)
S Vijayavenkataraman, S Zhang, WF Lu, ... (2018) Electrohydrodynamic-jetting (EHD-jet) 3D-printed functionally graded scaffolds for tissue engineering applications. Journal of Materials …, cambridge.org, cited by 14 (14.00 per year)
BN Johnson, KZ Lancaster, IB Hogue, F Meng, ... (2016) 3D printed nervous system on a chip. Lab on a Chip, pubs.rsc.org, cited by 64 (21.33 per year)

B Prayudhi (2016) 3F3D: Form Follows Force with 3D printing., repository.tudelft.nl, cited by 2 (0.67 per year)
E Unver, A Taylor (2015) 3D Additive Manufacturing Symposium & Workshop., eprints.hud.ac.uk, cited by 4 (1.00 per year)
ZM Jessop, A Al-Sabah, MD Gardiner, ... (2017) 3D bioprinting for reconstructive surgery: principles, applications and challenges. Journal of Plastic …, Elsevier, cited by 24 (12.00 per year)
H Cui, W Zhu, B Holmes, LG Zhang (2016) Biologically inspired smart release system based on 3D bioprinted perfused scaffold for vascularized tissue regeneration. Advanced science, Wiley Online Library, cited by 42 (14.00 per year)
S Rhee, JL Puetzer, BN Mason, ... (2016) 3D bioprinting of spatially heterogeneous collagen constructs for cartilage tissue engineering. ACS Biomaterials …, ACS Publications, cited by 76 (25.33 per year)
D Singh, D Singh, S Han (2016) 3D printing of scaffold for cells delivery: Advances in skin tissue engineering. Polymers, mdpi.com, cited by 43 (14.33 per year)
R Mutlu, C Tawk, G Alici, ... (2017) A 3D printed monolithic soft gripper with adjustable stiffness. IECON 2017-43rd Annual …, ieeexplore.ieee.org, cited by 4 (2.00 per year)
A Tirella, G Vozzi, A Ahluwalia (2008) Biomimicry of pam microfabricated hydrogel scaffold. NIP & Digital Fabrication …, ingentaconnect.com, cited by 5 (0.45 per year)
RP Rimington, AJ Capel, SDR Christie, MP Lewis (2017) Biocompatible 3D printed polymers via fused deposition modelling direct C 2 C 12 cellular phenotype in vitro. Lab on a Chip, pubs.rsc.org, cited by 15 (7.50 per year)
L Henderson, T Glaser, F Kuester (2017) Towards bio-inspired structural design of a 3D printable, ballistically deployable, multi-rotor UAV. 2017 IEEE Aerospace …, ieeexplore.ieee.org, cited by 7 (3.50 per year)
IC De Pauw, E Karana, P Kandachar, ... (2014) Comparing Biomimicry and Cradle to Cradle with Ecodesign: a case study of student design projects. Journal of Cleaner …, Elsevier, cited by 33 (6.60 per year)
S Allameh (2015) On the Development of a 3D Printer for Combinatorial Structural Composite Research. ASME 2015 International …, … .asmedigitalcollection.asme.org, cited by 5 (1.25 per year)
Z Liu, Q Lin, Y Sun, T Liu, C Bao, F Li, ... (2014) Spatiotemporally controllable and cytocompatible approach builds 3D cell culture matrix by photo-uncaged-thiol Michael addition reaction. Advanced …, Wiley Online Library, cited by 56 (11.20 per year)
A Fischer, S Rommel, A Verl (2015) 3D Printed Objects and Components Enabling Next Generation of True Soft Robotics. Soft Robotics, Springer, cited by 5 (1.25 per year)
E Axpe, M Oyen (2016) Applications of alginate-based bioinks in 3D bioprinting. International journal of molecular sciences, mdpi.com, cited by 78 (26.00 per year)
A De Mel, B Ramesh, DJ Scurr, ... (2014) Fumed Silica Nanoparticle Mediated Biomimicry for Optimal Cell–M aterial Interactions for Artificial Organ Development. Macromolecular …, Wiley Online Library, cited by 10 (2.00 per year)
P Sander (2015) Additive Layer Manufacturing. Airbus technical magazine-FAST, sfa-am.ch, cited by 7 (1.75 per year)
IT Ozbolat (2016) 3D Bioprinting: fundamentals, principles and applications., books.google.com, cited by 24 (8.00 per year)
KJ Tsai, S Dixon, LR Hale, A Darbyshire, ... (2017) Biomimetic heterogenous elastic tissue development. NPJ Regenerative …, nature.com, cited by 10 (5.00 per year)

S Perkins (2015) Building on evolution: from insects to drones [biomimicry]. Engineering & Technology, IET, cited by 1 (0.25 per year)
MJ Thompson, J Burnett, DM Ixtabalan, D Tran, ... (2015) Experimental design of a flapping wing micro air vehicle through biomimicry of bumblebees. AIAA Infotech …, arc.aiaa.org, cited by 1 (0.25 per year)
HJ So, J Lee, B Kye (2017) An Exploratory Study about the Activity Framework for 3D Printing in Education and Implementation. 정보교육학회논문지, koreascience.or.kr, cited by 3 (1.50 per year)
MJ Lerman, J Lembong, G Gillen, ... (2018) 3D printing in cell culture systems and medical applications. Applied Physics …, aip.scitation.org, cited by 1 (1.00 per year)
B Feigl, D Hutmacher (2013) Eyes on 3D-Current 3D Biomimetic Disease Concept Models and Potential Applications in Age-Related Macular Degeneration. Advanced healthcare materials, Wiley Online Library, cited by 8 (1.33 per year)
S Vijayavenkataraman, J Fuh, W Lu (2017) 3D printing and 3D bioprinting in pediatrics. Bioengineering, mdpi.com, cited by 13 (6.50 per year)
JT Eom (2016) Development and Application of a Biomimicry focused Convergence Teaching Program Using 3D Printing (Master's thesis). Korea National University of Education, Chongju …, cited by 2 (0.67 per year)
X Ma, X Qu, W Zhu, YS Li, S Yuan, ... (2016) Deterministically patterned biomimetic human iPSC-derived hepatic model via rapid 3D bioprinting. Proceedings of the …, National Acad Sciences, cited by 206 (68.67 per year)
E Benrashid, CC McCoy, LM Youngwirth, J Kim, ... (2016) Tissue engineered vascular grafts: Origins, development, and current strategies for clinical application. Methods, Elsevier, cited by 42 (14.00 per year)
D Thomas, D Singh (2017) 3D-printing for engineering the next generation of artificial trabecular bone structures. International journal of surgery (London …, researchgate.net, cited by 1 (0.50 per year)
BN Johnson, MC McAlpine (2016) From print to patient: 3D-printed personalized nerve regeneration. Biochemist, biochemist.org, cited by 2 (0.67 per year)
A Kumar, KC Nune, RDK Misra (2016) Biological functionality of extracellular matrix-ornamented three-dimensional printed hydroxyapatite scaffolds. Journal of Biomedical …, Wiley Online Library, cited by 26 (8.67 per year)
Z Zhang, B Wang, D Hui, J Qiu, S Wang (2017) 3D bioprinting of soft materials-based regenerative vascular structures and tissues. Composites Part B: Engineering, Elsevier, cited by 16 (8.00 per year)
A Atala, K Richardson (2017) The quest to 3D print body parts., biochemist.org, cited by 1 (0.50 per year)
X Li, L Liu, X Zhang, T Xu (2018) Research and development of 3D printed vasculature constructs. Biofabrication, iopscience.iop.org, cited by 4 (4.00 per year)
BS Kim, JS Lee, G Gao, DW Cho (2017) Direct 3D cell-printing of human skin with functional transwell system. Biofabrication, iopscience.iop.org, cited by 47 (23.50 per year)
S Dunham, B Mosadegh, EA Romito, ... (2018) Applications of 3D Printing. 3D Printing Applications in …, Elsevier, cited by 2 (2.00 per year)
H Kim, J Jang, J Park, KP Lee, S Lee, DM Lee, ... (2019) Shear-induced alignment of collagen fibrils using 3D cell printing for corneal stroma tissue engineering. …, iopscience.iop.org, cited by 1 (1.00 per year)

AD Lantada, S Hengsbach, K Bade (2017) Lotus-on-chip: computer-aided design and 3D direct laser writing of bioinspired surfaces for controlling the wettability of materials and devices. Bioinspiration & biomimetics, iopscience.iop.org, cited by 5 (2.50 per year)
B Maiti, D Díaz Díaz (2018) 3D printed polymeric hydrogels for nerve regeneration. Polymers, mdpi.com, cited by 2 (2.00 per year)
J Knowles, T Kelley, J Holland (2018) Increasing teacher awareness of STEM careers. Journal of STEM Education, learntechlib.org, cited by 4 (4.00 per year)
J Hildebrandt, A Bezama, ... (2017) Cascade use indicators for selected biopolymers: are we aiming for the right solutions in the design for recycling of bio-based polymers?. Waste Management & …, journals.sagepub.com, cited by 12 (6.00 per year)
CG Helguero, JL Amaya, DE Komatsu, S Pentyala, ... (2017) Trabecular scaffolds' mechanical properties of bone reconstruction using biomimetic implants. Procedia Cirp, Elsevier, cited by 3 (1.50 per year)
ES Bishop, S Mostafa, M Pakvasa, HH Luu, MJ Lee, ... (2017) 3-D bioprinting technologies in tissue engineering and regenerative medicine: Current and future trends. Genes & diseases, Elsevier, cited by 47 (23.50 per year)
D Vyas, D Udyawar (2019) A review on current state of art of bioprinting. 3D Printing and Additive Manufacturing Technologies, Springer, cited by 2 (2.00 per year)
M Sauerwein, CA Bakker, ... (2017) îï î î î î. PLATE: Product Lifetimes …, books.google.com, cited by 3 (1.50 per year)
G Montalbano, S Fiorilli, A Caneschi, ... (2018) Type I Collagen and Strontium-Containing Mesoporous Glass Particles as Hybrid Material for 3D Printing of Bone-Like Materials. Materials, mdpi.com, cited by 4 (4.00 per year)
CE Corcione, F Gervaso, F Scalera, ... (2019) Highly loaded hydroxyapatite microsphere/PLA porous scaffolds obtained by fused deposition modelling. Ceramics …, Elsevier, cited by 6 (6.00 per year)
C Kengla, A Kidiyoor, SV Murphy (2017) Bioprinting Complex 3D Tissue and Organs. Kidney Transplantation, Bioengineering …, Elsevier, cited by 1 (0.50 per year)
C Wise, M Pawlyn, M Braungart (2013) Eco-engineering: Living in a materials world. Nature, nature.com, cited by 8 (1.33 per year)
GX Gu, CT Chen, DJ Richmond, MJ Buehler (2018) Bioinspired hierarchical composite design using machine learning: simulation, additive manufacturing, and experiment. Materials Horizons, pubs.rsc.org, cited by 17 (17.00 per year)
C Lucas, M Spiegel (2015) 3D printing skin grafts for facial reconstruction., Academic Press, cited by 4 (1.00 per year)
TH Jovic, ZM Jessop, A Al-Sabah, ... (2018) The clinical need for 3D printed tissue in reconstructive surgery. 3D Bioprinting for …, Elsevier, cited by 1 (1.00 per year)
J García-Ruíz, A Diaz Lantada (2018) 3D Printed Structures Filled with Carbon Fibers and Functionalized with Mesenchymal Stem Cell Conditioned Media as In Vitro Cell Niches for Promoting …. Materials, mdpi.com, cited by 2 (2.00 per year)
CR Goulding, A Moelenhoff, CG Goulding (2011) The New Shape of R&D Tax Credits. Corp. Bus. Tax'n Monthly, HeinOnline, cited by 1 (0.13 per year)
BK Hsiung, RH Siddique, L Jiang, Y Liu, ... (2017) Tarantula-Inspired Noniridescent Photonics with Long-Range Order. Advanced Optical …, Wiley Online Library, cited by 19 (9.50 per year)
JE Trachtenberg, M Santoro, C Williams III, ... (2017) Effects of shear stress gradients on ewing sarcoma cells using 3D printed scaffolds and flow perfusion. ACS Biomaterials …, ACS Publications, cited by 10 (5.00 per year)

Y Yu, KK Moncal, J Li, W Peng, I Rivero, JA Martin, ... (2016) Three-dimensional bioprinting using self-assembling scalable scaffold-free “tissue strands” as a new bioink. Scientific reports, nature.com, cited by 82 (27.33 per year)
S Azukizawa, H Shinoda, K Tokumaru, ... (2018) 3D printing system of magnetic anisotropy for artificial cilia. Journal of Photopolymer …, jstage.jst.go.jp, cited by 3 (3.00 per year)
S Adepu, N Dhiman, A Laha, CS Sharma, ... (2017) Three-dimensional bioprinting for bone tissue regeneration. Current Opinion in …, Elsevier, cited by 19 (9.50 per year)
JFM Molenbroek, M Fleuren, ... (2013) From SML-XL to Mass Customization Case Study: External Ankle Sprain Protection with Exo-L. Proceedings of the 4th …, academia.edu, cited by 2 (0.33 per year)
VM Čolić-Damjanovic, ... (2016) Potentials of fablabs for biomimetic architectural research. 2016 International …, ieeexplore.ieee.org, cited by 1 (0.33 per year)
H Kim, S Kim (2018) Analysis of the Type of 3D Printing Development Linked with the Textile Structure Principle. Journal of Fashion Business, koreascience.or.kr, cited by 1 (1.00 per year)
A Fu (2017) Copyright Serverability: The Hurdle between 3D-Printing and Mass Crowdsourced Innovation. Duke L. & Tech. Rev., HeinOnline, cited by 1 (0.50 per year)
S Vijayavenkataraman, S Thaharah, S Zhang, WF Lu, ... (2019) Electrohydrodynamic jet 3D-printed PCL/PAA conductive scaffolds with tunable biodegradability as nerve guide conduits (NGCs) for peripheral nerve injury …. Materials & Design, Elsevier, cited by 1 (1.00 per year)
V Sorensen, N Thummanapalli (2017) Digital amulet: smart necklace. Proceedings of the 2017 ACM …, dl.acm.org, cited by 1 (0.50 per year)
M Sauerwein, E Doubrovski, R Balkenende, ... (2019) Exploring the potential of additive manufacturing for product design in a circular economy. Journal of Cleaner …, Elsevier, cited by 2 (2.00 per year)
E Grain, E Unver (2016) 3D Printed Fashion: A Dual Approach., eprints.hud.ac.uk, cited by 1 (0.33 per year)
AK Goel, DA McAdams, RB Stone (2015) Biologically inspired design., Springer, cited by 78 (19.50 per year)
M Reilly (2013) The future will build itself. New Scientist, Elsevier, cited by 1 (0.17 per year)
WW Wits, D Jafari, Y Jeggels, ... (2018) Freeform-optimized shapes for natural-convection cooling. … Investigations of ICs …, ieeexplore.ieee.org, cited by 1 (1.00 per year)
G Mazza, W Al-Akkad, K Rombouts, ... (2018) Liver tissue engineering: From implantable tissue to whole organ engineering. Hepatology …, Wiley Online Library, cited by 8 (8.00 per year)
K Meggs (2015) 3D Printing of Microstructures for Cellular Investigation and Biomimicry., escholarship.org
S Vamvakidis (2015) Gradient Transparency: Marine Animals as a Source of Inspiration Exploring Material Bio-Mimicry through the Latest 3D Printing Technology in Architectural …., pearl.plymouth.ac.uk
MJ Lerman (2019) 3D-PRINTED POLYSTYRENE FOR CELL CULTURE., 129.2.19.62
D Medeiros 3D Printing: Organs & Tissues. pdfs.semanticscholar.org
CB Ustundag (2018) 3D printing for tissue engineering applications. Politeknik Dergisi, dergipark.org.tr
ME Wade (2016) Engineering of Elastomeric Biomaterials and Biomimicry of Extracellular Matrix for Soft Tissue Regeneration., rave.ohiolink.edu

G Shi, Y Wang, S Derakhshanfar, K Xu, W Zhong, ... (2019) Biomimicry of oil infused layer on 3D printed poly (dimethylsiloxane): Non-fouling, antibacterial and promoting infected wound healing. Materials Science and …, Elsevier
LB June Revolutionizing Business Through 3D Printing. emerypharma.com
YS Rubtsova, ASP ONAT THE 3D BIOPRINTING AS NOVEL REALM IN IT MEDICINE. Б Б К 32 І 74, onat.edu.ua
A du Plessis (2019) MicroCT for metal 3D printing. Quest, journals.co.za
NA Sears (2017) Emulsion Inks: A New Class of Materials for 3D Printing Porous Tissue Engineered Grafts., oaktrust.library.tamu.edu
RP Harrison … , Department for Nanostructured Materials, Jozef Stefan Institute, Slovenia Additional hardware: Roman Bevc 3D printing: Stephan Doepner, Cirkulacija 2. ultramono.org
D Evenski (2017) HeadStrong: Concussion Reduction Using Biomimicry., scholarworks.rit.edu
B Toprakhisar (2016) Development of tendon derived natural extracellular matrix hydrogels for 3D bioprinting applications., research.sabanciuniv.edu
RP Siegel (2016) Can 3-D Printing Go Green?. Mechanical Engineering Magazine …, … .asmedigitalcollection.asme.org
JU Lee, WJ Kim, YW Koo, YE Choe, ... (2018) … Hybrid Scaffold Consisting of Compact and Spongy Bone Structure Using Gelatin/α-Tricalcium Phosphate via 3D-Printing and Electrohydrodynamic Jetting Processes. Nanoscience and …, ingentaconnect.com
C Hu, Y Chen, MJA Tan, K Ren, H Wu (2019) Microfluidic Technologies for Vasculature Biomimicry. Analyst, pubs.rsc.org
SH Park, UH Koh, DY Yang, NK Lee, ... (2015) Development of 3D printed biomimetic scaffold for tissue engineering. 2015 15th International …, ieeexplore.ieee.org
AK Mishra, A Degl'Innocenti, ... (2018) Three-dimensional reconstruction of root shape in the moth orchid Phalaenopsi s sp.: a biomimicry methodology for robotic applications. BMC research …, bmcresnotes.biomedcentral.com
DU Weerasinghe, S Perera, ... (2019) Application of biomimicry for sustainable functionalization of textiles: review of current status and prospectus. Textile Research …, journals.sagepub.com
RP Harrison … Podpečan, Department of Knowledge Technologies, Institute Jozef Stefan Custom-made electronics for Arbora Protectors objects: Gregor Krpič 3D printing …. ultramono.org
J Joseph, K Deshmukh, T Tung, ... (2019) 3D Printing Technology of Polymer Composites and Hydrogels for Artificial Skin Tissue Implementations. Polymer …, Springer
S Chameettachal, F Pati (2017) 3D printed in vitro disease models. 3D Printing in Medicine, Elsevier
B Hallgrimsson, A Dewar, J Laliberte TAKING 3D PRINTING FROM ART TO SCIENCE: DESIGNING FOR A TRANSFORMATIVE TECHNOLOGY. idsa.org
P Petrov, B Kim, OR Hernandez, M Mulvihill (2013) Biomimicry, 3D printing and the future of the green manufacturing., Sustainable Products & Solutions …
BB Gobbato (2019) Setting Up Your Own Home 3D Printing “Plant”. 3D Printing in Orthopaedic Surgery, Elsevier
I Noh (2018) Biomimetic Medical Materials: From Nanotechnology to 3D Bioprinting., books.google.com
RH Crawford, A Stephan Biomimicry versus machinery: the notion of functionality in design. researchgate.net

MP BALLESTEROS, A LALATSA (2019) Market Demands in 3D Printing Pharmaceuticals Products. 3D Printing Technology in …, books.google.com
MO Coppens, A Perera (2018) Re-designing materials for biomedical applications: from biomimicry to nature-inspired chemical engineering. … Transactions of the Royal Society A …, discovery.ucl.ac.uk
H Ashraf, B Meer, R Naz, A Saeed, ... (2018) 3D-Bioprinting: A stepping stone towards enhanced medical approaches. … in Life Sciences, submission.als-journal.com
L Reclaru, D Grecu 3D Printing Yesterday, Today, and Tomorrow in orthopedic field. researchgate.net
AWAYTOLL LONGER 3D ORGAN PRINTING. OVERFISHING+ MEGAFIRES
SH Kwon, YJ Lee, YJ Kwon (2018) An active learning approach to investigate the ecosystem of tide flats using 3D modeling and printing. Journal of Biological Education, Taylor & Francis
K Brand (2017) A novice's guide to 3D printing: Making the process less magical and more understandable. Additive Manufacturing Handbook, taylorfrancis.com
AS Perera, MO Coppens (2018) Re-designing materials for biomedical applications: from biomimicry to nature-inspired chemical engineering. Philosophical Transactions of …, royalsocietypublishing.org
K Hsu (2015) A Laser-Based Local Preheating Process to Enhance the as Built Strength Isotropy of the Fused Filament Fabrication (FFF) Part., asu.pure.elsevier.com
TG Papaioannou, D Manolesou, E Dimakakos, ... (2019) 3D Bioprinting Methods and Techniques: Applications on Artificial Blood Vessel Fabrication. Acta Cardiologica …, tsoc.org.tw
LT Dean (2016) The Factory of the Future, Group Exhibition., dora.dmu.ac.uk
X Tan, YJ Tan (2019) 3D Printing of Metallic Cellular Scaffolds for Bone Implants. 3D and 4D Printing in …, Wiley-VCH Verlag GmbH & Co …
G Aranda-Mena, E Baez, M Arashpoor (2018) 3-D Printing Biomimetic Façades with Recycled Glass. 3DcP 2018, researchgate.net
C Clerc (2011) Biomimicry: Towards a Sustain-Able Design. Biomimicry in Higher Education Webinar, researchgate.net
RV Raggi, IP Munhoz, CL Noriega Biomimicry Applied in Engineering Education: a Case Study in PUC-SP. researchgate.net
SY Hann, H Cui, T Esworthy, S Miao, X Zhou, ... (2019) Recent advances in 3D printing: vascular network for tissue and organ regeneration. Translational …, Elsevier
G Varinlioğlu, B Pasin Integrating Biomimicry and Geoinformatics: A Designerly Approach to Underwater Colonization. International Journal of Environment and …, dergipark.org.tr
D Aguiar, A Albuquerque, B Li 3D INKJETTING DROPLET FORMATION OF BACTERIAL CELLULOSIC EXOPOLYSACCHARIDE GEL. sffsymposium.engr.utexas.edu
K Boura 3-D Bioprinting. pdfs.semanticscholar.org
JY Tey, D Teh, WH Yeo, KPY Shak, ... (2019) Development of 3D printer for functionally graded material using fused deposition modelling method. … Series: Earth and …, iopscience.iop.org
P Damodaram, R Mitra (2018) Bio-Mimetic Design with 3D Printable Composites., openprairie.sdstate.edu
I Paredes Almaraz (2019) New dynamic pallet for Volvo Trucks based in biomimicry: New support modular and adaptable to different geometries, based in the structures of the radiolarian …., diva-portal.org

S Vamvakidis (2015) Gradient Transparency: Marine Animals as a Source of Inspiration., pdfs.semanticscholar.org
R Albuquerque, G Arbelaez, F Cruz, ... (2018) Modelling, printing and validation of dental dry models for implantology skills training. … and Innovation (ICE …, ieeexplore.ieee.org
S Crawford Bio-Appropriation.
AP Haring, EG Thompson, Y Tong, S Laheri, ... (2019) Process-and bio-inspired hydrogels for 3D bioprinting of soft free-standing neural and glial tissues. …, iopscience.iop.org
P Carter, N Bhattarai (2013) Engineered Biomimicry: Chapter 7. Bioscaffolds: Fabrication and Performance., books.google.com
RP Rimington (2018) Design and additive manufacture of microphysiological perfusion systems for pharmaceutical screening of tissue engineered skeletal muscle., ethos.bl.uk
O Kırdök, A Tokuç Solar regulation in architecture with 3D fibrous production methods. researchgate.net
P MARTINS-MOURÃO, P JANUÁRIO Design with Nature: The connection between Critical Regionalism and Biomimicry in a Moroccan village. researchgate.net
N Aghababaie (2014) The Future: Design and my part in it., researchgate.net
GD Mahumane, P Kumar, LC du Toit, ... (2018) 3D scaffolds for brain tissue regeneration: architectural challenges. Biomaterials …, pubs.rsc.org
T Grose (2016) PLAYING THE BLUES. ASEE Prism, search.proquest.com
RP Harrison Installation (generative digital environment) Project by Tanja Vujinović Production: Ultramono and SciArt Lab IJS, 2019 3D objects, generative modelling …. ultramono.org
YM Benyoucef, A Razin (2018) BIOMIMICRY ARCHITECTURE, FROM THE INSPIRATION BY NATURE TO THE INNOVATION OF THE SAHARAN ARCHITECTURE. Architecture and Engineering, aej.spbgasu.ru
E Skorpinski AN ETHICAL DILEMMA IN BIOPRINTED ORGANS.
K Goffin, A Kates (2015) The Debate. Engineering & Technology, IET
HY Shim, HE Lee (2019) Multi-Converging Educational Program for Design with the usage of 3D., aodr.org
SH Teoh, BT Goh, J Lim (2019) 3D printed polycaprolactone scaffolds for bone regeneration-success and future perspective.. Tissue Engineering, liebertpub.com
C Bakker, R Mugge (2017) Sauerwein M., Bakker CA and Balkenende AR. Delft University of Technology, researchgate.net
BY Mohammed, R Andrey (2018) BIOMIMICRY ARCHITECTURE, FROM THE INSPIRATION BY NATURE TO THE INNOVATION OF THE SAHARAN ARCHITECTURE. Architecture and Engineering, cyberleninka.ru
S Bairagi, MK Vinchurkar, R Kushwah, N Balekar Journal Of Harmonized Research (JOHR). researchgate.net
W Laufs (2017) Expansion of Spacial Realities-Digital Fabrication Built in USA. Proceedings of IASS Annual Symposia, ingentaconnect.com
ME Staymates, WA MacCrehan, JL Staymates, ... (2016) Biomimetic Sniffing with an Artificial Dog's Nose Leads to Improvements in Vapor Sampling and Detection., nist.gov
B Zhang, L Gao, L Ma, Y Luo, H Yang, Z Cui (2019) 3D Bioprinting: A Novel Avenue for Manufacturing Tissues and Organs. Engineering, Elsevier
B Bekaert (2018) 3D BIOPRINTING OF VASCULARIZED MICRO-AGGREGATES., lib.ugent.be
R Koontz (2019) Bio-Inspired Transportation and Communication., books.google.com

OS Fenton, M Paolini, JL Andresen, ... (2019) Outlooks on Three-Dimensional Printing for Ocular Biomaterials Research. Journal of Ocular …, liebertpub.com
HY Shim, HE Lee (2019) Multi-Converging Educational Program for Design with the usage of 3D Printer: Targeted for Middle School Students. Archives of Design Research, aodr.org
TTH Brown Speedy Shark Skin. ursa.browntth.com
N Alharbi, SF Khan, O Bretcanu, ... (2015) Processing of Apatite-Wollastonite (AW) glass-ceramic for Three Dimensional Printing (3DP). Applied Mechanics and …, Trans Tech Publ
M JAWAD (2019) Manufactured by Nature: Growing Generatively Designed Products., scholarscompass.vcu.edu
LW Henderson (2017) Adaptive Design & Optimization of 3D Printable, Shape-Changing, Ballistically Deployable Drone Platforms., cloudfront.escholarship.org
D Fecheyr Lippens (2017) Implementing Biomimicry Thinking from fundamental R&D to creating nature-aligned organizations., rave.ohiolink.edu
هبـة سـامي منصـور (2018) التصنيع بالأضافة ودورها في التصميم الداخلي المستدام المحاكي للطبيعة. Journal of Researches in Science and …, rssa.alexu.edu.eg
R GODZISZ, K VODON, N WILLIAMS, N HUDSON RESEARCH REPORT DRAFT. nathanielh.webfactional.com
N Marisol Leon-Lugo, ... (2018) Characterization of bioceramic composites in titanium sponges by interpenetration by assisted pulsed current. MATERIA-RIO …, UNIV FED RIO DE JANEIRO, LAB …
R Ng, S Patel (2013) Trajectories of Performative Materials., papers.cumincad.org
SM Allameh, R Miller, ... (2018) Mechanical Properties of 3D Printed Biomimicked Composites. ASME 2018 …, … .asmedigitalcollection.asme.org
K Robson Brown, D Bacheva, ... (2019) The structural efficiency of the sea sponge Euplectella aspergillum skeleton: bio-inspiration for 3D printed architectures. Journal of the Royal …, royalsocietypublishing.org
PC into Learning turning students into inquisitive creators. search.proquest.com
M Flisykowska (2018) Application of Incremental Technologies in Considerations of Transhumanist Aesthetics–Project" Who nose". Journal of Science and Technology of the Arts, artes.ucp.pt
G Vamvakidis (2017) Fabricating Gradient Transparency: A Bio-Inspired Digital Design Model and Prototyping Methods. 3D Printing and Additive Manufacturing, liebertpub.com
J Schutte, X Yuan, S Dirven, ... (2017) The opportunity of electrospinning as a form of additive manufacturing in biotechnology. 2017 24th International …, ieeexplore.ieee.org
A Ghazlan (2017) A bio-inspired composite system for protecting critical structural components from extreme loads., minerva-access.unimelb.edu.au
M Aydin (2015) Additive Manufacturing: Is It a New Era for Furniture Production?. Journal of Mechanics Engineering and …, pdfs.semanticscholar.org
J Parthasarathy (2015) 15 Medical Applications of Additive Manufacturing. Additive Manufacturing: Innovations, Advances …, books.google.com, cited by 2 (0.50 per year)
A Roy, V Saxena, LM Pandey (2018) 3D printing for cardiovascular tissue engineering: a review. Materials technology, Taylor & Francis, cited by 5 (5.00 per year)
J Ok, D Scudder (2016) Emotive qualities of parametrically designed and 3D printed surfaces. Systems&design: beyond processes and thinking, riunet.upv.es
E Logan From Maker, to Manufacturer, to Market, to Municipal Waste. sites.temple.edu
M Moreno Beguerisse, O Ponte, F Charnley (2017) Taxonomy of design strategies for a circular design tool., dspace.lib.cranfield.ac.uk

V Serpooshan, M Mahmoudi, DA Hu, ... (2017) Bioengineering cardiac constructs using 3D printing. Journal of 3D printing …, Future Medicine, cited by 11 (5.50 per year)
GJM Krijnen, RGP Sanders (2017) Recent developments in bio-inspired sensors fabricated by additive manufacturing technologies. Advances in science and technology, Trans Tech Publ, cited by 1 (0.50 per year)
J Ammar (2016) The Medical Mile Gearing toward 3D-Bespoke Healthcare: A Comparison of United States and European Union Patent Regimes. Gonz. L. Rev., HeinOnline
F Huijben (2016) Vacuumatic formwork: a novel granular manufacturing technique for producing topology-optimised structures in concrete. Granular Matter, Springer, cited by 8 (2.67 per year)
J Tian, BC Bryksa, RY Yada (2016) Feeding the world into the future–food and nutrition security: the role of food science and technology. Frontiers in Life Science, Taylor & Francis, cited by 14 (4.67 per year)
GX Gu, M Takaffoli, MJ Buehler (2017) Hierarchically enhanced impact resistance of bioinspired composites. Advanced Materials, Wiley Online Library, cited by 55 (27.50 per year)
EL Gill, X Li, MA Birch, YYS Huang (2018) Multi-length scale bioprinting towards simulating microenvironmental cues. Bio-design and manufacturing, Springer, cited by 4 (4.00 per year)
L Rademeyer A skincare servicescape for Margaret Roberts: biomimicry and biophilia as a model and mentor for design. repository.up.ac.za
S Kyle, ZM Jessop, A Al-Sabah, ... (2017) 'Printability'of Candidate Biomaterials for Extrusion Based 3D Printing: State-of-the-Art. Advanced healthcare …, Wiley Online Library, cited by 50 (25.00 per year)
J Hoarau-Véchot, A Rafii, C Touboul, ... (2018) Halfway between 2D and animal models: are 3D cultures the ideal tool to study cancer-microenvironment interactions?. International journal of …, mdpi.com, cited by 25 (25.00 per year)
RM Costa, S Rauf, CAE Hauser (2017) Towards biologically relevant synthetic designer matrices in 3D bioprinting for tissue engineering and regenerative medicine. Current Opinion in Biomedical …, Elsevier, cited by 5 (2.50 per year)
S Summit (2014) Additive manufacturing of a prosthetic limb. Rapid Prototyping of Biomaterials, Elsevier, cited by 2 (0.40 per year)
P Gruber (2018) What is the Architect doing in the Jungle?., esf.edu
K Kattaiya, V Veeraiyan, K Kaliappa, ... (2019) A Strategic Approach Towards Form and Design Development of Bikers Shoes for Indian Market. Research into Design for a …, Springer
LA van der Elst, S Quintanar-Guzmán, ... (2017) Design of an electromechanical prosthetic finger using shape memory alloy wires. … on Robotics and …, ieeexplore.ieee.org, cited by 2 (1.00 per year)
A Mazzocchi, S Soker, A Skardal (2019) 3D bioprinting for high-throughput screening: Drug screening, disease modeling, and precision medicine applications. Applied Physics Reviews, aip.scitation.org
AV Volkov, AV Ryzhenkov, AG Parygin (2018) To the question about the prospects to the use of trigeneration energy sources based on hydropower. J Appl Biotechnol …, researchgate.net
T Vujinović, RP Harrison METAGARDEN SPHERE2. media.virbcdn.com
G Unruh (2018) Circular economy, 3D printing, and the biosphere rules. California Management Review, journals.sagepub.com, cited by 3 (3.00 per year)

L Moroni, A Nandakumar, FB de Groot, ... (2015) Plug and play: combining materials and technologies to improve bone regenerative strategies. Journal of tissue …, Wiley Online Library, cited by 19 (4.75 per year)
JB Jones, DE Cooper, DI Wimpenny, GJ Gibbons (2012) Gateways Toward Dissimilar Multi-material Parts., dora.dmu.ac.uk
J Huang, H Fu, C Li, J Dai, Z Zhang (2017) Recent advances in cell-laden 3D bioprinting: materials, technologies and applications. Journal of 3D printing in …, Future Medicine, cited by 1 (0.50 per year)
K Chung, S Yu, CJ Heo, JW Shim, SM Yang, ... (2012) Flexible, angle-independent, structural color reflectors inspired by morpho butterfly wings. Advanced …, Wiley Online Library, cited by 168 (24.00 per year)
A Dhawan, PM Kennedy, EB Rizk, ... (2019) Three-dimensional bioprinting for bone and cartilage restoration in orthopaedic surgery. JAAOS-Journal of the …, journals.lww.com, cited by 1 (1.00 per year)
엄증태, 권승혁, 오재영, 이영지, 송용진, ... (2016) 조류의 부리에 대한 생체모방을 활용한 로봇팔 구조개선 융합 탐구-3D 모델링과 프린팅을 이용하여. 생물교육 (구 생물교육 …, papersearch.net, cited by 2 (0.67 per year)
MJ Domingue, DP Pulsifer, ... (2014) Fine-scale features on bioreplicated decoys of the emerald ash borer provide necessary visual verisimilitude. Bioinspiration …, spiedigitallibrary.org, cited by 3 (0.60 per year)
ZX Low, YT Chua, BM Ray, D Mattia, IS Metcalfe, ... (2017) Perspective on 3D printing of separation membranes and comparison to related unconventional fabrication techniques. Journal of Membrane …, Elsevier, cited by 70 (35.00 per year)
KM Rodriguez, TP Kohn, AB Davis, ... (2017) Penile implants: a look into the future. … andrology and urology, ncbi.nlm.nih.gov, cited by 6 (3.00 per year)
T Clayton (2015) The future is chemical. Chemistry in Australia, search.informit.com.au
S Allig, M Mayer, C Thielemann (2018) Workflow for bioprinting of cell-laden bioink. Lékař a technika-Clinician and …, ojs.cvut.cz
Y Liu, K Xiang, Y Li, H Chen, Q Hu (2014) Combining 3D Printing and Electrospinning for the Fabrication of a Bioabsorbable Poly-p-dioxanone Stent.. ISPE CE, books.google.com, cited by 1 (0.20 per year)
HA Almeida, ESG Oliveira (2016) Sustainability based on biomimetic design models. Handbook of Sustainability in Additive …, Springer, cited by 5 (1.67 per year)
K Narayanan, S Mishra, S Singh, M Pei, ... (2017) Engineering concepts in stem cell research. Biotechnology …, Wiley Online Library, cited by 5 (2.50 per year)
JO Gordeladze, HJ Haugen, ... (2017) Bone tissue engineering: state of the art, challenges, and prospects. … for Artificial Organs …, researchgate.net, cited by 9 (4.50 per year)
EB Kennedy (2017) Biomimicry in Industry: The Philosophical and Empirical Rationale for Reimagining R&D., rave.ohiolink.edu
Y Zhou, J Chyu, M Zumwalt (2018) Recent progress of fabrication of cell scaffold by electrospinning technique for articular cartilage tissue engineering. International journal of biomaterials, hindawi.com, cited by 2 (2.00 per year)
TK Merceron, SV Murphy (2015) Hydrogels for 3D bioprinting applications. Essentials of 3D biofabrication and translation, Elsevier, cited by 7 (1.75 per year)
C Beyer (2014) Strategic implications of current trends in additive manufacturing. Journal of Manufacturing Science and …, … .asmedigitalcollection.asme.org, cited by 96 (19.20 per year)

L Moroni, T Boland, JA Burdick, C De Maria, ... (2018) Biofabrication: a guide to technology and terminology. Trends in …, Elsevier, cited by 58 (58.00 per year)
DP Troskie (2017) The 4th industrial revolution: understanding its impact on the Western Cape's agricultural sector. Agriprobe, journals.co.za
J Nowell, J Connor, M Joordens, ... (2018) Analysis of the Effect Waveform Parameters have on Stingray Surface Velocity. 2018 13th Annual …, ieeexplore.ieee.org
L Labusca, U Greisser, K Mashayekhi (2016) Jacobs Journal of Bone Marrow and Stem Cell Research., pdfs.semanticscholar.org
A Agirbas A proposal for the use of fractal geometry algorithmically in tiling design. researchgate.net
MC Pires (2017) China, middle-income trap and the fourth industrial., bibliotecadigital.fgv.br
R Yang, A Zaheri, W Gao, C Hayashi, ... (2017) AFM identification of beetle exocuticle: Bouligand structure and nanofiber anisotropic elastic properties. Advanced Functional …, Wiley Online Library, cited by 21 (10.50 per year)
B Lorber, WK Hsiao, KR Martin (2016) Three-dimensional printing of the retina. Current opinion in ophthalmology, ncbi.nlm.nih.gov, cited by 15 (5.00 per year)
J Euchner (2016) Changing the Design Space., Taylor & Francis
Y Yang, X Song, X Li, Z Chen, C Zhou, ... (2018) Recent progress in biomimetic additive manufacturing technology: from materials to functional structures. Advanced …, Wiley Online Library, cited by 39 (39.00 per year)
C Evans-Pughe (2014) Learning from birds and bees [design]. Engineering & Technology, IET
J Snyder, AR Son, Q Hamid, C Wang, Y Lui, ... (2015) Mesenchymal stem cell printing and process regulated cell properties. Biofabrication, iopscience.iop.org, cited by 11 (2.75 per year)
R Naboni, A Kunic (2017) Design and Additive Manufacturing of Lattice-based Cellular Solids at Building Scale. Proceedings of Sigradi 2017, XXI Congress of …, researchgate.net, cited by 2 (1.00 per year)
NC Paxton, SK Powell, ... (2016) Biofabrication: The future of regenerative medicine. Techniques in …, ingentaconnect.com, cited by 6 (2.00 per year)
H Zhang, JK Nagel, A Al-Qas, E Gibbons, ... (2018) Additive Manufacturing with Bioinspired Sustainable Product Design: A Conceptual Model. Procedia Manufacturing, Elsevier, cited by 6 (6.00 per year)
M Kaminski, E Loth, C Qin, DT Griffith (2018) Gravo-Aeroelastic Scaling a 13.2 MW Wind Turbine Blade to a 1-meter Model. 2018 Wind Energy Symposium, arc.aiaa.org, cited by 2 (2.00 per year)
A Blaeser, SC Heilshorn, DF Duarte Campos (2019) Smart Bioinks as de novo Building Blocks to Bioengineer Living Tissues. Gels, mdpi.com
M Lopez, R Rubio, S Martín, ... (2015) Active materials for adaptive architectural envelopes based on plant adaptation principles. Journal of Facade …, content.iospress.com, cited by 28 (7.00 per year)
SSD Carter, X Liu, Z Yue, GG Wallace (2017) Three-dimensional neuronal cell culture: in pursuit of novel treatments for neurodegenerative disease. MRS Communications, cambridge.org
D Qu, CZ Mosher, MK Boushell, HH Lu (2015) Engineering Complex Orthopaedic Tissues Via Strategic Biomimicry. Annals of biomedical engineering, Springer, cited by 36 (9.00 per year)

F Taraballi, G Bauza, P McCulloch, ... (2017) Concise review: biomimetic functionalization of biomaterials to stimulate the endogenous healing process of cartilage and bone tissue. Stem cells …, Wiley Online Library, cited by 7 (3.50 per year)
S Lim, M Anand, T Rousek (2015) Estimation of energy and material use of sintering-based construction for a lunar outpost-with the example of SinterHab module design. 46th Lunar and Planetary Science …, oro.open.ac.uk, cited by 2 (0.50 per year)
AP Haring, H Sontheimer, BN Johnson (2017) Microphysiological human brain and neural systems-on-a-chip: potential alternatives to small animal models and emerging platforms for drug discovery and …. Stem cell reviews and reports, Springer, cited by 19 (9.50 per year)
M Temirel, B Yenilmez, S Knowlton, ... (2016) Three-dimensional-printed carnivorous plant with snap trap. 3D Printing and …, liebertpub.com, cited by 2 (0.67 per year)
RR Naik, S Singamaneni (2017) Introduction: Bioinspired and biomimetic materials., ACS Publications, cited by 11 (5.50 per year)
Z Huang, C Cai, G Wang, H Zhang, ... (2016) STRUCTURAL COLOR MODEL BASED ON SURFACE MORPHOLOGY OF MORPHO BUTTERFLY WING SCALE. Surface Review and …, World Scientific, cited by 5 (1.67 per year)
MJ Lerman, J Lembong, S Muramoto, ... (2018) The evolution of polystyrene as a cell culture material. … Engineering Part B …, liebertpub.com, cited by 6 (6.00 per year)
H Petersson (2017) Optimizing Products and Production Using Additive Manufacturing by Introducing Bionics Into the Engineering Design Process. ASME 2017 International …, … .asmedigitalcollection.asme.org
MJ de Oliveira, VM Rato, C Leitão (2018) KINE [SIS] TEM'17 From Nature to Architectural Matter International Conference. Nexus Network Journal, Springer
LE Murr, LE Murr (2015) Biomimetics and biologically inspired materials. … of materials structures, properties, processing and …, Springer, cited by 11 (2.75 per year)
DW Green, JS Lee, HS Jung (2016) Small-Scale Fabrication of Biomimetic Structures for Periodontal Regeneration. Frontiers in physiology, frontiersin.org, cited by 1 (0.33 per year)
T Campobasso (2015) Super Soldiers: 3D Bioprinting and the Future Fighter. Journal Article| Dec, community.apan.org, cited by 1 (0.25 per year)
L Chougrani, JP Pernot, P Véron, S Abed (2019) Parts internal structure definition using non-uniform patterned lattice optimization for mass reduction in additive manufacturing. Engineering with Computers, Springer
M Santoro, J Navarro, JP Fisher (2018) Micro-and Macrobioprinting: Current Trends in Tissue Modeling and Organ Fabrication. Small methods, Wiley Online Library, cited by 1 (1.00 per year)
DW Green, B Ben-Nissan, KS Yoon, B Milthorpe, ... (2017) Natural and synthetic coral biomineralization for human bone revitalization. Trends in …, Elsevier, cited by 13 (6.50 per year)
G Wright (2017) Bio-Inspired Wind Turbine Blade Profile Design., aura.alfred.edu
PD Innovation (2014) New Sustainable Materials for Product Designers., Institute of Technology, Carlow
P Wu (2019) Innovative Production And Construction: Transforming Construction Through Emerging Technologies., books.google.com
NAA Sayuti, MA Zainal, AFA Zamri, ... (2018) FROM CULTURE TO INNOVATIVE PRODUCT: BATIK DESIGN IN PORCELAIN WARES. Malaysian Journal of …, myjms.mohe.gov.my
H Lui, C Vaquette, R Bindra (2017) Tissue engineering in hand surgery: A technology update. The Journal of hand surgery, Elsevier, cited by 6 (3.00 per year)

Y Jiang, X Xv, D Liu, Z Yang, Q Zhang, H Shi, ... (2018) Preparation of Cellulose Nanofiber-reinforced Gelatin Hydrogel and Optimization for 3D Printing Applications. …, stargate.cnr.ncsu.edu
GS Forman, C Carvalho (2018) Design of sustainable textiles through biological systems and materials–innovative narratives within the circular economy. … : Design the Future: Proceedings of the …, books.google.com
VB Meyer (2015) Prototyping the Environmental Impacts of 3D Printing: Claims and Realities of Additive Manufacturing., fordham.bepress.com, cited by 1 (0.25 per year)
A Agkathidis Implementing Biomorphic Design. researchgate.net
BK Hsiung, TA Blackledge, ... (2019) Modulating iridescence in structural colors through hierarchy, micro-geometry, but randomness (Conference Presentation). … for Micro/Nano …, spiedigitallibrary.org
M Naghavi More Channels Showcase Channel Catalog. invasive152.rssing.com
AHC Au (2015) CYLINDRICAL 3-D BIOPRINTER., researchgate.net
C Chircov, AM Grumezescu (2019) Three-dimensional bioprinting in drug delivery. Materials for Biomedical Engineering, Elsevier
S Trabia (2018) Comprehensive Study of Spray-Painting and 3d Printing Fabrication Methods for Nafion® and Nafion® Equivalents in Ionic Polymer-Metal Composite Actuators and …., digitalscholarship.unlv.edu
DW Green, ST Kelly, KKH Lee, ... (2018) High-Definition X-Ray Imaging of Small Gecko Skin Surface Protuberances for Digitization and 3D Printing. Advanced Materials …, Wiley Online Library, cited by 1 (1.00 per year)
A Zaheri, JS Fenner, BP Russell, ... (2018) Revealing the mechanics of helicoidal composites through additive manufacturing and beetle developmental stage analysis. Advanced Functional …, Wiley Online Library, cited by 9 (9.00 per year)
C Krosinsky, W Martindale (2016) The future of innovation. Sustainable Investing: Revolutions …, books.google.com
SR Govindarajan (2016) … OF A MULTIFUNCTIONAL INITIATOR-FREE SOFT POLYESTER PLATFORM FOR ROOM-TEMPERATURE EXTRUSION-BASED 3D PRINTING, AND ANALYSIS OF …., rave.ohiolink.edu
P PITTAS EXPLORING SELF-REPAIRING MATERIALS AND THEIR APPLICATION TOWARDS SUSTAINABLE DESIGN. unswcode.org
S Stratton, NB Shelke, K Hoshino, S Rudraiah, ... (2016) Bioactive polymeric scaffolds for tissue engineering. Bioactive materials, Elsevier, cited by 87 (29.00 per year)
S Farah Synthesized Skins.
KS Lim, M Baptista, S Moon, TBF Woodfield, ... (2019) Microchannels in Development, Survival, and Vascularisation of Tissue Analogues for Regenerative Medicine. Trends in …, Elsevier
Ž Kačarević, P Rider, S Alkildani, S Retnasingh, ... (2018) An introduction to 3D bioprinting: possibilities, challenges and future aspects. Materials, mdpi.com, cited by 8 (8.00 per year)
B Natarajan, JW Gilman (2017) Bioinspired Bouligand cellulose nanocrystal composites: a review of mechanical properties. Philosophical Transactions of …, royalsocietypublishing.org, cited by 13 (6.50 per year)
하은아, 송민정, 조재경 (2016) 생체모방으로부터 영감을 얻은 3D 프린팅 활용 디자인. 기초조형학연구, papersearch.net, cited by 1 (0.33 per year)
M Buehler (2017) Tu (r) ning weakness to strength: Mechanomutable bioinspired materials., apps.dtic.mil

AF Martins, M Bessant, L Manukyan, MC Milinkovitch (2015) R2OBBIE-3D, a fast robotic high-resolution system for quantitative phenotyping of surface geometry and colour-texture. PloS one, journals.plos.org, cited by 12 (3.00 per year)
A Eltaweel, SU Yuehong (2017) Parametric design and daylighting: A literature review. Renewable and Sustainable Energy Reviews, Elsevier, cited by 24 (12.00 per year)
J Jang (2017) 3D bioprinting and in vitro cardiovascular tissue modeling. Bioengineering, mdpi.com, cited by 13 (6.50 per year)
EIMM Habib (2009) TOWARDS BIOGENESIS IN ARCHITECTURE., academia.edu
PN Sivasankaran, TA Ward, E Salami, ... (2017) An experimental study of elastic properties of dragonfly-like flapping wings for use in biomimetic micro air vehicles (BMAVs). Chinese Journal of …, Elsevier, cited by 5 (2.50 per year)
WU YUMO (2017) FORCE AND FORM: NATURE-INSPIRED COLUMNAR, PLANAR, AND FOAM STRUCTURES., scholarbank.nus.edu.sg
엄증태, 권용주 (2016) 생명과학에서 3D 모델링과 프린팅을 활용한 생체모방 중심 융합수업 프로그램의 개발. 생물교육 (구 생물교육학회지), papersearch.net, cited by 1 (0.33 per year)
V Ziegler, O Fritz (2017) InBetween: material encounters in human/non-human interactions. Proceedings of Intersections: Collaborations in …, researchgate.net
S Chen, SK Boda, SK Batra, X Li, ... (2018) Emerging roles of electrospun nanofibers in cancer research. Advanced healthcare …, Wiley Online Library, cited by 15 (15.00 per year)
J Li, J Fei, Y Xu, D Li, T Yuan, G Li, C Wang, J Li (2005) Angewandte., researchgate.net, cited by 1 (0.07 per year)
L Zhu, N Li, PRN Childs (2018) Light-weighting in aerospace component and system design. Propulsion and Power Research, Elsevier, cited by 3 (3.00 per year)
L Hoad, TA Erlandson, V Mulyani (2018) Mars: Design for the Red Planet. Interiors, Taylor & Francis
CM O'Brien, B Holmes, S Faucett, ... (2014) Three-dimensional printing of nanomaterial scaffolds for complex tissue regeneration. Tissue Engineering Part …, liebertpub.com, cited by 95 (19.00 per year)
A Ibrahim (2018) 3D bioprinting bone. 3D Bioprinting for Reconstructive Surgery, Elsevier
J Bargmann (2013) Urbee 2, the 3D-Printed Car That Will Drive Across the Country. Popular Mechanics, cited by 5 (0.83 per year)
J von Braun (2018) Bioeconomy–The global trend and its implications for sustainability and food security. Global food security, Elsevier, cited by 2 (2.00 per year)
M Kasonde, V Kanyanta (2016) Future of superhard material design, processing and manufacturing. … -Property Correlations for Hard, Superhard, and …, Springer, cited by 1 (0.33 per year)
S Li, H Bai, RF Shepherd, H Zhao (2019) Bioinspired design and additive manufacturing of soft materials, machines, robots, and haptic interfaces. Angewandte Chemie, Wiley Online Library
S Ford, M Despeisse, A Viljakainen (2015) Extending product life through additive manufacturing: the sustainability implications. Global Cleaner Production …, researchgate.net, cited by 7 (1.75 per year)
DW Green, B Ben-Nissan, KS Yoon, ... (2016) Bioinspired materials for regenerative medicine: going beyond the human archetypes. Journal of Materials …, pubs.rsc.org, cited by 10 (3.33 per year)
N Haskell (2016) Digital Utopia: The Role of Materially and Digital Competency. Materia Arquitectura

J Ma, Y Xue, X Liang, C Liao, Z Tan, B Tang (2019) Bi-directional regulatable mechanical properties of 3D braided polyetheretherketone (PEEK). Materials Science and …, Elsevier
AGT Fane (2018) A grand challenge for membrane desalination: More water, less carbon. Desalination, Elsevier, cited by 11 (11.00 per year)
J Liu, L Sun, W Xu, Q Wang, S Yu, J Sun (2018) Current advances and future perspectives of 3D printing natural-derived biopolymers. Carbohydrate polymers, Elsevier, cited by 4 (4.00 per year)
G Nicholls, K Youdim (2016) Emerging Transporter Science and Challenges for the Future. Drug Transporters, books.google.com
DA Foyt, MDA Norman, TL Tracy, ... (2018) Exploiting advanced hydrogel technologies to address key challenges in regenerative medicine. Advanced healthcare …, Wiley Online Library, cited by 10 (10.00 per year)
NB Swift IV (2016) Hedgemon: A Hedgehog-inspired Helmet Liner., rave.ohiolink.edu
E Garreta, R Oria, C Tarantino, M Pla-Roca, P Prado, ... (2017) Tissue engineering by decellularization and 3D bioprinting. Materials Today, Elsevier, cited by 55 (27.50 per year)
SGL Persiani, A Battisti (2018) Frontiers of adaptive design, synthetic biology and growing skins for ephemeral hybrid structures. Energy-Efficient Approaches in Industrial …, intechopen.com, cited by 1 (1.00 per year)
S Vijayavenkataraman, WF Lu, JYH Fuh (2016) 3D bioprinting–an ethical, legal and social aspects (ELSA) framework. Bioprinting, Elsevier, cited by 23 (7.67 per year)
S Mustafa, A Estim, AD Tuzan, CC Ann, LL Seng, ... Nature-Based and Technology-Based Solutions for Sustainable Blue Growth and Climate Change Mitigation in Marine Biodiversity Hotspots. researchgate.net
LG Bracaglia, BT Smith, E Watson, N Arumugasaamy, ... (2017) 3D printing for the design and fabrication of polymer-based gradient scaffolds. Acta biomaterialia, Elsevier, cited by 54 (27.00 per year)
L Kang, X Liu, Z Yue, Z Chen, C Baker, P Winberg, ... (2018) Fabrication and in vitro characterization of electrochemically compacted collagen/sulfated xylorhamnoglycuronan matrix for wound healing applications. Polymers, mdpi.com, cited by 6 (6.00 per year)
ÁC Molinari (2017) E-commerce for ecosystems. International Trade Forum, search.proquest.com
RPOO HOUSE (1953) PTICAL SOCIETY OF AMERICA., osarochester.org
T Luthe (2018) Systemic Design Lab: Incubating systemic design skills by experiential didactics and nature-based creativity. ETH Learning and Teaching Journal, learningteaching.ethz.ch
J Hawkins, P Sewell, M Dupac, T Macquart, D Lukac, ... PROCEEDINGS MEDO 2016. ieeexplore.ieee.org
Y Fang, T Zhang, L Zhang, W Gong, W Sun (2019) Biomimetic design and fabrication of scaffolds integrating oriented micro-pores with branched channel networks for myocardial tissue engineering. Biofabrication, iopscience.iop.org
G Kaushik, J Leijten, A Khademhosseini (2017) Concise review: organ engineering: design, technology, and integration. Stem Cells, Wiley Online Library, cited by 27 (13.50 per year)
JP Tate (2016) The Effects of Carbon Nanotubes on Cells in a Synthetic Oxygen Carrier Enriched Alginate Scaffold., digitalcommons.lsu.edu
F Hagn, C Thamm, T Scheibel, ... (2011) pH-Dependent Dimerization and Salt-Dependent Stabilization of the N-terminal Domain of Spider Dragline Silk—Implications for Fiber Formation. Angewandte Chemie …, Wiley Online Library, cited by 105 (13.13 per year)

RP Donahue, JC Hu, KA Athanasiou (2019) Remaining Hurdles for Tissue-Engineering the Temporomandibular Joint Disc. Trends in molecular medicine, Elsevier
GS Forman, C Carvalho (2017) Circular economy and biological materials—innovative narratives for the development of sustainable textile products. Challenges for Technology …, ingentaconnect.com
E Salami, E Montazer, TA Ward, ... (2017) Nano-mechanical properties and structural of a 3D-printed biodegradable biomimetic micro air vehicle wing. IOP Conference Series …, iopscience.iop.org
W Xie, V Kothari, BS Terry (2015) A bio-inspired attachment mechanism for long-term adhesion to the small intestine. Biomedical microdevices, Springer, cited by 11 (2.75 per year)
S Pollard, A Turney, F Charnley, K Webster (2016) The circular economy: A reappraisal of the stuff we 'love'. Geography, researchgate.net, cited by 13 (4.33 per year)
J FREITAS, A LEITÃO BACK TO REALITY. ist.utl.pt
P Morel (2019) The Origins of Discretism: Thinking Unthinkable Architecture. Architectural Design, Wiley Online Library
MJ Mirzaali, V Mussi, P Vena, F Libonati, L Vergani, ... (2017) Mimicking the loading adaptation of bone microstructure with aluminum foams. Materials & Design, Elsevier, cited by 8 (4.00 per year)
U Ghosh, S Ning, Y Wang, ... (2018) Addressing unmet clinical needs with 3D printing technologies. Advanced healthcare …, Wiley Online Library, cited by 13 (13.00 per year)
CJ Drol, EB Kennedy, BK Hsiung, NB Swift, KT Tan (2019) Bioinspirational understanding of flexural performance in hedgehog spines. Acta biomaterialia, Elsevier
J Jiang, MA Carlson, MJ Teusink, H Wang, ... (2015) Expanding two-dimensional electrospun nanofiber membranes in the third dimension by a modified gas-foaming technique. ACS Biomaterials …, ACS Publications, cited by 38 (9.50 per year)
GC Sresty (2017) MENTOR 2., apps.dtic.mil
A du Plessis, SG le Roux, M Tshibalanganda (2019) Advancing X-ray micro computed tomography in Africa: going far, together. Scientific African, Elsevier, cited by 1 (1.00 per year)
S Prendeville, E Cherim, N Bocken (2018) Circular cities: mapping six cities in transition. Environmental innovation and societal …, Elsevier, cited by 43 (43.00 per year)
M Malinauskas, E Skliutas, ... (2015) Tailoring bulk mechanical properties of 3D printed objects of polylactic acid varying internal micro-architecture. Quantum Optics …, spiedigitallibrary.org, cited by 7 (1.75 per year)
D Barlex (2011) Dear minister, this is why design and technology is a very important subject in the school curriculum. Design and Technology Education: An International …, ojs.lboro.ac.uk, cited by 24 (3.00 per year)
TSS Kumar, VY Chakrapani (2018) Electrospun 3D Scaffolds for Tissue Regeneration. Cutting-Edge Enabling Technologies for …, Springer, cited by 1 (1.00 per year)
MM Durban, JM Lenhardt, AS Wu, ... (2018) Custom 3D printable silicones with tunable stiffness. Macromolecular …, Wiley Online Library, cited by 8 (8.00 per year)
MSC Ryan 12 Using Soft Robotics Technologies in Order to Construct a Biomimetic Robotic Prehensile Tail as Found in New World Monkeys for Use as a Robotic …. believeinohio.org
H Johnston (2018) Giant baby planet spotted within planet-forming disc. Physics World, iopscience.iop.org

DA Chen (2016) The Adaptable Growth of Seashells: Informing the Design of the Built Environment through Quantitative Biomimicry., tigerprints.clemson.edu, cited by 1 (0.33 per year)
M Bordoni, F Rey, V Fantini, O Pansarasa, ... (2018) From Neuronal Differentiation of iPSCs to 3D Neuro-Organoids: Modelling and Therapy of Neurodegenerative Diseases. International journal of …, mdpi.com, cited by 6 (6.00 per year)
Y Zhou (2017) The recent development and applications of fluidic channels by 3D printing. Journal of biomedical science, jbiomedsci.biomedcentral.com, cited by 4 (2.00 per year)
GS Watson, JA Watson, BW Cribb (2017) Diversity of cuticular micro-and nanostructures on insects: Properties, functions, and potential applications. Annual review of …, annualreviews.org, cited by 20 (10.00 per year)
A du Plessis, SG le Roux, M Tshibalanganda (2019) Scientific African., researchgate.net
A Musetescu (2014) RE_MOVE: A Biomimetics inspired movable habitat structure for research and exploration purposes., repository.tudelft.nl
LF Boesel, C Greiner, E Arzt, ... (2010) Gecko-inspired surfaces: a path to strong and reversible dry adhesives. Advanced Materials, Wiley Online Library, cited by 338 (37.56 per year)
C Großardt, A Ewald, LM Grover, JE Barralet, ... (2010) Passive and Active In Vitro Resorption of Calcium and Magnesium Phosphate Cements by Osteoclastic Cells. … Engineering Part A, liebertpub.com, cited by 84 (9.33 per year)
T LIDDELL (2018) TESSILE. Design proposal for the use of elasticized textiles in responsive window coverings., politesi.polimi.it
R Levato, WR Webb, IA Otto, A Mensinga, Y Zhang, ... (2017) The bio in the ink: cartilage regeneration with bioprintable hydrogels and articular cartilage-derived progenitor cells. Acta biomaterialia, Elsevier, cited by 57 (28.50 per year)
I Ivan (2014) Mars Continuum., repository.tudelft.nl
JR Chang (2018) Defining a Novel Meaning of the New Organic Architecture. A+ BE| Architecture and the Built …, superheroscitech.tudelft.nl
V Ozbolat, M Dey, B Ayan, A Povilianskas, ... (2018) 3D printing of PDMS improves its mechanical and cell adhesion properties. ACS Biomaterials …, ACS Publications, cited by 15 (15.00 per year)
D Lizoňová, Z Tončíková (2019) EXPLORING THE APPLICATION OF NATURE-INSPIRED GEOMETRIC PRINCIPLES WHEN DESIGNING FURNITURE AND INTERIOR EQUIPMENT. Acta Facultatis Xylologiae Zvolen …, search.proquest.com
S BEKY, F DEROLEZ (2015) CIRCULAR ECONOMY APPLIED TO HUMAN RESSOURCES MANAGEMENT. Tendances sociales et culturelles de la …, Editions L'Harmattan
H Park, H Choi, JR Usherwood Nano-mechanical properties and structural of a 3D-printed biodegradable biomimetic micro air vehicle wing. researchportal.hw.ac.uk
M Lei, X Wang (2016) Biodegradable polymers and stem cells for bioprinting. Molecules, mdpi.com, cited by 27 (9.00 per year)
N Oxman, M Kayser, J Laucks, ... (2013) Robotically controlled fiber-based manufacturing as case study for biomimetic digital fabrication. Green Design, Materials …, books.google.com, cited by 8 (1.33 per year)
A Munaz, RK Vadivelu, JS John, M Barton, ... (2016) Three-dimensional printing of biological matters. Journal of Science …, Elsevier, cited by 38 (12.67 per year)
J Jang, JY Park, G Gao, DW Cho (2018) Biomaterials-based 3D cell printing for next-generation therapeutics and diagnostics. Biomaterials, Elsevier, cited by 41 (41.00 per year)

M Lewandowski (2018) Public Sector and Circular Business Models: From Public Support Towards Implementation Through Design. Sustainable Business Models, Springer
T Hayes (2018) Among (21st) century school children: The'new literateurs'a response to'new literacies' in the middle years. Literacy Learning: The Middle Years, search.informit.com.au
C Kengla, E Renteria, C Wivell, A Atala, ... (2017) Clinically Relevant Bioprinting Workflow and Imaging Process for Tissue Construct Design and Validation. 3D Printing and …, liebertpub.com, cited by 5 (2.50 per year)
Y Wu, Y Han, YS Wong, JYH Fuh (2018) Fibre-based scaffolding techniques for tendon tissue engineering. Journal of tissue …, Wiley Online Library, cited by 8 (8.00 per year)
M Eng (2010) Exploring property driven design fabrication through materials testing and software development., dspace.mit.edu
A Astreinidi Blandin, I Bernardeschi, L Beccai (2018) Biomechanics in soft mechanical sensing: From natural case studies to the artificial world. Biomimetics, mdpi.com, cited by 1 (1.00 per year)
N Hoeller, A Goel, C Freixas, R Anway, ... (2013) Developing a common ground for learning from nature. Zygote …, bioinspired.sinet.ca, cited by 9 (1.50 per year)
T Ruberto, G Polverino, M Porfiri (2017) How different is a 3D-printed replica from a conspecific in the eyes of a zebrafish?. Journal of the experimental …, Wiley Online Library, cited by 14 (7.00 per year)
BS Kennedy (2017) The de Mestral Project: using macro photo-journaling to stimulate interest in bio-inspired design and science, technology, engineering and mathematics disciplines. International Journal of Design & Nature and …, witpress.com
J Wang (2017) Trabecular topology: computational structural design inspired by bone remodeling., dspace.mit.edu
K Kikegawa, K Takamatsu, M Kawakami, ... (2017) Evaluation of 3D Printer Accuracy in Producing Fractal Structure. Journal of oleo …, jstage.jst.go.jp
C Vendrely, T Scheibel (2007) Biotechnological production of spider-silk proteins enables new applications. Macromolecular bioscience, Wiley Online Library, cited by 219 (18.25 per year)
L Badarnah (2017) Form follows environment: biomimetic approaches to building envelope design for environmental adaptation. Buildings, mdpi.com, cited by 8 (4.00 per year)
M Husack (2015) Aiding lower-limb amputees in traversing uneven terrain through product design., smartech.gatech.edu
R Bernabei, J Power (2016) Living Designs. Conference on Biomimetic and Biohybrid Systems, Springer
A du Plessis, C Broeckhoven, I Yadroitsava, ... (2019) Beautiful and functional: a review of biomimetic design in additive manufacturing. Additive …, Elsevier, cited by 5 (5.00 per year)
G Genta (2016) Are there severe limitations to the bioinspired approach in machine design?. Proceedings of the Institution of Mechanical …, journals.sagepub.com, cited by 4 (1.33 per year)
YS Zhang, Y Xia (2015) Multiple facets for extracellular matrix mimicking in regenerative medicine. Nanomedicine, Future Medicine, cited by 26 (6.50 per year)
JA Serna, SL Florez, VA Talero, JC Briceño, ... (2019) Formulation and Characterization of a SIS-Based Photocrosslinkable Bioink. Polymers, mdpi.com
M Despeisse, M Baumers, P Brown, F Charnley, ... (2017) Unlocking value for a circular economy through 3D printing: A research agenda. … Forecasting and Social …, Elsevier, cited by 89 (44.50 per year)

V Pillay, P Kumar, YE Choonara (2015) Integrated biomaterial composites for accelerated wound healing. Biomaterials in Regenerative Medicine …, Springer, cited by 2 (0.50 per year)
MECSS Thong, MECW Wen 3D Printing–Revolutionising Military Operations. Pointer J. Singap. Armed Forces, mindef.gov.sg, cited by 1 (0.00 per year)
R Watson (2015) TABLE OF TRENDS & TECHNOLOGIES FOR THE WORLD IN 2020., nowandnext.com, cited by 2 (0.50 per year)
K Jakab, C Norotte, B Damon, F Marga, ... (2008) Tissue engineering by self-assembly of cells printed into topologically defined structures. … Engineering Part A, liebertpub.com, cited by 283 (25.73 per year)
O Kırdök, A Tokuç (2018) 0102-SOLAR REGULATION IN ARCHITECTURE WITH 3D FIBROUS PRODUCTION METHODS. FROM SCIENTIFIC EDITORS, researchgate.net
DW Green, GS Watson, JA Watson, JM Lee, ... (2018) Simulated embryonic and fetal cellular dynamics inside structured biomaterials. Applied Materials …, Elsevier
S Varma, J Voldman, J Marzioch, J Kieninger, A Weltin, ... (2018) Devices and applications at the micro-and nanoscale rsc. li/loc. Lab Chip, pubs.rsc.org
LM Ricles, JC Coburn, M Di Prima, ... (2018) Regulating 3D-printed medical products. Science translational …, stm.sciencemag.org, cited by 9 (9.00 per year)
M Dade-Robertson, ... (2017) Bio-materialism: Experiments in biological material computation. Proceedings of the …, researchonline.rca.ac.uk
D HES, J BUSH STUDIO OUTLINE.
G Kelly (2018) Summer 2018 Studio 5., edsc.unimelb.edu.au
D Das, I Noh (2018) Overviews of biomimetic medical materials. Biomimetic Medical Materials, Springer, cited by 1 (1.00 per year)
E Kalayci, OO Avinc, A Yavas FUTURISTIC DESIGN APPROACHES FOR NEW GENERATION TEXTILES. TEXTILE SCIENCE AND ECONOMY, academia.edu
O Tricinci, T Terencio, B Mazzolai, ... (2015) 3D Micropatterned Surface Inspired by Salvinia molesta via Direct Laser Lithography. … applied materials & …, ACS Publications, cited by 42 (10.50 per year)
M Juvonen (2018) Autodesk Inventor ja lisäohjelmat 3D-tulostamiseen., theseus.fi
JM Mayfield (2018) Synthesis and Behavior Characterization of Multi-Scale Hierarchical Structured Composites., digitalcommons.georgiasouthern …
B Passlack, A Ehrmann, K Finsterbusch, M Niederrhein 3D-Druck–eine neue industrielle Revolution. researchgate.net
GM Elrayies (2018) Architectural ornaments in the twenty-first century: An analytical study'. Cities' Identity Through Architecture and Arts–Catalani …, diva-portal.org, cited by 2 (2.00 per year)
J Ku, K Jang, M Lee, J Lim, S Park, ... (2018) P-BE14: The Effects of The Salt on Chitosan Bioink for 3D Bioprinting. 한국농업기계학회 학술 …, papersearch.net
김영민, 임경란 (2017) 3D 프린팅을 활용한 창의 융합 디자인 교육프로그램 모델 개발: 초등학생 창의교육을 중심으로. 한국디자인문화학회지, dbpia.co.kr
구본강, 박상준, 최동진, 김천호 (2017) 1P-187 Fabrication of optimized 3D scaffolds with various process parameters adjustment in 3D printing. 한국공업화학회 연구논문 초록집, papersearch.net
MAM Jahromi, A Abdoli, M Rahmanian, ... (2019) Microfluidic Brain-on-a-Chip: Perspectives for Mimicking Neural System Disorders. Molecular …, Springer

공미식, 정우재 (2017) 1P-186 Colorimetric Biomimetic Sensor System based on Self-Assembled Peptide Films for Aldehyde Gases Detection. 한국공업화학회 연구논문 초록집, papersearch.net
윤예지, 윤종헌, 이낙규, 박석희 (2017) 전기방사 나노섬유를 포함한 하이드로겔 프린팅 연구. 한국생산제조학회 학술발표대회 논문집
김수연, 권용주 (2018) 생체모방 기반 융합 학습 모델을 적용한 '골격근의 구조와 수축'에 대한 디지털 교재 개발. 과학교육연구지, papersearch.net
T Penko (2017) Tehnologija 3-D tiskanja oblačil v modnem oblikovanju. Tekstilec, tekstilec.si
김명환, 남승윤 (2018) 알지네이트 하이드로겔의 유변학적 특성 및 3D printability 에 미치는 카라기난의 영향성. 한국정밀공학회 학술발표대회 논문집, dbpia.co.kr
MA dos Santos Laranjeira, ... (2014) DESIGN DE SUPERFÍCIE E OS NOVOS PARADIGMAS DA CIÊNCIA. Blucher Design …, proceedings.blucher.com.br
구현철, 한가득, 손태일 (2017) 1P-189 Preparation of thermo-sensitive hydrogel for drug delivery. 한국공업화학회 연구논문 초록집, papersearch.net
구본강, 박상준, 김천호 (2017) 1P-188 The beneficial effect on cell infiltration and viability on porous chitosan nanofiber scaffolds. 한국공업화학회 연구논문 초록집, papersearch.net
고은주, 문정인, 박희민, 이용택 (2017) 1P-185 전기방사를 통한 용해성이 우수한 나노파이버의 제조. 한국공업화학회 연구논문 초록집, papersearch.net
박호현 (2016) 요리스 라만과 프랑수아 로쉬의 작업에 나타난 디지털 도구와 바이오미미크리 특성에 관한 연구. 한국공간디자인학회 논문집, papersearch.net
권기완, 이우걸, 박찬희, 안혜림, 조훈영, ... (2017) 1P-190 Biomineral coated on protein-polymer hybrid 3D scaffolds for tissue engineering. 한국공업화학회 연구 …, papersearch.net
M Hacker, D Crismond, D Hecht, ... (2017) engineering for all: a middle school program to introduce students to engineering as a potential social good.. Technology & …, search.ebscohost.com, cited by 3 (1.50 per year)
AN Leberfinger, S Dinda, Y Wu, SV Koduru, V Ozbolat, ... (2019) Bioprinting functional tissues. Acta biomaterialia, Elsevier, cited by 1 (1.00 per year)
E Rovalo, J McCardle, E Smith, ... (2019) Growing the practice of biomimicry: opportunities for mission-based organisations based on a global survey of practitioners. Technology Analysis & …, Taylor & Francis
YJ Grobman (2013) Cellular Building Envelopes. ICoRD'13, Springer, cited by 3 (0.50 per year)
M Marien (2018) Book Review of Come On!: A Report to the Club of Rome: New Frontiers. Cadmus, cadmusjournal.org
M López, R Rubio, S Martín, B Croxford (2017) How plants inspire façades. From plants to architecture: Biomimetic principles for the development of adaptive architectural envelopes. Renewable and Sustainable …, Elsevier, cited by 33 (16.50 per year)

M Mashkour, T Kimura, M Mashkour, ... (2018) Printing Birefringent Figures by Surface Tension-Directed Self-Assembly of a Cellulose Nanocrystal/Polymer Ink Components. … applied materials & …, ACS Publications
M Despeisse, S Ford, A Viljakainen Product life extension through additive manufacturing. researchgate.net, cited by 1 (0.00 per year)
T Masseck, E Den Ouden, R Valkenburg (2017) Roadmaps for Energy (R4E): Smart Buildings General Roadmap., upcommons.upc.edu
K Kuusk, O Tomico, G Langereis, ... (2012) Crafting smart textiles: a meaningful way towards societal sustainability in the fashion field?. Nordic Textile Journal, diva-portal.org, cited by 14 (2.00 per year)
J Koerner (2017) Digitally Crafted Couture. Architectural Design, Wiley Online Library
ZZ Gu, H Uetsuka, K Takahashi, ... (2003) Structural color and the lotus effect. Angewandte Chemie …, Wiley Online Library, cited by 456 (28.50 per year)
OZ Fisher, A Khademhosseini, R Langer, ... (2009) Bioinspired materials for controlling stem cell fate. Accounts of chemical …, ACS Publications, cited by 281 (28.10 per year)
T Gupta, SE Swiontek, A Lakhtakia (2015) Simpler Mass Production of Polymeric Visual Decoys for the Male Emerald Ash Borer (AgriAgrilus planipennis). Journal of Bionic Engineering, Elsevier, cited by 5 (1.25 per year)
F Engineer, K Energy Randall B. Hellman.
N Hu, P Feng, G Dai (2013) The gift from nature: bio-inspired strategy for developing innovative bridges. Journal of Bionic Engineering, Elsevier, cited by 19 (3.17 per year)
HA Bruck, AL Gershon, I Golden, ... (2007) Training mechanical engineering students to utilize biological inspiration during product development. Bioinspiration & …, iopscience.iop.org, cited by 40 (3.33 per year)
C Collet (2017) Grow-Made Textiles., ualresearchonline.arts.ac.uk, cited by 2 (1.00 per year)
T Koleva, E Titianova (2017) Principles of Three-Dimensional Bioprinting in Medicine. NEUROSONOLOGY AND CEREBRAL …, researchgate.net
NRW Lipholt (2019) Biomimicry-Where Nature is Changing Innovation., essay.utwente.nl
R Van Berkel, Z Fadeeva Role of Industries in Resource Efficiency and Circular Economy. researchgate.net
D Rana, TS Kumar, ... (2017) Impact of nanotechnology on 3D bioprinting. Journal of Bionanoscience, ingentaconnect.com, cited by 3 (1.50 per year)
M Burke, BM Carter, AW Perriman (2017) Bioprinting: uncovering the utility layer-by-layer. Journal of 3D printing in …, Future Medicine, cited by 1 (0.50 per year)
PN Sivasankaran, TA Ward, E Salami, R Viyapuri, ... (2017) An experimental study of elastic properties of., cyberleninka.org
A Tokuç, FF Özkaban, ÖA Çakır (2018) Biomimetic Facade Applications for a More Sustainable Future. … With the Power of Biomimicry, books.google.com
SA Abbah, LM Delgado, A Azeem, ... (2015) Harnessing hierarchical nano-and micro-fabrication technologies for musculoskeletal tissue engineering. Advanced …, Wiley Online Library, cited by 38 (9.50 per year)
SS Xie, O Vasylkiv, V Silberschmidt, ... (2013) Bio-inspired structured boron carbide-boron nitride composite by reactive spark plasma sintering: This paper describes the prototyping of an approach to improve …. Virtual and Physical …, Taylor & Francis, cited by 2 (0.33 per year)
JF Leary (2019) Design of sophisticated shaped, multilayered, and multifunctional nanoparticles for combined in-vivo imaging and advanced drug delivery. Nanoscale Imaging, Sensing, and Actuation for …, spiedigitallibrary.org

S Ushiba, S Shoji, K Masui, J Kono, ... (2014) Direct laser writing of 3D architectures of aligned carbon nanotubes. Advanced …, Wiley Online Library, cited by 36 (7.20 per year)
M Gazit (2016) Living matter: biomaterials for design and architecture., dspace.mit.edu, cited by 2 (0.67 per year)
LA Beck (2014) New as Renewal: A Framework for Adaptive Reuse in the Sustainable Paradigm., scholarworks.umass.edu, cited by 1 (0.20 per year)
R Edgeman (2018) Urgent evolution: excellence and wicked Anthropocene Age challenges. Total Quality Management & Business Excellence, Taylor & Francis, cited by 1 (1.00 per year)
MJ Kang (2016) High performance curtain wall mullion section design with various densities of gyroid., dspace.mit.edu
CC Haddal, DL Bull, PMP Hernandez, JT Foley (2017) Reducing Future International Chemical and Biological Dangers., researchgate.net
T Jiang, J Munguia-Lopez, S Flores-Torres, ... (2018) Bioprintable Alginate/Gelatin Hydrogel 3D In Vitro Model Systems Induce Cell Spheroid Formation. JoVE (Journal of …, jove.com, cited by 1 (1.00 per year)
N West, K Sammut, Y Tang (2018) Material selection and manufacturing of riblets for drag reduction: An updated review. Proceedings of the Institution of …, journals.sagepub.com, cited by 3 (3.00 per year)
C Pothecary An Evaluation of the Impact of Open Source Digital Desktop Fabrication Technologies on Socially Responsible Design. academia.edu
V Minucciani, NS Onay (2018) Evaluation of Design Approaches for Wellbeing in Interiors. Journal of Engineering, researchgate.net
L Badarnah Environmental adaptation of buildings through morphological differentiation. researchgate.net
N Lee, J Robinson, H Lu (2016) Biomimetic strategies for engineering composite tissues. Current opinion in biotechnology, Elsevier, cited by 21 (7.00 per year)
J Kuusniemi (2016) Exploration of network-based digital business ecosystems and the models within., theseus.fi, cited by 1 (0.33 per year)
T Page (2017) Advanced Design Applications of Polymers Developed for Additive Manufacturing. i-Manager's Journal on Material Science, search.proquest.com
L Badarnah (2018) Environmental adaptation of buildings through morphological differentiation: A biomimetic approach., eprints.uwe.ac.uk
AC Balazs, GM Whitesides, CJ Brinker, IS Aranson, ... (2016) Designing Biomimetic, Dissipative Material Systems., osti.gov
S Vijayavenkataraman, L Zhang, S Zhang, ... (2018) Triply Periodic Minimal Surfaces Sheet Scaffolds for Tissue Engineering Applications: An Optimization Approach toward Biomimetic Scaffold Design. ACS Applied Bio …, ACS Publications, cited by 6 (6.00 per year)
RS Aldrich, S Benton, L Schaper, ... (2013) Sustainable thinking. THE GREEN …, books.google.com, cited by 1 (0.17 per year)
G Chabaud, M Castro, A Le Duigou (2019) Impression 3D et 4D de matériaux composites à fibres de carbone continues: des applications structurelles au morphing de structures 3D and 4D., hal.archives-ouvertes.fr
DB Patel, M Santoro, LJ Born, JP Fisher, SM Jay (2018) Towards rationally designed biomanufacturing of therapeutic extracellular vesicles: impact of the bioproduction microenvironment. Biotechnology advances, Elsevier, cited by 4 (4.00 per year)
MJ Domingue, A Lakhtakia, ... (2014) Bioreplicated visual features of nanofabricated buprestid beetle decoys evoke stereotypical male mating flights. Proceedings of the …, National Acad Sciences, cited by 17 (3.40 per year)

S Chen, R Li, X Li, J Xie (2018) Electrospinning: An enabling nanotechnology platform for drug delivery and regenerative medicine. Advanced drug delivery reviews, Elsevier, cited by 21 (21.00 per year)
LC du Toit, P Kumar, YE Choonara, V Pillay (2018) Use of nanostructured materials in hard tissue engineering. Nanobiomaterials, Elsevier, cited by 1 (1.00 per year)
S Sharif (2010) The Confluence of Digital Design/Fabrication and Biological Principles: Systematic knowledge transfer for the development of integrated architectural systems., dspace.mit.edu, cited by 1 (0.11 per year)
H Matamu (2012) Speculation on architecture in the absence of gravity: how might architectural elements and design strategies function in outer space?., unitec.researchbank.ac.nz
SP Nadeem, JA Garza-Reyes, D Glanville (2018) The Challenges of the Circular Economy. Contemporary Issues in …, Springer
V Sumini, C Mueller, C Chesi (2018) Design and fabrication of the Martian habitat prototype, WATER. Proceedings of IASS Annual …, ingentaconnect.com, cited by 1 (1.00 per year)
C Brass, F Mazzarella (2015) Are we asking the right questions? Rethinking post-graduate design education towards sustainable visions for the future., dspace.lboro.ac.uk, cited by 3 (0.75 per year)
CS Adjiman, NM Harrison, SZ Weider (2017) Molecular science and engineering: a powerful transdisciplinary approach to solving grand challenges., spiral.imperial.ac.uk
SA Ásgeirsdóttir (2013) Biomimicry in Iceland: Present Status and Future Significance., skemman.is, cited by 3 (0.50 per year)
C Collet, G Foissac (2015) Botanical Fabrication: A research project at the intersection of design, botany and horticulture., ualresearchonline.arts.ac.uk, cited by 2 (0.50 per year)
N Dulake, I Gwilt (2017) Flying with data: Openness, forms and understanding.. The Design Journal, Taylor & Francis, cited by 2 (1.00 per year)
I Deniz, T Keskin-Gundogdu (2018) Biomimetic Design for a Bioengineered World. … With the Power of Biomimicry, books.google.com
C Chircov (2018) Biomaterials and Tissue Engineering Bulletin. Biomaterials, researchgate.net
Z Zhang, LH Klausen, M Chen, M Dong (2018) Electroactive Scaffolds for Neurogenesis and Myogenesis: Graphene-Based Nanomaterials. Small, Wiley Online Library, cited by 5 (5.00 per year)
HB Kim, DK Patel, YR Seo, KT Lim (2019) 3D-Printed Scaffolds with Reinforced Poly (Lactic Acid)/Carbon Nanotube Filaments Based on Melt Extrusion. Journal of Biosystems Engineering, Springer
DS Finch, T Oreskovic, K Ramadurai, ... (2008) Biocompatibility of atomic layer-deposited alumina thin films. … Research Part A: An …, Wiley Online Library, cited by 73 (6.64 per year)
Y Hwang, RN Candler (2017) Non-planar PDMS microfluidic channels and actuators: a review. Lab on a Chip, pubs.rsc.org, cited by 13 (6.50 per year)
M Amatullo (2014) the mOst WicKed prOBLem OF aLL: impLementatiOn. Where can design have the greatest …, designmattersatartcenter.org, cited by 2 (0.40 per year)
M Biondi, F Ungaro, F Quaglia, PA Netti (2008) Controlled drug delivery in tissue engineering. Advanced drug delivery reviews, Elsevier, cited by 348 (31.64 per year)
LJ Burton, N Cheng, JWM Bush (2014) The cocktail boat., academic.oup.com, cited by 7 (1.40 per year)
M Cruz, R Beckett (2016) Bioreceptive design: A novel approach to biodigital materiality. Arq: Architectural Research Quarterly, cambridge.org, cited by 4 (1.33 per year)

P Moazzam, H Tavassoli, A Razmjou, ME Warkiani, ... (2018) Mist harvesting using bioinspired polydopamine coating and microfabrication technology. Desalination, Elsevier, cited by 13 (13.00 per year)
M Hospodiuk, M Dey, D Sosnoski, IT Ozbolat (2017) The bioink: a comprehensive review on bioprintable materials. Biotechnology advances, Elsevier, cited by 147 (73.50 per year)
Y Liu, K Xiang, H Chen, Y Li, Q Hu (2015) Composite vascular repair grafts via micro-imprinting and electrospinning. AIP Advances, aip.scitation.org, cited by 12 (3.00 per year)
Y Wu (2019) Novel technique produces micropores, allowing diffusion of nutrients in biofabricated tissues. Journal of 3D printing in medicine, Future Medicine
JG Fernandez, DE Ingber (2012) Unexpected strength and toughness in chitosan-fibroin laminates inspired by insect cuticle. Advanced materials, Wiley Online Library, cited by 57 (8.14 per year)
O Kırdök, D Altun, D Dokgöz, A Tokuç (2018) REHABILITATING THE CARBON CYCLE WITH BIODESIGN IN ARCHITECTURE., researchgate.net
BM Ogle, N Bursac, I Domian, NF Huang, ... (2016) Distilling complexity to advance cardiac tissue engineering. Science translational …, stm.sciencemag.org, cited by 62 (20.67 per year)
J Loy, S Canning (2016) Clash of Cultures: Fashion, Engineering, and 3D Printing. Creative Technologies for Multidisciplinary …, igi-global.com, cited by 2 (0.67 per year)
RK Katzschmann, J DelPreto, ... (2018) Exploration of underwater life with an acoustically controlled soft robotic fish. Science …, robotics.sciencemag.org, cited by 40 (40.00 per year)
J Zhou, DA Khodakov, AV Ellis, NH Voelcker (2012) Surface modification for PDMS-based microfluidic devices. Electrophoresis, Wiley Online Library, cited by 214 (30.57 per year)
J Liu, Z Guan, X Chen, C Xu (2018) Digital Morphogenesis: A Synthetic Approach to Generate Architectural Elaborate Components. 2017 3rd International Forum on …, atlantis-press.com
K AGRAWAL MODERN METHODS OF CONSTRUCTION AND ITS FEASIBILITY ANALYSIS. researchgate.net
S Bukner, F Dialami, L Ding, ... (2015) Bio-inspired design to support reduced energy consumption via the 'light weighting'of machine system elements. International Journal of …, eprints.uwe.ac.uk, cited by 2 (0.50 per year)
R Edgeman Urgent evolution: excellence and wicked. Total Quality Management & Business Excellence, pure.au.dk
GX Gu (2018) Bioinspired algorithmic-driven design of additively manufactured composites., dspace.mit.edu
김헌, 최성경, 문정민, 노현지, 이용일, 박지혜, ... MC 에셔의 인지적 착시 (多시점) 가 TV 광고에 나타난 표현특성에 관한 연구.
R Solanki (2015) A life on Mars: an architectural research project into the creation of a permanent human presence on the surface of Mars., unitec.researchbank.ac.nz
E Solanas, I Pla-Palacín, P Sainz-Arnal, M Almeida, ... (2018) Tissue Organoids: Liver. Tumor Organoids, Springer
W Liu, J Lipner, J Xie, CN Manning, ... (2014) Nanofiber scaffolds with gradients in mineral content for spatial control of osteogenesis. … applied materials & …, ACS Publications, cited by 80 (16.00 per year)
CM Teven, S Fisher, GA Ameer, TC He, ... (2015) Biomimetic approaches to complex craniofacial defects. Annals of maxillofacial …, ncbi.nlm.nih.gov, cited by 21 (5.25 per year)

M Rimmer (2016) 3D printing Jurassic Park: Copyright law, cultural institutions, and makerspaces. Pandora's Box, papers.ssrn.com, cited by 2 (0.67 per year)

S Knowlton, A Joshi, B Yenilmez, IT Ozbolat, ... (2016) Advancing cancer research using bioprinting for tumor-on-a-chip platforms. Int J …, tasoglulab.net, cited by 19 (6.33 per year)

D Singh (2018) 3D bioprinting for scaffold fabrication. 3D Bioprinting for Reconstructive Surgery, Elsevier

AM Holban, A Grumezescu (2019) Materials for Biomedical Engineering: Nanomaterials-based Drug Delivery., books.google.com

P Chandra, C Kengla, SJ Lee (2016) 1 Microfabrication and. … Medicine Technology: On-a-Chip …, books.google.com

JY Park, J Jang, HW Kang (2018) 3D Bioprinting and its application to organ-on-a-chip. Microelectronic Engineering, Elsevier, cited by 2 (2.00 per year)

NA Hammad, MA Hammad (2017) Sustainable Design Thinking: Adaptability, Resilience, and Productivity at the Core of Regionally Responsive Architecture., researchgate.net

O Diegel, P Kristav, D Motte, B Kianian (2016) Additive manufacturing and its effect on sustainable design. Handbook of sustainability in …, Springer, cited by 7 (2.33 per year)

S Camere, E Karana (2017) Growing materials for product design. … of the International Conference of the DRS …, researchgate.net, cited by 3 (1.50 per year)

JO Wolff, D Wells, CR Reid, ... (2017) Clarity of objectives and working principles enhances the success of biomimetic programs. Bioinspiration & …, iopscience.iop.org, cited by 5 (2.50 per year)

A Eltom, G Zhong, A Muhammad (2019) Scaffold Techniques and Designs in Tissue Engineering Functions and Purposes: A Review. Advances in Materials Science and …, hindawi.com

PDE Baniqued, JR Dungao, ... (2018) Biomimetics in the design of a robotic exoskeleton for upper limb therapy. AIP Conference …, aip.scitation.org, cited by 2 (2.00 per year)

B Farahi (2016) Caress of the gaze: A gaze actuated 3D printed body architecture. 36th Annual Conference of the Association for …, papers.cumincad.org, cited by 6 (2.00 per year)

S Koskela, T Mattila, R Antikainen, I Mäenpää (2013) Identifying key sectors and measures for a transition towards a low resource economy. Resources, mdpi.com, cited by 16 (2.67 per year)

K Schacht, T Jüngst, M Schweinlin, ... (2015) Biofabrication of cell-loaded 3D spider silk constructs. Angewandte Chemie …, Wiley Online Library, cited by 104 (26.00 per year)

F Libonati, AE Vellwock, F Ielmini, D Abliz, ... (2019) Bone-inspired enhanced fracture toughness of de novo fiber reinforced composites. Scientific reports, nature.com, cited by 1 (1.00 per year)

A Paio, S Eloy, VM Rato, R Resende, MJ de Oliveira (2012) Prototyping vitruvius, new challenges: Digital education, research and practice. Digital Fabrication, Springer, cited by 14 (2.00 per year)

JR Chang (2018) From Interactive to Intra-active Body. A+ BE| Architecture and the Built …, superheroscitech.tudelft.nl

S Vijayavenkataraman, WC Yan, WF Lu, ... (2018) 3D bioprinting of tissues and organs for regenerative medicine. Advanced drug delivery …, Elsevier, cited by 29 (29.00 per year)

DM Aukes, HB Amor, K Luck, M Jansen, ... (2019) Systems and methods for rapid-prototyped robotic devices. US Patent App. 16 …, Google Patents

B von Mengersen (2018) Textiles, a Dynamic Exchange: Challenges and Opportunities. Handbook of Technology Education, Springer

H Gu, Y Zhao, Y Cheng, Z Xie, F Rong, J Li, B Wang, ... (2013) Tailoring colloidal photonic crystals with wide viewing angles. Small, Wiley Online Library, cited by 55 (9.17 per year)
C Kayat, C Magalhães (2017) Experimentos biomiméticos e novas tecnologias digitais para o design de embalagem. DAT Journal, datjournal.anhembi.br
A Almaraz (2015) Evolutionary optimization of parametric structures: understanding structure and architecture as a whole from early design stages., ruc.udc.es, cited by 1 (0.25 per year)
A de Mel, T Yap, G Cittadella, LR Hale, ... (2015) A potential platform for developing 3D tubular scaffolds for paediatric organ development. Journal of Materials …, Springer, cited by 11 (2.75 per year)
ВА Плеханова (2015) 3D-технологии и их применение в дизайне. Территория новых возможностей. Вестник …, cyberleninka.ru, cited by 4 (1.00 per year)
P Huebner Dos Reis (2018) 3D-Bioplotted Scaffold for Autograft Substitution in Osteochondral Plug Transfer Approaches: Design and Process Characterization.., repository.lib.ncsu.edu
JH Kim, A Atala, JJ Yoo (2017) Translation and Applications of Biofabrication. 3D Printing and Biofabrication, Springer
H Kopnina, J Blewitt (2018) Human resource management, green jobs, and a green economy. Sustainable Business, 2nd Edition, ingentaconnect.com
S Lee (2017) Research of Future Furniture Design: Exploring Trends and Aesthetics in Futurism (2017)., libres.uncg.edu
R Masaeli, K Zandsalimi, M Rasoulianboroujeni, ... (2019) Challenges in 3D Printing of Bone Substitutes. Tissue …, liebertpub.com
JJ Mohr BMKT 460.01: Marketing Hi-Technology Products and Innovations. pdfs.semanticscholar.org
AS Mathews, S Abraham, SK Kumaran, J Fan, ... (2017) Bio nano ink for 4D printing membrane proteins. RSC Advances, pubs.rsc.org, cited by 2 (1.00 per year)
ML Stern (2015) Aligning design and development processes for additive manufacturing., dspace.mit.edu, cited by 2 (0.50 per year)
IM Sandvik, W Stubbs (2019) Circular fashion supply chain through textile-to-textile recycling. Journal of Fashion Marketing and …, emeraldinsight.com
J Gerdes, A Holness, A Perez-Rosado, L Roberts, ... (2014) Robo Raven: a flapping-wing air vehicle with highly compliant and independently controlled wings. Soft …, liebertpub.com, cited by 48 (9.60 per year)
F Cavas-Martínez, B Eynard, FJF Cañavate, ... (2019) Advances on Mechanics, Design Engineering and Manufacturing II., Springer
M Orquéra, S Campocasso, D Millet (2017) Design for additive manufacturing method for a mechanical system downsizing. Procedia CIRP, Elsevier, cited by 18 (9.00 per year)
TA Lenau, AM Orrù, L Linkola (2018) Biomimicry in the Nordic Countries., diva-portal.org
T Spencer (2017) Additive Manufacturing Materials and Design Considerations for Thunniform Propulsion. The Equilibrium, escholarship.org
RT Edwards (2016) Performance of a Biomimetically Inspired Notch Design., rave.ohiolink.edu
A Battisti, SGL Persiani, M Crespi (2019) Review and Mapping of Parameters for the Early Stage Design of Adaptive Building Technologies through Life Cycle Assessment Tools. Energies, mdpi.com

P Gruber, T McGinley, ... (2017) Towards an agile biodigital architecture: Supporting a dynamic evolutionary and developmental view of architecture. … the Power of Biomimicry, books.google.com, cited by 2 (1.00 per year)
L Bechthold, V Fischer, A Hainzlmaier, D Hugenroth, ... (2015) 3D printing: A qualitative assessment of applications, recent trends and the technology's future potential., econstor.eu, cited by 19 (4.75 per year)
F Cleymand, M Rousseau, JF Mano (2015) Introducing biomimetic approaches to materials development and product design for engineering students. Bioinspired Biomimetic …, researchgate.net, cited by 1 (0.25 per year)
강지인, 박수용, 정일두 (2017) 1P-184 Synthesis of Biodegradable Nanoparticles Based on Polyfumarateutethane. 한국공업화학회 연구논문 초록집, papersearch.net
K Ling, G Huang, J Liu, X Zhang, Y Ma, T Lu, F Xu (2015) Bioprinting-based high-throughput fabrication of three-dimensional MCF-7 human breast cancer cellular spheroids. Engineering, Elsevier, cited by 32 (8.00 per year)
CT Chen, GX Gu (2019) Effect of Constituent Materials on Composite Performance: Exploring Design Strategies via Machine Learning. Advanced Theory and Simulations, Wiley Online Library
T Kamps, M Gralow, G Schlick, G Reinhart (2017) Systematic biomimetic part design for additive manufacturing. Procedia CIRP, Elsevier, cited by 10 (5.00 per year)
S Patel, JM Caldwell, SB Doty, ... (2018) Integrating soft and hard tissues via interface tissue engineering. Journal of …, Wiley Online Library, cited by 8 (8.00 per year)
M Deng, SG Kumbar, LS Nair, AL Weikel, ... (2011) Biomimetic Structures: Biological Implications of Dipeptide-Substituted Polyphosphazene–Polyester Blend Nanofiber Matrices for Load-Bearing Bone Regeneration. Advanced Functional …, Wiley Online Library, cited by 104 (13.00 per year)
CF de Lima, LA van der Elst, VN Koraganji, ... (2019) Towards Digital Manufacturing of Smart Multimaterial Fibers. Nanoscale Research …, Springer
PA Netti, L Ambrosio (2011) Multifunctional polymer based structures for human tissues reconstruction. Composite Materials, Springer, cited by 1 (0.13 per year)
TR Kelley, E Sung (2017) design fixation. Technology and Engineering Teacher, search.proquest.com
S Deuerling, S Kugler, M Klotz, C Zollfrank, ... (2018) A Perspective on Bio-Mediated Material Structuring. Advanced …, Wiley Online Library, cited by 2 (2.00 per year)
P Gruber, B Imhof (2017) Patterns of Growth—Biomimetics and Architectural Design. Buildings, mdpi.com, cited by 1 (0.50 per year)
D Motro, S Tosunoglu Development of Vision Systems for Search and Rescue Robotics. pdfs.semanticscholar.org
R Naboni, A Kunic, ... (2017) Load-responsive cellular envelopes with additive manufacturing. Journal of Facade …, superheroscitech.tudelft.nl, cited by 5 (2.50 per year)
E Gawell, A Nowak, W Rokicki (2019) Searching for Bionics Structural Forms Optimization. IOP Conference Series …, iopscience.iop.org
N Rashedi Banafshehvaragh (2018) 3D bioprinting using hydrogel and synthetic fillers., dk.upce.cz
I Paoletti, L Ceccon (2018) The Evolution of 3D Printing in AEC: From Experimental to Consolidated Techniques. 3D Printing, books.google.com

S Abdulghani, PG Morouço (2019) Biofabrication for osteochondral tissue regeneration: bioink printability requirements. Journal of Materials Science: Materials in …, Springer, cited by 1 (1.00 per year)
S Allig Controlled Cell Formation Using Bioprinting. poseidon2.feld.cvut.cz
BO Okesola, C Redondo-Gómez, A Mata (2018) Multicomponent self-assembly: Supramolecular design of complex hydrogels for biomedical applications. Self-assembling Biomaterials, Elsevier
R Oxman Naturalizing Architecture Archilab 2013 Orléans France April 2013 Naturalizing Design: In Pursuit of Tectonic Materiality. academia.edu
M Cianchetti, C Laschi, A Menciassi, ... (2018) Biomedical applications of soft robotics. Nature Reviews Materials, nature.com, cited by 37 (37.00 per year)
C Bakker, R Mugge (2017) î î î îï î Àe. Delft University of Technology, pure.tudelft.nl
L Moroni (2016) Tempo: timing & metamorphosis in biofabrication., cris.maastrichtuniversity.nl
A Judge (2014) Representation of Creative Processes through Dynamics in Three Dimensions., laetusinpraesens.org
A EDUCATION (2000) GABOR KOSA PhD., eng.tau.ac.il
Z Tong, A Solanki, A Hamilos, O Levy, K Wen, ... (2015) Application of biomaterials to advance induced pluripotent stem cell research and therapy. The EMBO …, embopress.org, cited by 53 (13.25 per year)
J Liu (2018) Engineering of vascular networks within biocompatible hydrogels using 3D printing technology., mediatum.ub.tum.de
W Rokicki, A Stefańska (2019) Architectural Ephemerids in Terms of Generative and Parametric Design., ijscer.com
PA Wieringa, AR Gonçalves de Pinho, ... (2018) Biomimetic architectures for peripheral nerve repair: a review of biofabrication strategies. Advanced …, Wiley Online Library, cited by 9 (9.00 per year)
SK Pazhanimala, D Vllasaliu, BT Raimi-Abraham (2019) Electrospun Nanometer to Micrometer Scale Biomimetic Synthetic Membrane Scaffolds in Drug Delivery and Tissue Engineering: A Review. Applied Sciences, mdpi.com
W Wilson, YM Choi (2018) A Multi-disciplinary Approach to Design in Education Applied to Medical Problems. International Conference on Applied Human Factors …, Springer
M Despeisse, M Yang, S Evans, S Ford, T Minshall (2017) Sustainable value roadmapping framework for additive manufacturing. Procedia CIRP, Elsevier, cited by 15 (7.50 per year)
Y Xing, P Jones, M Bosch, I Donnison, ... (2018) Exploring design principles of biological and living building envelopes: what can we learn from plant cell walls?. Intelligent Buildings …, Taylor & Francis
H Philamore, J Rossiter, A Stinchcombe, ... (2015) Row-bot: An energetically autonomous artificial water boatman. 2015 IEEE/RSJ …, ieeexplore.ieee.org, cited by 9 (2.25 per year)
CG Helguero, VM Mustahsan, ... (2017) Biomechanical properties of 3D-printed bone scaffolds are improved by treatment with CRFP. Journal of …, josr-online.biomedcentral.com, cited by 5 (2.50 per year)
D Tolfree, A Smith Healthcare products and materials inspired by nature. cmmmagazine.com
R Armstrong (2017) Experimental Architecture and Unconventional Computing. Advances in Unconventional Computing, Springer, cited by 3 (1.50 per year)
JFV Vincent (2016) Biomimetics in architectural design. Intelligent Buildings International, Taylor & Francis, cited by 11 (3.67 per year)

C Kim, T Ruberto, P Phamduy, M Porfiri (2018) Closed-loop control of zebrafish behaviour in three dimensions using a robotic stimulus. Scientific reports, nature.com, cited by 16 (16.00 per year)
S Ford, M Despeisse (2016) Additive manufacturing and sustainability: an exploratory study of the advantages and challenges. Journal of Cleaner Production, Elsevier, cited by 336 (112.00 per year)
N Biloria, JR Chang A bio-inspired information design framework for real-time adaptive spatial components. academia.edu
SGK Madiraju, JJ Wallen, SP Rydelek, RE Carrion, ... (2019) Biomechanical Studies of the Inflatable Penile Prosthesis: A Review. Sexual medicine …, Elsevier
P Moerbeck (2019) Biological Principles of Nanostructured Hydroxyapatite Associated with Metals: A Literature Review. EC Orthopaedics, ecronicon.com
M Gürsoy, M Karaman (2017) Surfaces in Nature. Surface …, Wiley-VCH Verlag GmbH & Co …
R Antikainen, D Lazarevic, J Seppälä (2018) Circular economy: origins and future orientations. Factor X, Springer, cited by 3 (3.00 per year)
RX Wu, Y Yin, XT He, X Li, FM Chen (2017) Engineering a cell home for stem cell homing and accommodation. Advanced Biosystems, Wiley Online Library, cited by 19 (9.50 per year)
A Pagano Didactics articles now available online and in print. emis.ams.org
N Biloria, JR Chang (2012) Hypercell: A bio-inspired information design framework for real-time adaptive spatial components., papers.cumincad.org, cited by 1 (0.14 per year)
N Almouemen, HM Kelly, C O'Leary (2019) Tissue engineering: Understanding the role of biomaterials and biophysical forces on cell functionality through computational and structural biotechnology …. Computational and structural …, Elsevier
HC Huang, YJ Chang, WC Chen, HIC Harn, ... (2013) Enhancement of renal epithelial cell functions through microfluidic-based coculture with adipose-derived stem cells. … Engineering Part A, liebertpub.com, cited by 29 (4.83 per year)
DB Kell, E Lurie-Luke (2015) The virtue of innovation: innovation through the lenses of biological evolution. Journal of The Royal Society …, royalsocietypublishing.org, cited by 15 (3.75 per year)
R Soar (2016) Part 2: Pushing the envelope. A process perspective for architecture, engineering and construction. Intelligent Buildings International, Taylor & Francis, cited by 1 (0.33 per year)
W Luo, H Liu, C Wang, Y Qin, Q Liu, ... (2019) Bioprinting of Human Musculoskeletal Interface. Advanced Engineering …, Wiley Online Library
R Portillo-Lara, ES Sani, N Annabi (2017) Biomimetic Orthopedic Materials. Orthopedic Biomaterials, Springer, cited by 1 (0.50 per year)
J Zhu, M Dexheimer, H Cheng (2017) Reconfigurable systems for multifunctional electronics. npj Flexible Electronics, nature.com, cited by 12 (6.00 per year)
L Wu, I Chauhan, Y Tadesse (2018) A novel soft actuator for the musculoskeletal system. Advanced Materials …, Wiley Online Library, cited by 6 (6.00 per year)
M Moreno, R Court, M Wright, ... (2019) Opportunities for redistributed manufacturing and digital intelligence as enablers of a circular economy. International Journal of …, Taylor & Francis, cited by 4 (4.00 per year)
Y Wu, P Kennedy, N Bonazza, Y Yu, A Dhawan, ... (2018) Three-Dimensional Bioprinting of Articular Cartilage: A Systematic Review. Cartilage, journals.sagepub.com, cited by 2 (2.00 per year)

DJ Byard, AL Woern, RB Oakley, MJ Fiedler, ... (2019) Green fab lab applications of large-area waste polymer-based additive manufacturing. Additive …, Elsevier, cited by 4 (4.00 per year)
CTW Thanthrige (2013) Emergent Alternative Home 2050., scholarspace.manoa.hawaii.edu
A Graham, JHP Bayley (2015) The design and implementation of a 3D-bioprinter., ora.ox.ac.uk
V Townsend (2010) Relating additive and subtractive processes teleologically for hybrid design and manufacturing., scholar.uwindsor.ca, cited by 1 (0.11 per year)
L Kagali (2014) EMOTION DESIGN OF AN ICONIC (VOLKSWAGEN) CAMPERVAN., theseus.fi
X Cherny-Scanlon (2016) Putting glam into green: A case for sustainable luxury fashion. Spirituality and sustainability, Springer, cited by 2 (0.67 per year)
JD Lee, SA Moore (2017) The trajectory of architectural research in the UK and US: 1995–2016. arq: Architectural Research Quarterly, cambridge.org
H Henderson, TJ Nash, R Sanquiche, H Henderson, ... (2013) Green Transition Inflection Point: Green Transition Scoreboard® 2013 Report Cover: Green Construction–green cover over green tech Cover design by Regine …., ethicalmarkets.com
JD Lee, SA Moore The Trajectory of Architectural Research in the UK and United States: 1995-2016. researchgate.net
R Oxman Naturalizing Architecture Archilab 2013 Orléans France April 2013. academia.edu
NB Swift, BK Hsiung, EB Kennedy, KT Tan (2016) Dynamic impact testing of hedgehog spines using a dual-arm crash pendulum. Journal of the mechanical …, Elsevier, cited by 12 (4.00 per year)
RS Nathani (2015) Three-Dimensional Fabrication of Scaffolds for Drug Discovery and Regenerative Medicine., indigo.uic.edu
H Turunen (2016) Additive Manufacturing and Value Creation-in Architectural Design, Design Process and End-products., papers.cumincad.org, cited by 2 (0.67 per year)
Q Zhao, Y Wang, H Cui, X Du (2019) Bio-inspired sensing and actuating materials. Journal of Materials Chemistry C, pubs.rsc.org, cited by 1 (1.00 per year)
A Keune, KA Peppler, KE Wohlwend (2019) Recognition in makerspaces: Supporting opportunities for women to "make" a STEM career. Computers in Human Behavior, Elsevier
S Park, D Kim, S Park, S Kim, D Lee, W Kim, ... (2018) Nanopatterned Scaffolds for Neural Tissue Engineering and Regenerative Medicine. Cutting-Edge Enabling …, Springer
Y Zhou (2016) The application of ultrasound in 3d bio-printing. Molecules, mdpi.com, cited by 10 (3.33 per year)
F Klassen Matter, Material and Making. filiz-klassen.com
L Yan, Z Yao, T Lin, Q Zhu, J Qi, L Gu, J Fang, ... (2017) The role of precisely matching fascicles in the quick recovery of nerve function in long peripheral nerve defects. …, ncbi.nlm.nih.gov, cited by 7 (3.50 per year)
MA Fernandez-Yague, SA Abbah, L McNamara, ... (2015) Biomimetic approaches in bone tissue engineering: Integrating biological and physicomechanical strategies. Advanced drug delivery …, Elsevier, cited by 180 (45.00 per year)
G Bressanelli, F Adrodegari, M Perona, ... (2018) The role of digital technologies to overcome Circular Economy challenges in PSS Business Models: an exploratory case study. Procedia …, researchgate.net, cited by 6 (6.00 per year)
K Zhang, S Wang, C Zhou, L Cheng, X Gao, X Xie, ... (2018) Advanced smart biomaterials and constructs for hard tissue engineering and regeneration. Bone research, nature.com, cited by 5 (5.00 per year)

K Oliver, A Seddon, RS Trask (2016) Morphing in nature and beyond: a review of natural and synthetic shape-changing materials and mechanisms. Journal of Materials Science, Springer, cited by 30 (10.00 per year)
COF SILKwORMS silk paVilion: a case study in Fibre-based digital Fabrication. JSTOR
AH Flood (2016) Creating molecular macrocycles for anion recognition. Beilstein journal of organic chemistry, beilstein-journals.org, cited by 16 (5.33 per year)
S Xia, Z Wang, H Chen, W Fu, J Wang, Z Li, L Jiang (2015) Nanoasperity: structure origin of nacre-inspired nanocomposites. ACS nano, ACS Publications, cited by 38 (9.50 per year)
BT Hoffmann (2018) Characterization of Regenerated Silk Material for Biomimetic Spinning and Film Casting., library.ndsu.edu
TM Rankin, BA Wormer, JD Miller, ... (2018) Image once, print thrice? Three-dimensional printing of replacement parts. The British journal of …, birpublications.org, cited by 5 (5.00 per year)
P Pelli (2016) Service ecosystems as frameworks to elaborate sustainable futures. Tiziana Russo-Spenaand Cristina Mele, researchgate.net, cited by 1 (0.33 per year)
JH Jeong, V Chan, C Cha, P Zorlutuna, ... (2012) "Living" microvascular stamp for patterning of functional neovessels; orchestrated control of matrix property and geometry. Advanced …, Wiley Online Library, cited by 56 (8.00 per year)
AS Gill, PK Deol, IP Kaur (2019) An Update on the Use of Alginate in Additive Biofabrication Techniques. Current pharmaceutical design, ingentaconnect.com
TS Shim, SH Kim, CJ Heo, HC Jeon, ... (2012) Controlled origami folding of hydrogel bilayers with sustained reversibility for robust microcarriers. Angewandte Chemie …, Wiley Online Library, cited by 143 (20.43 per year)
P Chandra, A Atala (2019) Engineering blood vessels and vascularized tissues: technology trends and potential clinical applications. Clinical Science, clinsci.org
LE Van den Heuvel (2016) Toward functional magnetic applications for multi-material inkjet 3D printing., dspace.mit.edu
R Raman, R Langer (2019) Biohybrid Design Gets Personal: New Materials for Patient-Specific Therapy. Advanced Materials, Wiley Online Library
AG Fane, R Wang, MX Hu (2015) Synthetic membranes for water purification: status and future. Angewandte Chemie International …, Wiley Online Library, cited by 244 (61.00 per year)
R Morcos (2010) Structural Biomimetic Integration in the Formation of Load-Bearing Skins., cpas-egypt.com
H Dill (2016) Advantages, enablers and barriers to implementing circular economic principles in South African financial services organisations., repository.up.ac.za, cited by 1 (0.33 per year)
C Chung, O France, J Lee, I Mehrotra, R Phillips Mechanical Snake. pdfs.semanticscholar.org
M Hovilehto (2016) Characterization of design of a product for additive manufacturing., lutpub.lut.fi, cited by 1 (0.33 per year)
L Pambaguian, E van Schreven, ... (2018) Space Hardware Advanced Manufacturing Engineering: SHAME to miss out on a potential game changer?. Concurrent …, journals.sagepub.com
N Hauptmann, Q Lian, J Ludolph, H Rothe, ... (2019) Biomimetic Designer Scaffolds Made of D,L-Lactide-ε-Caprolactone Polymers by 2-Photon Polymerization. … Engineering Part B …, liebertpub.com

MT Poldervaart, H Gremmels, K van Deventer, ... (2014) Prolonged presence of VEGF promotes vascularization in 3D bioprinted scaffolds with defined architecture. Journal of controlled …, Elsevier, cited by 99 (19.80 per year)
AS Neto, JM Ferreira (2019) BIPHASIC CALCIUM PHOSPHATE SCAFFOLDS DERIVED FROM HYDROTHERMALLY SYNTHESIZED POWDERS. Lékař a technika-Clinician and Technology, ojs.cvut.cz
HW Ooi, S Hafeez, CA Van Blitterswijk, L Moroni, ... (2017) Hydrogels that listen to cells: a review of cell-responsive strategies in biomaterial design for tissue regeneration. Materials …, pubs.rsc.org, cited by 31 (15.50 per year)
M Lennaárd (2017) MADE IN SWEDEN: Förslag till en möbel av lokala hållbara material tillverkad med nya digitala produktionsverktyg.., diva-portal.org
M Vaezi, H Seitz, S Yang (2013) A review on 3D micro-additive manufacturing technologies. The International Journal of Advanced …, Springer, cited by 648 (108.00 per year)
AD Lantada (2016) Addressing the Complexity of Biomaterials by Means of Biomimetic Computer Aided Design. Microsystems for Enhanced Control of Cell Behavior, Springer
CAM Versos, DA Coelho (2012) Bionic Design: Presentation of a Two Way Methodology. Design Principles and Practices: an …, researchgate.net, cited by 3 (0.43 per year)
C Vyas, G Poologasundarampillai, J Hoyland, ... (2017) 3D printing of biocomposites for osteochondral tissue engineering. Biomedical …, Elsevier, cited by 3 (1.50 per year)
MJ Song, D Dean, MLK Tate (2012) Optimization of Tissue Engineering Scaffold Geometry, Seeding & Flow Conditions to Steer Stem Cell Shape and Fate. We hereby approve the thesis …, etd.ohiolink.edu, cited by 2 (0.29 per year)
MJ Hill, M Mahmoudi, PPSS Abadi (2019) Nanobiomaterial Advances in Cardiovascular Tissue Engineering. Cardiovascular Regenerative …, Springer
EK Hendow, P Guhmann, ... (2016) Biomaterials for hollow organ tissue engineering. … & tissue repair, fibrogenesis.biomedcentral.com, cited by 16 (5.33 per year)
G Aquadro (2013) OpuntiaGenesis: The hybrid generating plant., open.uct.ac.za
M Puyuelo Cazorla, P Fuentes-Durá, ... (2016) The opportunity in Industrial Design Engineering. A profile that matches the challenges of contemporary design. Elisava TdD (English …, riunet.upv.es
G Vignesh, RN Raghavan, ... (2015) Relevance of 3D Bioprinting in Tissue Engineering Application: Concept and Perspectives.. … in Biomaterials & …, search.ebscohost.com
MR Abir, TE Tay, HP Lee (2019) On the improved ballistic performance of bio-inspired composites. Composites Part A: Applied Science and …, Elsevier
D Andrews (2015) The circular economy, design thinking and education for sustainability. Local Economy, journals.sagepub.com, cited by 115 (28.75 per year)
JW Lee, PX Lan, B Kim, G Lim, ... (2008) Fabrication and characteristic analysis of a poly (propylene fumarate) scaffold using micro-stereolithography technology. Journal of Biomedical …, Wiley Online Library, cited by 79 (7.18 per year)
J Wang, Z Wang, Z Song, L Ren, ... (2019) Biomimetic Shape–Color Double-Responsive 4D Printing. Advanced Materials …, Wiley Online Library
B Cantrell (2015) Synthetic Mudscapes. Paradigms in Computing: Making, Machines, and …, books.google.com, cited by 1 (0.25 per year)
M Park (2017) 3D printing and product repair. Routledge Handbook of Sustainable Product Design, books.google.com, cited by 2 (1.00 per year)
F Libonati, GX Gu, Z Qin, L Vergani, ... (2016) Bone-inspired materials by design: toughness amplification observed using 3D printing and testing. Advanced Engineering …, Wiley Online Library, cited by 58 (19.33 per year)

AD Lantada (2016) Towards Effective and Efficient Biofabrication Technologies. Microsystems for Enhanced Control of Cell Behavior, Springer
B Sung, MH Kim (2018) Liquid-crystalline nanoarchitectures for tissue engineering. Beilstein journal of nanotechnology, beilstein-journals.org, cited by 3 (3.00 per year)
M Farr (2017) MaterialNature: An Opportunistic Paradigm of Architecture and Landscape Ecology., theplanjournal.com
E Cimetta, A Godier-Furnémont, ... (2013) Bioengineering heart tissue for in vitro testing. Current opinion in …, Elsevier, cited by 25 (4.17 per year)
AS Deshpande, I Burgert, O Paris (2006) Hierarchically Structured Ceramics by High-Precision Nanoparticle Casting of Wood. Small, Wiley Online Library, cited by 66 (5.08 per year)
G Maliaris, IT Sarafis, T Lazaridis, ... (2016) Random lattice structures. Modelling, manufacture and FEA of their mechanical response. IOP Conference …, researchgate.net, cited by 2 (0.67 per year)
M Laukkanen, J Huiskonen, ... (2015) Promoting sustainable business model innovation with scenario planning. ISPIM Conference …, search.proquest.com, cited by 1 (0.25 per year)
MT Maybury (2013) Global Horizons (Briefing Charts)., apps.dtic.mil
J Yoon (2018) SMP Prototype Design and Fabrication for Thermo-responsive Façade Elements. Journal of Facade Design and Engineering, superheroscitech.tudelft.nl
IM Sandvik Applying circular economy to the fashion industry in Scandinavia through textile-to-textile recycling. mistrafuturefashion.com
R Pal (2017) Sustainable design and business models in textile and fashion industry. Sustainability in the Textile Industry, Springer, cited by 7 (3.50 per year)
TR Nachtigall, O Tomico, R Wakkary, ... (2018) Towards ultra personalized 4D printed shoes. Extended Abstracts of …, dl.acm.org, cited by 5 (5.00 per year)
B Ben-Nissan (2015) Discovery and development of marine biomaterials. Functional marine biomaterials, Elsevier, cited by 3 (0.75 per year)
SI Meghna, SS Chowdhury (2013) Contextual customization of design process. Design through the digital and the material., papers.cumincad.org
BB de Vries, TU Chairman, ITT Kunen, BG BV (2016) Building Circularity Indicators., pure.tue.nl
J Idaszek, M Costantini, TA Karlsen, J Jaroszewicz, ... (2019) 3D bioprinting of hydrogel constructs with cell and material gradients for the regeneration of full-thickness chondral defect using a microfluidic printing head. …, iopscience.iop.org
H Pickering (2016) Conversational ecologies., digitalwindow.vassar.edu, cited by 1 (0.33 per year)
PCG Glas (2016) Cities and regions–Connected by water in mutual dependency., oecd-ilibrary.org
G AKBAŞ, O Okan, S BİLGİN THREE DIMENSIONAL PRINTER APPLICATIONS IN THE HEALTH SECTOR: CURRENT SITUATION AND FUTURE. International Journal of 3D Printing …, dergipark.org.tr
PS Mehta, JS Ocampo, A Tovar, P Chaudhari (2016) Bio-inspired design of lightweight and protective structures., sae.org, cited by 4 (1.33 per year)
S Tsunenari, T Oya (2016) Method for evaluating mechanical characteristics of biological material for bio-inspired lightweight design. Computer-Aided Design and Applications, Taylor & Francis, cited by 3 (1.00 per year)

D Munro (2013) Development of an automated manufacturing course with lab for undergraduates. 2013 IEEE Frontiers in Education Conference (FIE), ieeexplore.ieee.org, cited by 7 (1.17 per year)
A Agirbas (2019) Façade form-finding with swarm intelligence. Automation in Construction, Elsevier, cited by 2 (2.00 per year)
AG Mikos, SW Herring, P Ochareon, J Elisseeff, ... (2006) Engineering complex tissues. Tissue …, liebertpub.com, cited by 517 (39.77 per year)
N Futas, K Rajput, R Schiano-Phan (2019) Cradle to Cradle and Whole-Life Carbon assessment–Barriers and opportunities towards a circular economic building sector. IOP Conference Series …, iopscience.iop.org
PK Paritala, T Yarlagadda, R Sreeram, ... (2017) Impact of digital manufacturing on health care industry., eprints.qut.edu.au
M Li, F He, Q Liao, J Liu, L Xu, L Jiang, ... (2008) Ultrasensitive DNA detection using photonic crystals. Angewandte Chemie …, Wiley Online Library, cited by 144 (13.09 per year)
P Trogadas, JIS Cho, TP Neville, J Marquis, ... (2018) A lung-inspired approach to scalable and robust fuel cell design. Energy & …, pubs.rsc.org, cited by 14 (14.00 per year)
RPTWO DESIGN (2017) Workshop Report., events.tti.tamu.edu
Y Cai, L Lin, Z Xue, M Liu, S Wang, ... (2014) Filefish-inspired surface design for anisotropic underwater oleophobicity. Advanced Functional …, Wiley Online Library, cited by 120 (24.00 per year)
D King (2014) Ossature: Bone Remodeling as a Generative Structuring Process in Architecture., curve.carleton.ca, cited by 1 (0.20 per year)
C Jansona, A Palmqvista (2017) Formation mechanisms of iron-nitrogen functionalized mesoporous carbons as non-precious metal cathode catalysts for fuel cells. SCIENCE & TECHNOLOGY DAY, chalmers.se
GD Belyea, L Kawashiri, D Rinker, K Beske (2016) Biomimetic Seal Flipper Test Rig., digitalcommons.calpoly.edu
KT Faber, T Asefa, M Backhaus-Ricoult, ... (2017) The role of ceramic and glass science research in meeting societal challenges: Report from an NSF-sponsored workshop. Journal of the …, Wiley Online Library, cited by 8 (4.00 per year)
K Kapat, S Dhara (2017) Biopolymers Modification and Their Utilization in Biomimetic Composites for Osteochondral Tissue Engineering. Handbook of Composites from Renewable …, John Wiley & Sons, cited by 1 (0.50 per year)
P Jensen-Haxel (2015) A New Framework for a Novel Lattice: 3D Printers, DNA Fabricators, and the Perils in Regulating the Raw Materials of the Next Era of Revolution, Renaissance …. Wake Forest JL & Pol'y, HeinOnline, cited by 5 (1.25 per year)
N Peltonen (2018) Suunnittelu 3D-tulostimelle NX CAD-ohjelmistolla., theseus.fi
E Greenberg (2015) Innovating the Joint: Connectivity Strategies for Higher Functionality in Architectural Design. Proceedings of IASS Annual Symposia, ingentaconnect.com, cited by 1 (0.25 per year)
G Dwivedi, K Flynn, M Resnick, S Sampath, ... (2015) Bioinspired hybrid materials from spray-formed ceramic templates. Advanced …, Wiley Online Library, cited by 34 (8.50 per year)
H Hava, L Zhou, EM Lombardi, K Cui, H Joung, ... SIRONA: Sustainable Integration of Regenerative Outer-space Nature and Agriculture. bigidea.nianet.org
BF Sloane, Y Xu, JI Kyungmin, H Tu, ... (2017) Cell culture devices for biomimetic and pathomimetic cell cultures. US Patent App. 15 …, Google Patents
E Obert (2013) The bioinspired design process: according to project complexity and motivation., dspace.mit.edu, cited by 1 (0.17 per year)

YJ Choi, HG Yi, SW Kim, DW Cho (2017) 3D cell printed tissue analogues: a new platform for theranostics. Theranostics, ncbi.nlm.nih.gov, cited by 26 (13.00 per year)
A Przybytek, I Gubańska, ... (2018) Polyurethanes as a Potential Medical-Grade Filament for Use in Fused Deposition Modeling 3D Printers–a Brief Review. Fibres & Textiles in …, yadda.icm.edu.pl, cited by 1 (1.00 per year)
F Baino, M Ferraris (2017) Learning from Nature: Using bioinspired approaches and natural materials to make porous bioceramics. International Journal of Applied Ceramic …, Wiley Online Library, cited by 11 (5.50 per year)
김민식 (2018) Fabrication and Control of Bio-mimetic Flapping Ornithopter Using Smart Soft Composite., s-space.snu.ac.kr
T Hussain (2018) Re-fashioning the garment industry: Exploring innovations for a circular economy. Clothing Cultures, ingentaconnect.com
SJ Morris, JPR Dudman, L Körner, P Melo, LH Newton, ... (2016) Complex functional surface design for additive manufacturing., eprints.nottingham.ac.uk
E Marsillac, S Hudson (2019) 22 Sustainability in logistics. Handbook on the Sustainable Supply …, books.google.com
R Matta, AL Gonzalez (2019) Engineered Biomimetic Neural Stem Cell Niche. Current Stem Cell Reports, Springer
A Aksamija (2017) Integrating Innovation in Architecture: Design, Methods and Technology for Progressive Practice and Research., books.google.com, cited by 14 (7.00 per year)
G Parry (2016) Smooth Seas Do Not Make Good Sailors. How Should Humanity Steer the Future?, Springer, cited by 1 (0.33 per year)
K Lindström, V Zurapovic (2018) Myoelectric Prosthetic Hand., diva-portal.org
R Tan, X Yang, Y Shen (2017) Robot-aided electrospinning toward intelligent biomedical engineering. Robotics and biomimetics, biomedcentral.com, cited by 3 (1.50 per year)
A Meyer, I Fourie (2018) Information behaviour of architecture students in creative design projects. Aslib Journal of Information Management, emeraldinsight.com
N Sears, P Dhavalikar, M Whitely, ... (2017) Fabrication of biomimetic bone grafts with multi-material 3D printing. …, iopscience.iop.org, cited by 13 (6.50 per year)
Y Ding, MA Invernale, GA Sotzing (2010) Conductivity trends of PEDOT-PSS impregnated fabric and the effect of conductivity on electrochromic textile. ACS applied materials & …, ACS Publications, cited by 129 (14.33 per year)
RK Katzschmann (2018) Building and controlling fluidically actuated soft robots: from open loop to model-based control., dspace.mit.edu
K McIntyre, JA Ortiz (2016) Multinational corporations and the circular economy: how Hewlett Packard scales innovation and technology in its global supply chain. Taking Stock of Industrial Ecology, oapen.org, cited by 9 (3.00 per year)
S Knowlton, S Onal, CH Yu, JJ Zhao, S Tasoglu (2015) Bioprinting for cancer research. Trends in biotechnology, Elsevier, cited by 130 (32.50 per year)
J You, RJ Preen, L Bull, J Greenman, ... (2017) 3D printed components of microbial fuel cells: Towards monolithic microbial fuel cell fabrication using additive layer manufacturing. … Energy Technologies and …, Elsevier, cited by 14 (7.00 per year)
G Bridges, M Raach, MF Stoelen (2017) Jellyfish inspired soft robot prototype which uses circumferential contraction for jet propulsion. Conference on Biomimetic and …, Springer
R De Angelis (2018) Sustainable Development, Corporate Sustainability and the Circular Economy. Business Models in the Circular Economy, Springer
CS Ong, X Zhou, CY Huang, T Fukunishi, ... (2017) Tissue engineered vascular grafts: current state of the field. Expert review of …, Taylor & Francis, cited by 16 (8.00 per year)

JC Culver, JC Hoffmann, RA Poché, ... (2012) Three-dimensional biomimetic patterning in hydrogels to guide cellular organization. Advanced …, Wiley Online Library, cited by 139 (19.86 per year)
W Peng, D Unutmaz, IT Ozbolat (2016) Bioprinting towards physiologically relevant tissue models for pharmaceutics. Trends in Biotechnology, Elsevier, cited by 69 (23.00 per year)
N Foster, D Sebastia-Saez, ... (2019) Fractal branch-like fractal shell-and-tube heat exchangers: A CFD study of the shell side performance. IFAC …, epubs.surrey.ac.uk
CA Nobre, G Sampaio, LS Borma, ... (2016) Land-use and climate change risks in the Amazon and the need of a novel sustainable development paradigm. Proceedings of the …, National Acad Sciences, cited by 106 (35.33 per year)
ЛО Щербань, ЮО Костогриз, ВВ Керсновська, ... (2018) Інноваційні технології дизайн-проектування сучасного одягу. Технології та …, er.knutd.edu.ua
DS Martin (2017) An Investigation of Avian Wing Tip Vortex Generation Using a Biomimetic Approach., digitalcommons.calpoly.edu
BC Simionescu, D Ivanov (2016) Natural and synthetic polymers for designing composite materials. Handbook of Bioceramics and Biocomposites, Springer, cited by 4 (1.33 per year)
JY Kim, DA Fluri, JM Kelm, ... (2015) 96-well format-based microfluidic platform for parallel interconnection of multiple multicellular spheroids. Journal of laboratory …, journals.sagepub.com, cited by 28 (7.00 per year)
IF Cengiz, M Pitikakis, L Cesario, P Parascandolo, ... (2016) Building the basis for patient-specific meniscal scaffolds: from human knee MRI to fabrication of 3D printed scaffolds. Bioprinting, Elsevier, cited by 28 (9.33 per year)
P Datta, A Barui, Y Wu, V Ozbolat, KK Moncal, ... (2018) Essential steps in bioprinting: From pre-to post-bioprinting. Biotechnology …, Elsevier, cited by 11 (11.00 per year)
SB Debord, LA Lyon (2003) Influence of particle volume fraction on packing in responsive hydrogel colloidal crystals. The Journal of Physical Chemistry B, ACS Publications, cited by 120 (7.50 per year)
A Azeem, A English, P Kumar, A Satyam, M Biggs, ... (2015) The influence of anisotropic nano- to micro-topography on in vitro and in vivo osteogenesis. …, Future Medicine, cited by 29 (7.25 per year)
K Chaloupka, M Motwani, ... (2011) Development of a new lacrimal drainage conduit using POSS nanocomposite. … and applied biochemistry, Wiley Online Library, cited by 58 (7.25 per year)
H Sharma, S Verma (2018) Regenerative periodontics in restoring oral functions: A power to regenerate what's lost. SRM Journal of Research in Dental Sciences, srmjrds.in
E MacArthur (2019) The virtuous circle., European Investment Bank
NF Hasselmann, W Horn (2018) Attachment of microstructures to single bacteria by two-photon patterning of a protein based hydrogel. Biomedical Physics & Engineering …, iopscience.iop.org
D Wu, JN Wang, LG Niu, XL Zhang, ... (2014) Bioinspired Fabrication of High-Quality 3D Artificial Compound Eyes by Voxel-Modulation Femtosecond Laser Writing for Distortion-Free Wide-Field-of-View …. Advanced Optical …, Wiley Online Library, cited by 56 (11.20 per year)
SMM Valashani, F Barthelat (2015) A laser-engraved glass duplicating the structure, mechanics and performance of natural nacre. Bioinspiration & biomimetics, iopscience.iop.org, cited by 57 (14.25 per year)

B Fischer, A Schulz, MM Gepp, J Neubauer, ... (2016) 3D printing of hydrogels in a temperature controlled environment with high spatial resolution. Current Directions in …, degruyter.com, cited by 3 (1.00 per year)
DO Visscher, E Farré-Guasch, MN Helder, S Gibbs, ... (2016) Advances in bioprinting technologies for craniofacial reconstruction. Trends in …, Elsevier, cited by 35 (11.67 per year)
E Kroski (2018) 63 Ready-to-use Maker Projects., books.google.com
TS Ramasamy, ALC Ong, W Cui (2018) Impact of Three-Dimentional Culture Systems on Hepatic Differentiation of Puripotent Stem Cells and Beyond. Novel Biomaterials for Regenerative …, Springer
J Russo, A Frankic, CM Lindsey (2019) Systems and methods for providing a water supply through in-situ water collection. US Patent App. 16/164,700, Google Patents
C Vyas, G Poologasundarampillai, J Hoyland, ... (2017) University of Manchester, Manchester, United Kingdom., researchgate.net
US Universities, AR List (2015) Appendix C University Nanotechnology Research and Educational Centers. Michael Tomczyk, Wiley Online Library
S Ford, M Despeisse (2015) Can additive manufacturing be a driving force for sustainable industrial systems?. J. Clean. Prod, researchgate.net, cited by 5 (1.25 per year)
F Imad (2014) Green Relationship., scholarscompass.vcu.edu
KP Fuller, D Gaspar, LM Delgado, A Pandit, ... (2016) Influence of porosity and pore shape on structural, mechanical and biological properties of poly ε-caprolactone electro-spun fibrous scaffolds. …, Future Medicine, cited by 20 (6.67 per year)
E Sayed (2019) 18 Offsite manufacturing innovation in a biomimetic future. Offsite Production and Manufacturing for Innovative …, books.google.com
CS Ong, P Yesantharao, CY Huang, G Mattson, ... (2018) 3D bioprinting using stem cells. Pediatric …, nature.com, cited by 20 (20.00 per year)
F Perona SKINNING FUTURE TEXTILES through living. francescaperona.com
Y Sun, Z Guo (2019) Recent advances of bioinspired functional materials with specific wettability: from nature and beyond nature. Nanoscale Horizons, pubs.rsc.org, cited by 5 (5.00 per year)
GD Pins, KA Bush, LP Cunningham, ... (2006) Multiphoton excited fabricated nano and micro patterned extracellular matrix proteins direct cellular morphology. … Research Part A: An …, Wiley Online Library, cited by 38 (2.92 per year)
S Gong, W Cui, Q Zhang, A Cao, L Jiang, Q Cheng (2015) Integrated Ternary Bioinspired Nanocomposites via Synergistic Toughening of Reduced Graphene Oxide and Double-Walled Carbon Nanotubes. ACS nano, ACS Publications, cited by 73 (18.25 per year)
SM , ... (2017) STEM., digitalcommons.georgiasouthern …
MR Cutkosky, S Kim (2009) Design and fabrication of multi-material structures for bioinspired robots. Philosophical Transactions of the …, royalsocietypublishing.org, cited by 91 (9.10 per year)
R Hamilton, SL Godding, SA Gray (2018) Mechanical Stimulation of Engineered Muscle., digitalcommons.wpi.edu
RF Pereira, PJ Bártolo (2015) 3D photo-fabrication for tissue engineering and drug delivery. Engineering, Elsevier, cited by 39 (9.75 per year)
C Weetman (2016) A circular economy handbook for business and supply chains: Repair, remake, redesign, rethink., books.google.com, cited by 35 (11.67 per year)

S Rahimifard, J Stone, P Lumsakul, H Trollman (2018) Net Positive Manufacturing: A Restoring, Self-healing and Regenerative Approach to Future Industrial Development. Procedia Manufacturing, Elsevier, cited by 1 (1.00 per year)
M Guo, DV Gealy, J Liang, J Mahler, ... (2017) Design of parallel-jaw gripper tip surfaces for robust grasping. … on Robotics and …, ieeexplore.ieee.org, cited by 12 (6.00 per year)
B Hsiung (2017) Structural colorants that do not exhibit iridescence. US Patent App. 15/632,421, Google Patents
GX Gu, S Wettermark, MJ Buehler (2017) Algorithm-driven design of fracture resistant composite materials realized through additive manufacturing. Additive Manufacturing, Elsevier, cited by 18 (9.00 per year)
H King, S Ocko, L Mahadevan (2015) Termite mounds harness diurnal temperature oscillations for ventilation. Proceedings of the …, National Acad Sciences, cited by 39 (9.75 per year)
Y Zhao, X Zhao, B Tang, W Xu, J Li, ... (2010) Quantum-dot-tagged bioresponsive hydrogel suspension array for multiplex label-free DNA detection. Advanced Functional …, Wiley Online Library, cited by 132 (14.67 per year)
I Cavero, JM Guillon, HH Holzgrefe (2019) Human organotypic bioconstructs from organ-on-chip devices for human-predictive biological insights on drug candidates. Expert opinion on drug safety, Taylor & Francis
MJ Ambrosi (2016) Current energy storage technologies, developments and the future., search.proquest.com
L Yuan, S Ding, C Wen (2019) Additive manufacturing technology for porous metal implant applications and triple minimal surface structures: A review. Bioactive Materials, Elsevier, cited by 8 (8.00 per year)
A Jansen, KS Luck, J Campbell, HB Amor, ... (2017) Bio-inspired Robot Design Considering Load-Bearing and Kinematic Ontogeny of Chelonioidea Sea Turtles. … on Biomimetic and …, Springer
R Barua, S Datta, P Datta, ... (2019) Scaffolds and Tissue Engineering Applications by 3D Bio-Printing Process: A New Approach. Design, Development, and …, igi-global.com
C Karamini (2006) Biopolymer matrix enhances bone regeneration in rat critical size skull defects., search.proquest.com
HD Lynh, C Pin-Chuan (2018) Novel solvent bonding method for creation of a three-dimensional, non-planar, hybrid PLA/PMMA microfluidic chip. Sensors and Actuators A: Physical, Elsevier, cited by 2 (2.00 per year)
Y Xiao, S Ahadian, M Radisic (2017) Biochemical and biophysical cues in matrix design for chronic and diabetic wound treatment. Tissue Engineering Part B: Reviews, liebertpub.com, cited by 12 (6.00 per year)
C Cvetkovic, R Raman, V Chan, ... (2014) Three-dimensionally printed biological machines powered by skeletal muscle. Proceedings of the …, National Acad Sciences, cited by 184 (36.80 per year)
IO Smith, XH Liu, LA Smith, ... (2009) Nanostructured polymer scaffolds for tissue engineering and regenerative medicine. Wiley Interdisciplinary …, Wiley Online Library, cited by 242 (24.20 per year)
N Bitterman, K Klimovich, G Pillar (2019) Home healthcare devices; Challenge of CPAP design for effective home treatment. The Design Journal, Taylor & Francis
AG Domel, M Saadat, JC Weaver, ... (2018) Shark skin-inspired designs that improve aerodynamic performance. Journal of the …, royalsocietypublishing.org, cited by 10 (10.00 per year)

L Li, J Zhang, Z Peng, Y Li, C Gao, Y Ji, R Ye, ... (2016) High-performance pseudocapacitive microsupercapacitors from laser-induced graphene. Advanced …, Wiley Online Library, cited by 147 (49.00 per year)
C Haddal, DL Bull, PM Hernandez, EJK Keller, ... (2018) Global Security Implications of Chemical and Biological Innovation.., osti.gov
ÇK PAZARBAŞI, ÖÜP ÖZEMİR, AGE ERCİŞ (2019) KNOWLEDGE SHARING BETWEEN DESIGNERS AND CRAFTSPEOPLE IN TURKEY: DEVELOPMENT OF A PLATFORM. MILLI FOLKLOR, millifolklor.com
Ç KAYA PAZARBAŞI, ÜP ÖZEMİR, E ERCİŞ (2019) KNOWLEDGE SHARING BETWEEN DESIGNERS AND CRAFTSPEOPLE IN TURKEY: DEVELOPMENT OF A PLATFORM.. Milli Folklor, search.ebscohost.com
D Yang, J Xiao, B Wang, L Li, X Kong, J Liao (2019) The immune reaction and degradation fate of scaffold in cartilage/bone tissue engineering. Materials Science and …, Elsevier
N Margolis (2017) Hygromorphic Scales for Use in Water from Morning Dew and Elementary Model of Hydrogel Expansion Properties., scholar.colorado.edu
S Abdollahi, J Boktor, N Hibino (2019) Bioprinting of freestanding vascular grafts and the regulatory considerations for additively manufactured vascular prostheses. Translational Research, Elsevier, cited by 2 (2.00 per year)
AD Lantada (2013) Biofabrication: Main Advances and Challenges. Handbook on Advanced Design and Manufacturing …, Springer
R Edgeman (2019) Performance Management and Enterprise Excellence through Sustainable Business Models. Sustainable Business Models, Springer, cited by 1 (1.00 per year)
S Dobson (2015) Rammed earth in the modern world. … of the First International Conference on …, content.taylorfrancis.com, cited by 7 (1.75 per year)
G Scott (2019) Preparing work ready plus graduates for an uncertain future. Education for Employability (Volume 1), brill.com, cited by 1 (1.00 per year)
NM León-Lugo, SA Torres-Vázquez, ... (2018) Caracterización de compositos biocerámicos en esponjas de titanio mediante interpenetración por corriente pulsada asistida. Matéria (Rio de …, SciELO Brasil
C Rice, KT Tan (2019) Horse hoof inspired biomimetic structure for improved damage tolerance and crack diversion. Composite Structures, Elsevier
N Gerszberg, K Mortensen, A Saravanan, L Stambler, ... (2018) Electronic Ornithopter Systems: Manual Navigation and Autonomous Hovering in Micro Air Vehicles., soe.rutgers.edu
JW Lee, YJ Choi, WJ Yong, F Pati, JH Shim, ... (2016) Development of a 3D cell printed construct considering angiogenesis for liver tissue engineering. …, iopscience.iop.org, cited by 69 (23.00 per year)
D Olivero (2017) Maker in architettura. Esperimenti di fabbricazione di una Responsive Surface., webthesis.biblio.polito.it
M Mirkhalaf, F Barthelat (2016) Nacre-like materials using a simple doctor blading technique: Fabrication, testing and modeling. Journal of the mechanical behavior of biomedical …, Elsevier, cited by 20 (6.67 per year)
N Vermeulen, G Haddow, T Seymour, ... (2017) 3D bioprint me: a socioethical view of bioprinting human organs and tissues. Journal of Medical …, jme.bmj.com, cited by 21 (10.50 per year)
L Simisic Pasic (2018) Justify Beauty, Architectural Sensorium., tdx.cat

MK ERBAŞ, GR AKTAŞ, S ŞANVER, ... (2017) MR GÖRÜNTÜLERİNDEN BEYİN DAMARININ MODELLENMESİ VE 3 BOYUTLU BASKISI. Karabük Üniversitesi …, 3dprintturkey.org
PS Lee, G Cai, ALS Eh, ... (2017) Electrochromics for Printed Displays and Smart Windows. … for 2D and 3D Printing, John Wiley & Sons, cited by 2 (1.00 per year)
N Wang, PE Phelan, C Harris, J Langevin, ... (2018) Past visions, current trends, and future context: A review of building energy, carbon, and sustainability. … and Sustainable Energy …, Elsevier, cited by 13 (13.00 per year)
M Ferrara, E De Tommasi, G Coppola, ... (2016) Diatom valve three-dimensional representation: a new imaging method based on combined microscopies. International journal of …, mdpi.com, cited by 3 (1.00 per year)
JB Costa, J Silva-Correia, JM Oliveira, ... (2017) Fast Setting Silk Fibroin Bioink for Bioprinting of Patient-Specific Memory-Shape Implants. Advanced healthcare …, Wiley Online Library, cited by 10 (5.00 per year)
X Zhou, NJ Castro, W Zhu, H Cui, M Aliabouzar, ... (2016) Improved human bone marrow mesenchymal stem cell osteogenesis in 3D bioprinted tissue scaffolds with low intensity pulsed ultrasound stimulation. Scientific reports, nature.com, cited by 43 (14.33 per year)
RS Marcucio, L Qin, E Alsberg, ... (2017) Reverse engineering development: crosstalk opportunities between developmental biology and tissue engineering. Journal of Orthopaedic …, Wiley Online Library, cited by 6 (3.00 per year)
F Calignano, T Tommasi, D Manfredi, A Chiolerio (2015) Additive manufacturing of a microbial fuel cell—a detailed study. Scientific reports, nature.com, cited by 25 (6.25 per year)
Y Li, Y Xiao, C Liu (2017) The horizon of materiobiology: a perspective on material-guided cell behaviors and tissue engineering. Chemical reviews, ACS Publications, cited by 90 (45.00 per year)
X Li, X Wang, X Wang, H Chen, X Zhang, L Zhou, T Xu (2018) 3D bioprinted rat Schwann cell-laden structures with shape flexibility and enhanced nerve growth factor expression. 3 Biotech, Springer
C Rhodes (2014) PLANT-BIOMIMETIC HYDROGEL ACTUATORS: CREATING MOVEMENT IN HYDROGELS THROUGH THE STUDY OF PLANTS., scholarcommons.sc.edu
CM Smith (2016) An adaptive paradigm for human space settlement. Acta Astronautica, Elsevier, cited by 3 (1.00 per year)
L Jiang, Y Zhao, J Zhai (2004) A lotus-leaf-like superhydrophobic surface: a porous microsphere/nanofiber composite film prepared by electrohydrodynamics. Angewandte Chemie International …, Wiley Online Library, cited by 1053 (70.20 per year)
L Ribas, A Rangel, M Verdicchio, M Carvalhais (2018) xCoAx 2018 Special Issue., aisberg.unibg.it
D Neutopia Universal Income, Women's Liberation, and Neutopian Thought. basicincome.org
O Al-Ketan, A Soliman, AM AlQubaisi, ... (2018) Nature-Inspired Lightweight Cellular Co-Continuous Composites with Architected Periodic Gyroidal Structures. Advanced …, Wiley Online Library, cited by 15 (15.00 per year)
J Sun, D Wei, K Yang, Y Yang, X Liu, H Fan, ... (2017) The development of cell-initiated degradable hydrogel based on methacrylated alginate applicable to multiple microfabrication technologies. Journal of Materials …, pubs.rsc.org, cited by 7 (3.50 per year)
J Dinoro, M Maher, S Talebian, M Jarfarkhani, ... (2019) Sulfated polysaccharide-based scaffolds for orthopaedic tissue engineering. Biomaterials, Elsevier

M Tavakoli, C Viegas (2015) Bio-inspired climbing robots. Biomimetic Technologies, Elsevier, cited by 1 (0.25 per year)
C Wall, L Pawloski (2014) The Maker Cookbook: Recipes for Children's and'tween Library Programs., books.google.com, cited by 3 (0.60 per year)
S Krishtul, L Baruch, M Machluf (2019) Processed Tissue–Derived Extracellular Matrices: Tailored Platforms Empowering Diverse Therapeutic Applications. Advanced Functional …, Wiley Online Library
A Dolcimascolo, G Calabrese, S Conoci, ... (2019) Innovative Biomaterials for Tissue Engineering. Biomaterial-supported …, intechopen.com
D Kisailus, P Zavattieri (2015) Uncovering and Validating Toughening Mechanisms in High Performance Composites., apps.dtic.mil
S Habib (2018) Co-Creation: a model for Collaboration+ Creativity., drum.lib.umd.edu
SI Abdul Kudus, RI Campbell, RJ Bibb (2016) Assessing the value of 3D printed personalised products., dspace.lboro.ac.uk
D Wolpert WorldWide ElectroActive Polymers. Citeseer
B Kennedy, C Melhuish, ... (2001) Biologically inspired robots. … Polymer (EAP) Actuators …, spiedigitallibrary.org, cited by 13 (0.72 per year)
MJ Harrington, F Jehle, T Priemel (2018) Mussel Byssus Structure-Function and Fabrication as Inspiration for Biotechnological Production of Advanced Materials. Biotechnology journal, Wiley Online Library, cited by 3 (3.00 per year)
F Bianconi, M Filippucci (2019) WOOD, CAD AND AI: Digital Modelling as Place of Convergence of Natural and Artificial Intelligent to Design. Digital Wood Design: Innovative …, books.google.com
T Kamps, M Biedermann, C Seidel, G Reinhart (2018) Design approach for additive manufacturing employing Constructal Theory for point-to-circle flows. Additive Manufacturing, Elsevier, cited by 12 (12.00 per year)
A Székács (2017) Environmental and ecological aspects in the overall assessment of bioeconomy. Journal of Agricultural and Environmental Ethics, Springer, cited by 16 (8.00 per year)
G SCOTT (2019) 9. PREPARING WORK READY PLUS GRADUATES FOR AN UNCERTAIN FUTURE. Education for Employability (Volume 1): The …, books.google.com
I Chiesa, GM Fortunato, A Lapomarda, ... (2019) Ultrasonic mixing chamber as an effective tool for the biofabrication of fully graded scaffolds for interface tissue engineering. … journal of artificial …, journals.sagepub.com
YH Jung, B Park, JU Kim, T Kim (2018) Bioinspired Electronics for Artificial Sensory Systems. Advanced Materials, Wiley Online Library
S Kim, F Qiu, S Kim, A Ghanbari, C Moon, ... (2013) Fabrication and characterization of magnetic microrobots for three-dimensional cell culture and targeted transportation. Advanced …, Wiley Online Library, cited by 190 (31.67 per year)
MJDE Oliveira, JP Sousa, VC Costa, ... (2017) Musical morphogenesis-a self-organizing system. … -a self-organizing …, repositorio.iscte-iul.pt
PJ Bártolo, CK Chua, HA Almeida, ... (2009) Biomanufacturing for tissue engineering: present and future trends. Virtual and Physical …, Taylor & Francis, cited by 129 (12.90 per year)
B Dhariwala, E Hunt, T Boland (2004) Rapid prototyping of tissue-engineering constructs, using photopolymerizable hydrogels and stereolithography. Tissue engineering, liebertpub.com, cited by 292 (19.47 per year)

Y Li, L Li, ZN Chen, G Gao, R Yao, W Sun (2017) Engineering-derived approaches for iPSC preparation, expansion, differentiation and applications. Biofabrication, iopscience.iop.org, cited by 10 (5.00 per year)
A Tabet, C Wang (2019) Gels without Vapor Pressure: Soft, Nonaqueous, and Solvent-Free Supramolecular Biomaterials for Prospective Parenteral Drug Delivery Applications. Advanced healthcare materials, Wiley Online Library, cited by 3 (3.00 per year)
A Aimar, A Palermo, B Innocenti (2019) The Role of 3D Printing in Medical Applications: A State of the Art. Journal of healthcare engineering, hindawi.com, cited by 3 (3.00 per year)
D Anderson (2016) Future Perspective: Design Process of Perfume Packaging., theseus.fi
R Vullings, M Heleven (2015) Not invented here: cross-industry innovation., books.google.com, cited by 14 (3.50 per year)
NM Wragg, L Burke, SL Wilson (2019) A critical review of current progress in 3D kidney biomanufacturing: advances, challenges, and recommendations. Renal Replacement …, rrtjournal.biomedcentral.com
F Bianconi, M Filippucci (2019) WOOD, CAD AND AI: Digital Modelling as Place of Convergence of Natural and Artificial Intelligent to Design Timber Architecture. Digital Wood Design, Springer
M LUMINI (2018) CHAPTER SEVEN ABOUT BIOMORPHIC EXUBERANCE AND DIGITAL ROCOCO IN DESIGN AND PARAMETRIC CONTEMPORARY ARCHITECTURE MASSIMO …. Following Forms, Following Functions: Practices and …, books.google.com
JG Knowles (2017) Impacts of professional development in integrated STEM education on teacher self-efficacy, outcome expectancy, and STEM career awareness., docs.lib.purdue.edu, cited by 2 (1.00 per year)
오은열, 이성효, 김경일, 박지영, 박미선, ... (2019) 스마트한 QR 코드에 의한 비저장식 데이터 기록 시스템및 비저장식 데이터 제공방법에 관한 연구. 융합정보논문지 (구 …, earticle.net
Y Du, JL Guo, J Wang, AG Mikos, S Zhang (2019) Hierarchically Designed Bone Scaffolds: From Internal Cues to External Stimuli. Biomaterials, Elsevier
R Fuhrer, EK Athanassiou, NA Luechinger, WJ Stark (2009) Crosslinking metal nanoparticles into the polymer backbone of hydrogels enables preparation of soft, magnetic field-driven actuators with muscle-like flexibility. Small, Wiley Online Library, cited by 207 (20.70 per year)
SG MacLean The Info List-Space Suit. theinfolist.com
N Picollet-D'hahan, ME Dolega, L Liguori, ... (2016) A 3D toolbox to enhance physiological relevance of human tissue models. Trends in …, Elsevier, cited by 34 (11.33 per year)
MR Mansor, SM Sapuan (2017) Concurrent Conceptual Design and Materials Selection of Natural Fiber Composite Products., books.google.com, cited by 4 (2.00 per year)
P LORENZO-EIROA (2015) FROM CO DI NG TO REPRESENTATION TO FORMAL AUTONOMY TOMEDIAREPRODUCTION. … : Making, Machines, and Models for Design …, books.google.com
M Lewandowski (2016) Designing the business models for circular economy—Towards the conceptual framework. Sustainability, mdpi.com, cited by 274 (91.33 per year)
AK Rajvanshi (2016) Roadmap for rural India. Current Science, nariphaltan.org, cited by 2 (0.67 per year)
A Galperin, RA Oldinski, SJ Florczyk, ... (2013) Integrated bi-layered scaffold for osteochondral tissue engineering. Advanced …, Wiley Online Library, cited by 57 (9.50 per year)

A Karmakar, U Sahib (2017) Smart Dubai: Accelerating innovation and leapfrogging E-democracy. E-Democracy for Smart Cities, Springer, cited by 3 (1.50 per year)
RF Pereira, PJ Bártolo (2015) 3D Printing., core.ac.uk
G Byrne, D Dimitrov, L Monostori, R Teti, ... (2018) Biologicalisation: Biological transformation in manufacturing. CIRP Journal of …, Elsevier, cited by 11 (11.00 per year)
Q Xing, K Yates, M Tahtinen, E Shearier, ... (2014) Decellularization of fibroblast cell sheets for natural extracellular matrix scaffold preparation. … Engineering Part C …, liebertpub.com, cited by 58 (11.60 per year)
Z Wang, J Zhang, J Xie, C Li, Y Li, ... (2010) Bioinspired water-vapor-responsive organic/inorganic hybrid one-dimensional photonic crystals with tunable full-color stop band. Advanced Functional …, Wiley Online Library, cited by 139 (15.44 per year)
F Klein, B Richter, T Striebel, CM Franz, ... (2011) Two-component polymer scaffolds for controlled three-dimensional cell culture. Advanced …, Wiley Online Library, cited by 235 (29.38 per year)
RF Pereira, PJ Bártolo (2015) 3D bioprinting of photocrosslinkable hydrogel constructs. Journal of Applied Polymer Science, Wiley Online Library, cited by 71 (17.75 per year)
R Raman, B Bhaduri, M Mir, ... (2016) High-resolution projection microstereolithography for patterning of neovasculature. Advanced …, Wiley Online Library, cited by 45 (15.00 per year)
N Hunter, C Lefteri, S Thomas, R Turner They have been generated through discussion and exploration with informed and inspirational educators, students and practitioners.. arts.ac.uk
S Chen, G Schueneman, RB Pipes, ... (2014) Effects of crystal orientation on cellulose nanocrystals–cellulose acetate nanocomposite fibers prepared by dry spinning. …, ACS Publications, cited by 52 (10.40 per year)
JL Liu, HP Lee, VBC Tan (2018) Effects of inter-ply angles on the failure mechanisms in bioinspired helicoidal laminates. Composites Science and Technology, Elsevier
ALS Eh, AWM Tan, X Cheng, S Magdassi, ... (2018) Recent advances in flexible electrochromic devices: prerequisites, challenges, and prospects. Energy …, Wiley Online Library, cited by 23 (23.00 per year)
P Kim (2017) Rationalization of Combinatorial Design in Architecture for Microhousing., rave.ohiolink.edu
Y Loo, A Lakshmanan, M Ni, LL Toh, S Wang, ... (2015) Peptide bioink: self-assembling nanofibrous scaffolds for three-dimensional organotypic cultures. Nano …, ACS Publications, cited by 52 (13.00 per year)
SM Mantooth, BG Munoz-Robles, ... (2019) Dynamic hydrogels from host–guest supramolecular interactions. Macromolecular …, Wiley Online Library, cited by 6 (6.00 per year)
S Sant, DF Coutinho, AK Gaharwar, ... (2017) Self-assembled hydrogel fiber bundles from oppositely charged polyelectrolytes mimic micro-/nanoscale hierarchy of collagen. Advanced Functional …, Wiley Online Library, cited by 9 (4.50 per year)
Y Benkler, H Masum (2008) Collective intelligence: creating a prosperous world at peace., libros.metabiblioteca.org, cited by 87 (7.91 per year)
KMA Shanks (2017) Identification and Development of Novel Optics for Concentrator Photovoltaic Applications., ore.exeter.ac.uk
P Gaudenzi, S Atek, V Cardini, M Eugeni, GG Nisi, ... (2018) Revisiting the configuration of small satellites structures in the framework of 3D additive manufacturing. Acta Astronautica, Elsevier, cited by 4 (4.00 per year)

CS Ong, T Fukunishi, H Zhang, CY Huang, A Nashed, ... (2017) Biomaterial-free three-dimensional bioprinting of cardiac tissue using human induced pluripotent stem cell derived cardiomyocytes. Scientific reports, nature.com, cited by 57 (28.50 per year)
DE Hebel, F Heisel (2017) Cultivated Building Materials. Industrialized Natural Resources for …, researchgate.net, cited by 3 (1.50 per year)
A Espinha, G Guidetti, MC Serrano, ... (2016) Shape memory cellulose-based photonic reflectors. … applied materials & …, ACS Publications, cited by 27 (9.00 per year)
E James, A Taylor How change happens and the process of humanitarian innovation. Managing Humanitarian Innovation, developmentbookshelf.com
R Stigter (2016) Suppliers going circular., repository.tudelft.nl, cited by 5 (1.67 per year)
B Duan (2017) State-of-the-art review of 3D bioprinting for cardiovascular tissue engineering. Annals of biomedical engineering, Springer, cited by 99 (49.50 per year)
SV Murphy, A Atala (2016) Regenerative Medicine Technology: On-a-Chip Applications for Disease Modeling, Drug Discovery and Personalized Medicine., taylorfrancis.com, cited by 6 (2.00 per year)
JR Jungck, R Wagner, D Van Loo, B Grossman, ... (2019) Art Forms in Nature: radiolaria from Haeckel and Blaschka to 3D nanotomography, quantitative image analysis, evolution, and contemporary art. Theory in …, Springer
PAK Szabo (2016) A Comprehensive Piezoelectric Bending-Beam Model Inspired by Microaerial Vehicle Applications., tspace.library.utoronto.ca, cited by 1 (0.33 per year)
A Brewer (2017) An Investigation on the use of Processed Hemp Fiber in Digital Fabrication., search.proquest.com
X VENCE, Á PEREIRA ECO-INNOVATION AND BUSINESS MODELS AS DRIVERS FOR CIRCULAR ECONOMY. researchgate.net
ME Hoque, YL Chuan, I Pashby (2012) Extrusion based rapid prototyping technique: an advanced platform for tissue engineering scaffold fabrication. Biopolymers, Wiley Online Library, cited by 100 (14.29 per year)
M Mirkhalaf, B Ashrafi (2017) A numerical study on improving the specific properties of staggered composites by incorporating voids. Materials Today Communications, Elsevier, cited by 5 (2.50 per year)
RU Ahmed, S Banerjee (2016) A predictive model for biomimetic plate type broadband frequency sensor. Bioinspiration, Biomimetics, and …, spiedigitallibrary.org
YT Matsunaga, Y Morimoto, S Takeuchi (2011) Molding cell beads for rapid construction of macroscopic 3D tissue architecture. Advanced materials, Wiley Online Library, cited by 244 (30.50 per year)
A Khademhosseini, Y Ling, JM Karp, ... (2007) Micro-and nanoscale control of cellular environment for tissue engineering. … II. Weinheim: Wiley-VCH, tissueeng.net, cited by 12 (1.00 per year)
LK Chim, AG Mikos (2018) Biomechanical forces in tissue engineered tumor models. Current opinion in biomedical engineering, Elsevier, cited by 3 (3.00 per year)
MR Cutkosky, S Kim (2009) Design and fabrication of multi-material structures., researchgate.net
NC Paxton (2017) Designing patient-specific melt-electrospun scaffolds for bone regeneration., eprints.qut.edu.au
VB Fernández, FAZ Montoya (2017) Creación de una prenda mediante la fabricación digitalizada de superficies impresas en 3D. Iconofacto, revistas.upb.edu.co

O Kontovourkis, G Tryfonos, ... (2019) Robotic additive manufacturing (RAM) with clay using topology optimization principles for toolpath planning: the example of a building element. Architectural Science …, Taylor & Francis
C Archer-Brown, D Johns Accelerating the development process: Bloodhound SSC Steering Wheel Project–where man meets machine.
J Gopinathan, I Noh (2018) Current Status of Development and Intellectual Properties of Biomimetic Medical Materials. Biomimetic Medical Materials, Springer
S Prendeville, E Cherim, N Bocken (2018) Environmental Innovation and Societal Transitions. … Innovation and Societal …, researchgate.net
E Saiz, EA Zimmermann, JS Lee, UGK Wegst, ... (2013) Perspectives on the role of nanotechnology in bone tissue engineering. Dental Materials, Elsevier, cited by 57 (9.50 per year)
X Li, J Chen, B Liu, X Wang, D Ren, T Xu (2018) Inkjet Printing for Biofabrication. 3D Printing and Biofabrication, Springer, cited by 1 (1.00 per year)
TDH Le (2018) Diatom Biosilica: from Biosilicification to Bone Tissue Applications. 2018 4th International Conference on Green …, ieeexplore.ieee.org
A Phillips (2019) Silicon Valley Immersion Program., ideaexchange.uakron.edu
CH Chuang, YY Chiang (2019) Bio-O-Pump: a novel portable microfluidic device driven by osmotic pressure. Sensors and Actuators B: Chemical, Elsevier
M Elbadawi, G Andrikopoulos, ... (2018) Bio-Inspired Climbing Robots in Wet Environments: Recent Trends in Adhesion Methods and Materials. … on Robotics and …, ieeexplore.ieee.org
R Raman, L Grant, Y Seo, C Cvetkovic, ... (2017) Damage, healing, and remodeling in optogenetic skeletal muscle bioactuators. Advanced …, Wiley Online Library, cited by 16 (8.00 per year)
M Costantini, S Testa, P Mozetic, A Barbetta, C Fuoco, ... (2017) Microfluidic-enhanced 3D bioprinting of aligned myoblast-laden hydrogels leads to functionally organized myofibers in vitro and in vivo. Biomaterials, Elsevier, cited by 37 (18.50 per year)
X Zhou, T Esworthy, SJ Lee, S Miao, H Cui, ... (2019) 3D Printed scaffolds with hierarchical biomimetic structure for osteochondral regeneration. … , Biology and Medicine, Elsevier
ZL Wang (2012) Self-powered nanosensors and nanosystems. Advanced Materials, Wiley Online Library, cited by 400 (57.14 per year)
T Saraceno (2017) Interview Between Markus Buehler and Markus Buehler and. Active Matter, MIT Press
SQ Hachey (2018) Prospects for Sustainable Micro-Factory Retailing in Canada: A Case Study of 3D Printed Electric Vehicles., macsphere.mcmaster.ca
SM Halıcı, GD Turhan, MS Aksu, ... (2017) Uzay Mimarlığında Sayısal Tasarım ve Üretim Araçlarının Değerlendirilmesi Üzerine Mars Özelinde Bir Çalışma. MSTAS 2017, mstas2017.metu.edu.tr, cited by 1 (0.50 per year)
AA Alsheghri, O Alageel, MA Mezour, B Sun, S Yue, ... (2018) Bio-inspired and optimized interlocking features for strengthening metal/polymer interfaces in additively manufactured prostheses. Acta biomaterialia, Elsevier
JAS Neiman, R Raman, V Chan, ... (2015) Photopatterning of hydrogel scaffolds coupled to filter materials using stereolithography for perfused 3D culture of hepatocytes. Biotechnology and …, Wiley Online Library, cited by 44 (11.00 per year)
SM Sapuan Muhd Ridzuan Mansor. Springer

A Bruyas, F Lou, AM Stahl, M Gardner, ... (2018) Systematic characterization of 3D-printed PCL/β-TCP scaffolds for biomedical devices and bone tissue engineering: influence of composition and porosity. Journal of materials …, cambridge.org, cited by 7 (7.00 per year)
RD Sochol, NR Gupta, JV Bonventre (2016) A role for 3D printing in kidney-on-a-chip platforms. Current transplantation reports, Springer, cited by 11 (3.67 per year)
IT Ozbolat, M Hospodiuk (2016) Current advances and future perspectives in extrusion-based bioprinting. Biomaterials, Elsevier, cited by 345 (115.00 per year)
DJ Fitzgerald (2015) HydroDog: A Quadruped Robot Actuated by Soft Fluidic Muscles., digitalcommons.wpi.edu, cited by 1 (0.25 per year)
KS Allan, RM Pilliar, J Wang, MD Grynpas, ... (2007) Formation of biphasic constructs containing cartilage with a calcified zone interface. Tissue …, liebertpub.com, cited by 114 (9.50 per year)
P Russell 3-D Printed Earthen Architecture. academia.edu
D D'Uva (2018) Handbook of Research on Form and Morphogenesis in Modern Architectural Contexts., books.google.com, cited by 1 (1.00 per year)
T Zhai, X Lu, Y Ling, M Yu, G Wang, T Liu, ... (2014) A New Benchmark Capacitance for Supercapacitor Anodes by Mixed-Valence Sulfur-Doped V6O13−x. Advanced …, Wiley Online Library, cited by 190 (38.00 per year)
AD Lantada, A de Blas Romero, ... (2017) Monolithic 3D labs-and organs-on-chips obtained by lithography-based ceramic manufacture. … International Journal of …, Springer, cited by 5 (2.50 per year)
L Paxton, D Knuth, S Gribben, E Tunstel (2019) CRICKET: Cryogenic Reservoir Inventory by Cost-Effective Kinetically Enhanced Technology., ntrs.nasa.gov
S Heinonen, J Karjalainen, J Ruotsalainen, ... Neo-Carbon Core Concepts. utu.fi
E Alarçin, X Guan, SS Kashaf, K Elbaradie, ... (2016) Recreating composition, structure, functionalities of tissues at nanoscale for regenerative medicine. Regenerative …, Future Medicine, cited by 5 (1.67 per year)
K Haberstroh, K Ritter, J Kuschnierz, ... (2010) Bone repair by cell-seeded 3D-bioplotted composite scaffolds made of collagen treated tricalciumphosphate or tricalciumphosphate-chitosan-collagen hydrogel or …. … Research Part B …, Wiley Online Library, cited by 54 (6.00 per year)
D Yang, W Luo, Y Huang, S Huang (2019) Facile Synthesis of Monodispersed SiO2@Fe3O4 Core–Shell Colloids for Printing and Three-Dimensional Coating with Noniridescent Structural Colors. ACS Omega, ACS Publications, cited by 1 (1.00 per year)
B Wendel, D Rietzel, F Kühnlein, ... (2008) Additive processing of polymers. Macromolecular …, Wiley Online Library, cited by 196 (17.82 per year)
A Du Plessis, I Yadroitsava, I Yadroitsev, ... (2018) Numerical comparison of lattice unit cell designs for medical implants by additive manufacturing. Virtual and Physical …, Taylor & Francis, cited by 20 (20.00 per year)
R Fabela, A María, R Pedroza Flores Challenges of the professional training of the industrial designer in the Fourth Industrial Revolution (4RI). ri.uaemex.mx
M Esposito, T Tse, K Soufani (2018) Introducing a circular economy: new thinking with new managerial and policy implications. California Management …, journals.sagepub.com, cited by 20 (20.00 per year)
J Jang, HG Yi, DW Cho (2016) 3D printed tissue models: present and future. ACS Biomaterials Science & Engineering, ACS Publications, cited by 45 (15.00 per year)

T Trantidou, A Regoutz, XN Voon, DJ Payne, ... (2018) A "cleanroom-free" and scalable manufacturing technology for the microfluidic generation of lipid-stabilized droplets and cell-sized multisomes. Sensors and Actuators B …, Elsevier, cited by 7 (7.00 per year)
R Yuan, J Lee, HW Su, E Levy, ... (2018) Microfluidics in structured multimaterial fibers. Proceedings of the …, National Acad Sciences, cited by 4 (4.00 per year)
S Bourgeois-Bougrine, S Latorre, F Mourey (2018) Promoting creative imagination of non-expressed needs: exploring a combined approach to enhance design thinking. Creativity Studies, journals.vgtu.lt
E Katsanevakis, B Whatley, V Beachley, ... (2013) Mineralized nanofibril arrays as building units for tissue engineering of long bones. … of Biomaterials and …, ingentaconnect.com, cited by 3 (0.50 per year)
KA Alberti (2015) Bioskiving: Tendon-derived Scaffolds for Biomedical Applications., search.proquest.com
AJ Enyedy, FA Sanchez (2019) Swarm Scaffolding MQP., digitalcommons.wpi.edu
KJ De France, KG Yager, KJW Chan, B Corbett, ... (2017) Injectable anisotropic nanocomposite hydrogels direct in situ growth and alignment of myotubes. Nano …, ACS Publications, cited by 19 (9.50 per year)
M Vaezi, G Zhong, H Kalami, S Yang (2018) Extrusion-based 3D printing technologies for 3D scaffold engineering. Functional 3D Tissue Engineering …, Elsevier, cited by 3 (3.00 per year)
M Jamal, SS Kadam, R Xiao, F Jivan, ... (2013) Bio-origami hydrogel scaffolds composed of photocrosslinked PEG bilayers. Advanced …, Wiley Online Library, cited by 90 (15.00 per year)
O Borgue, M Panarotto, ... (2018) Impact on design when introducing additive manufacturing in space applications. 15th International …, pdfs.semanticscholar.org, cited by 1 (1.00 per year)
SH Sadati, L Sullivan, ID Walker, K Althoefer, ... 3D-printable thermoactive helical interface with decentralized morphological stiffness control for continuum manipulators. spiral.imperial.ac.uk
L Neely, J Gaiennie, N Noble, ... (2016) Stingray-inspired robot with simply actuated intermediate motion. … and Bioreplication 2016, spiedigitallibrary.org, cited by 1 (0.33 per year)
N Magee (2017) A Practical Approach To Harnessing Small Scale Wind Energy Through Piezoelectric Wind Harvesting., search.proquest.com
B Stamm (2017) Innovation: A necessity, not nicety. Visionary Leadership in a Turbulent World: Thriving …, emeraldinsight.com, cited by 2 (1.00 per year)
J Buffington (2015) Frictionless Markets: The 21st Century Supply Chain., Springer, cited by 4 (1.00 per year)
EE Ureña-Benavides, CL Kitchens (2011) Wide-angle X-ray diffraction of cellulose nanocrystal– alginate nanocomposite fibers. Macromolecules, ACS Publications, cited by 44 (5.50 per year)
J Rosser, DJ Thomas (2018) Bioreactor processes for maturation of 3D bioprinted tissue. 3D Bioprinting for Reconstructive Surgery, Elsevier, cited by 2 (2.00 per year)
S Vaje UČNI NAČRT PREDMETA/COURSE SYLLABUS. Environmental Protection, um.si
S DIAMOND (2015) The Fabric of Memory. The Handbook of Textile Culture, books.google.com
A Sensini, L Cristofolini (2018) Biofabrication of electrospun scaffolds for the regeneration of tendons and ligaments. Materials, mdpi.com, cited by 6 (6.00 per year)

W Xie (2017) Design and Validation of an In Vivo Long-Term Attachment Capsule Robot., digitalcommons.unl.edu
V Kapsali (2015) Northumbria University, Newcastle upon Tyne, UK. Textiles for Sportswear, books.google.com
AG Leal-Junior, A Theodosiou, R Min, ... (2019) Quasi-Distributed Torque and Displacement Sensing on a Series Elastic Actuator's Spring Using FBG Arrays Inscribed in CYTOP Fibers. IEEE Sensors …, ieeexplore.ieee.org, cited by 2 (2.00 per year)
L Svendsen, S Tang (2018) Circular economy in the construction industry., projekter.aau.dk
O Zincir, AÖ Tunç (2017) An Imagination of Organizations in the Future: Rethinking McKinsey's 7S Model. Strategic Imperatives and Core Competencies in …, igi-global.com, cited by 1 (0.50 per year)
宋晓艳, 邢金峰 (2015) 双光子聚合 3D 打印. 化工学报, hgxb.com.cn, cited by 2 (0.50 per year)
N Tandon, A Marsano, R Maidhof, ... (2011) Optimization of electrical stimulation parameters for cardiac tissue engineering. Journal of tissue …, Wiley Online Library, cited by 105 (13.13 per year)
M Kitsara, D Kontziampasis, O Agbulut, ... (2018) Heart on a chip: micro-nanofabrication and microfluidics steering the future of cardiac tissue engineering. Microelectronic …, Elsevier, cited by 3 (3.00 per year)
SM Halıcı, GD Turhan, MS Aksu, G Varinlioğlu Digital Design and Fabrication Tools in Space Architecture Workshop. mstas2017.metu.edu.tr
К Гедда-Мудров (2014) Рассматривая землю как общее благо: ключевые движущие силы для инноваций и конкуренции в бизнесе. Вестник УрФУ. Серия: Экономика и управление …, elar.urfu.ru
A Nebuloni, A Rossi (2017) Codice e progetto., researchgate.net
M Rossi, G Buratti (2017) Disegno e complessità. Verso nuovo scenari di disegno di progetto.., re.public.polimi.it
АВ Моргун, ВВ Салмин, ЮА Успенская, ... (2016) Микрофлюидные технологии в изучении и моделировании гематоэнцефалического барьера. …, cyberleninka.ru
M Laranjeira, JF Marar (2014) CAOS & COMPLEXIDADE: DESIGN DE SUPERFÍCIE E OS NOVOS PARADIGMAS DA CIÊNCIA. Educação Gráfica, pdf.blucher.com.br, cited by 1 (0.20 per year)
M Kynast, M Eichmann, G Witt (2019) Rapid. Tech+ FabCon 3. D International Hub for Additive Manufacturing: Exhibition+ Conference+ Networking: Proceedings of the 16th Rapid. Tech …., Carl Hanser Verlag GmbH Co KG
K Takala (2013) Supermaterials. Inspiring sustainable materials for the concept elevator car of the 2020.., aaltodoc.aalto.fi
D Ewinger, A Ternès, J Koerbel, I Towers (2016) Arbeitswelt im Zeitalter der Individualisierung: Trends: Multigrafie und Multi-Option in der Generation Y., books.google.com, cited by 12 (4.00 per year)
O Malmerberg (2014) Att spegla naturen., stud.epsilon.slu.se

bionics AND 3D-printing
Publish or Perish 7.10.2373.7118
Windows (x64) edition, running on Windows 10.0.16299 (x64)
Search terms
Keywords: bionics AND 3D-printing
Years: all

Data retrieval
Data source: Google Scholar
Search date: 2019-07-09 10:12:19 +1200
Cache date: 2019-07-09 10:26:31 +1200
Search result: [0] No error

Metrics
Reference date: 2019-07-09 10:26:31 +1200
Publication years: 1994-2019
Citation years: 25 (1994-2019)
Papers: 999
Citations: 26013
Citations/year: 1040.52
Citations/paper: 26.04 (acc1=479, acc2=391, acc5=252, acc10=154, acc20=78)
Authors/paper: 3.52/4.0/4 (mean/median/mode)
Age-weighed citation rate: 6662.41 (sqrt=81.62), 2041.87/author
Hirsch h-index: 80 (a=4.06, m=3.20, 19865 cites=76.4% coverage)
Egghe g-index: 152 (g/h=1.90, 23152 cites=89.0% coverage)
PoP hI,norm: 42
PoP hI,annual: 1.68

Results
MS Mannoor, Z Jiang, T James, YL Kong, ... (2013) 3D printed bionic ears. Nano …, ACS Publications, cited by 509 (84.83 per year)
A Grzesiak, R Becker, A Verl (2011) The bionic handling assistant: a success story of additive manufacturing. Assembly Automation, emeraldinsight.com, cited by 109 (13.63 per year)
W Zhu, X Ma, M Gou, D Mei, K Zhang, ... (2016) 3D printing of functional biomaterials for tissue engineering. Current opinion in …, Elsevier, cited by 153 (51.00 per year)
BC Gross, JL Erkal, SY Lockwood, C Chen, ... (2014) Evaluation of 3D printing and its potential impact on biotechnology and the chemical sciences., ACS Publications, cited by 908 (181.60 per year)
H Dodziuk (2016) Applications of 3D printing in healthcare. … i torakochirurgia polska= Polish journal of cardio …, ncbi.nlm.nih.gov, cited by 60 (20.00 per year)
YL Kong, MK Gupta, BN Johnson, MC McAlpine (2016) 3D printed bionic nanodevices. Nano Today, Elsevier, cited by 45 (15.00 per year)
E MacDonald, R Wicker (2016) Multiprocess 3D printing for increasing component functionality. Science, science.sciencemag.org, cited by 187 (62.33 per year)
X Wang, M Jiang, Z Zhou, J Gou, D Hui (2017) 3D printing of polymer matrix composites: A review and prospective. Composites Part B: Engineering, Elsevier, cited by 423 (211.50 per year)

X Pei, B Zhang, Y Fan, X Zhu, Y Sun, Q Wang, X Zhang, ... (2017) Bionic mechanical design of titanium bone tissue implants and 3D printing manufacture. Materials Letters, Elsevier, cited by 14 (7.00 per year)
J Prikryl, F Foret (2014) Fluorescence detector for capillary separations fabricated by 3D printing. Analytical chemistry, ACS Publications, cited by 32 (6.40 per year)
K Sun, TS Wei, BY Ahn, JY Seo, SJ Dillon, ... (2013) 3D printing of interdigitated Li-Ion microbattery architectures. Advanced …, Wiley Online Library, cited by 627 (104.50 per year)
C Schubert, MC Van Langeveld, ... (2014) Innovations in 3D printing: a 3D overview from optics to organs. British Journal of …, bjo.bmj.com, cited by 346 (69.20 per year)
J Koprnický, P Najman, J Šafka (2017) 3D printed bionic prosthetic hands. 2017 IEEE International …, ieeexplore.ieee.org, cited by 12 (6.00 per year)
D Radenkovic, A Solouk, A Seifalian (2016) Personalized development of human organs using 3D printing technology. Medical hypotheses, Elsevier, cited by 51 (17.00 per year)
CZ Liu, ZD Xia, ZW Han, PA Hulley, ... (2008) Novel 3D collagen scaffolds fabricated by indirect printing technique for tissue engineering. … Research Part B …, Wiley Online Library, cited by 89 (8.09 per year)
A Anastasiou, C Tsirmpas, A Rompas, ... (2013) 3D printing: Basic concepts mathematics and technologies. 13th IEEE …, ieeexplore.ieee.org, cited by 22 (3.67 per year)
RL Scawn, A Foster, BW Lee, DO Kikkawa, BS Korn (2015) Customised 3D printing: an innovative training tool for the next generation of orbital surgeons. Orbit, Taylor & Francis, cited by 25 (6.25 per year)
G Baronio, S Harran, A Signoroni (2016) A critical analysis of a hand orthosis reverse engineering and 3D printing process. Applied bionics and biomechanics, hindawi.com, cited by 36 (12.00 per year)
Y Zhang, F Zhang, Z Yan, Q Ma, X Li, Y Huang, ... (2017) Printing, folding and assembly methods for forming 3D mesostructures in advanced materials. Nature Reviews …, nature.com, cited by 172 (86.00 per year)
TJ Hinton, A Hudson, K Pusch, A Lee, ... (2016) 3D printing PDMS elastomer in a hydrophilic support bath via freeform reversible embedding. … biomaterials science & …, ACS Publications, cited by 108 (36.00 per year)
W Huang, X Zhang (2014) 3D printing: print the future of ophthalmology. Investigative ophthalmology & visual …, iovs.arvojournals.org, cited by 40 (8.00 per year)
TP Mpofu, C Mawere, M Mukosera (2014) The impact and application of 3D printing technology., academia.edu, cited by 11 (2.20 per year)
Q Gu, J Hao, Y Lu, L Wang, GG Wallace, ... (2015) Three-dimensional bio-printing. Science China Life …, Springer, cited by 41 (10.25 per year)
SZ Guo, K Qiu, F Meng, SH Park, ... (2017) 3D printed stretchable tactile sensors. Advanced …, Wiley Online Library, cited by 82 (41.00 per year)
YH Cha, KH Lee, HJ Ryu, IW Joo, A Seo, ... (2017) Ankle-foot orthosis made by 3D printing technique and automated design software. Applied bionics and …, hindawi.com, cited by 16 (8.00 per year)
Q Yan, H Dong, J Su, J Han, B Song, Q Wei, Y Shi (2018) A review of 3D printing technology for medical applications. Engineering, Elsevier, cited by 15 (15.00 per year)
D Mitsouras, P Liacouras, A Imanzadeh, ... (2015) Medical 3D printing for the radiologist. Radiographics, pubs.rsna.org, cited by 207 (51.75 per year)
MC McAlpine, M Sebastian-Mannoor, YL Kong, ... (2016) Multi-functional hybrid devices/structures using 3D printing. US Patent …, Google Patents, cited by 9 (3.00 per year)

JY Choi, S Das, ND Theodore, I Kim, ... (2015) Advances in 2D/3D printing of functional nanomaterials and their applications. ECS Journal of Solid …, jss.ecsdl.org, cited by 18 (4.50 per year)
RL Truby, JA Lewis (2016) Printing soft matter in three dimensions. Nature, nature.com, cited by 277 (92.33 per year)
PH Warnke, H Seitz, F Warnke, ... (2010) Ceramic scaffolds produced by computer-assisted 3D printing and sintering: Characterization and biocompatibility investigations. … Research Part B …, Wiley Online Library, cited by 148 (16.44 per year)
BC Thompson, E Murray, GG Wallace (2015) Graphite oxide to graphene. Biomaterials to bionics. Advanced Materials, Wiley Online Library, cited by 56 (14.00 per year)
M Kamran, A Saxena (2016) A comprehensive study on 3D printing technology. MIT Int J Mech Eng, researchgate.net, cited by 21 (7.00 per year)
J Zhao, Y Zhang, Y Huang, J Xie, X Zhao, ... (2018) 3D Printing Fiber Electrodes for an All-Fiber Integrated Electronic Device via Hybridization of an Asymmetric Supercapacitor and a Temperature Sensor. Advanced …, Wiley Online Library, cited by 14 (14.00 per year)
JJ Adams, EB Duoss, TF Malkowski, ... (2011) Conformal printing of electrically small antennas on three-dimensional surfaces. Advanced …, Wiley Online Library, cited by 370 (46.25 per year)
Y He, G Xue, J Fu (2014) Fabrication of low cost soft tissue prostheses with the desktop 3D printer. Scientific reports, nature.com, cited by 132 (26.40 per year)
J Ten Kate, G Smit, P Breedveld (2017) 3D-printed upper limb prostheses: a review. Disability and Rehabilitation …, Taylor & Francis, cited by 56 (28.00 per year)
N Zhong, X Zhao (2017) 3D printing for clinical application in otorhinolaryngology. European Archives of Oto-Rhino-Laryngology, Springer, cited by 12 (6.00 per year)
X Wei, D Li, W Jiang, Z Gu, X Wang, Z Zhang, Z Sun (2015) 3D printable graphene composite. Scientific reports, nature.com, cited by 156 (39.00 per year)
Y He, FF Yang, HM Zhao, Q Gao, B Xia, JZ Fu (2016) Research on the printability of hydrogels in 3D bioprinting. Scientific reports, nature.com, cited by 159 (53.00 per year)
S Mao, E Dong, M Xu, H Jin, F Li, ... (2013) Design and development of starfish-like robot: Soft bionic platform with multi-motion using SMA actuators. 2013 IEEE International …, ieeexplore.ieee.org, cited by 12 (2.00 per year)
TL Gerstle, AMS Ibrahim, PS Kim, ... (2014) A plastic surgery application in evolution: three-dimensional printing. Plastic and …, journals.lww.com, cited by 132 (26.40 per year)
BN Peele, TJ Wallin, H Zhao, ... (2015) 3D printing antagonistic systems of artificial muscle using projection stereolithography. Bioinspiration & …, iopscience.iop.org, cited by 78 (19.50 per year)
Y He, S Guo, L Shi, S Pan, ... (2014) 3D printing technology-based an amphibious spherical robot. 2014 IEEE International …, ieeexplore.ieee.org, cited by 23 (4.60 per year)
A Goyanes, J Wang, A Buanz, ... (2015) 3D printing of medicines: engineering novel oral devices with unique design and drug release characteristics. Molecular …, ACS Publications, cited by 197 (49.25 per year)
JS Lee, JM Hong, JW Jung, JH Shim, JH Oh, ... (2014) 3D printing of composite tissue with complex shape applied to ear regeneration. Biofabrication, iopscience.iop.org, cited by 201 (40.20 per year)
SE Bakarich, S Beirne, GG Wallace, ... (2013) Extrusion printing of ionic–covalent entanglement hydrogels with high toughness. Journal of Materials …, pubs.rsc.org, cited by 96 (16.00 per year)

S Sharma (2014) 3D-printed prosthetics roll off the presses. Chemical Engineering Process, aiche.org, cited by 8 (1.60 per year)
A Frutiger, JT Muth, DM Vogt, Y Mengüç, ... (2015) Capacitive soft strain sensors via multicore–shell fiber printing. Advanced …, Wiley Online Library, cited by 149 (37.25 per year)
Y Hwang, OH Paydar, RN Candler (2015) 3D printed molds for non-planar PDMS microfluidic channels. Sensors and Actuators A: Physical, Elsevier, cited by 82 (20.50 per year)
LR Hart, S Li, C Sturgess, R Wildman, ... (2016) 3D printing of biocompatible supramolecular polymers and their composites. … applied materials & …, ACS Publications, cited by 54 (18.00 per year)
DH Ballard, AP Trace, S Ali, T Hodgdon, ME Zygmont, ... (2018) Clinical applications of 3D printing: primer for radiologists. Academic radiology, Elsevier, cited by 29 (29.00 per year)
YS Zhang, K Yue, J Aleman, ... (2017) 3D bioprinting for tissue and organ fabrication. Annals of biomedical …, Springer, cited by 156 (78.00 per year)
YL Kong, IA Tamargo, H Kim, BN Johnson, ... (2014) 3D printed quantum dot light-emitting diodes. Nano …, ACS Publications, cited by 211 (42.20 per year)
T Wu, S Yu, D Chen, Y Wang (2017) Bionic design, materials and performance of bone tissue scaffolds. Materials, mdpi.com, cited by 12 (6.00 per year)
SE Hudson (2014) Printing teddy bears: a technique for 3D printing of soft interactive objects. Proceedings of the SIGCHI Conference on Human …, dl.acm.org, cited by 91 (18.20 per year)
CMB Ho, SH Ng, YJ Yoon (2015) A review on 3D printed bioimplants. International Journal of Precision Engineering …, Springer, cited by 50 (12.50 per year)
D Hua, X Zhang, Z Ji, C Yan, B Yu, Y Li, ... (2018) 3D printing of shape changing composites for constructing flexible paper-based photothermal bilayer actuators. Journal of Materials …, pubs.rsc.org, cited by 14 (14.00 per year)
L Wang, J Liu (2014) Compatible hybrid 3D printing of metal and nonmetal inks for direct manufacture of end functional devices. Science China Technological Sciences, Springer, cited by 29 (5.80 per year)
H Seitz, W Rieder, S Irsen, B Leukers, ... (2005) Three-dimensional printing of porous ceramic scaffolds for bone tissue engineering. Journal of Biomedical …, Wiley Online Library, cited by 654 (46.71 per year)
G Zhou, KP Wang, HW Liu, L Wang, XF Xiao, ... (2018) Three-dimensional polylactic acid@ graphene oxide/chitosan sponge bionic filter: highly efficient adsorption of crystal violet dye. International journal of …, Elsevier, cited by 11 (11.00 per year)
GI Peterson, MB Larsen, MA Ganter, ... (2014) 3D-printed mechanochromic materials. … applied materials & …, ACS Publications, cited by 117 (23.40 per year)
JO Hardin, TJ Ober, AD Valentine, ... (2015) Microfluidic printheads for multimaterial 3D printing of viscoelastic inks. Advanced …, Wiley Online Library, cited by 116 (29.00 per year)
S Joshi, E Cook, MS Mannoor (2018) Bacterial Nanobionics via 3D printing. Nano letters, ACS Publications, cited by 4 (4.00 per year)
KK VanKoevering, SJ Hollister, ... (2017) Advances in 3-dimensional printing in otolaryngology: a review. … Otolaryngology–Head & …, jamanetwork.com, cited by 14 (7.00 per year)
SH Park, JH Park, HJ Lee, NK Lee (2014) Current status of biomedical applications using 3D printing technology. Journal of the Korean Society …, koreascience.or.kr, cited by 19 (3.80 per year)

J Shi, L Zhu, L Li, Z Li, J Yang, X Wang (2018) A TPMS-based method for modeling porous scaffolds for bionic bone tissue engineering. Scientific reports, nature.com, cited by 7 (7.00 per year)
F Pati, J Gantelius, HA Svahn (2016) 3D bioprinting of tissue/organ models. … Chemie International Edition, Wiley Online Library, cited by 87 (29.00 per year)
CZ Liu, E Sachlos, DA Wahl, ZW Han, ... (2007) On the manufacturability of scaffold mould using a 3D printing technology. Rapid Prototyping …, emeraldinsight.com, cited by 24 (2.00 per year)
AS Munoz-Abraham, MI Rodriguez-Davalos, ... (2016) 3D printing of organs for transplantation: where are we and where are we heading?. Current Transplantation …, Springer, cited by 13 (4.33 per year)
NW Bartlett, MT Tolley, JTB Overvelde, ... (2015) A 3D-printed, functionally graded soft robot powered by combustion. …, science.sciencemag.org, cited by 368 (92.00 per year)
A Rindfleisch, M O'Hern, ... (2017) The digital revolution, 3D printing, and innovation as data. Journal of Product …, Wiley Online Library, cited by 22 (11.00 per year)
R Miclaus, A Repanovici, N Roman (2017) Biomaterials: polylactic acid and 3D printing processes for orthosis and prosthesis. Materiale Plastice, revmaterialeplastice.ro, cited by 10 (5.00 per year)
E Mikołajewska, M Macko, Ł Ziarnecki, S Stańczak, ... (2014) 3D printing technologies in rehabilitation engineering., repozytorium.ukw.edu.pl, cited by 13 (2.60 per year)
IT Ozbolat, W Peng, V Ozbolat (2016) Application areas of 3D bioprinting. Drug discovery today, Elsevier, cited by 89 (29.67 per year)
N Bharti, S Gonzalez, A Buhler (2015) 3D technology in libraries: Applications for teaching and research. 2015 4th International …, ieeexplore.ieee.org, cited by 10 (2.50 per year)
M Mohammed, J Tatineni, B Cadd, ... (2016) Applications of 3D topography scanning and multi-material additive manufacturing for facial prosthesis development and production. Proceedings of the …, researchgate.net, cited by 84 (28.00 per year)
X Zhang, J Xie, J Chen, Y Okabe, L Pan, M Xu (2017) The beetle elytron plate: a lightweight, high-strength and buffering functional-structural bionic material. Scientific reports, nature.com, cited by 18 (9.00 per year)
SH Jariwala, GS Lewis, ZJ Bushman, ... (2015) 3D printing of personalized artificial bone scaffolds. 3D printing and …, liebertpub.com, cited by 32 (8.00 per year)
CJ Hansen, R Saksena, DB Kolesky, ... (2013) High-Throughput Printing via Microvascular Multinozzle Arrays. Advanced …, Wiley Online Library, cited by 83 (13.83 per year)
JM Korde, M Shaikh, ... (2018) Bionic prototyping of honeycomb patterned polymer composite and its engineering application. … -Plastics Technology and …, Taylor & Francis, cited by 6 (6.00 per year)
Y Xu, X Guo, S Yang, L Li, P Zhang, ... (2018) Construction of bionic tissue engineering cartilage scaffold based on three-dimensional printing and oriented frozen technology. … Research Part A, Wiley Online Library, cited by 4 (4.00 per year)
IB Abdallah, Y Bouteraa, C Rekik (2017) DESIGN AND DEVELOPMENT OF 3D PRINTED MYOELECTRIC ROBOTIC EXOSKELETON FOR HAND REHABILITATION.. International Journal on Smart …, researchgate.net, cited by 59 (29.50 per year)
C Zhong, HY Xie, L Zhou, X Xu, SS Zheng (2016) Human hepatocytes loaded in 3D bioprinting generate mini-liver. Hepatobiliary & Pancreatic …, Elsevier, cited by 18 (6.00 per year)

E Suaste-Gómez, G Rodríguez-Roldán, H Reyes-Cruz, ... (2016) Developing an ear prosthesis fabricated in polyvinylidene fluoride by a 3D printer with sensory intrinsic properties of pressure and temperature. Sensors, mdpi.com, cited by 19 (6.33 per year)
D Weger, D Lowke, C Gehlen (2016) 3D printing of concrete structures using the selective binding method–Effect of concrete technology on contour precision and compressive strength. Proceedings of 11th fib …, researchgate.net, cited by 12 (4.00 per year)
A Mahmoud, M Bennett (2015) Introducing 3-dimensional printing of a human anatomic pathology specimen: potential benefits for undergraduate and postgraduate education and anatomic …. Archives of pathology & …, archivesofpathology.org, cited by 35 (8.75 per year)
S Vijayavenkataraman, J Fuh, W Lu (2017) 3D printing and 3D bioprinting in pediatrics. Bioengineering, mdpi.com, cited by 13 (6.50 per year)
CW Hull (2015) The birth of 3D printing. Research-Technology Management, Taylor & Francis, cited by 19 (4.75 per year)
JS Miller (2014) The billion cell construct: will three-dimensional printing get us there?. PLoS biology, journals.plos.org, cited by 57 (11.40 per year)
B Lu, H Lan, H Liu (2018) Additive manufacturing frontier: 3D printing electronics. Opto-Electronic Advances, oejournal.org, cited by 11 (11.00 per year)
J Glasschroeder, E Prager, ... (2015) Powder-bed-based 3D-printing of function integrated parts. Rapid Prototyping …, emeraldinsight.com, cited by 29 (7.25 per year)
Q Mu, L Wang, CK Dunn, X Kuang, F Duan, ... (2017) Digital light processing 3D printing of conductive complex structures. Additive …, Elsevier, cited by 39 (19.50 per year)
TJ Wallin, J Pikul, RF Shepherd (2018) 3D printing of soft robotic systems. Nature Reviews Materials, nature.com, cited by 45 (45.00 per year)
CS O'Bryan, T Bhattacharjee, S Hart, ... (2017) Self-assembled micro-organogels for 3D printing silicone structures. Science …, advances.sciencemag.org, cited by 52 (26.00 per year)
Y Li, S Chen, X Cai, J Hong, X Wu, Y Xu, ... (2018) Rational design and preparation of hierarchical monoliths through 3D printing for syngas methanation. Journal of Materials …, pubs.rsc.org, cited by 4 (4.00 per year)
M Schaffner, JA Faber, L Pianegonda, PA Rühs, ... (2018) 3D printing of robotic soft actuators with programmable bioinspired architectures. Nature …, nature.com, cited by 32 (32.00 per year)
JP Jung, DB Bhuiyan, BM Ogle (2016) Solid organ fabrication: comparison of decellularization to 3D bioprinting. Biomaterials …, biomaterialsres.biomedcentral.com, cited by 37 (12.33 per year)
S Sayyar, M Bjorninen, S Haimi, ... (2016) UV cross-linkable graphene/poly (trimethylene carbonate) composites for 3D printing of electrically conductive scaffolds. … applied materials & …, ACS Publications, cited by 25 (8.33 per year)
AMS Ibrahim, RR Jose, AN Rabie, ... (2015) Three-dimensional printing in developing countries. … surgery Global open, ncbi.nlm.nih.gov, cited by 32 (8.00 per year)
Y Wang, X Li, C Li, M Yang, Q Wei (2015) Binder droplet impact mechanism on a hydroxyapatite microsphere surface in 3D printing of bone scaffolds. Journal of Materials Science, Springer, cited by 8 (2.00 per year)
C Xue, X Shi, X Fang, H Tao, H Zhu, F Yu, ... (2016) The "pure marriage" between 3D printing and well-ordered nanoarrays by using peald assisted hydrothermal surface engineering. … applied materials & …, ACS Publications, cited by 10 (3.33 per year)

S Ji, M Guvendiren (2017) Recent advances in bioink design for 3D bioprinting of tissues and organs. Frontiers in bioengineering and biotechnology, frontiersin.org, cited by 84 (42.00 per year)
B Wilcox, RJ Mobbs, AM Wu, K Phan (2017) Systematic review of 3D printing in spinal surgery: the current state of play. Journal of Spine Surgery, ncbi.nlm.nih.gov, cited by 25 (12.50 per year)
A Liu, G Xue, M Sun, H Shao, C Ma, Q Gao, Z Gou, ... (2016) 3D printing surgical implants at the clinic: a experimental study on anterior cruciate ligament reconstruction. Scientific reports, nature.com, cited by 52 (17.33 per year)
AJ Bauermeister, A Zuriarrain, ... (2016) Three-dimensional printing in plastic and reconstructive surgery: a systematic review. Annals of plastic …, journals.lww.com, cited by 53 (17.67 per year)
C Ladd, JH So, J Muth, MD Dickey (2013) 3D printing of free standing liquid metal microstructures. Advanced Materials, Wiley Online Library, cited by 414 (69.00 per year)
KJ McHugh, TD Nguyen, AR Linehan, D Yang, ... (2017) Fabrication of fillable microparticles and other complex 3D microstructures. …, science.sciencemag.org, cited by 34 (17.00 per year)
Q Han, Y Qin, Y Zou, C Wang, H Bai, ... (2017) Novel exploration of 3D printed wrist arthroplasty to solve the severe and complicated bone defect of wrist. Rapid Prototyping …, emeraldinsight.com, cited by 8 (4.00 per year)
LS Dimas, GH Bratzel, I Eylon, ... (2013) Tough composites inspired by mineralized natural materials: computation, 3D printing, and testing. Advanced Functional …, Wiley Online Library, cited by 205 (34.17 per year)
W Wu, A DeConinck, JA Lewis (2011) Omnidirectional printing of 3D microvascular networks. Advanced materials, Wiley Online Library, cited by 361 (45.13 per year)
AE Jakus, AL Rutz, RN Shah (2016) Advancing the field of 3D biomaterial printing. Biomedical Materials, iopscience.iop.org, cited by 75 (25.00 per year)
TR Mackie, NJ Patterson, BL Cox, ... (2014) Three-dimensional printing system using dual rotation axes. US Patent …, Google Patents, cited by 33 (6.60 per year)
JF Hornick (2014) 3D printing and the future (or demise) of intellectual property. 3D Printing and Additive Manufacturing, liebertpub.com, cited by 8 (1.60 per year)
M Saari, B Cox, E Richer, PS Krueger, ... (2015) Fiber encapsulation additive manufacturing: An enabling technology for 3D printing of electromechanical devices and robotic components. 3D Printing and …, liebertpub.com, cited by 33 (8.25 per year)
D Hendricks (2016) 3D Printing Is Already Changing Health Care. Harvard Business Review Digital …, digitalmarketing.temple.edu, cited by 6 (2.00 per year)
TJ Hinton, Q Jallerat, RN Palchesko, ... (2015) Three-dimensional printing of complex biological structures by freeform reversible embedding of suspended hydrogels. Science …, advances.sciencemag.org, cited by 372 (93.00 per year)
S Arabnejad, B Johnston, M Tanzer, ... (2017) Fully porous 3D printed titanium femoral stem to reduce stress-shielding following total hip arthroplasty. Journal of Orthopaedic …, Wiley Online Library, cited by 65 (32.50 per year)
S Derakhshanfar, R Mbeleck, K Xu, X Zhang, W Zhong, ... (2018) 3D bioprinting for biomedical devices and tissue engineering: A review of recent trends and advances. Bioactive materials, Elsevier, cited by 61 (61.00 per year)
MT Ross, R Cruz, C Hutchinson, WL Arnott, ... (2018) Aesthetic reconstruction of microtia: a review of current techniques and new 3D printing approaches. Virtual and Physical …, Taylor & Francis, cited by 5 (5.00 per year)

Y Xu, X Wu, X Guo, B Kong, M Zhang, X Qian, S Mi, ... (2017) The boom in 3D-printed sensor technology. Sensors, mdpi.com, cited by 57 (28.50 per year)
B Zhang, K Kowsari, A Serjouei, ML Dunn, ... (2018) Reprocessable thermosets for sustainable three-dimensional printing. Nature communications, nature.com, cited by 23 (23.00 per year)
Y Ni, R Ji, K Long, T Bu, K Chen, ... (2017) A review of 3D-printed sensors. Applied Spectroscopy …, Taylor & Francis, cited by 13 (6.50 per year)
JN Hanson Shepherd, ST Parker, ... (2011) 3D microperiodic hydrogel scaffolds for robust neuronal cultures. Advanced functional …, Wiley Online Library, cited by 175 (21.88 per year)
N Bharti, S Singh (2017) Three-dimensional (3D) printers in libraries: Perspective and preliminary safety analysis. Journal of chemical education, ACS Publications, cited by 18 (9.00 per year)
RD Sochol, E Sweet, CC Glick, SY Wu, C Yang, ... (2018) 3D printed microfluidics and microelectronics. Microelectronic …, Elsevier, cited by 31 (31.00 per year)
M del Junco, Z Okhunov, R Yoon, ... (2015) Development and initial porcine and cadaver experience with three-dimensional printing of endoscopic and laparoscopic equipment. Journal of …, liebertpub.com, cited by 23 (5.75 per year)
SA Morin, Y Shevchenko, J Lessing, ... (2014) Using "Click-e-Bricks" to Make 3D Elastomeric Structures. Advanced …, Wiley Online Library, cited by 51 (10.20 per year)
JA Camisa, V Verma, DO Marler, ... (2014) Additive manufacturing and 3D printing for oil and gas-transformative potential and technology constraints. The Twenty-fourth …, onepetro.org, cited by 9 (1.80 per year)
X Li, Y Wang, Y Zhao, J Liu, S Xiao, K Mao (2017) Multilevel 3D printing implant for reconstructing cervical spine with metastatic papillary thyroid carcinoma. Spine, journals.lww.com, cited by 9 (4.50 per year)
J Koprnicky, J Šafka, M Ackermann (2018) Using of 3D Printing Technology in Low Cost Prosthetics. Materials Science Forum, Trans Tech Publ, cited by 2 (2.00 per year)
D Weger, D Lowke, C Gehlen (2016) 3D printing of concrete structures with calcium silicate based cements using the selective binding method—effects of concrete technology on penetration depth …. Proceedings of Hipermat, researchgate.net, cited by 7 (2.33 per year)
KS Tanaka, N Lightdale-Miric (2016) Advances in 3D-printed pediatric prostheses for upper extremity differences. JBJS, journals.lww.com, cited by 21 (7.00 per year)
C Rocchini, P Cignoni, C Montani, ... (2001) A low cost 3D scanner based on structured light. Computer Graphics …, Wiley Online Library, cited by 353 (19.61 per year)
D Gu (2016) Materials creation adds new dimensions to 3D printing. Science Bulletin, Springer, cited by 13 (4.33 per year)
Y Kim, K Kang, J Jeong, SS Paik, ... (2017) Three-dimensional (3D) printing of mouse primary hepatocytes to generate 3D hepatic structure. Annals of surgical …, synapse.koreamed.org, cited by 11 (5.50 per year)
D Speranza, D Citro, F Padula, B Motyl, ... (2017) Additive manufacturing techniques for the reconstruction of 3D fetal faces. Applied bionics and …, hindawi.com, cited by 6 (3.00 per year)
MR Skorski, JM Esenther, Z Ahmed, ... (2016) The chemical, mechanical, and physical properties of 3D printed materials composed of TiO2-ABS nanocomposites. … and Technology of …, Taylor & Francis, cited by 40 (13.33 per year)

DG Ahn (2016) Direct metal additive manufacturing processes and their sustainable applications for green technology: A review. International Journal of Precision Engineering and …, Springer, cited by 47 (15.67 per year)
DB Kolesky, RL Truby, AS Gladman, ... (2014) 3D bioprinting of vascularized, heterogeneous cell-laden tissue constructs. Advanced …, Wiley Online Library, cited by 951 (190.20 per year)
SH Park, R Su, J Jeong, SZ Guo, K Qiu, ... (2018) 3D printed polymer photodetectors. Advanced …, Wiley Online Library, cited by 8 (8.00 per year)
J Fajardo, A Lemus, E Rohmer (2015) Galileo bionic hand: sEMG activated approaches for a multifunction upper-limb prosthetic. 2015 IEEE Thirty Fifth Central …, ieeexplore.ieee.org, cited by 13 (3.25 per year)
CL Manzanares Palenzuela, F Novotný, ... (2018) 3D-printed graphene/polylactic acid electrodes promise high sensitivity in electroanalysis. Analytical …, ACS Publications, cited by 20 (20.00 per year)
G Ahn, KH Min, C Kim, JS Lee, D Kang, JY Won, ... (2017) Precise stacking of decellularized extracellular matrix based 3D cell-laden constructs by a 3D cell printing system equipped with heating modules. Scientific reports, nature.com, cited by 22 (11.00 per year)
WR Small, M in het Panhuis (2007) Inkjet printing of transparent, electrically conducting single-walled carbon-nanotube composites. Small, Wiley Online Library, cited by 128 (10.67 per year)
N GUO, Z XIONG (2006) Product Form Bionics Design and Reverse Engineering Technique. Packaging Engineering, en.cnki.com.cn, cited by 7 (0.54 per year)
Z Xia, S Jin, K Ye (2018) Tissue and organ 3D bioprinting. SLAS TECHNOLOGY: Translating Life …, journals.sagepub.com, cited by 11 (11.00 per year)
DL Cohen, E Malone, HOD Lipson, ... (2006) Direct freeform fabrication of seeded hydrogels in arbitrary geometries. Tissue engineering, liebertpub.com, cited by 320 (24.62 per year)
Y Hwang, OH Paydar, RN Candler (2015) Pneumatic microfinger with balloon fins for linear motion using 3D printed molds. Sensors and Actuators A: Physical, Elsevier, cited by 17 (4.25 per year)
K Jakab, C Norotte, B Damon, F Marga, ... (2008) Tissue engineering by self-assembly of cells printed into topologically defined structures. … Engineering Part A, liebertpub.com, cited by 283 (25.73 per year)
A Rompas, C Tsirmpas, I Papatheodorou, ... (2013) 3D Printing: Basic Concepts Mathematics and Technologies. International Journal of …, igi-global.com, cited by 2 (0.33 per year)
D Drotman, S Jadhav, M Karimi, ... (2017) 3D printed soft actuators for a legged robot capable of navigating unstructured terrain. … on Robotics and …, ieeexplore.ieee.org, cited by 34 (17.00 per year)
BC Chen, M Zou, GM Liu, JF Song, HX Wang (2018) Experimental study on energy absorption of bionic tubes inspired by bamboo structures under axial crushing. International Journal of …, Elsevier, cited by 6 (6.00 per year)
CJ Park, HW Kim, S Jeong, S Seo, Y Park, ... (2015) Anti-Reflux Ureteral Stent with Polymeric Flap Valve Using Three-Dimensional Printing: An In Vitro Study. Journal of …, liebertpub.com, cited by 19 (4.75 per year)
JF Rusling (2018) Developing Microfluidic Sensing Devices Using 3D Printing. ACS sensors, ACS Publications, cited by 9 (9.00 per year)
M He, Y Zhao, B Wang, Q Xi, J Zhou, Z Liang (2015) 3D printing fabrication of amorphous thermoelectric materials with ultralow thermal conductivity. Small, Wiley Online Library, cited by 31 (7.75 per year)

SE Bakarich, R Gorkin III, M in het Panhuis, ... (2014) Three-dimensional printing fiber reinforced hydrogel composites. … applied materials & …, ACS Publications, cited by 97 (19.40 per year)
JL Simon, S Michna, JA Lewis, ... (2007) In vivo bone response to 3D periodic hydroxyapatite scaffolds assembled by direct ink writing. … Research Part A: An …, Wiley Online Library, cited by 142 (11.83 per year)
Y He, S Guo, L Shi, S Pan, P Guo (2016) Dynamic gait analysis of a multi-functional robot with bionic springy legs. 2016 IEEE International …, ieeexplore.ieee.org, cited by 4 (1.33 per year)
R Attalla, P Selvaganapathy (2014) 3D printing of gels with integrated vascular channels for cell culture using a microfluidic printhead. … on Miniaturized Systems …, pdfs.semanticscholar.org, cited by 3 (0.60 per year)
KK VanKoevering, KM Malloy (2017) Emerging role of three-dimensional printing in simulation in otolaryngology. Otolaryngologic Clinics of North …, oto.theclinics.com, cited by 8 (4.00 per year)
F Yang, F Lin, C Song, C Zhou, Z Jin, ... (2016) Pbench: a benchmark suite for characterizing 3D printing prefabrication. 2016 IEEE International …, ieeexplore.ieee.org, cited by 3 (1.00 per year)
C Wang, Q Zhao, M Wang (2017) Cryogenic 3D printing for producing hierarchical porous and rhBMP-2-loaded Ca-P/PLLA nanocomposite scaffolds for bone tissue engineering. Biofabrication, iopscience.iop.org, cited by 22 (11.00 per year)
MK Gupta, F Meng, BN Johnson, YL Kong, L Tian, ... (2015) 3D printed programmable release capsules. Nano …, ACS Publications, cited by 70 (17.50 per year)
JL Jiménez-Pérez, PV Pincel, A Cruz-Orea, ... (2016) Thermal characterization of a liquid resin for 3D printing using photothermal techniques. Applied Physics A, Springer, cited by 4 (1.33 per year)
M Wszola, J Idaszek, A Berman, A Kosik, ... (2015) Bionic Pancreas and Bionic Organs–how far we are from the success. MEDtube …, researchgate.net, cited by 2 (0.50 per year)
A Ruzza, M Parekh, S Ferrari, G Salvalaio, ... (2015) Preloaded donor corneal lenticules in a new validated 3D printed smart storage glide for Descemet stripping automated endothelial keratoplasty. British Journal of …, bjo.bmj.com, cited by 21 (5.25 per year)
H Ota, M Chao, Y Gao, E Wu, LC Tai, K Chen, ... (2017) 3d printed "earable" smart devices for real-time detection of core body temperature. ACS …, ACS Publications, cited by 16 (8.00 per year)
G Baronio, P Volonghi, A Signoroni (2017) Concept and design of a 3D printed support to assist hand scanning for the realization of customized orthosis. Applied bionics and …, hindawi.com, cited by 4 (2.00 per year)
I Vujaklija, D Farina (2018) 3D printed upper limb prosthetics. Expert review of medical devices, Taylor & Francis, cited by 4 (4.00 per year)
R Attalla, C Ling, P Selvaganapathy (2016) Fabrication and characterization of gels with integrated channels using 3D printing with microfluidic nozzle for tissue engineering applications. Biomedical microdevices, Springer, cited by 38 (12.67 per year)
Z Tao, HJ Ahn, C Lian, KH Lee, CH Lee (2017) Design and optimization of prosthetic foot by using polylactic acid 3D printing. Journal of Mechanical Science …, Springer, cited by 4 (2.00 per year)
D Schmelzeisen, H Koch, C Pastore, T Gries (2018) 4D textiles: hybrid textile structures that can change structural form with time by 3D printing. Narrow and Smart Textiles, Springer, cited by 3 (3.00 per year)

B Zhang, X Pei, C Zhou, Y Fan, Q Jiang, A Ronca, ... (2018) The biomimetic design and 3D printing of customized mechanical properties porous Ti6Al4V scaffold for load-bearing bone reconstruction. Materials & Design, Elsevier, cited by 21 (21.00 per year)
L Lin, H Zhang, Y Yao, A Tong, Q Hu, ... (2007) Application of Image Processing and Finite Element Analysis in Bionic Scaffolds' Design Optimizing and Fabrication. … Conference on Life System …, Springer, cited by 2 (0.17 per year)
JN Fullerton, GCM Frodsham, RM Day (2014) 3D printing for the many, not the few. Nature biotechnology, nature.com, cited by 37 (7.40 per year)
M Nowicki, NJ Castro, R Rao, M Plesniak, ... (2017) Integrating three-dimensional printing and nanotechnology for musculoskeletal regeneration. …, iopscience.iop.org, cited by 10 (5.00 per year)
T Möller, M Amoroso, D Hägg, C Brantsing, ... (2017) In vivo chondrogenesis in 3D bioprinted human cell-laden hydrogel constructs. … surgery global open, ncbi.nlm.nih.gov, cited by 32 (16.00 per year)
Z Liu, J Zhan, M Fard, JL Davy (2017) Acoustic properties of multilayer sound absorbers with a 3D printed micro-perforated panel. Applied Acoustics, Elsevier, cited by 27 (13.50 per year)
H Cho, U Jammalamadaka, K Tappa (2018) Nanogels for pharmaceutical and biomedical applications and their fabrication using 3D printing technologies. Materials, mdpi.com, cited by 5 (5.00 per year)
TWB Kim, OJ Lopez, JP Sharkey, KR Marden, ... (2017) 3D printed liner for treatment of periprosthetic joint infections. Medical hypotheses, Elsevier, cited by 4 (2.00 per year)
SS Rehmani, FY Bhora (2017) Current state of 3D printing in tissue engineering., Future Medicine, cited by 3 (1.50 per year)
T Hodgdon, R Danrad, MJ Patel, SE Smith, ... (2018) Logistics of three-dimensional printing: primer for radiologists. Academic radiology, Elsevier, cited by 8 (8.00 per year)
H Peng, J Briggs, CY Wang, K Guo, J Kider, ... (2018) RoMA: Interactive fabrication with augmented reality and a robotic 3D printer. Proceedings of the …, dl.acm.org, cited by 19 (19.00 per year)
RK Kankala, K Zhu, J Li, CS Wang, SB Wang, ... (2017) Fabrication of arbitrary 3D components in cardiac surgery: from macro-, micro-to nanoscale. …, iopscience.iop.org, cited by 31 (15.50 per year)
NS Moghaddam, R Skoracki, M Miller, M Elahinia, ... (2016) Three dimensional printing of stiffness-tuned, nitinol skeletal fixation hardware with an example of mandibular segmental defect repair. Procedia CIRP, Elsevier, cited by 37 (12.33 per year)
S Kumar, BL Wardle, MF Arif (2016) Strength and performance enhancement of bonded joints by spatial tailoring of adhesive compliance via 3D printing. ACS applied materials & interfaces, ACS Publications, cited by 22 (7.33 per year)
T Sittiwanchai, I Nakayama, S Inoue, ... (2014) Transhumeral prosthesis prototype with 3D printing and sEMG-based elbow joint control method. Proceedings of the …, ieeexplore.ieee.org, cited by 6 (1.20 per year)
MI Maksud, MN Nodin, MS Yusof, ... (2016) Utilizing rapid prototyping 3D printer for fabricating flexographic PDMS printing plate. ARPN Journal of …, arpnjournals.org, cited by 2 (0.67 per year)
Q Gao, X Niu, L Shao, L Zhou, Z Lin, A Sun, J Fu, ... (2019) 3D printing of complex GelMA-based scaffolds with nanoclay. …, iopscience.iop.org, cited by 2 (2.00 per year)

A Cañizares, J Pazos, D Benítez (2017) On the use of 3D printing technology towards the development of a low-cost robotic prosthetic arm. 2017 IEEE International …, ieeexplore.ieee.org, cited by 2 (1.00 per year)
UK Roopavath, DM Kalaskar (2017) Introduction to 3D printing in medicine. 3D Printing in Medicine, Elsevier, cited by 6 (3.00 per year)
A Jones, J Straub (2017) Concepts for 3D Printing-Based Self-Replicating Robot Command and Coordination Techniques. Machines, mdpi.com, cited by 4 (2.00 per year)
E Unver, A Taylor (2015) 3D Additive Manufacturing Symposium & Workshop., eprints.hud.ac.uk, cited by 4 (1.00 per year)
Y Hao, Z Gong, Z Xie, S Guan, X Yang, T Wang, ... (2018) A soft bionic gripper with variable effective length. Journal of Bionic …, Springer, cited by 6 (6.00 per year)
LM Arruda, H Carvalho (2018) 3D Printing as a Design Tool for Wearables: Case Study of a Printed Glove. International Conference on Innovation …, Springer, cited by 4 (4.00 per year)
ME Hoque, WY San, F Wei, S Li, MH Huang, ... (2009) Processing of polycaprolactone and polycaprolactone-based copolymers into 3D scaffolds, and their cellular responses. … Engineering Part A, liebertpub.com, cited by 80 (8.00 per year)
J Wang, L Zhang, P Liu (2015) The 3D printing development study based on co-word analysis and multivariate statistics. 2015 International Industrial Informatics …, atlantis-press.com, cited by 5 (1.25 per year)
A Clausen, F Wang, JS Jensen, O Sigmund, ... (2015) Topology optimized architectures with programmable Poisson's ratio over large deformations. Advanced …, Wiley Online Library, cited by 145 (36.25 per year)
G Zhao, ZZ Sun, LL Li, Y Ge (2016) Fabrication of bionic linear actuator and application study based on 3d printing. Journal of Biomimetics, Biomaterials …, Trans Tech Publ, cited by 1 (0.33 per year)
Y Lin, J Xu (2018) Microstructures Fabricated by Two-Photon Polymerization and Their Remote Manipulation Techniques: Toward 3D Printing of Micromachines. Advanced Optical Materials, Wiley Online Library, cited by 8 (8.00 per year)
J Cunha, R Sethi, K Mellis, T Siauw, ... (2014) WE-F-16A-01: Commissioning and Clinical Use of PC-ISO for Customized, 3D Printed, Gynecological Brachytherapy Applicators. Medical …, Wiley Online Library, cited by 6 (1.20 per year)
H Liu, H Zhou, H Lan, T Liu (2017) Organ regeneration: integration application of cell encapsulation and 3D bioprinting. Virtual and Physical Prototyping, Taylor & Francis, cited by 7 (3.50 per year)
L Zhao, X Pei, L Jiang, C Hu, J Sun, F Xing, ... (2019) Bionic design and 3D printing of porous titanium alloy scaffolds for bone tissue repair. Composites Part B …, Elsevier, cited by 1 (1.00 per year)
S Shan, SH Kang, JR Raney, P Wang, ... (2015) Multistable architected materials for trapping elastic strain energy. Advanced …, Wiley Online Library, cited by 198 (49.50 per year)
L Wang, J Kang, C Sun, D Li, Y Cao, Z Jin (2017) Mapping porous microstructures to yield desired mechanical properties for application in 3D printed bone scaffolds and orthopaedic implants. Materials & Design, Elsevier, cited by 29 (14.50 per year)
ME Pogarasteanu, AG Barbilian (2014) Bionic hand exoprosthesis–Perspectives for the future in Romania. Journal of medicine and life, ncbi.nlm.nih.gov, cited by 4 (0.80 per year)
JS Lee, BS Kim, D Seo, JH Park, ... (2017) Three-dimensional cell printing of large-volume tissues: Application to ear regeneration. Tissue Engineering Part C …, liebertpub.com, cited by 12 (6.00 per year)

TJ Hinton, A Lee, AW Feinberg (2017) 3D bioprinting from the micrometer to millimeter length scales: Size does matter. Current Opinion in Biomedical …, Elsevier, cited by 16 (8.00 per year)
SA Park, SJ Lee, JM Seok, JH Lee, WD Kim, ... (2018) Fabrication of 3D printed PCL/PEG polyblend scaffold using rapid prototyping system for bone tissue engineering application. Journal of Bionic …, Springer, cited by 4 (4.00 per year)
R Becker, A Grzesiak, A Henning (2005) Rethink assembly design. Assembly automation, emeraldinsight.com, cited by 51 (3.64 per year)
Z Zhang, XJ Wang (2017) Current progresses of 3D bioprinting based tissue engineering. Quantitative Biology, Springer, cited by 3 (1.50 per year)
K Osouli-Bostanabad, K Adibkia (2018) Made-on-demand, complex and personalized 3D-printed drug products. BioImpacts: BI, ncbi.nlm.nih.gov, cited by 3 (3.00 per year)
H Sareen, U Umapathi, P Shin, Y Kakehi, J Ou, ... (2017) Printflatables: printing human-scale, functional and dynamic inflatable objects. Proceedings of the …, dl.acm.org, cited by 22 (11.00 per year)
G Haghiashtiani, E Habtour, SH Park, F Gardea, ... (2018) 3D printed electrically-driven soft actuators. Extreme Mechanics …, Elsevier, cited by 9 (9.00 per year)
MI Mohammed, B Cadd, G Peart, ... (2018) Augmented patient-specific facial prosthesis production using medical imaging modelling and 3D printing technologies for improved patient outcomes. Virtual and Physical …, Taylor & Francis, cited by 7 (7.00 per year)
H Ko, H Yi, HE Jeong (2017) Wall and ceiling climbing quadruped robot with superior water repellency manufactured using 3D printing (UNIclimb). International Journal of Precision Engineering and …, Springer, cited by 15 (7.50 per year)
E Biazar, M Najafi S, S Heidari K, ... (2018) 3D bio-printing technology for body tissues and organs regeneration. Journal of medical …, Taylor & Francis, cited by 4 (4.00 per year)
MA de Souza, C Schmitz, MM Pinhel, ... (2017) Proposal of custom made wrist orthoses based on 3D modelling and 3D printing. 2017 39th Annual …, ieeexplore.ieee.org, cited by 2 (1.00 per year)
T Mikolajczyk, A Borboni, XW Kong, ... (2015) 3D printed biped walking robot. Applied Mechanics …, Trans Tech Publ, cited by 2 (0.50 per year)
MS Alsoufi, AE Elsayed (2017) How surface roughness performance of printed parts manufactured by desktop FDM 3D printer with PLA+ is influenced by measuring direction. Am. J. Mech. Eng, researchgate.net, cited by 13 (6.50 per year)
YZ Zhang (2015) Innovations in orthopedics and traumatology in China. Chinese medical journal, ncbi.nlm.nih.gov, cited by 13 (3.25 per year)
K Arai, Y Tsukamoto, H Yoshida, H Sanae, ... (2016) The development of cell-adhesive hydrogel for 3D printing. Int. J …, researchgate.net, cited by 20 (6.67 per year)
DB Jones, R Sung, C Weinberg, T Korelitz, ... (2016) Three-dimensional modeling may improve surgical education and clinical practice. Surgical …, journals.sagepub.com, cited by 48 (16.00 per year)
SH Ahn, J Lee, SA Park, WD Kim (2016) Three-dimensional bio-printing equipment technologies for tissue engineering and regenerative medicine. Tissue engineering and regenerative …, Springer, cited by 6 (2.00 per year)
LC Hieu, N Zlatov, J Vander Sloten, ... (2005) Medical rapid prototyping applications and methods. Assembly …, emeraldinsight.com, cited by 174 (12.43 per year)
R Yadav, M Naebe, X Wang, ... (2017) Review on 3D prototyping of damage tolerant interdigitating brick arrays of nacre. Industrial & …, ACS Publications, cited by 9 (4.50 per year)

J Huang, H Fu, Z Wang, Q Meng, S Liu, H Wang, ... (2016) BMSCs-laden gelatin/sodium alginate/carboxymethyl chitosan hydrogel for 3D bioprinting. Rsc Advances, pubs.rsc.org, cited by 14 (4.67 per year)
Y QIAO, J WANG (2012) Application of 3D Printing Technology in Container Molding Design [J]. Packaging Engineering, en.cnki.com.cn, cited by 3 (0.43 per year)
L Wang, J Lau, EL Thomas, MC Boyce (2011) Co-continuous composite materials for stiffness, strength, and energy dissipation. Advanced Materials, Wiley Online Library, cited by 139 (17.38 per year)
Z Wu, X Su, Y Xu, B Kong, W Sun, S Mi (2016) Bioprinting three-dimensional cell-laden tissue constructs with controllable degradation. Scientific reports, nature.com, cited by 134 (44.67 per year)
S Midha, S Ghosh (2017) Silk-based bioinks for 3D bioprinting. Regenerative Medicine: Laboratory to Clinic, Springer, cited by 4 (2.00 per year)
MV Sarakinioti, M Turrin, T Konstantinou, ... (2018) Developing an integrated 3D-printed façade with complex geometries for active temperature control. Materials Today …, Elsevier, cited by 4 (4.00 per year)
S Anwar, GK Singh, J Miller, ... (2018) 3D printing is a transformative technology in congenital heart disease. JACC: Basic to …, basictranslational.onlinejacc.org, cited by 3 (3.00 per year)
S Kelly, A Paterson, RJ Bibb (2015) A review of wrist splint designs for additive manufacture., dspace.lboro.ac.uk, cited by 13 (3.25 per year)
F Blaya, P San Pedro, JL Silva, R D'Amato, ... (2018) Design of an Orthopedic Product by using additive manufacturing technology: The arm splint. Journal of medical …, Springer, cited by 11 (11.00 per year)
M Macko, E Mikołajewska, Z Szczepański, ... (2016) Repository of images for reverse engineering and medical simulation purposes., dspace.ukw.edu.pl, cited by 9 (3.00 per year)
J Firth, AW Basit, S Gaisford (2018) The role of semi-solid extrusion printing in clinical practice. 3D Printing of Pharmaceuticals, Springer, cited by 2 (2.00 per year)
QIU Haifei (2017) Application of 3D printing in beating-up and shedding mechanism of loom. Journal of Textile Research, en.cnki.com.cn, cited by 1 (0.50 per year)
G Thrivikraman, CM França, A Athirasala, ... (2017) Nanomaterials in 3D bioprinting: current approaches and future possibilities. … Prev Regener Med …, books.google.com, cited by 4 (2.00 per year)
CS Reddy, RM Hegde (2016) Design and development of bionic ears for rendering binaural audio. 2016 International Conference on …, ieeexplore.ieee.org, cited by 1 (0.33 per year)
Q Zhao, Y Liang, L Ren, Z Yu, Z Zhang, L Ren (2018) Bionic intelligent hydrogel actuators with multimodal deformation and locomotion. Nano Energy, Elsevier, cited by 4 (4.00 per year)
J Luo, Y Guo, X Wang (2018) Enhancing the imaging quality and fabrication efficiency of bionic compound eyes using a sandwich structure. Journal of Modern Optics, Taylor & Francis, cited by 1 (1.00 per year)
Y Tong, JM Murbach, V Subramanian, S Chhatre, ... (2018) A hybrid 3D printing and robotic-assisted embedding approach for design and fabrication of nerve cuffs with integrated locking mechanisms. MRS …, cambridge.org, cited by 3 (3.00 per year)
L Banović, B Vihar (2018) Development of an extruder for open source 3D bioprinting. Journal of Open Hardware, openhardware.metajnl.com, cited by 5 (5.00 per year)

MA Culver, PJ Tennant, M Cooper (2015) Contoured pick and a method of multiple variations of 3D CAD models. US Patent App. 13/998,186, Google Patents, cited by 8 (2.00 per year)
YE Tarang (2015) 3d printing additive manufacturing. International Education and Research Journal, academia.edu, cited by 2 (0.50 per year)
GB Hatton, CM Madla, S Gaisford, AW Basit (2018) Medical applications of 3D Printing. 3D Printing of …, Springer, cited by 1 (1.00 per year)
K Karzyński, K Kosowska, ... (2018) Use of 3D bioprinting in biomedical engineering for clinical application. Medical Studies …, researchgate.net, cited by 1 (1.00 per year)
Y Yang, Y Chen (2017) 3D printing of smart materials for robotics with variable stiffness and position feedback. 2017 IEEE International Conference on …, ieeexplore.ieee.org, cited by 3 (1.50 per year)
JZ Gul, BS Yang, YJ Yang, DE Chang, ... (2016) In situ UV curable 3D printing of multi-material tri-legged soft bot with spider mimicked multi-step forward dynamic gait. Smart Materials and …, iopscience.iop.org, cited by 21 (7.00 per year)
D Rus, MT Tolley (2015) Design, fabrication and control of soft robots. Nature, nature.com, cited by 1292 (323.00 per year)
P Jahnke, FRP Limberg, A Gerbl, GL Ardila Pardo, ... (2016) Radiopaque three-dimensional printing: a method to create realistic CT phantoms. Radiology, pubs.rsna.org, cited by 13 (4.33 per year)
M Alimanova, S Borambayeva, ... (2017) Gamification of hand rehabilitation process using virtual reality tools: Using leap motion for hand rehabilitation. 2017 First IEEE …, ieeexplore.ieee.org, cited by 11 (5.50 per year)
HI Wen, S Zhang, K Hapeshi, X Wang (2008) An innovative methodology of product design from nature. Journal of Bionic Engineering, Elsevier, cited by 34 (3.09 per year)
PY Tsai, CC Huang, YH Wen, HH Shen, ... (2015) Bionic fixing apparatus. US Patent App. 14 …, Google Patents, cited by 1 (0.25 per year)
S Kazi Marzuka, JU Kulsum (2016) 3D printing: a new avenue in pharmaceuticals. World Journal of Pharmaceutical Research, wjpr.net, cited by 3 (1.00 per year)
M Wilson (2011) Festo drives automation forwards. Assembly Automation, emeraldinsight.com, cited by 17 (2.13 per year)
BG Compton, JA Lewis (2014) 3D-printing of lightweight cellular composites. Advanced materials, Wiley Online Library, cited by 560 (112.00 per year)
C Kim, D Espalin, M Liang, H Xin, A Cuaron, ... (2017) 3D printed electronics with high performance, multi-layered electrical interconnect. IEEE …, ieeexplore.ieee.org, cited by 6 (3.00 per year)
M Chen, Z Xu, JH Kim, SK Seol, JT Kim (2018) Meniscus-on-Demand Parallel 3D Nanoprinting. ACS nano, ACS Publications, cited by 4 (4.00 per year)
L Zhao, G Zeng, Y Gu, Z Tang, G Wang, T Tang, ... (2019) Nature inspired fractal tree-like photobioreactor via 3D printing for CO2 capture by microaglae. Chemical Engineering …, Elsevier, cited by 2 (2.00 per year)
YD Choi, Y Kim, ES Park (2017) Patient-specific augmentation rhinoplasty using a three-dimensional simulation program and three-dimensional printing. Aesthetic surgery journal, academic.oup.com, cited by 8 (4.00 per year)
L Nickels (2015) AM and aerospace: an ideal combination. Metal Powder Report, Elsevier, cited by 51 (12.75 per year)
GL Goh, S Agarwala, WY Yong (2016) 3D printing of microfludic sensor for soft robots: a preliminary study in design and fabrication., dr.ntu.edu.sg, cited by 2 (0.67 per year)

Y Xiqiong (2013) Discussion on Three Dimensional Printing in Furniture Products Development [J]. Furniture & Interior Design, en.cnki.com.cn, cited by 6 (1.00 per year)
F Gilbert, CD O'Connell, T Mladenovska, ... (2018) Print me an organ? Ethical and regulatory issues emerging from 3D bioprinting in medicine. Science and engineering …, Springer, cited by 14 (14.00 per year)
E KluSKa, P Gruda, N Majca-Nowak (2018) The accuracy and the printing resolution comparison of different 3D printing technologies. Transactions on Aerospace …, yadda.icm.edu.pl, cited by 1 (1.00 per year)
M Gallab, K Tomita, S Omata, F Arai (2018) Fabrication of 3D Capillary Vessel Models with Circulatory Connection Ports. Micromachines, mdpi.com, cited by 1 (1.00 per year)
S Fleischer, R Feiner, T Dvir (2017) Cutting-edge platforms in cardiac tissue engineering. Current opinion in biotechnology, Elsevier, cited by 10 (5.00 per year)
PR Selvaganapathy, R Attalla (2016) Microfluidic vascular channels in gels using commercial 3D printers. Microfluidics, BioMEMS, and …, spiedigitallibrary.org, cited by 4 (1.33 per year)
SH Park, CS Jung, BH Min (2016) Advances in three-dimensional bioprinting for hard tissue engineering. Tissue engineering and regenerative …, Springer, cited by 14 (4.67 per year)
DP Laverty, MBM Thomas, P Clark, LD Addy (2016) The use of 3D metal printing (direct metal laser sintering) in removable prosthodontics. Dental update, magonlinelibrary.com, cited by 4 (1.33 per year)
M Bible, M Sefa, JA Fedchak, J Scherschligt, ... (2018) 3D-printed acrylonitrile butadiene styrene-metal organic framework composite materials and their gas storage properties. 3D Printing and …, liebertpub.com, cited by 6 (6.00 per year)
B Farahi (2016) Caress of the gaze: A gaze actuated 3D printed body architecture. 36th Annual Conference of the Association for …, papers.cumincad.org, cited by 6 (2.00 per year)
J Li, H Tanaka (2018) Feasibility study applying a parametric model as the design generator for 3D–printed orthosis for fracture immobilization. 3D printing in medicine, threedmedprint.biomedcentral.com, cited by 6 (6.00 per year)
P Volonghi, G Baronio, A Signoroni (2018) 3D scanning and geometry processing techniques for customised hand orthotics: an experimental assessment. Virtual and Physical …, Taylor & Francis, cited by 6 (6.00 per year)
YC Sun, R Li, YS Zhou, Y Wang (2017) Application and outlook of three-dimensional printing in prosthetic dentistry. Zhonghua kou qiang yi xue za zhi …, europepmc.org, cited by 1 (0.50 per year)
R Mutlu, SK Yildiz, G Alici, ... (2016) Mechanical stiffness augmentation of a 3D printed soft prosthetic finger. 2016 IEEE …, ieeexplore.ieee.org, cited by 7 (2.33 per year)
H Zhao, W Liang (2017) A novel comby scaffold with improved mechanical strength for bone tissue engineering. Materials Letters, Elsevier, cited by 14 (7.00 per year)
F Obregon, C Vaquette, S Ivanovski, ... (2015) Three-dimensional bioprinting for regenerative dentistry and craniofacial tissue engineering. Journal of Dental …, journals.sagepub.com, cited by 88 (22.00 per year)
SW Kwok, KHH Goh, ZD Tan, STM Tan, WW Tjiu, ... (2017) Electrically conductive filament for 3D-printed circuits and sensors. Applied Materials …, Elsevier, cited by 36 (18.00 per year)
S Mao, E Dong, H Jin, M Xu, S Zhang, J Yang, ... (2014) Gait study and pattern generation of a starfish-like soft robot with flexible rays actuated by SMAs. Journal of Bionic …, Springer, cited by 45 (9.00 per year)
JJ Schwartz, AJ Boydston (2019) Multimaterial actinic spatial control 3D and 4D printing. Nature communications, nature.com, cited by 4 (4.00 per year)

TL Nguyen, SJ Allen, SJ Phee (2013) Exploiting 3D printing technology to develop robotic running foot for footwear testing: This paper describes a framework for a prosthesis that has four controlled …. Virtual and Physical Prototyping, Taylor & Francis, cited by 5 (0.83 per year)
R Stach, J Haas, E Tu̇tu̇ncu̇, S Daboss, ... (2017) PolyHWG: 3D Printed Substrate-Integrated Hollow Waveguides for Mid-Infrared Gas Sensing. ACS …, ACS Publications, cited by 3 (1.50 per year)
S Wang, L Zhou, Z Luo, Y Wang, ... (2017) Lightweight of Artificial Bone Models Utilizing Porous Structures and 3D Printing.. International Journal of …, search.ebscohost.com, cited by 1 (0.50 per year)
A Kumar (2018) Methods and Materials for Smart Manufacturing: Additive Manufacturing, Internet of Things, Flexible Sensors and Soft Robotics. Manufacturing Letters, Elsevier, cited by 12 (12.00 per year)
EG Gordeev, AS Galushko, VP Ananikov (2018) Improvement of quality of 3D printed objects by elimination of microscopic structural defects in fused deposition modeling. PloS one, journals.plos.org, cited by 10 (10.00 per year)
AA Portnova, G Mukherjee, KM Peters, A Yamane, ... (2018) Design of a 3D-printed, open-source wrist-driven orthosis for individuals with spinal cord injury. PloS one, journals.plos.org, cited by 6 (6.00 per year)
L Lu, W Chen (2010) Biocompatible composite actuator: a supramolecular structure consisting of the biopolymer chitosan, carbon nanotubes, and an ionic liquid. Advanced Materials, Wiley Online Library, cited by 80 (8.89 per year)
J Gardan (2016) Additive manufacturing technologies: state of the art and trends. International Journal of Production Research, Taylor & Francis, cited by 141 (47.00 per year)
R Ballagas, S Ghosh, J Landay (2018) The design space of 3D printable interactivity. Proceedings of the ACM on Interactive …, dl.acm.org, cited by 5 (5.00 per year)
HM Zhao, FF Yang, JZ Fu, Q Gao, A Liu, ... (2017) Printing@ clinic: from medical models to organ implants. ACS Biomaterials …, ACS Publications, cited by 3 (1.50 per year)
Z Sun, S Aldosari (2018) Three-dimensional printing in medicine: Opportunities for development of optimal CT scanning protocols. Australasian Medical Journal (Online), search.proquest.com, cited by 2 (2.00 per year)
Q Yang, H Li, M Li, Y Li, S Chen, B Bao, ... (2017) Rayleigh instability-assisted satellite droplets elimination in inkjet printing. ACS applied materials …, ACS Publications, cited by 4 (2.00 per year)
T Han, S Kundu, A Nag, Y Xu (2019) 3D Printed Sensors for Biomedical Applications: A Review. Sensors, mdpi.com, cited by 2 (2.00 per year)
UJ Pai, NP Sarath, R Sidharth, ... (2016) Design and manufacture of 3D printec myoelectric multi-fingered hand for prosthetic application. … on Robotics and …, ieeexplore.ieee.org, cited by 4 (1.33 per year)
X Zhao, J Xiao, Y Sun, Z Zhu, M Xu, X Wang, ... (2018) Novel 3D printed modular hemipelvic prosthesis for successful hemipelvic arthroplasty: a case study. Journal of Bionic …, Springer, cited by 1 (1.00 per year)
A Aimar, A Palermo, B Innocenti (2019) The Role of 3D Printing in Medical Applications: A State of the Art. Journal of healthcare engineering, hindawi.com, cited by 3 (3.00 per year)
Y Shanjani, JNA De Croos, RM Pilliar, ... (2010) Solid freeform fabrication and characterization of porous calcium polyphosphate structures for tissue engineering purposes. … Research Part B …, Wiley Online Library, cited by 100 (11.11 per year)

A Miriyev, K Stack, H Lipson (2017) Soft material for soft actuators. Nature communications, nature.com, cited by 103 (51.50 per year)
J Chen, X Zhang, Y Okabe, K Saito, Z Guo, L Pan (2017) The deformation mode and strengthening mechanism of compression in the beetle elytron plate. Materials & Design, Elsevier, cited by 17 (8.50 per year)
MV Sarakinioti, T Konstantinou, ... (2018) Development and prototyping of an integrated 3D-printed façade for thermal regulation in complex geometries. Journal of Facade …, journals.open.tudelft.nl, cited by 2 (2.00 per year)
C Dordlofva, A Lindwall, P Törlind (2016) Opportunities and challenges for additive manufacturing in space applications. DS 85-1: Proceedings of …, designsociety.org, cited by 12 (4.00 per year)
JO Figueroa-Cavazos, E Flores-Villalba, ... (2016) Design concepts of polycarbonate-based intervertebral lumbar cages: finite element analysis and compression testing. Applied bionics and …, hindawi.com, cited by 6 (2.00 per year)
JC Hu, KA Athanasiou (2006) A self-assembling process in articular cartilage tissue engineering. Tissue engineering, liebertpub.com, cited by 235 (18.08 per year)
BN Johnson, KZ Lancaster, G Zhen, ... (2015) 3D printed anatomical nerve regeneration pathways. Advanced functional …, Wiley Online Library, cited by 107 (26.75 per year)
W Zhu, C O'Brien, JR O'Brien, LG Zhang (2014) 3D nano/microfabrication techniques and nanobiomaterials for neural tissue regeneration. Nanomedicine, Future Medicine, cited by 78 (15.60 per year)
LA Hockaday, B Duan, KH Kang, ... (2014) 3D-printed hydrogel technologies for tissue-engineered heart valves. 3D Printing and Additive …, liebertpub.com, cited by 16 (3.20 per year)
GH Vardhan, GH Charan, PVS Reddy, ... (2013) 3D printing: the dawn of a new era in manufacturing. Int. J. Recent Innov …, academia.edu, cited by 6 (1.00 per year)
ES Bishop, S Mostafa, M Pakvasa, HH Luu, MJ Lee, ... (2017) 3-D bioprinting technologies in tissue engineering and regenerative medicine: Current and future trends. Genes & diseases, Elsevier, cited by 47 (23.50 per year)
J Fajardo, V Ferman, A Lemus, ... (2017) An affordable open-source multifunctional upper-limb prosthesis with intrinsic actuation. 2017 IEEE Workshop on …, ieeexplore.ieee.org, cited by 7 (3.50 per year)
C Majidi (2014) Soft robotics: a perspective—current trends and prospects for the future. Soft Robotics, liebertpub.com, cited by 510 (102.00 per year)
A Vikram Singh, M Hasan Dad Ansari, S Wang, ... (2019) The adoption of three-dimensional additive manufacturing from biomedical material design to 3d organ printing. Applied Sciences, mdpi.com, cited by 3 (3.00 per year)
Y Wang, K Lee (2017) 3D-printed semi-soft mechanisms inspired by origami twisted tower. 2017 NASA/ESA Conference on Adaptive …, ieeexplore.ieee.org, cited by 2 (1.00 per year)
M Ceccarelli, D Cafolla, M Russo, ... (2017) LARM bot humanoid design towards a prototype. MOJ Appl. Bionics …, pdfs.semanticscholar.org, cited by 5 (2.50 per year)
S Zhou, W Zhang, Y Zou, B Ou, Y Zhang, C Wang (2018) Piezoelectric-driven self-assembling micro air vehicle with bionic reciprocating wings. Electronics Letters, IET, cited by 1 (1.00 per year)
M Macko, Z Szczepański, D Mikołajewski, ... (2016) The method of artificial organs fabrication based on reverse engineering in medicine. 1st Renewable Energy …, Springer, cited by 6 (2.00 per year)

X Guo, X Wang, D Ou, J Ye, W Pang, Y Huang, ... (2018) Controlled mechanical assembly of complex 3D mesostructures and strain sensors by tensile buckling. npj Flexible …, nature.com, cited by 3 (3.00 per year)
J O'Donnell, M Kim, HS Yoon (2017) A review on electromechanical devices fabricated by additive manufacturing. Journal of …, … .asmedigitalcollection.asme.org, cited by 24 (12.00 per year)
AY Alhaddad, SE AlKhatib, RA Khan, SM Ismail, ... (2017) Toward 3D printed prosthetic hands that can satisfy psychosocial needs: Grasping force comparisons between a prosthetic hand and human hands. … Conference on Social …, Springer, cited by 3 (1.50 per year)
Q Zhang, F Zhang, X Xu, C Zhou, D Lin (2018) Three-dimensional printing hollow polymer template-mediated graphene lattices with tailorable architectures and multifunctional properties. ACS nano, ACS Publications, cited by 8 (8.00 per year)
Y Zhang, T Zhang, M Liu, Y Kuang, G Zu, ... (2018) Aptamer-targeted magnetic resonance imaging contrast agents and their applications. … of nanoscience and …, ingentaconnect.com, cited by 3 (3.00 per year)
G Oberoi, S Nitsch, M Edelmayer, K Janjić, ... (2018) 3D printing—encompassing the facets of dentistry. … in bioengineering and …, ncbi.nlm.nih.gov, cited by 3 (3.00 per year)
A Ghanizadeh Tabriz, CG Mills, JJ Mullins, ... (2017) Rapid fabrication of cell-laden alginate hydrogel 3D structures by micro dip-coating. … in Bioengineering and …, frontiersin.org, cited by 7 (3.50 per year)
J Zhao, Y Zhang, Y Huang, X Zhao, Y Shi, ... (2019) Duplex printing of all-in-one integrated electronic devices for temperature monitoring. Journal of Materials …, pubs.rsc.org, cited by 3 (3.00 per year)
S Mao, E Dong, H Jin, M Xu, ... (2016) Locomotion and gait analysis of multi-limb soft robots driven by smart actuators. 2016 IEEE/RSJ …, ieeexplore.ieee.org, cited by 4 (1.33 per year)
I Chtioui, F Bossuyt, J Vanfleteren, ... (2018) 2.5/3D dynamically stretchable and permanently shaped electronic circuits. Microsystem Technologies, Springer, cited by 3 (3.00 per year)
S Sayyar, DL Officer, GG Wallace (2017) Fabrication of 3D structures from graphene-based biocomposites. Journal of Materials Chemistry B, pubs.rsc.org, cited by 7 (3.50 per year)
L Ben-Yehoshua, M Ochoa, ... (2015) Rapid fabrication of 3D elastomeric structures via laser-machining and vacuum deformation. 2015 Transducers-2015 …, ieeexplore.ieee.org, cited by 2 (0.50 per year)
C Basgul, T Yu, DW MacDonald, R Siskey, ... (2018) Structure–property relationships for 3D-printed PEEK intervertebral lumbar cages produced using fused filament fabrication. Journal of materials …, cambridge.org, cited by 5 (5.00 per year)
CO Yilmaz, ZS Xu, DH Gracias (2014) Curved and folded micropatterns in 3D cell culture and tissue engineering. Methods in cell biology, Elsevier, cited by 2 (0.40 per year)
MK Hofmann (2015) Making connections: modular 3D printing for designing assistive Attachments to prosthetic devices. Proceedings of the 17th International ACM …, parasol.tamu.edu, cited by 3 (0.75 per year)
E Salami, PB Ganesan, TA Ward, ... (2016) Design and mechanical analysis of a 3D-printed biodegradable biomimetic micro air vehicle wing. IOP Conference …, iopscience.iop.org, cited by 2 (0.67 per year)
R Liu, Z Wang, T Sparks, F Liou, J Newkirk (2017) Aerospace applications of laser additive manufacturing. Laser additive manufacturing, Elsevier, cited by 36 (18.00 per year)
M Conese (2014) Bioprinting: a further step to effective regenerative medicine and tissue engineering. Adv Genet Eng, longdom.org, cited by 5 (1.00 per year)

A Patwardhan (2018) How 3D Printing Will Change the Future of Borrowing Lending and Spending?. Handbook of Blockchain, Digital Finance, and Inclusion …, Elsevier, cited by 1 (1.00 per year)
C Meng, W Xu, H Li, T Wang (2015) A novel soft manipulator based on beehive structure. 2015 IEEE/RSJ International …, ieeexplore.ieee.org, cited by 6 (1.50 per year)
M Tian, Y Xiao, X Wang, J Chen, W Zhao (2017) Design and experimental research of pneumatic soft humanoid robot hand. Robot Intelligence Technology …, Springer, cited by 5 (2.50 per year)
M Abshirini, M Charara, P Marashizadeh, MC Saha, ... (2019) Functional nanocomposites for 3D printing of stretchable and wearable sensors. Applied …, Springer, cited by 1 (1.00 per year)
D Zhenbo, Z Changchun, ... (2016) Design and characterization of porous titanium scaffold for bone tissue engineering. RARE METAL …, … PO BOX 51, XIAN, SHAANXI 710016 …, cited by 2 (0.67 per year)
M Zou, S Xu, C Wei, H Wang, Z Liu (2016) A bionic method for the crashworthiness design of thin-walled structures inspired by bamboo. Thin-Walled Structures, Elsevier, cited by 30 (10.00 per year)
TL Khuong, Z Gang, M Farid, R Yu, ZZ Sun, ... (2014) Tensile strength and flexural strength testing of acrylonitrile butadiene styrene (ABS) materials for biomimetic robotic applications. Journal of …, Trans Tech Publ, cited by 4 (0.80 per year)
X Guo, Z Xu, F Zhang, X Wang, Y Zi, JA Rogers, ... (2018) Reprogrammable 3D mesostructures through compressive buckling of thin films with prestrained shape memory polymer. Acta Mechanica Solida …, Springer, cited by 3 (3.00 per year)
R Gorkin III, S Dodds (2013) The ultimate iron chef-when 3D printers invade the kitchen., ro.uow.edu.au, cited by 3 (0.50 per year)
C Dunn (2017) Integrated multi-media platform for hybrid 3D printing., smartech.gatech.edu, cited by 2 (1.00 per year)
JP Martins, MPA Ferreira, NZ Ezazi, JT Hirvonen, ... (2018) 3D printing: prospects and challenges. … in Preventive and …, Elsevier, cited by 1 (1.00 per year)
J Li, H Tanaka (2018) Rapid customization system for 3D-printed splint using programmable modeling technique–a practical approach. 3D printing in medicine, biomedcentral.com, cited by 3 (3.00 per year)
Q Hao, Z Wang, J Cao, F Zhang (2018) A hybrid bionic image sensor achieving FOV extension and foveated imaging. Sensors, mdpi.com, cited by 2 (2.00 per year)
AD Marchese, CD Onal, D Rus (2014) Autonomous soft robotic fish capable of escape maneuvers using fluidic elastomer actuators. Soft Robotics, liebertpub.com, cited by 336 (67.20 per year)
Y Zhang, X Wu, Y Liu, C Hu, S Sun, ... (2012) Bionic design of the body of tank-like climbing robot. 2012 IEEE International …, ieeexplore.ieee.org, cited by 1 (0.14 per year)
J Shi, J Yang, L Zhu, L Li, Z Li, X Wang (2017) A porous scaffold design method for bone tissue engineering using triply periodic minimal surfaces. IEEE Access, ieeexplore.ieee.org, cited by 7 (3.50 per year)
O Al-Ketan, RKA Al-Rub, ... (2017) Mechanical properties of a new type of architected interpenetrating phase composite materials. Advanced Materials …, Wiley Online Library, cited by 26 (13.00 per year)
R Mio, B Villegas, L Ccorimanya, ... (2017) Development and assessment of a powered 3D-printed prosthetic hand for transmetacarpal amputees. 2017 3rd …, ieeexplore.ieee.org, cited by 2 (1.00 per year)

F Schmieder, J Ströbel, M Rösler, ... (2016) 3D printing–a key technology for tailored biomedical cell culture lab ware. Current Directions in …, degruyter.com, cited by 5 (1.67 per year)
H Feng, W Dong, N Chai (2014) A bionic micro sucker actuated by IPMC. 2014 International Conference on …, ieeexplore.ieee.org, cited by 2 (0.40 per year)
MA Culver, PJ Tennant, M Cooper (2019) Contoured pick and a method of multiple variations of 3D CAD models. US Patent App. 10/319,349, Google Patents, cited by 2 (2.00 per year)
I Chtioui, F Bossyut, MH Bedoui (2016) Finite element simulation of 2.5/3D shaped and rigid electronic circuits. 2016 13th International …, ieeexplore.ieee.org, cited by 1 (0.33 per year)
H Li, YJ Tan, L Li (2018) A strategy for strong interface bonding by 3D bioprinting of oppositely charged κ-carrageenan and gelatin hydrogels. Carbohydrate polymers, Elsevier, cited by 2 (2.00 per year)
T Huang, C Fan, M Zhu, Y Zhu, W Zhang, L Li (2019) 3D-printed scaffolds of biomineralized hydroxyapatite nanocomposite on silk fibroin for improving bone regeneration. Applied Surface Science, Elsevier, cited by 1 (1.00 per year)
CAM Versos, DA Coelho (2012) Bionic Design: Presentation of a Two Way Methodology. Design Principles and Practices: an …, researchgate.net, cited by 3 (0.43 per year)
Y Luo, X Lin, P Huang (2018) 3D bioprinting of artificial tissues: Construction of biomimetic microstructures. Macromolecular bioscience, Wiley Online Library, cited by 2 (2.00 per year)
Y Govdeli, ZW Wong, E Kayacan (2016) Additive manufacturing of unmanned aerial vehicles: current status, recent advances, and future perspectives., dr.ntu.edu.sg, cited by 3 (1.00 per year)
T Beyrouthy, SK Al Kork, JA Korbane, ... (2016) EEG mind controlled smart prosthetic arm. … Practices for the …, ieeexplore.ieee.org, cited by 13 (4.33 per year)
P Alvial, G Bravo, MP Bustos, G Moreno, R Alfaro, ... (2018) Quantitative functional evaluation of a 3D–printed silicone-embedded prosthesis for partial hand amputation: A case report. Journal of Hand …, Elsevier, cited by 3 (3.00 per year)
MC McAlpine, YL Kong (2018) 3D printed active electronic materials and devices. US Patent 9,887,356, Google Patents, cited by 8 (8.00 per year)
W Jo, BJ Yoon, H Lee, MW Moon (2017) 3D Printed Hierarchical Gyroid Structure with Embedded Photocatalyst TiO2 Nanoparticles. 3D Printing and Additive …, liebertpub.com, cited by 2 (1.00 per year)
P XU, RUI SONG, S MAO, X RONG, ... (2016) Quadruped robot mechanism design and motion simulation based on SolidWorks and Adams. … ROBOTICS: Proceedings of …, World Scientific, cited by 2 (0.67 per year)
R Tognato, AR Armiento, V Bonfrate, ... (2019) A Stimuli-Responsive Nanocomposite for 3D Anisotropic Cell-Guidance and Magnetic Soft Robotics. Advanced Functional …, Wiley Online Library, cited by 3 (3.00 per year)
V Shulunov (2017) Comparison of algorithms for converting 3D objects into rolls, using a spiral coordinate system. Virtual and Physical Prototyping, Taylor & Francis, cited by 4 (2.00 per year)
U Ghosh, S Ning, Y Wang, ... (2018) Addressing unmet clinical needs with 3D printing technologies. Advanced healthcare …, Wiley Online Library, cited by 13 (13.00 per year)
JG Michaeli, MC DeGroff, RC Roxas (2017) Error aggregation in the reengineering process from 3D scanning to printing. Scanning, hindawi.com, cited by 1 (0.50 per year)

T Guo, J Lembong, LG Zhang, ... (2017) Three-Dimensional Printing Articular Cartilage: Recapitulating the Complexity of Native Tissue. Tissue Engineering Part B …, liebertpub.com, cited by 18 (9.00 per year)
R Xu, Z Wang, T Ma, Z Ren, H Jin (2019) Effect of 3D Printing Individualized Ankle-Foot Orthosis on Plantar Biomechanics and Pain in Patients with Plantar Fasciitis: A Randomized Controlled Trial. Medical science monitor …, ncbi.nlm.nih.gov, cited by 1 (1.00 per year)
S Smith (2016) 'Limbitless Solutions': the Prosthetic Arm, Iron Man and the Science Fiction of Technoscience. Medical humanities, mh.bmj.com, cited by 3 (1.00 per year)
JJ Cabibihan, MK Abubasha, N Thakor (2018) A Method for 3-D Printing Patient-Specific Prosthetic Arms With High Accuracy Shape and Size. IEEE Access, ieeexplore.ieee.org, cited by 3 (3.00 per year)
W Jiang, L Ma, B Zhang, Y Fan, X Qu, ... (2018) Evaluation of the 3D Augmented Reality–Guided Intraoperative Positioning of Dental Implants in Edentulous Mandibular Models.. … Journal of Oral & …, search.ebscohost.com, cited by 2 (2.00 per year)
L Wu, M Larkin, A Potnuru, Y Tadesse (2016) HBS-1: a modular child-size 3D printed humanoid. Robotics, mdpi.com, cited by 14 (4.67 per year)
A Ielapi, N Lammens, W Van Paepegem, ... (2019) A validated computational framework to evaluate the stiffness of 3D Printed Ankle Foot Orthoses. Computer methods in …, Taylor & Francis, cited by 2 (2.00 per year)
TA Mir, M Nakamura (2017) Three-Dimensional Bioprinting: Toward the Era of Manufacturing Human Organs as Spare Parts for Healthcare and Medicine. Tissue Engineering Part B: Reviews, liebertpub.com, cited by 26 (13.00 per year)
JW Boley, EL White, GTC Chiu, ... (2014) Direct writing of gallium-indium alloy for stretchable electronics. Advanced Functional …, Wiley Online Library, cited by 223 (44.60 per year)
PV Pincel, JL Jimenez-Perez, A Cruz-Orea, ... (2015) Photoacoustic study of curing time by UV laser radiation of a photoresin with different thickness. Thermochimica acta, Elsevier, cited by 3 (0.75 per year)
NH Tran, VC Nguyen, ... (2017) Study on design and manufacture of 3D printer based on fused deposition modeling technique. International Journal of …, pdfs.semanticscholar.org, cited by 1 (0.50 per year)
I Llop-Harillo, A Pérez-González (2017) System for the experimental evaluation of anthropomorphic hands. Application to a new 3D-printed prosthetic hand prototype. International BIomechanics, Taylor & Francis, cited by 2 (1.00 per year)
T Li, G Li, Y Liang, T Cheng, J Dai, ... (2017) Fast-moving soft electronic fish. Science …, advances.sciencemag.org, cited by 115 (57.50 per year)
C Tawk, M in het Panhuis, GM Spinks, G Alici (2018) Bioinspired 3D Printable Soft Vacuum Actuators for Locomotion Robots, Grippers and Artificial Muscles. Soft robotics, liebertpub.com, cited by 3 (3.00 per year)
H Guo, J Zhang, T Wang, Y Li, ... (2017) Design and control of an inchworm-inspired soft robot with omega-arching locomotion. 2017 IEEE International …, ieeexplore.ieee.org, cited by 8 (4.00 per year)
F Fang, S Aabith, S Homer-Vanniasinkam, ... (2017) High-resolution 3D printing for healthcare underpinned by small-scale fluidics. 3D Printing in …, Elsevier, cited by 1 (0.50 per year)
K Füzesi, A Basarab, G Cserey, ... (2017) Validation of image restoration methods on 3D-printed ultrasound phantoms. 2017 IEEE …, ieeexplore.ieee.org, cited by 1 (0.50 per year)

Y Zhou, W Sun, Q Ma, L Zhang (2018) Method of producing personalized biomimetic drug-eluting coronary stents by 3D-printing. US Patent 9,943,627, Google Patents, cited by 2 (2.00 per year)
K HU, LI Lh (2015) Application of 3D printing technology in orthopaedics personalized treatment. Polymer Bulletin, en.cnki.com.cn, cited by 1 (0.25 per year)
M Varkey, A Atala (2015) Organ bioprinting: a closer look at ethics and policies. Wake Forest JL & Pol'y, HeinOnline, cited by 25 (6.25 per year)
S Ghai, Y Sharma, N Jain, M Satpathy, ... (2018) Use of 3-D printing technologies in craniomaxillofacial surgery: a review. Oral and maxillofacial …, Springer, cited by 2 (2.00 per year)
X Yao, Q Chen, L Xu, Q Li, Y Song, ... (2010) Bioinspired ribbed nanoneedles with robust superhydrophobicity. Advanced functional …, Wiley Online Library, cited by 158 (17.56 per year)
G Savio, R Meneghello, S Rosso, ... (2019) 3D model representation and data exchange for additive manufacturing. Advances on Mechanics …, Springer, cited by 2 (2.00 per year)
F Blaya, PS Pedro, J Lopez-Silva, R D'Amato, ... (2017) Study, design and prototyping of arm splint with additive manufacturing process. Proceedings of the 5th …, dl.acm.org, cited by 2 (1.00 per year)
J Zarbakhsh, A Iravani, ... (2015) Sub-modeling finite element analysis of 3D printed structures. 2015 16th International …, ieeexplore.ieee.org, cited by 5 (1.25 per year)
CH Moon, JY Kim, MJ Kim, H Tchah, BG Lim, ... (2016) Effect of three-dimensional printed personalized moisture chamber spectacles on the periocular humidity. Journal of …, hindawi.com, cited by 3 (1.00 per year)
M Firoz (2016) Physics based modeling of filaments melting in fused deposition modeling for 3D printing., search.proquest.com, cited by 1 (0.33 per year)
F Gilbert, AR Harris, S Dodds, RMI Kapsa (2015) Is a 'last chance'treatment possible after an irreversible brain intervention?. AJOB Neuroscience, Taylor & Francis, cited by 3 (0.75 per year)
J Lee, S Chu, H Kim, K Choi, E Oh, JH Shim, WS Yun, ... (2017) Osteogenesis of adipose-derived and bone marrow stem cells with polycaprolactone/tricalcium phosphate and three-dimensional printing technology in a dog model …. Polymers, mdpi.com, cited by 2 (1.00 per year)
L Dobrzański (2014) Overview and general ideas of the development of constructions, materials, technologies and clinical applications of scaffolds engineering for regenerative …. Archives of Materials Science and Engineering, yadda.icm.edu.pl, cited by 19 (3.80 per year)
AJ Lin, JL Bernstein, JA Spector (2018) Ear Reconstruction and 3D Printing: Is It Reality?. Current Surgery Reports, Springer, cited by 1 (1.00 per year)
H Hwang, JH Bae, BC Min (2017) Design Guidelines for Sensor Locations on 3D Printed Prosthetic Hands. 2017 First IEEE International …, ieeexplore.ieee.org, cited by 1 (0.50 per year)
W Sun, B Starly, A Darling, ... (2004) Computer-aided tissue engineering: application to biomimetic modelling and design of tissue scaffolds. … and applied biochemistry, Wiley Online Library, cited by 218 (14.53 per year)
M Fernandez-Vicente, A Escario Chust, ... (2017) Low cost digital fabrication approach for thumb orthoses. Rapid Prototyping …, emeraldinsight.com, cited by 2 (1.00 per year)
Q Lian, C Chen, MC Uwayezu, W Zhang, ... (2015) Biphasic mechanical properties of in vivo repaired cartilage. Journal of Bionic …, Springer, cited by 4 (1.00 per year)

S Ponader, C Von Wilmowsky, ... (2010) In vivo performance of selective electron beam-melted Ti-6Al-4V structures. … Research Part A: An …, Wiley Online Library, cited by 125 (13.89 per year)
T Giffney, E Bejanin, AS Kurian, J Travas-Sejdic, ... (2017) Highly stretchable printed strain sensors using multi-walled carbon nanotube/silicone rubber composites. Sensors and Actuators A …, Elsevier, cited by 30 (15.00 per year)
M Sullivan, B Oh, I Taylor (2017) 3d Printed Prosthetic Hand., openscholarship.wustl.edu, cited by 1 (0.50 per year)
T Do, TJ Bauder, H Suen, ... (2018) Additively Manufactured Full-Density Stainless Steel 316L with Binder Jet Printing. ASME 2018 …, … .asmedigitalcollection.asme.org, cited by 2 (2.00 per year)
D Liu, W Nie, D Li, W Wang, L Zheng, J Zhang, ... (2019) 3D printed PCL/SrHA scaffold for enhanced bone regeneration. Chemical Engineering …, Elsevier, cited by 2 (2.00 per year)
SY Heo, SC Ko, GW Oh, N Kim, IW Choi, ... (2018) Fabrication and characterization of the 3D-printed polycaprolactone/fish bone extract scaffolds for bone tissue regeneration. … Research Part B …, Wiley Online Library, cited by 2 (2.00 per year)
LE Murr, LE Murr (2015) Biomimetics and biologically inspired materials. … of materials structures, properties, processing and …, Springer, cited by 11 (2.75 per year)
MECSS Thong, MECW Wen 3D Printing–Revolutionising Military Operations. Pointer J. Singap. Armed Forces, mindef.gov.sg, cited by 1 (0.00 per year)
Q Gu, H Zhu, J Li, X Li, J Hao, ... (2016) Three-dimensional bioprinting speeds up smart regenerative medicine. National Science …, academic.oup.com, cited by 4 (1.33 per year)
X Pu, G Li, H Huang (2016) Preparation, anti-biofouling and drag-reduction properties of a biomimetic shark skin surface. Biology open, bio.biologists.org, cited by 21 (7.00 per year)
SH Hashimdeen, M Miodownik, MJ Edirisinghe (2014) The design and construction of an electrohydrodynamic Cartesian robot for the preparation of tissue engineering constructs. PloS one, journals.plos.org, cited by 10 (2.00 per year)
M Wang, H Zhang, Q Hu, D Liu, H Lammer (2019) Research and implementation of a non-supporting 3D printing method based on 5-axis dynamic slice algorithm. Robotics and Computer …, Elsevier, cited by 1 (1.00 per year)
GI Peterson, JJ Schwartz, D Zhang, ... (2016) Production of materials with spatially-controlled cross-link density via vat photopolymerization. … applied materials & …, ACS Publications, cited by 34 (11.33 per year)
IA Otto, FPW Melchels, X Zhao, MA Randolph, ... (2015) Auricular reconstruction using biofabrication-based tissue engineering strategies. …, iopscience.iop.org, cited by 30 (7.50 per year)
S Sanadhya, N Vij, P Chaturvedi, S Tiwari, ... (2015) Medical applications of additive manufacturing. Int J of Scientific …, researchgate.net, cited by 2 (0.50 per year)
D Quiñones, J Ferragud-Agulló, R Pérez-Feito, ... (2018) A Tangible Educative 3D Printed Atlas of the Rat Brain. Materials, mdpi.com, cited by 1 (1.00 per year)
A Wang, C Zhou, Z Jin, W Xu (2017) Towards scalable and efficient GPU-enabled slicing acceleration in continuous 3D printing. 2017 22nd Asia and South …, ieeexplore.ieee.org, cited by 1 (0.50 per year)
L Ren, X Zhou, Q Liu, Y Liang, Z Song, ... (2018) 3D magnetic printing of bio-inspired composites with tunable mechanical properties. Journal of materials …, Springer, cited by 2 (2.00 per year)

D Ferreira, T Duarte, JL Alves, ... (2018) Development of low-cost customised hand prostheses by additive manufacturing. Plastics, Rubber and …, Taylor & Francis, cited by 2 (2.00 per year)
L Zhang, C Niu, S Bi, Y Cai (2013) Kinematic model analysis and design optimization of a bionic pectoral fins. 2013 IEEE International …, ieeexplore.ieee.org, cited by 2 (0.33 per year)
Q Hu, W Li, H Zhang, D Liu, F Peng, Y Duan (2017) Research into topology optimization and the FDM method for a space cracked membrane. Acta Astronautica, Elsevier, cited by 2 (1.00 per year)
SA Curline-Wandl, M Azam Ali (2016) Single channel myoelectric control of a 3D printed transradial prosthesis. Cogent Engineering, Taylor & Francis, cited by 2 (0.67 per year)
Y Takeuchi (2016) Printable hydroponic gardens: Initial explorations and considerations. Proceedings of the 2016 CHI Conference Extended …, dl.acm.org, cited by 2 (0.67 per year)
YC Fung, P Tong (2004) Bioengineering., World Scientific, cited by 2 (0.13 per year)
V Agrahari, V Agrahari, AK Mitra (2017) Inner ear targeted drug delivery: what does the future hold?., Future Science, cited by 2 (1.00 per year)
P Slade, A Akhtar, M Nguyen, ... (2015) Tact: Design and performance of an open-source, affordable, myoelectric prosthetic hand. 2015 IEEE International …, ieeexplore.ieee.org, cited by 38 (9.50 per year)
V Doroshenko, O Mul, ... (2015) Modeling of the Process of Three-Dimensional Metal Casting. Boundary Field Problems …, bfpcs-journals.rtu.lv, cited by 3 (0.75 per year)
M Zhu, Y Mori, M Xie, A Wada, ... (2018) A 3D printed Two DoF Soft Robotic Finger With Variable Stiffness. 2018 12th France …, ieeexplore.ieee.org, cited by 1 (1.00 per year)
E Yang, S Miao, J Zhong, Z Zhang, DK Mills, ... (2018) Bio-based polymers for 3D printing of bioscaffolds. Polymer …, Taylor & Francis, cited by 1 (1.00 per year)
GJ Gillispie, J Park, JS Copus, AKPR Asari, ... (2019) Three-Dimensional Tissue and Organ Printing in Regenerative Medicine. … of regenerative medicine, Elsevier, cited by 1 (1.00 per year)
MT Leddy, JT Belter, KD Gemmell, ... (2015) Lightweight custom composite prosthetic components using an additive manufacturing-based molding technique. 2015 37th Annual …, ieeexplore.ieee.org, cited by 4 (1.00 per year)
E Wojciechowski, AY Chang, ... (2019) Feasibility of designing, manufacturing and delivering 3D printed ankle-foot orthoses: a systematic review. Journal of foot …, jfootankleres.biomedcentral.com, cited by 1 (1.00 per year)
B Akhoundi, AH Behravesh (2019) Effect of filling pattern on the tensile and flexural mechanical properties of FDM 3D printed products. Experimental Mechanics, Springer, cited by 1 (1.00 per year)
VJ Prakash, M Möller, J Weber, ... (2019) Laser Metal Deposition of Titanium Parts with Increased Productivity. 3D Printing and Additive …, Springer, cited by 1 (1.00 per year)
F Buonamici, R Furferi, L Governi, S Lazzeri, ... (2018) A CAD-based procedure for designing 3D printable arm-wrist-hand cast. Comput. Aided Des …, cad-journal.net, cited by 1 (1.00 per year)
M Schult, E Buckow, H Seitz (2016) Experimental studies on 3D printing of barium titanate ceramics for medical applications. Current Directions in Biomedical …, degruyter.com, cited by 2 (0.67 per year)
N Li, P Yu, T Yang, L Zhao, Z Liu, ... (2017) Bio-inspired wearable soft upper-limb exoskeleton robot for stroke survivors. 2017 IEEE International …, ieeexplore.ieee.org, cited by 1 (0.50 per year)

M Jamal, SS Kadam, R Xiao, F Jivan, ... (2013) Bio-origami hydrogel scaffolds composed of photocrosslinked PEG bilayers. Advanced …, Wiley Online Library, cited by 90 (15.00 per year)
B Fischer, A Schulz, MM Gepp, J Neubauer, ... (2016) 3D printing of hydrogels in a temperature controlled environment with high spatial resolution. Current Directions in …, degruyter.com, cited by 3 (1.00 per year)
KA Thayer (2014) Mapping Human Enhancement Rhetoric. Global Issues and Ethical Considerations in Human …, igi-global.com, cited by 1 (0.20 per year)
Y Xiao, J Yan, K Hu, L Zhu, S Pan, L Li, ... (2016) Effect of high temperature on morphology and structure of a new composite as raw material of filament for fused deposition modeling processes. … Academic Conference on …, Springer, cited by 1 (0.33 per year)
ER Youngstrom (2016) 3D Printing and Healthcare: Will Laws, Lawyers, and Companies Stand in the Way of Patient Care. Pace Intell. Prop. Sports & Ent. LF, HeinOnline, cited by 1 (0.33 per year)
F Zhu, NP Macdonald, JM Cooper, ... (2013) Additive manufacturing of lab-on-a-chip devices: promises and challenges. Micro/Nano Materials …, spiedigitallibrary.org, cited by 14 (2.33 per year)
J Yan, K Yang, T Wang, J Zhao (2015) Research on design and jumping performance of a new water-jumping robot imitating water striders. 2015 IEEE International …, ieeexplore.ieee.org, cited by 1 (0.25 per year)
H Werner, JRL Dos Santos, R Fontes, ... (2010) Additive manufacturing models of fetuses built from three-dimensional ultrasound, magnetic resonance imaging and computed tomography scan data. … in Obstetrics and …, Wiley Online Library, cited by 56 (6.22 per year)
X Huang, S Lin, Y Hu, Y Liao, W Wang, ... (2018) Preparation and characterization of digital coral hydroxyapatite artificial bone scaffolds based on 3D printing. … of Biomaterials and …, ingentaconnect.com, cited by 4 (4.00 per year)
HA Almeida, AF Costa, C Ramos, C Torres, ... (2019) Additive Manufacturing Systems for Medical Applications: Case Studies. … in Training and …, Springer, cited by 1 (1.00 per year)
PY Tsai, CC Huang, YH Wen, HH Shen, ... (2015) Bionic fixing apparatus and apparatus for pulling out the same. US Patent App. 14 …, Google Patents, cited by 1 (0.25 per year)
T Duong, B Wagner, T Abraham, ... (2017) Comparative study of functional grasp and efficiency between a 3d-printed and commercial myoelectric transradial prosthesis using able-bodied subjects: A pilot study. JPO: Journal of …, journals.lww.com, cited by 1 (0.50 per year)
P Trogu (2015) Bioinspired Design: Aristotle's Lantern and Models of Rotational Geometry by Giorgio Scarpa. Abstracts, Design of Medical Devices Conference …, unixlab.sfsu.edu, cited by 1 (0.25 per year)
J Huang, H Fu, C Li, J Dai, Z Zhang (2017) Recent advances in cell-laden 3D bioprinting: materials, technologies and applications. Journal of 3D printing in …, Future Medicine, cited by 1 (0.50 per year)
C Altenhofen, TH Luu, T Grasser, ... (2018) Continuous property gradation for multi-material 3d-printed objects. Solid Freeform …, sffsymposium.engr.utexas.edu, cited by 1 (1.00 per year)
SH Abbasi, A Mahmood (2017) Bond graph modelling of a customized anthropomorphic prosthetic hand with LQR control synthesis. 2017 International Multi-topic …, ieeexplore.ieee.org, cited by 1 (0.50 per year)

X Xin (2016) Patent Eligibility of 3D-Printed Organs. AIPLA QJ, HeinOnline, cited by 1 (0.33 per year)
G Zhao, Z Sun, D Qiao, J Yang, ... (2017) Fabrication and applied investigation of a muscle-like linear actuator using Ionic polymer metal composites. Polymer …, Wiley Online Library, cited by 1 (0.50 per year)
M Gardiner (2011) Oribotics: The future unfolds. Origami^ 5: Fifth International Meeting of Origami …, books.google.com, cited by 8 (1.00 per year)
JK Carrow, A Di Luca, ... (2018) 3D-printed bioactive scaffolds from nanosilicates and PEOT/PBT for bone tissue engineering. Regenerative …, academic.oup.com, cited by 1 (1.00 per year)
J Chen, K Wang, C Zhang, B Wang (2018) An efficient statistical approach to design 3D-printed metamaterials for mimicking mechanical properties of soft biological tissues. Additive Manufacturing, Elsevier, cited by 1 (1.00 per year)
T Li, H Luo, L Qin, X Wang, Z Xiong, H Ding, Y Gu, ... (2016) Flexible capacitive tactile sensor based on micropatterned dielectric layer. Small, Wiley Online Library, cited by 74 (24.67 per year)
MZU Khan, MJ Yoga (2015) Governing policies & strategy for 3D Printing/additive manufacturing. 2015 International Conference on …, ieeexplore.ieee.org, cited by 1 (0.25 per year)
C Kengla, A Kidiyoor, SV Murphy (2017) Bioprinting Complex 3D Tissue and Organs. Kidney Transplantation, Bioengineering …, Elsevier, cited by 1 (0.50 per year)
T Lewis (1994) The replicator. Editor-in-chief's message, Computer, computer.org, cited by 1 (0.04 per year)
H Zhang, MY Wang, F Chen, Y Wang, ... (2017) Design and development of a soft gripper with topology optimization. 2017 IEEE/RSJ …, ieeexplore.ieee.org, cited by 9 (4.50 per year)
L Lu, J Liu, Y Hu, Y Zhang, ... (2012) Highly stable air working bimorph actuator based on a graphene nanosheet/carbon nanotube hybrid electrode. Advanced …, Wiley Online Library, cited by 80 (11.43 per year)
VY Shevchenko, MM Sychev, ... (2017) Ceramic Materials with the Triply Periodic Minimal Surface for Constructions Functioning under Conditions of Extreme Loads.. Glass Physics & …, search.ebscohost.com, cited by 4 (2.00 per year)
SR Grob, MK Yoon (2017) Innovations in orbital surgical navigation, orbital implants, and orbital surgical training. International ophthalmology clinics, journals.lww.com, cited by 1 (0.50 per year)
Z Li, S Jia, Z Xiong, Q Long, S Yan, F Hao, J Liu, ... (2018) 3D-printed scaffolds with calcified layer for osteochondral tissue engineering. Journal of bioscience …, Elsevier, cited by 6 (6.00 per year)
RL Simpson, FE Wiria, AA Amis, ... (2008) Development of a 95/5 poly(L-lactide-co-glycolide)/hydroxylapatite and β-tricalcium phosphate scaffold as bone replacement material via selective laser sintering. … Research Part B …, Wiley Online Library, cited by 148 (13.45 per year)
J Ponomareva, A Sokolova (2015) The identification of weak signals and wild cards in foresight methodology: Stages and methods. Higher School of Economics …, papers.ssrn.com, cited by 3 (0.75 per year)
X Li, J He, W Zhang, N Jiang, D Li (2016) Additive manufacturing of biomedical constructs with biomimetic structural organizations. Materials, mdpi.com, cited by 12 (4.00 per year)

A Bandyopadhyay, S Vahabzadeh, A Shivaram, ... (2015) Three-dimensional printing of biomaterials and soft materials. MRS …, cambridge.org, cited by 7 (1.75 per year)
CNM Ryan, KP Fuller, A Larrañaga, ... (2015) An academic, clinical and industrial update on electrospun, additive manufactured and imprinted medical devices. Expert review of …, Taylor & Francis, cited by 22 (5.50 per year)
Y Yang, X Song, X Li, Z Chen, C Zhou, ... (2018) Recent progress in biomimetic additive manufacturing technology: from materials to functional structures. Advanced …, Wiley Online Library, cited by 39 (39.00 per year)
J Kaspar, T Häfele, C Kaldenhoff, J Griebsch, ... (2017) Hybrid Additive Design of FRP Components–Fiber-Reinforced Sandwich Structures Based on Selective Laser Sintering Technology. Procedia CIRP, Elsevier, cited by 4 (2.00 per year)
W Liu, S Xu (2016) 3D Printing Technology and Its Applications. Advanced Material Engineering: Proceedings of the …, World Scientific, cited by 1 (0.33 per year)
M McAlpine (2015) 3D printed bionic nanomaterials. NIP & Digital Fabrication Conference, ingentaconnect.com
MC McAlpine (2014) Bionic Nano-Printing. NIP & Digital Fabrication Conference, ingentaconnect.com
SM Gulbrandsen (2015) Bionic hand: replicating the human hand through 3D printing., duo.uio.no
SUN Yanzheng, WU Jincheng, LI Yi, ... (2018) Mechanical Experiment for 3D Printing of Titanium Bone Bionic Dental Implants.. Medicinal …, search.ebscohost.com
RC Chiechi, EA Weiss, MD Dickey, ... (2008) Eutectic gallium–indium (EGaIn): a moldable liquid metal for electrical characterization of self-assembled monolayers. Angewandte Chemie …, Wiley Online Library, cited by 489 (44.45 per year)
K Lin, HE Shu, Y Song, Z Wang, ... (2016) Fabrication of a bionic artificial bone scaffold using a room temperature three dimensional printing technique. Chinese Journal of …, wprim.whocc.org.cn
OP Gupta (2016) 3D bioprinting: A revolutionary tool in medicine. Journal of Mahatma Gandhi Institute of Medical …, jmgims.co.in
H Mcculloch 3D Printing. webspace.pria.at
C Seepersad (2015) Presentation: The Future of 3D Printing: The Democratization of Design. The Future of 3D Printing: The Democratization of …, esi.utexas.edu
M Sebastian Mannoor (2014) Bionic Nanosystems., adsabs.harvard.edu
MC MCALPINE (2014) Bionic Nanosystems., dataspace.princeton.edu
MTJ Brown, PE Chong Chen A New Low-Cost Bionic Hand. peer.asee.org
Y Chen, Y Wang, Q Yang, Y Liao, B Zhu, ... (2018) A novel thixotropic magnesium phosphate-based bioink with excellent printability for application in 3D printing. Journal of Materials …, pubs.rsc.org
JV Pagaduan, A Bhatta, LH Romer, DH Gracias (2018) 3D Hybrid Small Scale Devices. Small, Wiley Online Library
Y Zhang, M Yin, O Xia, AP Zhang, ... (2018) Optical 3D μ-printing of polytetrafluoroethylene (PTFE) microstructures. 2018 IEEE Micro Electro …, ieeexplore.ieee.org
A Manero, P Smith, J Sparkman, ... (2019) Implementation of 3D Printing Technology in the Field of Prosthetics: Past, Present, and Future. International journal of …, mdpi.com
YM Ma, YY Liu, HP Chen, QX Hu (2015) Preparation and Characterization of Tissue Engineering Scaffolds by Composite Molding of 3D Printing and Electrospinning. Key Engineering Materials, Trans Tech Publ

K Ji, Y Wang, Q Wei, K Zhang, A Jiang, Y Rao, ... (2018) Application of 3D printing technology in bone tissue engineering. Bio-Design and …, Springer
X Bai, P Ding, P Zhang, Z Yao (2018) Research on Modeling of Bionic Porous Scaffold for Bone Defect Repair Based on Bone Mineral Density Distribution. 2018 International Conference …, atlantis-press.com
TUO Jiangmin, Z Hongbin, SHU Yanxin, ... (2018) A Peristaltic Bionic Robot Controlled By A Single Elastic-Gasbag *. … WRC Symposium on …, ieeexplore.ieee.org
T Liu, Y Xu, D Zheng, L Zhou, X Li, L Liu (2019) Fabrication and absorbing property of the tower-like absorber based on 3D printing process. Physica B: Condensed Matter, Elsevier
YM Ma, YY Liu, HP Chen, QX Hu (2015) Biological CAD/CAM/3D printing integrated molding platform and system building. Key Engineering Materials, Trans Tech Publ
C Gao, Y Deng, P Feng, Z Mao, P Li, B Yang, ... (2014) Current progress in bioactive ceramic scaffolds for bone repair and regeneration. International journal of …, mdpi.com, cited by 128 (25.60 per year)
L Kölle, O Schwarz (2016) Bionic forceps for the handling of sensitive tissue. Current Directions in Biomedical Engineering, degruyter.com
D Akilbekova, D Mektepbayeva (2017) Patient specific in situ 3D printing. 3D Printing in Medicine, Elsevier
BB da Silva, RN Porsani, LAV Hellmeister, ... (2019) Design and Development of a Myoelectric Upper Limb Prosthesis with 3D Printing: A Low-Cost Alternative. … Conference on Applied …, Springer
MC McAlpine (2017) 3D printing functional materials and devices (Conference Presentation). Micro-and Nanotechnology Sensors, Systems …, spiedigitallibrary.org
G OPAIT (2019) The Statistical Force in the Worldwide Performance of the Healthcare Applications, Concerning 3D Printing and the Artificial Intelligence. Annals of the University Dunarea de Jos of Galati …, researchgate.net
SV Shilko, TV Ryabchenko, SV Panin, ... (2018) 3D printing as a" nature like" method of optimized endoprostheses fabrication. … : сборник тезисов IV …, earchive.tpu.ru
C Yeghiazarian, L Balan (2018) Improving Grasping of Bionic Hand by Using Finger Compliance Design and Rapid Prototyping. Interactive Mobile Communication …, Springer
BJ Vogel (2014) 3D Printing, Materials Development, and IP: Protecting What's in the Printer. Bloomberg BNA Patent, Trademark & Copyright …, robinskaplan.com
T Mikołajczyk, T Malinowski, A Łukaszewicz, ... Use of 3D printing techniques in design process of unconventional walking robot. researchgate.net
D Li, Z Zhang, C Zheng, B Zhao, K Sun, ... (2016) CYTOCOMPATIBILITY AND PREPARATION OF BONE TISSUE ENGINEERING SCAFFOLD BY COMBINING LOW TEMPERATURE THREE DIMENSIONAL …. Zhongguo xiu fu …, europepmc.org
ZHU Nana, Z Hongbin, L Bing, ... (2018) The Effect of Different Scales on the Crawling Rate of Bionic Snake Robot *. … WRC Symposium on …, ieeexplore.ieee.org
J Yan, Y Xiao, K Hu, S Pan, Y Wang, B Zheng, ... (2018) Preparation of Hydrogel Material for 3D Bioprinting. Applied Sciences in …, Springer
L Bing, Z Hongbin, ZHU Nana, ... (2018) System Design and Experiment of Bionics Robotic Arm with Humanoid Characteristics. … WRC Symposium on …, ieeexplore.ieee.org
D Rochman (2013) Some remarks about the 3d printing and parametric modelling of the parts and joints of an animated puppet that simulate a humanoid robot. Journal Biuletyn of Polish Society for Geometry and …, yadda.icm.edu.pl

M De Leon (2018) Breakthroughs in Eye Augmentation: Artificial retinas and 3D-printed optoelectronics show promise. Review of Optometry, go.galegroup.com
K Al Adem, SS Bawazir, K Alameri, G Lucarini, ... (2018) A Bionic Sphincter for Stress Urinary Incontinence: Design and Preliminary Experiments. … on Intelligent Human …, Springer
JC Davidson, J Zhang, TJ Kane, ... (2019) Investigation of Surface Treatment Methods for 3D Printed Optical Components. CLEO: Applications and …, osapublishing.org
B Li Controlling a 3D printed bionic hand by using brain waves. mountainscholar.org
Y Zeng, Y Hong, S Wang (2016) Application and prospect of three-dimensional printing in bone tissue engineering. International Journal of Biomedical …, wprim.whocc.org.cn
A Blasiak, THM Guerin, DBL Teh, ... (2019) Fibro-Neuronal Guidance on Common, 3D-Printed Textured Substrates. IEEE transactions on …, ieeexplore.ieee.org
F Fu, M Zhao, X Li, C Chen, L Wang, H Sun, ... (2016) PREPARATION OF PERSONALIZED BRAIN CAVITY SCAFFOLD WITH THREE-DIMENSIONAL PRINTING TECHNOLOGY BASED ON MAGNETIC RESONANCE …. Zhongguo xiu fu …, europepmc.org
J Liu (2014) Degradable Scaffold Microstructure of Artificial Bioactive Bone fabricated by 3D Braiding Method. Applied Mechanics and Materials, Trans Tech Publ
L Kong, Z Xu, M Xu (2019) Research and design of functional microstructures with directional transport for bionic microfluidics. … : Micro-and Nano-Optics, Catenary Optics …, spiedigitallibrary.org
TL Khuong (2016) Design, Implementation and Analysis of 3D Printed Grasshopper Robot for Jumping Mechanism. Journal of Biomimetics, Biomaterials and Biomedical …, Trans Tech Publ
Q Gu (2017) Functional tissues from intelligent materials, 3D printing and stem cells., ro.uow.edu.au
E Goldemberg SONIFICA–The New Bionic. pdf.blucher.com.br
A Lezhebokov, G Leonid, ... (2016) 3D IC encoding solutions mechanism on the basis of evolutionary approach. 2016 6th International …, ieeexplore.ieee.org
Y DENG, X GUO (2017) Application of 3D Printing Rapid Prototyping Technology in Processing of Polymeric Materials. China Plastics, en.cnki.com.cn
N Holzman, L Francis PNEUMATIC SYSTEM DESIGN FOR DIRECT WRITE 3D PRINTING. sffsymposium.engr.utexas.edu
Y Jin, J Yu Research on 3D Printing in China: A Review. researchgate.net
LB June Revolutionizing Business Through 3D Printing. emerypharma.com
YA Prokofyeva (2014) Innovative Features of 3D-printer. Reports Scientific Society, moofrnk.com
R Jackson, M Ransley NEW DEVELOPMENTS AND OPPORTUNITIES IN 3D PRINTING. changemakers.com
KA Cañizares Villegas (2017) On the Use of 3D Printing Technology for the Development of a Low-Cost Prosthetic Arm Prototype Controlled by EMG Signals., repositorio.usfq.edu.ec
C Liu, Y Wang, L Ren, L Ren (2019) A Review of Biological Fluid Power Systems and Their Potential Bionic Applications. Journal of Bionic Engineering, Springer
Z Chen, J Hu, Y Liu, Y He (2019) 3D printing of complex GelMA-based scaffolds with nanoclay., researchgate.net
N SAHAI, M TECH, M GOGOI (2019) Techniques and Software Used in 3D Printing for Nanomedicine Applications. 3D Printing Technology in …, books.google.com
Y Cao, W Wang, J Wang, C Zhang (2019) Experimental and numerical study on tensile failure behavior of bionic suture joints. Journal of the mechanical behavior of …, Elsevier

S Omar, A Kasem, A Ahmad, SR Ya'akub, ... (2018) Implementation of Low-Cost 3D-Printed Prosthetic Hand and Tasks-Based Control Analysis. … in Information System, Springer
E KluSKa, P Gruda, N Majca-Nowak (2018) Strain optical analysis of 3D printing elements in different additive technologies in comparison with the finite element method. Transactions on Aerospace …, yadda.icm.edu.pl
H Liu, X Cheng, XH Yang, GM Zheng, ... (2019) Experimental study on parameters of 3D printing process for PEEK materials. IOP Conference Series …, iopscience.iop.org
AS Munoz-Abraham, C Ibarra, R Agarwal, ... (2017) 3D Bioprinting in Transplantation. … Advances in Organ …, Springer
KH Lee, DK Kim, YH Cha, JY Kwon, ... (2018) Personalized assistive device manufactured by 3D modelling and printing techniques. Disability and …, Taylor & Francis
A Frydrychewicz (2019) Bionic implants and manufacturing methods thereof. US Patent App. 16/097,246, Google Patents
P Giacomelli, A Smedberg (2014) The Eve of 3D Printing in Telemedicine: State of the Art and Future Challenges. arXiv preprint arXiv:1405.2305, arxiv.org
H Choi, A Seo, J Lee (2019) Mallet Finger Lattice Casts Using 3D Printing. Journal of Healthcare Engineering, hindawi.com
S Shen, M Chen, W Guo, H Li, X Li, ... (2019) Three Dimensional Printing-Based Strategies for Functional Cartilage Regeneration. … Engineering Part B …, liebertpub.com
JS Artal-Sevil, JL Montañés, A Acón, ... (2018) Control of a Bionic Hand using real-time gesture recognition techniques through Leap Motion Controller. … Applied to Electronics …, ieeexplore.ieee.org
D Parras-Burgos, PP García-Sandoval, ... (2019) Tumor Reconstructive Surgery Assisted by Scale Models Using 3D Printing. Advances on Mechanics …, Springer
X Lin, Y Zhang, M Ji, X Zheng, ... (2018) Dipole Source Localization Based on Least Square Method and 3D Printing. … on Mechatronics and …, ieeexplore.ieee.org
P Wang, Y Wang, F Ru (2017) Walking trajectory generation for a 3D printing biped robot based on human natural gait and ZMP criteria. 2017 IEEE International Conference …, ieeexplore.ieee.org
H Petersson (2017) Optimizing Products and Production Using Additive Manufacturing by Introducing Bionics Into the Engineering Design Process. ASME 2017 International …, … .asmedigitalcollection.asme.org
KB Fritzler, VY Prinz (2019) 3D printing methods for micro-and nanostructures. Physics-Uspekhi, iopscience.iop.org
R Barua, S Datta, A Roychowdhury, ... (2019) Importance of 3D Printing Technology in Medical Fields. … Technologies From an …, igi-global.com
B Dumé (2018) 3D printing makes world's first bionic mushroom. Physics World, iopscience.iop.org
H Li, J Yao, P Zhou, X Chen, Y Xu, Y Zhao (2019) High-Load Soft Grippers Based on Bionic Winding Effect. Soft robotics, liebertpub.com
Y Zhang (2017) 59. Ceramic 3D Printing Technology Based on Particle filter algorithm., pdfs.semanticscholar.org
F Ma, Y Zhao, Y Pu, G Wang (2019) Performance Analysis on 3D Printed Beak-Shaped Automotive Tail Fin Filled with Honeycomb Cellular Structure., sae.org
S Tarafder, CH Lee (2017) 3D printing integrated with controlled delivery for musculoskeletal tissue engineering. Journal of 3D printing in medicine, Future Medicine

Y Zhang, Y Liu, H Chen, Z Jiang, Q Hu (2014) Preparation and Evaluation of Physical/Chemical Gradient Bone Scaffold by 3D Bio-printing Technology.. ISPE CE, books.google.com
LA García-García, M Rodríguez-Salvador, ... (2019) Development of a Customized Wrist Orthosis for Flexion and Extension Treatment Using Reverse Engineering and 3D Printing. World Congress on …, Springer
E Salami, E Montazer, TA Ward, ... (2017) Nano-mechanical properties and structural of a 3D-printed biodegradable biomimetic micro air vehicle wing. IOP Conference Series …, iopscience.iop.org
Z Sun (2018) 3D printing in medicine: current applications and future directions. Quantitative imaging in medicine and surgery, ncbi.nlm.nih.gov
G Wallace 3D Bioprinting–New Dimensions for Bionics.
L Jiang, J Guo, B Su, P Xu, R Dang (2018) AI in Locomotion: Quadruped Bionic Mobile Robot. International Conference on …, Springer
H Park, H Choi, JR Usherwood Nano-mechanical properties and structural of a 3D-printed biodegradable biomimetic micro air vehicle wing. researchportal.hw.ac.uk
Q Jiang, L Zhu, L LI, Z LI, K LI, ... (2019) 3D Printing Equipment Utilizing Biological Material, and Method. US Patent App. 16 …, freepatentsonline.com
Y Zou, P Tan, B Shi, H Ouyang, D Jiang, Z Liu, ... (2019) A bionic stretchable nanogenerator for underwater sensing and energy harvesting. Nature …, nature.com
JYH Fuh, J Sun, Y Wu, H Wang, ... (2016) Method for making functionally gradient coatings by 3d printing based on electrostatic spinning and electrostatic spraying. US Patent App. 14/861,670, Google Patents
SN Rath, S Sankar (2017) 3D printers for surgical practice. 3D Printing in Medicine, Elsevier
GL Goh, S Agarwala, WY Yong (2016) 3D printing of microfludic sensor for soft robots: a., researchgate.net
UQ Home 3D Bioprinting–New Dimensions for Bionics.
PS Valchanov (2017) 3D Printing in medicine–principles, applications and challenges. Scripta Scientifica Vox Studentium, journals.mu-varna.bg
CGY Ngan, RMI Kapsa, PFM Choong (2019) Strategies for neural control of prosthetic limbs: from electrode interfacing to 3D printing. Materials, mdpi.com
CI March 3D Printing Revolutionizes The Healthcare Industry. mms.kabuku.io
N Galarza, B Rubio, A Bereziartua, I Lozano, J Gascon, ... (2019) IN-SERVICE INSPECTION OF AERONAUTICS PARTS PRODUCED BY ADDITIVE LAYER MANUFACTURING (ALM)-in the framework of Bionic Aircraft project (GA n° …., dsp.tecnalia.com
RP Harrison … , Department for Nanostructured Materials, Jozef Stefan Institute, Slovenia Additional hardware: Roman Bevc 3D printing: Stephan Doepner, Cirkulacija 2. ultramono.org
SH Tang, YT Wang, RCJ Jing, ... (2017) Bionic compound eye using microlens array with multi-focus and long focal depth. 2017 Symposium on …, ieeexplore.ieee.org
E Mussi, R Furferi, Y Volpe, F Facchini, KS McGreevy, ... (2019) Ear Reconstruction Simulation: From Handcrafting to 3D Printing. Bioengineering, mdpi.com
R Mio, L Ccorimanya, KM Flores, G Salazar, ... (2017) Design of a 3D-Printable Powered Prosthetic Hand for Transmetacarpal Amputees. Advances in Automation …, Springer
MS Mannoor, Z Jiang, T James, YL Kong, KA Malatesta, ... Supporting Information: A 3D Printed Bionic Ear.
A Sadeqi, HR Nejad, ... (2019) 3D printed metamaterials for high-frequency applications. Terahertz, RF, Millimeter …, spiedigitallibrary.org

D Carter-Davies, J Chen, F Chen, M Li, ... (2018) Mechatronic Design and Control of a 3D Printed Low Cost Robotic Upper Limb. … Workshop on Human …, ieeexplore.ieee.org
MA Golovin, NV Marusin, YB Golubeva (2018) Use of 3D Printing in the Orthopedic Prosthetics Industry. Biomedical Engineering, researchgate.net
J Li, H Tanaka (2017) The flexibility controlling study for 3D printed splint. … , Biosensors, Info-Tech Sensors and 3D …, spiedigitallibrary.org
PHD VINOGRADOV (2019) 3D Printing in Medicine: Current Challenges and Potential Applications. 3D Printing Technology in Nanomedicine, books.google.com
P Cortes, L Duggen, J Jouffroy, K Rogers, ... 3D Printed Hybrid Flexible Electronics with Direct Light Synthesis. Advances in Additive …, books.google.com
D Ortiz-Acosta, T Moore (2018) Functional 3D Printed Polymeric Materials. Functional Materials, intechopen.com
RR Torrealba, SB Udelman, AJ Rivas, FJ Carvalho A START-UP PROJECT TO CREATE CUSTOMISED 3D-PRINTED HAND PROSTHESES AFFORDABLE IN DEVELOPING COUNTRIES. researchgate.net
MT Tolley, RF Shepherd, B Mosadegh, KC Galloway, ... (2014) A resilient, untethered soft robot. Soft robotics, liebertpub.com, cited by 384 (76.80 per year)
A Upadhyay, JS Sharma 3D MODELLING AND ITS APPLICATIONS. ijaconline.com
K Achim, T Johannes, ... (2018) A Framework for Implementation of 3D-printing of Manufacturing Equipment. 2018 IEEE International …, ieeexplore.ieee.org
Y Wan (2015) Principle and Prospect on the 3D Printing Construction Technology. Journal of Beijing University of Civil Engineering and …, en.cnki.com.cn
RP Harrison … Podpečan, Department of Knowledge Technologies, Institute Jozef Stefan Custom-made electronics for Arbora Protectors objects: Gregor Krpič 3D printing …. ultramono.org
B Zhang, Y Li, G Wang, Z Jia, H Li, Q Peng, ... (2018) Fabrication of agarose concave petridish for 3D-culture microarray method for spheroids formation of hepatic cells. Journal of Materials …, Springer
R Su, SH Park, Z Li, MC McAlpine (2019) 3D printed electronic materials and devices. Robotic Systems and Autonomous …, Elsevier
A Bhatt, A Anbarasu Nanoscale Biomaterials for 3D printing. researchgate.net
S Ballard, LA Bartenhagen (2018) The Role of 3-D Printing in Radiation Oncology: A Literature Review.. Radiation Therapist, search.ebscohost.com
M Walker, S Humphries (2019) 3D Printing: Applications in evolution and ecology. Ecology and evolution, Wiley Online Library
A Washburn (2018) A New Virtual Reality for 3D Technology in the Utility Industry. IEEE Power and Energy Magazine, ieeexplore.ieee.org
R Iuganson (2018) Artificial Intelligence in 3D Printing: Real-time 3D printing control., theseus.fi
M Rimmer (2019) Makers empire: Australian copyright law, 3D printing, and the'ideas boom'. 3D Printing and Beyond: Intellectual Property and …, papers.ssrn.com
JE Bara, CI Hawkins, DT Neuberger, SW Poppell 3D printing for CO. researchgate.net
ME Kjelland, CD Piercy, TM Swannack (2017) Beyond graphs and tables: Enhancing explanatory power of complex environmental simulations through 3D printed model output. Ecological Modelling, Elsevier
A Popa, B Zellers, S Iversen, D Kennedy, ... (2019) 3D Printed Hybrid Flexible Electronics with Direct Light Synthesis. … Conference on Applied …, Springer

T Zhang, A Wang, Q Wang, F Guan (2019) Bending characteristics analysis and lightweight design of a bionic beam inspired by bamboo structures. Thin-Walled Structures, Elsevier
G Ma (2018) Comparative Experimental Study of Ground Reaction Force on Hemispherical Foot and Bionic Equinus of Quadruped Robot. 2018 IEEE 4th Information Technology and …, ieeexplore.ieee.org
AI Golodnov, YN Loginov, SI Stepanov (2018) Numeric Loading Simulation of Titanium Implant Manufactured Using 3D Printing. Solid State Phenomena, Trans Tech Publ
M Allen (2018) Floating on sound. Physics World, iopscience.iop.org
M Gorn, G Cerwenka, M Gralow, ... (2019) Industrial 3D printing for modern machine and handling systems—Potential and solutions. Journal of Laser …, lia.scitation.org
X Yu, J Fang, J Luo, X Yang, D He, Z Gou, ... (2016) 3D printing and characterization of bioactive scaffold potential for reconstructing calcified cartilage zone. Asia-Pacific Journal of …, Elsevier
R Highfield (2013) Magic box of tissues. The World Today, JSTOR
A Geierlehner, S Malferrari, ... (2019) The optimization of a 3D scanning technique applied for 3D printing of bespoke medical devices. Journal of 3D printing in …, Future Medicine
W Lin, Q Peng (2014) 3D Printing Technologies for Tissue Engineering. ASME 2014 International …, … .asmedigitalcollection.asme.org
G Dong, Q Lian, L Yang, W Mao, S Liu, C Xu (2018) Preparation and Endothelialization of Multi-level Vessel-like Network in Enzymated Gelatin Scaffolds. Journal of Bionic …, Springer, cited by 1 (1.00 per year)
P Maróti, B Kocsis, A Ferencz, M Nyitrai, ... (2019) Differential thermal analysis of the antibacterial effect of PLA-based materials planned for 3D printing. Journal of Thermal …, Springer
S Wickramasinghe, M Navarreto-Lugo, ... (2018) Applications and challenges of using 3D printed implants for the treatment of birth defects. Birth defects …, Wiley Online Library
A Thallemer, M Danzer, D Diensthuber, A Kostadinov, ... (2018) Industrial design optimisation for 3D printed biomimetic grippers in titanium alloy., dr.ntu.edu.sg
D Speranza, F Padula, B Motyl, S Tornincasa, ... (2019) Parenthood Perception Enhancement Through Interaction with 3D Printed Fetal Face Models. Advances on Mechanics …, Springer
E Provaggi, C Capelli, B Rahmani, G Burriesci, ... (2019) 3D printing assisted finite element analysis for optimising the manufacturing parameters of a lumbar fusion cage. Materials & Design, Elsevier
H Zhang, J Chen, Z Wu, G Zhu, ... (2016) Materials Forming of Dolphin Model for Hydrodynamic Research Based on the 3D Printing Technology. … Engineering and Material …, atlantis-press.com
S Morkovsky 3D Printing–Global Economy Disruptor?.
GG Wallace Fabrication of Multifunctional Structures Containing Organic Conducting Polymers: The Impact on Medical Bionics.
A Hamidi, Y Tadesse (2019) Single step 3D printing of bioinspired structures via metal reinforced thermoplastic and highly stretchable elastomer. Composite Structures, Elsevier, cited by 1 (1.00 per year)
QQ Jiang, N Yu, SM Yang (2018) A hypothesis study on bionic active noise reduction of auditory organs. Military Medical …, mmrjournal.biomedcentral.com
J Liu, T Jin, L Li, F Yang, Y Tian, ... (2018) Research on Bending and Torsion Properties of Bionic Square Continuum Robot. 2018 IEEE International …, ieeexplore.ieee.org

Y Wang, S Sun, M Xu, W Li, ... (2018) Design of a Bionic Scallop Robot Based on Jet Propulsion. 2018 IEEE International …, ieeexplore.ieee.org
Y Li, T Zhang, Y Pang, L Li, ZN Chen, W Sun (2019) 3D bioprinting of hepatoma cells and application with microfluidics for pharmacodynamic test of Metuzumab. Biofabrication, iopscience.iop.org
T Lindner, D Wyrwał, A Kubacki (2018) Autonomous Stand for 3D Printing and Machine Vision System. Conference on Automation, Springer
Y Wang, D Mao, R Dou (2016) Analysis on Patent Application in Piezoelectric Inkjet Printing Head Based on Derwent Innovations Index. The 2nd Information Technology and …, atlantis-press.com
L Donaldson (2019) Bionic mushrooms that can produce electricity., Elsevier
H Yang, YY Zhao, F Jin, J Liu, XZ Dong, ... (2019) Additive manufacturing of millimeter-scale micron-accuracy 3D structures. 9th International …, spiedigitallibrary.org
D Li, A Miao, X Jin, X Shang, H Liang, R Yang (2019) An automated 3D visible light stereolithography platform for hydrogel-based micron-sized structures. AIP Advances, aip.scitation.org
JC Davidson, AD O'Neill, TJ Kane, ... (2019) Surface methodology for 3D printed multispectral systems. … , and Applications VI, spiedigitallibrary.org
M Maurizi, J Slavič, F Cianetti, M Jerman, J Valentinčič, ... (2019) Dynamic Measurements Using FDM 3D-Printed Embedded Strain Sensors. Sensors, mdpi.com
R Kaminski, T Speck, O Speck (2019) Biomimetic 3D printed lightweight constructions: a comparison of profiles with various geometries for efficient material usage inspired by square-shaped plant stems. Bioinspiration & biomimetics, iopscience.iop.org
MR Hartings, Z Ahmed (2019) Chemistry from 3D printed objects. Nature Reviews Chemistry, nature.com
S Dias, J Dsouza, R DCruz (2019) The Bionic Bird., dspace.dbit.in
MM Fitzgerald (2015) Development and 3D Printing of Interpenetrating Network Hydrogel Materials for use as Tissue-Mimetic Models., rave.ohiolink.edu
Y Yan, M Zou, J Yao, B Yuan, Y Lin, J Jin (2017) Endurance study of bionic wheels for Mars rovers. Journal of Terramechanics, Elsevier
K Jungnickel Printing the future? An analysis of the hype and hope of rapid prototyping technology. acola.org.au
M Davtyan, Z Khanamiryan, A Hakobyan, ... Sofya Arakelyan. researchgate.net
G Cho, H Jin, M Oh, WH Choi, ... (2014) P 24.04: A novel 3D printing of fetus based on 3D ultrasound images. Ultrasound in Obstetrics & …, Wiley Online Library
Y Tong, E Kucukdeger, J Halper, E Cesewski, ... (2019) Low-cost sensor-integrated 3D-printed personalized prosthetic hands for children with amniotic band syndrome: A case study in sensing pressure distribution …. PloS one, journals.plos.org
L Wang, D Li, J He, B Lu (2018) Research center of biomanufacturing in Xi'an Jiaotong University. Bio-Design and Manufacturing, Springer
JP Desai, J Sheng, SS Cheng, X Wang, ... (2019) Toward Patient-Specific 3D-Printed Robotic Systems for Surgical Interventions. … Robotics and Bionics, ieeexplore.ieee.org
M Gallab, S Omata, K Harada, M Mitsuishi, K Sugimoto, ... (2019) Development of a Spherical Model with a 3D Microchannel: An Application to Glaucoma Surgery. Micromachines, mdpi.com
F GAO, D WEI, J ZHANG, H CAO (2017) Preparation of Entity Fracture Model in Virtual Bone-Setting System of Traditional Chinese Medicine. Shandong Journal of Traditional …, en.cnki.com.cn

A Knoll (2017) Plenary Talk 1: Bionic systems: Basic research and recent results in neurorobotics in the human brain project. … IEEE International Conference on Cyborg and Bionic …, ieeexplore.ieee.org
AD Akessa, HG Lemu, ... (2017) Mechanical Property Characterization of Additive Manufactured ABS Material Using Design of Experiment Approach. ASME 2017 …, … .asmedigitalcollection.asme.org, cited by 3 (1.50 per year)
Y Wu (2017) 3D printing of soft hydrogels incorporating functional nanomaterials., uwspace.uwaterloo.ca
F Marcolin, D Speranza, RAJ Corral Fetal face normotype from the analysis of 3D ultrasounds. webthesis.biblio.polito.it
Y Li, PY Bian (2014) Hydrogel scaffold fabricated by 3D printing & ultrasonic spraying. Advanced Materials Research, Trans Tech Publ
J Wu, S Zhang, F Qu, H Zhou, J Tang (2019) Matrix material for a new 3D-printed diamond-impregnated bit with grid-shaped matrix. International Journal of Refractory …, Elsevier
M Schmidt, H Spieth, C Haubach, C Kühne (2019) Resource efficiency through addition–additive lightweight construction for industry 4.0. 100 Pioneers in Efficient …, Springer
X Nai, L Zhijun, D Jie, Z Fuchun (2015) Study on the Application of 3D Printing in the Teaching Reform of Plastic Processing and Mould Design Course Group. The Science Education Article …, en.cnki.com.cn
L Fan, Z Jianhui, T Ming, C Chaobin, ... (2018) Experimental study of valveless piezoelectric pump with raindrop-shaped tubes. Proceedings of the 2018 …, dl.acm.org
J Rubi, RJ Hemalatha, JA Dhivya, ... (2019) 3D Printed Eco Friendly Smart Prosthetic Arm with rotating Wrist. Indian Journal of …, indianjournals.com
GG Wallace (2015) From science fiction to reality: the dawn of the biofabricator., ro.uow.edu.au
A Dal Maso, F Cosmi (2019) 3D-printed ankle-foot orthosis: a design method. Materials Today: Proceedings, Elsevier
C Yang, BJ Venton (2017) High performance, low cost carbon nanotube yarn based 3D printed electrodes compatible with a conventional screen printed electrode system. 2017 IEEE International Symposium on …, ieeexplore.ieee.org
B YAN, G ZHANG, Z WU, J XU, Y YANG, ... (2014) Experimental study on accurate placement of the lumbar pedicle screws assisted by 3D printing navigation module. Chinese Journal of …, en.cnki.com.cn
POP OPORNIC (2019) APPLICATION OF A THERMOPLASTIC POLYURETHANE/POLYLACTIC ACID COMPOSITE FILAMENT FOR 3D-PRINTED PERSONALIZED ORTHOSIS. Materiali in tehnologije, researchgate.net
A Sadeqi, HR Nejad, RE Owyeung, ... (2019) Three dimensional printing of metamaterial embedded geometrical optics (MEGO). Microsystems & …, nature.com
I Senior Design, A Garcia, J Morales, T Smahliuk, ... Multiplex Bionic. eecs.ucf.edu
Z Wang, J Cao, Q Hao, F Zhang (2019) Combining compound eyes and human eye: a hybrid bionic imaging method for FOV extension and foveated vision. … International Symposium on …, spiedigitallibrary.org
X Zhu, D Ran, C Xiang, J Zhang, G Li, ... (2018) Design, Analysis and Experiments of Bionic Hexapod Robot with Multilayer C-shape Legs for Unstructured Terrain. 2018 13th World …, ieeexplore.ieee.org
FE Sandnes, LC Paschoarelli Manufacturing Technology in Rehabilitation Practice: Implications for Its Implementation in Assistive Technology Production. … and 3D Prototyping: Proceedings of the …, books.google.com

M Mansour, K Tsongas, D Tzetzis (2019) Measurement of the mechanical and dynamic properties of 3D printed polylactic acid reinforced with graphene. Polymer-Plastics Technology …, Taylor & Francis
Z Azhar (2015) Development of a Rapid Prototyped Hand Exoskeleton for use in Patient Rehabilitation., era.library.ualberta.ca
YL Kong (2019) Multiscale additive manufacturing of electronics and biomedical devices. Micro-and Nanotechnology Sensors, Systems, and …, spiedigitallibrary.org
PJK Hezwani, S Piotr A Portable 3D Printer System for the Diagnosis and Treatment of Multidrug-Resistant Bacteria. cyberleninka.org
NN Baranowska (2018) The Intersection of 3D Printing and Trademark Law. J. Intell. Prop. Info. Tech. & Elec. Com. L., HeinOnline
E Gawell, A Nowak, W Rokicki (2019) Searching for Bionics Structural Forms Optimization. IOP Conference Series …, iopscience.iop.org
T Liu, Y Wang, K Lee (2017) 3D Printable Origami Twisted Tower: Design, Fabrication, and Robot Embodiment.
I Asanovic, H Millward, A Lewis (2019) Development of a 3D scan posture-correction procedure to facilitate the direct-digital splinting approach. Virtual and Physical Prototyping, Taylor & Francis
A Motealleh, P Dorri, AH Schäfer, NS Kehr (2019) 3D bioprinting of triphasic nanocomposite hydrogels and scaffolds for cell adhesion and migration. Biofabrication, iopscience.iop.org
C Hayes (2017) Think and it's done [prosthetic design and production]. Engineering & Technology, IET
CY Lee, KF Lei, CL Ku, ... (2016) Development of bionic invasion membrane for the study of multiple sclerosis. 2016 IEEE 11th Annual …, ieeexplore.ieee.org
KH Persis, P Banumathi, B Gayathri, B Vinodhini ADVANCED ROBOTIC ARM–FOR VERSATILE AND PLIANT APLICATIONS. ripublication.com
IFP de Carvalho Filho, FO Medola, FE Sandnes, ... (2019) Manufacturing Technology in Rehabilitation Practice: Implications for Its Implementation in Assistive Technology Production. … Conference on Applied …, Springer
R Han (2017) 3D Printed Biocatalytic Silica Hydrogel Flow-Through Reactor For Atrazine Degradation., conservancy.umn.edu
B VENKATESH, MA KUMAR DESIGN AND DEVELOPMENT OF WIRELESS OPERATED LOW COST PROSTHETIC HAND BY FUSED DEPOSITION MODELING. researchgate.net
J Maric, F Rodhain, Y Barlette (2016) 3D printing trends and discussing societal, environmental and ethical implications., hal.archives-ouvertes.fr
S Mo, SHS Leung, ZYS Chan, LKY Sze, ... (2019) The biomechanical difference between running with traditional and 3D printed orthoses. Journal of sports …, Taylor & Francis
S Pande, F Shaikh, A Panchal, A Mateen THE DEVELOPMENT, APPLICATION AND BENEFITS OF 3-D PRINTING TECHNOLOGY.
F Froes (2019) Combining additive manufacturing with conventional casting and reduced density materials to greatly reduce the weight of airplane components such as passenger …. Additive Manufacturing for the Aerospace Industry, Elsevier
J Dong, H Zhang, S Liu (2019) 3D printed bio-inspired sealing disc of pipeline inspection gauges (PIGs) in small diameter pipeline. Journal of Natural Gas Science and Engineering, Elsevier
J Dong (2017) TOADFISH BEHAVIORAL ROBOT FOR FISH AGGRESSION STUDY., people.ece.cornell.edu

A Borboni, T Mikołajczyk, ... (2016) Recent Advances in Biomedical Applications. Applied bionics and …, downloads.hindawi.com
M Ariyanto, R Ismail, JD Setiawan, EP Yuandi (2019) Anthropomorphic transradial myoelectric hand using tendon-spring mechanism. TELKOMNIKA, researchgate.net
PK Paritala, T Yarlagadda, R Sreeram, ... (2017) Impact of digital manufacturing on health care industry., eprints.qut.edu.au
MNR Khan, MM Hasan, MZ Islam Customized Prosthetic Hands for Children.
S Hemleben (2017) Modeling a Spectrum of 3D Printed Materials for Soft Robots., ir.library.oregonstate.edu
S CHEN, Z SUN (2017) Research and Application of the 3-D Printing Bone Repair Technology Based on Biocompatibility Material. China Plastics Industry, en.cnki.com.cn
JP Ferdinand, H Flämig, U Petschow, ... (2016) Assessing the Environmental Impact of Decentralized Value-Chain Patterns Involving 3D Printing Technologies—A Comparative Case Study. The Decentralized and …, Springer
G Wallace CBC SEMINAR ANNOUNCEMENT.
Y Jiang, D Shen, M Liu, Z Ma, P Zhao, ... (2019) Fabrication of graphene/polyimide nanocomposite-based hair-like airflow sensor via direct inkjet printing and electrical breakdown. Smart Materials and …, iopscience.iop.org
D Su, X Li, Y Luo (2016) Anisotropic flexible mechanical design of prosthetic socket liner. International Journal of Applied …, content.iospress.com
C Torres Romero (2019) 3D Printed Prostheses and Organs., theseus.fi
JAA Corveira (2017) Design and development of a Soft Body-Actuated 3D printed prosthetic hand., eg.uc.pt
MV Sarakinioti, M Turrin, M Teeling, P de Ruiter, ... (2017) Spong3d: 3D printed facade system enabling movable fluid heat storage. …, superheroscitech.tudelft.nl
X Duan, H Fan, F Wang, P He, L Yang (2019) Application of 3D-printed Customized Guides in Subtalar Joint Arthrodesis. Orthopaedic surgery, Wiley Online Library
Y Zhang, S Wang, X Wang, ... (2018) Design and Control of Bionic Manta Ray Robot With Flexible Pectoral Fin. 2018 IEEE 14th …, ieeexplore.ieee.org
G Yaoyuneyong (2016) How are Bodies Shaped by Technology, and How is Technology Shaped by Bodies?., aquila.usm.edu
Z Liu, L Zhao, P Yu, T Yang, N Li, ... (2018) A Wearable Bionic Soft Exoskeleton Glove for Stroke Patients. 2018 IEEE 8th Annual …, ieeexplore.ieee.org
GFR Radig (2017) Redesigning Medical Instruments Using 3D Metal Printing: New tools for cardiology thanks to additive manufacturing. Laser Technik Journal, Wiley Online Library
GG Wallace, S Beirne (2013) Additive BIO Fabrication: Impact, Opportunities and Challenges., ro.uow.edu.au
MM Xiucan Li, Y Wang, Y Zhao, J Liu, S Xiao, K Mao (2017) SPINE An International Journal for the study of the spine Publish Ahead of Print.
N Kojima, A Inoue, T Hoshi (2015) DIVE: Fluid Display for 3D CG Manipulation with Visual Continuity. Proceedings of the Annual Meeting of the …, dl.acm.org
OS Fenton, M Paolini, JL Andresen, ... (2019) Outlooks on Three-Dimensional Printing for Ocular Biomaterials Research. Journal of Ocular …, liebertpub.com
J Cheung, G Wolbring (2014) Historical analysis of Canadian newspaper coverage of organ transplant and organ donation., prism.ucalgary.ca
О Petrenko, О Myronov, K Kugai (2016) New technologies in our life. Наукові розробки молоді на …, er.knutd.edu.ua

C Ma, D Gu, K Lin, D Dai, M Xia, J Yang, ... (2019) Selective laser melting additive manufacturing of cancer pagurus's claw inspired bionic structures with high strength and toughness. Applied Surface Science, Elsevier
KJA Brookes (2018) Europe salutes metal-based AM. Metal Powder Report, Elsevier
P Damodaram, R Mitra (2018) Bio-Mimetic Design with 3D Printable Composites., openprairie.sdstate.edu
XIE Fangli (2018) 3D pen. US Patent App. 15/868,990, Google Patents
M Fei (2018) Application Status and Prospect of Three-Dimensional Printing Technology in the Field of Medical Devices., ojs.bbwpublisher.com
B Data Living with a prosthesis that learns: A case-study in translational medicine. internetmedicine.com
MSE Livne (2016) Breakthrough Technologies: Shaping the Future (SPEC203)., overseas.huji.ac.il
H Shinoda, S Azukizawa, K Maeda, ... (2019) Bio-Mimic Motion of 3D-Printed Gel Structures Dispersed with Magnetic Particles. Journal of The …, jes.ecsdl.org
R Vdović, M Pap (2014) Digital Fabrication in the Field of Architecture. PLACES AND TECHNOLOGIES 2014, Citeseer
T Rajan, JD Valliath (2018) The Development of an Electroencephalography (EEG)-Derived, 3D-Printed Brain-Computer Interface (BCI) NeuroProsthesis Utilizing Machine Learning for Chronic …. Available at SSRN 3274806, papers.ssrn.com
J Moritz, A Seidel, B Braun, A Brandao, ... (2019) Functional integration approaches via laser powder bed processing. Journal of Laser …, lia.scitation.org
ME Orme, M Gschweitl, R Vernon, ... (2016) A demonstration of additive manufacturing as an enabling technology for rapid satellite design and fabrication. … , Long Beach, CA …, researchgate.net, cited by 5 (1.67 per year)
P Jie, S Zhenyun, W Tianmiao Variable-Modal SMA-Driven Spherical Robot. SCIENCE CHINA Technological …, engine.scichina.com
L Lenner (2016) Engine Redesign Utilizing 3D Sand Printing Techniques Resulting in Weight and Fuel Savings., rave.ohiolink.edu
R Angold, A Preuss, N Fleming, ... (2018) Systems and Methods for Creating Custom-Fit Exoskeletons. US Patent App. 15 …, Google Patents
RT Xavier, AA de Carvalho, E Rohmer, F Castro, ... (2019) Upper Limb Prosthesis for Patients with Congenital or Acquired Deformity. XXVI Brazilian Congress …, Springer
R Neethan, S Nidershan, V Mugilgeethan, ... (2019) A study of three-dimensional (3-D) printed prosthetic upper limb models in local context. Materials Today …, Elsevier
AM Román-Casares, O García-Gómez, ... (2018) Prosthetic Limb Design and Function: Latest Innovations and Functional Results. Current Trauma …, Springer
CT Coarsey, A Berger, C Medina, M Pavlovic, ... OPEN-SOURCE DEVICE FOR VARIABLE ULNAR PROMINENCE. bionicglove.org
A Surányi, Z Kozinszky, A Molnár, ... (2014) P 24.03: Placental volume relative to fetal weight estimated by 3D sonography in diabetic pregnancies. … in Obstetrics & …, Wiley Online Library
Z Xu, Q You, J Li, P Liu, N Wang, ... (2019) 3D Printed Biphasic Calcium Phosphate Scaffolds for Bone Tissue Engineering. Journal of Biobased …, ingentaconnect.com
MS Kim, VT Nguyen, SC Ko, WK Jung (2017) Fabrication and osteogenic effects of three-dimensional scaffold with poly (ε -caprolactone)/abalone peptide/collagen for bone tissue regeneration. 한국수산과학회 양식분과 학술대회

Q Wu, Y Pan, H Wan, N Hu, ... (2019) Research progress of organoids-on-chips in biomedical application. Chinese Science …, engine.scichina.com
MF Saqib, A Islam, MLA Bari, ... (2018) Gesture Controlled Prosthetic Arm with Sensation Sensors. … for Convergence in …, ieeexplore.ieee.org
PY Tsai, CC Huang, YH Wen, HH Shen, YH Lin, ... (2017) Bionic fixing apparatus. US Patent …, Google Patents
F ORTES, HK SURMEN, YZ ARSLAN (2016) A BIOMECHATRONIC APPLICATION ON PROSTHETICS FOR UNDERGRADUATE ENGINEERING STUDENTS. The Eurasia Proceedings of …, dergipark.org.tr
T Georgiou, D Asnaghi, A Liang, ... The Sparthan 3D Printed Exo-Glove. Journal of Medical …, … .asmedigitalcollection.asme.org
PN Hsieh, YW Shen, LJ Fuh (2018) Gowtham Raj, Murugesan. K, Peter John. The Journal of Indian …, search.proquest.com
N Guo, B Yang, X Ji, Y Wang, L Hu, ... (2019) Intensity-based 2D-3D registration for an ACL reconstruction navigation system. … International Journal of …, Wiley Online Library
J Wang, Z Wang, Z Song, L Ren, ... (2019) Biomimetic Shape–Color Double-Responsive 4D Printing. Advanced Materials …, Wiley Online Library
JS Artal-Sevil, A Acón, JL Montañés, ... (2018) Design of a Low-Cost Robotic Arm controlled by Surface EMG Sensors. … Applied to Electronics …, ieeexplore.ieee.org
R Xu, Z Wang, Z Ren, T Ma, Z Jia, ... (2019) Comparative Study of the Effects of Customized 3D printed insole and Prefabricated Insole on Plantar Pressure and Comfort in Patients with Symptomatic …. … medical journal of …, ncbi.nlm.nih.gov
G Haba, R Oyama, Y Kaido, T Kanasugi, ... (2014) P 24.02: To visualise multiple direction of the fetal skeletal dysplasia using 3D slicer software: a new approach of the fetal MRI. … in Obstetrics & …, Wiley Online Library
FI Birouas, A Nilgesz (2017) PROTOTYPING ROBOTIC MEDICAL REHABILITATION DEVICES. Revista de Tehnologii Neconventionale, researchgate.net
J Torner Ribé, F Alpiste Penalba, ... (2017) Robotic arm controlled through vision and biomechanical sensors. Dyna ingeniería e …, upcommons.upc.edu
J Wang, X Mu, D Li, C Yu, X Cheng, ... (2019) Modeling and Application of Planar-to-3D Structures via Optically Programmed Frontal Photopolymerization. Advanced Engineering …, Wiley Online Library
M Iliescu, L Vladareanu, C Frant, ... (2019) Research on upper limb biomechanical system. … of Engineering and …, pen.ius.edu.ba
A Otte (2018) Smart neuroprosthetics becoming smarter, but not for everyone?. EClinicalMedicine, thelancet.com
J Li, D Chen, Y Zhang, Y Yao, Z Mo, L Wang, ... (2019) Diagonal-symmetrical and Midline-symmetrical Unit Cells with Same Porosity for Bone Implant: Mechanical Properties Evaluation. Journal of Bionic …, Springer
P Bohse A MAN-MADE MAN.
S Bijadi, T Zalusky, J O'Neill, T Kowalewski Feasibility of Additive Manufacturing Method for Developing Stretchable Electronics for Bio-integrated Devices. me.umn.edu
S Bagassi, F Lucchi, F De Crescenzio, F Persiani GENERATIVE DESIGN: ADVANCED DESIGN OPTIMIZATION PROCESSES FOR AERONAUTICAL APPLICATIONS. icas.org
MR Patel (2007) CONCEPTUAL DESIGN: A CUSTOMIZABLE, 3D PRINTED BODY-POWERED. Technology, academia.edu
GG Wallace ISGD-5.

G da Silva Bertolaccini, FO Medola, ... Evaluation of Orthosis Rapid Prototyping During the Design Process: Analysis of Verification Models. Advances in Additive …, books.google.com
RR Dancel Case Study Paper on Additive Manufacturing (3D Printing Technology). researchgate.net
T Tang, Z Zhang, H Ni, J Deng, ... (2016) Femur Model Reconstruction Based on Reverse Engineering and Rapid Prototyping. … Engineering and Control …, World Scientific
A Nazo (2015) Can Reconstruction of the Human Body Reveal a New Transition Phase for the Human Species into a Yet to be Known Living Form? Posthumanism: Future …. 2015 IEEE International Symposium on Mixed and …, ieeexplore.ieee.org
MS Ruma, H Bradley, E Lovato, ... (2014) P 24.01: Patient satisfaction with a novel portable ultrasound system. Ultrasound in Obstetrics …, Wiley Online Library
W Xu, H Zhang, N Zheng, H Yuan (2018) Design and Experiments of a Compliant Adaptive Grasper Based on Fish Fin Structure. 2018 IEEE International …, ieeexplore.ieee.org
B Schramm, L Risse, JP Brüggemann, ... (2018) Overview of structural mechanical investigations on additively manufactured medical products. Journal of 3D printing …, Future Medicine
B Lippert, G Leuteritz, R Lachmayer (2017) An approach to implement design for additive manufacturing in engineering studies. … ICED 17) Vol 5: Design for …, designsociety.org
SFW Xian-fu, H Bei-qing, S Feng-xian, W Xian-fu Study on New Material of Grating which Used in Three-dimensional Printing. imaging.org
AJ Cowin (2018) Wound innovation: Now and in the future. Wound Practice & Research: Journal of the …, search.informit.com.au
MM Kelkis (2019) KIT-Karlsruhe School of Optics & Photonics-News Archive., ksop.kit.edu
S Kaur (2016) Additive manufacturing of upper and lower prosthetic limbs., search.proquest.com
C Thompson (2016) Technology: Tech of tomorrow. Medicus, search.informit.com.au
P Evans (2018) Twenty-first-century learning. Codify, taylorfrancis.com
EV Maksutina, A N Makarov, IA Sokolova, ... (2018) Neoindustrial paradigm of Russia based on fourth industrial revolution technologies and human capital development. … " Economy in the …, atlantis-press.com
A Karle Art of the 4th Industrial Revolution and its Contributions to Humankind. amykarle.com
J Cronin, G D'Orsi, K Chandler (2017) Breaking the Barriers to Access: Providing High-Tech Prostheses to Developing Countries.. Proceedings of the …, search.ebscohost.com
A Agkathidis Implementing Biomorphic Design. researchgate.net
ALM Ferrari, ADP dos Santos, ... (2019) Evaluation of Orthosis Rapid Prototyping During the Design Process: Analysis of Verification Models. … Conference on Applied …, Springer
A Farha, M Muhtadi, RI Morshed (2018) 3D fabrication of food through software implementation for patients of various diseases and dysphagia., dspace.bracu.ac.bd
B Wałpuski, B Podsiadły, JJ Krzemiński, ... (2019) Conductive Paths and Connections on Polymer Substrates for Structural Electronics. … and Computer Science, pp.bme.hu
MFM Fareezull, P Manugari, MRM Rafnor, L Zuraidi, ... DESIGN AND DEVELOPMENT OF QUADRUPED ROBOT, PART 5. researchgate.net
W Rokicki, A Stefańska (2019) Architectural Ephemerids in Terms of Generative and Parametric Design., ijscer.com
XH Arvelo Rodriguez (2019) Designing for Sustainable Bicycle Manufacturing., scholarworks.rit.edu

XHA Rodríguez (2019) Designing for Sustainable Bicycle Manufacturing., search.proquest.com
ÅK Jakobsson, R Crameri, ... (2014) Innovation and emergent technologies for defence—Logic and creativity. … on System of …, ieeexplore.ieee.org
J Liu, C Yan (2018) 3D printing of scaffolds for tissue engineering. 3D Printing, books.google.com
MC McAlpine, MF Semmelhack, HA Stone (2015) Bio-Nanocombinatoric Synthesis and Quorum Sensing., apps.dtic.mil
CM Saratti, GT Rocca, I Krejci (2019) The potential of three-dimensional printing technologies to unlock the development of new'bio-inspired'dental materials: an overview and research roadmap. Journal of prosthodontic research, jstage.jst.go.jp
KT Richardson (2018) Design and Analysis of a 3D-printed, Thermoplastic Elastomer (TPE) Spring Element for Use in Corrective Hand Orthotics., uknowledge.uky.edu
LIU Dongsheng, Z Hongbin, ... (2018) A Flytrap-inspired Soft Manipulator Driven by Single Airbag. … WRC Symposium on …, ieeexplore.ieee.org
Q He, D Vokoun, Q Shen (2018) Biomimetic Actuation and Artificial Muscle. Applied bionics and …, downloads.hindawi.com
F Wang, Z Xing, X Wang, J Zhao (2019) A Method to Fabricate Complex Structure for Variable Stiffness Manipulators Based on Low-Melting-Point Alloy. 2019 2nd IEEE International …, ieeexplore.ieee.org
K Moiduddin, SH Mian, H Alkhalefah, U Umer (2019) Digital Design, Analysis and 3D Printing of Prosthesis Scaffolds for Mandibular Reconstruction. Metals, mdpi.com
L Gasman (2019) Additive aerospace considered as a business. Additive Manufacturing for the Aerospace Industry, Elsevier
H Luqman, P Manugari, MRM Rafnor, L Zuraidi, ... DESIGN AND DEVELOPMENT OF QUADRUPED ROBOT, PART 4. researchgate.net
D Werner, SA Alawi … Extremity Amputation and Bionic Prosthesis Supply after Disseminated Intravascular Coagulation: A Follow-Up on Functionality and Quality of Life after Bionic …. wjps.ir
A Gohritz, K Knobloch, PM Vogt, H Plastic TECHNICAL RIGHT BELOW ELBOW AMPUTEE ISSUES. swisswuff.ch
V Tavakoli (2018) A LOW PROFILE TRANSSEPTAL FULLY ATRIAL SCAFFOLD FOR TRANS-CATHETER MITRAL VALVE REPLACEMENT: A FEASIBILITY STUDY. Journal of the American College of Cardiology, onlinejacc.org
RP Harrison Installation (generative digital environment) Project by Tanja Vujinović Production: Ultramono and SciArt Lab IJS, 2019 3D objects, generative modelling …. ultramono.org
S Omata, M Gallab, K Harada, ... (2018) Eye Surgery Simulator for the Training of Minamally Invassive Glaucoma Surgery Skills*. … Cyborg and Bionic …, ieeexplore.ieee.org
P Li, Q Wu, Y He, J Yuan, J Liu, K Liu, ... (2019) Development of Biomass Materials of the Byproducts of Sponge City Construction in Changde. … on Engineering and …, dpi-proceedings.com
E Ottaviano, P Rea, A Grandinetti (2018) Mechatronic Design of a Low-Cost Control System for Assisting Devices. International Conference on Innovation …, Springer
A Kumar (2018) Manufacturing Letters., researchgate.net
M Liarokapis, KA Lamkin-Kennard, MB Popovic (2019) Biomechatronics: A New Dawn., researchgate.net

W Qin, Z Wang, Y Lu, M Chan Use of Motion Sensors for Improved Headset User Interface. pdfs.semanticscholar.org
M Saffarzadeh, GJ Gillispie, P Brown SELECTIVE LASER SINTERING (SLS) RAPID PROTYTPING TECHNOLOGY: A REVIEW OF MEDICAL APPLICATIONS. researchgate.net
AI Laisha (2015) Science and immortality., Б Г У И Р
L Bonanomi (2017) Plant-Based Multifunctional Bionic Materials., research-collection.ethz.ch
R had studied Aeronautical (2012) The Airbus 'Joint family'product line., search.proquest.com
II Livshitz, PA Lontsikh, EP Kunakov, ... (2018) Improvement of the Activities of Machine-Building Enterprises Through the Use of Digital Technologies. 2018 IEEE …, ieeexplore.ieee.org
F Liravi, E Toyserkani (2018) Additive manufacturing of silicone structures: A review and prospective. Additive Manufacturing, Elsevier, cited by 7 (7.00 per year)
W ZHOU, Z LI, F LU, Q WANG, W CHEN, ... (2016) Application of 3D Printing Technology on Food Processing Field. Agricultural Science & …, en.cnki.com.cn
A du Plessis, C Broeckhoven (2018) Looking deep into nature: A review of micro-computed tomography in biomimicry. Acta biomaterialia, Elsevier, cited by 9 (9.00 per year)
Z Han, L Liu, K Wang, H Song, D Chen, Z Wang, ... (2018) Artificial hair-like sensors inspired from nature: A review. Journal of Bionic …, Springer, cited by 5 (5.00 per year)
M Banna, K Bera, R Sochol, L Lin, H Najjaran, R Sadiq, ... (2017) 3D printing-based integrated water quality sensing system. Sensors, mdpi.com, cited by 7 (3.50 per year)
D Kwan (2017) Potential applications of three-dimensional bioprinting in Regenerative Medicine. cmj, cambridgemedicine.org
B Ahuja, M Karg, M Schmidt (2015) Additive manufacturing in production: challenges and opportunities. Laser 3d manufacturing II, spiedigitallibrary.org, cited by 21 (5.25 per year)
A Manero, J Sparkman, M Dombrowski, ... (2018) Developing and Training Multi-gestural Prosthetic Arms. … Conference on Virtual …, Springer
MA Peregrina, AR Poveda (2018) Design and development of an open antropomorphic robotic hand development system. … Conference on Cyborg and Bionic …, ieeexplore.ieee.org
CP Radtke, N Hillebrandt, ... (2018) The Biomaker: an entry-level bioprinting device for biotechnological applications. Journal of Chemical …, Wiley Online Library, cited by 4 (4.00 per year)
Y Deeni, T Beccari, M Dundar, JS Gartland, M Maffia, ... 8 Trends Reshaping Biotechnology. hplusmagazine.com
K Füzesi, Á Makra, M Gyöngy (2017) A stippling algorithm to generate equivalent point scatterer distributions from ultrasound images. Proceedings of Meetings on …, asa.scitation.org
A Tan, R Chawla, G Natasha, ... (2016) Nanotechnology and regenerative therapeutics in plastic surgery: the next frontier. Journal of Plastic …, Elsevier, cited by 9 (3.00 per year)
R Koontz (2019) Bio-Inspired Transportation and Communication., books.google.com
Y He, L Shi, S Guo, S Pan, Z Wang (2016) Preliminary mechanical analysis of an improved amphibious spherical father robot. Microsystem Technologies, Springer, cited by 31 (10.33 per year)
S Panzavolta, P Torricelli, S Amadori, ... (2013) 3D interconnected porous biomimetic scaffolds: In vitro cell response. … Research Part A: An …, Wiley Online Library, cited by 36 (6.00 per year)

JH Low, MH Ang, CH Yeow (2015) Customizable soft pneumatic finger actuators for hand orthotic and prosthetic applications. 2015 IEEE International …, ieeexplore.ieee.org, cited by 19 (4.75 per year)
XH Hu, X Zhang, M Liu, YF Chen, P Li, WH Pei, ... (2014) A flexible capacitive tactile sensor array with micro structure for robotic application. Science China …, Springer, cited by 18 (3.60 per year)
ICO Rounds, YB Transfusion The Surprising Future of Artificial Organs.
A Ghobadian, I Talavera, A Bhattacharya, ... (2018) Examining legitimatisation of additive manufacturing in the interplay between innovation, lean manufacturing and sustainability. International Journal of …, Elsevier, cited by 11 (11.00 per year)
K Black-Bain (2015) An update in robotics in outpatient rehab. University of Utah Health Care, Salt Lake City, medicine.utah.edu, cited by 1 (0.25 per year)
F Fu, Z Qin, C Xu, X Chen, R Li, L Wang, ... (2017) Magnetic resonance imaging-three-dimensional printing technology fabricates customized scaffolds for brain tissue engineering. Neural regeneration …, ncbi.nlm.nih.gov, cited by 11 (5.50 per year)
MX Gan, CH Wong (2016) Practical support structures for selective laser melting. Journal of Materials Processing Technology, Elsevier, cited by 66 (22.00 per year)
CJ Thrasher, JJ Schwartz, ... (2017) Modular elastomer photoresins for digital light processing additive manufacturing. ACS applied materials & …, ACS Publications, cited by 19 (9.50 per year)
R Jalili, A Kanneganti, MI Romero-Ortega, ... (2017) Implantable electrodes. Current Opinion in …, Elsevier, cited by 4 (2.00 per year)
M Singh (2017) Conformal Additive Manufacturing for Organ Interface., vtechworks.lib.vt.edu
ME Orme, M Gschweitl, ... (2017) Designing for additive manufacturing: lightweighting through topology optimization enables lunar spacecraft. Journal of …, … .asmedigitalcollection.asme.org, cited by 12 (6.00 per year)
J Coles-Black, J Chuen, WJ Choy, ... (2018) MEETING ABSTRACTS Open Access. ANZ J …, threedmedprint.biomedcentral.com
Y Wang, J He, Y Liu, S Pang, W Zhang, ... (2012) Direct Imprinting of Three-Dimensional Microfluidic Scaffolds. Journal of Xi'an Jiaotong …, en.cnki.com.cn
Y Deeni, T Beccari, M Dundar, JS Gartland, ... (2014) Novel technologies and their applications in biotechnology. European Journal of …, researchgate.net
VS KAMLYUK (2018) Т Е Х Н И Ч Е С К И Е Н А У К И. П Е Р И О Д И Ч Е С К И Й Ж У Р Н А Л Н А У Ч Н Ы Х Т Р У Д О В …, elvestnik.com
SV D'yachenko, LA Lebedev, MM Sychev, ... (2018) Physicomechanical Properties of a Model Material in the Form of a Cube with the Topology of Triply Periodic Minimal Surfaces of the Gyroid Type. Technical Physics, Springer
D Cafolla, M Russo, G Carbone (2019) CUBE, a Cable-driven Device for Limb Rehabilitation. Journal of Bionic Engineering, Springer, cited by 1 (1.00 per year)
F Moreira, A Abundis, M Aguirre, J Castillo, ... (2018) An inchworm-inspired robot based on modular body, electronics and passive friction pads performing the two-anchor crawl gait. Journal of Bionic …, Springer, cited by 1 (1.00 per year)
C Soler (2017) Health 4.0 oriented to non-surgical treatment. Iberian Robotics conference, Springer, cited by 1 (0.50 per year)
A Zatopa, S Walker, Y Menguc (2018) Fully soft 3D-printed electroactive fluidic valve for soft hydraulic robots. Soft robotics, liebertpub.com, cited by 7 (7.00 per year)

P Hilkens, A Bronckaers, J Ratajczak, P Gervois, ... (2017) The angiogenic potential of DPSCs and SCAPs in an in vivo model of dental pulp regeneration. Stem cells …, hindawi.com, cited by 17 (8.50 per year)
A Radosh, W Kuczko, R Wichniarek, ... (2017) Prototyping of cosmetic prosthesis of upper limb using additive manufacturing technologies. Advances in Science …, yadda.icm.edu.pl, cited by 3 (1.50 per year)
B Hu, G Jin, Z Liu, P Wang, X Li, ... (2018) A Multi-actuator Soft Robot Inspired by Young Tiger Beetle. 2018 13th World …, ieeexplore.ieee.org
R Berkowitz (2017) 3D printing, in the flesh: Although not yet as established as other additive manufacturing applications, printing human tissue is poised to significantly advance the field …. Electro Optics, go.galegroup.com
S Shivalkar, S Singh (2017) Solid freeform techniques application in bone tissue engineering for scaffold fabrication. Tissue engineering and regenerative medicine, Springer, cited by 10 (5.00 per year)
Y Huang, JY Li (2014) Generative product design inspired by natural information. International Conference on Human Interface and the …, Springer, cited by 1 (0.20 per year)
G Mapili, Y Lu, S Chen, K Roy (2005) Laser-layered microfabrication of spatially patterned functionalized tissue-engineering scaffolds. Journal of Biomedical …, Wiley Online Library, cited by 189 (13.50 per year)
M Pasternak (2017) Component for producing elastic elements. US Patent App. 15/518,516, Google Patents
T Chen, Y Zhang, H Zuo, Y Zhao, ... (2016) Repairing skull defects in children with nano-hap/collagen composites: A clinical report of thirteen cases. Translational …, journals.sagepub.com, cited by 1 (0.33 per year)
R Hudak, M Penhaker, J Majernik (2012) Biomedical Engineering: Technical Applications in Medicine., books.google.com, cited by 8 (1.14 per year)
F Simone, A York, S Seelecke (2015) Design and fabrication of a three-finger prosthetic hand using SMA muscle wires. … , and Bioreplication 2015, spiedigitallibrary.org, cited by 17 (4.25 per year)
Y Wang, J Liu, D Zhu, H Chen (2018) Active Tube-Shaped Actuator with Embedded Square Rod-Shaped Ionic Polymer-Metal Composites for Robotic-Assisted Manipulation. Applied bionics and biomechanics, hindawi.com
VR Shulunov (2018) Enhanced roll powder sintering additive manufacturing technology. International Journal of Automation and Smart …, ausmt.org, cited by 2 (2.00 per year)
Y Zheng, X Li, L Tian, G Li (2018) Design of a Low-Cost and Humanoid Myoelectric Prosthetic Hand Driven by a Single Actuator to Realize Basic Hand Functions. … Conference on Cyborg and Bionic …, ieeexplore.ieee.org
AM El Kady, AE Mahfouz, ... (2010) Mechanical design of an anthropomorphic prosthetic hand for shape memory alloy actuation. 2010 5th Cairo …, ieeexplore.ieee.org, cited by 14 (1.56 per year)
JT Muth, DM Vogt, RL Truby, Y Mengüç, ... (2014) Embedded 3D printing of strain sensors within highly stretchable elastomers. Advanced …, Wiley Online Library, cited by 679 (135.80 per year)
Z Xu, V Kumar, E Todorov (2013) A low-cost and modular, 20-DOF anthropomorphic robotic hand: design, actuation and modeling. 2013 13th IEEE-RAS International …, ieeexplore.ieee.org, cited by 27 (4.50 per year)
C Ades, I Gonzalez, M AlSaidi, M Nojoumian, O Bai, ... Robotic Finger Force Sensor Fabrication and Evaluation Through a Glove. pdfs.semanticscholar.org

P Manugari, L Zuraidi, MRM Rafnor, H Luqman, ... DESIGN AND DEVELOPMENT OF QUADRUPED ROBOT PART. researchgate.net
C LI, Y HOU, P LI, Y ZHANG (2015) Four Axis Motion Optimal Coordinated Control of Three-Dimensional Molding Machine. Small & Special Electrical Machines, en.cnki.com.cn
M Muthusamy, S Safaee, R Chen (2018) Additive Manufacturing of Overhang Structures Using Moisture-Cured Silicone with Support Material. Journal of Manufacturing and …, mdpi.com, cited by 2 (2.00 per year)
B Harl, J Predan, N Gubeljak, ... (2017) On configuration-based optimal design of load-carrying lightweight parts. International Journal of …, researchgate.net, cited by 4 (2.00 per year)
JA Belgodere, CT King, JB Bursavich, ... (2018) Engineering breast cancer microenvironments and 3D bioprinting. … in bioengineering and …, frontiersin.org, cited by 5 (5.00 per year)
Z Lei, P Wu (2019) Adaptable polyionic elastomers with multiple sensations and entropy-driven actuations for prosthetic skins and neuromuscular systems. Materials Horizons, pubs.rsc.org, cited by 2 (2.00 per year)
O Tsaruk Cyber threats and opportunities embedded in the Fourth Industrial Revolution. uisgcon.org
Z Teng, G Xu, R Liang, M Li, S Zhang, ... (2018) Design of an Underactuated Prosthetic Hand with Flexible Multi-Joint Fingers and EEG-Based Control. … Cyborg and Bionic …, ieeexplore.ieee.org
J Cernohorsky, A Richter, ... (2018) Mechatronic Design of Rehabilitation Brace. 2018 IEEE 20th …, ieeexplore.ieee.org
A Ellery (2017) Bioinspiration lessons from a self-replicating machine concept in a constrained environment. 2017 IEEE International Conference on Robotics and …, ieeexplore.ieee.org
E Braund, ER Miranda (2017) On building practical biocomputers for real-world applications: Receptacles for culturing slime mould memristors and component standardisation. Journal of Bionic Engineering, Elsevier, cited by 4 (2.00 per year)
W Sun, JH Nam, AL Darling, S Khalil (2014) Method and apparatus for computer-aided tissue engineering for modeling, design and freeform fabrication of tissue scaffolds, constructs, and devices. US Patent 8,639,484, Google Patents, cited by 123 (24.60 per year)
YH Liu, XZ Dong, YY Zhao, ML Zheng, ... (2019) Three-dimensional micro-cones fabricated via two-photon photolithography. 9th International …, spiedigitallibrary.org
J He, M Mao, Y Liu, J Shao, Z Jin, ... (2013) Fabrication of nature-inspired microfluidic network for perfusable tissue constructs. Advanced healthcare …, Wiley Online Library, cited by 45 (7.50 per year)
H Jin, E Dong, S Mao, M Xu, ... (2014) Locomotion modeling of an actinomorphic soft robot actuated by SMA springs. 2014 IEEE International …, ieeexplore.ieee.org, cited by 7 (1.40 per year)
D Gates (2019) Conference Aims: Educate, Stimulate, Unite., optech.ucsf.edu
C Mavoungou, IL Aldinger, ... (2017) Learning from insects: studies of nest structures. Proceedings of IASS …, ingentaconnect.com
MN Anh, TD Tang, VT Trung, LC Hieu, NH Tu, ... (2018) Cost-Effective Design and Development of a Prosthetic Hand. … Conference on the …, Springer
J Jang (2017) 3D bioprinting and in vitro cardiovascular tissue modeling. Bioengineering, mdpi.com, cited by 13 (6.50 per year)

Z Ji, C Yan, S Ma, X Zhang, X Jia, ... (2019) Biomimetic Surface with Tunable Frictional Anisotropy Enabled by Photothermogenesis-Induced Supporting Layer Rigidity Variation. Advanced Materials …, Wiley Online Library
O Garibay, E Bermudez, S O'Neil (2015) EMG Prosthetic Finger:" GoGo Finger"., digitalcommons.calpoly.edu
IT Ozbolat, M Hospodiuk (2016) Current advances and future perspectives in extrusion-based bioprinting. Biomaterials, Elsevier, cited by 345 (115.00 per year)
X Zhao, D Zhang, W Zhao, J Cai (2018) Method for producing living tissue and organ. US Patent App. 15/768,283, Google Patents
JS Cuellar, G Smit, D Plettenburg, A Zadpoor (2018) Additive manufacturing of non-assembly mechanisms. Additive Manufacturing, Elsevier, cited by 18 (18.00 per year)
A Arjun, L Saharan, Y Tadesse (2016) Design of a 3D printed hand prosthesis actuated by nylon 6-6 polymer based artificial muscles. 2016 IEEE International …, ieeexplore.ieee.org, cited by 8 (2.67 per year)
M Baker, J Manweiler (2017) Improving Our Bodies, Our Meals, and Our Gadgets. IEEE Pervasive Computing, computer.org
IH Hwang, P Mehmert, M Tran (2017) Metal Additive Manufacturing Process Simulation for the Hinge of the Engine Hood. 한국정밀공학회 학술발표대회 논문집, dbpia.co.kr, cited by 1 (0.50 per year)
L Clunie, D Livingstone, PM Rea (2015) Innovative taught MSc in Medical Visualisation and Human Anatomy. Journal of visual communication …, Taylor & Francis
AV Kruglov, IV Shvedovchenko (2017) Current state of functional prosthetic application in disabled persons with hand and finger stumps (literature review). Genij Ortopedii, ilizarov-journal.com
SS Thonte, OG Bhusnure, VG Makanikar, O Pravin, ... (2016) Smart Bioelectronics: The Future of Medicine is Electric. Imagine, ijetsr.com
D Sarig (2018) Dental prosthetic. US Patent App. 15/574,808, Google Patents
M Deckert, M Lippert, K Takagaki, A Brose, ... (2016) Fabrication of MEMS-based 3D-μ ECoG-MEAs. Current Directions in …, degruyter.com
S Wang, L Zhang, N Li, B Li, ... (2017) Topology and Shape Preserved Lightweight of Shell Models Utilizing Heat Diffusion. … Conference on Virtual …, ieeexplore.ieee.org
CJ Bettinger (2018) Recent advances in materials and flexible electronics for peripheral nerve interfaces. Bioelectronic Medicine, bioelecmed.biomedcentral.com, cited by 5 (5.00 per year)
Y Song, K Lin, S He, C Wang, S Zhang, ... (2018) Nano-biphasic calcium phosphate/polyvinyl alcohol composites with enhanced bioactivity for bone repair via low-temperature three-dimensional printing and …. International journal …, ncbi.nlm.nih.gov, cited by 4 (4.00 per year)
G Savio, S Rosso, R Meneghello, ... (2018) Geometric modeling of cellular materials for additive manufacturing in biomedical field: a review. Applied bionics and …, hindawi.com, cited by 7 (7.00 per year)
X Li, WA Serdijn, W Zheng, Y Tian, B Zhang (2015) The injectable neurostimulator: an emerging therapeutic device. Trends in biotechnology, Elsevier, cited by 15 (3.75 per year)
MJ Schroeder, MS Lloyd (2017) Tissue engineering strategies for auricular reconstruction. Journal of Craniofacial Surgery, journals.lww.com, cited by 1 (0.50 per year)
M Petrov, A Talapov, T Robertson, ... (1998) Optical 3D digitizers: Bringing life to the virtual world. IEEE Computer …, ieeexplore.ieee.org, cited by 124 (5.90 per year)

LIU Guang-fu (2001) Rapid prototyping-oriented medical model modeling [J]. Manofacturing Automation, en.cnki.com.cn, cited by 4 (0.22 per year)
H Li, YJ Tan, S Liu, L Li (2018) Three-dimensional bioprinting of oppositely charged hydrogels with super strong Interface bonding. ACS applied materials & interfaces, ACS Publications, cited by 7 (7.00 per year)
RR Jose, MJ Rodriguez, TA Dixon, ... (2016) Evolution of bioinks and additive manufacturing technologies for 3D bioprinting. ACS Biomaterials …, ACS Publications, cited by 105 (35.00 per year)
S Ozturk, H Karagoz, F Zor (2015) The Future of Plastic Surgery: Surgeon's Perspective. Journal of Craniofacial Surgery, journals.lww.com, cited by 2 (0.50 per year)
F Jelínek, R Pessers, ... (2014) DragonFlex smart steerable laparoscopic instrument. Journal of …, … .asmedigitalcollection.asme.org, cited by 35 (7.00 per year)
A Shavandi, E Jalalvandi (2019) Biofabrication of Bacterial Constructs: New Three-Dimensional Biomaterials. Bioengineering, mdpi.com
L Lin, H Zhang, L Zhao, Q Hu, M Fang (2009) Design and preparation of bone tissue engineering scaffolds with porous controllable structure. Journal of Wuhan University of …, Springer, cited by 9 (0.90 per year)
A Potnuru, M Jafarzadeh, ... (2016) 3D printed dancing humanoid robot "Buddy" for homecare. 2016 IEEE International …, ieeexplore.ieee.org, cited by 5 (1.67 per year)
E Ach, C Gey (2018) Machining tool and method for manufacturing a machining tool. US Patent App. 16/058,556, Google Patents
L Lin, A Tong, H Zhang, Q Hu, M Fang (2007) The mechanical properties of bone tissue engineering scaffold fabricating via selective laser sintering. International Conference on Life …, Springer, cited by 21 (1.75 per year)
L Li, Y Li, L Yang, F Yu, K Zhang, J Jin, ... (2019) Polydopamine coating promotes early osteogenesis in 3D printing porous Ti6Al4V scaffolds. Annals of …, researchgate.net
ID User Novel water treatment technology surfaces at Ingenuity Lab. isbe-online.org
Y Xiong, W Tian (2016) Study of Mechanical Properties and Biocompatibility of the Stents. Journal of Nanoscience and …, ingentaconnect.com, cited by 1 (0.33 per year)
K Kumar, J Liu, C Christianson, M Ali, MT Tolley, ... (2017) A biologically inspired, functionally graded end effector for soft robotics applications. Soft robotics, liebertpub.com, cited by 11 (5.50 per year)
B Zi, Y Li (2017) Conclusions in theory and practice for advancing the applications of cable-driven mechanisms. Chinese Journal of Mechanical Engineering, Springer, cited by 12 (6.00 per year)
P Carboon (2015) New member profiles. Australasian BioTechnology, search.informit.com.au
T Posser, BF de Oliveira (2019) Design for additive manufacturing applied for mass reduction of a two-stroke engine cylinder for portable machine. International Journal on Interactive Design and …, Springer
YE WANG, XP LI, MM YANG, QH WEI, ... (2015) Three dimensional fabrication custom-made bionic bone preoperative diagnosis models for orthopaedics surgeries. SCIENTIA SINICA …, engine.scichina.com, cited by 2 (0.50 per year)
S Gupta, P Kulshreshtha 3-D Bioprinting, a Science of New Possibilities and New Cures. krishisanskriti.org
XD Li (2016) Application of new materials in Industrial Design. Key Engineering Materials, Trans Tech Publ

DG Sohn, MW Hong, YY Kim, YS Cho (2015) Fabrication of dual-pore scaffolds using a combination of wire-networked molding (WNM) and non-solvent induced phase separation (NIPS) techniques. Journal of Bionic Engineering, Elsevier, cited by 3 (0.75 per year)
E Mikołajewska, M Macko, D Mikołajewski, ... (2016) Medical and military applications of 3D printing. … Wojsk Lądowych im …, yadda.icm.edu.pl, cited by 6 (2.00 per year)
ВА Плеханова (2015) Концептуальный проект организации небольшого производственного помещения с помощью цифровых 3d-форм. Современные наукоемкие технологии, top-technologies.ru, cited by 2 (0.50 per year)
ЕЮ Степанова (2016) Влияние аддитивных технологий на экономику наукоемких отраслей промышленности: роль многоуровневых интегрированных компаний. Экономические и гуманитарные науки, oreluniver.ru, cited by 3 (1.00 per year)
V Ferman, C de la Cruz, A Lemus (2015) Galileo Hand: Diseño de una prótesis biónica subactuada de bajo costo utilizando impresión 3D. CONESCAPAN, Guatemala, researchgate.net, cited by 1 (0.25 per year)
FL Palombini, W Kindlein Junior, FP Silva, ... (2017) Design, biônica e novos paradigmas: uso de tecnologias 3D para análise e caracterização aplicadas em anatomia vegetal. … RS. n. 13 (2017), p. 46 …, lume.ufrgs.br, cited by 1 (0.50 per year)
X Niu, Q Feng, M Wang, R Tan (2009) Chitosan microspheres/nano hydroxyapatite/collagen/polylactide composite. Acta Materiae Compositae Sinica, en.cnki.com.cn, cited by 5 (0.50 per year)
G Lihua (2017) 3D Printing Technology of Bionics Artificial Bone with PEEK in Temperature-fluctuating Bone Wound Remediation. Bulletin of Science and Technology, en.cnki.com.cn
ИВ Терентьева (2019) 3D PRINTING TECHNOLOGIES IN MEDICINE. ПРОБЛЕМЫ ВЗАИМОДЕЙСТВИЯ НАУКИ И …, elibrary.ru
R Hoogendijk, B Huau, S Renault, I Stelmasiak, A Villar (2013) Bionic engineering-R-madillo project., upcommons.upc.edu
A Moneada, D Satizabal, G Hoyos, ... (2017) Integration of a prototype of bionic prosthesis hand. 2017 IEEE 3rd …, ieeexplore.ieee.org
L Wenjia (2015) Innovative Design Study on 3D Printing Products Based on Bio-inspired Perspective. Art & Design Research, en.cnki.com.cn
A Capell Gràcia (2015) Design and implementation of a bionic arm., repositori.udl.cat
СА Вересовский (2018) ПРИМЕНЕНИЕ 3D-ПЕЧАТИ В ПРОТЕЗИРОВАНИИ КОНЕЧНОСТЕЙ. Вестник науки и образования, cyberleninka.ru
ดุรงค์ ฤทธิ์ ตรี ภาค, พีร ยศ ภมร ศิลป ธรรม, ... (2015) การ พิมพ์ สาม มิติ: เทคโนโลยี เปลี่ยน โลก สุขภาพ 3D Printing: Technology that Changes the Health World. … and Health Science …, ejournals.swu.ac.th
孙延刚, 梁彦超, 郗昊, 韩鑫 (2017) 鲨鱼盾鳞 3D 打印复制工艺研究. 塑料工业, airitilibrary.com
G Drolc (2018) Razrez 3D modela za potrebe 3D tiskanja., eprints.fri.uni-lj.si
XY MA, HY LIANG, LF WANG (2015) Multi-materials 3D printing application of shell biomimetic structure. Chinese Science Bulletin, engine.scichina.com
周敬展 (2018) 利用 3D 列印模具和超音波溶解技術來製作全透明且非平面微流道晶片., ir.lib.ntust.edu.tw

DK Kim, CH Park (2015) Application of Three-Dimensional Printing in Facial Plastic and Reconstructive Surgery.. Korean Journal of Otorhinolaryngology-Head …, researchgate.net
杜国庆, 孙健, 李亚莉, 陈立强, 陈晨, 邓楠, 吴雨桐, ... (2018) 3D 仿生打印组织工程骨修复下颌骨缺损. 中国组织工程研究, crter.org
B Destoop 3D-printing in ergotherapie.
HE Yong, GAO Qing, LIU An, SUN Miao, ... (2019) 3D bioprinting: from structure to function. Journal of ZheJiang …, zjujournals.com
藤田大樹, 中野亜希人, 羽田久一 (2019) Elsa: 氷を素材とした 3D プリンタの開発. 情報処理学会論文誌デジタル …, ipsj.ixsq.nii.ac.jp
王冲, 刘玉龙, 刘仁, 李治全 (2018) DLP 型 3D 打印用巯烯光敏树脂的制备与性能研究. 影像科学与光化学, yxkxyghx.org
贺永, 高庆, 刘安, 孙苗, 傅建中 (2019) 生物 3D 打印——从形似到神似. 浙江大学学报 (工学版), zjujournals.com
M Nguyen Quoc (2019) Development and characterization of hydrogel ink for vessels bioprinting., repo.pw.edu.pl
이선우, 한현정, 최민석, 이선곤, 김주형, 김용래, ... (2018) 다중노즐 3D 프린터의 모니터링 시스템을 활용한 사용자 맞춤형 근골격계 재활기기 소재에 관한 연구. 한국재활복지공학회 학술 …
Q Lian, P Zhuang, WG Bian, DC Li, ... (2015) 3D Printing and application of large-size joint os-teochondral scaffolds. Scientia Sinica …, engine.scichina.com
OA Martins, CLF de Assis, RC de Carvalho, ... INFLUÊNCIA DA ORIENTAÇÃO DE DEPOSIÇÃO E DO TIPO DE MATERIAL POLIMÉRICO NA RESISTÊNCIA MECÂNICA DE PEÇAS PRODUZIDAS POR …. cbecimat.com.br
Z Zhang, J Huo (2015) Lumbar spinal implants for degenerative lumbar spondylolisthesis: biocompatibility evaluation. Chinese Journal of Tissue Engineering …, wprim.whocc.org.cn
董明锐, 孙伟圣, 薛倩雯, 曹惠敏, 王文斌, 林贤铣 (2018) 3D 打印仿生木材吸声结构的吸声性能. 林业科学, html.rhhz.net
M Pulkkinen (2014) 3D-tulostimen käyttötestaus ja sovelluksen valmistus., theseus.fi
廖俊琳, 王少华, 陈佳, 谢红炬, 周建大 (2017) 3D 生物打印在组织工程软骨再生与重建应用中的研究进展. 中南大学学报 (医学版), xbyxb.csu.edu.cn
İ Şahin, Mİ SARI, T ŞAHİN Hızlı Prototipleme Yaklaşımı ile Ortez üretimi: Kaynak Araştırması. Düzce Üniversitesi Bilim ve Teknoloji Dergisi, dergipark.org.tr
PRC Duarte (2018) Impressão 3D de Polímeros Biocompatíveis., run.unl.pt
史长春, 胡镔, 陈定方, 陈蓉, 单斌 (2018) 聚醚醚酮 3D 打印成形工艺的仿真和实验研究. 中国机械工程, cmemo.org.cn
김한나 (2017) 3D 바이오프린팅 기술의 법적 쟁점. 한국의료법학회지
한상욱 (2018) 하이드로젤과 폴리이미드의 물리적 접착과 오가노젤 이오닉 컨덕터의 3D printing 연구., s-space.snu.ac.kr
E Nyrobtseva (2019) Řídicí systém bionické ruky., dspace.tul.cz
E Jusić (2019) Razvoj 3D modela bioničke ruke., repozitorij.vtsbj.hr

МА Киселев (2016) Компьютерное моделирование компонентов робототехнических устройств при изготовлении на 3D принтере. Машины и установки: проектирование …, maplants.elpub.ru

DBS BELALANG, KKDAN KUMBANG STUDI PARAMETRIK PADA FLAPPING-WING MA V. researchgate.net

L Secco Protesi funzionali per arti superiori in stampa 3D. Design su Misura Atti dell'Assemblea annuale della …, re.public.polimi.it

B Moller, S Berner, K Ostertag Potenziale für die strategische Weiterentwicklung von r2.

B Gál, A Németh (2019) ADDITÍV GYÁRTÁSTECHNOLÓGIÁK KATONAI ALKALMAZÁSÁNAK VIZSGÁLATA, KÜLÖNÖS TEKINTETTEL A KATONAI ELEKTRONIKA TERÜLETÉRE. Hadmérnök, hadmernok.hu

S Hayano (2016) The Latest Trend of Additive Manufacturing (3D Printer). Journal of the Society of Powder Technology, Japan, jstage.jst.go.jp

西眞一, 酒井真理, 藤井雅彦, 鈴木幸栄, 小田正明 (2016) デジタルファブリケーション技術 2015. 日本画像学会誌, jstage.jst.go.jp

LF Palombini, JCS Linden, JEA Mariath, … (2018) Design aided science: o designer como promotor de tecnologias 3D para inovação em pesquisa científica. … gráfica. v. 22, n. 3 (dez …, lume.ufrgs.br

M Fette, A Herrmann (2017) Zivile Luftfahrtindustrie im Wandel der Digitalisierung. Sonderprojekte ATZ/MTZ, Springer

K Gremban (2013) Complexity is free, but at what cost?. XVIII Congreso Argentino de Ciencias de la …, sedici.unlp.edu.ar

S Markkula (2017) Algoritminen suunnittelu muotoilijan työkaluna., aaltodoc.aalto.fi

J Junttila (2015) 3D-suunnittelun hyödyntäminen PK-yrityksen tuotekehityksessä: Case-tutkimus Kuusamon Uistin Oy., lauda.ulapland.fi

JH LIU, WT XU, SK JING (2015) Product modeling for additive manufacturing. SCIENTIA SINICA Informationis, engine.scichina.com

Z Xia, C Zhengming, T Jing, Y Pengfei (2016) Optimization method of bones based on fused deposition modeling technology. Application Research of …, en.cnki.com.cn

王博, 张雷鹏, 徐高平, 李晓白, 李垚 (2019) 仿生新材料的应用及展望. 科技导报, kjdb.org

J Piippo (2018) Teknologia ja proteesit esteettömyyden tukena., theseus.fi

J Kotta (2019) Metallien 3D-tulostusta aloittava yritys: mihin tulisi kiinnittää huomiota?., lutpub.lut.fi

李隆洲 (2015) 以智財演進為基進行 3D 列印應用於生醫領域之發展趨勢分析. 清華大學工業工程與工程管理學系學位論文, airitilibrary.com

VS Lesovik, LK Zagorodnyuk, ES Glagolev, … (2018) NATURE SIMILAR TECHNOLOGIES IN CONSTRUCTION INDUSTRY. International Journal for …, ijccse.iasv.ru

İ AKBEN International Journal of Academic Value Studies (Javstudies). researchgate.net

倪裕杰, 张肖凡, 陈克坚 (2019) 基于液晶 5CB 的静电纺丝光控形变器件. 光学仪器, opticsjournal.net

马骁勇, 梁海弋, 王联凤 (2016) 三维打印贝壳仿生结构的力学性能. 科学通报, engine.scichina.com, cited by 1 (0.33 per year)

김규영, 정용록, 박재호, 김민성, 박인규 (2018) 3D 프린팅을 이용한 마이크로채널 제작 및 신축성 전극으로의 활용. 한국정밀공학회 학술발표대회 …, dbpia.co.kr

C Lüders Organe und Implantate. researchgate.net

А В Круглов, И В Шведовченко (2017) Современное состояние вопроса функционального протезирования инвалидов с культями кисти и пальцев (обзор литературы). Гений ортопедии, cyberleninka.ru, cited by 1 (0.50 per year)

S Grundsten (2015) 3D Skanneri Juurutamise Võimalused Tallinna Tehnikakõrgkooli Mõõtetehnika Laboris., eprints.tktk.ee, cited by 1 (0.25 per year)

M Kovačić (2018) Tehnološke zabilješke: Inovativni materijali za zelenu energiju. Kemija u industriji: Časopis kemičara i kemijskih …, hrcak.srce.hr

DIFHU Simmler Vergleich von Stützstrukturen für die additive Fertigung. monarch.qucosa.de

O Avrunin, M Tymkovych, ... (2016) Побудова тривимірної моделі кровоносної системи головного мозку для задач планування і тренування проведення нейрохірургічних втручань… «ХПІ». Серія: Нові…, vestnik2079-5459.khpi.edu.ua

A Mastrorilli, M Mansouri (2019) Le projet Matrice: Conception et Prototypage., hal-enpc.archives-ouvertes.fr

JG Melo, N Villacis, C Segura, L Segura, D Loza Estudio del arte y construcción de prótesis de mano de 1 GDL para amputación parcial de mano Study of art and construction of 1 GDL hand prosthesis for …. researchgate.net
